# Associations of ABO and Rhesus D blood groups with phenome-wide disease incidence: A 41-year retrospective cohort study of 482,914 patients

**DOI:** 10.7554/eLife.83116

**Published:** 2023-03-09

**Authors:** Peter Bruun-Rasmussen, Morten Hanefeld Dziegiel, Karina Banasik, Pär Ingemar Johansson, Søren Brunak

**Affiliations:** 1 Department of Clinical Immunology, Copenhagen University Hospital Copenhagen Denmark; 2 Novo Nordisk Foundation Center for Protein Research, University of Copenhagen Copenhagen Denmark; University of Michigan United States; McGill University Canada

**Keywords:** ABO blood groups, RhD blood groups, phewas, disease-wide associations, Human

## Abstract

**Background:**

Whether natural selection may have attributed to the observed blood group frequency differences between populations remains debatable. The ABO system has been associated with several diseases and recently also with susceptibility to COVID-19 infection. Associative studies of the RhD system and diseases are sparser. A large disease-wide risk analysis may further elucidate the relationship between the ABO/RhD blood groups and disease incidence.

**Methods:**

We performed a systematic log-linear quasi-Poisson regression analysis of the ABO/RhD blood groups across 1,312 phecode diagnoses. Unlike prior studies, we determined the incidence rate ratio for each individual ABO blood group relative to all other ABO blood groups as opposed to using blood group O as the reference. Moreover, we used up to 41 years of nationwide Danish follow-up data, and a disease categorization scheme specifically developed for diagnosis-wide analysis. Further, we determined associations between the ABO/RhD blood groups and the age at the first diagnosis. Estimates were adjusted for multiple testing.

**Results:**

The retrospective cohort included 482,914 Danish patients (60.4% females). The incidence rate ratios (IRRs) of 101 phecodes were found statistically significant between the ABO blood groups, while the IRRs of 28 phecodes were found statistically significant for the RhD blood group. The associations included cancers and musculoskeletal-, genitourinary-, endocrinal-, infectious-, cardiovascular-, and gastrointestinal diseases.

**Conclusions:**

We found associations of disease-wide susceptibility differences between the blood groups of the ABO and RhD systems, including cancer of the tongue, monocytic leukemia, cervical cancer, osteoarthrosis, asthma, and HIV- and hepatitis B infection. We found marginal evidence of associations between the blood groups and the age at first diagnosis.

**Funding:**

Novo Nordisk Foundation and the Innovation Fund Denmark

## Introduction

Still 100 years after the discovery of the ABO and Rhesus D (RhD) blood group systems, the selective forces that may have attributed to the observed blood group population differences remain elusive (Anstee, 2010). The pathophysiological mechanisms behind the observed relationship between blood groups and diseases are not well understood either. The ABO system has been associated with susceptibility to multiple diseases, including gastrointestinal- and cardiovascular diseases and pancreatic-, gastric-, and ovarian cancers (Vasan et al., 2016; Liumbruno and Franchini, 2014; Wolpin et al., 2010; Groot et al., 2020; Edgren et al., 2010; Dahlén et al., 2021; Li and Schooling, 2020). The ABO system has also been associated with the susceptibility, progression, and severity of COVID-19 (Ellinghaus et al., 2020). In contrast, apart from hemolytic disease of the newborn, reported associations between the RhD blood group and disease development are sparser (Anstee, 2010).

Specifically, higher levels of factor VIII (FVIII) and von Willebrand factor (vWF) observed in individuals with a non-O blood group have been suggested to affect the development of cardiovascular disease (Jenkins and O’Donnell, 2006; Franchini and Lippi, 2015). Additionally, blood group-related antigens have been suggested to be involved in the adhesion of trophoblast, inflammatory cells, and metastatic tumor cells to the endothelial cells of the vasculature (Ravn and Dabelsteen, 2000). The endothelial cells of the vasculature have also been suggested to contribute to the initiation and propagation of severe clinical manifestations of COVID-19 (Teuwen et al., 2020).

Recently, an associative disease-wide risk analysis of the ABO and RhD blood groups was conducted in a large Swedish cohort (Dahlén et al., 2021). The study generated further support for previous findings and suggested new associations. Here, we further uncover the relationship between the ABO and RhD blood groups and disease susceptibility using a Danish cohort of 482,914 patients. In contrast to previous studies, we use up to 41 years of follow-up data, and a disease categorization scheme specifically developed for disease-wide analysis called phecodes (Wu et al., 2019). Further, we determine the uniqueness of each individual ABO blood group as opposed to using blood group O as the reference.

We estimate incidence rate ratios of 1312 phecodes (diagnoses) for the ABO and RhD blood groups. Further, we determine associations between the ABO/RhD blood groups and the age at the first diagnosis to better disclose the temporal life course element of disease development.

## Methods

### Study design

This retrospective cohort study was based on the integration of the Danish National Patient Registry (DNPR) and data on ABO/RhD blood groups of hospitalized patients. We included Danish patients who had their ABO/RhD blood group determined in the Capital Region or Region Zealand (covering ~45% of the Danish population Nordjylland, 2022), between January 1, 2006, and April 10, 2018. A blood type determination is commonly done for patients who may require a blood transfusion during hospitalization for example, anemic patients and women in labor. In the inclusion period, approximately 90% of the population in the Capital Region and 97% of the Region Zealand population were of European ancestry (Supplementary file 1). The DNPR provided the International Classification of Diseases 8^th^ and 10^th^ revision (ICD-8 and ICD-10) diagnosis codes, dates of diagnosis, date of birth, date of potential emigration, and sex of patients, with records dating back to 1977.

Similar to a case-control study, the patients were included retrospectively. Here, selection into the study was based on an in-hospital ABO/RhD blood group determination. That is, the person-time and the entire disease history back to 1977 of patients hospitalized between 2006 and 2018 with known ABO/RhD blood groups were included retrospectively.

We defined diseased and non-diseased individuals using the phecode mapping from ICD-10 diagnosis codes (Wu et al., 2019). Before categorizing the assigned ICD diagnosis codes into phecodes, the ICD-8 codes were converted to ICD-10 codes (Pedersen et al., 2023). Further, referral diagnoses were excluded. Pregnancy- and perinatal diagnosis (ICD-10 chapters 15–16) assigned before or after age 10 were excluded, or, when possible, rightly assigned to the mother or newborn, respectively. The disease categories of injuries, poisonings, and symptoms were deemed unlikely to be associated with the blood groups and excluded from the analyses (phecode categories: ‘injuries and poisonings’, ‘symptoms’ and phecodes above 999). Only phecodes with at least 100 cases in the study sample were included. The patients were followed from the entry in the DNPR to the date of death, emigration, the first event of the studied phecode, or end study period (April 10, 2018), whichever came first. Thus, follow-up was up to 41 years. The patients were allowed to contribute events and time at risk to multiple phecode analysis.

### Diagnosis-wide incidence rate ratios

We used a log-linear quasi-Poisson regression model to estimate incidence rate ratios (IRRs) of each phecode among individuals with blood groups A, B, AB, and O relative to the other blood groups, respectively (e.g. A vs. B, AB, and O) (Dewey et al., 1995; Ver Hoef and Boveng, 2007). Further, we compared individuals with positive RhD type relative to negative RhD type. The analyses of diseases developed by both males and females were adjusted for sex, while analyses of sex-restricted diseases (e.g. cervical cancer) only included a subgroup of individuals of the restricted sex. Sex-restricted diseases were pre-defined by the phecode terminology. Sex was adjusted for as prior studies have found sex differences in the incidence rates of multiple diseases (Westergaard et al., 2019). Further, we adjusted for the year of birth and attained age, both modeled using restricted cubic splines with five knots. Attained age was split into 1 year intervals and treated as a time-dependent covariate, thus allowing individuals to move between categories with time. Herewith, age was used as the underlying time scale. Further, an interaction between attained age and sex was modeled for non-sex-restricted analyses. Patients were excluded from the analysis if they were assigned the phecode under study at the start of the DNPR. For analysis of congenital phecodes (e.g. sickle cell disease), prevalence ratios were estimated instead of IRRs by using the cohort size as the offset (see Supplementary file 2 for a list of the congenital phecodes). The analyses of ABO blood groups were adjusted for RhD type, and the RhD-analyses were adjusted for the ABO blood group. Adjustment for the birth year was done to control for societal changes and was used instead of the calendar period of diagnosis. The robust quasi-Poisson variance formula was used to control for over-dispersion (Ver Hoef and Boveng, 2007). We conducted a supplemental analysis using the same methodology but where blood group O was instead used as the reference to enable direct comparison and meta-analysis with previous studies.

### Age of first hospital diagnosis

We estimated differences in age of first phecode of individuals with blood group A, B, AB, and O relative to any other blood group, respectively. Similar analyses were done for RhD-positive individuals relative to RhD negative individuals. We used a linear regression model adjusted for sex and birth year (as a restricted cubic spline with five knots). Analysis of sex-restricted phecodes was not adjusted for sex. Individuals who were assigned the studied phecode at the start date of the DNPR were excluded as the age of diagnosis was uncertain. Further, congenital- and pregnancy-related phecodes were not included.

Statistical analyses were performed in R (version 3.6.2) using the survival and rms package. p-values were two-sided. p-values and confidence intervals were adjusted for multiple testing by the false discovery rate (FDR) approach, accounting for the number of performed tests (5 blood groups times 1312 phecodes; Benjamini and Hochberg, 1995; Altman and Bland, 2011). FDR adjusted p-values <0.05 were deemed statistically significant. The analysis pipeline was made in python (anaconda3/5.3.0) using snakemake for reproducibility (Köster and Rahmann, 2012). The analyses code is available through https://www.github.com/peterbruun/blood_type_study (copy archived at Bruun-Rasmussen, 2023). The manuscript complies with the STROBE reporting guidelines.

## Results

**Figure 1.**
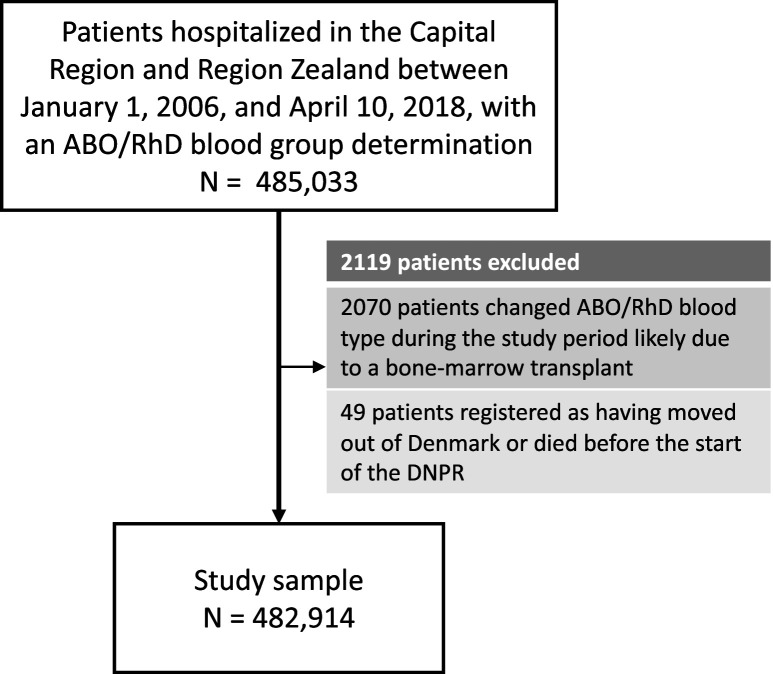
Selection of patients for the 41-year retrospective cohort study on ABO/RhD blood groups and associations with disease incidence in 482,914 Danish patients.

In total, 482,914patients (60.4%females) were included and 1312 phecodes (diagnosis codes) were examined (Figure 1, and Table 1). The median follow-up time for all phecode analyses was 17,555,322 person-years (Q1-Q3: 17,324,597–17,615,142). The cohort held a wide age distribution of patients born from 1901 to 2015 (Table 1, and Supplementary file 3). The ABO/RhD blood group distribution of the patients was similar to that of a previously summarized reference population of 2.2 million Danes (Table 1; Barnkob et al., 2020; Banks, 2022).

**Table 1.** Characteristics of patients in the 41-year retrospective cohort study on ABO/RhD blood groups and associations with disease incidence in 482,914 Danish patients.

		N=482,914
**ABO, n (%**)	**O**	197,634 (40.9)
	**A**	206,110 (42.7)
	**AB**	22,111 (4.6)
	**B**	57,059 (11.8)
**RhD, n (%**)	**Negative**	74,150 (15.4)
	**Positive**	408,764 (84.6)
**Sex, n (%**)	**K**	291,649 (60.4)
	**M**	191,265 (39.6)
**Birth year, median [Q1,Q3]**		1963 [1945,1982]
**Age at entry, median [Q1,Q3]**		13 [0,32]
**Follow-up time, median [Q1,Q3]**		40.8 [33.4,41.3]

### Incidence rate ratios

**Figure 2.**
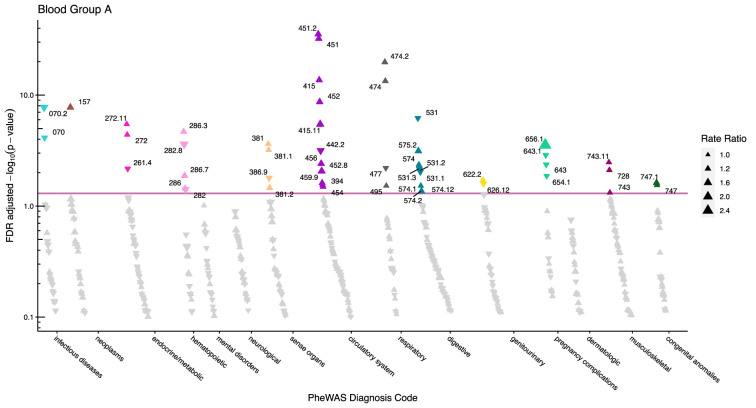
Manhattan plot for blood group A with phecodes included by category. The vertical axis shows the -log10 transformed FDR adjusted p-values on a log10-scale. The horizontal axis shows the phecodes by category. The red line indicates the statistically significant level of <0.05 for FDR adjusted p-values. Associations with p-value >0.8 are not displayed. Coloured and annotated associations were deemed statistically significant. The direction of the triangles indicates positive or inverse associations (upward: IRR >1, downward: IRR <1). The size of the triangles indicates the size of the incidence rate ratio.

**Figure 3.**
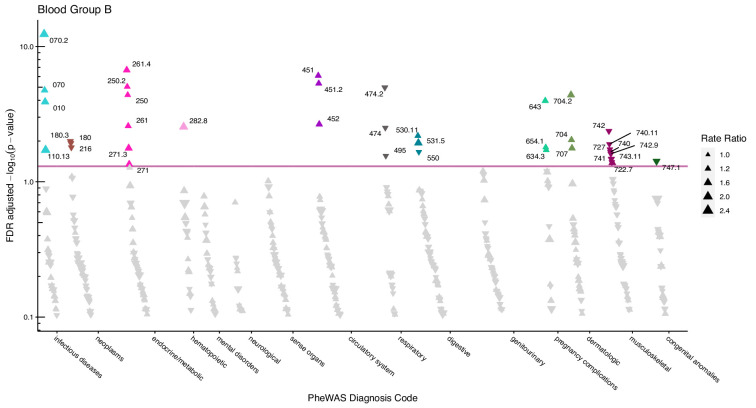
Manhattan plot for blood group B with phecodes included by category. The vertical axis shows the -log10 transformed FDR adjusted p-values on a log10-scale. The horizontal axis shows the phecodes by category. The red line indicates the statistically significant level of <0.05 for FDR adjusted p-values. Associations with p-value >0.8 are not displayed. Coloured and annotated associations were deemed statistically significant. The direction of the triangles indicates positive or inverse associations (upward: IRR >1, downward: IRR <1). The size of the triangles indicates the size of the incidence rate ratio.

**Figure 4.**
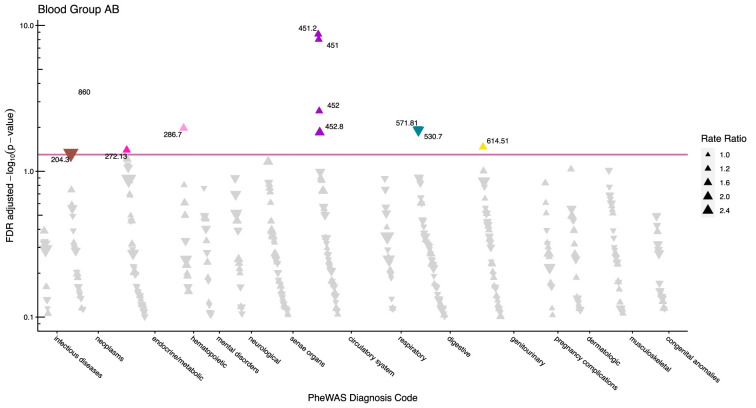
Manhattan plot for blood group AB with phecodes included by category. The vertical axis shows the -log10 transformed FDR adjusted p-values on a log10-scale. The horizontal axis shows the phecodes by category. The red line indicates the statistically significant level of <0.05 for FDR adjusted p-values. Associations with p-value >0.8 are not displayed. Coloured and annotated associations were deemed statistically significant. The direction of the triangles indicates positive or inverse associations (upward: IRR >1, downward: IRR <1). The size of the triangles indicates the size of the incidence rate ratio.

**Figure 5.**
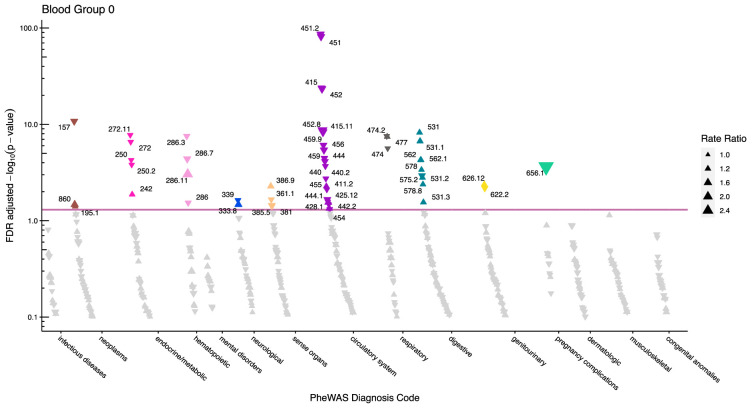
Manhattan plot for blood group O with phecodes included by category. The vertical axis shows the -log10 transformed FDR adjusted p-values on a log10-scale. The horizontal axis shows the phecodes by category. The red line indicates the statistically significant level of <0.05 for FDR adjusted p-values. Associations with p-value >0.8 are not displayed. Coloured and annotated associations were deemed statistically significant. The direction of the triangles indicates positive or inverse associations (upward: IRR >1, downward: IRR <1). The size of the triangles indicates the size of the incidence rate ratio.

**Figure 6.**
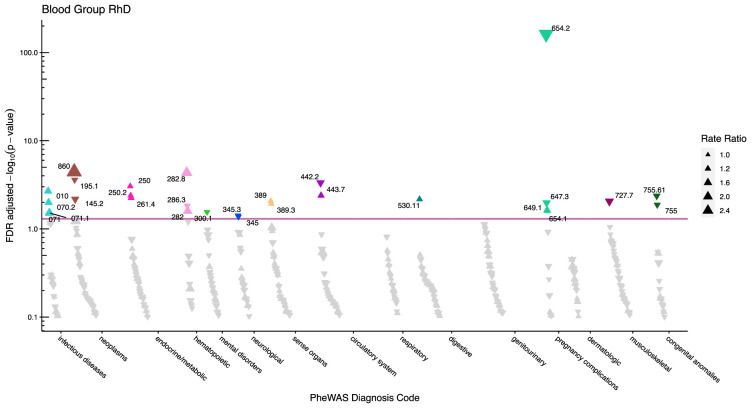
Manhattan plot for the Rhesus D blood group with phecodes included by category. The vertical axis shows the -log10 transformed FDR adjusted p-values on a log10-scale. The horizontal axis shows the phecodes by category. The red line indicates the statistically significant level of <0.05 for FDR adjusted p-values. Associations with p-value >0.8 are not displayed. Coloured and annotated associations were deemed statistically significant. The direction of the triangles indicates positive or inverse associations (upward: IRR >1, downward: IRR <1). The size of the triangles indicates the size of the incidence rate ratio.

After adjustment for multiple testing, we found the incidence rate ratios (IRRs) of 101 and 28 phecodes (116 unique) to be statistically significant for the ABO and RhD blood groups, respectively. The statistically significant IRRs are given with 95% confidence intervals in Table 2. The estimates of all examined phecodes are given in Supplementary file 4. Further, Manhattan plots of the p-values and disease categories are presented in Figures 2—6 .

**Table 2.** Statistically significant incidence rate ratios for each individual blood group A, B, AB, and O relative to any other blood group (e.g. A vs. B, AB, and O combined). Further, also for RhD-positive blood group relative to the RhD negative blood group. Table 2—source data 1.Associations between the ABO/RhD blood groups and phecode incidence rate ratios.

				Blood group A		Blood group B		Blood group AB		Blood group 0		Blood group RhD	
Phecode	Phenotype	Cases	Person-years	IRR (95% CI)	p-value	IRR (95% CI)	p-value	IRR (95% CI)	p-value	IRR (95% CI)	p-value	IRR (95% CI)	p-value
**Infectious Diseases**													
010	Tuberculosis	2101	17603440	0.87 (0.74, 1.02)	0.093	**1.4 (1.18, 1.65**)	**<0.001**	1.07 (0.32, 3.57)	0.915	0.97 (0.59, 1.57)	0.899	**1.36 (1.12, 1.65**)	**0.002**
070	Viral hepatitis	6596	17557078	**0.88 (0.82, 0.93**)	**<0.001**	**1.22 (1.12, 1.34**)	**<0.001**	1 (0.81, 1.23)	0.97	1.04 (0.9, 1.21)	0.583	1.12 (0.99, 1.28)	0.069
070.2	Viral hepatitis B	1664	17613572	**0.7 (0.62, 0.79**)	**<0.001**	**1.66 (1.46, 1.9**)	**<0.001**	1.12 (0.38, 3.26)	0.851	1.06 (0.7, 1.61)	0.794	**1.36 (1.07, 1.71**)	**0.01**
071	Human immunodeficiency virus [HIV] disease	1182	17620808	0.81 (0.63, 1.05)	0.109	1.18 (0.54, 2.59)	0.688	1.19 (0.52, 2.73)	0.693	1.1 (0.67, 1.81)	0.725	**1.49 (1.04, 2.15**)	**0.031**
071.1	HIV infection, symptomatic	1182	17620808	0.81 (0.63, 1.05)	0.109	1.18 (0.54, 2.59)	0.688	1.19 (0.52, 2.73)	0.693	1.1 (0.67, 1.81)	0.725	**1.49 (1.04, 2.15**)	**0.031**
110.13	Dermatophytosis of the body	211	17630531	0.89 (0.35, 2.22)	0.809	**1.64 (1.08, 2.49**)	**0.02**	0.93 (0.03, 30.15)	0.97	0.88 (0.35, 2.23)	0.804	1.13 (0.2, 6.46)	0.897
**Neoplasms**													
145.2	Cancer of tongue	606	17629055	0.95 (0.51, 1.76)	0.877	1.13 (0.71, 1.82)	0.616	1.21 (0.43, 3.38)	0.726	0.96 (0.46, 2.03)	0.93	**0.74 (0.6, 0.92**)	**0.007**
157	Pancreatic cancer	2828	17627948	**1.26 (1.16, 1.36**)	**<0.001**	1.02 (0.34, 3.13)	0.97	1.11 (0.53, 2.33)	0.8	**0.76 (0.7, 0.82**)	**<0.001**	1.01 (0.52, 1.98)	0.97
180	Cervical cancer and dysplasia	12538	10308860	1.05 (0.99, 1.12)	0.118	**0.9 (0.83, 0.97**)	**0.01**	0.96 (0.66, 1.39)	0.839	1 (0.99, 1.01)	0.97	0.93 (0.86, 1.01)	0.083
180.3	Cervical intraepithelial neoplasia [CIN] [Cervical dysplasia]	10895	10327107	1.06 (0.99, 1.13)	0.115	**0.89 (0.82, 0.97**)	**0.009**	0.95 (0.67, 1.34)	0.766	1 (0.84, 1.19)	0.97	0.93 (0.85, 1.01)	0.102
195.1	Malignant neoplasm, other	7383	17603424	0.96 (0.87, 1.05)	0.368	0.95 (0.77, 1.17)	0.642	0.96 (0.55, 1.69)	0.906	**1.07 (1, 1.15**)	**0.038**	**0.88 (0.82, 0.94**)	**<0.001**
204.3	Monocytic leukemia	179	17631300	1.04 (0.15, 7.27)	0.97	0.92 (0.06, 13.67)	0.957	**0.25 (0.06, 0.98**)	**0.047**	1.13 (0.44, 2.91)	0.809	0.84 (0.37, 1.93)	0.698
216	Benign neoplasm of skin	12993	17495431	1.02 (0.84, 1.23)	0.883	**0.9 (0.82, 0.98**)	**0.014**	0.99 (0.62, 1.57)	0.97	1.03 (0.93, 1.14)	0.583	0.95 (0.85, 1.05)	0.32
860	Bone marrow or stem cell transplant	142	17631302	1.01 (0.77, 1.31)	0.97	0.63 (0.15, 2.57)	0.531	**0.15 (0.05, 0.44**)	**<0.001**	**1.37 (1.02, 1.82**)	**0.033**	**4.15 (2.13, 8.09**)	**<0.001**
**Endocrine/Metabolic**													
242	Thyrotoxicosis with or without goiter	9744	17527426	0.93 (0.86, 1.02)	0.127	0.99 (0.59, 1.65)	0.97	0.9 (0.66, 1.22)	0.505	**1.09 (1.02, 1.18**)	**0.013**	1.02 (0.7, 1.48)	0.927
250	Diabetes mellitus	36810	17295033	1.01 (0.92, 1.11)	0.849	**1.09 (1.05, 1.13**)	**<0.001**	1.06 (0.95, 1.18)	0.32	**0.95 (0.92, 0.97**)	**<0.001**	**1.07 (1.03, 1.11**)	**<0.001**
250.2	Type 2 diabetes	32505	17346533	1 (0.82, 1.23)	0.97	**1.1 (1.06, 1.15**)	**<0.001**	1.07 (0.96, 1.19)	0.213	**0.94 (0.92, 0.97**)	**<0.001**	**1.07 (1.02, 1.12**)	**0.004**
261	Vitamin deficiency	6674	17594082	0.94 (0.86, 1.03)	0.183	**1.15 (1.05, 1.26**)	**0.003**	0.93 (0.66, 1.31)	0.683	1.01 (0.75, 1.36)	0.94	1.06 (0.92, 1.22)	0.405
261.4	Vitamin D deficiency	4105	17613013	**0.91 (0.85, 0.97**)	**0.007**	**1.23 (1.14, 1.34**)	**<0.001**	0.95 (0.56, 1.61)	0.86	1.01 (0.63, 1.63)	0.97	**1.14 (1.04, 1.25**)	**0.006**
271	Disorders of carbohydrate transport and metabolism	1306	17619038	0.86 (0.69, 1.07)	0.176	**1.28 (1, 1.64**)	**0.047**	1.07 (0.23, 4.92)	0.934	1.01 (0.51, 2)	0.97	1.16 (0.78, 1.72)	0.475
271.3	Intestinal disaccharidase deficiencies and disaccharide malabsorption	1154	17621337	0.85 (0.68, 1.05)	0.135	**1.32 (1.05, 1.67**)	**0.018**	1.08 (0.24, 4.88)	0.924	1.01 (0.55, 1.86)	0.97	1.15 (0.73, 1.8)	0.562
272	Disorders of lipoid metabolism	41222	17347032	**1.06 (1.03, 1.09**)	**<0.001**	1.01 (0.72, 1.42)	0.97	1.03 (0.63, 1.69)	0.92	**0.93 (0.91, 0.96**)	**<0.001**	1.02 (0.9, 1.17)	0.733
272.11	Hypercholesterolemia	35565	17395012	**1.06 (1.03, 1.09**)	**<0.001**	1.01 (0.79, 1.29)	0.956	1.03 (0.88, 1.21)	0.709	**0.93 (0.91, 0.96**)	**<0.001**	1.03 (0.96, 1.09)	0.433
272.13	Mixed hyperlipidemia	1324	17619911	1.05 (0.66, 1.67)	0.841	1.04 (0.14, 7.94)	0.97	**1.39 (1.01, 1.91**)	**0.04**	0.87 (0.7, 1.08)	0.211	1.09 (0.58, 2.04)	0.809
**Hematopoietic**													
282	Hereditary hemolytic anemias	947	482914*	0.76 (0.59, 0.99)**	**0.039**	1.36 (0.9, 2.06)**	0.141	1.42 (0.72, 2.8)**	0.316	1.03 (0.25, 4.26)**	0.97	1.65 (1.06, 2.56)**	**0.026**
282.8	Other hemoglobinopathies	557	482914*	0.65 (0.51, 0.82)**	**<0.001**	1.56 (1.16, 2.1)**	**0.003**	1.47 (0.77, 2.83)**	0.248	1.09 (0.58, 2.07)**	0.798	2.24 (1.52, 3.29)**	**<0.001**
286	Coagulation defects	4124	17606796	**1.14 (1.01, 1.3**)	**0.035**	0.95 (0.56, 1.6)	0.854	1.14 (0.67, 1.95)	0.633	**0.87 (0.76, 0.99**)	**0.029**	0.93 (0.66, 1.33)	0.717
286.11	Von willebrand’s disease	214	482914*	0.71 (0.35, 1.47)**	0.368	0.49 (0.17, 1.45)**	0.199	0.39 (0.02, 8.74)**	0.562	1.96 (1.32, 2.91)**	**<0.001**	0.68 (0.29, 1.6)**	0.388
286.3	Coagulation defects complicating pregnancy or postpartum	2015	10502418	**1.15 (1.08, 1.22**)	**<0.001**	1.02 (0.58, 1.79)	0.946	1.14 (0.95, 1.38)	0.164	**0.84 (0.79, 0.89**)	**<0.001**	**0.88 (0.8, 0.97**)	**0.013**
286.7	Other and unspecified coagulation defects	1085	17621421	**1.23 (1.05, 1.45**)	**0.013**	0.98 (0.45, 2.16)	0.97	**1.52 (1.1, 2.08**)	**0.011**	**0.74 (0.64, 0.85**)	**<0.001**	1.03 (0.23, 4.72)	0.97
**Mental Disorders**													
300.1	Anxiety disorder	7985	17603188	1.03 (0.93, 1.14)	0.575	0.97 (0.8, 1.16)	0.735	0.96 (0.66, 1.41)	0.858	0.99 (0.76, 1.29)	0.953	**0.92 (0.86, 0.99**)	**0.027**
**Neurological**													
333.8	Other degenerative diseases of the basal ganglia	381	17630209	0.78 (0.57, 1.07)	0.128	1.05 (0.11, 9.95)	0.967	0.65 (0.24, 1.78)	0.407	**1.32 (1.02, 1.71**)	**0.033**	1 (0.95, 1.05)	0.97
339	Other headache syndromes	3466	17603269	1.05 (0.9, 1.22)	0.546	1.11 (0.95, 1.3)	0.204	1.03 (0.39, 2.73)	0.957	**0.9 (0.83, 0.99**)	**0.023**	1.06 (0.82, 1.36)	0.688
345	Epilepsy, recurrent seizures, convulsions	23469	17228780	0.99 (0.77, 1.28)	0.97	0.96 (0.86, 1.08)	0.51	0.97 (0.71, 1.32)	0.853	1.03 (0.96, 1.1)	0.44	**0.94 (0.89, 1**)	**0.039**
345.3	Convulsions	14391	17351676	0.99 (0.76, 1.3)	0.97	0.96 (0.79, 1.18)	0.732	1 (0.88, 1.13)	0.97	1.02 (0.89, 1.18)	0.77	**0.92 (0.85, 0.99**)	**0.036**
**Sense Organs**													
361.1	Retinal detachment with retinal defect	3037	17594394	1.06 (0.87, 1.29)	0.582	1.07 (0.69, 1.66)	0.787	1.23 (0.9, 1.69)	0.193	**0.88 (0.79, 0.98**)	**0.022**	0.97 (0.49, 1.96)	0.948
381	Otitis media and Eustachian tube disorders	22790	17144551	**1.07 (1.03, 1.11**)	**<0.001**	0.94 (0.86, 1.04)	0.226	1.01 (0.55, 1.87)	0.97	**0.95 (0.91, 1**)	**0.039**	0.96 (0.87, 1.06)	0.437
381.1	Otitis media	12313	17364091	**1.09 (1.04, 1.14**)	**<0.001**	0.95 (0.83, 1.09)	0.449	0.98 (0.44, 2.22)	0.97	0.94 (0.89, 1)	0.067	0.97 (0.82, 1.13)	0.688
381.2	Eustachian tube disorders	2358	17571118	**1.15 (1.01, 1.31**)	**0.033**	0.9 (0.62, 1.29)	0.566	0.87 (0.38, 2.01)	0.765	0.93 (0.74, 1.17)	0.531	0.88 (0.69, 1.13)	0.32
385.5	Tympanosclerosis and middle ear disease related to otitis media	530	17625215	1.25 (0.95, 1.64)	0.114	0.99 (0.55, 1.77)	0.97	1.21 (0.37, 3.96)	0.761	**0.77 (0.6, 0.98**)	**0.036**	1.08 (0.29, 3.98)	0.918
386.9	Dizziness and giddiness (Light-headedness and vertigo)	1060	17624097	**0.84 (0.73, 0.97**)	**0.016**	1.04 (0.43, 2.55)	0.933	0.82 (0.42, 1.57)	0.555	**1.2 (1.06, 1.37**)	**0.005**	1.08 (0.7, 1.68)	0.738
389	Hearing loss	43238	17166114	0.99 (0.88, 1.12)	0.897	1.01 (0.74, 1.37)	0.97	1.01 (0.78, 1.3)	0.97	1.01 (0.83, 1.21)	0.96	**1.06 (1.01, 1.1**)	**0.009**
389.3	Degenerative and vascular disorders of ear	22354	17419947	0.99 (0.81, 1.22)	0.94	1 (0.85, 1.19)	0.97	1.02 (0.53, 1.94)	0.961	1 (0.83, 1.22)	0.97	**1.08 (1.02, 1.15**)	**0.012**
**Circulatory System**													
394	Rheumatic disease of the heart valves	8422	17577658	**1.08 (1.01, 1.16**)	**0.029**	0.98 (0.61, 1.57)	0.924	0.93 (0.63, 1.38)	0.744	0.94 (0.86, 1.03)	0.2	0.96 (0.78, 1.19)	0.729
411.2	Myocardial infarction	25905	17411193	1.03 (0.96, 1.11)	0.434	1.06 (0.97, 1.16)	0.188	1.03 (0.58, 1.8)	0.932	**0.94 (0.9, 0.98**)	**0.008**	1.02 (0.83, 1.26)	0.867
415	Pulmonary heart disease	10870	17565369	**1.2 (1.15, 1.26**)	**<0.001**	1.07 (0.9, 1.27)	0.475	1.14 (0.96, 1.36)	0.125	**0.78 (0.75, 0.82**)	**<0.001**	0.95 (0.82, 1.09)	0.482
415.11	Pulmonary embolism and infarction, acute	1533	17612465	**1.39 (1.21, 1.59**)	**<0.001**	1.11 (0.56, 2.18)	0.781	1.14 (0.44, 2.98)	0.798	**0.65 (0.57, 0.75**)	**<0.001**	0.88 (0.59, 1.32)	0.562
425.12	Other hypertrophic cardiomyopathy	466	17628820	0.81 (0.61, 1.07)	0.138	0.83 (0.35, 1.98)	0.683	1.11 (0.12, 10.23)	0.93	**1.3 (1.03, 1.64**)	**0.028**	0.91 (0.32, 2.6)	0.877
428.1	Congestive heart failure (CHF) NOS	8357	17595328	1.03 (0.94, 1.13)	0.5	1.02 (0.76, 1.37)	0.906	1.12 (0.96, 1.3)	0.14	**0.94 (0.89, 0.99**)	**0.028**	1.01 (0.7, 1.46)	0.961
440	Atherosclerosis	10901	17554704	1.05 (0.95, 1.16)	0.345	1.1 (0.96, 1.25)	0.169	1.12 (0.91, 1.37)	0.3	**0.9 (0.85, 0.95**)	**<0.001**	1.04 (0.88, 1.23)	0.675
440.2	Atherosclerosis of the extremities	8348	17570336	1.06 (0.96, 1.18)	0.258	1.09 (0.9, 1.32)	0.406	1.08 (0.75, 1.55)	0.686	**0.9 (0.84, 0.96**)	**0.002**	1.03 (0.8, 1.33)	0.823
442.2	Aneurysm of iliac artery	326	17630660	**0.75 (0.64, 0.89**)	**<0.001**	1.13 (0.63, 2.04)	0.69	1.2 (0.61, 2.38)	0.607	**1.21 (1, 1.47**)	**0.045**	**0.72 (0.6, 0.87**)	**<0.001**
443	Peripheral vascular disease	13791	17546796	1.05 (0.98, 1.12)	0.153	1.01 (0.59, 1.73)	0.97	1.03 (0.71, 1.5)	0.872	**0.94 (0.89, 1**)	**0.035**	1.03 (0.91, 1.18)	0.63
443.7	Peripheral angiopathy in diseases classified elsewhere	3677	17611414	1.02 (0.68, 1.51)	0.94	1.1 (0.9, 1.34)	0.37	1.1 (0.78, 1.55)	0.603	0.93 (0.83, 1.04)	0.214	**1.18 (1.05, 1.31**)	**0.004**
444	Arterial embolism and thrombosis	2390	17614619	1.14 (0.98, 1.34)	0.093	1.11 (0.77, 1.61)	0.583	1.28 (0.93, 1.76)	0.134	**0.79 (0.7, 0.89**)	**<0.001**	1 (0.92, 1.08)	0.97
444.1	Arterial embolism and thrombosis of lower extremity artery	1286	17623872	1.13 (0.78, 1.63)	0.533	1.13 (0.61, 2.13)	0.706	1.38 (0.86, 2.23)	0.185	**0.78 (0.63, 0.97**)	**0.022**	0.98 (0.35, 2.76)	0.97
451	Phlebitis and thrombophlebitis	16748	17479709	**1.22 (1.18, 1.25**)	**<0.001**	**1.15 (1.09, 1.21**)	**<0.001**	**1.29 (1.18, 1.4**)	**<0.001**	**0.73 (0.7, 0.75**)	**<0.001**	0.97 (0.84, 1.13)	0.717
451.2	Phlebitis and thrombophlebitis of lower extremities	15650	17489528	**1.23 (1.19, 1.27**)	**<0.001**	**1.14 (1.08, 1.2**)	**<0.001**	**1.31 (1.2, 1.42**)	**<0.001**	**0.72 (0.69, 0.74**)	**<0.001**	0.97 (0.84, 1.12)	0.678
452	Other venous embolism and thrombosis	4275	17607194	**1.24 (1.16, 1.32**)	**<0.001**	**1.21 (1.07, 1.36**)	**0.002**	**1.31 (1.1, 1.56**)	**0.003**	**0.69 (0.64, 0.74**)	**<0.001**	1 (0.89, 1.11)	0.97
452.8	Postphlebitic syndrome	341	17629425	**1.35 (1.08, 1.68**)	**0.009**	1.26 (0.76, 2.08)	0.378	**1.82 (1.13, 2.94**)	**0.014**	**0.55 (0.45, 0.67**)	**<0.001**	1.18 (0.54, 2.56)	0.688
454	Varicose veins	16500	17381971	**1.1 (1.01, 1.2**)	**0.032**	0.98 (0.57, 1.68)	0.946	1.04 (0.51, 2.1)	0.93	0.91 (0.83, 1)	0.05	0.98 (0.67, 1.42)	0.906
455	Hemorrhoids	9001	17523962	0.95 (0.84, 1.08)	0.481	0.91 (0.78, 1.08)	0.279	0.91 (0.67, 1.24)	0.562	**1.1 (1.03, 1.18**)	**0.005**	0.99 (0.67, 1.46)	0.97
456	Chronic venous insufficiency [CVI]	925	17626709	**1.24 (1.07, 1.44**)	**0.004**	1.04 (0.23, 4.7)	0.967	1.37 (0.91, 2.05)	0.131	**0.73 (0.64, 0.83**)	**<0.001**	0.94 (0.49, 1.79)	0.858
459	Other disorders of circulatory system	2555	17616713	1.12 (0.99, 1.27)	0.076	1.12 (0.85, 1.48)	0.433	1.15 (0.75, 1.75)	0.528	**0.82 (0.75, 0.9**)	**<0.001**	1.08 (0.8, 1.46)	0.636
459.9	Circulatory disease NEC	2174	17618930	**1.16 (1.02, 1.31**)	**0.024**	1.13 (0.86, 1.5)	0.381	1.22 (0.85, 1.74)	0.279	**0.78 (0.71, 0.86**)	**<0.001**	1.02 (0.38, 2.74)	0.966
**Respiratory**													
474	Acute and chronic tonsillitis	41427	16817602	**1.1 (1.08, 1.13**)	**<0.001**	**0.93 (0.88, 0.97**)	**0.002**	1.01 (0.63, 1.63)	0.97	**0.94 (0.91, 0.96**)	**<0.001**	0.97 (0.9, 1.05)	0.466
474.2	Chronic tonsillitis and adenoiditis	27077	17030480	**1.14 (1.11, 1.17**)	**<0.001**	**0.89 (0.84, 0.93**)	**<0.001**	1.02 (0.75, 1.4)	0.897	**0.92 (0.89, 0.94**)	**<0.001**	0.96 (0.89, 1.03)	0.259
477	Epistaxis or throat hemorrhage	12337	17506794	**0.93 (0.89, 0.98**)	**0.006**	0.92 (0.83, 1.02)	0.118	0.94 (0.71, 1.23)	0.648	**1.12 (1.08, 1.16**)	**<0.001**	1.01 (0.73, 1.41)	0.948
495	Asthma	31106	17238738	**1.04 (1, 1.08**)	**0.028**	**0.94 (0.88, 0.99**)	**0.023**	1 (1, 1)	0.97	0.99 (0.9, 1.08)	0.777	0.99 (0.78, 1.27)	0.97
**Digestive**													
530.11	GERD	7461	17582158	0.99 (0.69, 1.43)	0.966	**1.12 (1.03, 1.22**)	**0.007**	1.04 (0.62, 1.72)	0.897	0.95 (0.88, 1.03)	0.223	**1.12 (1.03, 1.21**)	**0.007**
530.7	Gastroesophageal laceration-hemorrhage syndrome	597	17623900	1.16 (0.8, 1.66)	0.439	1.04 (0.15, 7.23)	0.97	**0.43 (0.22, 0.83**)	**0.012**	0.94 (0.44, 2.02)	0.883	1.09 (0.39, 2.99)	0.883
531	Peptic ulcer (excl. esophageal)	16678	17443704	**0.9 (0.87, 0.94**)	**<0.001**	1 (0.94, 1.07)	0.97	0.92 (0.75, 1.14)	0.471	**1.12 (1.08, 1.16**)	**<0.001**	1.02 (0.83, 1.25)	0.86
531.1	Hemorrhage from gastrointestinal ulcer	6277	17583751	**0.91 (0.84, 0.98**)	**0.009**	0.89 (0.75, 1.04)	0.149	0.91 (0.6, 1.4)	0.688	**1.17 (1.11, 1.25**)	**<0.001**	1.07 (0.91, 1.27)	0.421
531.2	Gastric ulcer	6745	17555312	**0.91 (0.85, 0.98**)	**0.008**	1.01 (0.63, 1.63)	0.97	0.92 (0.62, 1.36)	0.681	**1.11 (1.04, 1.18**)	**0.002**	0.97 (0.73, 1.28)	0.838
531.3	Duodenal ulcer	4534	17555684	**0.88 (0.8, 0.96**)	**0.007**	1.06 (0.71, 1.57)	0.803	0.97 (0.21, 4.56)	0.97	**1.12 (1.01, 1.24**)	**0.028**	1.05 (0.77, 1.43)	0.789
531.5	Gastrojejunal ulcer	586	17628539	0.83 (0.61, 1.11)	0.213	**1.41 (1.08, 1.84**)	**0.012**	1.12 (0.23, 5.41)	0.897	1.01 (0.64, 1.6)	0.97	0.85 (0.56, 1.28)	0.439
550	Abdominal hernia	47761	16976073	1.01 (0.94, 1.09)	0.757	**0.94 (0.89, 0.99**)	**0.021**	0.97 (0.81, 1.17)	0.765	1.02 (0.96, 1.08)	0.575	1.01 (0.77, 1.32)	0.97
562	Diverticulosis and diverticulitis	16569	17515568	0.94 (0.87, 1.03)	0.19	0.92 (0.82, 1.03)	0.138	0.91 (0.7, 1.18)	0.481	**1.11 (1.06, 1.17**)	**<0.001**	0.98 (0.75, 1.28)	0.884
562.1	Diverticulosis	16569	17515568	0.94 (0.87, 1.03)	0.19	0.92 (0.82, 1.03)	0.138	0.91 (0.7, 1.18)	0.481	**1.11 (1.06, 1.17**)	**<0.001**	0.98 (0.75, 1.28)	0.884
571.81	Portal hypertension	1101	17627608	0.94 (0.66, 1.35)	0.766	1.17 (0.83, 1.63)	0.376	**0.62 (0.43, 0.9**)	**0.011**	1.06 (0.76, 1.47)	0.746	0.99 (0.52, 1.88)	0.97
574	Cholelithiasis and cholecystitis	31530	17299206	**1.05 (1.02, 1.09**)	**0.004**	0.98 (0.85, 1.13)	0.766	0.99 (0.64, 1.53)	0.97	0.96 (0.92, 1)	0.062	1.01 (0.73, 1.39)	0.957
574.1	Cholelithiasis	24028	17364917	**1.05 (1.01, 1.1**)	**0.029**	0.98 (0.81, 1.18)	0.809	0.99 (0.49, 1.96)	0.97	0.96 (0.91, 1.02)	0.183	1.01 (0.75, 1.35)	0.97
574.12	Cholelithiasis with other cholecystitis	2650	17605642	**1.12 (1, 1.25**)	**0.044**	0.88 (0.69, 1.12)	0.32	0.91 (0.48, 1.7)	0.77	0.95 (0.76, 1.2)	0.688	0.96 (0.61, 1.52)	0.878
574.2	Calculus of bile duct	8401	17560937	**1.07 (1, 1.15**)	**0.042**	0.95 (0.78, 1.17)	0.651	0.98 (0.46, 2.06)	0.953	0.95 (0.87, 1.05)	0.33	1.07 (0.94, 1.21)	0.32
575.2	Obstruction of bile duct	1593	17626684	**1.24 (1.1, 1.41**)	**<0.001**	0.97 (0.28, 3.43)	0.97	1.04 (0.18, 5.91)	0.97	**0.8 (0.7, 0.91**)	**0.001**	1.1 (0.71, 1.69)	0.688
578	Gastrointestinal hemorrhage	20111	17502200	0.96 (0.9, 1.03)	0.296	0.94 (0.83, 1.08)	0.393	0.91 (0.78, 1.07)	0.249	**1.08 (1.03, 1.12**)	**<0.001**	1.01 (0.77, 1.33)	0.93
578.8	Hemorrhage of rectum and anus	11095	17555331	0.96 (0.87, 1.06)	0.413	0.93 (0.79, 1.1)	0.395	0.92 (0.71, 1.18)	0.505	**1.09 (1.03, 1.15**)	**0.004**	1 (0.8, 1.26)	0.97
**Genitourinary**													
614.51	Cervicitis and endocervicitis	660	10488791	0.97 (0.52, 1.8)	0.918	1.1 (0.7, 1.73)	0.696	**1.4 (1.03, 1.91**)	**0.033**	0.93 (0.64, 1.34)	0.706	1.16 (0.86, 1.57)	0.326
622.2	Mucous polyp of cervix	1401	10498802	**1.17 (1.03, 1.34**)	**0.02**	0.99 (0.64, 1.53)	0.97	1.12 (0.56, 2.21)	0.766	**0.83 (0.73, 0.95**)	**0.007**	0.97 (0.43, 2.18)	0.948
626.12	Excessive or frequent menstruation	10504	10375823	**0.92 (0.86, 0.99**)	**0.026**	0.96 (0.74, 1.24)	0.757	1.02 (0.48, 2.14)	0.97	**1.1 (1.03, 1.17**)	**0.004**	0.97 (0.76, 1.23)	0.811
**Pregnancy Complications**													
634.3	Ectopic pregnancy	5034	10426967	0.97 (0.87, 1.08)	0.592	**1.11 (1.01, 1.21**)	**0.022**	1.11 (0.93, 1.34)	0.25	0.96 (0.86, 1.08)	0.524	0.99 (0.65, 1.5)	0.957
643	Excessive vomiting in pregnancy	4314	10470323	**0.91 (0.85, 0.97**)	**0.005**	**1.17 (1.08, 1.27**)	**<0.001**	1.12 (0.83, 1.51)	0.48	1 (0.87, 1.14)	0.97	1 (0.89, 1.11)	0.97
643.1	Hyperemesis gravidarum	3558	10488446	**0.9 (0.84, 0.96**)	**0.002**	1.15 (0.97, 1.37)	0.105	1.11 (0.91, 1.37)	0.313	1.01 (0.76, 1.36)	0.927	1.05 (0.78, 1.4)	0.777
647.3	Major puerperal infection	274	10504689	1.04 (0.34, 3.13)	0.952	1.37 (0.98, 1.92)	0.068	0.78 (0.25, 2.45)	0.688	0.86 (0.59, 1.25)	0.439	**0.72 (0.56, 0.93**)	**0.01**
649.1	Diabetes or abnormal glucose tolerance complicating pregnancy	5053	10480682	0.92 (0.64, 1.33)	0.679	1.22 (0.95, 1.56)	0.118	1.17 (0.75, 1.82)	0.504	0.95 (0.55, 1.64)	0.866	**1.26 (1.03, 1.55**)	**0.026**
654.1	Abnormality of organs and soft tissues of pelvis complicating pregnancy, childbirth, or the puerperium	5842	10479533	**0.94 (0.89, 0.99**)	**0.016**	**1.09 (1.01, 1.16**)	**0.019**	0.93 (0.76, 1.13)	0.467	1.04 (0.96, 1.12)	0.378	**1.08 (1.01, 1.16**)	**0.03**
654.2	Rhesus isoimmunization in pregnancy	808	10503190	1.12 (0.91, 1.38)	0.294	0.8 (0.61, 1.06)	0.118	0.82 (0.4, 1.71)	0.612	1.01 (0.65, 1.57)	0.97	**0.26 (0.24, 0.29**)	**<0.001**
656.1	Isoimmunization of fetus or newborn	508	482914*	2.6 (1.57, 4.33)**	**<0.001**	1.57 (0.53, 4.61)**	0.42	0.4 (0.01, 11.01)**	0.599	0.24 (0.11, 0.51)**	**<0.001**	1.03 (0.21, 5.13)**	0.97
**Dermatologic**													
704	Diseases of hair and hair follicles	4102	17578353	0.99 (0.62, 1.59)	0.97	**1.18 (1.04, 1.34**)	**0.01**	1.03 (0.25, 4.3)	0.97	0.93 (0.82, 1.06)	0.273	1.04 (0.68, 1.59)	0.876
704.2	Hirsutism	1778	17608888	0.94 (0.7, 1.26)	0.688	**1.32 (1.15, 1.5**)	**<0.001**	1.26 (0.96, 1.65)	0.093	0.88 (0.75, 1.04)	0.13	1.13 (0.87, 1.45)	0.369
707	Chronic ulcer of skin	2121	17617533	0.94 (0.74, 1.19)	0.617	**1.23 (1.04, 1.47**)	**0.017**	0.94 (0.29, 3.07)	0.927	0.98 (0.51, 1.88)	0.959	1.01 (0.59, 1.74)	0.97
**Musculoskeletal**													
722.7	Intervertebral disc disorder with myelopathy	747	17625461	0.95 (0.61, 1.47)	0.82	**1.26 (1.01, 1.59**)	**0.042**	0.65 (0.31, 1.35)	0.248	1.01 (0.58, 1.76)	0.97	0.97 (0.28, 3.31)	0.961
727	Other disorders of synovium, tendon, and bursa	30487	17272753	1.02 (0.95, 1.09)	0.627	**0.94 (0.89, 0.99**)	**0.016**	0.99 (0.57, 1.7)	0.97	1.01 (0.92, 1.11)	0.809	0.97 (0.9, 1.04)	0.352
727.7	Contracture of tendon (sheath)	391	17628006	0.91 (0.45, 1.84)	0.8	1.14 (0.38, 3.43)	0.826	1.11 (0.05, 24.01)	0.951	1.02 (0.46, 2.25)	0.97	**0.65 (0.47, 0.9**)	**0.009**
728	Disorders of muscle, ligament, and fascia	11338	17510763	**1.08 (1.02, 1.13**)	**0.008**	0.93 (0.83, 1.04)	0.199	0.88 (0.74, 1.05)	0.164	0.98 (0.85, 1.12)	0.766	0.93 (0.85, 1.01)	0.085
740	Osteoarthrosis	53711	17187748	1.01 (0.94, 1.09)	0.815	**0.95 (0.9, 0.99**)	**0.021**	1.02 (0.79, 1.31)	0.911	1.01 (0.93, 1.1)	0.838	0.97 (0.92, 1.01)	0.132
740.11	Osteoarthrosis, localized, primary	40386	17333061	1.01 (0.94, 1.09)	0.779	**0.94 (0.9, 0.99**)	**0.012**	0.99 (0.72, 1.35)	0.94	1.01 (0.95, 1.08)	0.663	0.97 (0.92, 1.02)	0.188
741	Symptoms and disorders of the joints	5085	17578450	0.99 (0.66, 1.48)	0.97	**0.85 (0.73, 0.99**)	**0.032**	0.93 (0.52, 1.69)	0.83	1.09 (0.99, 1.2)	0.072	1 (0.82, 1.24)	0.97
742	Derangement of joint, non-traumatic	16652	17438176	1.03 (0.96, 1.11)	0.357	**0.9 (0.83, 0.97**)	**0.004**	0.98 (0.61, 1.56)	0.924	1.02 (0.88, 1.17)	0.838	0.95 (0.87, 1.04)	0.291
742.9	Other derangement of joint	13669	17490182	1.02 (0.9, 1.16)	0.757	**0.91 (0.83, 0.99**)	**0.023**	0.98 (0.47, 2.06)	0.97	1.02 (0.91, 1.15)	0.714	0.95 (0.86, 1.04)	0.26
743	Osteoporosis, osteopenia and pathological fracture	15875	17536424	**1.05 (1, 1.11**)	**0.049**	0.94 (0.85, 1.05)	0.26	0.92 (0.76, 1.12)	0.405	0.99 (0.85, 1.14)	0.858	1 (0.98, 1.02)	0.97
743.11	Osteoporosis NOS	13633	17553976	**1.06 (1.02, 1.1**)	**0.003**	**0.93 (0.87, 1**)	**0.039**	0.94 (0.78, 1.13)	0.514	0.98 (0.9, 1.07)	0.68	1 (0.82, 1.21)	0.97
**Congenital Anomalies**													
747	Cardiac and circulatory congenital anomalies	15297	482914*	1.07 (1.01, 1.14)**	**0.029**	0.94 (0.8, 1.11)**	0.475	1.02 (0.47, 2.23)**	0.961	0.95 (0.88, 1.03)**	0.223	0.98 (0.77, 1.25)**	0.884
747.1	Cardiac congenital anomalies	1621	482914*	1.19 (1.02, 1.39)**	**0.023**	0.77 (0.6, 0.98)**	**0.036**	1.03 (0.27, 3.86)**	0.97	0.93 (0.69, 1.25)**	0.635	1.02 (0.48, 2.16)**	0.97
755	Congenital anomalies of limbs	6557	482914*	0.99 (0.65, 1.5)**	0.957	0.96 (0.71, 1.29)**	0.778	0.96 (0.46, 1.97)**	0.91	1.04 (0.89, 1.23)**	0.627	0.88 (0.8, 0.97)**	**0.013**
755.61	Congenital hip dysplasia and deformity	2823	482914*	1.01 (0.57, 1.78)**	0.97	0.87 (0.66, 1.16)**	0.355	0.89 (0.42, 1.88)**	0.766	1.07 (0.85, 1.35)**	0.57	0.81 (0.71, 0.94)**	**0.004**

a. Statistically significant IRRs are marked with bold (FDR adjusted *P*-value <0.05).

b. The IRRs are adjusted for age, sex, interaction between age and sex, and birth year.

c. All other ABO blood groups and the RhD negative blood group was used as a reference, respectively.

d. FDR-adjusted p-values and 95% confidence intervals are presented.

e. FDR adjusted p-values above 0.97 were set to 0.97 to avoid exploding adjusted confidence intervals.

e. Phecodes are divided by PheWAS disease categories.

f. The number of events and the follow-up time in person-years for each phecode is presented.

g. For study results of congenital phecodes estimates marked with ** are prevalence ratios instead of IRRs and the corresponding person-year marked with * are the size of the cohort.

The number of statistically significant IRRs for A, B, AB, O, and RhD were 50, 38, 11, 53, and 28, respectively. However, a between blood group comparison on the number of statistically significant IRRs is problematic because the analyses of blood group A and O had the highest power given that these blood groups were most frequent in the study sample (Table 1). For 13 phecodes, an association was found for both the ABO blood group and the RhD blood group. The ABO blood groups were found positively associated with 75 phecodes and inversely associated with 67 phecodes. The RhD-positive blood group was found to have 16 positive- and 12 inverse associations. Blood groups A and O were associated with diseases of the circulatory and digestive system. Blood group B was associated with several infectious, metabolic, and musculoskeletal diseases. The associations of the RhD blood group included cancers, infectious diseases, and pregnancy complications. The results of the supplementary analyses where blood group O was used as the reference is shown in Supplementary files 6 and 7.

### Age at first diagnosis

We found the B blood group to be associated with a later diagnosis of viral infection. Further, blood group O was associated with a later diagnosis of phlebitis and thrombophlebitis (Table 3 and Supplementary file 5). The RhD-positive group was associated with a later diagnosis of acute and chronic tonsilitis diagnosis.

**Table 3.** Statistically significant associations between the ABO/RhD blood groups and the age of the first diagnosis. Table 3—source data 1.Associations between the ABO/RhD blood groups and the age of the first diagnosis.

			Blood group A		Blood group B		Blood group AB		Blood group 0		Blood group RhD	
Phecode	Phenotype	N	Estimate (95% CI)	p-value	Estimate (95% CI)	p-value	Estimate (95% CI)	p-value	Estimate (95% CI)	p-value	Estimate (95% CI)	p-value
079	Viral infection	25075	–0.26 (–3.06, 2.54)	0.864	**0.92 (0.13, 1.71**)	**0.022**	0.53 (–6.18, 7.23)	0.887	–0.23 (–3.22, 2.75)	0.887	0.27 (–5.22, 5.75)	0.93
451	Phlebitis and thrombophlebitis	16748	–**0.58 (–1.02,–0.13**)	**0.011**	–0.24 (–5.71, 5.22)	0.936	–0.6 (–5.87, 4.66)	0.833	**0.91 (0.57, 1.25**)	**<0.001**	0.01 (–0.33, 0.34)	0.97
451.2	Phlebitis and thrombophlebitis of lower extremities	15650	–0.53 (–1.08, 0.01)	0.055	–0.27 (–5.85, 5.31)	0.93	–0.7 (–6.35, 4.95)	0.82	**0.9 (0.55, 1.25**)	**<0.001**	0.08 (–3.89, 4.06)	0.97
474	Acute and chronic tonsillitis	41428	–0.29 (–1.45, 0.87)	0.634	0.42 (–1.97, 2.8)	0.744	0.38 (–5.71, 6.48)	0.909	0.05 (–2.15, 2.24)	0.97	**0.67 (0.15, 1.19**)	**0.011**
474.1	Acute tonsillitis	18162	0.1 (–4.42, 4.61)	0.97	0.41 (–7.17, 7.98)	0.923	0.75 (–7.23, 8.74)	0.864	–0.42 (–3.76, 2.93)	0.82	**1.34 (0.64, 2.04**)	**<0.001**

a. Statistically significant effect estimates are marked with bold (FDR adjusted *P*-value <0.05).

b. FDR adjusted p-values and 95% confidence intervals are presented.

c. FDR adjusted p-values above 0.97 were set to 0.97 to enable estimation of adjusted confidence intervals.

d. Estimates represent increases or decreases in years of age of first diagnosis.

## Discussion

We found the ABO/RhD blood groups to be associated with a wide spectrum of diseases including cancers and musculoskeletal-, genitourinary-, endocrinal-, infectious-, cardiovascular-, and gastrointestinal diseases. Associations of the ABO blood groups included monocytic leukemia, tonsilitis, renal dialysis, diseases of the female reproductive system, and osteoarthrosis. Associations of the RhD blood group included cancer of the tongue, malignant neoplasm (other), tuberculosis-, HIV-, hepatitis B infection, type 2 diabetes, hereditary hemolytic anemias, major puerperal infection, anxiety disorders, and contracture of tendon.

The blood groups may reflect their corresponding genetic markers; thus, our findings may indicate an association between disease and the ABO locus on chromosome 9 and the RH locus on chromosome 1, respectively. Alternatively, the associations may indicate that the blood groups are involved in disease mechanisms at the molecular level mediated either through the blood group antigens or by the blood group reactive antibodies. However, our findings have a compromised causal interpretation given the retrospective inclusion of individuals (and person-time) after an in-hospital blood group test.

Our results support several previously observed associations including positive associations between the non-O blood groups and prothrombotic diseases of the circulatory system (phecodes: 411.1–459.9), associations with gastroduodenal ulcers, associations of blood group O and lower risk of type 2 diabetes, and positive association between blood group B and tuberculosis (Vasan et al., 2016; Edgren et al., 2010; Dahlén et al., 2021; Fagherazzi et al., 2015; Rao, 2012). Further, our results support findings associating non-O blood groups with increased risk of pancreatic cancer (Liumbruno and Franchini, 2014). The role of the ABO blood group in HIV susceptibility remains controversial; we only observed a positive association for the RhD-positive blood group (Davison et al., 2020).

We found blood group B to be positively associated with ‘ectopic pregnancy’, ‘excessive vomiting in pregnancy, and ‘abnormality of organs and soft tissues of pelvis complicating pregnancy’ indicating that blood group B mothers may be more likely to experience pregnancy complications. Further, we found positive associations of blood group A with both ‘mucous polyp of cervix’, and blood group AB with ‘cervicitis and endocervicitis’. Taken together these findings may indicate that the ABO blood groups are associated with diseases of the female reproductive system. However, the study design does not allow for any causal interpretation.

Only a few statistically significant associations were found for the analyses of the age of the first diagnosis; thus, indicating that the blood group’s involvement in disease onset may be marginal. However, we assumed a linear relationship with age because assessing potential non-linear relationships for each disease would be unfeasible given the large number of tests performed. The linearity assumption may not hold for all analyses which limits the interpretation of the estimates.

A strength of our approach is that we utilized the phecode disease classification scheme that is specifically developed for disease-wide risk analyses (Wu et al., 2019) The phecode mapping scheme combines ICD-10 codes that clinical domain experts have deemed to cover the same disease. For example, respiratory tuberculosis (A16), tuberculosis of nervous system (A17), and miliary tuberculosis (A19), are combined into the phecode tuberculosis (phecode 10). Phecodes may therefore provide increased power and precision compared with using ICD-10 categories (Denny et al., 2010). Further, contrary to previous studies, we compared each blood group to all other blood groups, instead of determining effect estimates relative to blood group O. Thus, here we better capture the uniqueness of each individual ABO blood group.

### Limitations

Our study has some important limitations, firstly, the retrospective inclusion of patients and person-time may have introduced an immortal time bias from deaths before enrollment (in-hospital ABO/RhD blood group test) (Yadav and Lewis, 2021). The findings are therefore conditioned on patients surviving until the enrollment period. This implies, for example, that if a specific blood group causes a higher incidence of a deadly disease, then patients with such blood group are more likely to have died before enrollment, and therefore fewer individuals having both that blood group and the disease will be present in our cohort. If so, the direction of the estimates for deadly diseases strongly related to any blood group will have been lowered or even flipped, relative to any causal relationship. The study design, however, enabled 41 year of follow-up and was deemed reasonable because the blood groups have not been associated with mortality differences. Moreover, the blood group distribution in our cohort was found to be almost identical to a reference population of 2.2 million Danish blood donors. Further, we replicated several findings of associations between the blood groups and severe diseases, including pancreatic cancer (Vasan et al., 2016; Liumbruno and Franchini, 2014). This may indicate that the potential bias was less prevalent. Further, by controlling for year of birth, the potential effects of immortal time bias were likely reduced, however, this could not be tested. Immortal time biases are potentially applicable in many biobanks studies, e.g. when using the UK Biobank for retrospective studies (Yadav and Lewis, 2021).

The generalizability of our findings is limited further because our cohort solely included hospitalized patients with known ABO and RhD blood groups. These are patients whom the treating doctor has deemed likely to potentially require a blood transfusion during hospitalization. The patients under study might therefore suffer from other diseases than patients without a determined blood group, and than never hospitalized individuals. Further, diseases that do not require hospitalization could not be examined. If the effect sizes are modified by factors which are more common in our cohort than in the general population then the estimates may not be generalizable. However, it is unclear if such effect modifier exists. Lastly, it was not possible to adjust for possible confounding from the geographical distribution or ethnicity of the patients (Anstee, 2010). This may have biased some estimates because the distribution of blood groups varies between ethnicities while ethnicity is also associated with differences in disease susceptibility. Particularly, ethnicity has been associated with differences in prevalence of infectious-, cardiovascular-, sickle cell disease, and thasalamia (Kurian and Cardarelli, 2007; McQuillan et al., 2004). Thus, the estimate of these disease groups should be interpreted with caution. The Danish population is however quite homogenous and approximately 94% of Danes have European ancestry (Supplementary file 1). Therefore, a potential bias from ethnicity may be less prevalent in our cohort as compared with studies in populations of more admixed origin.

In conclusion, we found the ABO/RhD blood groups to be associated with a wide spectrum of diseases, including cardiovascular-, infectious-, gastrointestinal- and musculoskeletal diseases. This may indicate that some of the potential selective pressure on the blood groups can be attributed to disease susceptibility differences. We found few associations between the blood groups and age of first diagnosis.

## Data Availability

Anonymized patient data was used in this study. Due to national and EU regulations, the data cannot be shared with the wider research community. However, data can be accessed upon relevant application to the Danish authorities. The Danish Patient Safety Authority and the Danish Health Data Authority have permitted the use of the data in this study; whilst currently, the appropriate authority for journal data use in research is the regional committee ("Regionsråd"). The statistical summary data used to create the tables and graphs are available as Table 2—source data 1 and Table 3—source data 1. The analysis code is publicly available through https://www.github.com/peterbruun/blood_type_study (copy archived at Bruun-Rasmussen, 2023).

## Additional files

Supplementary file 1. Population at the first day of the quarter by region and country of origin.

Supplementary file 2. List of Phecodes defined as congenital or hereditary.

Supplementary file 3. The study sample birth year distribution.

Supplementary file 4. Associations between the ABO/RhD blood groups and Phecode incidence rate ratios of all analyzed Phecodes. a. Statistically significant IRRs are marked with bold (FDR adjusted *P*-value <0.05). b. The IRRs are adjusted for age, sex, interaction between age and sex, and birth year. c. All other ABO blood groups and the RhD negative blood group was used as a reference, respectively. d. The FDR adjusted p-values and 95% confidence intervals are presented. e. FDR adjusted p-values above 0.97 were set to 0.97 to avoid exploding adjusted confidence intervals. e. Phecodes are divided by PheWAS disease categories. f. The number of events and the follow-up time in person-years for each Phecode is also presented. g. For study results of congenital Phecodes estimates marked with ** are prevalence ratios instead of IRRs and the corresponding person-year marked with * are the size of the cohort.

Supplementary file 5. Associations between ABO/RhD blood groups and the age of the first diagnosis. a. Statistically significant effect estimates are marked with bold (FDR adjusted *P*-value <0.05). b. The FDR adjusted p-values and 95% confidence intervals are presented. c. FDR adjusted p-values above 0.97 were set to 0.97 to enable estimation of adjusted confidence intervals. d. Estimates represent increases or decreases in years of age of first diagnosis.

Supplementary file 6. Statistically significant associations for blood groups A, B and AB relative to blood group O. Further, also for RhD positive blood group relative to the RhD negative blood group. a. Statistically significant IRRs are marked with bold (FDR adjusted *P*-value <0.05). b. The IRRs are adjusted for age, sex, interaction between age and sex, and birth year. c. Blood group O and the RhD negative blood group was used as a reference, respectively. d. The FDR adjusted p-values and 95% confidence intervals are presented. e. FDR adjusted p-values above 0.97 were set to 0.97 to avoid exploding adjusted confidence intervals. f. Phecodes are divided by PheWAS disease categories. g. The number of events and the follow-up time in person-years for each Phecode is also presented. h. For study results of congenital Phecodes estimates marked with ** are prevalence ratios instead of IRRs and the corresponding person-year marked with * are the size of the cohort.

Supplementary file 7. Associations between the ABO/RhD blood groups and Phecode incidence rate ratios of all analyzed Phecodes with blood group O and RhD negative as the reference, respectively. a. Statistically significant IRRs are marked with bold (FDR adjusted *P*-value <0.05). b. The IRRs are adjusted for age, sex, interaction between age and sex, and birth year. c. Blood group O and the RhD negative blood group was used as a reference, respectively. d. The FDR adjusted p-values and 95% confidence intervals are presented. e. FDR adjusted p-values above 0.97 were set to 0.97 to avoid exploding adjusted confidence intervals. f. Phecodes are divided by PheWAS disease categories. g. The number of events and the follow-up time in person-years for each Phecode is also presented. h. For study results of congenital Phecodes estimates marked with ** are prevalence ratios instead of IRRs and the corresponding person-year marked with * are the size of the cohort.

MDAR checklist
