# Supplementary figures and images for: Associations of ABO and Rhesus D blood groups with phenome-wide disease incidence: A 41-year retrospective cohort study of 482,914 patients
Source: eLife. 2023 Mar 9;12:e83116. doi: 10.7554/eLife.83116 (PMC10042530; doi:10.7554/eLife.83116)

## Supplementary file 1: Population at the first day of the quarter by region and country of origin.


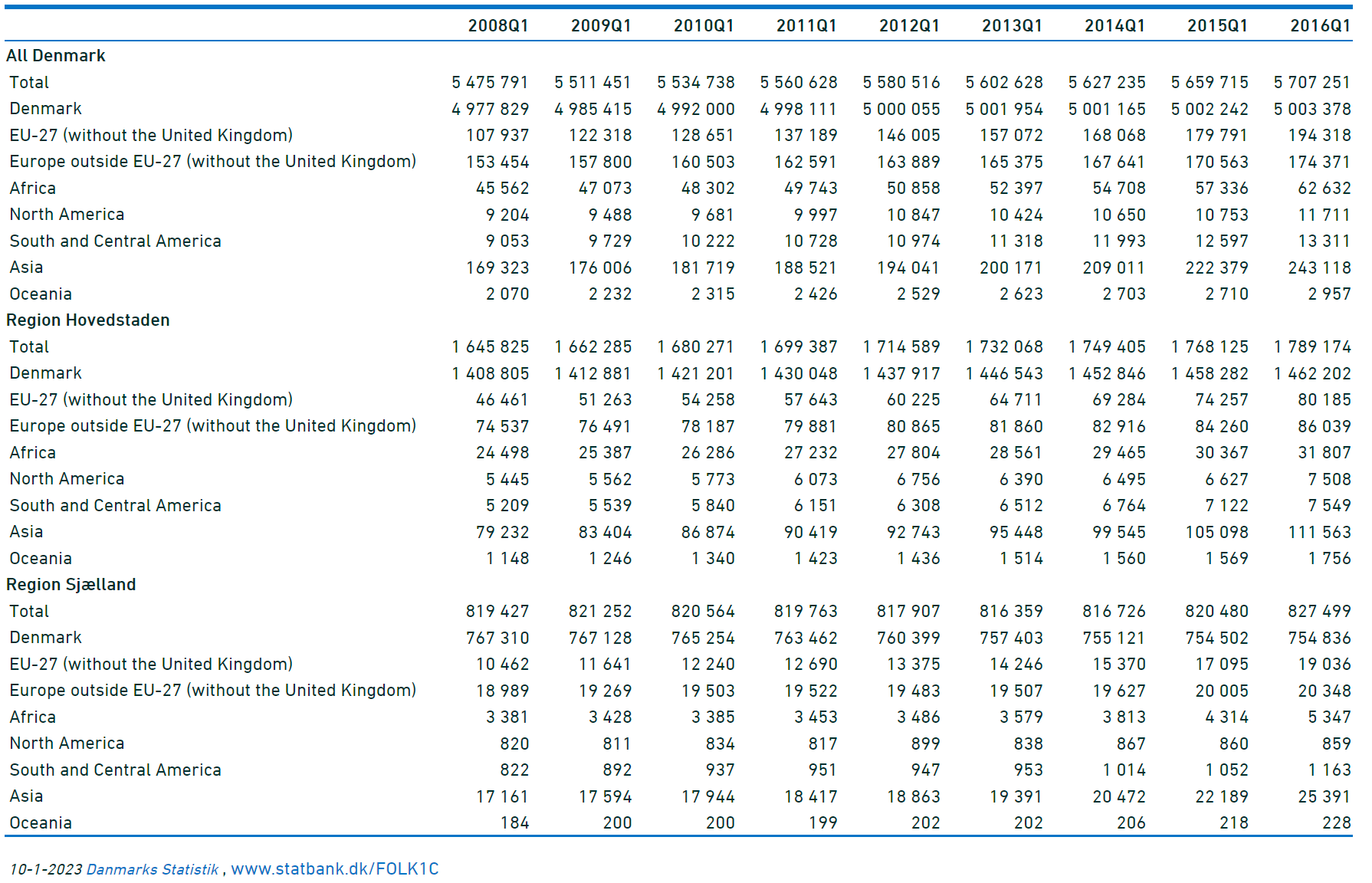

Supplement: Supplementary file 1. [file elife-83116-supp1.docx]

## Supplementary file 3: The study sample birth year distribution.


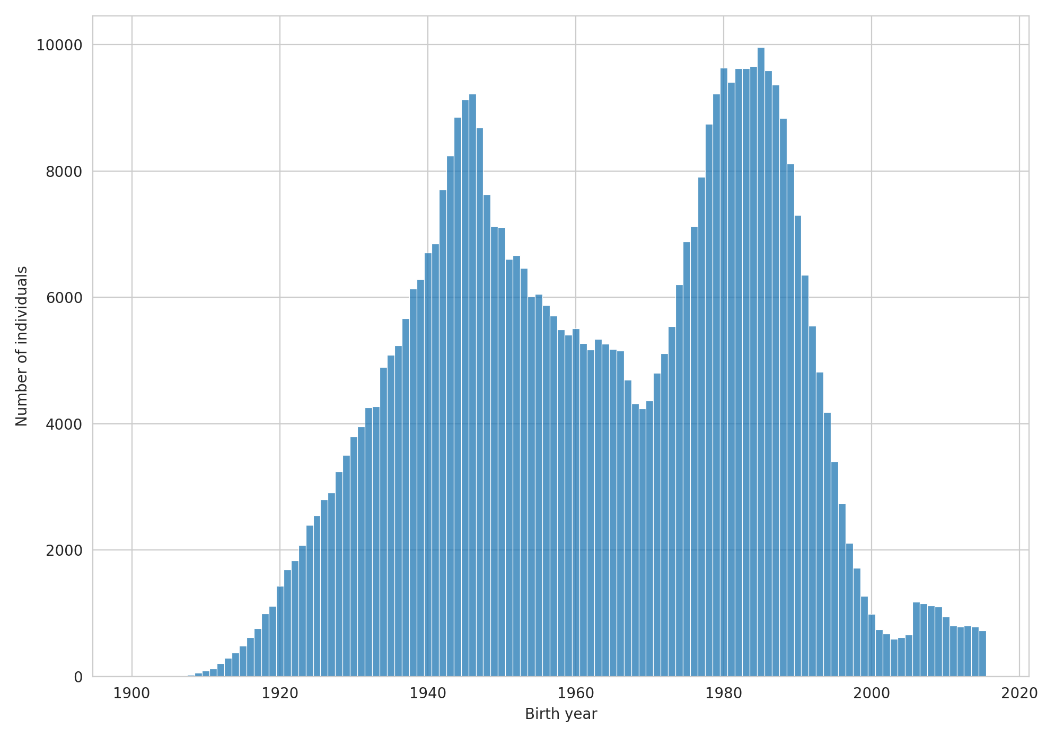

Supplement: Supplementary file 3. [file elife-83116-supp3.docx]
