# Supplementary material for: Associations of ABO and Rhesus D blood groups with phenome-wide disease incidence: A 41-year retrospective cohort study of 482,914 patients
Source: eLife. 2023 Mar 9;12:e83116. doi: 10.7554/eLife.83116 (PMC10042530; doi:10.7554/eLife.83116)
Supplement: Supplementary file 2. [file elife-83116-supp2.docx]

## Supplementary file 2: List of Phecodes defined as congenital or hereditary.

|  | **3-digit Phecodes** | **4-digit Phecodes** |
| --- | --- | --- |
| Phecodes | "356", "691", "637", "658", "661", "657", "656", "747", "748", "749", "750", "751", "752", "753", "754", "755", "756", "757", "758", "759", "665", "282" | "199.4", "244.5", "612.3", "286.1", "282.9", "362.7", "520.1", "282.8", "334.1", "364.5" |
