# Supplementary material for: Associations of ABO and Rhesus D blood groups with phenome-wide disease incidence: A 41-year retrospective cohort study of 482,914 patients
Source: eLife. 2023 Mar 9;12:e83116. doi: 10.7554/eLife.83116 (PMC10042530; doi:10.7554/eLife.83116)
Supplement: Supplementary file 5. — a. Statistically significant effect estimates are marked with bold (FDR adjusted P-value <0.05). b. The FDR adjusted p-values and 95% confidence intervals are presented. c. FDR adjusted p-values above 0.97 were set to 0.97 to enable estimation of adjusted confidence intervals. d. Estimates represent increases or decreases in years of age of first diagnosis. [file elife-83116-supp5.docx]

## Supplementary file 5: Associations between ABO/RhD blood groups and the age of the first diagnosis.

|  | | | **Blood group A** | | **Blood group B** | | **Blood group AB** | | **Blood group 0** | | **Blood group RhD** | |
| --- | --- | --- | --- | --- | --- | --- | --- | --- | --- | --- | --- | --- |
| **Phecode** | **Phenotype** | **N** | **Estimate (95%CI)** | **P-value** | **Estimate (95%CI)** | **P-value** | **Estimate (95%CI)** | **P-value** | **Estimate (95%CI)** | **P-value** | **Estimate (95%CI)** | **P-value** |
| 008 | Intestinal infection | 12268 | -0.03 (-1.66, 1.59) | 0.97 | 0.28 (-7.37, 7.94) | 0.948 | -0.75 (-8.68, 7.19) | 0.864 | 0.04 (-2.04, 2.12) | 0.97 | 0.19 (-7.56, 7.94) | 0.966 |
| 008.5 | Bacterial enteritis | 3292 | 0.08 (-3.72, 3.88) | 0.97 | 0.53 (-21.48, 22.55) | 0.966 | -0.9 (-32.62, 30.82) | 0.96 | -0.16 (-7.66, 7.35) | 0.97 | 0.47 (-19.02, 19.97) | 0.966 |
| 008.51 | Intestinal e.coli | 141 | 2.2 (-43, 47.4) | 0.93 | -2.14 (-92.14, 87.86) | 0.966 | -3.72 (-179.72, 172.28) | 0.97 | -1.25 (-55.95, 53.45) | 0.967 | -0.03 (-1.67, 1.6) | 0.97 |
| 008.52 | Intestinal infection due to C. difficile | 3777 | 0.06 (-2.95, 3.08) | 0.97 | -0.06 (-3.11, 2.98) | 0.97 | -0.4 (-6.08, 5.29) | 0.9 | 0.03 (-1.58, 1.65) | 0.97 | 0.24 (-2.86, 3.35) | 0.887 |
| 008.6 | Viral Enteritis | 4401 | -0.4 (-4.63, 3.83) | 0.864 | 0.39 (-10.12, 10.89) | 0.948 | -0.59 (-16.74, 15.56) | 0.948 | 0.34 (-5.79, 6.48) | 0.919 | -0.2 (-9.47, 9.08) | 0.97 |
| 008.7 | Intestinal infection due to protozoa | 447 | 0.21 (-9.75, 10.17) | 0.97 | 1.57 (-63.21, 66.35) | 0.966 | -3.35 (-72.51, 65.81) | 0.931 | -0.27 (-13.22, 12.67) | 0.97 | -1.7 (-47.97, 44.57) | 0.948 |
| 010 | Tuberculosis | 2101 | 0.31 (-14.16, 14.77) | 0.97 | 0.77 (-17.04, 18.58) | 0.938 | 0.09 (-4.11, 4.29) | 0.97 | -0.74 (-10.39, 8.91) | 0.89 | 1.28 (-12.37, 14.94) | 0.864 |
| 031 | Diseases due to other mycobacteria | 131 | 1 (-34.19, 36.18) | 0.96 | 2.63 (-25.35, 30.62) | 0.864 | 3.03 (-116.48, 122.53) | 0.964 | -2.27 (-20.59, 16.04) | 0.82 | 2.01 (-23.53, 27.54) | 0.887 |
| 038 | Septicemia | 26086 | -0.06 (-2.88, 2.75) | 0.967 | 0.04 (-2.04, 2.13) | 0.97 | 0.05 (-2.26, 2.36) | 0.97 | 0.04 (-1.8, 1.88) | 0.97 | 0.25 (-1.92, 2.42) | 0.833 |
| 038.1 | Gram negative septicemia | 3854 | -0.42 (-4.93, 4.08) | 0.864 | 0.26 (-12, 12.52) | 0.97 | 0.06 (-2.92, 3.05) | 0.97 | 0.31 (-6.23, 6.85) | 0.932 | 0.47 (-8.94, 9.87) | 0.929 |
| 038.2 | Gram positive septicemia | 2108 | 0.24 (-9.84, 10.33) | 0.966 | 0 (-0.16, 0.17) | 0.97 | -0.2 (-9.85, 9.44) | 0.97 | -0.22 (-9.31, 8.88) | 0.966 | 0.76 (-6.36, 7.87) | 0.845 |
| 041 | Bacterial infection NOS | 19993 | 0.01 (-0.68, 0.71) | 0.97 | 0.2 (-5.36, 5.77) | 0.948 | 0.08 (-3.59, 3.74) | 0.97 | -0.12 (-4.21, 3.98) | 0.96 | 0.29 (-2.76, 3.34) | 0.864 |
| 041.1 | Staphylococcus infections | 1543 | -0.54 (-6.28, 5.21) | 0.865 | 0.68 (-12.11, 13.47) | 0.923 | -0.11 (-5.47, 5.25) | 0.97 | 0.27 (-10.84, 11.38) | 0.966 | 0.44 (-15.34, 16.22) | 0.961 |
| 041.2 | Streptococcus infection | 6169 | 0.03 (-1.61, 1.68) | 0.97 | 0.12 (-5.75, 6) | 0.97 | 0 (-0.18, 0.19) | 0.97 | -0.09 (-4.37, 4.18) | 0.97 | 0.52 (-5, 6.04) | 0.864 |
| 041.4 | E. coli | 284 | 0.01 (-0.34, 0.36) | 0.97 | -1.07 (-17.63, 15.49) | 0.907 | -0.09 (-4.24, 4.06) | 0.97 | 0.47 (-16.45, 17.39) | 0.961 | 0.35 (-16.43, 17.14) | 0.97 |
| 053 | Herpes zoster | 2573 | 0.17 (-7.96, 8.3) | 0.97 | -0.14 (-6.57, 6.3) | 0.97 | 0.53 (-24.43, 25.49) | 0.97 | -0.2 (-9.73, 9.33) | 0.97 | 0.21 (-9.49, 9.9) | 0.97 |
| 053.1 | Herpes zoster with nervous system complications | 282 | 1.21 (-26.01, 28.43) | 0.936 | -1.55 (-60.82, 57.71) | 0.963 | 0.91 (-41.89, 43.7) | 0.97 | -0.82 (-34.54, 32.91) | 0.966 | 1.02 (-47.17, 49.2) | 0.97 |
| 054 | Herpes simplex | 3690 | 0.17 (-7.78, 8.12) | 0.97 | -1.13 (-13.11, 10.85) | 0.864 | 1.07 (-28.1, 30.25) | 0.948 | 0.12 (-5.72, 5.96) | 0.97 | 0.53 (-21.47, 22.54) | 0.966 |
| 070 | Viral hepatitis | 6603 | -0.41 (-5.63, 4.81) | 0.887 | 0.36 (-11.16, 11.89) | 0.955 | 0.39 (-18, 18.78) | 0.97 | 0.16 (-7.19, 7.5) | 0.97 | 0.06 (-2.69, 2.81) | 0.97 |
| 070.1 | Viral hepatitis A | 404 | 0.33 (-15.15, 15.8) | 0.97 | 0 (-0.12, 0.12) | 0.97 | 0.97 (-44.84, 46.78) | 0.97 | -0.55 (-26.63, 25.53) | 0.97 | -0.9 (-43.62, 41.82) | 0.97 |
| 070.2 | Viral hepatitis B | 1666 | -0.75 (-10.26, 8.76) | 0.887 | 1.46 (-5.76, 8.68) | 0.705 | 1.94 (-18.65, 22.52) | 0.864 | -0.59 (-12.8, 11.61) | 0.93 | 1.28 (-12.32, 14.88) | 0.864 |
| 070.3 | Viral hepatitis C | 1836 | -0.26 (-9.72, 9.2) | 0.961 | 0.01 (-0.66, 0.69) | 0.97 | 0.01 (-0.31, 0.33) | 0.97 | 0.25 (-10.15, 10.66) | 0.966 | -0.54 (-9.69, 8.61) | 0.915 |
| 070.4 | Chronic hepatitis | 936 | 0.07 (-3.43, 3.58) | 0.97 | -1.74 (-20.22, 16.75) | 0.864 | -0.66 (-32.01, 30.68) | 0.97 | 0.84 (-16.33, 18.01) | 0.93 | 1.76 (-15.46, 18.98) | 0.852 |
| 070.9 | Hepatitis NOS | 1715 | 0.27 (-12.66, 13.21) | 0.97 | -0.2 (-9.73, 9.32) | 0.97 | -1.82 (-25.02, 21.37) | 0.887 | 0.12 (-5.61, 5.85) | 0.97 | -0.74 (-21.01, 19.52) | 0.948 |
| 071 | Human immunodeficiency virus [HIV] disease | 1183 | 0.25 (-11.79, 12.3) | 0.97 | -0.04 (-1.78, 1.71) | 0.97 | 0.45 (-20.76, 21.65) | 0.97 | -0.32 (-13.44, 12.81) | 0.966 | -0.13 (-6.08, 5.83) | 0.97 |
| 071.1 | HIV infection, symptomatic | 1183 | 0.25 (-11.79, 12.3) | 0.97 | -0.04 (-1.78, 1.71) | 0.97 | 0.45 (-20.76, 21.65) | 0.97 | -0.32 (-13.44, 12.81) | 0.966 | -0.13 (-6.08, 5.83) | 0.97 |
| 078 | Viral warts & HPV | 8674 | -0.12 (-5.22, 4.99) | 0.967 | 0.1 (-4.63, 4.83) | 0.97 | 0.16 (-7.22, 7.54) | 0.97 | 0.05 (-2.25, 2.35) | 0.97 | 0.37 (-3.6, 4.35) | 0.864 |
| 079 | Viral infection | 25075 | -0.26 (-3.06, 2.54) | 0.864 | **0.92 (0.13, 1.71)** | **0.022** | 0.53 (-6.18, 7.23) | 0.887 | -0.23 (-3.22, 2.75) | 0.887 | 0.27 (-5.22, 5.75) | 0.93 |
| 079.1 | Varicella infection | 1005 | -1.11 (-12.93, 10.71) | 0.864 | 2.78 (-1.23, 6.8) | 0.175 | 0.96 (-44.54, 46.47) | 0.97 | -0.63 (-19.37, 18.12) | 0.952 | 1.8 (-13.85, 17.44) | 0.833 |
| 079.2 | Infectious mononucleosis | 3492 | -0.12 (-5.71, 5.47) | 0.97 | 0.45 (-11.8, 12.7) | 0.948 | -0.39 (-18.81, 18.03) | 0.97 | 0.02 (-0.98, 1.02) | 0.97 | 0.13 (-5.87, 6.12) | 0.97 |
| 080 | Postoperative infection | 11787 | 0.25 (-4.79, 5.28) | 0.93 | -0.06 (-2.93, 2.81) | 0.97 | -0.8 (-9.29, 7.69) | 0.864 | -0.08 (-3.81, 3.65) | 0.97 | 0.1 (-4.67, 4.87) | 0.97 |
| 081 | Infection/inflammation of internal prosthetic device; implant; and graft | 1474 | 0.37 (-7.09, 7.83) | 0.929 | -0.56 (-13.12, 12) | 0.936 | 0.78 (-18.37, 19.93) | 0.942 | -0.31 (-8.73, 8.11) | 0.948 | 0.32 (-12.78, 13.42) | 0.966 |
| 090 | Sexually transmitted infections (not HIV or hepatitis) | 2673 | 0.04 (-1.91, 2) | 0.97 | -0.03 (-1.49, 1.43) | 0.97 | 0.03 (-1.5, 1.57) | 0.97 | -0.04 (-1.72, 1.64) | 0.97 | -0.22 (-10.39, 9.95) | 0.97 |
| 090.2 | Gonococcal infections | 1223 | 0.05 (-2.51, 2.62) | 0.97 | -0.47 (-22.66, 21.72) | 0.97 | 0.61 (-28.21, 29.43) | 0.97 | 0.02 (-0.92, 0.96) | 0.97 | -0.13 (-6.22, 5.96) | 0.97 |
| 090.3 | Venereal diseases due to Chlamydia trachomatis | 1005 | -0.33 (-12.05, 11.38) | 0.96 | -0.02 (-0.95, 0.91) | 0.97 | -0.77 (-25.58, 24.04) | 0.956 | 0.5 (-7.55, 8.55) | 0.911 | -0.62 (-13.15, 11.9) | 0.929 |
| 110 | Dermatophytosis / Dermatomycosis | 3700 | 0.02 (-0.7, 0.73) | 0.97 | 0.36 (-9.39, 10.11) | 0.948 | -0.12 (-5.63, 5.4) | 0.97 | -0.17 (-7.59, 7.25) | 0.967 | 0.7 (-4.95, 6.35) | 0.82 |
| 110.1 | Dermatophytosis | 897 | 0.1 (-4.58, 4.78) | 0.97 | 0.37 (-17.33, 18.08) | 0.97 | 0.03 (-1.61, 1.68) | 0.97 | -0.28 (-13.48, 12.92) | 0.97 | 1.46 (-14.02, 16.93) | 0.864 |
| 110.11 | Dermatophytosis of nail | 210 | 0.08 (-3.9, 4.07) | 0.97 | 1.07 (-49.54, 51.68) | 0.97 | 0.87 (-40.49, 42.24) | 0.97 | -0.7 (-33.72, 32.32) | 0.97 | 1.66 (-36.15, 39.48) | 0.937 |
| 110.12 | Althete's foot | 245 | 0.94 (-37.24, 39.13) | 0.965 | -1.52 (-42.79, 39.76) | 0.948 | 0.55 (-25.35, 26.45) | 0.97 | -0.37 (-17.65, 16.91) | 0.97 | 2.29 (-22.04, 26.61) | 0.864 |
| 110.13 | Dermatophytosis of the body | 211 | 1.12 (-21.92, 24.17) | 0.93 | 0.33 (-15.15, 15.81) | 0.97 | -2.5 (-58.67, 53.66) | 0.936 | -0.94 (-26.51, 24.63) | 0.948 | -1.47 (-34.51, 31.57) | 0.936 |
| 110.2 | Dermatomycoses | 542 | 0.6 (-23.93, 25.12) | 0.966 | 0.62 (-28.84, 30.08) | 0.97 | 0.73 (-33.64, 35.09) | 0.97 | -1.04 (-17.31, 15.23) | 0.908 | 1.29 (-25.4, 27.99) | 0.931 |
| 112 | Candidiasis | 4773 | 0.24 (-9.67, 10.16) | 0.966 | 0.7 (-8.19, 9.58) | 0.887 | 0.4 (-18.73, 19.53) | 0.97 | -0.61 (-5.9, 4.69) | 0.833 | -0.18 (-8.91, 8.54) | 0.97 |
| 112.3 | Candidiasis of skin and nails | 109 | 1.27 (-50.89, 53.42) | 0.966 | -2.09 (-59.04, 54.86) | 0.948 | -3.25 (-91.58, 85.09) | 0.948 | 0.48 (-22.4, 23.36) | 0.97 | -1.58 (-67.89, 64.74) | 0.966 |
| 117 | Mycoses | 756 | -0.29 (-12.42, 11.85) | 0.966 | 0.72 (-14.31, 15.74) | 0.932 | -0.61 (-29.49, 28.27) | 0.97 | 0.09 (-4.07, 4.24) | 0.97 | -0.42 (-17.72, 16.88) | 0.966 |
| 117.4 | Aspergillosis | 302 | -0.8 (-17.2, 15.6) | 0.93 | 1.36 (-18.34, 21.06) | 0.901 | 0.06 (-2.96, 3.08) | 0.97 | 0.22 (-10.02, 10.46) | 0.97 | -0.66 (-28.02, 26.69) | 0.966 |
| 130 | Spirochetal infection | 1883 | -0.56 (-7.89, 6.77) | 0.89 | 0.8 (-13.49, 15.1) | 0.919 | -1.14 (-26.64, 24.37) | 0.936 | 0.39 (-10.21, 10.99) | 0.948 | -0.77 (-10.52, 8.99) | 0.887 |
| 130.1 | Lyme disease | 1424 | -0.27 (-13.11, 12.56) | 0.97 | 0.94 (-11.07, 12.96) | 0.887 | 0.07 (-3.19, 3.33) | 0.97 | -0.15 (-7.23, 6.93) | 0.97 | -0.85 (-11.44, 9.74) | 0.884 |
| 131 | Protozoan infection | 393 | 0.18 (-8.21, 8.56) | 0.97 | 1.42 (-60.71, 63.55) | 0.967 | -2.87 (-81.04, 75.3) | 0.948 | -0.17 (-8.09, 7.75) | 0.97 | -0.35 (-16.89, 16.19) | 0.97 |
| 132 | Infestation (lice, mites) | 361 | 1.55 (-30.86, 33.95) | 0.932 | -3.62 (-42.09, 34.86) | 0.864 | -1.56 (-75.56, 72.43) | 0.97 | 0.17 (-7.72, 8.05) | 0.97 | 1.46 (-58.59, 61.51) | 0.966 |
| 132.1 | Pediculosis and phthirus infestation | 120 | 0.69 (-32.02, 33.41) | 0.97 | 4.47 (-117.08, 126.01) | 0.948 | -7.58 (-88.24, 73.07) | 0.864 | -0.27 (-13.07, 12.52) | 0.97 | -1.02 (-49.06, 47.02) | 0.97 |
| 133 | Arthropod-borne diseases | 667 | -0.39 (-18.62, 17.85) | 0.97 | -1.01 (-42.67, 40.65) | 0.966 | 0.54 (-24.86, 25.93) | 0.97 | 0.79 (-31.74, 33.32) | 0.966 | -0.15 (-7, 6.71) | 0.97 |
| 134 | Helminthiases | 658 | 1.7 (-16.41, 19.82) | 0.864 | -2.5 (-29.07, 24.07) | 0.864 | -1.15 (-55.36, 53.07) | 0.97 | -0.25 (-12.15, 11.64) | 0.97 | -1.89 (-40.44, 36.66) | 0.93 |
| 134.1 | Intestinal helminthiases | 301 | 3.28 (-25.27, 31.84) | 0.833 | -1.13 (-54.55, 52.29) | 0.97 | -0.89 (-42.98, 41.2) | 0.97 | -2.39 (-32.71, 27.94) | 0.887 | -2.91 (-82.19, 76.37) | 0.948 |
| 136 | Other infectious and parasitic diseases | 475 | -0.63 (-24.69, 23.43) | 0.963 | 0.05 (-2.2, 2.3) | 0.97 | 0.56 (-25.94, 27.06) | 0.97 | 0.52 (-21.1, 22.14) | 0.966 | 1.25 (-19.76, 22.26) | 0.915 |
| 145 | Cancer of mouth | 1890 | 0.34 (-7.24, 7.91) | 0.936 | -0.98 (-8.86, 6.91) | 0.82 | 0.21 (-9.61, 10.02) | 0.97 | 0.01 (-0.48, 0.5) | 0.97 | 0.7 (-6.78, 8.19) | 0.864 |
| 145.1 | Cancer of lip | 153 | 1.38 (-36.12, 38.87) | 0.948 | -1.51 (-72.99, 69.97) | 0.97 | -5.24 (-91.83, 81.36) | 0.913 | -0.19 (-9.41, 9.02) | 0.97 | 5.71 (-6.95, 18.37) | 0.383 |
| 145.2 | Cancer of tongue | 606 | 0.14 (-6.27, 6.54) | 0.97 | 0.08 (-3.69, 3.85) | 0.97 | 1.12 (-22.38, 24.63) | 0.932 | -0.41 (-14.8, 13.98) | 0.96 | 0.76 (-9.13, 10.65) | 0.89 |
| 145.3 | Cancer of major salivary glands | 120 | -1.29 (-36.52, 33.94) | 0.948 | 0.75 (-34.88, 36.39) | 0.97 | -0.73 (-35.05, 33.6) | 0.97 | 1.26 (-32.97, 35.49) | 0.948 | -0.04 (-1.92, 1.84) | 0.97 |
| 145.4 | Cancer of the gums | 176 | 0.68 (-31.3, 32.66) | 0.97 | -2.56 (-53.96, 48.85) | 0.929 | -2.31 (-97.52, 92.9) | 0.966 | 0.37 (-17.26, 18) | 0.97 | -1.04 (-49.59, 47.51) | 0.97 |
| 145.5 | Cancer of the mouth floor | 366 | 0.44 (-15.44, 16.32) | 0.961 | -1.92 (-9.54, 5.7) | 0.634 | 0.29 (-13.24, 13.81) | 0.97 | 0.26 (-11.87, 12.39) | 0.97 | 1.25 (-9.66, 12.17) | 0.833 |
| 149 | Cancer of larynx, pharynx, nasal cavities | 3451 | -0.29 (-5.16, 4.58) | 0.915 | 0.08 (-3.67, 3.83) | 0.97 | 0.12 (-5.61, 5.85) | 0.97 | 0.24 (-5.19, 5.67) | 0.936 | -0.13 (-6.33, 6.06) | 0.97 |
| 149.1 | Cancer of oropharynx | 1518 | -0.81 (-1.87, 0.24) | 0.132 | 0.01 (-0.56, 0.58) | 0.97 | 0.87 (-10.18, 11.92) | 0.887 | 0.66 (-1.38, 2.71) | 0.537 | 0.41 (-7.89, 8.72) | 0.929 |
| 149.2 | Cancer of nasopharynx | 203 | -2.02 (-18.3, 14.26) | 0.82 | 0.5 (-23.16, 24.16) | 0.97 | -0.82 (-39.55, 37.91) | 0.97 | 1.91 (-13.49, 17.32) | 0.82 | 0.75 (-34.93, 36.44) | 0.97 |
| 149.3 | Cancer of hypopharynx | 608 | 0.25 (-11.73, 12.24) | 0.97 | 0.49 (-19.84, 20.83) | 0.966 | -0.28 (-13.49, 12.93) | 0.97 | -0.4 (-11.33, 10.53) | 0.948 | -0.36 (-16.01, 15.29) | 0.967 |
| 149.4 | Cancer of larynx | 1164 | 0.2 (-9.32, 9.73) | 0.97 | 0.01 (-0.33, 0.35) | 0.97 | -0.33 (-16.17, 15.5) | 0.97 | -0.14 (-6.54, 6.27) | 0.97 | -0.32 (-15.5, 14.86) | 0.97 |
| 149.9 | Cancer of of nasal cavities | 381 | 0.82 (-16.02, 17.65) | 0.931 | -1.65 (-29.3, 26.01) | 0.915 | 0.56 (-25.71, 26.82) | 0.97 | -0.4 (-19.14, 18.34) | 0.97 | -0.24 (-11.5, 11.02) | 0.97 |
| 150 | Cancer of esophagus | 2442 | 0 (-0.1, 0.1) | 0.97 | 0.14 (-6.61, 6.89) | 0.97 | -0.5 (-10.56, 9.56) | 0.929 | 0.03 (-1.43, 1.49) | 0.97 | -0.16 (-6.75, 6.43) | 0.966 |
| 151 | Cancer of stomach | 2423 | 0.37 (-3.59, 4.34) | 0.864 | -0.3 (-12.71, 12.11) | 0.966 | 0.26 (-11.9, 12.42) | 0.97 | -0.31 (-6.01, 5.4) | 0.923 | 0.02 (-0.81, 0.85) | 0.97 |
| 153 | Colorectal cancer | 9507 | 0.05 (-2.47, 2.57) | 0.97 | -0.22 (-6.31, 5.87) | 0.948 | -0.27 (-11.35, 10.81) | 0.966 | 0.08 (-3.63, 3.78) | 0.97 | 0.33 (-3.17, 3.83) | 0.864 |
| 153.2 | Colon cancer | 8870 | 0.02 (-1.13, 1.18) | 0.97 | -0.22 (-6.34, 5.89) | 0.948 | -0.25 (-11.31, 10.8) | 0.967 | 0.11 (-4.32, 4.53) | 0.966 | 0.37 (-2.85, 3.59) | 0.833 |
| 153.3 | Malignant neoplasm of rectum, rectosigmoid junction, and anus | 658 | 0.2 (-9.3, 9.7) | 0.97 | -0.24 (-11.51, 11.03) | 0.97 | -0.61 (-29.28, 28.07) | 0.97 | -0.02 (-0.89, 0.85) | 0.97 | 0.21 (-9.89, 10.32) | 0.97 |
| 155 | Cancer of liver and intrahepatic bile duct | 1368 | 0 (-0.05, 0.05) | 0.97 | -0.58 (-6.74, 5.58) | 0.864 | -0.4 (-16.87, 16.07) | 0.966 | 0.3 (-5.35, 5.96) | 0.923 | 0.41 (-7.88, 8.7) | 0.929 |
| 155.1 | Malignant neoplasm of liver, primary | 853 | -0.04 (-1.94, 1.86) | 0.97 | -0.37 (-14.57, 13.83) | 0.963 | -0.82 (-12.57, 10.93) | 0.9 | 0.34 (-5.98, 6.65) | 0.923 | 0.11 (-5.03, 5.25) | 0.97 |
| 157 | Pancreatic cancer | 2828 | 0.07 (-3.29, 3.43) | 0.97 | -0.02 (-1.06, 1.02) | 0.97 | -0.35 (-9.77, 9.07) | 0.948 | 0 (-0.08, 0.08) | 0.97 | 0.14 (-6.19, 6.46) | 0.97 |
| 158 | Neoplasm of unspecified nature of digestive system | 811 | 0.71 (-28.52, 29.94) | 0.966 | 1.48 (-51.8, 54.76) | 0.961 | 0.09 (-4.08, 4.25) | 0.97 | -1.25 (-23.51, 21.01) | 0.919 | -0.34 (-16.34, 15.67) | 0.97 |
| 159 | Malignant neoplasm of other and ill-defined sites within the digestive organs and peritoneum | 4459 | 0.12 (-5, 5.25) | 0.966 | 0.13 (-5.92, 6.18) | 0.97 | -0.2 (-9.84, 9.43) | 0.97 | -0.14 (-4.41, 4.14) | 0.954 | -0.14 (-5.95, 5.67) | 0.966 |
| 159.2 | Malignant neoplasm of small intestine, including duodenum | 535 | 0.48 (-12.63, 13.59) | 0.948 | 0.21 (-9.69, 10.1) | 0.97 | 0.3 (-14.05, 14.65) | 0.97 | -0.6 (-11.77, 10.58) | 0.923 | -0.24 (-11.37, 10.9) | 0.97 |
| 159.3 | Malignant neoplasm of gallbladder and extrahepatic bile ducts | 806 | 0.12 (-5.68, 5.92) | 0.97 | -0.5 (-15.43, 14.43) | 0.952 | -0.07 (-3.55, 3.4) | 0.97 | 0.08 (-3.56, 3.71) | 0.97 | -0.01 (-0.59, 0.57) | 0.97 |
| 159.4 | Malignant neoplasm of retroperitoneum and peritoneum | 439 | -0.28 (-13.76, 13.19) | 0.97 | 1.57 (-26.37, 29.51) | 0.919 | -2.13 (-38.36, 34.11) | 0.916 | 0.13 (-6.1, 6.36) | 0.97 | -1.56 (-19.48, 16.36) | 0.874 |
| 164 | Cancer of intrathoracic organs | 195 | -0.91 (-25.75, 23.92) | 0.948 | -1.5 (-42.41, 39.4) | 0.948 | 3.01 (-64.21, 70.24) | 0.936 | 1.21 (-21.48, 23.91) | 0.923 | -2.01 (-23.33, 19.32) | 0.864 |
| 165 | Cancer within the respiratory system | 11019 | 0.03 (-1.17, 1.22) | 0.97 | -0.12 (-5.01, 4.78) | 0.966 | -0.12 (-6.03, 5.78) | 0.97 | 0.04 (-1.91, 2) | 0.97 | -0.14 (-3.34, 3.05) | 0.936 |
| 165.1 | Cancer of bronchus; lung | 10403 | 0 (-0.23, 0.22) | 0.97 | -0.12 (-5.04, 4.8) | 0.966 | -0.13 (-6.51, 6.24) | 0.97 | 0.07 (-2.96, 3.11) | 0.966 | -0.16 (-3.44, 3.12) | 0.929 |
| 170 | Cancer of bone and connective tissue | 1751 | -0.69 (-8.04, 6.66) | 0.864 | 0.52 (-20.74, 21.77) | 0.966 | -0.92 (-26.01, 24.17) | 0.948 | 0.68 (-6.51, 7.86) | 0.864 | 0.2 (-9.4, 9.8) | 0.97 |
| 170.1 | Bone cancer | 564 | -0.71 (-23.46, 22.05) | 0.956 | 0.38 (-17.43, 18.19) | 0.97 | -0.77 (-37.13, 35.59) | 0.97 | 0.77 (-20.1, 21.63) | 0.948 | 0.58 (-26.73, 27.89) | 0.97 |
| 170.2 | Cancer of connective tissue | 1369 | -0.56 (-11.74, 10.62) | 0.929 | 0.4 (-18.5, 19.3) | 0.97 | -0.49 (-23.8, 22.82) | 0.97 | 0.5 (-9.93, 10.92) | 0.932 | 0 (-0.13, 0.12) | 0.97 |
| 172 | Skin cancer | 17775 | -0.09 (-3.77, 3.59) | 0.966 | 0.03 (-1.53, 1.59) | 0.97 | -0.23 (-9.69, 9.23) | 0.966 | 0.12 (-3.07, 3.31) | 0.948 | -0.32 (-2.93, 2.28) | 0.82 |
| 172.11 | Melanomas of skin | 6833 | -0.01 (-0.34, 0.33) | 0.97 | 0.23 (-9.62, 10.07) | 0.967 | -0.16 (-7.87, 7.54) | 0.97 | -0.05 (-2.43, 2.33) | 0.97 | -0.49 (-5.68, 4.71) | 0.864 |
| 172.2 | Other non-epithelial cancer of skin | 11667 | -0.08 (-3.91, 3.75) | 0.97 | -0.14 (-6.21, 5.93) | 0.968 | -0.19 (-9.35, 8.96) | 0.97 | 0.17 (-3.23, 3.57) | 0.929 | -0.21 (-4.59, 4.16) | 0.93 |
| 172.3 | Carcinoma in situ of skin | 442 | -1.45 (-8.61, 5.71) | 0.705 | 0.8 (-32.29, 33.89) | 0.966 | 0.64 (-29.81, 31.1) | 0.97 | 1.01 (-9.7, 11.71) | 0.864 | -0.61 (-26.11, 24.89) | 0.966 |
| 173 | Neoplasm of uncertain behavior of skin | 332 | 0.58 (-26.83, 27.98) | 0.97 | -0.35 (-16.81, 16.12) | 0.97 | -0.48 (-23.14, 22.18) | 0.97 | -0.37 (-17.75, 17.02) | 0.97 | 0.94 (-43.28, 45.15) | 0.97 |
| 174 | Breast cancer | 13763 | -0.16 (-4.39, 4.08) | 0.948 | -0.08 (-3.88, 3.71) | 0.97 | 0.55 (-6.65, 7.75) | 0.89 | 0.09 (-4.34, 4.53) | 0.97 | -0.04 (-1.82, 1.74) | 0.97 |
| 174.1 | Breast cancer [female] | 1217 | 0.53 (-5.1, 6.16) | 0.864 | -0.46 (-19.35, 18.43) | 0.966 | -0.8 (-17.65, 16.04) | 0.932 | -0.19 (-9.15, 8.77) | 0.97 | -0.09 (-4.32, 4.14) | 0.97 |
| 174.11 | Malignant neoplasm of female breast | 12875 | -0.19 (-4.16, 3.78) | 0.931 | -0.09 (-4.35, 4.17) | 0.97 | 0.52 (-9.18, 10.21) | 0.923 | 0.14 (-5.64, 5.92) | 0.966 | -0.08 (-3.8, 3.64) | 0.97 |
| 174.3 | Neoplasm of uncertain behavior of breast | 582 | 0.16 (-7.42, 7.74) | 0.97 | -0.99 (-41.73, 39.76) | 0.966 | 0.99 (-45.79, 47.77) | 0.97 | 0.04 (-1.7, 1.78) | 0.97 | 0.98 (-36.56, 38.53) | 0.963 |
| 175 | Acquired absence of breast | 2260 | 0.1 (-4.8, 5.01) | 0.97 | -0.21 (-10.16, 9.74) | 0.97 | 0.19 (-8.67, 9.05) | 0.97 | -0.06 (-2.88, 2.76) | 0.97 | 0.33 (-8.56, 9.21) | 0.948 |
| 180 | Cervical cancer and dysplasia | 12538 | -0.14 (-5.79, 5.51) | 0.966 | 0.11 (-4.96, 5.17) | 0.97 | -0.22 (-10.76, 10.31) | 0.97 | 0.14 (-5.44, 5.71) | 0.966 | -0.22 (-8.18, 7.74) | 0.961 |
| 180.1 | Cervical cancer | 2003 | 0.08 (-3.93, 4.1) | 0.97 | -0.15 (-7.41, 7.1) | 0.97 | -0.06 (-2.66, 2.55) | 0.97 | -0.01 (-0.63, 0.6) | 0.97 | -0.65 (-18.32, 17.02) | 0.948 |
| 180.3 | Cervical intraepithelial neoplasia [CIN] [Cervical dysplasia] | 10895 | -0.24 (-3.41, 2.92) | 0.89 | 0.13 (-5.92, 6.18) | 0.97 | -0.26 (-12.63, 12.11) | 0.97 | 0.24 (-3.21, 3.69) | 0.9 | -0.02 (-1.17, 1.12) | 0.97 |
| 182 | Malignant neoplasm of uterus | 3447 | -0.69 (-6.22, 4.85) | 0.82 | 0 (-0.13, 0.12) | 0.97 | 0.45 (-20.86, 21.76) | 0.97 | 0.63 (-5.12, 6.39) | 0.84 | 0.04 (-1.64, 1.71) | 0.97 |
| 184 | Cancer of other female genital organs | 4018 | 0.23 (-10.83, 11.3) | 0.97 | -0.48 (-22.87, 21.92) | 0.97 | 1.22 (-32.11, 34.56) | 0.948 | -0.24 (-11.54, 11.06) | 0.97 | -0.22 (-10.47, 10.04) | 0.97 |
| 184.1 | Malignant neoplasm of ovary and other uterine adnexa | 261 | -0.04 (-1.79, 1.72) | 0.97 | -0.9 (-43.27, 41.47) | 0.97 | -1.74 (-73.5, 70.01) | 0.966 | 0.82 (-32.92, 34.56) | 0.966 | -0.32 (-15.33, 14.69) | 0.97 |
| 184.2 | Cancer of other female genital organs (excluding uterus and ovary) | 3144 | -0.42 (-17.67, 16.84) | 0.966 | -0.25 (-11.87, 11.38) | 0.97 | 1.61 (-28.41, 31.63) | 0.923 | 0.25 (-11.48, 11.98) | 0.97 | -0.19 (-9.25, 8.87) | 0.97 |
| 187 | Cancer of other male genital organs | 2197 | -1.36 (-4.52, 1.81) | 0.408 | 0.48 (-22.43, 23.4) | 0.97 | 1.31 (-28.27, 30.88) | 0.937 | 0.99 (-6.97, 8.95) | 0.82 | -0.8 (-16.86, 15.27) | 0.929 |
| 187.1 | Malignant neoplasm of unspecified male genital organ | 320 | -1.47 (-20.2, 17.25) | 0.887 | 0.17 (-7.99, 8.34) | 0.97 | -1.81 (-77.8, 74.18) | 0.966 | 1.67 (-16.12, 19.47) | 0.864 | -0.14 (-6.71, 6.44) | 0.97 |
| 187.2 | Malignant neoplasm of testis | 1673 | -0.64 (-12.63, 11.34) | 0.923 | 0.23 (-10.84, 11.31) | 0.97 | 1.38 (-27.51, 30.27) | 0.932 | 0.33 (-14.2, 14.86) | 0.967 | -1.11 (-12.89, 10.68) | 0.864 |
| 187.8 | Neoplasm of uncertain behavior of male genital organs | 189 | -2.69 (-31.34, 25.95) | 0.864 | 1.48 (-68.51, 71.47) | 0.97 | 2.98 (-119.71, 125.66) | 0.966 | 1.81 (-38.9, 42.53) | 0.936 | 1.45 (-61.69, 64.59) | 0.967 |
| 189 | Cancer of urinary organs (incl. kidney and bladder) | 4208 | -0.2 (-8.45, 8.05) | 0.966 | 0.04 (-1.64, 1.71) | 0.97 | -0.01 (-0.57, 0.55) | 0.97 | 0.19 (-7.61, 7.98) | 0.966 | 0.1 (-4.65, 4.85) | 0.97 |
| 189.2 | Cancer of bladder | 953 | -0.09 (-4.35, 4.17) | 0.97 | 0.23 (-10.77, 11.23) | 0.97 | 0.57 (-26.16, 27.29) | 0.97 | -0.11 (-5.39, 5.17) | 0.97 | 0.08 (-3.88, 4.05) | 0.97 |
| 189.21 | Malignant neoplasm of bladder | 3371 | -0.32 (-6.86, 6.22) | 0.931 | -0.07 (-3.49, 3.35) | 0.97 | -0.03 (-1.4, 1.35) | 0.97 | 0.35 (-6.21, 6.91) | 0.923 | 0.03 (-1.44, 1.51) | 0.97 |
| 190 | Cancer of eye | 362 | 1.39 (-24.66, 27.45) | 0.923 | -0.34 (-16.42, 15.74) | 0.97 | -1.55 (-74.88, 71.78) | 0.97 | -1.07 (-30.06, 27.93) | 0.948 | -0.23 (-11.26, 10.79) | 0.97 |
| 191 | Manlignant and unknown neoplasms of brain and nervous system | 3389 | -0.33 (-7.86, 7.2) | 0.937 | 0.15 (-7.11, 7.42) | 0.97 | -1.02 (-14, 11.96) | 0.887 | 0.47 (-4.56, 5.5) | 0.865 | 0.77 (-5.89, 7.42) | 0.833 |
| 191.1 | Cancer of brain and nervous system | 169 | -0.82 (-36.47, 34.83) | 0.967 | -0.68 (-32.94, 31.58) | 0.97 | 1 (-46.36, 48.36) | 0.97 | 0.78 (-33.11, 34.66) | 0.967 | 2.38 (-22.95, 27.71) | 0.864 |
| 191.11 | Cancer of brain | 2498 | -0.2 (-8.27, 7.87) | 0.966 | -0.1 (-4.89, 4.68) | 0.97 | 0.12 (-5.61, 5.85) | 0.97 | 0.21 (-8.52, 8.94) | 0.966 | 0.58 (-5.63, 6.79) | 0.864 |
| 194 | Cancer of other endocrine glands | 265 | -0.08 (-4.06, 3.89) | 0.97 | -1.33 (-56.2, 53.53) | 0.966 | -0.68 (-33.02, 31.65) | 0.97 | 0.76 (-30.37, 31.88) | 0.966 | 0.27 (-12.42, 12.96) | 0.97 |
| 195 | Cancer, suspected or other | 92731 | -0.04 (-0.89, 0.81) | 0.932 | 0.07 (-1.31, 1.45) | 0.93 | 0.12 (-1.42, 1.67) | 0.887 | -0.01 (-0.38, 0.37) | 0.97 | -0.01 (-0.56, 0.54) | 0.97 |
| 195.1 | Malignant neoplasm, other | 7383 | -0.01 (-0.26, 0.25) | 0.97 | -0.2 (-4.28, 3.88) | 0.931 | -0.1 (-4.64, 4.45) | 0.97 | 0.1 (-2.57, 2.77) | 0.948 | 0.06 (-2.81, 2.93) | 0.97 |
| 198 | Secondary malignant neoplasm | 15007 | -0.07 (-2.83, 2.69) | 0.966 | 0.14 (-3.65, 3.93) | 0.948 | 0.16 (-6.34, 6.65) | 0.966 | -0.01 (-0.72, 0.69) | 0.97 | 0.01 (-0.39, 0.41) | 0.97 |
| 198.1 | Secondary malignancy of lymph nodes | 3442 | -0.19 (-7.87, 7.49) | 0.966 | 0.25 (-11.49, 11.99) | 0.97 | 0.87 (-10.24, 11.99) | 0.887 | -0.06 (-2.88, 2.76) | 0.97 | -0.07 (-3.52, 3.37) | 0.97 |
| 198.2 | Secondary malignancy of respiratory organs | 3618 | -0.17 (-3.74, 3.39) | 0.93 | 0.05 (-2.12, 2.21) | 0.97 | -0.1 (-4.67, 4.47) | 0.97 | 0.17 (-3.39, 3.73) | 0.932 | 0.19 (-4.96, 5.34) | 0.948 |
| 198.3 | Secondary malignant neoplasm of digestive systems | 2432 | 0.04 (-1.98, 2.07) | 0.97 | -0.01 (-0.72, 0.69) | 0.97 | 0.48 (-9.61, 10.56) | 0.932 | -0.12 (-5.15, 4.92) | 0.967 | 0.03 (-1.27, 1.32) | 0.97 |
| 198.4 | Secondary malignant neoplasm of liver | 4039 | -0.12 (-3.48, 3.23) | 0.948 | 0.08 (-3.79, 3.96) | 0.97 | -0.01 (-0.57, 0.55) | 0.97 | 0.09 (-3.78, 3.97) | 0.966 | 0.08 (-3.55, 3.71) | 0.97 |
| 198.5 | Secondary malignancy of brain/spine | 2495 | -0.05 (-2.29, 2.19) | 0.97 | -0.36 (-6.31, 5.59) | 0.913 | 0.4 (-10.48, 11.28) | 0.948 | 0.12 (-4.96, 5.2) | 0.966 | 0.14 (-6.56, 6.84) | 0.97 |
| 198.6 | Secondary malignancy of bone | 2389 | 0.02 (-1.15, 1.2) | 0.97 | -0.07 (-3.34, 3.2) | 0.97 | -0.63 (-7.31, 6.05) | 0.864 | 0.11 (-4.86, 5.09) | 0.967 | 0 (-0.23, 0.24) | 0.97 |
| 198.7 | Secondary malignant neoplasm of skin | 703 | 0.18 (-8.37, 8.74) | 0.97 | 0.25 (-11.63, 12.13) | 0.97 | 0.82 (-32.87, 34.5) | 0.966 | -0.42 (-11.88, 11.04) | 0.948 | -0.21 (-9.97, 9.55) | 0.97 |
| 199 | Neoplasm of uncertain behavior | 2744 | -0.28 (-13.3, 12.75) | 0.97 | 0.21 (-9.64, 10.06) | 0.97 | 0.73 (-31.41, 32.87) | 0.968 | 0.06 (-2.93, 3.05) | 0.97 | 0.77 (-15, 16.54) | 0.93 |
| 200 | Myeloproliferative disease | 2051 | 0.09 (-4.39, 4.57) | 0.97 | 0.59 (-6.89, 8.07) | 0.887 | -0.8 (-11.03, 9.42) | 0.887 | -0.14 (-6.91, 6.63) | 0.97 | -0.16 (-7.72, 7.4) | 0.97 |
| 201 | Hodgkin's disease | 889 | -0.92 (-19.91, 18.06) | 0.93 | -0.23 (-11.17, 10.71) | 0.97 | 2.73 (-29.82, 35.28) | 0.879 | 0.53 (-22.68, 23.74) | 0.967 | 1.23 (-24.06, 26.53) | 0.93 |
| 202 | Cancer of other lymphoid, histiocytic tissue | 4011 | -0.19 (-7.88, 7.51) | 0.966 | 0.15 (-6.89, 7.19) | 0.97 | 1 (-9.68, 11.68) | 0.864 | -0.04 (-2.04, 1.96) | 0.97 | -0.02 (-1.05, 1) | 0.97 |
| 202.2 | Non-Hodgkins lymphoma | 3358 | -0.26 (-7.43, 6.9) | 0.948 | 0.16 (-7.29, 7.61) | 0.97 | 1.12 (-10.73, 12.97) | 0.863 | 0.01 (-0.41, 0.43) | 0.97 | -0.04 (-2.01, 1.93) | 0.97 |
| 202.21 | Nodular lymphoma | 396 | 0.24 (-11.2, 11.69) | 0.97 | -0.42 (-20.06, 19.22) | 0.97 | -0.6 (-28.93, 27.74) | 0.97 | 0.02 (-1.1, 1.15) | 0.97 | 0.93 (-24.34, 26.19) | 0.948 |
| 202.24 | Large cell lymphoma | 1587 | -0.24 (-6.67, 6.2) | 0.948 | -0.02 (-1.07, 1.03) | 0.97 | -0.07 (-3.61, 3.46) | 0.97 | 0.26 (-5.65, 6.17) | 0.937 | -0.09 (-4.57, 4.38) | 0.97 |
| 204 | Leukemia | 4423 | 0.19 (-5.04, 5.42) | 0.948 | -0.06 (-2.79, 2.68) | 0.97 | -0.28 (-13.43, 12.87) | 0.97 | -0.12 (-5.92, 5.68) | 0.97 | 0.3 (-6.41, 7) | 0.936 |
| 204.1 | Lymphoid leukemia | 293 | 1.19 (-41.77, 44.15) | 0.961 | -0.35 (-16.93, 16.23) | 0.97 | 3.42 (-89.66, 96.5) | 0.948 | -1.53 (-33.51, 30.45) | 0.932 | 0.04 (-1.92, 2) | 0.97 |
| 204.11 | Lymphoid leukemia, acute | 398 | -0.2 (-9.76, 9.36) | 0.97 | 0.01 (-0.36, 0.37) | 0.97 | -0.59 (-28.72, 27.53) | 0.97 | 0.34 (-15.59, 16.26) | 0.97 | 0.48 (-22.24, 23.21) | 0.97 |
| 204.12 | Lymphoid leukemia, chronic | 1476 | 0.32 (-6.48, 7.13) | 0.932 | -0.14 (-6.52, 6.25) | 0.97 | 0.16 (-7.48, 7.8) | 0.97 | -0.31 (-7.67, 7.06) | 0.94 | -0.11 (-5.5, 5.28) | 0.97 |
| 204.2 | Myeloid leukemia | 366 | -0.48 (-21.6, 20.64) | 0.967 | 1.51 (-26.79, 29.82) | 0.923 | 1.07 (-45.82, 47.97) | 0.967 | -0.33 (-16.04, 15.38) | 0.97 | 0.49 (-22.68, 23.66) | 0.97 |
| 204.21 | Myeloid leukemia, acute | 752 | -0.12 (-5.92, 5.67) | 0.97 | 0.59 (-15.58, 16.76) | 0.948 | 0.8 (-32.09, 33.69) | 0.966 | -0.21 (-9.91, 9.5) | 0.97 | 0.25 (-11.7, 12.21) | 0.97 |
| 204.22 | Myeloid leukemia, chronic | 249 | 0 (-0.14, 0.13) | 0.97 | 0.04 (-1.8, 1.88) | 0.97 | 0.76 (-35.2, 36.72) | 0.97 | -0.17 (-8.29, 7.95) | 0.97 | 0.41 (-18.82, 19.63) | 0.97 |
| 204.3 | Monocytic leukemia | 179 | -0.79 (-22.3, 20.72) | 0.948 | 1.77 (-28.31, 31.85) | 0.915 | 0.55 (-25.59, 26.7) | 0.97 | 0.1 (-4.83, 5.04) | 0.97 | 0.68 (-29.12, 30.47) | 0.968 |
| 204.4 | Multiple myeloma | 1212 | 0 (-0.09, 0.09) | 0.97 | -0.38 (-16.16, 15.4) | 0.966 | -1.18 (-13.75, 11.39) | 0.864 | 0.38 (-7.49, 8.26) | 0.93 | 0.54 (-10.33, 11.41) | 0.929 |
| 208 | Benign neoplasm of colon | 18530 | -0.08 (-3.65, 3.5) | 0.97 | -0.11 (-5.27, 5.05) | 0.97 | 0.14 (-6.68, 6.97) | 0.97 | 0.1 (-3.82, 4.01) | 0.966 | -0.14 (-5.82, 5.54) | 0.966 |
| 210 | Benign neoplasm of lip, oral cavity, and pharynx | 4905 | 0.04 (-2.07, 2.16) | 0.97 | 0.59 (-11.29, 12.48) | 0.929 | 0.05 (-2.44, 2.54) | 0.97 | -0.3 (-8.36, 7.77) | 0.948 | 0.35 (-14.24, 14.95) | 0.966 |
| 211 | Benign neoplasm of other parts of digestive system | 2253 | -0.19 (-9.1, 8.73) | 0.97 | 0.32 (-14.81, 15.45) | 0.97 | 1.7 (-13.12, 16.52) | 0.833 | -0.23 (-11.15, 10.69) | 0.97 | 0.36 (-14.36, 15.07) | 0.966 |
| 212 | Benign neoplasm of respiratory and intrathoracic organs | 3878 | -0.07 (-3.15, 3.02) | 0.97 | -0.31 (-14.8, 14.18) | 0.97 | 0.3 (-13.69, 14.28) | 0.97 | 0.14 (-6.31, 6.58) | 0.97 | 0.14 (-6.51, 6.79) | 0.97 |
| 213 | Benign neoplasm of bone and articular cartilage | 3179 | 0.15 (-7.11, 7.42) | 0.97 | 0.22 (-10.35, 10.8) | 0.97 | 1.2 (-25.78, 28.19) | 0.936 | -0.45 (-12.68, 11.78) | 0.948 | 0.97 (-9.33, 11.26) | 0.864 |
| 214 | Lipoma | 8640 | -0.17 (-7.1, 6.76) | 0.966 | -0.05 (-2.2, 2.11) | 0.97 | 0.89 (-7.04, 8.82) | 0.837 | 0.02 (-1.11, 1.15) | 0.97 | 0.05 (-2.15, 2.24) | 0.97 |
| 214.1 | Lipoma of skin and subcutaneous tissue | 5742 | 0 (-0.22, 0.21) | 0.97 | -0.43 (-5.03, 4.16) | 0.864 | 0.13 (-6.03, 6.29) | 0.97 | 0.18 (-4.62, 4.97) | 0.948 | -0.19 (-8.04, 7.66) | 0.966 |
| 215 | Other benign neoplasm of connective and other soft tissue | 2466 | -0.45 (-12.81, 11.91) | 0.948 | 0.46 (-19.55, 20.47) | 0.967 | 0.04 (-1.83, 1.91) | 0.97 | 0.26 (-12.02, 12.54) | 0.97 | 0.81 (-12.79, 14.41) | 0.915 |
| 216 | Benign neoplasm of skin | 13065 | -0.07 (-3.16, 3.03) | 0.97 | -0.04 (-1.94, 1.86) | 0.97 | -0.13 (-6.36, 6.1) | 0.97 | 0.11 (-4.89, 5.1) | 0.97 | -0.17 (-7.79, 7.44) | 0.967 |
| 217 | Vascular hamartomas and non-neoplastic nevi | 1427 | 0.3 (-12.24, 12.85) | 0.966 | -0.84 (-16.56, 14.88) | 0.923 | 0.02 (-0.76, 0.79) | 0.97 | 0.02 (-1.14, 1.19) | 0.97 | -0.18 (-8.75, 8.39) | 0.97 |
| 217.1 | Nevus, non-neoplastic | 312 | 1.19 (-14.33, 16.71) | 0.89 | -3.29 (-16.34, 9.76) | 0.634 | -1.56 (-75.49, 72.36) | 0.97 | 0.29 (-13.4, 13.98) | 0.97 | 1.43 (-28.54, 31.4) | 0.932 |
| 218 | Benign neoplasm of uterus | 18307 | 0.04 (-1.89, 1.97) | 0.97 | 0.03 (-1.23, 1.28) | 0.97 | 0.1 (-4.68, 4.88) | 0.97 | -0.07 (-3.4, 3.26) | 0.97 | 0.08 (-3.87, 4.04) | 0.97 |
| 218.1 | Uterine leiomyoma | 17472 | 0.15 (-3.26, 3.56) | 0.936 | -0.04 (-1.9, 1.82) | 0.97 | -0.01 (-0.59, 0.56) | 0.97 | -0.13 (-3.81, 3.54) | 0.948 | 0.1 (-4.42, 4.61) | 0.97 |
| 218.2 | Other benign neoplasm of uterus | 1131 | -0.84 (-11.46, 9.79) | 0.887 | 0.77 (-31.14, 32.69) | 0.966 | 1.53 (-32.62, 35.68) | 0.936 | 0.31 (-14.3, 14.91) | 0.97 | -0.57 (-24.18, 23.03) | 0.966 |
| 221 | Benign neoplasm of other female genital organs | 1080 | 0.63 (-25.47, 26.74) | 0.966 | -1.78 (-22.18, 18.63) | 0.874 | 0.7 (-32.43, 33.83) | 0.97 | -0.02 (-1.08, 1.04) | 0.97 | -0.11 (-5.42, 5.19) | 0.97 |
| 222 | Benign neoplasm of male genital organs | 529 | -0.14 (-6.65, 6.37) | 0.97 | 0.57 (-26.35, 27.49) | 0.97 | -1.67 (-74.66, 71.31) | 0.967 | 0.23 (-10.82, 11.29) | 0.97 | -0.03 (-1.65, 1.58) | 0.97 |
| 223 | Benign neoplasm of kidney and other urinary organs | 3991 | -0.39 (-5.34, 4.56) | 0.887 | 0.91 (-6.43, 8.25) | 0.82 | 0.05 (-2.43, 2.53) | 0.97 | 0.03 (-1.46, 1.52) | 0.97 | 0.05 (-2.24, 2.33) | 0.97 |
| 224 | Benign neoplasm of eye | 1080 | 0.18 (-8.11, 8.46) | 0.97 | 0.25 (-11.55, 12.05) | 0.97 | -0.74 (-35.59, 34.11) | 0.97 | -0.13 (-6.49, 6.22) | 0.97 | -1.29 (-14.97, 12.4) | 0.864 |
| 224.1 | Benign neoplasm of eye, uveal | 377 | -0.12 (-5.81, 5.57) | 0.97 | -0.49 (-23.88, 22.89) | 0.97 | -0.96 (-46.11, 44.2) | 0.97 | 0.45 (-17.95, 18.84) | 0.966 | -0.2 (-9.61, 9.21) | 0.97 |
| 225 | Benign neoplasm of brain and other parts of nervous system | 3775 | -0.61 (-5.92, 4.7) | 0.833 | 0.79 (-7.65, 9.24) | 0.864 | 0.18 (-8.19, 8.54) | 0.97 | 0.26 (-10.52, 11.04) | 0.966 | 0.63 (-7, 8.27) | 0.88 |
| 225.1 | Benign neoplasm of brain, cranial nerves, meninges | 3487 | -0.57 (-6.62, 5.49) | 0.864 | 0.75 (-8.75, 10.24) | 0.887 | 0.31 (-14.35, 14.97) | 0.97 | 0.22 (-9.31, 9.74) | 0.967 | 0.55 (-10.59, 11.7) | 0.929 |
| 225.2 | Benign neoplasm of spinal cord, meninges | 246 | -1.51 (-35.39, 32.37) | 0.936 | 2.74 (-36.67, 42.15) | 0.9 | 0.74 (-34.25, 35.73) | 0.97 | 0.08 (-3.56, 3.71) | 0.97 | 1.01 (-46.58, 48.6) | 0.97 |
| 227 | Benign neoplasm of other endocrine glands and related structures | 2490 | 0.24 (-10.21, 10.69) | 0.968 | -0.03 (-1.44, 1.38) | 0.97 | -0.33 (-15.89, 15.23) | 0.97 | -0.17 (-8.45, 8.1) | 0.97 | -0.46 (-12.89, 11.98) | 0.948 |
| 227.1 | Benign neoplasm of adrenal gland | 1115 | 0.09 (-4.16, 4.34) | 0.97 | 0.61 (-24.38, 25.59) | 0.966 | -0.53 (-25.45, 24.4) | 0.97 | -0.19 (-9.29, 8.9) | 0.97 | -0.84 (-11.6, 9.92) | 0.887 |
| 227.2 | Benign neoplasm of parathyroid gland | 258 | 0.32 (-14.92, 15.57) | 0.97 | 0.01 (-0.51, 0.53) | 0.97 | -0.52 (-24.97, 23.93) | 0.97 | -0.28 (-13.66, 13.1) | 0.97 | 0.2 (-9.4, 9.8) | 0.97 |
| 227.3 | Benign neoplasm of pituitary gland and craniopharyngeal duct (pouch) | 960 | 0.13 (-6.06, 6.33) | 0.97 | 0.39 (-18.01, 18.79) | 0.97 | -0.82 (-39.76, 38.11) | 0.97 | -0.19 (-9.27, 8.88) | 0.97 | 0.17 (-7.69, 8.02) | 0.97 |
| 228 | Hemangioma and lymphangioma, any site | 2450 | -0.11 (-5.18, 4.96) | 0.97 | 0.47 (-18.9, 19.84) | 0.966 | -1.3 (-27.39, 24.79) | 0.929 | 0.12 (-5.5, 5.73) | 0.97 | 0.83 (-9.77, 11.44) | 0.887 |
| 229 | Benign neoplasm of unspecified sites | 2123 | 0.07 (-3.2, 3.33) | 0.97 | 0.09 (-4.23, 4.41) | 0.97 | -0.72 (-34.99, 33.54) | 0.97 | 0.01 (-0.69, 0.72) | 0.97 | -0.25 (-12.05, 11.55) | 0.97 |
| 240 | Simple and unspecified goiter | 4078 | 0.35 (-9.21, 9.91) | 0.948 | -0.61 (-13.34, 12.13) | 0.932 | 0.85 (-22.4, 24.11) | 0.948 | -0.25 (-10.42, 9.93) | 0.966 | 0.69 (-7.87, 9.26) | 0.884 |
| 241 | Nontoxic nodular goiter | 8992 | 0.21 (-8.3, 8.72) | 0.966 | -0.72 (-6.96, 5.53) | 0.833 | 0.06 (-2.93, 3.06) | 0.97 | 0.06 (-2.99, 3.12) | 0.97 | 0.04 (-2.02, 2.11) | 0.97 |
| 241.1 | Nontoxic uninodular goiter | 4232 | -0.01 (-0.43, 0.41) | 0.97 | -0.08 (-3.85, 3.69) | 0.97 | -0.33 (-15.78, 15.13) | 0.97 | 0.09 (-4.31, 4.5) | 0.97 | 0.2 (-9.38, 9.78) | 0.97 |
| 241.2 | Nontoxic multinodular goiter | 6011 | 0.1 (-4.61, 4.81) | 0.97 | -0.85 (-9.85, 8.15) | 0.864 | 0.34 (-15.92, 16.61) | 0.97 | 0.18 (-8.17, 8.53) | 0.97 | 0.16 (-7.54, 7.87) | 0.97 |
| 242 | Thyrotoxicosis with or without goiter | 9745 | -0.16 (-6.76, 6.44) | 0.966 | 0.02 (-1.11, 1.16) | 0.97 | 0.67 (-11.77, 13.1) | 0.923 | 0.04 (-1.96, 2.05) | 0.97 | 0.13 (-6.18, 6.45) | 0.97 |
| 242.1 | Graves' disease | 4419 | -0.28 (-7.89, 7.33) | 0.948 | -0.06 (-3, 2.87) | 0.97 | -0.48 (-21.6, 20.64) | 0.967 | 0.38 (-6.78, 7.55) | 0.923 | 0.32 (-12.73, 13.36) | 0.966 |
| 242.2 | Toxic multinodular goiter | 2538 | -0.27 (-12.69, 12.16) | 0.97 | 0.51 (-20.39, 21.4) | 0.966 | 1.35 (-23.96, 26.67) | 0.923 | -0.14 (-6.55, 6.28) | 0.97 | 0.08 (-3.89, 4.06) | 0.97 |
| 242.3 | Exophthalmos | 440 | 0.29 (-13.6, 14.19) | 0.97 | 0.32 (-14.86, 15.5) | 0.97 | -0.52 (-25.11, 24.07) | 0.97 | -0.34 (-16.18, 15.51) | 0.97 | -0.81 (-34.29, 32.66) | 0.966 |
| 244 | Hypothyroidism | 14524 | -0.01 (-0.51, 0.49) | 0.97 | 0.08 (-3.7, 3.86) | 0.97 | -0.09 (-4.46, 4.27) | 0.97 | -0.01 (-0.34, 0.32) | 0.97 | -0.04 (-1.85, 1.78) | 0.97 |
| 244.1 | Secondary hypothyroidism | 1153 | -0.02 (-0.92, 0.88) | 0.97 | 0.16 (-7.55, 7.88) | 0.97 | 1.36 (-27.08, 29.79) | 0.932 | -0.29 (-14.24, 13.65) | 0.97 | -0.01 (-0.71, 0.68) | 0.97 |
| 244.2 | Acquired hypothyroidism | 661 | 0.02 (-1.04, 1.08) | 0.97 | 0.02 (-0.72, 0.75) | 0.97 | -1.28 (-54.02, 51.46) | 0.966 | 0.12 (-5.76, 6.01) | 0.97 | -0.32 (-15.48, 14.84) | 0.97 |
| 244.4 | Hypothyroidism NOS | 13473 | 0.01 (-0.67, 0.7) | 0.97 | 0.05 (-2.16, 2.25) | 0.97 | -0.2 (-8.4, 8) | 0.966 | 0 (-0.06, 0.06) | 0.97 | 0.04 (-1.72, 1.79) | 0.97 |
| 245 | Thyroiditis | 2848 | -0.25 (-10.61, 10.11) | 0.966 | 0.15 (-7, 7.31) | 0.97 | 0.93 (-12.43, 14.29) | 0.9 | 0 (-0.02, 0.02) | 0.97 | 0.07 (-3.38, 3.52) | 0.97 |
| 245.1 | Thyroiditis, acute and subacute | 696 | -1.05 (-14.4, 12.3) | 0.887 | 1.05 (-27.55, 29.66) | 0.948 | 1.2 (-48.32, 50.73) | 0.966 | 0.31 (-14.28, 14.9) | 0.97 | -1.48 (-17.32, 14.37) | 0.865 |
| 245.21 | Chronic lymphocytic thyroiditis | 1959 | 0.14 (-6.67, 6.96) | 0.97 | -0.37 (-15.7, 14.95) | 0.966 | 1.09 (-10.51, 12.7) | 0.864 | -0.19 (-8.87, 8.49) | 0.97 | 0.65 (-6.28, 7.59) | 0.864 |
| 246 | Other disorders of thyroid | 597 | -1.12 (-15.8, 13.55) | 0.89 | 1.69 (-29.96, 33.35) | 0.923 | -0.75 (-36.12, 34.63) | 0.97 | 0.6 (-24.11, 25.31) | 0.966 | 0.11 (-5.15, 5.37) | 0.97 |
| 249 | Secondary diabetes mellitus | 159 | 1.12 (-23.96, 26.19) | 0.936 | -1.38 (-58.39, 55.62) | 0.966 | 0.08 (-3.78, 3.94) | 0.97 | -0.74 (-31.66, 30.19) | 0.966 | -0.94 (-45.04, 43.15) | 0.97 |
| 250 | Diabetes mellitus | 36813 | 0.21 (-1.49, 1.92) | 0.82 | -0.21 (-2.96, 2.54) | 0.89 | -0.09 (-4.41, 4.23) | 0.97 | -0.11 (-2.56, 2.35) | 0.936 | 0.06 (-2.8, 2.92) | 0.97 |
| 250.1 | Type 1 diabetes | 12505 | 0.32 (-2.24, 2.87) | 0.82 | -0.09 (-4.57, 4.38) | 0.97 | -0.16 (-7.55, 7.24) | 0.97 | -0.26 (-2.97, 2.46) | 0.864 | 0.03 (-1.35, 1.4) | 0.97 |
| 250.11 | Type 1 diabetes with ketoacidosis | 994 | 0.37 (-14.83, 15.56) | 0.966 | -0.06 (-2.78, 2.66) | 0.97 | -0.21 (-9.95, 9.54) | 0.97 | -0.3 (-13.24, 12.65) | 0.967 | 1.6 (-3.35, 6.56) | 0.537 |
| 250.12 | Type 1 diabetes with renal manifestations | 988 | -0.47 (-19.73, 18.8) | 0.966 | 0.96 (-25.06, 26.97) | 0.948 | -2 (-27.45, 23.45) | 0.887 | 0.46 (-18.54, 19.46) | 0.966 | 0.52 (-22.19, 23.23) | 0.967 |
| 250.13 | Type 1 diabetes with ophthalmic manifestations | 1615 | -0.18 (-8.73, 8.37) | 0.97 | 0.94 (-11.37, 13.26) | 0.89 | -2.24 (-20.33, 15.85) | 0.82 | 0.15 (-6.92, 7.22) | 0.97 | 0.33 (-15.04, 15.69) | 0.97 |
| 250.14 | Type 1 diabetes with neurological manifestations | 1069 | 0.61 (-12.99, 14.21) | 0.936 | 0.75 (-30.18, 31.68) | 0.966 | -1.12 (-47.14, 44.9) | 0.966 | -0.7 (-13.72, 12.33) | 0.923 | 0.46 (-21.19, 22.12) | 0.97 |
| 250.2 | Type 2 diabetes | 32507 | 0.19 (-1.51, 1.9) | 0.837 | -0.17 (-4.14, 3.79) | 0.937 | -0.13 (-6.47, 6.2) | 0.97 | -0.09 (-3.51, 3.32) | 0.961 | 0.02 (-0.75, 0.78) | 0.97 |
| 250.21 | Type 2 diabetes with ketoacidosis | 358 | 0.42 (-16.89, 17.73) | 0.966 | 0.02 (-1.1, 1.15) | 0.97 | 0.27 (-12.5, 13.04) | 0.97 | -0.46 (-19.23, 18.32) | 0.966 | -0.15 (-7.31, 7.01) | 0.97 |
| 250.22 | Type 2 diabetes with renal manifestations | 3390 | -0.25 (-7.07, 6.57) | 0.948 | 0.1 (-4.57, 4.77) | 0.97 | -0.4 (-17.67, 16.88) | 0.967 | 0.28 (-6.14, 6.7) | 0.937 | -0.09 (-4.43, 4.25) | 0.97 |
| 250.23 | Type 2 diabetes with ophthalmic manifestations | 3453 | -0.02 (-1.02, 0.98) | 0.97 | 0.74 (-8.92, 10.4) | 0.89 | -0.93 (-20.46, 18.59) | 0.932 | -0.13 (-6.18, 5.93) | 0.97 | 0.57 (-11.19, 12.34) | 0.93 |
| 250.24 | Type 2 diabetes with neurological manifestations | 4018 | 0.26 (-8.89, 9.41) | 0.96 | 0.02 (-1.02, 1.07) | 0.97 | -1.07 (-12.47, 10.33) | 0.864 | -0.09 (-4.2, 4.03) | 0.97 | 0.31 (-12.65, 13.28) | 0.966 |
| 250.41 | Impaired fasting glucose | 349 | 0.18 (-8.12, 8.47) | 0.97 | -1.91 (-18.49, 14.68) | 0.833 | 1.05 (-42.17, 44.27) | 0.966 | 0.38 (-17.53, 18.29) | 0.97 | 0.97 (-20.56, 22.49) | 0.936 |
| 250.42 | Other abnormal glucose | 1605 | -0.18 (-8.31, 7.94) | 0.968 | 0.4 (-10.43, 11.23) | 0.948 | 0.23 (-10.71, 11.18) | 0.97 | -0.03 (-1.61, 1.55) | 0.97 | 0.38 (-9.89, 10.64) | 0.948 |
| 250.6 | Polyneuropathy in diabetes | 516 | 0.31 (-14.33, 14.95) | 0.97 | -1.28 (-14.87, 12.31) | 0.864 | -0.85 (-35.89, 34.19) | 0.966 | 0.44 (-17.55, 18.42) | 0.966 | 0.4 (-18.36, 19.15) | 0.97 |
| 250.7 | Diabetic retinopathy | 4762 | 0.11 (-4.91, 5.12) | 0.97 | 0.63 (-6.11, 7.37) | 0.864 | -0.28 (-13.68, 13.12) | 0.97 | -0.33 (-5.29, 4.63) | 0.904 | 0.41 (-7.95, 8.77) | 0.93 |
| 251 | Other disorders of pancreatic internal secretion | 4339 | -0.1 (-4.62, 4.42) | 0.97 | -0.29 (-13.81, 13.24) | 0.97 | -0.25 (-11.85, 11.36) | 0.97 | 0.26 (-10.57, 11.1) | 0.966 | 0.37 (-14.91, 15.65) | 0.966 |
| 251.1 | Hypoglycemia | 4256 | -0.08 (-4.03, 3.86) | 0.97 | -0.14 (-6.65, 6.37) | 0.97 | -0.25 (-11.99, 11.5) | 0.97 | 0.19 (-8.72, 9.1) | 0.97 | 0.33 (-13.39, 14.06) | 0.966 |
| 252 | Disorders of parathyroid gland | 2645 | -0.2 (-9.44, 9.05) | 0.97 | -0.15 (-7.42, 7.11) | 0.97 | -0.17 (-8.25, 7.91) | 0.97 | 0.29 (-7.64, 8.22) | 0.948 | -0.04 (-2.1, 2.01) | 0.97 |
| 252.1 | Hyperparathyroidism | 2139 | -0.27 (-11.53, 10.99) | 0.966 | -0.27 (-13.22, 12.67) | 0.97 | -0.31 (-15.02, 14.4) | 0.97 | 0.45 (-7.9, 8.8) | 0.923 | -0.09 (-4.4, 4.21) | 0.97 |
| 252.2 | Hypoparathyroidism | 513 | -0.23 (-10.93, 10.48) | 0.97 | 0.43 (-19.82, 20.67) | 0.97 | 0.16 (-7.52, 7.84) | 0.97 | 0.04 (-2.02, 2.11) | 0.97 | 0 (-0.15, 0.16) | 0.97 |
| 253 | Disorders of the pituitary gland and its hypothalamic control | 4014 | -0.24 (-8.9, 8.41) | 0.96 | 0.24 (-10.89, 11.36) | 0.97 | 0.7 (-15.1, 16.51) | 0.936 | 0.01 (-0.32, 0.33) | 0.97 | 0.33 (-11.52, 12.18) | 0.96 |
| 253.1 | Pituitary hyperfunction | 2127 | -0.5 (-5.8, 4.8) | 0.864 | 0.64 (-8.74, 10.01) | 0.902 | -0.41 (-19.63, 18.81) | 0.97 | 0.27 (-10.88, 11.42) | 0.966 | 0.1 (-4.84, 5.05) | 0.97 |
| 253.11 | Acromegaly and gigantism | 371 | -0.44 (-21.25, 20.37) | 0.97 | 1.67 (-29.5, 32.84) | 0.923 | 2.19 (-87.9, 92.27) | 0.966 | -0.83 (-35.19, 33.52) | 0.966 | 0.52 (-24.07, 25.11) | 0.97 |
| 253.2 | Pituitary hypofunction | 1150 | 0.3 (-13.8, 14.39) | 0.97 | 0.47 (-21.66, 22.61) | 0.97 | 2.28 (-21.74, 26.3) | 0.863 | -0.88 (-10.26, 8.5) | 0.864 | 0.79 (-16.84, 18.41) | 0.936 |
| 253.3 | Diabetes insipidus | 272 | 1.14 (-29.99, 32.28) | 0.948 | -1.46 (-61.61, 58.69) | 0.966 | 1.92 (-82.58, 86.42) | 0.968 | -0.74 (-35.66, 34.18) | 0.97 | -1.42 (-60.07, 57.22) | 0.966 |
| 253.7 | Other disorders of neurohypophysis | 102 | 0.49 (-22.35, 23.32) | 0.97 | -0.12 (-6.02, 5.77) | 0.97 | 1.33 (-56.93, 59.6) | 0.967 | -0.7 (-26.8, 25.39) | 0.962 | 1.62 (-18.95, 22.18) | 0.887 |
| 255 | Disorders of adrenal glands | 2481 | 0.1 (-4.81, 5.02) | 0.97 | -0.25 (-12.15, 11.65) | 0.97 | 0.93 (-24.31, 26.17) | 0.948 | -0.17 (-8.05, 7.72) | 0.97 | 0.21 (-9.77, 10.19) | 0.97 |
| 255.1 | Adrenal hyperfunction | 100 | 1.23 (-49.56, 52.02) | 0.966 | 1.62 (-65.33, 68.58) | 0.966 | -2.26 (-83.92, 79.4) | 0.961 | -1.32 (-50.36, 47.73) | 0.962 | -0.65 (-31.27, 29.98) | 0.97 |
| 255.11 | Cushing's syndrome | 308 | -0.77 (-33.05, 31.51) | 0.966 | 0.11 (-5.15, 5.37) | 0.97 | 3.84 (-73.38, 81.07) | 0.929 | 0.24 (-11.09, 11.57) | 0.97 | -0.14 (-6.71, 6.44) | 0.97 |
| 255.12 | Hyperaldosteronism | 252 | 1.71 (-20, 23.41) | 0.887 | -1.04 (-50.24, 48.16) | 0.97 | -0.21 (-10.02, 9.6) | 0.97 | -1.44 (-31.6, 28.73) | 0.932 | 0.77 (-35.77, 37.31) | 0.97 |
| 255.21 | Glucocorticoid deficiency | 978 | 0.54 (-14.2, 15.28) | 0.948 | -0.06 (-2.88, 2.76) | 0.97 | 0.68 (-31.58, 32.95) | 0.97 | -0.64 (-13.75, 12.48) | 0.931 | -0.25 (-12.12, 11.62) | 0.97 |
| 255.3 | Adrenogenital disorders | 419 | 0.06 (-2.79, 2.91) | 0.97 | 0.99 (-42.4, 44.37) | 0.968 | 2.93 (-57.59, 63.46) | 0.931 | -0.91 (-25.61, 23.79) | 0.948 | 0.84 (-38.98, 40.67) | 0.97 |
| 256 | Ovarian dysfunction | 3687 | 0.05 (-2.54, 2.65) | 0.97 | -0.41 (-5.81, 4.98) | 0.89 | 0.39 (-15.82, 16.61) | 0.966 | 0.08 (-3.61, 3.76) | 0.97 | -0.37 (-7.82, 7.08) | 0.929 |
| 256.4 | Polycystic ovaries | 2907 | 0.01 (-0.43, 0.45) | 0.97 | -0.33 (-9.22, 8.56) | 0.948 | 0.41 (-16.51, 17.33) | 0.966 | 0.08 (-3.6, 3.75) | 0.97 | -0.46 (-6.31, 5.39) | 0.887 |
| 257 | Testicular dysfunction | 682 | 0.13 (-5.89, 6.14) | 0.97 | -0.09 (-4.42, 4.23) | 0.97 | -1.28 (-54.11, 51.54) | 0.966 | 0.12 (-5.45, 5.68) | 0.97 | 0.26 (-11.94, 12.46) | 0.97 |
| 257.1 | Testicular hypofunction | 624 | -0.14 (-6.81, 6.53) | 0.97 | 0.18 (-8.33, 8.69) | 0.97 | -0.57 (-27.53, 26.39) | 0.97 | 0.16 (-7.41, 7.74) | 0.97 | -0.04 (-2.08, 1.99) | 0.97 |
| 258 | Iatrogenic endocrine disorders | 734 | 0.77 (-7.41, 8.94) | 0.864 | -1.08 (-14.71, 12.55) | 0.886 | -0.78 (-37.88, 36.31) | 0.97 | -0.17 (-8.39, 8.05) | 0.97 | 0.15 (-6.83, 7.12) | 0.97 |
| 259 | Other endocrine disorders | 3121 | -0.07 (-3.62, 3.47) | 0.97 | 0.36 (-14.42, 15.14) | 0.966 | 0.01 (-0.58, 0.6) | 0.97 | -0.08 (-4.02, 3.85) | 0.97 | 0.32 (-13.53, 14.16) | 0.967 |
| 259.2 | Carcinoid syndrome | 416 | 0.12 (-5.59, 5.83) | 0.97 | 0.65 (-16.92, 18.21) | 0.948 | -1.32 (-16.92, 14.29) | 0.878 | -0.06 (-2.68, 2.57) | 0.97 | 0.36 (-15.55, 16.28) | 0.967 |
| 259.3 | Delay in sexual development and puberty NEC | 281 | -0.55 (-12.96, 11.86) | 0.936 | -0.01 (-0.28, 0.27) | 0.97 | 0.95 (-38.29, 40.19) | 0.966 | 0.35 (-14.17, 14.87) | 0.966 | -0.21 (-10.06, 9.65) | 0.97 |
| 259.4 | Precocious sexual development and puberty NEC | 403 | -0.08 (-3.99, 3.82) | 0.97 | -0.13 (-6.08, 5.83) | 0.97 | -0.18 (-8.58, 8.22) | 0.97 | 0.17 (-7.7, 8.04) | 0.97 | 0.36 (-16.56, 17.27) | 0.97 |
| 260 | Protein-calorie malnutrition | 4568 | 0.16 (-6.43, 6.75) | 0.966 | -0.12 (-5.95, 5.7) | 0.97 | -0.35 (-14.7, 14) | 0.966 | -0.05 (-2.39, 2.29) | 0.97 | -0.01 (-0.46, 0.44) | 0.97 |
| 260.6 | Anorexia | 4360 | 0.05 (-2.28, 2.38) | 0.97 | -0.13 (-6.13, 5.88) | 0.97 | -0.44 (-12.43, 11.55) | 0.948 | 0.08 (-3.69, 3.85) | 0.97 | 0.02 (-0.9, 0.94) | 0.97 |
| 261 | Vitamin deficiency | 6683 | 0.18 (-3.63, 3.99) | 0.932 | -0.04 (-1.88, 1.8) | 0.97 | -0.24 (-11.66, 11.17) | 0.97 | -0.12 (-5.24, 4.99) | 0.966 | 0.12 (-5.52, 5.76) | 0.97 |
| 261.1 | Vitamin A deficiency | 343 | 0.12 (-5.66, 5.9) | 0.97 | -0.13 (-6.29, 6.03) | 0.97 | 0.4 (-18.53, 19.33) | 0.97 | -0.14 (-6.91, 6.62) | 0.97 | 1.01 (-20.06, 22.08) | 0.932 |
| 261.2 | Vitamin B-complex deficiencies | 1473 | 0.15 (-7.17, 7.48) | 0.97 | 0.29 (-13.21, 13.78) | 0.97 | -0.16 (-7.73, 7.41) | 0.97 | -0.25 (-10.7, 10.2) | 0.966 | -0.11 (-5.38, 5.16) | 0.97 |
| 261.4 | Vitamin D deficiency | 4105 | 0.1 (-3.83, 4.02) | 0.966 | -0.2 (-4.85, 4.44) | 0.937 | -0.13 (-6.29, 6.03) | 0.97 | 0.03 (-1.17, 1.22) | 0.97 | -0.05 (-2.3, 2.2) | 0.97 |
| 261.41 | Rickets or osteomalacia | 758 | 0.26 (-12.13, 12.65) | 0.97 | -1.02 (-28.8, 26.76) | 0.948 | 0.05 (-2.09, 2.18) | 0.97 | 0.24 (-10.99, 11.46) | 0.97 | -1 (-28.14, 26.14) | 0.948 |
| 262 | Mineral deficiency NEC | 533 | -0.03 (-1.56, 1.49) | 0.97 | 0.02 (-1.02, 1.06) | 0.97 | -1.9 (-18.46, 14.66) | 0.833 | 0.37 (-13.85, 14.6) | 0.963 | -0.54 (-20.19, 19.1) | 0.961 |
| 263 | Other nutritional deficiency | 1992 | -0.23 (-5.57, 5.1) | 0.937 | -0.09 (-4.56, 4.38) | 0.97 | -0.21 (-10.18, 9.76) | 0.97 | 0.31 (-3.65, 4.27) | 0.887 | 0.07 (-3.35, 3.49) | 0.97 |
| 264 | Lack of normal physiological development | 2961 | 0.11 (-4.9, 5.11) | 0.97 | 0.06 (-2.72, 2.83) | 0.97 | -0.57 (-7.86, 6.72) | 0.887 | -0.03 (-1.56, 1.49) | 0.97 | 0.59 (-1.75, 2.94) | 0.634 |
| 264.3 | Delayed milestones | 1318 | 0 (-0.12, 0.11) | 0.97 | 0.47 (-5.52, 6.47) | 0.887 | -1 (-9.69, 7.69) | 0.833 | -0.05 (-2.31, 2.22) | 0.97 | 0.89 (-1.19, 2.97) | 0.408 |
| 264.9 | Lack of normal physiological development, unspecified | 1970 | 0.07 (-3.37, 3.52) | 0.97 | -0.06 (-2.94, 2.82) | 0.97 | -0.53 (-14.87, 13.82) | 0.948 | 0.05 (-2.16, 2.25) | 0.97 | 0.19 (-8.74, 9.11) | 0.97 |
| 269 | Proteinuria | 901 | 0.21 (-9.68, 10.1) | 0.97 | 0.66 (-17.29, 18.61) | 0.948 | 0.9 (-36.01, 37.8) | 0.966 | -0.64 (-7.53, 6.25) | 0.865 | 0.17 (-7.76, 8.09) | 0.97 |
| 270 | Disorders of protein plasma/amino-acid transport and metabolism | 2110 | -0.2 (-9.01, 8.61) | 0.967 | 0.14 (-6.58, 6.86) | 0.97 | -0.08 (-3.87, 3.71) | 0.97 | 0.16 (-7.63, 7.96) | 0.97 | -0.04 (-1.78, 1.71) | 0.97 |
| 270.1 | Disturbances of amino-acid transport | 233 | -0.12 (-5.56, 5.33) | 0.97 | -1.14 (-49.17, 46.88) | 0.966 | 3.13 (-81.94, 88.19) | 0.948 | 0.22 (-10.17, 10.61) | 0.97 | 2.21 (-26.7, 31.11) | 0.89 |
| 270.32 | Paraproteinemia | 1025 | -0.18 (-8.52, 8.17) | 0.97 | 1.3 (-7.06, 9.65) | 0.774 | 0.16 (-7.19, 7.5) | 0.97 | -0.32 (-11.73, 11.1) | 0.961 | 0.18 (-8.52, 8.89) | 0.97 |
| 270.33 | Amyloidosis | 355 | -0.03 (-1.32, 1.27) | 0.97 | -0.25 (-11.89, 11.4) | 0.97 | -0.97 (-43.23, 41.3) | 0.967 | 0.3 (-13.93, 14.53) | 0.97 | 0.66 (-26.51, 27.83) | 0.966 |
| 270.35 | Macroglobulinemia | 358 | 0.69 (-13.23, 14.61) | 0.929 | -0.86 (-24.13, 22.42) | 0.948 | -2.35 (-21.29, 16.59) | 0.82 | 0.24 (-10.97, 11.44) | 0.97 | -0.82 (-23.27, 21.62) | 0.948 |
| 271 | Disorders of carbohydrate transport and metabolism | 1310 | -0.13 (-6.07, 5.82) | 0.97 | 0.57 (-22.97, 24.11) | 0.966 | 0.44 (-20.54, 21.42) | 0.97 | -0.27 (-12.9, 12.36) | 0.97 | 0.34 (-15.56, 16.23) | 0.97 |
| 271.3 | Intestinal disaccharidase deficiencies and disaccharide malabsorption | 1155 | -0.39 (-17.2, 16.43) | 0.967 | 0.07 (-3.14, 3.28) | 0.97 | 1.22 (-31.86, 34.29) | 0.948 | 0.1 (-4.65, 4.85) | 0.97 | 0.44 (-20.27, 21.14) | 0.97 |
| 271.9 | Other disorders of carbohydrate transport and metabolism | 162 | 2.63 (-51.43, 56.7) | 0.93 | 3.16 (-82.75, 89.07) | 0.948 | -2.76 (-133.13, 127.61) | 0.97 | -3.37 (-39.2, 32.46) | 0.864 | 2.14 (-91.47, 95.76) | 0.967 |
| 272 | Disorders of lipoid metabolism | 41222 | -0.03 (-1.55, 1.49) | 0.97 | -0.14 (-1.92, 1.64) | 0.884 | -0.3 (-2.76, 2.15) | 0.82 | 0.15 (-0.43, 0.72) | 0.634 | 0.04 (-1.73, 1.81) | 0.97 |
| 272.1 | Hyperlipidemia | 8676 | 0.05 (-2.29, 2.39) | 0.97 | 0.01 (-0.55, 0.57) | 0.97 | -0.59 (-3.53, 2.34) | 0.705 | 0.05 (-2.41, 2.51) | 0.97 | 0.08 (-3.77, 3.93) | 0.97 |
| 272.11 | Hypercholesterolemia | 35565 | -0.05 (-2.12, 2.02) | 0.966 | -0.1 (-2.82, 2.62) | 0.948 | -0.25 (-2.92, 2.41) | 0.864 | 0.14 (-0.97, 1.25) | 0.82 | 0.03 (-1.52, 1.59) | 0.97 |
| 272.12 | Hyperglyceridemia | 291 | -0.84 (-40.78, 39.09) | 0.97 | -0.11 (-5.53, 5.3) | 0.97 | 1.54 (-71.45, 74.54) | 0.97 | 0.62 (-28.66, 29.9) | 0.97 | -0.72 (-34.82, 33.38) | 0.97 |
| 272.13 | Mixed hyperlipidemia | 1324 | 0.33 (-13.42, 14.09) | 0.966 | -1.59 (-9.45, 6.27) | 0.705 | 0.42 (-19.66, 20.51) | 0.97 | 0.23 (-10.51, 10.96) | 0.97 | -0.15 (-7.47, 7.16) | 0.97 |
| 272.9 | Unspecified disorder of lipoid metabolism | 949 | 0.14 (-6.67, 6.95) | 0.97 | -0.59 (-12.46, 11.28) | 0.929 | -0.55 (-23.39, 22.28) | 0.966 | 0.19 (-8.83, 9.22) | 0.97 | 0.74 (-5.94, 7.41) | 0.84 |
| 274 | Gout and other crystal arthropathies | 8028 | 0.12 (-5.43, 5.66) | 0.97 | -0.53 (-5.19, 4.12) | 0.833 | -0.19 (-9.2, 8.82) | 0.97 | 0.13 (-5.39, 5.65) | 0.966 | -0.12 (-5.62, 5.38) | 0.97 |
| 274.1 | Gout | 4013 | -0.01 (-0.33, 0.32) | 0.97 | -0.29 (-12.08, 11.5) | 0.966 | 0.72 (-13.97, 15.41) | 0.93 | 0 (-0.1, 0.1) | 0.97 | 0.01 (-0.33, 0.34) | 0.97 |
| 274.11 | Gouty arthropathy | 4013 | 0.13 (-6.19, 6.46) | 0.97 | -1.04 (-5.15, 3.07) | 0.634 | -0.63 (-17.77, 16.51) | 0.948 | 0.41 (-4.58, 5.4) | 0.882 | -0.36 (-10.13, 9.41) | 0.948 |
| 274.2 | Crystal arthropathies | 262 | -0.55 (-24.67, 23.57) | 0.967 | -0.78 (-37.88, 36.31) | 0.97 | -1.5 (-72.25, 69.25) | 0.97 | 0.89 (-19.16, 20.94) | 0.936 | 0.72 (-29.48, 30.92) | 0.966 |
| 274.21 | Chondrocalcinosis | 1062 | -0.01 (-0.44, 0.43) | 0.97 | 0.23 (-10.72, 11.18) | 0.97 | -2.04 (-23.71, 19.63) | 0.864 | 0.22 (-10.03, 10.46) | 0.97 | -0.43 (-19.04, 18.18) | 0.967 |
| 275 | Disorders of mineral metabolism | 3083 | -0.07 (-3.2, 3.07) | 0.97 | -0.36 (-7.95, 7.23) | 0.932 | 0.13 (-5.81, 6.06) | 0.97 | 0.2 (-5.12, 5.51) | 0.948 | -0.28 (-7.88, 7.32) | 0.948 |
| 275.1 | Disorders of iron metabolism | 473 | -0.16 (-7.71, 7.39) | 0.97 | 1.12 (-29.47, 31.72) | 0.948 | -0.14 (-6.63, 6.35) | 0.97 | -0.25 (-12.27, 11.77) | 0.97 | 0.12 (-5.33, 5.56) | 0.97 |
| 275.3 | Disorders of magnesium metabolism | 798 | -0.17 (-7.75, 7.4) | 0.967 | -0.18 (-8.46, 8.11) | 0.97 | 0.12 (-5.76, 6.01) | 0.97 | 0.22 (-9.04, 9.49) | 0.966 | -0.25 (-11.02, 10.53) | 0.967 |
| 275.5 | Disorders of calcium/phosphorus metabolism | 1656 | -0.08 (-4.08, 3.91) | 0.97 | -0.53 (-7.26, 6.2) | 0.887 | -0.45 (-18.95, 18.05) | 0.966 | 0.38 (-3.65, 4.4) | 0.864 | -0.27 (-11.38, 10.84) | 0.966 |
| 275.53 | Disorders of phosphorus metabolism | 114 | 1.27 (-50.96, 53.5) | 0.966 | 0.55 (-25.57, 26.67) | 0.97 | 4.15 (-108.69, 116.98) | 0.948 | -1.95 (-30.03, 26.13) | 0.9 | -1.84 (-77.53, 73.86) | 0.966 |
| 276 | Disorders of fluid, electrolyte, and acid-base balance | 16318 | 0.05 (-2.53, 2.64) | 0.97 | 0.05 (-2.51, 2.62) | 0.97 | -0.25 (-6.97, 6.48) | 0.948 | -0.04 (-1.8, 1.72) | 0.97 | -0.03 (-1.29, 1.24) | 0.97 |
| 276.1 | Electrolyte imbalance | 697 | 0.22 (-8.92, 9.36) | 0.966 | -0.43 (-12.07, 11.22) | 0.948 | 0.17 (-8.06, 8.41) | 0.97 | -0.09 (-4.2, 4.02) | 0.97 | 0.1 (-4.46, 4.66) | 0.97 |
| 276.11 | Hyperosmolality and/or hypernatremia | 508 | -0.46 (-8.98, 8.06) | 0.923 | -0.83 (-9.71, 8.04) | 0.864 | 0.39 (-18.14, 18.92) | 0.97 | 0.8 (-3.15, 4.75) | 0.705 | -1.26 (-6.24, 3.73) | 0.634 |
| 276.12 | Hyposmolality and/or hyponatremia | 6048 | -0.01 (-0.58, 0.56) | 0.97 | -0.01 (-0.6, 0.57) | 0.97 | 0 (-0.21, 0.22) | 0.97 | 0.02 (-0.77, 0.8) | 0.97 | -0.23 (-3.13, 2.68) | 0.887 |
| 276.13 | Hyperpotassemia | 2660 | 0.12 (-4.63, 4.86) | 0.966 | -0.22 (-8.54, 8.11) | 0.963 | 0.14 (-6.57, 6.85) | 0.97 | -0.05 (-2.6, 2.49) | 0.97 | -0.1 (-4.68, 4.49) | 0.97 |
| 276.14 | Hypopotassemia | 6140 | -0.18 (-2.08, 1.72) | 0.864 | 0.15 (-5.12, 5.41) | 0.96 | 0.19 (-8.06, 8.44) | 0.967 | 0.09 (-3.53, 3.7) | 0.966 | 0.03 (-1.56, 1.63) | 0.97 |
| 276.41 | Acidosis | 2158 | 0.52 (-5.04, 6.09) | 0.864 | -0.15 (-7.07, 6.78) | 0.97 | -1.64 (-14.85, 11.57) | 0.82 | -0.21 (-9.48, 9.06) | 0.967 | -0.21 (-10.05, 9.63) | 0.97 |
| 276.42 | Alkalosis | 227 | 0.71 (-32.97, 34.4) | 0.97 | 0 (-0.09, 0.08) | 0.97 | 2.54 (-117.45, 122.52) | 0.97 | -1.15 (-51.96, 49.65) | 0.968 | -0.55 (-26.41, 25.32) | 0.97 |
| 276.6 | Fluid overload | 310 | 0.32 (-12.98, 13.63) | 0.966 | 0 (-0.23, 0.22) | 0.97 | 0.12 (-5.44, 5.68) | 0.97 | -0.34 (-14.42, 13.73) | 0.966 | -0.36 (-17.58, 16.85) | 0.97 |
| 276.8 | Polydipsia | 142 | -2.51 (-22.75, 17.73) | 0.82 | 0.71 (-32.98, 34.4) | 0.97 | -1.41 (-68.14, 65.32) | 0.97 | 2.29 (-17.6, 22.17) | 0.833 | 1.35 (-54.2, 56.89) | 0.966 |
| 277 | Other disorders of metabolism | 2405 | -0.98 (-4, 2.05) | 0.537 | 0.5 (-20.08, 21.07) | 0.966 | 0.81 (-32.41, 34.02) | 0.966 | 0.67 (-6.32, 7.65) | 0.862 | -0.52 (-14.74, 13.69) | 0.948 |
| 277.1 | Disorders of porphyrin metabolism | 277 | -0.26 (-12.54, 12.02) | 0.97 | -0.21 (-9.94, 9.53) | 0.97 | 4.07 (-79.41, 87.54) | 0.93 | -0.4 (-19.19, 18.39) | 0.97 | -0.21 (-10.24, 9.81) | 0.97 |
| 277.4 | Disorders of bilirubin excretion | 373 | -1.34 (-29.64, 26.97) | 0.932 | 2.65 (-25.57, 30.88) | 0.864 | -1.04 (-50, 47.93) | 0.97 | 0.16 (-7.48, 7.8) | 0.97 | -2.42 (-28.16, 23.32) | 0.864 |
| 277.5 | Other disorders of lipoid metabolism | 562 | -0.22 (-10.45, 10.02) | 0.97 | 0.14 (-6.56, 6.84) | 0.97 | -0.96 (-43.07, 41.15) | 0.967 | 0.31 (-14.47, 15.09) | 0.97 | -0.91 (-19.69, 17.86) | 0.93 |
| 277.51 | Lipoprotein disorders | 224 | -0.03 (-1.6, 1.53) | 0.97 | -0.62 (-29.81, 28.57) | 0.97 | -2.1 (-53.83, 49.63) | 0.942 | 0.69 (-27.94, 29.33) | 0.966 | 0.95 (-38.02, 39.91) | 0.966 |
| 278 | Overweight, obesity and other hyperalimentation | 43024 | -0.16 (-1.44, 1.12) | 0.82 | 0.1 (-3.84, 4.05) | 0.963 | -0.07 (-3.48, 3.34) | 0.97 | 0.13 (-1.24, 1.5) | 0.864 | 0.07 (-3.06, 3.21) | 0.966 |
| 278.1 | Obesity | 42870 | -0.15 (-1.41, 1.12) | 0.833 | 0.1 (-3.88, 4.07) | 0.966 | -0.1 (-5.04, 4.83) | 0.97 | 0.12 (-1.2, 1.44) | 0.864 | 0.08 (-3.35, 3.51) | 0.966 |
| 279 | Disorders involving the immune mechanism | 1363 | 0.63 (-6.04, 7.3) | 0.864 | 0.31 (-14.42, 15.04) | 0.97 | -0.29 (-13.86, 13.29) | 0.97 | -0.72 (-7.81, 6.37) | 0.852 | -0.23 (-10.89, 10.44) | 0.97 |
| 279.1 | Immunity deficiency | 707 | 0.22 (-10.28, 10.73) | 0.97 | 0.09 (-3.99, 4.16) | 0.97 | 0.13 (-5.9, 6.16) | 0.97 | -0.28 (-13.75, 13.18) | 0.97 | 0.06 (-2.99, 3.12) | 0.97 |
| 279.11 | Deficiency of humoral immunity | 517 | 0.74 (-19.45, 20.94) | 0.948 | 0.39 (-17.95, 18.72) | 0.97 | 0.6 (-27.81, 29.01) | 0.97 | -1.04 (-18.3, 16.21) | 0.913 | -0.02 (-0.75, 0.72) | 0.97 |
| 279.7 | Other immunological findings | 163 | 0.48 (-22.03, 22.98) | 0.97 | 0.29 (-13.47, 14.05) | 0.97 | -0.72 (-34.56, 33.13) | 0.97 | -0.53 (-25.58, 24.52) | 0.97 | -1.07 (-32.81, 30.68) | 0.952 |
| 279.8 | Other specified disorders involving the immune mechanism | 103 | 0.64 (-29.41, 30.68) | 0.97 | 0.18 (-8.39, 8.76) | 0.97 | 2.4 (-110.01, 114.82) | 0.97 | -0.91 (-38.3, 36.48) | 0.966 | 1.52 (-39.88, 42.92) | 0.948 |
| 280 | Iron deficiency anemias | 9001 | -0.25 (-5.47, 4.96) | 0.93 | 0.07 (-3.17, 3.31) | 0.97 | -0.86 (-9.98, 8.27) | 0.864 | 0.37 (-3.59, 4.33) | 0.864 | -0.25 (-9.93, 9.42) | 0.963 |
| 280.1 | Iron deficiency anemias, unspecified or not due to blood loss | 7709 | -0.21 (-8.14, 7.72) | 0.962 | 0.03 (-1.35, 1.41) | 0.97 | -1.05 (-12.19, 10.1) | 0.864 | 0.38 (-3.64, 4.39) | 0.864 | -0.35 (-9.54, 8.84) | 0.946 |
| 280.2 | Iron deficiency anemia secondary to blood loss (chronic) | 1890 | -0.21 (-7.61, 7.19) | 0.96 | 0.3 (-12.21, 12.82) | 0.966 | 0.16 (-7.57, 7.9) | 0.97 | 0.07 (-3.04, 3.17) | 0.97 | 0.1 (-4.73, 4.93) | 0.97 |
| 281 | Other deficiency anemia | 2145 | 0.04 (-1.86, 1.94) | 0.97 | 1.03 (-7.96, 10.02) | 0.833 | 0.7 (-28.02, 29.41) | 0.966 | -0.57 (-6.67, 5.52) | 0.864 | -0.01 (-0.62, 0.6) | 0.97 |
| 281.1 | Megaloblastic anemia | 163 | 0.35 (-16.35, 17.05) | 0.97 | 0.95 (-40.31, 42.2) | 0.967 | -0.71 (-34.12, 32.71) | 0.97 | -0.57 (-27.23, 26.09) | 0.97 | -0.48 (-23.18, 22.22) | 0.97 |
| 281.11 | Pernicious anemia | 552 | -0.25 (-12.3, 11.79) | 0.97 | 1.68 (-35.77, 39.13) | 0.936 | 3 (-49.32, 55.32) | 0.918 | -0.81 (-29.52, 27.89) | 0.96 | 0.84 (-33.65, 35.32) | 0.966 |
| 281.12 | Other vitamin B12 deficiency anemia | 1049 | 0.2 (-9.37, 9.78) | 0.97 | 0.47 (-18.77, 19.7) | 0.966 | 0.4 (-18.43, 19.23) | 0.97 | -0.46 (-10.06, 9.14) | 0.932 | 0.09 (-3.99, 4.16) | 0.97 |
| 281.13 | Folate-deficiency anemia | 337 | -0.35 (-16.68, 15.99) | 0.97 | 0.97 (-38.91, 40.84) | 0.966 | -1.37 (-58.94, 56.2) | 0.966 | 0.11 (-5.19, 5.42) | 0.97 | -1 (-28.12, 26.13) | 0.948 |
| 281.9 | Deficiency anemias | 184 | 0.62 (-28.63, 29.87) | 0.97 | 0.4 (-18.43, 19.23) | 0.97 | 0.28 (-13.06, 13.63) | 0.97 | -0.85 (-40.49, 38.8) | 0.97 | -1 (-48.38, 46.37) | 0.97 |
| 283 | Acquired hemolytic anemias | 649 | 0.08 (-3.69, 3.85) | 0.97 | -0.42 (-20.5, 19.66) | 0.97 | -1.56 (-33.65, 30.52) | 0.93 | 0.44 (-17.56, 18.44) | 0.966 | -0.41 (-19.81, 18.99) | 0.97 |
| 283.1 | Autoimmune hemolytic anemias | 333 | 0.33 (-15.13, 15.79) | 0.97 | -0.8 (-33.63, 32.03) | 0.966 | -2.94 (-21.89, 16.01) | 0.774 | 0.58 (-15.31, 16.47) | 0.948 | -0.88 (-20.49, 18.73) | 0.936 |
| 283.2 | Non-autoimmune hemolytic anemias | 104 | -1.92 (-22.36, 18.52) | 0.864 | 1.61 (-34.9, 38.11) | 0.937 | -3.87 (-40.13, 32.4) | 0.845 | 2.22 (-17.06, 21.49) | 0.833 | 1.14 (-45.85, 48.13) | 0.966 |
| 284 | Aplastic anemia | 1104 | 0.04 (-1.73, 1.8) | 0.97 | 0.35 (-16.11, 16.81) | 0.97 | -0.65 (-27.65, 26.34) | 0.966 | -0.06 (-2.67, 2.56) | 0.97 | -1.06 (-9.59, 7.47) | 0.82 |
| 285 | Other anemias | 24970 | -0.03 (-1.37, 1.32) | 0.97 | 0.05 (-2.52, 2.63) | 0.97 | -0.26 (-5.21, 4.68) | 0.923 | 0.05 (-2.46, 2.57) | 0.97 | 0.03 (-1.29, 1.34) | 0.97 |
| 285.1 | Acute posthemorrhagic anemia | 187 | -0.72 (-20.21, 18.78) | 0.948 | 0.41 (-18.98, 19.8) | 0.97 | 1.68 (-44.07, 47.43) | 0.948 | 0.19 (-8.63, 9) | 0.97 | 0.33 (-15.37, 16.03) | 0.97 |
| 285.2 | Anemia of chronic disease | 1276 | -0.19 (-7.89, 7.52) | 0.966 | 0.1 (-4.8, 5.01) | 0.97 | 0.12 (-5.58, 5.82) | 0.97 | 0.13 (-6.18, 6.45) | 0.97 | -0.1 (-4.8, 4.6) | 0.97 |
| 285.22 | Anemia in neoplastic disease | 3640 | -0.21 (-2.09, 1.68) | 0.84 | 0.23 (-4.48, 4.94) | 0.93 | -0.07 (-3.23, 3.09) | 0.97 | 0.13 (-2.65, 2.92) | 0.931 | -0.09 (-4.41, 4.23) | 0.97 |
| 285.3 | Sideroblastic anemia | 285 | -0.19 (-9.18, 8.8) | 0.97 | 0.53 (-24.46, 25.52) | 0.97 | 0.34 (-15.7, 16.38) | 0.97 | -0.07 (-3.41, 3.27) | 0.97 | -1.56 (-21.41, 18.3) | 0.887 |
| 286 | Coagulation defects | 4131 | -0.13 (-5.78, 5.52) | 0.967 | -0.12 (-5.85, 5.61) | 0.97 | -0.48 (-11.15, 10.19) | 0.936 | 0.29 (-3.4, 3.98) | 0.887 | -0.01 (-0.68, 0.66) | 0.97 |
| 286.4 | Acquired coagulation factor deficiency | 470 | -0.25 (-12.14, 11.63) | 0.97 | -1.14 (-22.45, 20.17) | 0.923 | 0.36 (-16.46, 17.17) | 0.97 | 0.55 (-11.71, 12.81) | 0.936 | 0.66 (-17.43, 18.76) | 0.948 |
| 286.5 | Hemorrhagic disorder due to intrinsic circulating anticoagulants | 503 | -0.12 (-5.69, 5.45) | 0.97 | -0.95 (-17.82, 15.93) | 0.919 | -0.09 (-4.15, 3.98) | 0.97 | 0.54 (-10.6, 11.68) | 0.931 | -0.8 (-12.77, 11.17) | 0.904 |
| 286.7 | Other and unspecified coagulation defects | 1088 | -0.37 (-15.68, 14.94) | 0.966 | -0.56 (-23.53, 22.41) | 0.966 | 0.62 (-26.39, 27.62) | 0.967 | 0.49 (-12.85, 13.83) | 0.948 | 0.66 (-17.29, 18.61) | 0.948 |
| 287 | Purpura and other hemorrhagic conditions | 6041 | 0.05 (-2.25, 2.35) | 0.97 | 0.91 (-6.4, 8.21) | 0.82 | 0.64 (-16.67, 17.94) | 0.948 | -0.52 (-5.07, 4.02) | 0.833 | 0.64 (-6.21, 7.5) | 0.864 |
| 287.1 | Spontaneous ecchymoses | 305 | -1.93 (-22.43, 18.57) | 0.864 | 2.14 (-41.79, 46.07) | 0.93 | -1.19 (-57.29, 54.92) | 0.97 | 1.24 (-26.4, 28.88) | 0.936 | -0.69 (-33.53, 32.14) | 0.97 |
| 287.2 | Allergic purpura | 1102 | 0 (-0.08, 0.08) | 0.97 | 0.66 (-28.49, 29.81) | 0.968 | -0.33 (-16.1, 15.43) | 0.97 | -0.2 (-9.68, 9.28) | 0.97 | 1.01 (-19.83, 21.85) | 0.931 |
| 287.3 | Thrombocytopenia | 2049 | 0.46 (-12.11, 13.03) | 0.948 | 0.01 (-0.49, 0.52) | 0.97 | 2.64 (-7.83, 13.12) | 0.634 | -0.94 (-8.49, 6.62) | 0.82 | 0.52 (-20.81, 21.85) | 0.966 |
| 287.31 | Primary thrombocytopenia | 1139 | 0.15 (-7, 7.3) | 0.97 | 0.9 (-19.26, 21.05) | 0.936 | 0.87 (-34.8, 36.53) | 0.966 | -0.67 (-13.24, 11.89) | 0.923 | 0.3 (-13.75, 14.34) | 0.97 |
| 287.32 | Secondary thrombocytopenia | 248 | 1.11 (-10.67, 12.88) | 0.864 | 0.62 (-28.83, 30.07) | 0.97 | -4.4 (-20.67, 11.87) | 0.608 | -0.66 (-18.67, 17.35) | 0.948 | -0.5 (-24.33, 23.32) | 0.97 |
| 287.4 | Qualitative platelet defects | 110 | -2.03 (-85.62, 81.56) | 0.966 | 1.48 (-68.39, 71.35) | 0.97 | -0.83 (-39.85, 38.2) | 0.97 | 1.53 (-69.79, 72.84) | 0.97 | -1.44 (-69.32, 66.45) | 0.97 |
| 288 | Diseases of white blood cells | 1791 | 0.04 (-1.8, 1.88) | 0.97 | 0.17 (-7.68, 8.01) | 0.97 | -0.02 (-1.09, 1.05) | 0.97 | -0.11 (-5.36, 5.13) | 0.97 | 0.11 (-5.16, 5.38) | 0.97 |
| 288.3 | Eosinophilia | 303 | -0.71 (-34.2, 32.78) | 0.97 | -2.75 (-64.54, 59.03) | 0.936 | 0.64 (-29.74, 31.02) | 0.97 | 1.8 (-38.7, 42.31) | 0.936 | -1.94 (-70.19, 66.32) | 0.96 |
| 289 | Other diseases of blood and blood-forming organs | 10334 | -0.27 (-6.18, 5.65) | 0.935 | 0.32 (-12.06, 12.7) | 0.963 | -0.13 (-6.45, 6.18) | 0.97 | 0.16 (-6.76, 7.08) | 0.967 | 0.33 (-8.58, 9.23) | 0.948 |
| 289.4 | Lymphadenitis | 8567 | -0.26 (-7.22, 6.71) | 0.948 | 0.35 (-13.98, 14.67) | 0.966 | 0.35 (-16.15, 16.85) | 0.97 | 0.05 (-2.4, 2.51) | 0.97 | 0.34 (-8.79, 9.47) | 0.948 |
| 289.5 | Diseases of spleen | 378 | -2.04 (-23.71, 19.63) | 0.864 | 0.39 (-18, 18.77) | 0.97 | -2.61 (-110.16, 104.94) | 0.966 | 2.32 (-21.33, 25.97) | 0.858 | -0.78 (-37.76, 36.2) | 0.97 |
| 289.8 | Polycythemia, secondary | 912 | 0.42 (-18.12, 18.97) | 0.968 | -0.22 (-10.39, 9.96) | 0.97 | -1.82 (-51.31, 47.68) | 0.948 | -0.07 (-3.34, 3.2) | 0.97 | 0.67 (-26.98, 28.32) | 0.966 |
| 290 | Delirium dementia and amnestic and other cognitive disorders | 12979 | -0.04 (-1.76, 1.69) | 0.97 | 0.27 (-5.28, 5.81) | 0.931 | 0.36 (-9.47, 10.19) | 0.948 | -0.13 (-4.79, 4.53) | 0.96 | 0.05 (-2.5, 2.61) | 0.97 |
| 290.1 | Dementias | 1590 | 0.02 (-0.93, 0.97) | 0.97 | 0.28 (-11.52, 12.08) | 0.966 | -0.14 (-6.64, 6.36) | 0.97 | -0.13 (-6.06, 5.81) | 0.97 | 0.24 (-10.37, 10.85) | 0.967 |
| 290.11 | Alzheimer's disease | 5298 | -0.06 (-2.92, 2.8) | 0.97 | 0.18 (-8.54, 8.9) | 0.97 | -0.12 (-6, 5.75) | 0.97 | 0.01 (-0.36, 0.37) | 0.97 | 0.04 (-1.8, 1.87) | 0.97 |
| 290.12 | Dementia with cerebral degenerations | 308 | 0.13 (-5.94, 6.19) | 0.97 | 0.6 (-23.97, 25.16) | 0.966 | -0.89 (-37.39, 35.62) | 0.966 | -0.18 (-8.91, 8.54) | 0.97 | -0.1 (-4.77, 4.57) | 0.97 |
| 290.16 | Vascular dementia | 1452 | -0.1 (-4.61, 4.42) | 0.97 | 0.07 (-3.18, 3.31) | 0.97 | -0.05 (-2.48, 2.38) | 0.97 | 0.08 (-3.54, 3.69) | 0.97 | 0.15 (-6.84, 7.14) | 0.97 |
| 290.2 | Delirium due to conditions classified elsewhere | 4980 | -0.09 (-4.37, 4.19) | 0.97 | 0.13 (-6.19, 6.45) | 0.97 | 0.83 (-6.38, 8.03) | 0.833 | -0.1 (-4.64, 4.45) | 0.97 | -0.04 (-2.16, 2.07) | 0.97 |
| 290.3 | Other persistent mental disorders due to conditions classified elsewhere | 1282 | 0.04 (-1.72, 1.8) | 0.97 | 0.2 (-9.12, 9.52) | 0.97 | 2.01 (-39.34, 43.37) | 0.93 | -0.44 (-19.75, 18.88) | 0.968 | 0.31 (-14.34, 14.96) | 0.97 |
| 291 | Other specified nonpsychotic and/or transient mental disorders | 1748 | -0.13 (-6.08, 5.82) | 0.97 | 0.39 (-15.74, 16.52) | 0.966 | -1.27 (-17.4, 14.87) | 0.887 | 0.16 (-7.33, 7.64) | 0.97 | -0.19 (-9.18, 8.8) | 0.97 |
| 291.1 | Transient mental disorders due to conditions classified elsewhere | 501 | 0.51 (-20.57, 21.59) | 0.966 | 0.94 (-32.2, 34.07) | 0.96 | -5.11 (-13.19, 2.96) | 0.216 | -0.04 (-1.84, 1.76) | 0.97 | 0.15 (-6.76, 7.05) | 0.97 |
| 291.4 | Specific nonpsychotic mental disorders due to brain damage | 600 | -0.92 (-12.62, 10.78) | 0.887 | 0.27 (-12.53, 13.07) | 0.97 | 1.37 (-55.18, 57.93) | 0.966 | 0.61 (-15.92, 17.14) | 0.948 | -0.39 (-18.84, 18.06) | 0.97 |
| 291.8 | Alteration of consciousness | 674 | 0.38 (-15.27, 16.03) | 0.966 | 0.11 (-5.12, 5.34) | 0.97 | -0.37 (-18.09, 17.35) | 0.97 | -0.37 (-15.44, 14.71) | 0.966 | -0.54 (-22.98, 21.89) | 0.966 |
| 292 | Neurological disorders | 8633 | -0.11 (-4.67, 4.45) | 0.966 | -0.09 (-4.54, 4.36) | 0.97 | -0.39 (-9.17, 8.39) | 0.936 | 0.22 (-2.57, 3.01) | 0.887 | 0.19 (-4.93, 5.31) | 0.948 |
| 292.1 | Aphasia/speech disturbance | 1022 | -0.33 (-14.84, 14.18) | 0.967 | 0.04 (-2.05, 2.13) | 0.97 | -0.43 (-20.94, 20.07) | 0.97 | 0.39 (-15.7, 16.48) | 0.966 | 1.11 (-10.68, 12.9) | 0.864 |
| 292.12 | Symbolic dysfunction | 151 | 0.11 (-4.94, 5.15) | 0.97 | 0.46 (-21.34, 22.26) | 0.97 | -3.99 (-78.51, 70.53) | 0.923 | 0.18 (-8.13, 8.48) | 0.97 | -0.28 (-13.57, 13.01) | 0.97 |
| 292.2 | Mild cognitive impairment | 844 | 0.26 (-10.34, 10.85) | 0.966 | -0.24 (-11.63, 11.15) | 0.97 | 0.29 (-13.37, 13.94) | 0.97 | -0.2 (-9.77, 9.37) | 0.97 | 0.56 (-11.01, 12.14) | 0.93 |
| 292.3 | Memory loss | 3241 | -0.09 (-4.3, 4.12) | 0.97 | 0.08 (-3.83, 4) | 0.97 | -0.37 (-13.52, 12.77) | 0.96 | 0.12 (-4.91, 5.14) | 0.967 | -0.19 (-8.22, 7.83) | 0.966 |
| 292.4 | Altered mental status | 3282 | -0.02 (-0.96, 0.92) | 0.97 | -0.34 (-4.13, 3.45) | 0.871 | -0.38 (-8.94, 8.18) | 0.936 | 0.23 (-2.25, 2.71) | 0.864 | 0.02 (-0.82, 0.86) | 0.97 |
| 292.6 | Hallucinations | 182 | 1.24 (-32.56, 35.04) | 0.948 | 0.49 (-22.76, 23.74) | 0.97 | -2.09 (-101.13, 96.94) | 0.97 | -1.24 (-45.12, 42.64) | 0.96 | 1.63 (-65.45, 68.71) | 0.966 |
| 293 | Symptoms involving head and neck | 5655 | -0.05 (-2.21, 2.12) | 0.97 | 0.09 (-4.38, 4.57) | 0.97 | 0.22 (-10, 10.43) | 0.97 | -0.03 (-1.52, 1.46) | 0.97 | -0.16 (-7.75, 7.43) | 0.97 |
| 293.1 | Swelling, mass, or lump in head and neck [Space-occupying lesion, intracranial NOS] | 943 | -0.2 (-9.72, 9.32) | 0.97 | 0.91 (-17.44, 19.26) | 0.929 | 0.58 (-26.64, 27.79) | 0.97 | -0.3 (-14.67, 14.06) | 0.97 | -0.19 (-9.27, 8.88) | 0.97 |
| 295 | Schizophrenia and other psychotic disorders | 3037 | 0.27 (-11.59, 12.14) | 0.967 | -0.25 (-12.05, 11.55) | 0.97 | -0.65 (-28.04, 26.74) | 0.966 | -0.05 (-2.4, 2.3) | 0.97 | -0.21 (-10.21, 9.79) | 0.97 |
| 295.1 | Schizophrenia | 2220 | -0.07 (-3.3, 3.16) | 0.97 | -0.01 (-0.43, 0.41) | 0.97 | -0.07 (-3.54, 3.4) | 0.97 | 0.08 (-3.88, 4.05) | 0.97 | 0.06 (-2.78, 2.9) | 0.97 |
| 295.2 | Paranoid disorders | 739 | 0.81 (-27.75, 29.37) | 0.96 | 0.25 (-11.35, 11.84) | 0.97 | 0.51 (-23.57, 24.59) | 0.97 | -1.02 (-22.52, 20.49) | 0.932 | 0.38 (-17.67, 18.44) | 0.97 |
| 295.3 | Psychosis | 367 | -0.48 (-23.4, 22.43) | 0.97 | -4.49 (-50.27, 41.29) | 0.858 | -0.36 (-17.43, 16.71) | 0.97 | 2.36 (-27.6, 32.31) | 0.887 | -3.75 (-43.06, 35.57) | 0.862 |
| 296 | Mood disorders | 4033 | 0.02 (-1.02, 1.06) | 0.97 | -0.18 (-8.87, 8.5) | 0.97 | 0.05 (-2.53, 2.64) | 0.97 | 0.05 (-2.18, 2.27) | 0.97 | -0.13 (-6.1, 5.85) | 0.97 |
| 296.1 | Bipolar | 1551 | 0.13 (-6.1, 6.36) | 0.97 | -0.81 (-15.88, 14.27) | 0.923 | -0.92 (-33.37, 31.53) | 0.96 | 0.38 (-13.14, 13.91) | 0.96 | 0.05 (-2.16, 2.26) | 0.97 |
| 296.22 | Major depressive disorder | 2405 | -0.18 (-7.6, 7.24) | 0.966 | 0.37 (-7.36, 8.09) | 0.932 | -0.16 (-7.79, 7.47) | 0.97 | 0.05 (-2.3, 2.4) | 0.97 | -0.24 (-10.17, 9.68) | 0.966 |
| 297 | Suicidal ideation or attempt | 376 | 0.25 (-11.78, 12.29) | 0.97 | 0.63 (-20.47, 21.73) | 0.958 | -1.87 (-18.13, 14.39) | 0.833 | -0.17 (-8.1, 7.77) | 0.97 | 0.58 (-15.13, 16.29) | 0.948 |
| 297.2 | Suicide or self-inflicted injury | 376 | 0.25 (-11.78, 12.29) | 0.97 | 0.63 (-20.47, 21.73) | 0.958 | -1.87 (-18.13, 14.39) | 0.833 | -0.17 (-8.1, 7.77) | 0.97 | 0.58 (-15.13, 16.29) | 0.948 |
| 300 | Anxiety disorders | 12598 | -0.1 (-4.83, 4.63) | 0.97 | 0.41 (-3.96, 4.78) | 0.864 | 0.46 (-9.26, 10.19) | 0.932 | -0.15 (-6.15, 5.86) | 0.966 | 0.5 (-1.98, 2.99) | 0.705 |
| 300.1 | Anxiety disorder | 7985 | -0.06 (-2.89, 2.77) | 0.97 | -0.13 (-5.4, 5.14) | 0.966 | 0.15 (-6.99, 7.29) | 0.97 | 0.09 (-3.5, 3.67) | 0.966 | 0.12 (-4.65, 4.88) | 0.966 |
| 300.11 | Generalized anxiety disorder | 378 | 0.05 (-2.42, 2.53) | 0.97 | -0.96 (-27.07, 25.15) | 0.948 | 0.54 (-25.05, 26.13) | 0.97 | 0.21 (-9.75, 10.17) | 0.97 | -0.29 (-13.86, 13.29) | 0.97 |
| 300.12 | Agorophobia, social phobia, and panic disorder | 882 | 0.07 (-3.1, 3.23) | 0.97 | -0.39 (-18.96, 18.17) | 0.97 | 0.44 (-20.55, 21.44) | 0.97 | 0.01 (-0.34, 0.35) | 0.97 | -0.01 (-0.44, 0.42) | 0.97 |
| 300.13 | Phobia | 278 | -1.4 (-21.65, 18.85) | 0.9 | 2.02 (-39.48, 43.52) | 0.93 | -0.13 (-6.05, 5.8) | 0.97 | 0.7 (-29.88, 31.28) | 0.967 | 1.61 (-34.29, 37.51) | 0.936 |
| 300.3 | Obsessive-compulsive disorders | 382 | -0.49 (-20.81, 19.82) | 0.966 | 0.14 (-6.47, 6.75) | 0.97 | 1.53 (-40.12, 43.18) | 0.948 | 0.14 (-6.66, 6.94) | 0.97 | 0.83 (-30.07, 31.73) | 0.962 |
| 300.4 | Dysthymic disorder | 985 | -1.2 (-25.33, 22.93) | 0.929 | 2.38 (-25.02, 29.79) | 0.874 | -0.06 (-3.1, 2.97) | 0.97 | 0.31 (-14.58, 15.21) | 0.97 | 2.13 (-20.49, 24.75) | 0.864 |
| 300.8 | Acute reaction to stress | 1073 | -0.17 (-8.06, 7.73) | 0.97 | 0.5 (-20.06, 21.06) | 0.966 | 0.44 (-20.45, 21.33) | 0.97 | -0.11 (-5.2, 4.98) | 0.97 | 0.35 (-15.1, 15.81) | 0.968 |
| 300.9 | Posttraumatic stress disorder | 605 | -0.3 (-14.39, 13.79) | 0.97 | 0.67 (-17.65, 19) | 0.948 | -1.85 (-21.54, 17.84) | 0.864 | 0.31 (-13.29, 13.92) | 0.967 | -0.05 (-2.55, 2.45) | 0.97 |
| 301 | Personality disorders | 1696 | -0.55 (-14.72, 13.62) | 0.945 | 1.3 (-12.53, 15.13) | 0.864 | 1.29 (-33.92, 36.51) | 0.948 | -0.16 (-7.87, 7.55) | 0.97 | -0.73 (-18.71, 17.25) | 0.942 |
| 301.2 | Antisocial/borderline personality disorder | 708 | 0.26 (-12.26, 12.79) | 0.97 | -0.25 (-12.24, 11.73) | 0.97 | 0.7 (-30.15, 31.55) | 0.968 | -0.29 (-13.8, 13.22) | 0.97 | -0.47 (-20.01, 19.06) | 0.966 |
| 302 | Sexual and gender identity disorders | 1052 | -0.17 (-8.13, 7.79) | 0.97 | 0.22 (-10.1, 10.53) | 0.97 | -0.41 (-19.66, 18.84) | 0.97 | 0.14 (-6.61, 6.89) | 0.97 | -0.36 (-17.5, 16.78) | 0.97 |
| 302.1 | Decreased libido | 169 | -0.57 (-27.39, 26.25) | 0.97 | -0.09 (-4.53, 4.34) | 0.97 | -0.93 (-44.96, 43.1) | 0.97 | 0.84 (-33.59, 35.26) | 0.966 | -0.97 (-43.68, 41.74) | 0.968 |
| 303 | Psychogenic and somatoform disorders | 2867 | 0.11 (-5.16, 5.38) | 0.97 | 0.32 (-14.77, 15.41) | 0.97 | 0.54 (-24.92, 26) | 0.97 | -0.35 (-15.46, 14.77) | 0.967 | 0.18 (-8.53, 8.89) | 0.97 |
| 303.1 | Dissociative disorder | 522 | -1.8 (-20.94, 17.34) | 0.864 | 1.98 (-50.2, 54.16) | 0.946 | -1.6 (-77.48, 74.27) | 0.97 | 1.26 (-27.11, 29.63) | 0.936 | -1.54 (-43.5, 40.42) | 0.948 |
| 303.3 | Psychogenic disorder | 1150 | 0.65 (-22.93, 24.24) | 0.961 | 0.22 (-10.12, 10.56) | 0.97 | -2.23 (-42.65, 38.19) | 0.921 | -0.33 (-15.73, 15.07) | 0.97 | 0.39 (-18.06, 18.84) | 0.97 |
| 303.4 | Somatoform disorder | 1137 | -0.22 (-10.55, 10.12) | 0.97 | -0.45 (-21.57, 20.67) | 0.97 | 1.25 (-24.98, 27.49) | 0.932 | 0.13 (-5.85, 6.1) | 0.97 | 0.67 (-17.48, 18.81) | 0.948 |
| 304 | Adjustment reaction | 4219 | -0.54 (-7.32, 6.23) | 0.884 | 0.38 (-16.13, 16.89) | 0.967 | 1.53 (-11.8, 14.87) | 0.833 | 0.09 (-4.33, 4.52) | 0.97 | 0.29 (-13.53, 14.11) | 0.97 |
| 305.2 | Eating disorder | 872 | 0.24 (-11.15, 11.63) | 0.97 | 0.45 (-20.95, 21.85) | 0.97 | -0.63 (-30.24, 28.98) | 0.97 | -0.34 (-14.15, 13.48) | 0.966 | 0.47 (-19.01, 19.96) | 0.966 |
| 305.21 | Anorexia nervosa | 589 | 0.55 (-22.17, 23.27) | 0.966 | -0.29 (-13.85, 13.28) | 0.97 | -2.89 (-33.65, 27.87) | 0.864 | 0.04 (-1.9, 1.98) | 0.97 | -0.02 (-0.86, 0.83) | 0.97 |
| 306 | Other mental disorder | 6671 | -0.04 (-2.06, 1.97) | 0.97 | -0.26 (-7.3, 6.78) | 0.948 | 0.4 (-11.4, 12.2) | 0.952 | 0.09 (-4.07, 4.25) | 0.97 | 0.32 (-5.61, 6.25) | 0.923 |
| 306.9 | Tension headache | 6038 | -0.02 (-1.17, 1.12) | 0.97 | -0.25 (-8.05, 7.55) | 0.954 | 0.34 (-13.76, 14.45) | 0.966 | 0.08 (-3.49, 3.64) | 0.97 | 0.3 (-5.9, 6.5) | 0.93 |
| 312 | Conduct disorders | 361 | 0.01 (-0.49, 0.52) | 0.97 | 1.24 (-26.67, 29.16) | 0.936 | 0.03 (-1.3, 1.36) | 0.97 | -0.61 (-25.83, 24.61) | 0.966 | -0.37 (-17.96, 17.21) | 0.97 |
| 313 | Pervasive developmental disorders | 2254 | -0.35 (-14.97, 14.26) | 0.966 | -0.28 (-13.76, 13.19) | 0.97 | 0.33 (-15.08, 15.73) | 0.97 | 0.43 (-11.17, 12.02) | 0.948 | -0.36 (-17.31, 16.6) | 0.97 |
| 313.1 | Attention deficit hyperactivity disorder | 216 | 0.64 (-29.62, 30.9) | 0.97 | -3.25 (-31.54, 25.03) | 0.833 | 0.26 (-11.86, 12.38) | 0.97 | 0.62 (-28.52, 29.75) | 0.97 | -0.06 (-2.67, 2.56) | 0.97 |
| 313.2 | Tics and stuttering | 466 | -0.04 (-1.96, 1.88) | 0.97 | 1.26 (-24.64, 27.17) | 0.93 | 2.6 (-50.82, 56.02) | 0.93 | -0.88 (-18.97, 17.21) | 0.93 | -0.39 (-18.9, 18.11) | 0.97 |
| 313.3 | Autism | 504 | -0.56 (-15.92, 14.8) | 0.948 | -0.42 (-20.19, 19.36) | 0.97 | -0.42 (-20.04, 19.21) | 0.97 | 0.85 (-8.21, 9.91) | 0.864 | -0.13 (-6.28, 6.02) | 0.97 |
| 315 | Develomental delays and disorders | 1951 | -0.23 (-11.14, 10.68) | 0.97 | 1.04 (-12.55, 14.63) | 0.89 | -0.85 (-35.88, 34.18) | 0.966 | -0.08 (-3.82, 3.66) | 0.97 | 0.35 (-16.22, 16.92) | 0.97 |
| 315.2 | Speech and language disorder | 221 | 0.87 (-34.94, 36.67) | 0.966 | -2.19 (-43.16, 38.78) | 0.923 | 0.07 (-3.43, 3.57) | 0.97 | 0.07 (-3.24, 3.38) | 0.97 | 0.41 (-19.1, 19.92) | 0.97 |
| 315.3 | Mental retardation | 1555 | -0.41 (-17.49, 16.67) | 0.966 | 1.3 (-12.52, 15.12) | 0.864 | -0.58 (-27.78, 26.63) | 0.97 | -0.06 (-3.01, 2.89) | 0.97 | 0.28 (-12.89, 13.44) | 0.97 |
| 316 | Substance addiction and disorders | 3523 | 0.28 (-9.46, 10.01) | 0.96 | 0.18 (-8.53, 8.9) | 0.97 | 1.42 (-10.93, 13.77) | 0.833 | -0.62 (-5.61, 4.37) | 0.82 | -0.02 (-0.84, 0.8) | 0.97 |
| 317 | Alcohol-related disorders | 30909 | -0.08 (-3.6, 3.44) | 0.967 | 0.14 (-5.61, 5.89) | 0.966 | -0.05 (-2.2, 2.11) | 0.97 | 0.03 (-1.53, 1.59) | 0.97 | 0.12 (-5, 5.25) | 0.966 |
| 317.1 | Alcoholism | 6553 | 0.17 (-4.57, 4.91) | 0.948 | 0.02 (-0.9, 0.94) | 0.97 | -0.36 (-15.27, 14.55) | 0.966 | -0.13 (-5.34, 5.08) | 0.966 | 0.64 (-1.03, 2.31) | 0.46 |
| 317.11 | Alcoholic liver damage | 4796 | -0.18 (-7.71, 7.34) | 0.966 | -0.67 (-7.85, 6.5) | 0.864 | 0.71 (-13.8, 15.22) | 0.93 | 0.33 (-5.07, 5.72) | 0.913 | -0.05 (-2.65, 2.54) | 0.97 |
| 318 | Tobacco use disorder | 19440 | 0.04 (-1.67, 1.74) | 0.97 | 0 (-0.02, 0.02) | 0.97 | -0.16 (-4.4, 4.09) | 0.948 | -0.01 (-0.46, 0.44) | 0.97 | 0.14 (-1.31, 1.58) | 0.864 |
| 320 | Meningitis | 3235 | -0.11 (-5.34, 5.12) | 0.97 | -0.79 (-22.2, 20.63) | 0.948 | -0.5 (-24.1, 23.1) | 0.97 | 0.53 (-11.62, 12.68) | 0.938 | -0.12 (-5.7, 5.46) | 0.97 |
| 323 | Encephalitis | 1134 | 0.16 (-7.61, 7.94) | 0.97 | -0.86 (-38.6, 36.87) | 0.967 | -1.21 (-56.43, 54.02) | 0.969 | 0.38 (-17.75, 18.52) | 0.97 | -1.83 (-21.25, 17.6) | 0.864 |
| 323.2 | Acute (transverse) myelitis | 141 | -0.11 (-5.1, 4.89) | 0.97 | -2.06 (-58.23, 54.1) | 0.948 | -1.61 (-77.86, 74.63) | 0.97 | 1.06 (-36.18, 38.29) | 0.96 | 0.94 (-43.1, 44.99) | 0.97 |
| 323.8 | Encephalitis, non-infectious | 141 | 2.01 (-74.51, 78.52) | 0.963 | -3.76 (-88.07, 80.55) | 0.936 | 0.08 (-3.5, 3.66) | 0.97 | -0.33 (-16.09, 15.43) | 0.97 | -4.21 (-65.27, 56.85) | 0.901 |
| 324 | Other CNS infection and poliomyelitis | 572 | -0.22 (-10.63, 10.19) | 0.97 | 0.7 (-32.61, 34.02) | 0.97 | -1.75 (-49.44, 45.94) | 0.948 | 0.23 (-10.68, 11.14) | 0.97 | 0.26 (-11.85, 12.36) | 0.97 |
| 327 | Sleep disorders | 14396 | -0.14 (-2.81, 2.53) | 0.923 | 0.24 (-2.83, 3.31) | 0.887 | 0.04 (-2.06, 2.15) | 0.97 | 0.04 (-1.8, 1.88) | 0.97 | 0.2 (-2.88, 3.28) | 0.907 |
| 327.3 | Sleep apnea | 10414 | -0.25 (-2.24, 1.75) | 0.82 | 0.13 (-5.61, 5.88) | 0.967 | 0.17 (-7.89, 8.23) | 0.97 | 0.17 (-2.23, 2.56) | 0.9 | 0.12 (-5, 5.25) | 0.966 |
| 327.4 | Insomnia | 282 | 0.33 (-15.5, 16.17) | 0.97 | -3.39 (-12.15, 5.37) | 0.457 | 2.12 (-40.5, 44.74) | 0.929 | 0.56 (-22.43, 23.54) | 0.966 | -1.17 (-27.79, 25.44) | 0.937 |
| 327.41 | Organic or persistent insomnia | 216 | -0.15 (-7.2, 6.9) | 0.97 | 2.44 (-14.56, 19.44) | 0.791 | 1.37 (-54.92, 57.65) | 0.966 | -1.36 (-15.86, 13.13) | 0.864 | -0.5 (-23.98, 22.99) | 0.97 |
| 327.6 | Circadian rhythm sleep disorder | 254 | -0.23 (-11.29, 10.82) | 0.97 | -0.31 (-15.2, 14.57) | 0.97 | -0.22 (-10.68, 10.24) | 0.97 | 0.38 (-17.81, 18.58) | 0.97 | 1.15 (-23.01, 25.32) | 0.932 |
| 331 | Other cerebral degenerations | 4477 | -0.02 (-1.02, 0.98) | 0.97 | 0.18 (-8.51, 8.88) | 0.97 | -0.43 (-18.01, 17.15) | 0.966 | 0.01 (-0.67, 0.69) | 0.97 | 0.19 (-8.03, 8.41) | 0.967 |
| 331.1 | Hydrocephalus | 2752 | 0.12 (-5.58, 5.82) | 0.97 | 0.19 (-8.64, 9.02) | 0.97 | -0.06 (-2.79, 2.67) | 0.97 | -0.19 (-8.27, 7.9) | 0.967 | 0 (-0.22, 0.21) | 0.97 |
| 331.9 | Cerebral degeneration, unspecified | 723 | -0.24 (-11.64, 11.16) | 0.97 | 0.39 (-18, 18.78) | 0.97 | -1.26 (-27.68, 25.16) | 0.932 | 0.29 (-12.49, 13.07) | 0.968 | 0.64 (-16.67, 17.94) | 0.948 |
| 333 | Extrapyramidal disease and abnormal movement disorders | 2998 | -0.31 (-6.71, 6.09) | 0.93 | 0.2 (-9.13, 9.52) | 0.97 | 0.25 (-11.36, 11.85) | 0.97 | 0.19 (-7.45, 7.82) | 0.966 | -0.49 (-6.76, 5.77) | 0.887 |
| 333.1 | Essential tremor | 698 | -0.55 (-12.3, 11.19) | 0.932 | -0.44 (-21.09, 20.22) | 0.97 | 0.68 (-31.6, 32.96) | 0.97 | 0.62 (-11.88, 13.12) | 0.929 | 0 (-0.17, 0.18) | 0.97 |
| 333.4 | Torsion dystonia | 1482 | -0.4 (-11.15, 10.36) | 0.948 | 0.41 (-17.6, 18.43) | 0.967 | 0.42 (-19.57, 20.41) | 0.97 | 0.15 (-7.01, 7.32) | 0.97 | -0.71 (-9.93, 8.52) | 0.89 |
| 333.8 | Other degenerative diseases of the basal ganglia | 381 | 0.29 (-13.35, 13.92) | 0.97 | -0.51 (-22.55, 21.54) | 0.967 | -2.26 (-26.33, 21.81) | 0.864 | 0.19 (-8.68, 9.05) | 0.97 | 0.43 (-18.33, 19.19) | 0.967 |
| 334 | Degenerative disease of the spinal cord | 5503 | -0.03 (-1.44, 1.38) | 0.97 | -0.11 (-5.22, 5.01) | 0.97 | -0.41 (-14.94, 14.12) | 0.96 | 0.15 (-6.18, 6.48) | 0.966 | 0.23 (-8.05, 8.51) | 0.96 |
| 334.2 | Anterior horn cell disease | 543 | 0.61 (-24.56, 25.78) | 0.966 | 0.25 (-11.58, 12.08) | 0.97 | -2.74 (-31.83, 26.36) | 0.864 | -0.13 (-6.45, 6.18) | 0.97 | -0.74 (-31.41, 29.93) | 0.966 |
| 335 | Multiple sclerosis | 138 | -0.09 (-4.35, 4.17) | 0.97 | -0.3 (-14.45, 13.85) | 0.97 | -2.45 (-103.34, 98.44) | 0.966 | 0.92 (-42.42, 44.26) | 0.97 | -3.65 (-35.39, 28.09) | 0.833 |
| 337 | Disorders of the autonomic nervous system | 1032 | 0.32 (-13.84, 14.48) | 0.968 | -0.29 (-14.19, 13.6) | 0.97 | 0.25 (-11.45, 11.94) | 0.97 | -0.23 (-10.95, 10.49) | 0.97 | -0.36 (-17.44, 16.71) | 0.97 |
| 337.1 | Peripheral autonomic neuropathy | 548 | 0.21 (-9.78, 10.21) | 0.97 | 0.23 (-10.65, 11.11) | 0.97 | -3.44 (-31.16, 24.28) | 0.82 | 0.03 (-1.57, 1.64) | 0.97 | 0.4 (-18.72, 19.53) | 0.97 |
| 338 | Pain | 34421 | -0.02 (-1.06, 1.02) | 0.97 | -0.09 (-2.41, 2.24) | 0.948 | 0.01 (-0.52, 0.54) | 0.97 | 0.06 (-1.5, 1.61) | 0.948 | -0.04 (-2.07, 1.99) | 0.97 |
| 338.1 | Acute pain | 25349 | 0 (-0.01, 0.01) | 0.97 | -0.05 (-2.33, 2.23) | 0.97 | 0.08 (-3.88, 4.04) | 0.97 | 0.01 (-0.29, 0.31) | 0.97 | -0.13 (-1.73, 1.48) | 0.887 |
| 338.2 | Chronic pain | 11033 | -0.06 (-3.03, 2.9) | 0.97 | -0.21 (-3.71, 3.29) | 0.915 | -0.03 (-1.62, 1.56) | 0.97 | 0.16 (-1.52, 1.84) | 0.864 | 0.17 (-3.23, 3.56) | 0.93 |
| 339 | Other headache syndromes | 3466 | 0.23 (-4.72, 5.19) | 0.932 | -0.24 (-10.18, 9.69) | 0.966 | 0.36 (-14.62, 15.34) | 0.966 | -0.2 (-5.56, 5.16) | 0.948 | 0.11 (-5.02, 5.23) | 0.97 |
| 340 | Migraine | 12365 | -0.25 (-4.95, 4.45) | 0.923 | 0.7 (-2.47, 3.86) | 0.68 | -0.23 (-11.13, 10.67) | 0.97 | -0.01 (-0.45, 0.44) | 0.97 | 0.04 (-1.88, 1.96) | 0.97 |
| 340.1 | Migrain with aura | 3450 | -0.28 (-6.06, 5.5) | 0.93 | -0.2 (-9.86, 9.45) | 0.97 | -0.27 (-13.17, 12.62) | 0.97 | 0.44 (-3.36, 4.23) | 0.833 | 0.15 (-6.91, 7.21) | 0.97 |
| 341 | Other demyelinating diseases of central nervous system | 709 | -0.06 (-2.91, 2.79) | 0.97 | -0.98 (-27.56, 25.61) | 0.948 | 1.41 (-52.56, 55.39) | 0.963 | 0.24 (-11.24, 11.73) | 0.97 | 0.79 (-27.33, 28.92) | 0.96 |
| 342 | Hemiplegia | 676 | 0.22 (-10.31, 10.75) | 0.97 | -1.4 (-15.73, 12.92) | 0.858 | -0.16 (-7.77, 7.45) | 0.97 | 0.46 (-18.31, 19.22) | 0.966 | -1.02 (-13.12, 11.07) | 0.878 |
| 343 | Infantile cerebral palsy | 1544 | 0.75 (-13.27, 14.77) | 0.923 | 0.21 (-9.93, 10.36) | 0.97 | 0.79 (-36.55, 38.13) | 0.97 | -0.97 (-11.28, 9.34) | 0.864 | 0.47 (-21.73, 22.66) | 0.97 |
| 344 | Other paralytic syndromes | 2595 | -0.2 (-8.49, 8.09) | 0.966 | 0.07 (-3.21, 3.35) | 0.97 | 0.22 (-10.31, 10.76) | 0.97 | 0.14 (-6.43, 6.71) | 0.97 | -0.22 (-10.85, 10.4) | 0.97 |
| 345 | Epilepsy, recurrent seizures, convulsions | 23558 | 0.2 (-3.43, 3.84) | 0.919 | 0.44 (-3.41, 4.3) | 0.833 | 0.06 (-2.83, 2.95) | 0.97 | -0.4 (-1.71, 0.9) | 0.556 | 0.49 (-1.6, 2.59) | 0.658 |
| 345.1 | Epilepsy | 2693 | -0.02 (-1.11, 1.07) | 0.97 | 0.03 (-1.37, 1.43) | 0.97 | 0.25 (-11.71, 12.21) | 0.97 | -0.03 (-1.48, 1.42) | 0.97 | 0.65 (-6.62, 7.92) | 0.871 |
| 345.11 | Generalized convulsive epilepsy | 2428 | 0 (-0.12, 0.12) | 0.97 | 0.86 (-11.55, 13.26) | 0.901 | 0.1 (-4.46, 4.66) | 0.97 | -0.4 (-11.42, 10.61) | 0.948 | 0.45 (-18.01, 18.9) | 0.966 |
| 345.12 | Partial epilepsy | 4915 | 0.05 (-2.52, 2.63) | 0.97 | 0.26 (-12.04, 12.56) | 0.97 | -0.25 (-11.92, 11.42) | 0.97 | -0.11 (-5.53, 5.3) | 0.97 | 0.94 (-2.77, 4.64) | 0.634 |
| 345.3 | Convulsions | 14466 | 0.19 (-5.01, 5.39) | 0.948 | 0.36 (-6.8, 7.51) | 0.929 | 0.02 (-0.88, 0.91) | 0.97 | -0.35 (-3.38, 2.68) | 0.833 | 0.3 (-5.87, 6.47) | 0.93 |
| 346 | Abnormal findings on study of brain and/or nervous system | 744 | -0.72 (-8.34, 6.91) | 0.864 | 0.24 (-11.1, 11.58) | 0.97 | -0.46 (-22.19, 21.27) | 0.97 | 0.69 (-6.68, 8.07) | 0.864 | 0.31 (-14.41, 15.03) | 0.97 |
| 346.2 | Nonspecific abnormal results of function study of brain and central nervous system | 639 | -0.56 (-11.91, 10.78) | 0.929 | 0.04 (-1.97, 2.06) | 0.97 | -1.01 (-39.49, 37.48) | 0.963 | 0.71 (-6.8, 8.21) | 0.864 | 0.47 (-18.98, 19.92) | 0.966 |
| 347 | Cataplexy and narcolepsy | 314 | -1.71 (-19.87, 16.45) | 0.864 | -0.18 (-8.91, 8.54) | 0.97 | -0.57 (-27.5, 26.36) | 0.97 | 1.82 (-17.58, 21.23) | 0.864 | 0.97 (-43.6, 45.54) | 0.969 |
| 348 | Other conditions of brain | 3294 | 0.1 (-4.82, 5.03) | 0.97 | -0.64 (-13.83, 12.55) | 0.93 | 0.21 (-9.51, 9.92) | 0.97 | 0.14 (-6.26, 6.53) | 0.97 | 0.49 (-12.92, 13.9) | 0.948 |
| 348.2 | Cerebral edema and compression of brain | 644 | 0.35 (-14.14, 14.85) | 0.966 | -0.73 (-15.64, 14.19) | 0.93 | -0.42 (-20.27, 19.43) | 0.97 | 0.04 (-2.03, 2.12) | 0.97 | 0.47 (-18.99, 19.94) | 0.966 |
| 348.4 | Cerebral cysts | 468 | 0.15 (-6.73, 7.02) | 0.97 | -0.83 (-23.46, 21.8) | 0.948 | 0.95 (-38.39, 40.3) | 0.966 | 0.01 (-0.27, 0.28) | 0.97 | 0.45 (-20.94, 21.85) | 0.97 |
| 348.7 | Coma | 776 | 0.13 (-5.85, 6.11) | 0.97 | -0.98 (-9.59, 7.64) | 0.835 | 0.64 (-25.74, 27.02) | 0.966 | 0.15 (-7.08, 7.38) | 0.97 | 0.12 (-5.33, 5.56) | 0.97 |
| 348.8 | Encephalopathy, not elsewhere classified | 486 | 0.64 (-29.51, 30.79) | 0.97 | -0.49 (-23.51, 22.54) | 0.97 | -8.04 (-47.75, 31.67) | 0.705 | 0.23 (-10.76, 11.22) | 0.97 | 2.06 (-24.05, 28.18) | 0.886 |
| 348.9 | Other conditions of brain, NOS | 610 | -1.21 (-25.51, 23.09) | 0.929 | -0.09 (-4.24, 4.07) | 0.97 | -1.34 (-64.66, 61.98) | 0.97 | 1.55 (-14.97, 18.08) | 0.864 | 2.33 (-22.45, 27.11) | 0.864 |
| 349 | Other and unspecified disorders of the nervous system | 10929 | 0.02 (-0.94, 0.98) | 0.97 | 0.08 (-3.62, 3.78) | 0.97 | -0.06 (-3.12, 2.99) | 0.97 | -0.04 (-2.04, 1.95) | 0.97 | 0.1 (-2.64, 2.84) | 0.948 |
| 350 | Abnormal movement | 7571 | 0.06 (-2.84, 2.96) | 0.97 | -0.38 (-8.21, 7.45) | 0.93 | 0.01 (-0.44, 0.46) | 0.97 | 0.1 (-4.46, 4.65) | 0.97 | -0.03 (-1.53, 1.46) | 0.97 |
| 350.1 | Abnormal involuntary movements | 5779 | 0.18 (-7.22, 7.58) | 0.966 | -0.18 (-8.62, 8.27) | 0.97 | -0.58 (-16.28, 15.13) | 0.948 | -0.01 (-0.43, 0.41) | 0.97 | 0 (-0.23, 0.24) | 0.97 |
| 350.2 | Abnormality of gait | 1473 | -0.02 (-0.92, 0.88) | 0.97 | -1.25 (-11.34, 8.84) | 0.82 | 0.91 (-23.93, 25.76) | 0.948 | 0.41 (-10.69, 11.5) | 0.948 | 0.07 (-3.47, 3.62) | 0.97 |
| 350.3 | Lack of coordination | 252 | 1.28 (-33.69, 36.26) | 0.948 | -1.75 (-73.81, 70.31) | 0.966 | -0.19 (-9.03, 8.66) | 0.97 | -0.58 (-27.94, 26.78) | 0.97 | -3.32 (-32.23, 25.58) | 0.833 |
| 350.6 | Disturbances of sensation of smell and taste | 164 | -0.37 (-18.04, 17.29) | 0.97 | -1.46 (-61.79, 58.86) | 0.966 | -0.6 (-29.18, 27.97) | 0.97 | 1.23 (-42.12, 44.57) | 0.96 | -1.15 (-55.3, 53.01) | 0.97 |
| 351 | Other peripheral nerve disorders | 18969 | 0.05 (-2.32, 2.42) | 0.97 | -0.08 (-3.93, 3.76) | 0.97 | -0.02 (-0.84, 0.8) | 0.97 | -0.01 (-0.71, 0.68) | 0.97 | 0 (-0.11, 0.11) | 0.97 |
| 352 | Disorders of other cranial nerves | 5544 | -0.06 (-2.8, 2.68) | 0.97 | 0.57 (-6.91, 8.06) | 0.89 | -0.64 (-18.03, 16.76) | 0.948 | -0.07 (-3.5, 3.35) | 0.97 | 0.52 (-6.3, 7.35) | 0.89 |
| 352.1 | Trigeminal nerve disorders [CN5] | 1858 | 0.71 (-6.89, 8.32) | 0.864 | 0.31 (-14.13, 14.74) | 0.97 | -2.66 (-13.2, 7.88) | 0.634 | -0.37 (-15.63, 14.89) | 0.966 | 0.89 (-10.45, 12.23) | 0.887 |
| 352.2 | Facial nerve disorders [CN7] | 3520 | -0.43 (-8.43, 7.58) | 0.923 | 0.69 (-9.26, 10.65) | 0.9 | 0.49 (-22.5, 23.47) | 0.97 | 0.04 (-2.01, 2.09) | 0.97 | 0.45 (-11.84, 12.74) | 0.948 |
| 353 | Nerve root and plexus disorders | 2124 | 0.23 (-9.4, 9.87) | 0.966 | 0.47 (-12.22, 13.16) | 0.948 | -1.15 (-13.38, 11.08) | 0.864 | -0.21 (-9.49, 9.07) | 0.967 | 0.14 (-6.46, 6.74) | 0.97 |
| 353.1 | Nerve plexus lesions | 876 | 0.29 (-13.26, 13.83) | 0.97 | -0.09 (-4.11, 3.94) | 0.97 | -0.47 (-22.79, 21.84) | 0.97 | -0.18 (-8.58, 8.22) | 0.97 | 0.57 (-22.91, 24.05) | 0.966 |
| 353.2 | Nerve root lesions | 729 | 0.17 (-7.86, 8.19) | 0.97 | 0.6 (-15.82, 17.02) | 0.948 | -1.67 (-19.47, 16.12) | 0.864 | -0.14 (-6.65, 6.37) | 0.97 | -0.14 (-6.8, 6.52) | 0.97 |
| 355 | Complex regional/central pain syndrome | 182 | 0.15 (-6.99, 7.29) | 0.97 | 1.37 (-36.02, 38.77) | 0.948 | -3.73 (-42.85, 35.4) | 0.862 | -0.05 (-2.17, 2.08) | 0.97 | 0.8 (-32.18, 33.78) | 0.966 |
| 357 | Inflammatory and toxic neuropathy | 5784 | -0.27 (-5.61, 5.08) | 0.929 | 0.5 (-5.86, 6.86) | 0.887 | -0.33 (-14.67, 14.02) | 0.968 | 0.14 (-5.98, 6.26) | 0.968 | -0.34 (-7.41, 6.72) | 0.93 |
| 358 | Myoneural disorders | 609 | -0.33 (-16.17, 15.5) | 0.97 | 0.83 (-35.31, 36.96) | 0.967 | 2.4 (-46.82, 51.61) | 0.93 | -0.43 (-20.84, 19.98) | 0.97 | 0.6 (-27.87, 29.08) | 0.97 |
| 358.1 | Myasthenia gravis | 307 | 0.1 (-4.86, 5.07) | 0.97 | 0.47 (-21.64, 22.57) | 0.97 | 5.01 (-38.61, 48.64) | 0.833 | -1.46 (-34.19, 31.28) | 0.936 | 2.73 (-32, 37.47) | 0.887 |
| 359 | Muscular dystrophies and other myopathies | 2471 | -0.02 (-1.15, 1.11) | 0.97 | 0.58 (-15.08, 16.24) | 0.948 | -0.01 (-0.41, 0.39) | 0.97 | -0.22 (-10.57, 10.13) | 0.97 | 0.35 (-13.89, 14.59) | 0.966 |
| 359.1 | Muscular dystrophies | 375 | -0.58 (-27.93, 26.78) | 0.97 | 2.72 (-31.83, 37.27) | 0.887 | 2.24 (-90.23, 94.72) | 0.966 | -0.92 (-39.04, 37.19) | 0.966 | 0 (-0.06, 0.06) | 0.97 |
| 359.2 | Myopathy | 2208 | 0.15 (-6.96, 7.27) | 0.97 | 0.13 (-5.81, 6.06) | 0.97 | -0.67 (-28.28, 26.94) | 0.966 | -0.09 (-4.17, 4) | 0.97 | 0.49 (-12.8, 13.78) | 0.948 |
| 360 | Disorders of the globe | 943 | -0.53 (-12.72, 11.66) | 0.938 | 0.24 (-11.04, 11.52) | 0.97 | 0.49 (-22.84, 23.83) | 0.97 | 0.35 (-14.26, 14.96) | 0.966 | -0.23 (-11.24, 10.77) | 0.97 |
| 360.2 | Progressive myopia | 563 | -1.07 (-9.69, 7.55) | 0.82 | 0.42 (-19.57, 20.42) | 0.97 | -0.67 (-32.13, 30.8) | 0.97 | 1.02 (-7.84, 9.87) | 0.833 | -0.71 (-19.94, 18.53) | 0.948 |
| 361 | Retinal detachments and defects | 5399 | -0.18 (-7.8, 7.43) | 0.966 | 0.46 (-9.01, 9.93) | 0.931 | 0.36 (-16.74, 17.47) | 0.97 | -0.07 (-3.46, 3.32) | 0.97 | -0.24 (-10.57, 10.1) | 0.967 |
| 361.1 | Retinal detachment with retinal defect | 3037 | -0.16 (-7.75, 7.43) | 0.97 | 0.4 (-17.22, 18.03) | 0.967 | 0.98 (-19.12, 21.08) | 0.93 | -0.22 (-10.56, 10.12) | 0.97 | -0.53 (-15.02, 13.96) | 0.948 |
| 361.2 | Retinoschisis and retinal cysts | 395 | 0.25 (-11.44, 11.94) | 0.97 | 0.49 (-22.65, 23.63) | 0.97 | 0.87 (-40.46, 42.21) | 0.97 | -0.59 (-28.47, 27.29) | 0.97 | -0.44 (-21.37, 20.49) | 0.97 |
| 362 | Other retinal disorders | 19034 | 0.05 (-2.29, 2.39) | 0.97 | -0.1 (-4.63, 4.44) | 0.97 | -0.31 (-6.08, 5.46) | 0.923 | 0.04 (-2.08, 2.17) | 0.97 | 0.06 (-2.73, 2.85) | 0.97 |
| 362.1 | Retinopathy of prematurity | 370 | 0.08 (-3.93, 4.1) | 0.97 | -0.3 (-14.38, 13.79) | 0.97 | 0.03 (-1.29, 1.34) | 0.97 | 0.07 (-3.31, 3.46) | 0.97 | -1.66 (-8.22, 4.91) | 0.634 |
| 362.29 | Macular degeneration (senile) of retina NOS | 11754 | -0.07 (-3.2, 3.07) | 0.97 | 0.06 (-2.97, 3.09) | 0.97 | 0.19 (-8.18, 8.57) | 0.967 | 0.01 (-0.43, 0.45) | 0.97 | 0.05 (-2.14, 2.24) | 0.97 |
| 362.3 | Other nondiabetic retinopathy | 1697 | -0.02 (-1.04, 0.99) | 0.97 | 0.37 (-9.72, 10.47) | 0.948 | 0.01 (-0.38, 0.4) | 0.97 | -0.12 (-5.81, 5.56) | 0.97 | 0.16 (-7.21, 7.52) | 0.97 |
| 362.31 | Separation of retinal layers | 571 | -0.09 (-4.23, 4.06) | 0.97 | 0.33 (-15.25, 15.91) | 0.97 | -2.69 (-22.45, 17.07) | 0.802 | 0.43 (-17.12, 17.97) | 0.966 | 0.7 (-18.28, 19.68) | 0.948 |
| 362.4 | Retinal vascular changes and abnomalities | 5501 | 0.15 (-6.19, 6.48) | 0.967 | -0.36 (-10.1, 9.38) | 0.948 | -0.68 (-9.33, 7.97) | 0.887 | 0.13 (-5.87, 6.13) | 0.97 | 0.27 (-7.18, 7.73) | 0.948 |
| 362.6 | Peripheral retinal degenerations | 165 | 0.78 (-33.06, 34.62) | 0.967 | -1.07 (-51.74, 49.59) | 0.97 | 1.04 (-48.23, 50.31) | 0.97 | -0.63 (-30.45, 29.18) | 0.97 | -1.09 (-48.67, 46.49) | 0.967 |
| 362.8 | Retinal hemorrhage/ischemia | 265 | -0.02 (-1.13, 1.08) | 0.97 | -1.64 (-46.24, 42.96) | 0.948 | -1 (-48.17, 46.17) | 0.97 | 1.09 (-37.49, 39.68) | 0.96 | -0.45 (-21.62, 20.72) | 0.97 |
| 363 | Chorioretinal inflammations, scars, and other disorders of choroid | 355 | -1.33 (-15.5, 12.84) | 0.864 | 0.64 (-29.79, 31.08) | 0.97 | 2.27 (-40.25, 44.8) | 0.923 | 0.6 (-24.61, 25.81) | 0.966 | -0.62 (-29.91, 28.67) | 0.97 |
| 364 | Corneal opacity and other disorders of cornea | 2391 | -0.12 (-5.94, 5.7) | 0.97 | 0.14 (-6.39, 6.67) | 0.97 | 1 (-19.04, 21.03) | 0.929 | -0.11 (-5.21, 4.99) | 0.97 | 0.66 (-7.75, 9.08) | 0.887 |
| 364.1 | Corneal opacity | 380 | -0.8 (-33.83, 32.23) | 0.966 | 0.19 (-8.67, 9.04) | 0.97 | 2.6 (-68.14, 73.34) | 0.948 | 0.32 (-14.75, 15.39) | 0.97 | 1.46 (-38.2, 41.11) | 0.948 |
| 364.2 | Corneal edema | 168 | -0.42 (-20.16, 19.32) | 0.97 | -0.1 (-4.95, 4.74) | 0.97 | -1.73 (-83.64, 80.18) | 0.97 | 0.82 (-34.77, 36.4) | 0.967 | 1.45 (-50.83, 53.73) | 0.961 |
| 364.4 | Corneal degenerations | 259 | 0.09 (-4.06, 4.24) | 0.97 | -0.8 (-38.87, 37.26) | 0.97 | 2.06 (-87.91, 92.03) | 0.967 | 0.06 (-2.79, 2.92) | 0.97 | 0.49 (-22.51, 23.49) | 0.97 |
| 364.41 | Keratoconus | 321 | 0.23 (-10.72, 11.18) | 0.97 | 0.61 (-28.09, 29.3) | 0.97 | -0.65 (-31.25, 29.96) | 0.97 | -0.42 (-20.06, 19.23) | 0.97 | 0.49 (-22.68, 23.66) | 0.97 |
| 364.9 | Cornea replaced by transplant | 197 | 1.27 (-33.37, 35.92) | 0.948 | -0.08 (-3.74, 3.58) | 0.97 | 0.04 (-1.7, 1.77) | 0.97 | -1.33 (-35.9, 33.23) | 0.945 | 3.12 (-24.03, 30.28) | 0.833 |
| 365 | Glaucoma | 5441 | -0.07 (-3.45, 3.31) | 0.97 | 0.63 (-6.08, 7.34) | 0.864 | -0.05 (-2.38, 2.28) | 0.97 | -0.19 (-7.95, 7.57) | 0.966 | -0.18 (-8.86, 8.49) | 0.97 |
| 365.11 | Primary open angle glaucoma | 2608 | -0.27 (-11.35, 10.81) | 0.966 | 0.45 (-16.43, 17.34) | 0.962 | -0.18 (-8.76, 8.4) | 0.97 | 0.11 (-5.11, 5.34) | 0.97 | -0.36 (-15.11, 14.4) | 0.966 |
| 365.2 | Primary angle-closure glaucoma | 1207 | 0.73 (-8.54, 10) | 0.887 | 0.69 (-27.83, 29.21) | 0.966 | -2.23 (-21.62, 17.17) | 0.833 | -0.58 (-13.49, 12.33) | 0.936 | -0.29 (-13.93, 13.35) | 0.97 |
| 366 | Cataract | 56831 | -0.02 (-1.18, 1.14) | 0.97 | -0.15 (-1.76, 1.46) | 0.864 | -0.04 (-1.78, 1.71) | 0.97 | 0.09 (-0.88, 1.07) | 0.864 | 0.09 (-1.79, 1.98) | 0.929 |
| 366.1 | Nonsenile Cataract | 2970 | 0.15 (-7.14, 7.45) | 0.97 | -0.11 (-5.28, 5.06) | 0.97 | -0.14 (-6.53, 6.26) | 0.97 | -0.09 (-4.16, 3.99) | 0.97 | 0.73 (-6.75, 8.22) | 0.858 |
| 366.2 | Senile cataract | 52310 | -0.02 (-0.81, 0.78) | 0.97 | -0.21 (-1.04, 0.62) | 0.634 | -0.06 (-2.85, 2.73) | 0.97 | 0.11 (-0.78, 1) | 0.82 | 0.04 (-1.63, 1.7) | 0.97 |
| 366.3 | Traumatic cataract | 196 | 1.8 (-39.09, 42.69) | 0.937 | 0.04 (-1.77, 1.84) | 0.97 | 5.78 (-112.85, 124.41) | 0.93 | -2.56 (-31.05, 25.94) | 0.87 | 1.41 (-64.46, 67.28) | 0.97 |
| 367 | Disorders of refraction and accommodation; blindness and low vision | 11954 | -0.27 (-3.12, 2.57) | 0.862 | 0.31 (-4.34, 4.96) | 0.904 | -0.42 (-9.16, 8.32) | 0.932 | 0.21 (-2.83, 3.25) | 0.9 | 0.14 (-6.02, 6.3) | 0.967 |
| 367.1 | Myopia | 5639 | -0.31 (-3.62, 2.99) | 0.864 | 0.37 (-6.56, 7.3) | 0.923 | 0.05 (-2.13, 2.22) | 0.97 | 0.13 (-5.69, 5.96) | 0.967 | 0.04 (-1.72, 1.79) | 0.97 |
| 367.2 | Astigmatism | 1991 | -0.58 (-6.81, 5.64) | 0.864 | -0.39 (-16.64, 15.85) | 0.966 | 0.54 (-22.94, 24.02) | 0.967 | 0.67 (-5.43, 6.77) | 0.84 | 0.03 (-1.31, 1.37) | 0.97 |
| 367.4 | Presbyopia | 941 | 0.35 (-14.18, 14.89) | 0.966 | -0.17 (-8.23, 7.89) | 0.97 | -0.68 (-28.87, 27.5) | 0.966 | -0.14 (-7, 6.71) | 0.97 | 0.89 (-8.67, 10.45) | 0.865 |
| 367.8 | Hypermetropia | 2452 | 0.41 (-8.08, 8.9) | 0.931 | 0.31 (-14.16, 14.77) | 0.97 | -1.82 (-10.1, 6.46) | 0.68 | -0.18 (-8.81, 8.44) | 0.97 | -0.09 (-4.25, 4.07) | 0.97 |
| 367.9 | Blindness and low vision | 2862 | -0.38 (-5.19, 4.43) | 0.887 | 0 (-0.06, 0.05) | 0.97 | 0.57 (-23.07, 24.22) | 0.966 | 0.29 (-6.28, 6.86) | 0.936 | 0.59 (-5.67, 6.85) | 0.864 |
| 368 | Visual disturbances | 6403 | -0.23 (-6.61, 6.14) | 0.948 | -0.16 (-7.63, 7.31) | 0.97 | 0.93 (-8.17, 10.03) | 0.852 | 0.13 (-5.91, 6.16) | 0.97 | -0.23 (-9.73, 9.27) | 0.966 |
| 368.1 | Amblyopia | 992 | 0.05 (-2.45, 2.55) | 0.97 | -0.23 (-11.21, 10.74) | 0.97 | -0.13 (-6.09, 5.83) | 0.97 | 0.07 (-3.25, 3.39) | 0.97 | -0.83 (-12.82, 11.15) | 0.9 |
| 368.2 | Diplopia and disorders of binocular vision | 1164 | -0.32 (-13.56, 12.92) | 0.966 | 0.44 (-20.05, 20.93) | 0.97 | -0.57 (-27.63, 26.49) | 0.97 | 0.25 (-11.43, 11.93) | 0.97 | -0.04 (-1.73, 1.66) | 0.97 |
| 368.3 | Anisometropia | 482 | 0.03 (-1.32, 1.37) | 0.97 | -0.76 (-34.11, 32.58) | 0.967 | -1.74 (-49.18, 45.7) | 0.948 | 0.71 (-24.57, 26) | 0.96 | 0.09 (-4.16, 4.34) | 0.97 |
| 368.4 | Visual field defects | 1658 | 0.03 (-1.31, 1.37) | 0.97 | -0.05 (-2.38, 2.29) | 0.97 | 1.34 (-12.94, 15.62) | 0.864 | -0.31 (-13, 12.38) | 0.966 | 0.25 (-11.72, 12.22) | 0.97 |
| 368.9 | Subjective visual disturbances | 449 | -0.13 (-6.14, 5.89) | 0.97 | 0.67 (-30.78, 32.11) | 0.97 | 0.3 (-13.99, 14.59) | 0.97 | -0.22 (-10.72, 10.28) | 0.97 | 0.65 (-29.92, 31.23) | 0.97 |
| 369 | Infection of the eye | 19110 | -0.03 (-1.55, 1.49) | 0.97 | 0.08 (-3.84, 4) | 0.97 | 0.32 (-8.41, 9.05) | 0.948 | -0.06 (-2.95, 2.83) | 0.97 | -0.03 (-1.43, 1.37) | 0.97 |
| 369.2 | Eye infection, viral | 1414 | 0.25 (-11.37, 11.87) | 0.97 | -0.54 (-22.92, 21.83) | 0.966 | 0.61 (-28.18, 29.39) | 0.97 | -0.14 (-6.57, 6.3) | 0.97 | -1.15 (-13.4, 11.09) | 0.864 |
| 369.5 | Conjunctivitis, infectious | 17393 | -0.05 (-2.56, 2.46) | 0.97 | 0.13 (-5.82, 6.07) | 0.97 | 0.24 (-9.83, 10.32) | 0.966 | -0.04 (-2.15, 2.06) | 0.97 | 0.02 (-0.96, 1) | 0.97 |
| 370 | Keratitis | 3911 | -0.1 (-5.01, 4.8) | 0.97 | 0.06 (-2.72, 2.83) | 0.97 | 1.05 (-10.15, 12.26) | 0.864 | -0.1 (-5.06, 4.85) | 0.97 | -0.42 (-9.21, 8.37) | 0.932 |
| 370.1 | Corneal ulcer | 378 | -0.47 (-22.9, 21.95) | 0.97 | 2.13 (-55.88, 60.14) | 0.948 | 3.36 (-59.4, 66.12) | 0.923 | -0.97 (-41.07, 39.12) | 0.966 | -1.45 (-61.06, 58.17) | 0.966 |
| 370.3 | Keratoconjunctivitis | 713 | -0.18 (-8.46, 8.1) | 0.97 | 2.81 (-3.42, 9.05) | 0.383 | 1.45 (-37.96, 40.86) | 0.948 | -1.28 (-11.57, 9.01) | 0.82 | -0.28 (-13.33, 12.78) | 0.97 |
| 371 | Inflammation of the eye | 8336 | -0.21 (-5.97, 5.55) | 0.948 | -0.04 (-1.7, 1.63) | 0.97 | 0.18 (-8.44, 8.8) | 0.97 | 0.19 (-5.1, 5.49) | 0.948 | -0.21 (-8.82, 8.4) | 0.966 |
| 371.1 | Uveitis, noninfectious or NOS | 2926 | -0.38 (-8.99, 8.23) | 0.938 | -0.27 (-13.21, 12.66) | 0.97 | -0.1 (-4.69, 4.5) | 0.97 | 0.51 (-5.98, 7) | 0.887 | -0.33 (-14.31, 13.64) | 0.966 |
| 371.2 | Conjunctivitis, noninfectious | 594 | 0.58 (-19.86, 21.02) | 0.96 | -0.17 (-8.43, 8.08) | 0.97 | -1.24 (-52.25, 49.77) | 0.966 | -0.33 (-15.74, 15.09) | 0.97 | -0.64 (-27.08, 25.79) | 0.966 |
| 371.3 | Inflammation of eyelids | 4505 | -0.12 (-5.84, 5.6) | 0.97 | 0.09 (-4.33, 4.51) | 0.97 | 0.02 (-1.04, 1.09) | 0.97 | 0.08 (-3.76, 3.92) | 0.97 | 0.02 (-1.01, 1.05) | 0.97 |
| 371.33 | Noninfectious dermatoses of eyelid | 124 | 0.96 (-38.71, 40.64) | 0.966 | -1.89 (-44.1, 40.32) | 0.936 | 3.71 (-35.75, 43.17) | 0.864 | -1.17 (-42.37, 40.04) | 0.96 | -0.87 (-41.95, 40.21) | 0.97 |
| 372 | Disorders of conjunctiva | 3677 | -0.03 (-1.27, 1.22) | 0.97 | 0.62 (-6.01, 7.26) | 0.864 | -0.36 (-17.39, 16.67) | 0.97 | -0.18 (-7.94, 7.58) | 0.967 | 0.36 (-9.53, 10.25) | 0.948 |
| 374 | Other disorders of eyelids | 7119 | 0.01 (-0.37, 0.38) | 0.97 | -0.05 (-2.55, 2.45) | 0.97 | 0.32 (-12.79, 13.42) | 0.966 | -0.05 (-2.27, 2.18) | 0.97 | -0.1 (-4.6, 4.41) | 0.97 |
| 374.1 | Ectropion or entropion | 1925 | 0.05 (-2.53, 2.64) | 0.97 | -0.74 (-9.19, 7.72) | 0.874 | 1.18 (-11.35, 13.71) | 0.864 | 0 (-0.07, 0.07) | 0.97 | 0.33 (-13.4, 14.06) | 0.966 |
| 374.2 | Lagophthalmos | 302 | 0.43 (-19.98, 20.84) | 0.97 | 0.08 (-3.52, 3.67) | 0.97 | 0.7 (-32.33, 33.72) | 0.97 | -0.6 (-25.42, 24.21) | 0.966 | 1.36 (-27.22, 29.95) | 0.932 |
| 374.3 | Ptosis of eyelid | 4143 | 0.09 (-4.01, 4.18) | 0.97 | 0.07 (-3.42, 3.57) | 0.97 | -0.02 (-0.81, 0.78) | 0.97 | -0.12 (-5.58, 5.35) | 0.97 | -0.23 (-9.59, 9.13) | 0.966 |
| 375 | Disorders of lacrimal system | 1693 | 0.47 (-16.12, 17.06) | 0.96 | 0.78 (-25.32, 26.88) | 0.958 | 0.18 (-8.27, 8.63) | 0.97 | -0.78 (-9.73, 8.17) | 0.874 | 0.32 (-14.85, 15.49) | 0.97 |
| 375.2 | Epiphora | 339 | -0.62 (-26.24, 25) | 0.966 | 1.72 (-33.62, 37.07) | 0.93 | 0.16 (-7.32, 7.63) | 0.97 | 0.04 (-1.98, 2.07) | 0.97 | 0.04 (-1.91, 1.99) | 0.97 |
| 376 | Disorders of the orbit | 303 | -0.1 (-4.69, 4.49) | 0.97 | 0.4 (-18.59, 19.4) | 0.97 | 0.76 (-35.17, 36.69) | 0.97 | -0.19 (-9.15, 8.77) | 0.97 | -0.77 (-37.31, 35.76) | 0.97 |
| 377 | Disorders of optic nerve and visual pathways | 1565 | 0.17 (-8.08, 8.42) | 0.97 | 0.16 (-7.41, 7.73) | 0.97 | -0.09 (-4.55, 4.36) | 0.97 | -0.23 (-11.11, 10.65) | 0.97 | 0.5 (-20.01, 21.01) | 0.966 |
| 377.1 | Optic atrophy | 437 | -0.11 (-5.38, 5.16) | 0.97 | 1.75 (-37.49, 40.99) | 0.936 | -0.52 (-25.32, 24.27) | 0.97 | -0.6 (-28.89, 27.69) | 0.97 | 0.67 (-31.18, 32.53) | 0.97 |
| 377.3 | Optic neuritis/neuropathy | 457 | 0.57 (-19.85, 20.98) | 0.961 | -0.21 (-10.18, 9.76) | 0.97 | 0.08 (-3.8, 3.96) | 0.97 | -0.5 (-20.99, 19.99) | 0.966 | 0.97 (-20.79, 22.73) | 0.936 |
| 378 | Strabismus and other disorders of binocular eye movements | 7239 | -0.48 (-5.63, 4.66) | 0.864 | -0.04 (-1.91, 1.83) | 0.97 | -0.45 (-19.93, 19.03) | 0.967 | 0.59 (-4.16, 5.34) | 0.82 | -0.15 (-7.12, 6.82) | 0.97 |
| 378.1 | Strabismus (not specified as paralytic) | 5499 | -0.33 (-9.23, 8.57) | 0.948 | 0.17 (-8, 8.35) | 0.97 | -0.49 (-21.8, 20.83) | 0.967 | 0.35 (-7.59, 8.3) | 0.936 | -0.48 (-11.16, 10.21) | 0.936 |
| 378.5 | Paralytic strabismus | 1674 | -0.89 (-8.98, 7.2) | 0.84 | -0.67 (-27.99, 26.64) | 0.965 | -0.05 (-2.61, 2.5) | 0.97 | 1.18 (-3.49, 5.85) | 0.634 | 0.36 (-16.5, 17.21) | 0.97 |
| 379 | Other disorders of eye | 15271 | -0.09 (-3.92, 3.73) | 0.966 | 0.02 (-1.09, 1.13) | 0.97 | -0.58 (-5.25, 4.09) | 0.82 | 0.19 (-1.86, 2.24) | 0.865 | 0.07 (-3.02, 3.15) | 0.97 |
| 379.1 | Scleritis and episcleritis | 389 | 0.63 (-25.44, 26.7) | 0.966 | 1.49 (-26.32, 29.3) | 0.923 | -3.41 (-30.88, 24.06) | 0.82 | -0.56 (-23.44, 22.33) | 0.966 | -0.21 (-10.34, 9.91) | 0.97 |
| 379.2 | Disorders of vitreous body | 5579 | -0.31 (-3.55, 2.94) | 0.864 | 0.12 (-5.73, 5.98) | 0.97 | -0.69 (-8, 6.62) | 0.864 | 0.38 (-2.7, 3.47) | 0.82 | -0.19 (-8.05, 7.67) | 0.966 |
| 379.3 | Aphakia and other disorders of lens | 1036 | -0.2 (-9.58, 9.19) | 0.97 | -0.81 (-34.24, 32.62) | 0.966 | -0.37 (-17.67, 16.94) | 0.97 | 0.63 (-25.52, 26.79) | 0.966 | 0.17 (-8.09, 8.44) | 0.97 |
| 379.4 | Anomalies of pupillary function | 247 | 1.13 (-45.28, 47.54) | 0.966 | -0.52 (-25.29, 24.24) | 0.97 | -4.04 (-53.53, 45.46) | 0.882 | 0.15 (-6.95, 7.25) | 0.97 | -0.91 (-43.91, 42.09) | 0.97 |
| 379.5 | Disorders of iris and ciliary body | 504 | 0.12 (-5.77, 6.02) | 0.97 | 1.49 (-29.7, 32.68) | 0.932 | 0.5 (-22.93, 23.93) | 0.97 | -0.76 (-21.45, 19.93) | 0.948 | -0.46 (-22.25, 21.33) | 0.97 |
| 379.9 | Pain, swelling or discharge of eye | 1330 | 0.03 (-1.23, 1.28) | 0.97 | 0.38 (-16.5, 17.27) | 0.968 | -1.62 (-18.84, 15.6) | 0.864 | 0.09 (-4.14, 4.32) | 0.97 | -0.01 (-0.38, 0.36) | 0.97 |
| 380 | Disorders of external ear | 6707 | 0.18 (-7.3, 7.67) | 0.966 | -0.3 (-12.79, 12.18) | 0.966 | -0.4 (-16.82, 16.02) | 0.966 | 0.02 (-0.92, 0.96) | 0.97 | 0.37 (-7.25, 7.99) | 0.931 |
| 380.1 | Otitis externa | 4546 | 0.29 (-7.63, 8.21) | 0.948 | -0.26 (-12.59, 12.07) | 0.97 | -0.35 (-16.74, 16.04) | 0.97 | -0.11 (-5.53, 5.31) | 0.97 | 0.53 (-7.11, 8.17) | 0.9 |
| 380.4 | Impacted cerumen | 1545 | 0.06 (-2.6, 2.72) | 0.97 | -0.69 (-24.95, 23.58) | 0.96 | -1.28 (-28.45, 25.88) | 0.932 | 0.42 (-14.5, 15.35) | 0.96 | 0.01 (-0.57, 0.59) | 0.97 |
| 381 | Otitis media and Eustachian tube disorders | 22791 | -0.34 (-2.82, 2.15) | 0.802 | 0.76 (-0.29, 1.82) | 0.157 | 0.46 (-8.7, 9.61) | 0.929 | -0.07 (-3.41, 3.27) | 0.97 | 0.43 (-3.02, 3.88) | 0.82 |
| 381.1 | Otitis media | 12313 | -0.12 (-5.77, 5.53) | 0.97 | 0.94 (-0.42, 2.3) | 0.175 | 0.3 (-13.04, 13.65) | 0.968 | -0.34 (-3.97, 3.29) | 0.864 | 0.43 (-4.17, 5.03) | 0.864 |
| 381.11 | Suppurative and unspecified otitis media | 11470 | -0.29 (-5.79, 5.2) | 0.923 | 0.54 (-5.16, 6.23) | 0.864 | 0.41 (-16.32, 17.13) | 0.966 | -0.02 (-0.73, 0.7) | 0.97 | 0.52 (-5.06, 6.11) | 0.864 |
| 381.2 | Eustachian tube disorders | 2358 | 0.06 (-2.71, 2.83) | 0.97 | -0.21 (-10.17, 9.75) | 0.97 | -0.26 (-12.33, 11.82) | 0.97 | 0.07 (-3.2, 3.34) | 0.97 | -0.33 (-16.14, 15.47) | 0.97 |
| 381.3 | Mastoiditis & related conditions | 447 | -0.74 (-35.54, 34.07) | 0.97 | 3.81 (-29.35, 36.97) | 0.833 | -0.38 (-18.41, 17.64) | 0.97 | -0.66 (-31.66, 30.35) | 0.97 | 0.1 (-4.73, 4.94) | 0.97 |
| 381.9 | Otorrhea | 110 | -1.93 (-24.06, 20.21) | 0.874 | 0.11 (-4.96, 5.17) | 0.97 | -0.07 (-3.49, 3.34) | 0.97 | 1.91 (-21.9, 25.72) | 0.884 | 1.74 (-45.68, 49.16) | 0.948 |
| 382 | Otalgia | 1061 | -0.42 (-11.94, 11.09) | 0.948 | 0.08 (-3.93, 4.1) | 0.97 | -0.81 (-34.19, 32.57) | 0.966 | 0.53 (-10.39, 11.44) | 0.931 | 0.38 (-17.36, 18.11) | 0.97 |
| 383 | Otosclerosis | 2321 | -0.14 (-6.7, 6.42) | 0.97 | 0.52 (-20.93, 21.97) | 0.966 | 0.52 (-23.95, 24.98) | 0.97 | -0.17 (-8.04, 7.7) | 0.97 | -0.02 (-0.87, 0.84) | 0.97 |
| 384 | Other disorders of tympanic membrane | 3200 | -0.16 (-7.61, 7.29) | 0.97 | 0.53 (-10.03, 11.08) | 0.929 | 0.36 (-16.7, 17.43) | 0.97 | -0.16 (-7.93, 7.6) | 0.97 | -0.45 (-10.63, 9.73) | 0.936 |
| 384.1 | Myringitis | 241 | 0.13 (-5.79, 6.04) | 0.97 | -0.99 (-47.96, 45.98) | 0.97 | 0.41 (-18.89, 19.71) | 0.97 | 0.15 (-6.81, 7.1) | 0.97 | -0.86 (-41.64, 39.91) | 0.97 |
| 384.4 | Perforation of tympanic membrane | 2943 | -0.19 (-8.22, 7.83) | 0.966 | 0.32 (-12.99, 13.63) | 0.966 | 0.39 (-17.88, 18.67) | 0.97 | -0.04 (-1.82, 1.74) | 0.97 | -0.38 (-10.63, 9.88) | 0.948 |
| 385 | Other disorders of middle ear and mastoid | 779 | 0.29 (-13.3, 13.87) | 0.97 | -0.69 (-29.22, 27.83) | 0.966 | -0.77 (-37.39, 35.84) | 0.97 | 0.17 (-8, 8.35) | 0.97 | -1.64 (-15.88, 12.61) | 0.833 |
| 385.3 | Cholesteatoma | 212 | -0.26 (-12.67, 12.15) | 0.97 | 0.86 (-39.83, 41.55) | 0.97 | -3.29 (-38.29, 31.71) | 0.864 | 0.85 (-33.56, 35.25) | 0.965 | 0.06 (-2.8, 2.92) | 0.97 |
| 385.5 | Tympanosclerosis and middle ear disease related to otitis media | 530 | 0.78 (-17.06, 18.62) | 0.938 | -0.99 (-41, 39.02) | 0.965 | 0.63 (-29.12, 30.38) | 0.97 | -0.55 (-23.4, 22.29) | 0.966 | -2.57 (-12.74, 7.61) | 0.634 |
| 386 | Vertiginous syndromes and other disorders of vestibular system | 9954 | -0.24 (-3.36, 2.87) | 0.887 | 0.64 (-2.51, 3.78) | 0.705 | 0.16 (-7.26, 7.58) | 0.97 | -0.03 (-1.27, 1.22) | 0.97 | -0.14 (-6.73, 6.45) | 0.97 |
| 386.1 | Meniere's disease | 1400 | 0.03 (-1.58, 1.65) | 0.97 | 1.9 (-10.36, 14.16) | 0.774 | -0.21 (-10.29, 9.87) | 0.97 | -0.69 (-13.58, 12.2) | 0.923 | -0.19 (-9.31, 8.92) | 0.97 |
| 386.2 | Peripheral or central vertigo | 5291 | -0.2 (-8.54, 8.13) | 0.966 | 0.76 (-5.87, 7.39) | 0.833 | 0.26 (-12.13, 12.66) | 0.97 | -0.13 (-6.48, 6.21) | 0.97 | 0.01 (-0.27, 0.28) | 0.97 |
| 386.21 | Central origin vertigo | 354 | 1.09 (-12.48, 14.66) | 0.884 | -1.15 (-32.47, 30.17) | 0.948 | -3.13 (-30.39, 24.13) | 0.833 | -0.05 (-2.55, 2.44) | 0.97 | -0.12 (-5.76, 5.52) | 0.97 |
| 386.3 | Labyrinthitis | 597 | -0.14 (-6.92, 6.63) | 0.97 | -0.44 (-21.18, 20.3) | 0.97 | 1.28 (-51.58, 54.14) | 0.966 | 0.11 (-5.08, 5.3) | 0.97 | -0.76 (-32.28, 30.75) | 0.966 |
| 386.9 | Dizziness and giddiness (Light-headedness and vertigo) | 1060 | -0.21 (-8.05, 7.63) | 0.962 | 0.22 (-10.33, 10.78) | 0.97 | 0.63 (-16.43, 17.68) | 0.948 | 0.02 (-0.92, 0.96) | 0.97 | 0.03 (-1.24, 1.29) | 0.97 |
| 388 | Other disorders of ear | 5263 | -0.1 (-4.65, 4.46) | 0.97 | -0.22 (-10.59, 10.15) | 0.97 | -0.55 (-15.5, 14.4) | 0.948 | 0.27 (-5.33, 5.88) | 0.93 | 0.41 (-7.24, 8.06) | 0.923 |
| 389 | Hearing loss | 43240 | -0.05 (-2.28, 2.17) | 0.966 | 0.16 (-1.97, 2.29) | 0.89 | 0.02 (-1.15, 1.2) | 0.97 | -0.02 (-0.76, 0.73) | 0.97 | 0.03 (-1.54, 1.61) | 0.97 |
| 389.1 | Sensorineural hearing loss | 9172 | -0.09 (-4.23, 4.05) | 0.97 | 0.18 (-7.38, 7.75) | 0.966 | 0.3 (-12.17, 12.77) | 0.966 | -0.04 (-2.02, 1.93) | 0.97 | 0.24 (-5.1, 5.57) | 0.936 |
| 389.2 | Conductive hearing loss | 2123 | 0.13 (-6.06, 6.33) | 0.97 | 0.21 (-9.71, 10.13) | 0.97 | -0.15 (-7.08, 6.79) | 0.97 | -0.2 (-9.54, 9.14) | 0.97 | -0.11 (-5.51, 5.28) | 0.97 |
| 389.3 | Degenerative and vascular disorders of ear | 22354 | -0.1 (-2.19, 1.99) | 0.931 | 0.23 (-2.18, 2.64) | 0.864 | -0.01 (-0.24, 0.23) | 0.97 | 0.01 (-0.64, 0.66) | 0.97 | -0.08 (-3.58, 3.41) | 0.966 |
| 389.4 | Tinnitus | 6854 | -0.14 (-5.93, 5.65) | 0.966 | -0.02 (-0.97, 0.93) | 0.97 | -0.12 (-5.64, 5.4) | 0.97 | 0.17 (-4.56, 4.91) | 0.948 | -0.1 (-4.77, 4.57) | 0.97 |
| 389.5 | Disorders of acoustic nerve | 776 | -0.48 (-23.41, 22.44) | 0.97 | 1.36 (-35.62, 38.34) | 0.948 | 0.56 (-26.09, 27.22) | 0.97 | -0.15 (-7.45, 7.15) | 0.97 | 0.22 (-10.32, 10.77) | 0.97 |
| 394 | Rheumatic disease of the heart valves | 8428 | 0.02 (-0.82, 0.86) | 0.97 | -0.04 (-2.11, 2.02) | 0.97 | 0.43 (-9.21, 10.07) | 0.936 | -0.07 (-3.49, 3.35) | 0.97 | 0.12 (-5.52, 5.76) | 0.97 |
| 394.2 | Mitral valve disease | 3866 | 0.14 (-6.47, 6.74) | 0.97 | -0.28 (-12.01, 11.44) | 0.966 | -0.16 (-7.5, 7.19) | 0.97 | -0.01 (-0.54, 0.52) | 0.97 | 0.09 (-4.26, 4.44) | 0.97 |
| 394.3 | Aortic valve disease | 5050 | -0.09 (-4.3, 4.12) | 0.97 | -0.05 (-2.21, 2.12) | 0.97 | 0.58 (-11.3, 12.46) | 0.93 | 0.02 (-0.97, 1.01) | 0.97 | 0.07 (-3.11, 3.25) | 0.97 |
| 395 | Heart valve disorders | 7805 | 0.04 (-1.64, 1.71) | 0.97 | -0.2 (-8.24, 7.85) | 0.966 | -0.08 (-3.93, 3.77) | 0.97 | 0.05 (-2.51, 2.62) | 0.97 | -0.2 (-5.52, 5.13) | 0.948 |
| 395.1 | Nonrheumatic mitral valve disorders | 4004 | 0.08 (-3.83, 3.99) | 0.97 | -0.24 (-10.34, 9.85) | 0.966 | -0.25 (-12.1, 11.59) | 0.97 | 0.05 (-2.35, 2.45) | 0.97 | 0.04 (-1.78, 1.86) | 0.97 |
| 395.2 | Nonrheumatic aortic valve disorders | 1475 | -0.05 (-2.63, 2.53) | 0.97 | 0.25 (-11.4, 11.89) | 0.97 | 0.4 (-18.54, 19.34) | 0.97 | -0.12 (-5.61, 5.37) | 0.97 | -0.74 (-8.61, 7.13) | 0.864 |
| 395.3 | Nonrheumatic tricuspid valve disorders | 300 | 0.43 (-19.83, 20.69) | 0.97 | 0.06 (-2.6, 2.71) | 0.97 | -1.22 (-59.14, 56.69) | 0.97 | -0.29 (-14.23, 13.64) | 0.97 | -1.65 (-23.15, 19.86) | 0.89 |
| 395.4 | Nonrheumatic pulmonary valve disorders | 178 | -1.75 (-41.55, 38.05) | 0.937 | -0.56 (-26.92, 25.8) | 0.97 | 2.52 (-101.38, 106.42) | 0.966 | 1.54 (-52.68, 55.76) | 0.96 | -1.09 (-52.81, 50.62) | 0.97 |
| 395.6 | Heart valve replaced | 3668 | 0.2 (-3.92, 4.32) | 0.93 | -0.23 (-8.33, 7.87) | 0.96 | -0.22 (-10.7, 10.26) | 0.97 | -0.07 (-3.53, 3.39) | 0.97 | -0.2 (-7.16, 6.77) | 0.96 |
| 396 | Abnormal heart sounds | 1638 | -0.02 (-1.16, 1.11) | 0.97 | -0.08 (-3.88, 3.72) | 0.97 | 0.51 (-20.44, 21.46) | 0.966 | -0.03 (-1.28, 1.23) | 0.97 | 0.03 (-1.28, 1.34) | 0.97 |
| 401 | Hypertension | 11768 | 0.15 (-6.72, 7.01) | 0.97 | -0.07 (-3.24, 3.1) | 0.97 | -0.83 (-11.35, 9.7) | 0.887 | 0.03 (-1.4, 1.46) | 0.97 | 0.16 (-7.43, 7.75) | 0.97 |
| 401.1 | Essential hypertension | 3161 | -0.13 (-6.21, 5.95) | 0.97 | 0 (-0.2, 0.21) | 0.97 | -0.4 (-16.77, 15.97) | 0.966 | 0.21 (-5.45, 5.87) | 0.948 | 0.29 (-7.6, 8.18) | 0.948 |
| 401.2 | Hypertensive heart and/or renal disease | 281 | 0.06 (-2.77, 2.89) | 0.97 | -1.14 (-24.65, 22.36) | 0.93 | -0.05 (-2.21, 2.11) | 0.97 | 0.46 (-18.39, 19.3) | 0.966 | 0.32 (-14.89, 15.53) | 0.97 |
| 401.21 | Hypertensive heart disease | 3905 | -0.11 (-5.1, 4.89) | 0.97 | 0.02 (-1.11, 1.16) | 0.97 | -0.01 (-0.4, 0.39) | 0.97 | 0.1 (-4.6, 4.8) | 0.97 | 0 (-0.04, 0.04) | 0.97 |
| 401.22 | Hypertensive chronic kidney disease | 858 | -0.11 (-5.26, 5.05) | 0.97 | -0.42 (-20.11, 19.27) | 0.97 | -1.97 (-22.9, 18.96) | 0.864 | 0.6 (-10.67, 11.87) | 0.923 | -0.16 (-7.56, 7.25) | 0.97 |
| 401.3 | Other hypertensive complications | 4449 | 0.16 (-6.13, 6.45) | 0.963 | 0.04 (-1.88, 1.96) | 0.97 | 0.05 (-2.24, 2.33) | 0.97 | -0.19 (-5.39, 5.01) | 0.948 | 0.26 (-6.82, 7.34) | 0.948 |
| 402 | Elevated blood pressure reading without diagnosis of hypertension | 462 | 0.18 (-8.4, 8.76) | 0.97 | 0.03 (-1.31, 1.37) | 0.97 | 0.56 (-25.75, 26.86) | 0.97 | -0.28 (-11.87, 11.31) | 0.966 | 0.52 (-13.68, 14.72) | 0.948 |
| 411 | Ischemic Heart Disease | 54856 | -0.02 (-1.14, 1.09) | 0.97 | -0.12 (-3.32, 3.08) | 0.947 | -0.18 (-4.68, 4.31) | 0.942 | 0.1 (-1.23, 1.44) | 0.887 | 0.04 (-1.7, 1.78) | 0.97 |
| 411.1 | Unstable angina (intermediate coronary syndrome) | 8841 | 0.03 (-1.46, 1.52) | 0.97 | -0.11 (-5.26, 5.05) | 0.97 | -0.42 (-5.7, 4.87) | 0.887 | 0.09 (-3.6, 3.78) | 0.966 | -0.06 (-3.01, 2.89) | 0.97 |
| 411.2 | Myocardial infarction | 25907 | -0.22 (-1.98, 1.55) | 0.82 | -0.18 (-4.95, 4.6) | 0.948 | -0.42 (-4.84, 4.01) | 0.864 | 0.37 (-0.02, 0.77) | 0.062 | 0.15 (-3.94, 4.24) | 0.948 |
| 411.3 | Angina pectoris | 27819 | -0.04 (-1.91, 1.83) | 0.97 | 0.08 (-3.59, 3.74) | 0.97 | 0.02 (-1.13, 1.18) | 0.97 | 0 (-0.18, 0.19) | 0.97 | -0.03 (-1.52, 1.45) | 0.97 |
| 411.4 | Coronary atherosclerosis | 18006 | -0.04 (-2.09, 2) | 0.97 | -0.12 (-3.5, 3.25) | 0.948 | 0.05 (-2.45, 2.56) | 0.97 | 0.09 (-2.27, 2.45) | 0.948 | 0.05 (-2.33, 2.43) | 0.97 |
| 411.41 | Aneurysm and dissection of heart | 721 | 0.12 (-5.34, 5.57) | 0.97 | -0.68 (-19.11, 17.75) | 0.948 | 0.38 (-17.57, 18.33) | 0.97 | 0.09 (-4.36, 4.55) | 0.97 | -0.6 (-16.8, 15.61) | 0.948 |
| 411.8 | Other chronic ischemic heart disease, unspecified | 26664 | 0.08 (-2.07, 2.23) | 0.948 | -0.2 (-2.34, 1.94) | 0.864 | -0.18 (-5.06, 4.7) | 0.948 | 0.03 (-1.53, 1.59) | 0.97 | 0.1 (-3.36, 3.56) | 0.96 |
| 411.9 | Other acute and subacute forms of ischemic heart disease | 2173 | 0.08 (-3.74, 3.9) | 0.97 | 0.12 (-5.62, 5.87) | 0.97 | -0.1 (-5, 4.79) | 0.97 | -0.11 (-5.55, 5.32) | 0.97 | 0 (0, 0) | 0.97 |
| 414 | Other forms of chronic heart disease | 3481 | 0.55 (-3.86, 4.96) | 0.82 | -0.4 (-11.28, 10.48) | 0.948 | 0.69 (-16.34, 17.73) | 0.942 | -0.5 (-4.89, 3.88) | 0.833 | 0.15 (-6.74, 7.04) | 0.97 |
| 415 | Pulmonary heart disease | 10881 | -0.25 (-2.88, 2.38) | 0.864 | -0.15 (-7.18, 6.88) | 0.97 | -0.12 (-5.9, 5.66) | 0.97 | 0.36 (-2.56, 3.29) | 0.82 | 0.06 (-2.99, 3.12) | 0.97 |
| 415.11 | Pulmonary embolism and infarction, acute | 1533 | 0.01 (-0.5, 0.53) | 0.97 | -1.17 (-32.89, 30.56) | 0.948 | -0.16 (-7.6, 7.29) | 0.97 | 0.59 (-23.82, 25) | 0.966 | -0.01 (-0.62, 0.6) | 0.97 |
| 415.2 | Chronic pulmonary heart disease | 1458 | -0.17 (-8.34, 7.99) | 0.97 | 0.39 (-15.82, 16.61) | 0.966 | -0.56 (-23.55, 22.43) | 0.966 | 0.15 (-7.15, 7.46) | 0.97 | 0.21 (-9.65, 10.07) | 0.97 |
| 415.21 | Primary pulmonary hypertension | 1040 | 0.6 (-11.5, 12.7) | 0.929 | -0.18 (-8.84, 8.47) | 0.97 | 1.56 (-27.53, 30.64) | 0.923 | -0.77 (-8.94, 7.41) | 0.864 | -0.35 (-16.8, 16.11) | 0.97 |
| 416 | Cardiomegaly | 338 | 0.45 (-20.69, 21.58) | 0.97 | -0.01 (-0.43, 0.41) | 0.97 | -2.66 (-74.97, 69.66) | 0.948 | 0 (-0.21, 0.2) | 0.97 | -1.36 (-38.48, 35.75) | 0.948 |
| 418 | Nonspecific chest pain | 21599 | -0.01 (-0.47, 0.45) | 0.97 | 0.1 (-4, 4.2) | 0.966 | 0.04 (-1.67, 1.74) | 0.97 | -0.04 (-1.92, 1.84) | 0.97 | 0.05 (-2.24, 2.34) | 0.97 |
| 418.1 | Precordial pain | 1106 | -0.68 (-14.69, 13.32) | 0.93 | 0.07 (-3.17, 3.31) | 0.97 | -0.25 (-12.19, 11.68) | 0.97 | 0.7 (-13.66, 15.06) | 0.93 | 0.42 (-19.55, 20.4) | 0.97 |
| 420 | Carditis | 5370 | 0.11 (-4.96, 5.18) | 0.97 | 0.7 (-6.79, 8.19) | 0.864 | 0.08 (-3.76, 3.93) | 0.97 | -0.42 (-4.89, 4.05) | 0.864 | 0.23 (-10.43, 10.88) | 0.97 |
| 420.1 | Myocarditis | 666 | 0.31 (-14.17, 14.79) | 0.97 | 1.83 (-21.39, 25.05) | 0.887 | -1.69 (-71.43, 68.05) | 0.966 | -0.84 (-23.84, 22.15) | 0.948 | 1.15 (-30.05, 32.34) | 0.948 |
| 420.2 | Pericarditis | 1307 | 0.72 (-6.9, 8.34) | 0.864 | 1.17 (-11.29, 13.63) | 0.864 | -1.13 (-31.8, 29.54) | 0.948 | -1.03 (-6.9, 4.84) | 0.744 | -0.11 (-5.42, 5.2) | 0.97 |
| 420.21 | Acute pericarditis | 2187 | -0.18 (-8.58, 8.22) | 0.97 | 0.18 (-8.11, 8.46) | 0.97 | -0.05 (-2.47, 2.36) | 0.97 | 0.12 (-5.55, 5.79) | 0.97 | 0.5 (-20.22, 21.23) | 0.966 |
| 420.22 | Chronic pericarditis | 398 | 1.2 (-23.99, 26.39) | 0.932 | 0.25 (-11.34, 11.83) | 0.97 | -2.53 (-59.95, 54.9) | 0.937 | -0.86 (-36.3, 34.58) | 0.966 | 1.28 (-47.54, 50.1) | 0.963 |
| 420.3 | Endocarditis | 1520 | 0.04 (-1.75, 1.82) | 0.97 | -0.42 (-20.11, 19.28) | 0.97 | 0.71 (-30.39, 31.82) | 0.967 | 0.01 (-0.29, 0.3) | 0.97 | -0.62 (-14.61, 13.36) | 0.936 |
| 425 | Cardiomyopathy | 3919 | -0.1 (-4.83, 4.63) | 0.97 | 0.15 (-6.84, 7.14) | 0.97 | 0.35 (-15.27, 15.98) | 0.968 | -0.02 (-1.15, 1.1) | 0.97 | -0.07 (-3.17, 3.04) | 0.97 |
| 425.1 | Primary/intrinsic cardiomyopathies | 3331 | -0.12 (-5.69, 5.46) | 0.97 | 0.17 (-7.81, 8.15) | 0.97 | 0.49 (-19.86, 20.85) | 0.966 | -0.04 (-1.87, 1.79) | 0.97 | -0.15 (-7.16, 6.86) | 0.97 |
| 425.11 | Hypertrophic obstructive cardiomyopathy | 413 | -0.63 (-17.8, 16.54) | 0.948 | 0.05 (-2.24, 2.34) | 0.97 | 2.57 (-45.39, 50.53) | 0.923 | 0.33 (-15.28, 15.94) | 0.97 | -0.07 (-3.48, 3.34) | 0.97 |
| 425.12 | Other hypertrophic cardiomyopathy | 472 | 0.14 (-6.42, 6.7) | 0.97 | 1.47 (-17.27, 20.22) | 0.887 | -1.19 (-50.13, 47.76) | 0.966 | -0.43 (-18.02, 17.17) | 0.966 | -0.28 (-13.35, 12.79) | 0.97 |
| 425.8 | Other cardiomyopathy | 140 | -1.5 (-29.58, 26.58) | 0.923 | -1.25 (-60.46, 57.95) | 0.97 | 1.62 (-74.95, 78.18) | 0.97 | 1.53 (-27.02, 30.08) | 0.923 | 0.13 (-6.04, 6.3) | 0.97 |
| 426 | Cardiac conduction disorders | 13823 | 0.02 (-0.89, 0.93) | 0.97 | 0.15 (-5.42, 5.72) | 0.961 | -0.21 (-8.98, 8.56) | 0.966 | -0.04 (-2.15, 2.07) | 0.97 | 0.11 (-4.29, 4.51) | 0.966 |
| 426.2 | Atrioventricular [AV] block | 897 | 0.22 (-9.96, 10.39) | 0.97 | 0.06 (-2.68, 2.8) | 0.97 | 0.62 (-28.89, 30.14) | 0.97 | -0.35 (-15.7, 15) | 0.967 | -0.14 (-6.61, 6.33) | 0.97 |
| 426.21 | First degree AV block | 455 | 0.66 (-13.17, 14.49) | 0.932 | -0.16 (-7.94, 7.61) | 0.97 | 2.56 (-29.97, 35.09) | 0.887 | -0.85 (-11.43, 9.73) | 0.884 | 1.02 (-16.86, 18.89) | 0.918 |
| 426.23 | Second degree AV block | 1305 | -0.51 (-5.98, 4.95) | 0.864 | 0.4 (-15.95, 16.74) | 0.966 | 0.02 (-0.74, 0.77) | 0.97 | 0.36 (-7.81, 8.54) | 0.936 | 0.34 (-13.47, 14.14) | 0.966 |
| 426.24 | Atrioventricular block, complete | 2950 | -0.08 (-3.99, 3.83) | 0.97 | -0.06 (-2.67, 2.56) | 0.97 | 0.13 (-5.96, 6.22) | 0.97 | 0.08 (-3.74, 3.9) | 0.97 | -0.05 (-2.65, 2.54) | 0.97 |
| 426.25 | Other heart block | 1227 | 0.18 (-8.23, 8.59) | 0.97 | 0.73 (-15.53, 16.99) | 0.936 | -1.2 (-25.24, 22.85) | 0.929 | -0.26 (-12.67, 12.14) | 0.97 | -0.29 (-14.2, 13.61) | 0.97 |
| 426.3 | Bundle branch block | 353 | -0.93 (-39.13, 37.27) | 0.966 | 0.7 (-32.34, 33.74) | 0.97 | -1.49 (-71.92, 68.94) | 0.97 | 0.96 (-37.81, 39.74) | 0.965 | 0.21 (-9.63, 10.05) | 0.97 |
| 426.31 | Right bundle branch block | 255 | 0.66 (-26.5, 27.82) | 0.966 | -1.1 (-35.73, 33.54) | 0.955 | -2.43 (-52.38, 47.51) | 0.93 | 0.2 (-9.2, 9.59) | 0.97 | -0.3 (-14.45, 13.85) | 0.97 |
| 426.32 | Left bundle branch block | 522 | -0.21 (-9.92, 9.51) | 0.97 | -0.52 (-23.37, 22.33) | 0.968 | 0.09 (-3.99, 4.16) | 0.97 | 0.41 (-16.59, 17.41) | 0.966 | -0.01 (-0.24, 0.23) | 0.97 |
| 426.4 | Anomalous atrioventricular excitation | 968 | 0.32 (-12.82, 13.46) | 0.966 | -0.5 (-20.96, 19.96) | 0.966 | 0.79 (-31.79, 33.37) | 0.966 | -0.27 (-12.87, 12.33) | 0.97 | 0.11 (-5.01, 5.23) | 0.97 |
| 426.9 | Cardiac pacemaker/device in situ | 471 | 0.34 (-13.53, 14.21) | 0.966 | -0.9 (-19.37, 17.57) | 0.93 | 1.46 (-27.83, 30.74) | 0.929 | -0.23 (-11.12, 10.66) | 0.97 | -0.88 (-12.14, 10.37) | 0.887 |
| 426.91 | Cardiac pacemaker in situ | 9546 | -0.14 (-1.91, 1.63) | 0.887 | 0.05 (-2.42, 2.52) | 0.97 | -0.14 (-6.96, 6.67) | 0.97 | 0.14 (-1.7, 1.99) | 0.887 | 0.34 (-0.99, 1.67) | 0.634 |
| 427 | Cardiac dysrhythmias | 32726 | 0.01 (-0.62, 0.64) | 0.97 | 0.06 (-2.73, 2.84) | 0.97 | -0.01 (-0.36, 0.34) | 0.97 | -0.04 (-1.75, 1.68) | 0.97 | 0.33 (-0.97, 1.63) | 0.634 |
| 427.1 | Paroxysmal tachycardia, unspecified | 3178 | 0.31 (-8.25, 8.88) | 0.948 | 0.44 (-17.67, 18.55) | 0.966 | -0.86 (-19.01, 17.3) | 0.932 | -0.33 (-9.42, 8.75) | 0.948 | 0.59 (-7.9, 9.08) | 0.9 |
| 427.11 | Paroxysmal supraventricular tachycardia | 9108 | -0.04 (-1.71, 1.64) | 0.97 | -0.19 (-8.61, 8.23) | 0.967 | -0.04 (-1.93, 1.85) | 0.97 | 0.12 (-5.12, 5.36) | 0.968 | 0.36 (-4.18, 4.9) | 0.887 |
| 427.12 | Paroxysmal ventricular tachycardia | 2922 | 0.42 (-4, 4.83) | 0.864 | -1.26 (-2.81, 0.29) | 0.112 | 0.46 (-18.46, 19.37) | 0.966 | 0.05 (-2.5, 2.61) | 0.97 | 0.42 (-8.02, 8.86) | 0.929 |
| 427.2 | Atrial fibrillation and flutter | 889 | 0.71 (-15.7, 17.12) | 0.938 | 2.09 (-16.1, 20.28) | 0.833 | -2.02 (-39.72, 35.68) | 0.923 | -1.12 (-12.98, 10.75) | 0.864 | 0.78 (-22.34, 23.89) | 0.952 |
| 427.3 | Other specified cardiac dysrhythmias | 3827 | -0.03 (-1.36, 1.3) | 0.97 | 0.01 (-0.43, 0.45) | 0.97 | 0.12 (-5.36, 5.59) | 0.97 | 0 (-0.13, 0.13) | 0.97 | 0.21 (-8.61, 9.04) | 0.966 |
| 427.41 | Ventricular fibrillation and flutter | 803 | -0.63 (-13.49, 12.24) | 0.93 | -0.23 (-11.01, 10.55) | 0.97 | -0.26 (-12.54, 12.02) | 0.97 | 0.78 (-9.08, 10.64) | 0.886 | -0.26 (-12.76, 12.23) | 0.97 |
| 427.42 | Cardiac arrest | 4179 | 0.09 (-3.97, 4.16) | 0.968 | 0.03 (-1.31, 1.37) | 0.97 | 0.01 (-0.57, 0.6) | 0.97 | -0.11 (-4.51, 4.29) | 0.966 | 0.07 (-3.42, 3.56) | 0.97 |
| 427.5 | Arrhythmia (cardiac) NOS | 3360 | 0.01 (-0.45, 0.47) | 0.97 | 0.46 (-10.05, 10.96) | 0.938 | -1.16 (-13.45, 11.14) | 0.864 | 0 (-0.12, 0.11) | 0.97 | 0.14 (-6.6, 6.89) | 0.97 |
| 427.6 | Premature beats | 3820 | 0.34 (-6.7, 7.38) | 0.93 | -0.76 (-8.82, 7.3) | 0.864 | 0.9 (-15.13, 16.93) | 0.919 | -0.21 (-8.91, 8.49) | 0.966 | 0.71 (-5.44, 6.85) | 0.833 |
| 427.61 | Supraventricular premature beats | 1822 | 0.39 (-10.16, 10.94) | 0.948 | -0.06 (-3.08, 2.95) | 0.97 | 1.28 (-15.49, 18.06) | 0.89 | -0.59 (-7.31, 6.14) | 0.874 | -0.84 (-9.83, 8.14) | 0.864 |
| 427.7 | Tachycardia NOS | 2284 | -0.49 (-9.62, 8.65) | 0.923 | 0.09 (-4.29, 4.48) | 0.97 | 0.65 (-26.3, 27.61) | 0.966 | 0.34 (-12.41, 13.09) | 0.962 | 1.31 (-3.89, 6.52) | 0.634 |
| 427.8 | Sinoatrial node dysfunction (Bradycardia) | 2604 | -0.17 (-7.34, 7) | 0.966 | 0.4 (-7.71, 8.51) | 0.929 | -0.09 (-4.34, 4.16) | 0.97 | 0.03 (-1.16, 1.21) | 0.97 | 0.4 (-4.63, 5.42) | 0.887 |
| 427.9 | Palpitations | 6952 | -0.02 (-1.08, 1.03) | 0.97 | -0.02 (-0.78, 0.75) | 0.97 | 0.02 (-0.78, 0.81) | 0.97 | 0.03 (-1.22, 1.27) | 0.97 | 0.27 (-3.13, 3.67) | 0.887 |
| 428 | Congestive heart failure; nonhypertensive | 25731 | -0.06 (-2.49, 2.36) | 0.963 | 0.03 (-1.32, 1.38) | 0.97 | 0.11 (-4.93, 5.16) | 0.968 | 0.03 (-1.56, 1.62) | 0.97 | 0.06 (-2.81, 2.93) | 0.97 |
| 428.1 | Congestive heart failure (CHF) NOS | 8359 | -0.17 (-1.99, 1.65) | 0.864 | -0.06 (-2.77, 2.66) | 0.97 | 0.16 (-6.79, 7.11) | 0.967 | 0.17 (-1.64, 1.97) | 0.864 | 0.22 (-2.12, 2.56) | 0.864 |
| 428.2 | Heart failure NOS | 23387 | -0.04 (-2.02, 1.94) | 0.97 | 0.04 (-1.86, 1.94) | 0.97 | 0.07 (-3.4, 3.54) | 0.97 | 0.01 (-0.65, 0.68) | 0.97 | 0.04 (-1.91, 2) | 0.97 |
| 429 | Ill-defined descriptions and complications of heart disease | 896 | -0.07 (-3.3, 3.17) | 0.97 | 0.03 (-1.26, 1.32) | 0.97 | 1.15 (-23.03, 25.33) | 0.932 | -0.11 (-5.15, 4.94) | 0.97 | -0.48 (-13.56, 12.6) | 0.948 |
| 429.1 | Heart transplant/surgery | 237 | 0.09 (-4.38, 4.57) | 0.97 | -0.92 (-38.95, 37.1) | 0.966 | 2.17 (-87.26, 91.6) | 0.966 | 0.07 (-3.33, 3.48) | 0.97 | -0.38 (-18.26, 17.5) | 0.97 |
| 429.3 | Symptoms involving cardiovascular system | 580 | -0.2 (-9.84, 9.43) | 0.97 | 0.12 (-5.56, 5.8) | 0.97 | 1.36 (-15.95, 18.67) | 0.887 | -0.05 (-2.29, 2.19) | 0.97 | -0.18 (-8.92, 8.55) | 0.97 |
| 430 | Intracranial hemorrhage | 7797 | -0.14 (-5.94, 5.66) | 0.966 | 0.41 (-7.18, 7.99) | 0.923 | 0.36 (-14.29, 15) | 0.966 | -0.08 (-3.78, 3.63) | 0.97 | 0.02 (-0.73, 0.77) | 0.97 |
| 430.1 | Subarachnoid hemorrhage | 2756 | -0.52 (-7.33, 6.29) | 0.89 | 0.12 (-5.73, 5.98) | 0.97 | 1.43 (-13.73, 16.58) | 0.864 | 0.22 (-10.14, 10.57) | 0.97 | 0.12 (-5.53, 5.77) | 0.97 |
| 430.2 | Intracerebral hemorrhage | 4771 | 0.03 (-1.41, 1.47) | 0.97 | 0.42 (-8.33, 9.18) | 0.931 | -0.12 (-5.82, 5.58) | 0.97 | -0.17 (-7.29, 6.95) | 0.966 | -0.14 (-6.65, 6.38) | 0.97 |
| 430.3 | Subdural hemorrhage | 440 | 0.4 (-18.52, 19.32) | 0.97 | 0.41 (-18.87, 19.69) | 0.97 | -1.73 (-73.08, 69.62) | 0.966 | -0.42 (-20.04, 19.21) | 0.97 | -0.06 (-2.94, 2.82) | 0.97 |
| 433 | Cerebrovascular disease | 37172 | -0.05 (-2.41, 2.3) | 0.967 | -0.1 (-4.27, 4.07) | 0.966 | -0.17 (-6.28, 5.94) | 0.961 | 0.13 (-1.21, 1.46) | 0.864 | -0.01 (-0.72, 0.69) | 0.97 |
| 433.1 | Occlusion and stenosis of precerebral arteries | 2798 | 0.12 (-5.47, 5.71) | 0.97 | -0.2 (-9.63, 9.23) | 0.97 | -0.84 (-18.2, 16.52) | 0.931 | 0.1 (-4.4, 4.59) | 0.97 | 0.38 (-9.87, 10.62) | 0.948 |
| 433.11 | Occlusion of cerebral arteries, with cerebral infarction | 950 | 0.73 (-5.18, 6.64) | 0.82 | 0.13 (-5.98, 6.23) | 0.97 | -1.97 (-11.73, 7.78) | 0.705 | -0.46 (-9.84, 8.91) | 0.929 | 0.41 (-16.3, 17.11) | 0.966 |
| 433.12 | Cerebral atherosclerosis | 293 | -0.42 (-20.08, 19.25) | 0.97 | 0.73 (-33.82, 35.28) | 0.97 | -1.81 (-87.17, 83.56) | 0.97 | 0.39 (-17.85, 18.62) | 0.97 | 0.39 (-17.87, 18.65) | 0.97 |
| 433.2 | Occlusion of cerebral arteries | 17998 | -0.11 (-2.25, 2.04) | 0.929 | 0.14 (-3.65, 3.92) | 0.948 | -0.15 (-6.2, 5.9) | 0.966 | 0.08 (-2.31, 2.47) | 0.952 | -0.14 (-3.02, 2.74) | 0.929 |
| 433.21 | Cerebral artery occlusion, with cerebral infarction | 3012 | 0.5 (-9.46, 10.45) | 0.929 | 0.03 (-1.55, 1.61) | 0.97 | 1.33 (-15.52, 18.17) | 0.887 | -0.76 (-7.41, 5.88) | 0.833 | 0.56 (-12.24, 13.36) | 0.938 |
| 433.3 | Cerebral ischemia | 222 | -1.76 (-34.59, 31.07) | 0.923 | -1.45 (-64.87, 61.96) | 0.967 | 1.54 (-71.43, 74.52) | 0.97 | 2.05 (-23.96, 28.06) | 0.887 | -1.21 (-57.88, 55.46) | 0.97 |
| 433.31 | Transient cerebral ischemia | 12202 | -0.11 (-4.85, 4.62) | 0.966 | -0.24 (-6.74, 6.26) | 0.948 | -0.28 (-11.63, 11.08) | 0.966 | 0.26 (-2.49, 3.01) | 0.864 | -0.09 (-4.34, 4.16) | 0.97 |
| 433.5 | Cerebral aneurysm | 848 | -0.14 (-6.8, 6.52) | 0.97 | -0.15 (-7.15, 6.85) | 0.97 | -0.58 (-25.69, 24.54) | 0.967 | 0.32 (-12.8, 13.44) | 0.966 | -0.17 (-8.18, 7.84) | 0.97 |
| 433.8 | Late effects of cerebrovascular disease | 17385 | 0 (-0.13, 0.13) | 0.97 | -0.15 (-3.33, 3.02) | 0.932 | -0.26 (-4.73, 4.21) | 0.915 | 0.1 (-1.99, 2.2) | 0.929 | -0.01 (-0.64, 0.62) | 0.97 |
| 440 | Atherosclerosis | 10903 | -0.25 (-2.86, 2.37) | 0.864 | 0.18 (-7.2, 7.56) | 0.966 | 0 (0, 0) | 0.97 | 0.18 (-3.49, 3.85) | 0.93 | -0.2 (-5.61, 5.21) | 0.948 |
| 440.1 | Atherosclerosis of renal artery | 190 | 1.36 (-54.66, 57.38) | 0.966 | 1.49 (-63.71, 66.7) | 0.967 | 1.25 (-57.99, 60.5) | 0.97 | -2.49 (-28.99, 24.01) | 0.864 | -0.07 (-3.48, 3.34) | 0.97 |
| 440.2 | Atherosclerosis of the extremities | 8349 | -0.09 (-4.54, 4.35) | 0.97 | 0.17 (-7.32, 7.66) | 0.968 | -0.23 (-11.09, 10.63) | 0.97 | 0.07 (-3.17, 3.31) | 0.97 | -0.2 (-8.39, 8) | 0.966 |
| 440.9 | Atherosclerosis of aorta | 293 | -0.57 (-27.52, 26.38) | 0.97 | 3.88 (-29.85, 37.6) | 0.833 | 1.27 (-58.79, 61.33) | 0.97 | -1.12 (-45.47, 43.22) | 0.964 | -3.55 (-34.41, 27.32) | 0.833 |
| 441 | Vascular insufficiency of intestine | 1471 | -0.13 (-6.16, 5.91) | 0.97 | 0.16 (-7.38, 7.7) | 0.97 | 0.08 (-3.6, 3.76) | 0.97 | 0.06 (-2.57, 2.68) | 0.97 | -0.01 (-0.61, 0.59) | 0.97 |
| 441.1 | Acute vascular insufficiency of intestine | 666 | -0.66 (-7.69, 6.37) | 0.864 | 0.68 (-17.73, 19.08) | 0.948 | 0.46 (-21.08, 21.99) | 0.97 | 0.35 (-12.83, 13.52) | 0.963 | -0.19 (-9.27, 8.89) | 0.97 |
| 441.2 | Chronic vascular insufficiency of intestine | 650 | 0.18 (-8.29, 8.64) | 0.97 | 0.19 (-8.74, 9.12) | 0.97 | -1 (-21.19, 19.18) | 0.929 | -0.1 (-4.75, 4.56) | 0.97 | 0.47 (-10.06, 11.01) | 0.936 |
| 442 | Other aneurysm | 7542 | -0.01 (-0.37, 0.35) | 0.97 | 0.34 (-3.31, 4) | 0.864 | -0.2 (-9.45, 9.05) | 0.97 | -0.09 (-4.22, 4.03) | 0.967 | -0.22 (-4.82, 4.37) | 0.93 |
| 442.1 | Aortic aneurysm | 2720 | 0.25 (-4.76, 5.26) | 0.929 | 0.01 (-0.5, 0.52) | 0.97 | -0.71 (-9.76, 8.34) | 0.887 | -0.15 (-6.13, 5.84) | 0.966 | -0.08 (-3.85, 3.69) | 0.97 |
| 442.11 | Abdominal aortic aneurysm | 4104 | 0.04 (-1.66, 1.73) | 0.97 | 0.11 (-5.13, 5.35) | 0.97 | 0.13 (-5.92, 6.17) | 0.97 | -0.1 (-4.62, 4.41) | 0.967 | -0.31 (-4.12, 3.51) | 0.884 |
| 442.2 | Aneurysm of iliac artery | 326 | 0.77 (-7.33, 8.87) | 0.862 | -0.41 (-19.7, 18.88) | 0.97 | -1.02 (-24.27, 22.23) | 0.937 | -0.35 (-14.76, 14.06) | 0.966 | -0.48 (-13.68, 12.71) | 0.948 |
| 442.3 | Aneurysm of artery of lower extremity | 502 | -0.44 (-12.5, 11.61) | 0.948 | 0.16 (-7.47, 7.8) | 0.97 | 0.01 (-0.65, 0.67) | 0.97 | 0.39 (-15.58, 16.35) | 0.966 | 0.12 (-5.54, 5.78) | 0.97 |
| 442.4 | Arterial dissection | 174 | 0.52 (-20.92, 21.96) | 0.966 | 1.06 (-27.7, 29.81) | 0.948 | -3.5 (-31.7, 24.7) | 0.82 | -0.39 (-18.82, 18.04) | 0.97 | 0.03 (-1.36, 1.42) | 0.97 |
| 442.8 | Aneurysm of other specified artery | 891 | -0.28 (-11.98, 11.41) | 0.966 | 1.1 (-8.47, 10.68) | 0.833 | 0.24 (-11.23, 11.72) | 0.97 | -0.13 (-6.45, 6.18) | 0.97 | 0.02 (-0.91, 0.95) | 0.97 |
| 443 | Peripheral vascular disease | 13793 | 0.07 (-3.14, 3.29) | 0.968 | 0.19 (-4.48, 4.86) | 0.942 | -0.15 (-7.36, 7.05) | 0.97 | -0.13 (-2.94, 2.69) | 0.936 | -0.13 (-5.55, 5.28) | 0.965 |
| 443.1 | Raynaud's syndrome | 1003 | -0.17 (-8.22, 7.88) | 0.97 | 0.67 (-28.56, 29.9) | 0.967 | -1.08 (-48.31, 46.15) | 0.967 | 0.08 (-3.78, 3.94) | 0.97 | -0.03 (-1.3, 1.25) | 0.97 |
| 443.7 | Peripheral angiopathy in diseases classified elsewhere | 3677 | 0.11 (-5.27, 5.5) | 0.97 | 0.64 (-4.95, 6.23) | 0.833 | 0.56 (-11.68, 12.8) | 0.935 | -0.51 (-2.8, 1.79) | 0.68 | -0.08 (-4.02, 3.86) | 0.97 |
| 443.8 | Other specified peripheral vascular diseases | 220 | 1.03 (-41.59, 43.65) | 0.966 | -0.49 (-23.63, 22.65) | 0.97 | -1.35 (-65.13, 62.43) | 0.97 | -0.55 (-26.41, 25.31) | 0.97 | 3.55 (-31.27, 38.38) | 0.852 |
| 443.9 | Peripheral vascular disease, unspecified | 9900 | 0.1 (-2.73, 2.94) | 0.948 | 0.03 (-1.46, 1.52) | 0.97 | -0.35 (-6.21, 5.5) | 0.913 | -0.06 (-2.71, 2.6) | 0.97 | -0.29 (-2.78, 2.2) | 0.833 |
| 444 | Arterial embolism and thrombosis | 2391 | -0.37 (-10.5, 9.76) | 0.948 | -0.11 (-5.28, 5.06) | 0.97 | -0.67 (-28.36, 27.02) | 0.966 | 0.61 (-5.89, 7.11) | 0.864 | -0.46 (-17.54, 16.62) | 0.962 |
| 444.1 | Arterial embolism and thrombosis of lower extremity artery | 1286 | -0.72 (-8.35, 6.91) | 0.864 | 0.43 (-20.05, 20.92) | 0.97 | 0.16 (-7.46, 7.79) | 0.97 | 0.54 (-10.89, 11.97) | 0.932 | 0.03 (-1.53, 1.6) | 0.97 |
| 446 | Polyarteritis nodosa and allied conditions | 2765 | 0.26 (-10.26, 10.77) | 0.966 | 0.21 (-9.54, 9.95) | 0.97 | -0.77 (-21.82, 20.27) | 0.948 | -0.21 (-10.13, 9.71) | 0.97 | 0.52 (-10.41, 11.45) | 0.932 |
| 446.3 | Hypersensitivity angiitis | 156 | 1.11 (-29.11, 31.33) | 0.948 | -2.74 (-63.93, 58.44) | 0.936 | 3.2 (-62.76, 69.15) | 0.931 | -1.07 (-38.73, 36.59) | 0.96 | 0.59 (-27.51, 28.7) | 0.97 |
| 446.4 | Wegener's granulomatosis | 333 | 0.71 (-28.45, 29.87) | 0.966 | 0.71 (-32.92, 34.34) | 0.97 | -0.11 (-5.08, 4.87) | 0.97 | -1.03 (-22.56, 20.5) | 0.932 | -0.34 (-16.45, 15.77) | 0.97 |
| 446.5 | Giant cell arteritis | 1661 | 0.32 (-11.11, 11.74) | 0.961 | -0.46 (-19.23, 18.32) | 0.966 | 0.57 (-26.53, 27.68) | 0.97 | -0.24 (-10.62, 10.15) | 0.967 | 0.22 (-10.38, 10.83) | 0.97 |
| 446.6 | Polyarteritis nodosa | 257 | 1.35 (-28.74, 31.44) | 0.936 | -0.08 (-3.86, 3.7) | 0.97 | -4.88 (-31.77, 22.02) | 0.735 | 0.15 (-6.83, 7.13) | 0.97 | -0.6 (-28.75, 27.56) | 0.97 |
| 446.8 | Thrombotic microangiopathy | 144 | -0.1 (-4.97, 4.76) | 0.97 | 1.86 (-48.78, 52.5) | 0.948 | -0.12 (-5.56, 5.33) | 0.97 | -0.8 (-38.61, 37.01) | 0.97 | 3.69 (-28.46, 35.85) | 0.833 |
| 446.9 | Arteritis NOS | 234 | 0.04 (-2.06, 2.14) | 0.97 | 1.72 (-33.57, 37.01) | 0.93 | -1.73 (-83.52, 80.06) | 0.97 | -0.49 (-23.89, 22.9) | 0.97 | 0.1 (-4.81, 5.02) | 0.97 |
| 447 | Other disorders of arteries and arterioles | 742 | -0.1 (-4.91, 4.71) | 0.97 | -0.83 (-35.03, 33.37) | 0.966 | -0.76 (-36.9, 35.37) | 0.97 | 0.55 (-22.21, 23.31) | 0.966 | 0.48 (-22.41, 23.38) | 0.97 |
| 448 | Disease of capillaries | 1564 | -0.43 (-9.1, 8.23) | 0.929 | 0.34 (-15.89, 16.58) | 0.97 | 1.17 (-13.72, 16.06) | 0.887 | 0.1 (-4.4, 4.59) | 0.97 | 0.16 (-7.59, 7.92) | 0.97 |
| 450 | Noninfectious disorders of lymphatic channels | 3019 | 0.1 (-4.7, 4.91) | 0.97 | -0.04 (-1.9, 1.82) | 0.97 | 0.47 (-18.93, 19.87) | 0.966 | -0.17 (-8.14, 7.81) | 0.97 | -0.15 (-7.19, 6.89) | 0.97 |
| 451 | Phlebitis and thrombophlebitis | 16748 | **-0.58 (-1.02, -0.13)** | **0.011** | -0.24 (-5.71, 5.22) | 0.936 | -0.6 (-5.87, 4.66) | 0.833 | **0.91 (0.57, 1.25)** | **<0.001** | 0.01 (-0.33, 0.34) | 0.97 |
| 451.2 | Phlebitis and thrombophlebitis of lower extremities | 15650 | -0.53 (-1.08, 0.01) | 0.055 | -0.27 (-5.85, 5.31) | 0.93 | -0.7 (-6.35, 4.95) | 0.82 | **0.9 (0.55, 1.25)** | **<0.001** | 0.08 (-3.89, 4.06) | 0.97 |
| 452 | Other venous embolism and thrombosis | 4276 | 0.23 (-4.53, 5) | 0.931 | -0.33 (-7.33, 6.66) | 0.932 | -0.6 (-8.21, 7.01) | 0.887 | 0.06 (-2.78, 2.9) | 0.97 | -0.03 (-1.42, 1.36) | 0.97 |
| 452.8 | Postphlebitic syndrome | 341 | 0.69 (-14.88, 16.25) | 0.937 | -0.23 (-11.13, 10.67) | 0.97 | -1.98 (-23.02, 19.07) | 0.864 | 0.02 (-1.1, 1.15) | 0.97 | 0.02 (-0.74, 0.78) | 0.97 |
| 454 | Varicose veins | 16500 | -0.04 (-2.13, 2.04) | 0.97 | -0.39 (-8.31, 7.54) | 0.93 | -0.92 (-8.96, 7.11) | 0.833 | 0.37 (-2.89, 3.64) | 0.833 | 0.13 (-5.87, 6.12) | 0.97 |
| 454.1 | Varicose veins of lower extremity | 12511 | 0.14 (-6.49, 6.77) | 0.97 | -0.53 (-7.25, 6.19) | 0.887 | -0.65 (-14.07, 12.76) | 0.93 | 0.2 (-7.9, 8.29) | 0.966 | 0.36 (-7.1, 7.82) | 0.93 |
| 454.11 | Varicose veins of lower extremity, symptomtic | 3478 | -0.22 (-9.97, 9.52) | 0.967 | -0.04 (-2.16, 2.07) | 0.97 | -1.4 (-16.35, 13.54) | 0.864 | 0.48 (-5.67, 6.63) | 0.887 | -0.6 (-8.48, 7.27) | 0.89 |
| 455 | Hemorrhoids | 9001 | -0.05 (-2.36, 2.26) | 0.97 | 0.24 (-4.74, 5.23) | 0.93 | 0.17 (-7.65, 7.98) | 0.97 | -0.07 (-3.42, 3.28) | 0.97 | 0.07 (-3.38, 3.52) | 0.97 |
| 456 | Chronic venous insufficiency [CVI] | 925 | -0.19 (-9.03, 8.66) | 0.97 | -0.06 (-2.67, 2.56) | 0.97 | 0.11 (-4.98, 5.2) | 0.97 | 0.21 (-9.68, 10.09) | 0.97 | 0.79 (-9.19, 10.76) | 0.886 |
| 458 | Hypotension | 5822 | -0.13 (-5.38, 5.13) | 0.966 | 0.1 (-4.49, 4.68) | 0.97 | -0.61 (-8.65, 7.42) | 0.89 | 0.19 (-4.52, 4.9) | 0.942 | 0.34 (-4.04, 4.73) | 0.887 |
| 458.1 | Orthostatic hypotension | 2573 | -0.35 (-3.37, 2.68) | 0.833 | 0.08 (-3.88, 4.04) | 0.97 | -0.06 (-3.13, 3) | 0.97 | 0.33 (-3.14, 3.8) | 0.864 | -0.17 (-7.48, 7.15) | 0.967 |
| 458.2 | Iatrogenic hypotension | 250 | -0.86 (-11.84, 10.11) | 0.887 | 0.7 (-32.33, 33.72) | 0.97 | -0.95 (-45.97, 44.06) | 0.97 | 0.78 (-14.83, 16.38) | 0.929 | -0.61 (-27.05, 25.84) | 0.967 |
| 458.9 | Hypotension NOS | 3122 | 0.18 (-7.88, 8.24) | 0.968 | 0.05 (-2.37, 2.47) | 0.97 | -0.88 (-17.41, 15.64) | 0.923 | -0.05 (-2.55, 2.45) | 0.97 | 0.85 (-4.01, 5.72) | 0.744 |
| 459 | Other disorders of circulatory system | 2556 | 0.31 (-3.69, 4.3) | 0.89 | -0.05 (-2.57, 2.46) | 0.97 | -0.12 (-5.63, 5.4) | 0.97 | -0.28 (-6.04, 5.48) | 0.93 | 0.08 (-3.86, 4.02) | 0.97 |
| 459.1 | Hemorrhage NOS | 139 | 0.47 (-21.62, 22.55) | 0.97 | -1.76 (-41.72, 38.21) | 0.937 | -1.19 (-57.67, 55.28) | 0.97 | 0.31 (-14.46, 15.08) | 0.97 | 2.49 (-23.99, 28.97) | 0.864 |
| 459.9 | Circulatory disease NEC | 2175 | 0.23 (-5.91, 6.36) | 0.948 | -0.12 (-5.71, 5.47) | 0.97 | -0.26 (-12.71, 12.18) | 0.97 | -0.13 (-6.23, 5.97) | 0.97 | 0.11 (-4.93, 5.15) | 0.97 |
| 464 | Acute sinusitis | 5910 | -0.22 (-9.09, 8.66) | 0.966 | 0.54 (-10.56, 11.64) | 0.93 | 0.07 (-3.35, 3.49) | 0.97 | -0.04 (-1.73, 1.66) | 0.97 | -0.56 (-7.77, 6.64) | 0.887 |
| 465 | Acute upper respiratory infections of multiple or unspecified sites | 22046 | 0.03 (-1.34, 1.4) | 0.97 | 0.46 (-4.4, 5.31) | 0.864 | 0.76 (-7.33, 8.85) | 0.864 | -0.37 (-3.62, 2.88) | 0.833 | 0.64 (-2.06, 3.34) | 0.654 |
| 465.2 | Acute pharyngitis | 3654 | 0.5 (-10.8, 11.81) | 0.936 | -0.16 (-7.9, 7.58) | 0.97 | -0.07 (-3.55, 3.41) | 0.97 | -0.42 (-15.2, 14.37) | 0.96 | 0.75 (-14.73, 16.24) | 0.93 |
| 465.4 | Acute laryngitis and tracheitis | 1843 | -0.4 (-17.03, 16.23) | 0.966 | 0.81 (-27.82, 29.43) | 0.96 | 0.25 (-11.73, 12.24) | 0.97 | 0.03 (-1.61, 1.68) | 0.97 | 0.55 (-22.71, 23.82) | 0.966 |
| 470 | Septal Deviations/Turbinate Hypertrophy | 9416 | -0.04 (-1.99, 1.9) | 0.97 | 0.21 (-9.67, 10.09) | 0.97 | -0.58 (-13.6, 12.44) | 0.936 | 0.06 (-2.88, 3) | 0.97 | -0.12 (-5.95, 5.71) | 0.97 |
| 471 | Nasal polyps | 2471 | 0.33 (-13.35, 14.02) | 0.966 | -0.03 (-1.31, 1.25) | 0.97 | 1.52 (-17.8, 20.84) | 0.887 | -0.61 (-12.05, 10.82) | 0.923 | -2.17 (-4.38, 0.05) | 0.055 |
| 472 | Chronic pharyngitis and nasopharyngitis | 1553 | -0.01 (-0.4, 0.39) | 0.97 | -0.86 (-24.16, 22.45) | 0.948 | 0.35 (-16.08, 16.77) | 0.97 | 0.29 (-13.36, 13.93) | 0.97 | -0.72 (-20.01, 18.57) | 0.947 |
| 473 | Diseases of the larynx and vocal cords | 10109 | -0.01 (-0.48, 0.46) | 0.97 | 0.41 (-6.8, 7.61) | 0.919 | -0.04 (-1.98, 1.89) | 0.97 | -0.15 (-6.14, 5.85) | 0.966 | 0.55 (-3.87, 4.96) | 0.82 |
| 473.1 | Chronic laryngitis | 1934 | -0.63 (-11.02, 9.77) | 0.913 | 0.21 (-9.94, 10.37) | 0.97 | 1.32 (-25.77, 28.41) | 0.93 | 0.3 (-13.13, 13.74) | 0.968 | 1.06 (-10.18, 12.3) | 0.864 |
| 473.3 | Paralysis/spasm of vocal cords or larynx | 1581 | 0.25 (-11.46, 11.95) | 0.97 | 0.08 (-3.82, 3.99) | 0.97 | -1.03 (-26.36, 24.3) | 0.942 | -0.06 (-2.83, 2.71) | 0.97 | 0.19 (-8.85, 9.24) | 0.97 |
| 473.4 | Voice disturbance | 4719 | 0.28 (-5.52, 6.08) | 0.931 | 0.12 (-5.78, 6.02) | 0.97 | -0.26 (-12.37, 11.86) | 0.97 | -0.29 (-6.18, 5.6) | 0.93 | 0.61 (-4.72, 5.94) | 0.833 |
| 474 | Acute and chronic tonsillitis | 41428 | -0.29 (-1.45, 0.87) | 0.634 | 0.42 (-1.97, 2.8) | 0.744 | 0.38 (-5.71, 6.48) | 0.909 | 0.05 (-2.15, 2.24) | 0.97 | **0.67 (0.15, 1.19)** | **0.011** |
| 474.1 | Acute tonsillitis | 18162 | 0.1 (-4.42, 4.61) | 0.97 | 0.41 (-7.17, 7.98) | 0.923 | 0.75 (-7.23, 8.74) | 0.864 | -0.42 (-3.76, 2.93) | 0.82 | **1.34 (0.64, 2.04)** | **<0.001** |
| 474.2 | Chronic tonsillitis and adenoiditis | 27077 | -0.32 (-1.56, 0.93) | 0.634 | 0.19 (-5.57, 5.95) | 0.953 | 0.27 (-9.46, 10.01) | 0.96 | 0.19 (-2.28, 2.67) | 0.887 | 0.14 (-5.7, 5.98) | 0.966 |
| 475 | Chronic sinusitis | 5282 | -0.39 (-7.7, 6.92) | 0.923 | 0.23 (-10.87, 11.34) | 0.97 | 0.61 (-24.67, 25.9) | 0.966 | 0.19 (-8.62, 8.99) | 0.97 | -0.5 (-10.77, 9.77) | 0.93 |
| 476 | Allergic rhinitis | 7408 | -0.38 (-4.44, 3.67) | 0.864 | 0.09 (-4.11, 4.29) | 0.97 | 0.94 (-7.63, 9.52) | 0.84 | 0.16 (-6.74, 7.06) | 0.966 | -0.06 (-2.83, 2.71) | 0.97 |
| 477 | Epistaxis or throat hemorrhage | 12337 | 0.08 (-3.52, 3.67) | 0.97 | 0.02 (-0.73, 0.76) | 0.97 | -0.15 (-7.46, 7.15) | 0.97 | -0.06 (-2.69, 2.58) | 0.97 | -0.04 (-1.82, 1.74) | 0.97 |
| 478 | Throat pain | 576 | 0.28 (-12.97, 13.53) | 0.97 | -0.67 (-18.95, 17.61) | 0.948 | -0.03 (-1.61, 1.55) | 0.97 | 0.04 (-1.92, 2) | 0.97 | 0.23 (-10.74, 11.21) | 0.97 |
| 479 | Other upper respiratory disease | 3940 | -0.78 (-7.59, 6.02) | 0.833 | 1.01 (-9.77, 11.8) | 0.864 | 0.61 (-27.98, 29.2) | 0.97 | 0.23 (-10.82, 11.29) | 0.97 | -0.01 (-0.5, 0.48) | 0.97 |
| 480 | Pneumonia | 76292 | -0.14 (-1.34, 1.06) | 0.833 | 0.17 (-1.96, 2.29) | 0.887 | 0.08 (-3.62, 3.77) | 0.97 | 0.06 (-2.29, 2.4) | 0.966 | 0.23 (-0.91, 1.37) | 0.705 |
| 480.1 | Bacterial pneumonia | 20715 | 0.11 (-2.1, 2.32) | 0.93 | -0.05 (-2.39, 2.29) | 0.97 | 0.02 (-0.71, 0.74) | 0.97 | -0.09 (-2.6, 2.42) | 0.948 | 0.16 (-2.86, 3.18) | 0.923 |
| 480.11 | Pneumococcal pneumonia | 2318 | -0.4 (-11.24, 10.44) | 0.948 | 0 (-0.04, 0.04) | 0.97 | 0.79 (-27.82, 29.4) | 0.961 | 0.25 (-10.84, 11.34) | 0.968 | 0.03 (-1.41, 1.48) | 0.97 |
| 480.12 | Pseudomonal pneumonia | 552 | 0.21 (-9.76, 10.18) | 0.97 | 1.03 (-11.68, 13.74) | 0.883 | -0.67 (-32.41, 31.07) | 0.97 | -0.53 (-11.45, 10.38) | 0.93 | 0.02 (-0.93, 0.97) | 0.97 |
| 480.2 | Viral pneumonia | 2172 | 0.78 (-6.04, 7.61) | 0.833 | -0.32 (-15.68, 15.04) | 0.97 | 0.18 (-8.14, 8.49) | 0.97 | -0.7 (-8.1, 6.71) | 0.864 | 0.46 (-18.59, 19.52) | 0.966 |
| 480.3 | Pneumonia due to fungus (mycoses) | 274 | -0.35 (-16.8, 16.1) | 0.97 | 1.44 (-16.89, 19.77) | 0.887 | -0.36 (-17.36, 16.64) | 0.97 | -0.2 (-9.51, 9.12) | 0.97 | -0.67 (-28.27, 26.93) | 0.966 |
| 480.5 | Bronchopneumonia and lung abscess | 2934 | 0.29 (-13.26, 13.83) | 0.97 | 0.69 (-23.74, 25.13) | 0.96 | -0.52 (-25.07, 24.03) | 0.97 | -0.48 (-13.54, 12.58) | 0.948 | 0.13 (-6.1, 6.36) | 0.97 |
| 481 | Influenza | 4871 | -0.18 (-7.41, 7.06) | 0.966 | -0.15 (-7.16, 6.87) | 0.97 | 0.11 (-4.97, 5.19) | 0.97 | 0.22 (-7.77, 8.21) | 0.961 | -0.36 (-10.26, 9.53) | 0.948 |
| 483 | Acute bronchitis and bronchiolitis | 15144 | -0.28 (-3.25, 2.69) | 0.864 | 0.35 (-4.07, 4.76) | 0.887 | 0.09 (-4.2, 4.38) | 0.97 | 0.12 (-4.89, 5.13) | 0.966 | -0.03 (-1.48, 1.42) | 0.97 |
| 495 | Asthma | 31106 | -0.26 (-2.31, 1.8) | 0.82 | -0.15 (-6.38, 6.07) | 0.966 | 0.57 (-4.36, 5.5) | 0.833 | 0.22 (-2.12, 2.56) | 0.864 | 0.17 (-4.48, 4.82) | 0.948 |
| 496 | Chronic airway obstruction | 30341 | 0.08 (-2.11, 2.27) | 0.948 | 0.05 (-2.2, 2.29) | 0.97 | -0.12 (-5.87, 5.63) | 0.97 | -0.08 (-2.25, 2.09) | 0.948 | -0.09 (-3.63, 3.45) | 0.966 |
| 496.1 | Emphysema | 2505 | 0.33 (-13.02, 13.67) | 0.965 | -0.46 (-19.53, 18.6) | 0.966 | -1.39 (-19.13, 16.34) | 0.887 | 0.07 (-3.29, 3.43) | 0.97 | -0.33 (-14.95, 14.29) | 0.968 |
| 496.2 | Chronic bronchitis | 681 | -0.42 (-20.05, 19.22) | 0.97 | 1.59 (-31.27, 34.45) | 0.931 | 3.96 (-27.98, 35.91) | 0.82 | -1.02 (-21.85, 19.82) | 0.93 | 0.61 (-28.17, 29.39) | 0.97 |
| 496.21 | Obstructive chronic bronchitis | 12484 | -0.09 (-3.64, 3.47) | 0.965 | 0.41 (-1.2, 2.02) | 0.634 | -0.29 (-6.27, 5.69) | 0.93 | -0.01 (-0.46, 0.44) | 0.97 | -0.1 (-4.33, 4.13) | 0.966 |
| 499 | Cystic fibrosis | 231 | -1.37 (-50.03, 47.28) | 0.96 | 1.89 (-80.66, 84.44) | 0.967 | 9.79 (-94.35, 113.93) | 0.864 | 0.11 (-5.15, 5.37) | 0.97 | -2.37 (-55.3, 50.55) | 0.936 |
| 500 | Lung disease due to external agents | 1287 | 0.14 (-6.71, 7) | 0.97 | 0.08 (-3.5, 3.65) | 0.97 | 1.13 (-24.17, 26.44) | 0.936 | -0.34 (-13.38, 12.7) | 0.963 | 0.37 (-15.78, 16.52) | 0.967 |
| 500.1 | Extrinsic allergic alveolitis | 202 | -1.21 (-26.06, 23.64) | 0.93 | 2.51 (-29.42, 34.44) | 0.887 | 2.3 (-60.18, 64.77) | 0.948 | -0.04 (-1.79, 1.72) | 0.97 | -0.75 (-36.4, 34.89) | 0.97 |
| 501 | Pneumonitis due to inhalation of food or vomitus | 2415 | 0.32 (-2.49, 3.13) | 0.833 | -0.11 (-5.42, 5.2) | 0.97 | 0.44 (-11.46, 12.34) | 0.948 | -0.36 (-3.22, 2.51) | 0.82 | 0.71 (-0.46, 1.87) | 0.236 |
| 502 | Postinflammatory pulmonary fibrosis | 1537 | 0.14 (-6.36, 6.64) | 0.97 | -0.01 (-0.32, 0.31) | 0.97 | 1.39 (-30.79, 33.57) | 0.938 | -0.35 (-15.26, 14.55) | 0.966 | 0.26 (-12.11, 12.63) | 0.97 |
| 503 | Pulmonary congestion and hypostasis | 143 | -0.33 (-15.84, 15.19) | 0.97 | 0.53 (-24.44, 25.5) | 0.97 | -2.73 (-64.78, 59.32) | 0.937 | 0.72 (-33.47, 34.91) | 0.97 | -0.47 (-22.58, 21.65) | 0.97 |
| 504 | Other alveolar and parietoalveolar pneumonopathy | 2678 | 0.18 (-7.42, 7.78) | 0.966 | -0.34 (-9.47, 8.8) | 0.948 | 0.45 (-17.95, 18.85) | 0.966 | -0.14 (-6.67, 6.39) | 0.97 | 0 (-0.08, 0.07) | 0.97 |
| 506 | Empyema and pneumothorax | 5852 | 0.09 (-4.35, 4.53) | 0.97 | 0.31 (-13.16, 13.78) | 0.967 | 1.14 (-10.97, 13.25) | 0.864 | -0.41 (-5.69, 4.86) | 0.887 | -0.01 (-0.49, 0.47) | 0.97 |
| 507 | Pleurisy; pleural effusion | 4582 | -0.06 (-2.7, 2.58) | 0.97 | 0.1 (-4.64, 4.84) | 0.97 | 1.23 (-9.46, 11.91) | 0.833 | -0.19 (-8.12, 7.74) | 0.966 | -0.18 (-8.81, 8.45) | 0.97 |
| 508 | Pulmonary collapse; interstitial and compensatory emphysema | 2022 | 0.03 (-1.55, 1.61) | 0.97 | -0.61 (-23.48, 22.27) | 0.962 | -0.57 (-27.37, 26.23) | 0.97 | 0.31 (-12.86, 13.49) | 0.966 | 0.19 (-8.69, 9.06) | 0.97 |
| 509 | Respiratory failure, insufficiency, arrest | 26026 | 0.01 (-0.67, 0.7) | 0.97 | 0.01 (-0.35, 0.37) | 0.97 | 0.03 (-1.46, 1.53) | 0.97 | -0.02 (-1.13, 1.08) | 0.97 | 0.03 (-1.32, 1.37) | 0.97 |
| 509.1 | Respiratory failure | 23954 | 0.02 (-1.14, 1.19) | 0.97 | -0.02 (-0.76, 0.73) | 0.97 | 0.03 (-1.43, 1.49) | 0.97 | -0.02 (-1.16, 1.11) | 0.97 | 0.01 (-0.32, 0.33) | 0.97 |
| 509.2 | Respiratory insufficiency | 12550 | 0.02 (-1.09, 1.14) | 0.97 | -0.21 (-2.03, 1.61) | 0.833 | -0.08 (-3.98, 3.81) | 0.97 | 0.07 (-1.93, 2.08) | 0.948 | 0.06 (-2.71, 2.83) | 0.97 |
| 509.3 | Pulmonary insufficiency or respiratory failure following trauma and surgery | 268 | 0.09 (-4.13, 4.31) | 0.97 | 1.49 (-14.31, 17.28) | 0.864 | -1.44 (-60.65, 57.78) | 0.966 | -0.56 (-15.77, 14.65) | 0.948 | -0.04 (-1.93, 1.85) | 0.97 |
| 509.5 | Respiratory arrest | 1258 | -0.15 (-6.17, 5.87) | 0.966 | -0.27 (-11.18, 10.64) | 0.965 | 0.15 (-7.09, 7.39) | 0.97 | 0.23 (-4.43, 4.88) | 0.931 | 0.21 (-8.55, 8.97) | 0.966 |
| 509.8 | Dependence on respirator [Ventilator] or supplemental oxygen | 2293 | -0.2 (-4.37, 3.96) | 0.93 | -0.01 (-0.63, 0.6) | 0.97 | -0.21 (-10.08, 9.66) | 0.97 | 0.25 (-2.88, 3.38) | 0.886 | 0.09 (-4.01, 4.18) | 0.97 |
| 510 | Other diseases of lung | 1028 | -0.25 (-12.27, 11.76) | 0.97 | -0.43 (-19.3, 18.44) | 0.968 | -0.71 (-34.35, 32.93) | 0.97 | 0.51 (-9.95, 10.97) | 0.93 | 0.51 (-19.1, 20.12) | 0.963 |
| 510.2 | Lung transplant | 246 | -0.11 (-5.43, 5.21) | 0.97 | -1.21 (-25.97, 23.55) | 0.93 | -0.6 (-28.89, 27.7) | 0.97 | 0.54 (-14.09, 15.16) | 0.948 | 0.41 (-18.84, 19.65) | 0.97 |
| 512 | Other symptoms of respiratory system | 32402 | -0.02 (-0.91, 0.87) | 0.97 | 0.04 (-2.03, 2.12) | 0.97 | -0.02 (-1.03, 0.99) | 0.97 | 0 (-0.22, 0.23) | 0.97 | 0 (-0.2, 0.19) | 0.97 |
| 512.2 | Painful respiration | 1707 | 0.08 (-3.8, 3.96) | 0.97 | -0.08 (-4.08, 3.91) | 0.97 | 0.49 (-10.59, 11.56) | 0.937 | -0.15 (-6.33, 6.03) | 0.966 | 0.11 (-5.08, 5.3) | 0.97 |
| 512.7 | Shortness of breath | 30319 | -0.04 (-1.8, 1.72) | 0.968 | 0.04 (-1.67, 1.74) | 0.97 | -0.09 (-4.37, 4.19) | 0.97 | 0.04 (-1.79, 1.87) | 0.967 | 0.02 (-0.79, 0.82) | 0.97 |
| 512.8 | Cough | 145 | 0.41 (-19.16, 19.99) | 0.97 | 0.58 (-26.97, 28.14) | 0.97 | -2.61 (-110.18, 104.96) | 0.966 | -0.51 (-24.86, 23.83) | 0.97 | -0.67 (-32.27, 30.94) | 0.97 |
| 512.9 | Other dyspnea | 676 | 0.01 (-0.41, 0.42) | 0.97 | 0.54 (-24.86, 25.94) | 0.97 | 0.55 (-25.37, 26.46) | 0.97 | -0.35 (-16.66, 15.97) | 0.97 | -0.54 (-22.95, 21.87) | 0.966 |
| 513 | Respiratory abnormalities | 5945 | 0.04 (-1.74, 1.81) | 0.97 | 0.22 (-9.95, 10.38) | 0.97 | 0.7 (-14.97, 16.37) | 0.936 | -0.27 (-7.5, 6.97) | 0.948 | 0.02 (-1.11, 1.16) | 0.97 |
| 513.3 | Hypoventilation | 545 | 0 (-0.09, 0.1) | 0.97 | -0.32 (-15.4, 14.76) | 0.97 | 2.69 (-70.61, 76) | 0.948 | -0.33 (-15.79, 15.13) | 0.97 | -1.98 (-27.19, 23.22) | 0.887 |
| 513.4 | Hyperventilation | 5060 | 0.16 (-7.19, 7.5) | 0.97 | 0.42 (-9.12, 9.95) | 0.938 | 0.41 (-17.39, 18.21) | 0.967 | -0.42 (-4.91, 4.07) | 0.864 | 0.23 (-9.79, 10.25) | 0.967 |
| 513.8 | Disorders of diaphragm | 261 | -0.38 (-18.59, 17.82) | 0.97 | 0.1 (-4.6, 4.8) | 0.97 | -0.18 (-8.76, 8.4) | 0.97 | 0.39 (-18.06, 18.84) | 0.97 | -0.9 (-43.56, 41.76) | 0.97 |
| 514 | Abnormal findings examination of lungs | 500 | -1.06 (-12.3, 10.18) | 0.864 | -1.4 (-29.46, 26.67) | 0.929 | 1.45 (-38.07, 40.98) | 0.948 | 1.33 (-10.22, 12.87) | 0.833 | -0.69 (-29.27, 27.88) | 0.966 |
| 514.1 | Abnormal results of function study of pulmonary system | 499 | -1.05 (-12.27, 10.16) | 0.864 | -1.4 (-29.51, 26.72) | 0.929 | 1.45 (-38.03, 40.93) | 0.948 | 1.32 (-10.2, 12.85) | 0.833 | -0.7 (-29.45, 28.06) | 0.966 |
| 516 | Abnormal sputum | 4111 | -0.19 (-8.19, 7.8) | 0.966 | 0.3 (-11.92, 12.52) | 0.966 | -0.76 (-16.33, 14.81) | 0.93 | 0.2 (-8.09, 8.49) | 0.966 | -0.15 (-7.43, 7.12) | 0.97 |
| 516.1 | Hemoptysis | 4034 | -0.17 (-8.17, 7.83) | 0.97 | 0.34 (-13.69, 14.37) | 0.966 | -0.67 (-19.04, 17.69) | 0.948 | 0.14 (-6.53, 6.81) | 0.97 | -0.13 (-6.37, 6.11) | 0.97 |
| 519 | Other diseases of respiratory system, not elsewhere classified | 3368 | -0.29 (-8.04, 7.47) | 0.948 | 0.52 (-10.07, 11.11) | 0.93 | 1.07 (-12.89, 15.02) | 0.89 | -0.08 (-3.98, 3.82) | 0.97 | -0.03 (-1.24, 1.18) | 0.97 |
| 519.2 | Respiratory complications | 655 | 0.22 (-8.81, 9.25) | 0.966 | 0.1 (-4.69, 4.89) | 0.97 | -0.63 (-17.81, 16.55) | 0.948 | -0.14 (-6.59, 6.32) | 0.97 | -0.39 (-11.12, 10.33) | 0.948 |
| 519.8 | Other diseases of respiratory system, NEC | 973 | -0.19 (-8.69, 8.3) | 0.967 | 0.43 (-11.2, 12.05) | 0.948 | -0.46 (-22.03, 21.12) | 0.97 | 0.07 (-3.22, 3.36) | 0.97 | -0.03 (-1.51, 1.44) | 0.97 |
| 519.9 | Symptoms involving respiratory system and other chest symptoms | 497 | 0.42 (-19.45, 20.29) | 0.97 | 1.79 (-17.29, 20.88) | 0.864 | 2.25 (-58.97, 63.47) | 0.948 | -1.48 (-13.41, 10.45) | 0.82 | 0.16 (-7.26, 7.57) | 0.97 |
| 520 | Disorders of tooth development | 4317 | -0.23 (-8.41, 7.95) | 0.96 | -0.55 (-8.52, 7.41) | 0.9 | 0.28 (-13.09, 13.66) | 0.97 | 0.42 (-4.01, 4.84) | 0.864 | -0.41 (-8.83, 8.01) | 0.93 |
| 520.2 | Disturbances in tooth eruption | 3002 | -0.27 (-11.39, 10.85) | 0.966 | -0.34 (-14.59, 13.91) | 0.966 | 0.05 (-2.11, 2.2) | 0.97 | 0.41 (-7.3, 8.13) | 0.923 | -0.68 (-7.89, 6.53) | 0.864 |
| 521 | Diseases of hard tissues of teeth | 4146 | 0.13 (-6, 6.26) | 0.97 | 0.1 (-4.67, 4.87) | 0.97 | -0.04 (-1.94, 1.86) | 0.97 | -0.17 (-8.17, 7.83) | 0.97 | -0.34 (-14.52, 13.83) | 0.966 |
| 521.1 | Dental caries | 3671 | 0.01 (-0.36, 0.37) | 0.97 | 0.2 (-9.39, 9.79) | 0.97 | 0.33 (-15.33, 15.99) | 0.97 | -0.14 (-6.81, 6.52) | 0.97 | -0.19 (-9.41, 9.02) | 0.97 |
| 521.4 | Tooth complications likely association with other diseases | 304 | -0.16 (-7.54, 7.23) | 0.97 | -0.63 (-30.26, 29) | 0.97 | -1.64 (-69.21, 65.93) | 0.966 | 0.84 (-33.31, 35) | 0.965 | -0.67 (-32.15, 30.82) | 0.97 |
| 522 | Diseases of pulp and periapical tissues | 1504 | 0.32 (-14.94, 15.59) | 0.97 | -0.42 (-20.15, 19.32) | 0.97 | 0.96 (-40.92, 42.84) | 0.967 | -0.31 (-14.95, 14.33) | 0.97 | -1.44 (-14, 11.11) | 0.833 |
| 522.1 | Pulpitis and necrosis of tooth pulp | 430 | 0.51 (-23.67, 24.69) | 0.97 | -2.96 (-28.76, 22.83) | 0.833 | 1.18 (-54.8, 57.17) | 0.97 | 0.65 (-29.97, 31.27) | 0.97 | -2.21 (-25.73, 21.3) | 0.864 |
| 522.5 | Periapical abscess | 702 | -0.64 (-17.98, 16.7) | 0.948 | 1.96 (-15.11, 19.03) | 0.833 | -0.29 (-13.86, 13.29) | 0.97 | -0.11 (-5.17, 4.96) | 0.97 | -2.06 (-10.22, 6.1) | 0.634 |
| 523 | Gingival and periodontal diseases | 8184 | 0.25 (-2.99, 3.49) | 0.89 | -0.01 (-0.59, 0.56) | 0.97 | -0.08 (-3.96, 3.8) | 0.97 | -0.23 (-4.57, 4.11) | 0.923 | -0.28 (-6.11, 5.56) | 0.932 |
| 523.1 | Gingivitis | 552 | 0.13 (-5.89, 6.14) | 0.97 | -0.36 (-17.2, 16.48) | 0.97 | -0.43 (-20.86, 20) | 0.97 | 0.09 (-4.36, 4.55) | 0.97 | -1.09 (-42.6, 40.42) | 0.963 |
| 523.3 | Periodontitis (acute or chronic) | 138 | -0.1 (-4.9, 4.7) | 0.97 | -1.08 (-52.08, 49.93) | 0.97 | -6.86 (-150.72, 136.99) | 0.932 | 1.25 (-58.01, 60.52) | 0.97 | -0.37 (-17.9, 17.16) | 0.97 |
| 523.31 | Acute periodontitis | 736 | -0.34 (-16.61, 15.92) | 0.97 | -0.12 (-5.94, 5.7) | 0.97 | -1.86 (-52.56, 48.83) | 0.948 | 0.67 (-24.25, 25.59) | 0.962 | -0.79 (-33.45, 31.86) | 0.966 |
| 523.32 | Chronic periodontitis | 6591 | 0.24 (-3.27, 3.76) | 0.9 | 0.01 (-0.56, 0.58) | 0.97 | -0.13 (-6.11, 5.86) | 0.97 | -0.23 (-4.56, 4.1) | 0.923 | -0.26 (-7.02, 6.5) | 0.945 |
| 524 | Dentofacial anomalies, including malocclusion | 3212 | -0.1 (-4.84, 4.64) | 0.97 | -0.38 (-10.76, 10) | 0.948 | -0.53 (-22.39, 21.33) | 0.966 | 0.35 (-5.34, 6.05) | 0.911 | -0.22 (-10.61, 10.17) | 0.97 |
| 524.3 | Anomalies of tooth position/malocclusion | 2606 | 0.19 (-8.11, 8.49) | 0.967 | -0.61 (-8.38, 7.16) | 0.887 | -0.98 (-13.38, 11.43) | 0.887 | 0.24 (-9.84, 10.33) | 0.966 | 0.01 (-0.43, 0.45) | 0.97 |
| 525 | Other diseases of the teeth and supporting structures | 6712 | -0.02 (-0.92, 0.88) | 0.97 | 0.45 (-8.01, 8.91) | 0.923 | 0.37 (-16.56, 17.3) | 0.969 | -0.23 (-6.37, 5.92) | 0.948 | -0.06 (-3.02, 2.9) | 0.97 |
| 525.2 | Atrophy of edentulous alveolar ridge | 807 | -0.06 (-2.89, 2.77) | 0.97 | 0.71 (-28.54, 29.96) | 0.966 | -2.41 (-28.1, 23.27) | 0.864 | 0.1 (-4.86, 5.07) | 0.97 | -0.02 (-0.73, 0.7) | 0.97 |
| 526 | Diseases of the jaws | 6679 | -0.13 (-6.1, 5.84) | 0.97 | 0.19 (-8.58, 8.95) | 0.97 | 0.54 (-11.64, 12.72) | 0.937 | -0.04 (-2.01, 1.93) | 0.97 | 0.24 (-9.17, 9.66) | 0.963 |
| 526.1 | Cysts of the jaws | 792 | 0.5 (-20.19, 21.19) | 0.966 | -1.08 (-30.61, 28.44) | 0.948 | 1.14 (-52.9, 55.19) | 0.97 | -0.18 (-8.91, 8.54) | 0.97 | -0.04 (-2.04, 1.96) | 0.97 |
| 526.3 | Anomalies of jaw size/symmetry | 2416 | 0.3 (-7.75, 8.34) | 0.948 | 0.21 (-9.61, 10.02) | 0.97 | -0.48 (-21.23, 20.28) | 0.967 | -0.3 (-8.53, 7.92) | 0.948 | 0.36 (-14.37, 15.09) | 0.966 |
| 526.41 | Temporomandibular joint disorder, unspecified | 2363 | -0.38 (-7.49, 6.73) | 0.923 | 0.96 (-6.81, 8.74) | 0.82 | 1.11 (-12.56, 14.77) | 0.883 | -0.19 (-8.67, 8.29) | 0.968 | 0.47 (-9.91, 10.84) | 0.936 |
| 526.5 | Inflammatory conditions of jaw | 1059 | -0.78 (-9.12, 7.55) | 0.864 | 0.66 (-25.9, 27.21) | 0.965 | 1.06 (-27.82, 29.94) | 0.948 | 0.37 (-14.94, 15.69) | 0.966 | -0.3 (-14.28, 13.69) | 0.97 |
| 526.9 | Jaw disease NOS | 504 | -0.8 (-17.56, 15.96) | 0.932 | 0.71 (-30.07, 31.48) | 0.967 | -0.03 (-1.35, 1.29) | 0.97 | 0.48 (-20.63, 21.6) | 0.967 | -0.59 (-28.55, 27.37) | 0.97 |
| 527 | Diseases of the salivary glands | 2632 | 0.1 (-4.62, 4.82) | 0.97 | 0.35 (-16.24, 16.94) | 0.97 | 0.32 (-14.86, 15.5) | 0.97 | -0.3 (-12.85, 12.24) | 0.966 | 0.27 (-12.54, 13.08) | 0.97 |
| 527.1 | Hypertrophy of salivary gland | 189 | -2.1 (-23.56, 19.35) | 0.858 | 1.59 (-62.37, 65.55) | 0.965 | 2.62 (-68.82, 74.07) | 0.948 | 0.99 (-39.68, 41.66) | 0.966 | -0.51 (-24.39, 23.38) | 0.97 |
| 527.2 | Sialoadenitis | 1794 | 0.18 (-8.49, 8.86) | 0.97 | 0.6 (-24.18, 25.39) | 0.966 | -1.19 (-33.6, 31.22) | 0.948 | -0.22 (-10.8, 10.36) | 0.97 | -0.58 (-24.35, 23.19) | 0.966 |
| 527.7 | Disturbance of salivary secretion | 501 | 0.79 (-15.05, 16.63) | 0.929 | 0.56 (-25.96, 27.08) | 0.97 | 0.87 (-37.18, 38.92) | 0.967 | -1.2 (-11.67, 9.27) | 0.833 | 1.9 (-7.5, 11.31) | 0.705 |
| 527.8 | Other specified diseases of the salivary glands | 111 | 0.44 (-20.32, 21.19) | 0.97 | -1.3 (-62.76, 60.16) | 0.97 | 1.92 (-88.77, 92.6) | 0.97 | -0.46 (-22.3, 21.37) | 0.97 | 3.18 (-62.06, 68.41) | 0.93 |
| 528 | Diseases of the oral soft tissues, excluding lesions specific for gingiva and tongue | 6874 | -0.08 (-3.65, 3.49) | 0.97 | -0.43 (-10.1, 9.25) | 0.937 | -0.57 (-15.96, 14.82) | 0.948 | 0.35 (-4.19, 4.89) | 0.89 | -0.63 (-6.9, 5.65) | 0.856 |
| 528.11 | Stomatitis and mucositis (ulcerative) | 734 | -0.21 (-10.07, 9.65) | 0.97 | -3.01 (-27.23, 21.22) | 0.82 | 1.5 (-61.67, 64.68) | 0.966 | 1.2 (-21.28, 23.68) | 0.923 | -0.84 (-40.1, 38.42) | 0.97 |
| 528.12 | Oral aphthae | 695 | 0.37 (-16.98, 17.72) | 0.97 | -1.01 (-42.61, 40.59) | 0.966 | -0.52 (-25.34, 24.29) | 0.97 | 0.11 (-4.96, 5.18) | 0.97 | -1.76 (-26.11, 22.6) | 0.896 |
| 528.3 | Cellulitis and abscess of oral soft tissues | 1450 | 0.03 (-1.36, 1.42) | 0.97 | -0.07 (-3.24, 3.1) | 0.97 | -0.81 (-34.08, 32.46) | 0.966 | 0.15 (-6.92, 7.22) | 0.97 | -0.7 (-19.25, 17.85) | 0.946 |
| 528.41 | Cyst of the salivary gland | 379 | -0.47 (-22.47, 21.54) | 0.97 | -2.58 (-30.06, 24.89) | 0.864 | -2.68 (-75.54, 70.18) | 0.948 | 2.11 (-16.24, 20.46) | 0.833 | 0.46 (-21.13, 22.04) | 0.97 |
| 528.5 | Diseases of lips | 234 | -0.52 (-25.02, 23.99) | 0.97 | -1.69 (-71.28, 67.9) | 0.966 | -2.8 (-118.34, 112.73) | 0.966 | 1.51 (-29.41, 32.42) | 0.93 | -3.23 (-37.57, 31.12) | 0.864 |
| 528.6 | Leukoplakia of oral mucosa | 1241 | 0.92 (-6.52, 8.37) | 0.82 | -1.12 (-13, 10.77) | 0.864 | -1.24 (-29.7, 27.21) | 0.938 | -0.3 (-13.53, 12.93) | 0.968 | 0.32 (-14.96, 15.61) | 0.97 |
| 528.7 | Sialolithiasis | 1275 | -0.5 (-20.98, 19.98) | 0.966 | 1.12 (-22.28, 24.51) | 0.932 | 0.76 (-35.09, 36.61) | 0.97 | -0.06 (-3.09, 2.96) | 0.97 | -0.99 (-21.77, 19.79) | 0.932 |
| 529 | Diseases and other conditions of the tongue | 932 | 0.46 (-18.49, 19.41) | 0.966 | -0.98 (-23.05, 21.09) | 0.936 | -0.11 (-5.33, 5.11) | 0.97 | -0.01 (-0.69, 0.66) | 0.97 | 1.25 (-12, 14.49) | 0.864 |
| 529.1 | Glossitis | 577 | -0.13 (-6.24, 5.98) | 0.97 | -1.42 (-20.43, 17.6) | 0.893 | -0.09 (-4.3, 4.12) | 0.97 | 0.77 (-15.54, 17.08) | 0.932 | 0.84 (-22.11, 23.8) | 0.948 |
| 529.6 | Glossodynia | 117 | 1.67 (-33.31, 36.64) | 0.932 | 1.48 (-63.05, 66) | 0.967 | -4.64 (-130.93, 121.65) | 0.948 | -1.85 (-36.51, 32.8) | 0.923 | 2.61 (-46.1, 51.31) | 0.923 |
| 530 | Diseases of esophagus | 17864 | -0.28 (-1.39, 0.83) | 0.634 | 0.03 (-1.3, 1.35) | 0.97 | 0.13 (-5.95, 6.21) | 0.97 | 0.25 (-1.75, 2.24) | 0.82 | 0.07 (-3.47, 3.62) | 0.97 |
| 530.11 | GERD | 7466 | -0.17 (-3.71, 3.38) | 0.932 | 0.2 (-6.07, 6.47) | 0.955 | -0.19 (-9.38, 8.99) | 0.97 | 0.12 (-4.65, 4.88) | 0.966 | -0.19 (-6.91, 6.54) | 0.961 |
| 530.12 | Ulcer of esophagus | 607 | -0.17 (-8.08, 7.74) | 0.97 | 0.16 (-7.24, 7.55) | 0.97 | 0.63 (-29.33, 30.6) | 0.97 | 0 (-0.15, 0.15) | 0.97 | 0.41 (-18.85, 19.66) | 0.97 |
| 530.14 | Reflux esophagitis | 6431 | -0.01 (-0.36, 0.35) | 0.97 | -0.12 (-5.75, 5.51) | 0.97 | 0.05 (-2.52, 2.62) | 0.97 | 0.05 (-2.23, 2.33) | 0.97 | -0.21 (-6.05, 5.62) | 0.948 |
| 530.2 | Esophageal bleeding (varices/hemorrhage) | 1946 | -0.31 (-11.46, 10.84) | 0.961 | -0.86 (-10.15, 8.43) | 0.867 | 0.31 (-14.16, 14.77) | 0.97 | 0.59 (-5.67, 6.84) | 0.864 | -0.2 (-9.68, 9.27) | 0.97 |
| 530.3 | Stricture and stenosis of esophagus | 1799 | -0.46 (-9.12, 8.19) | 0.923 | -0.34 (-16.46, 15.78) | 0.97 | 0.74 (-29.76, 31.24) | 0.966 | 0.46 (-8.1, 9.02) | 0.923 | -0.25 (-11.88, 11.39) | 0.97 |
| 530.5 | Disorders of esophageal motility | 688 | -0.48 (-23.35, 22.39) | 0.97 | -1.74 (-23.8, 20.31) | 0.886 | 3.11 (-23.98, 30.2) | 0.833 | 0.55 (-22.64, 23.75) | 0.966 | 0.97 (-25.31, 27.24) | 0.948 |
| 530.6 | Diverticulum of esophagus, acquired | 279 | -0.42 (-20.37, 19.53) | 0.97 | -4.26 (-21.13, 12.61) | 0.634 | 1.61 (-68.62, 71.83) | 0.967 | 1.66 (-17.83, 21.16) | 0.877 | 0.83 (-38.48, 40.14) | 0.97 |
| 530.7 | Gastroesophageal laceration-hemorrhage syndrome | 597 | -0.94 (-26.52, 24.64) | 0.948 | -0.22 (-10.5, 10.06) | 0.97 | 3.71 (-74.05, 81.47) | 0.932 | 0.75 (-30.12, 31.62) | 0.966 | 0.2 (-9.12, 9.51) | 0.97 |
| 531 | Peptic ulcer (excl. esophageal) | 16679 | 0.28 (-3.2, 3.76) | 0.884 | 0.16 (-7.37, 7.69) | 0.97 | 0.35 (-14.18, 14.88) | 0.966 | -0.39 (-3.57, 2.78) | 0.82 | -0.18 (-7.68, 7.33) | 0.966 |
| 531.1 | Hemorrhage from gastrointestinal ulcer | 6278 | -0.13 (-6.05, 5.8) | 0.97 | 0.34 (-13.49, 14.16) | 0.966 | 0.54 (-19.09, 20.18) | 0.961 | -0.08 (-3.99, 3.82) | 0.97 | 0.32 (-8.49, 9.14) | 0.948 |
| 531.2 | Gastric ulcer | 6745 | 0.61 (-4.33, 5.56) | 0.82 | 0.18 (-8.31, 8.67) | 0.97 | 0.25 (-11.6, 12.1) | 0.97 | -0.72 (-3.56, 2.13) | 0.634 | 0.05 (-2.17, 2.26) | 0.97 |
| 531.3 | Duodenal ulcer | 4534 | 0.21 (-9.77, 10.2) | 0.97 | 0.3 (-13.96, 14.57) | 0.97 | -0.39 (-18.99, 18.2) | 0.97 | -0.26 (-11.83, 11.3) | 0.967 | -0.72 (-12.78, 11.34) | 0.915 |
| 531.4 | Peptic ulcer, site unspecified | 1743 | -0.09 (-4.29, 4.11) | 0.97 | -0.18 (-8.87, 8.5) | 0.97 | 1.22 (-14.29, 16.73) | 0.887 | -0.03 (-1.22, 1.17) | 0.97 | -0.46 (-12.89, 11.98) | 0.948 |
| 531.5 | Gastrojejunal ulcer | 586 | 0.02 (-0.76, 0.79) | 0.97 | 0.22 (-10.09, 10.52) | 0.97 | 0.76 (-32.35, 33.87) | 0.967 | -0.28 (-13.3, 12.75) | 0.97 | 0.89 (-10.48, 12.27) | 0.887 |
| 535 | Gastritis and duodenitis | 17792 | 0.14 (-5.49, 5.78) | 0.964 | -0.07 (-3.32, 3.18) | 0.97 | 0.01 (-0.66, 0.68) | 0.97 | -0.12 (-4.91, 4.68) | 0.966 | 0.12 (-5.5, 5.74) | 0.97 |
| 535.1 | Acute gastritis | 2413 | 0.32 (-6.29, 6.94) | 0.93 | -0.09 (-4.56, 4.37) | 0.97 | 0.38 (-17.52, 18.27) | 0.97 | -0.35 (-6.81, 6.11) | 0.923 | 0.35 (-9.17, 9.87) | 0.948 |
| 535.2 | Atrophic gastritis | 443 | 1.27 (-24.54, 27.08) | 0.929 | -0.44 (-21.42, 20.54) | 0.97 | 0.97 (-44.93, 46.87) | 0.97 | -1.3 (-25.61, 23.01) | 0.923 | 0.16 (-7.36, 7.68) | 0.97 |
| 535.6 | Duodenitis | 1124 | 0.32 (-13.58, 14.21) | 0.967 | -0.19 (-9.2, 8.82) | 0.97 | -1 (-39.14, 37.14) | 0.963 | -0.08 (-3.77, 3.61) | 0.97 | -0.41 (-18.52, 17.69) | 0.967 |
| 535.8 | Other specified gastritis | 3785 | 0.11 (-5.08, 5.3) | 0.97 | 0.18 (-8.25, 8.6) | 0.97 | -0.2 (-9.64, 9.24) | 0.97 | -0.15 (-7.41, 7.11) | 0.97 | -0.01 (-0.27, 0.26) | 0.97 |
| 537 | Other disorders of stomach and duodenum | 1624 | 0.4 (-17.16, 17.97) | 0.967 | -0.29 (-14.23, 13.64) | 0.97 | 1.43 (-50.23, 53.1) | 0.961 | -0.5 (-21.24, 20.24) | 0.966 | 0.2 (-9.15, 9.55) | 0.97 |
| 540 | Appendiceal conditions | 11597 | -0.05 (-2.49, 2.38) | 0.97 | -0.23 (-4.76, 4.31) | 0.929 | 0.48 (-4.59, 5.54) | 0.864 | 0.07 (-3.16, 3.3) | 0.97 | -0.12 (-4.9, 4.67) | 0.966 |
| 540.11 | Acute appendicitis | 11416 | -0.02 (-1.07, 1.02) | 0.97 | -0.2 (-4.6, 4.2) | 0.936 | 0.36 (-6.28, 6.99) | 0.923 | 0.05 (-2.16, 2.25) | 0.97 | -0.12 (-5.15, 4.91) | 0.966 |
| 550 | Abdominal hernia | 47853 | 0.05 (-2.27, 2.37) | 0.97 | 0.14 (-5.48, 5.75) | 0.966 | 0.14 (-6.46, 6.73) | 0.97 | -0.13 (-2.99, 2.73) | 0.936 | -0.01 (-0.68, 0.65) | 0.97 |
| 550.1 | Inguinal hernia | 29377 | -0.04 (-1.7, 1.63) | 0.97 | 0.23 (-6.01, 6.47) | 0.948 | -0.11 (-5.25, 5.03) | 0.97 | -0.04 (-1.74, 1.67) | 0.97 | -0.17 (-7.03, 6.7) | 0.966 |
| 550.2 | Diaphragmatic hernia | 7387 | -0.09 (-4.31, 4.13) | 0.97 | -0.16 (-7.7, 7.38) | 0.97 | 0.43 (-17.48, 18.35) | 0.966 | 0.08 (-3.77, 3.94) | 0.97 | 0.01 (-0.39, 0.41) | 0.97 |
| 550.3 | Femoral hernia | 1237 | -0.04 (-1.97, 1.89) | 0.97 | -0.12 (-5.8, 5.56) | 0.97 | -0.39 (-18.96, 18.18) | 0.97 | 0.16 (-7.25, 7.56) | 0.97 | -0.69 (-29.2, 27.82) | 0.966 |
| 550.4 | Umbilical hernia | 7469 | -0.16 (-7.35, 7.02) | 0.967 | 0.54 (-7.46, 8.54) | 0.902 | 0.69 (-13.54, 14.93) | 0.93 | -0.18 (-7.58, 7.23) | 0.966 | 0.07 (-3.47, 3.62) | 0.97 |
| 550.5 | Ventral hernia | 6607 | 0.07 (-3.34, 3.48) | 0.97 | 0.21 (-9.65, 10.06) | 0.97 | 0.35 (-16.26, 16.96) | 0.97 | -0.21 (-9.03, 8.6) | 0.966 | 0.3 (-12.02, 12.62) | 0.966 |
| 555 | Inflammatory bowel disease and other gastroenteritis and colitis | 8904 | -0.4 (-3.9, 3.09) | 0.833 | 0.2 (-9.21, 9.61) | 0.97 | 0.35 (-14.88, 15.57) | 0.967 | 0.26 (-5.04, 5.56) | 0.93 | -0.4 (-6.15, 5.36) | 0.901 |
| 555.1 | Regional enteritis | 3969 | -0.33 (-9.29, 8.63) | 0.948 | 0.41 (-16.52, 17.34) | 0.966 | -0.2 (-9.46, 9.06) | 0.97 | 0.2 (-9.07, 9.46) | 0.97 | -0.25 (-11.93, 11.44) | 0.97 |
| 555.2 | Ulcerative colitis | 4991 | -0.44 (-6.09, 5.2) | 0.887 | 0.05 (-2.35, 2.45) | 0.97 | 0.08 (-3.65, 3.81) | 0.97 | 0.41 (-6.44, 7.26) | 0.915 | -0.67 (-7.75, 6.42) | 0.864 |
| 555.21 | Ulcerative colitis (chronic) | 3783 | -0.22 (-6.12, 5.69) | 0.948 | 0.02 (-1.04, 1.09) | 0.97 | 0.43 (-17.41, 18.28) | 0.966 | 0.14 (-6.33, 6.6) | 0.97 | -0.47 (-5.81, 4.88) | 0.874 |
| 556 | Ulceration of the lower GI tract | 175 | -1.24 (-35.05, 32.56) | 0.948 | 0.12 (-5.35, 5.58) | 0.97 | 5.07 (-108.69, 118.83) | 0.936 | 0.83 (-38.56, 40.23) | 0.97 | 0.69 (-31.99, 33.38) | 0.97 |
| 557 | Intestinal malabsorption (non-celiac) | 1750 | 0.48 (-19.24, 20.2) | 0.966 | 0.04 (-1.84, 1.92) | 0.97 | -2.74 (-24.86, 19.37) | 0.82 | -0.01 (-0.33, 0.32) | 0.97 | -0.24 (-11.46, 10.99) | 0.97 |
| 557.1 | Celiac disease | 1032 | 1.29 (-9.92, 12.5) | 0.833 | 1.34 (-26.13, 28.8) | 0.93 | -3.74 (-18.57, 11.08) | 0.634 | -1.12 (-12.98, 10.75) | 0.864 | 0.86 (-22.55, 24.27) | 0.948 |
| 558 | Noninfectious gastroenteritis | 10389 | -0.17 (-7.27, 6.93) | 0.966 | 0.16 (-7.45, 7.77) | 0.97 | -0.37 (-16.53, 15.79) | 0.967 | 0.17 (-6.71, 7.04) | 0.966 | 0 (-0.2, 0.19) | 0.97 |
| 559 | Ileostomy status | 3073 | 0.19 (-3.7, 4.08) | 0.93 | 0.14 (-6.69, 6.98) | 0.97 | -0.51 (-9.97, 8.96) | 0.923 | -0.16 (-4.5, 4.18) | 0.948 | -0.1 (-5.02, 4.81) | 0.97 |
| 560 | Intestinal obstruction without mention of hernia | 10123 | 0.35 (-3.38, 4.08) | 0.864 | -0.25 (-10.71, 10.2) | 0.966 | -0.5 (-14.25, 13.24) | 0.948 | -0.18 (-7.39, 7.04) | 0.966 | 0.38 (-6.77, 7.54) | 0.923 |
| 560.1 | Paralytic ileus | 6024 | -0.07 (-3.29, 3.16) | 0.97 | -0.03 (-1.38, 1.32) | 0.97 | -0.38 (-16.96, 16.2) | 0.968 | 0.14 (-6.4, 6.68) | 0.97 | -0.08 (-3.99, 3.82) | 0.97 |
| 560.2 | Impaction of intestine | 133 | -0.84 (-37.3, 35.63) | 0.967 | -0.4 (-19.36, 18.56) | 0.97 | -1.7 (-82.05, 78.65) | 0.97 | 1.24 (-32.49, 34.96) | 0.948 | -0.02 (-0.82, 0.78) | 0.97 |
| 560.3 | Peritoneal or intestinal adhesions | 1662 | 0.76 (-20.05, 21.58) | 0.948 | -1.01 (-38.81, 36.78) | 0.962 | 0.99 (-45.89, 47.87) | 0.97 | -0.47 (-22.63, 21.68) | 0.97 | 1.44 (-16.83, 19.7) | 0.887 |
| 560.4 | Other intestinal obstruction | 5874 | 0.41 (-7.85, 8.67) | 0.929 | -0.23 (-10.93, 10.48) | 0.97 | -0.17 (-8.12, 7.78) | 0.97 | -0.29 (-10.8, 10.21) | 0.961 | 0.77 (-7.37, 8.91) | 0.864 |
| 561 | Symptoms involving digestive system | 6628 | -0.22 (-6.28, 5.84) | 0.948 | 0.31 (-10.7, 11.32) | 0.96 | 0.07 (-3.18, 3.32) | 0.97 | 0.09 (-4.16, 4.34) | 0.97 | 0.09 (-4.12, 4.3) | 0.97 |
| 561.1 | Diarrhea | 2419 | -0.22 (-10.56, 10.12) | 0.97 | 0.46 (-18.5, 19.42) | 0.966 | -0.25 (-11.94, 11.45) | 0.97 | 0.07 (-3.23, 3.37) | 0.97 | 0.26 (-11.93, 12.44) | 0.97 |
| 562 | Diverticulosis and diverticulitis | 16569 | 0.06 (-2.83, 2.95) | 0.97 | 0.17 (-6.77, 7.11) | 0.966 | 0.08 (-3.64, 3.8) | 0.97 | -0.14 (-3.17, 2.9) | 0.936 | 0.11 (-4.76, 4.98) | 0.967 |
| 562.1 | Diverticulosis | 16569 | 0.06 (-2.83, 2.95) | 0.97 | 0.17 (-6.77, 7.11) | 0.966 | 0.08 (-3.64, 3.8) | 0.97 | -0.14 (-3.17, 2.9) | 0.936 | 0.11 (-4.76, 4.98) | 0.967 |
| 563 | Constipation | 28728 | 0.01 (-0.61, 0.64) | 0.97 | 0.1 (-4.6, 4.79) | 0.97 | -0.41 (-4.8, 3.98) | 0.864 | 0.02 (-0.97, 1.01) | 0.97 | -0.24 (-2.76, 2.28) | 0.864 |
| 564 | Functional digestive disorders | 15602 | -0.1 (-4.31, 4.12) | 0.968 | 0.08 (-3.48, 3.63) | 0.97 | 0.42 (-8.26, 9.11) | 0.93 | -0.01 (-0.59, 0.57) | 0.97 | 0.17 (-6.88, 7.22) | 0.966 |
| 564.1 | Irritable Bowel Syndrome | 9444 | -0.31 (-3.64, 3.01) | 0.864 | 0.37 (-7.24, 7.98) | 0.93 | 0.79 (-7.6, 9.18) | 0.864 | 0.02 (-0.94, 0.98) | 0.97 | 0.47 (-4.56, 5.5) | 0.864 |
| 564.8 | Abnormal findings on exam of gastrointestinal tract/ abdominal area | 748 | 0.07 (-3.16, 3.3) | 0.97 | -0.47 (-8.44, 7.49) | 0.915 | 0.26 (-12.25, 12.78) | 0.97 | 0.05 (-2.25, 2.35) | 0.97 | 0.12 (-5.77, 6.02) | 0.97 |
| 564.9 | Personal history of diseases of digestive system | 690 | -0.6 (-8.44, 7.24) | 0.89 | -0.29 (-14.13, 13.55) | 0.97 | 0.39 (-18, 18.78) | 0.97 | 0.65 (-7.01, 8.32) | 0.877 | 0.5 (-20.29, 21.3) | 0.966 |
| 565 | Anal and rectal conditions | 16721 | -0.1 (-4.74, 4.54) | 0.97 | 0.29 (-7.49, 8.06) | 0.948 | -0.09 (-4.44, 4.26) | 0.97 | -0.01 (-0.39, 0.37) | 0.97 | -0.03 (-1.47, 1.41) | 0.97 |
| 565.1 | Anal and rectal polyp | 1071 | -0.57 (-12.28, 11.14) | 0.93 | 0.48 (-22.42, 23.39) | 0.97 | -1.05 (-37.91, 35.82) | 0.96 | 0.58 (-11.29, 12.45) | 0.93 | 0.18 (-8.41, 8.78) | 0.97 |
| 567 | Peritonitis and retroperitoneal infections | 3949 | -0.11 (-5.25, 5.03) | 0.97 | -0.63 (-8.68, 7.42) | 0.887 | -0.62 (-26.33, 25.08) | 0.966 | 0.47 (-4.53, 5.47) | 0.864 | 0.36 (-13.23, 13.94) | 0.963 |
| 568 | Other disorders of peritoneum | 2156 | 0.77 (-8.25, 9.79) | 0.877 | -0.39 (-18.99, 18.21) | 0.97 | -1.61 (-33.92, 30.71) | 0.929 | -0.36 (-15.69, 14.96) | 0.966 | 0.61 (-20.93, 22.15) | 0.96 |
| 568.1 | Peritoneal adhesions (postoperative) (postinfection) | 1918 | 0.81 (-7.84, 9.47) | 0.864 | -0.93 (-20.07, 18.21) | 0.93 | -0.85 (-37.74, 36.05) | 0.967 | -0.28 (-13.55, 12.99) | 0.97 | 0.85 (-16.59, 18.29) | 0.93 |
| 569 | Other disorders of intestine | 5300 | -0.01 (-0.7, 0.67) | 0.97 | 0.37 (-9.63, 10.36) | 0.948 | -0.39 (-16.65, 15.86) | 0.966 | -0.06 (-3.04, 2.92) | 0.97 | 0.39 (-7.39, 8.16) | 0.929 |
| 569.1 | Toxic gastroenteritis and colitis | 409 | -0.05 (-2.36, 2.26) | 0.97 | 0.21 (-9.79, 10.22) | 0.97 | 0.35 (-16.35, 17.05) | 0.97 | -0.1 (-4.94, 4.73) | 0.97 | 0.16 (-7.23, 7.55) | 0.97 |
| 569.2 | Gastrointestinal complications | 1263 | 0.09 (-4.31, 4.49) | 0.97 | 0.61 (-11.71, 12.94) | 0.929 | -0.28 (-13.54, 12.98) | 0.97 | -0.3 (-8.59, 7.99) | 0.948 | 0.48 (-10.2, 11.15) | 0.936 |
| 571 | Chronic liver disease and cirrhosis | 6857 | 0.03 (-1.39, 1.45) | 0.97 | -0.3 (-7.13, 6.52) | 0.936 | 0.36 (-14.3, 15.01) | 0.966 | 0.04 (-1.99, 2.07) | 0.97 | -0.38 (-4.43, 3.67) | 0.864 |
| 571.5 | Other chronic nonalcoholic liver disease | 1524 | -0.36 (-15.28, 14.56) | 0.966 | -1.45 (-9.82, 6.92) | 0.747 | -0.02 (-1.03, 0.99) | 0.97 | 1.03 (-4.07, 6.13) | 0.705 | -0.48 (-20.36, 19.39) | 0.966 |
| 571.51 | Cirrhosis of liver without mention of alcohol | 2301 | 0.02 (-0.92, 0.96) | 0.97 | 0.2 (-9.27, 9.67) | 0.97 | 0.36 (-16.7, 17.42) | 0.97 | -0.16 (-7.82, 7.5) | 0.97 | -0.34 (-14.38, 13.7) | 0.966 |
| 571.6 | Primary biliary cirrhosis | 437 | -0.1 (-4.72, 4.52) | 0.97 | -1.03 (-43.67, 41.6) | 0.966 | 0.11 (-5.11, 5.33) | 0.97 | 0.45 (-20.89, 21.79) | 0.97 | -0.76 (-32.05, 30.53) | 0.966 |
| 571.8 | Liver abscess and sequelae of chronic liver disease | 3922 | 0 (-0.06, 0.05) | 0.97 | 0.02 (-0.95, 0.99) | 0.97 | 0.35 (-9.06, 9.76) | 0.948 | -0.07 (-3.22, 3.09) | 0.97 | -0.2 (-5.68, 5.28) | 0.948 |
| 571.81 | Portal hypertension | 1101 | 0.14 (-6.35, 6.63) | 0.97 | -0.33 (-13.76, 13.1) | 0.966 | -0.14 (-6.6, 6.32) | 0.97 | 0.03 (-1.24, 1.3) | 0.97 | -0.61 (-7.09, 5.88) | 0.864 |
| 573 | Other disorders of liver | 4833 | -0.03 (-1.31, 1.25) | 0.97 | -0.4 (-8.64, 7.83) | 0.93 | -0.09 (-4.52, 4.33) | 0.97 | 0.21 (-5.61, 6.03) | 0.948 | -0.15 (-7.11, 6.82) | 0.97 |
| 573.2 | Liver replaced by transplant | 285 | -0.81 (-10.17, 8.55) | 0.874 | -0.54 (-24.14, 23.05) | 0.967 | 0.63 (-29.36, 30.62) | 0.97 | 1.03 (-7.93, 9.99) | 0.833 | 0.54 (-21.72, 22.8) | 0.966 |
| 573.3 | Hepatomegaly | 453 | 0.33 (-15.08, 15.74) | 0.97 | -0.71 (-34.21, 32.79) | 0.97 | -0.8 (-38.39, 36.8) | 0.97 | 0.09 (-4.38, 4.57) | 0.97 | -1.63 (-35.75, 32.49) | 0.932 |
| 573.7 | Abnormal results of function study of liver | 108 | 0.63 (-29.01, 30.26) | 0.97 | -1.59 (-44.86, 41.68) | 0.948 | 3.99 (-184.65, 192.63) | 0.97 | 0.1 (-4.7, 4.9) | 0.97 | 1.06 (-48.94, 51.06) | 0.97 |
| 573.9 | Abnormal serum enzyme levels | 842 | 0.35 (-7.47, 8.17) | 0.936 | -0.44 (-12.53, 11.64) | 0.948 | -0.15 (-7, 6.71) | 0.97 | -0.12 (-5.87, 5.62) | 0.97 | -0.14 (-6.98, 6.69) | 0.97 |
| 574 | Cholelithiasis and cholecystitis | 31530 | -0.31 (-1.13, 0.5) | 0.457 | 0.38 (-2.71, 3.47) | 0.82 | 0.16 (-7.48, 7.8) | 0.97 | 0.14 (-2.65, 2.92) | 0.931 | -0.09 (-4.41, 4.23) | 0.97 |
| 574.1 | Cholelithiasis | 24028 | -0.35 (-1.3, 0.59) | 0.472 | 0.48 (-1.67, 2.64) | 0.673 | 0.28 (-10.33, 10.89) | 0.963 | 0.11 (-4.58, 4.81) | 0.966 | -0.14 (-6.03, 5.74) | 0.966 |
| 574.11 | Cholelithiasis with acute cholecystitis | 3331 | -0.14 (-6.86, 6.57) | 0.97 | -0.35 (-14.87, 14.17) | 0.966 | 0.33 (-15.38, 16.04) | 0.97 | 0.23 (-9.25, 9.72) | 0.966 | 0.09 (-4.09, 4.27) | 0.97 |
| 574.12 | Cholelithiasis with other cholecystitis | 2650 | -0.24 (-9.94, 9.47) | 0.966 | 0.11 (-5.2, 5.42) | 0.97 | -0.3 (-14.62, 14.02) | 0.97 | 0.25 (-10.08, 10.58) | 0.966 | -0.12 (-5.67, 5.44) | 0.97 |
| 574.2 | Calculus of bile duct | 8401 | -0.52 (-1.89, 0.85) | 0.469 | 0.7 (-2.75, 4.14) | 0.705 | -0.15 (-7.04, 6.75) | 0.97 | 0.28 (-3.22, 3.77) | 0.887 | -0.21 (-8.93, 8.51) | 0.966 |
| 574.3 | Cholecystitis without cholelithiasis | 5707 | -0.1 (-4.65, 4.46) | 0.97 | 0.49 (-9.89, 10.87) | 0.932 | -0.66 (-18.59, 17.27) | 0.948 | 0 (-0.17, 0.17) | 0.97 | -0.15 (-7.18, 6.88) | 0.97 |
| 575 | Other biliary tract disease | 4230 | 0.22 (-5.85, 6.29) | 0.948 | -0.39 (-9.13, 8.35) | 0.936 | 0.73 (-12.97, 14.44) | 0.923 | -0.19 (-8.02, 7.64) | 0.966 | -0.04 (-1.77, 1.7) | 0.97 |
| 575.1 | Cholangitis | 1592 | 0 (-0.21, 0.22) | 0.97 | -0.09 (-4.15, 3.98) | 0.97 | 0.11 (-5.02, 5.24) | 0.97 | 0.01 (-0.54, 0.56) | 0.97 | -0.22 (-10.43, 10) | 0.97 |
| 575.2 | Obstruction of bile duct | 1593 | 0.26 (-6.95, 7.48) | 0.948 | -0.22 (-10.65, 10.21) | 0.97 | 0.61 (-16.05, 17.27) | 0.948 | -0.31 (-7.4, 6.77) | 0.937 | -0.1 (-4.72, 4.52) | 0.97 |
| 575.7 | Other disorders of gallbladder | 543 | 0.12 (-5.44, 5.67) | 0.97 | -1.16 (-17.43, 15.1) | 0.897 | 0.8 (-37.19, 38.8) | 0.97 | 0.16 (-7.29, 7.61) | 0.97 | -0.08 (-3.97, 3.8) | 0.97 |
| 575.8 | Other disorders of biliary tract | 1264 | -0.15 (-7.24, 6.94) | 0.97 | -0.03 (-1.36, 1.31) | 0.97 | 1.7 (-22.72, 26.12) | 0.9 | -0.12 (-5.97, 5.72) | 0.97 | -0.15 (-7.06, 6.77) | 0.97 |
| 577 | Diseases of pancreas | 3101 | 0.88 (-3.45, 5.2) | 0.705 | -0.6 (-17.06, 15.85) | 0.948 | -0.98 (-27.56, 25.61) | 0.948 | -0.47 (-10.07, 9.13) | 0.93 | -0.27 (-12.96, 12.42) | 0.97 |
| 577.1 | Acute pancreatitis | 151 | -1.28 (-36.08, 33.52) | 0.948 | 1.49 (-59.88, 62.86) | 0.966 | -2.87 (-65.76, 60.03) | 0.935 | 1.14 (-29.87, 32.15) | 0.948 | 0.26 (-12.14, 12.66) | 0.97 |
| 577.2 | Chronic pancreatitis | 2170 | 0.68 (-6.51, 7.86) | 0.864 | -1.1 (-12.76, 10.56) | 0.864 | -0.65 (-31.39, 30.09) | 0.97 | -0.1 (-4.78, 4.58) | 0.97 | -1.11 (-10.81, 8.58) | 0.833 |
| 577.3 | Cyst and pseudocyst of pancreas | 739 | -0.72 (-20.25, 18.82) | 0.948 | 1.6 (-18.03, 21.23) | 0.882 | -2.11 (-29.66, 25.44) | 0.89 | 0.52 (-21.07, 22.12) | 0.966 | 0.18 (-8.27, 8.63) | 0.97 |
| 578 | Gastrointestinal hemorrhage | 20117 | 0.19 (-1.86, 2.25) | 0.864 | 0.04 (-2.02, 2.1) | 0.97 | -0.12 (-5.91, 5.66) | 0.97 | -0.19 (-2.21, 1.83) | 0.864 | 0.18 (-3.83, 4.19) | 0.936 |
| 578.1 | Hematemesis | 5358 | 0.3 (-5.38, 5.99) | 0.923 | 0.24 (-10.38, 10.86) | 0.968 | 0.56 (-14.76, 15.89) | 0.948 | -0.49 (-4.42, 3.44) | 0.82 | 0.09 (-3.98, 4.15) | 0.97 |
| 578.2 | Blood in stool | 3161 | 0.35 (-6.96, 7.67) | 0.931 | 0.11 (-4.87, 5.08) | 0.97 | -0.4 (-19.54, 18.73) | 0.97 | -0.33 (-7.71, 7.06) | 0.937 | 0.17 (-7.69, 8.03) | 0.97 |
| 578.8 | Hemorrhage of rectum and anus | 11100 | 0.06 (-2.78, 2.9) | 0.97 | -0.14 (-6.69, 6.42) | 0.97 | -0.35 (-13.44, 12.73) | 0.962 | 0.05 (-2.33, 2.43) | 0.97 | 0.19 (-6.97, 7.35) | 0.962 |
| 578.9 | Hemorrhage of gastrointestinal tract | 3431 | 0.23 (-2.74, 3.21) | 0.887 | 0.27 (-7.08, 7.62) | 0.948 | 0.23 (-10.53, 10.99) | 0.97 | -0.37 (-2.18, 1.45) | 0.705 | 0.15 (-6.47, 6.77) | 0.967 |
| 579 | Other symptoms involving abdomen and pelvis | 1355 | -0.07 (-3.26, 3.12) | 0.97 | 0.77 (-16.59, 18.14) | 0.936 | -0.48 (-23.1, 22.14) | 0.97 | -0.2 (-9.43, 9.04) | 0.97 | -0.74 (-15.97, 14.48) | 0.93 |
| 579.2 | Splenomegaly | 550 | 0.09 (-4.11, 4.29) | 0.97 | 0.44 (-20.45, 21.33) | 0.97 | -0.07 (-3.52, 3.38) | 0.97 | -0.26 (-12.79, 12.26) | 0.97 | -1.19 (-26.03, 23.66) | 0.932 |
| 579.8 | Nonspecific abnormal findings in stool contents | 557 | -0.27 (-11.96, 11.42) | 0.967 | 0.64 (-13.34, 14.62) | 0.935 | -0.66 (-27.78, 26.46) | 0.966 | 0.1 (-4.6, 4.79) | 0.97 | 0.1 (-4.5, 4.7) | 0.97 |
| 580 | Nephritis; nephrosis; renal sclerosis | 2488 | 0.67 (-6.5, 7.85) | 0.864 | -0.14 (-6.6, 6.32) | 0.97 | 1.27 (-22.43, 24.96) | 0.923 | -0.85 (-7.72, 6.02) | 0.82 | 0.35 (-15.99, 16.68) | 0.97 |
| 580.11 | Proliferative glomerulonephritis | 553 | 0.21 (-9.53, 9.94) | 0.97 | 1.13 (-45.57, 47.84) | 0.966 | 2.36 (-52.81, 57.54) | 0.939 | -1.02 (-22.1, 20.06) | 0.931 | -0.21 (-10.21, 9.79) | 0.97 |
| 580.12 | Non-proliferative glomerulonephritis | 568 | 0 (-0.18, 0.17) | 0.97 | 0.51 (-23.81, 24.84) | 0.97 | -0.25 (-12.3, 11.79) | 0.97 | -0.17 (-8.2, 7.86) | 0.97 | 1.56 (-30.38, 33.5) | 0.93 |
| 580.14 | Chronic glomerulonephritis, NOS | 579 | 1.37 (-16.55, 19.3) | 0.89 | -1.29 (-52.3, 49.72) | 0.964 | -0.59 (-28.7, 27.51) | 0.97 | -0.68 (-29.35, 27.99) | 0.966 | 1.58 (-30.88, 34.04) | 0.93 |
| 580.2 | Nephrotic syndrome without mention of glomerulonephritis | 916 | 0.37 (-17.22, 17.96) | 0.97 | -0.75 (-31.48, 29.99) | 0.966 | 0.19 (-8.9, 9.28) | 0.97 | -0.1 (-4.59, 4.4) | 0.97 | -0.19 (-9.28, 8.9) | 0.97 |
| 580.32 | Nephritis and nephropathy with pathological lesion | 879 | 0.35 (-16.22, 16.92) | 0.97 | -0.53 (-25.66, 24.59) | 0.97 | 2.75 (-26.47, 31.97) | 0.864 | -0.64 (-18.05, 16.77) | 0.948 | 0.3 (-13.97, 14.57) | 0.97 |
| 585 | Renal failure | 14340 | -0.06 (-2.55, 2.43) | 0.966 | -0.03 (-1.64, 1.57) | 0.97 | 0 (-0.03, 0.03) | 0.97 | 0.07 (-1.97, 2.12) | 0.948 | 0.04 (-1.77, 1.84) | 0.97 |
| 585.1 | Acute renal failure | 6882 | -0.05 (-2.55, 2.44) | 0.97 | -0.11 (-5.54, 5.31) | 0.97 | -0.1 (-4.84, 4.64) | 0.97 | 0.12 (-3.12, 3.35) | 0.948 | -0.03 (-1.64, 1.58) | 0.97 |
| 585.3 | Chronic renal failure [CKD] | 9218 | 0 (-0.03, 0.03) | 0.97 | -0.03 (-1.24, 1.18) | 0.97 | 0.11 (-5.12, 5.34) | 0.97 | -0.01 (-0.38, 0.37) | 0.97 | 0.01 (-0.42, 0.44) | 0.97 |
| 585.31 | Renal dialysis | 1893 | 0.17 (-6.78, 7.11) | 0.966 | 0.12 (-5.77, 6.01) | 0.97 | -0.43 (-18.01, 17.16) | 0.966 | -0.15 (-6.69, 6.39) | 0.968 | 0.15 (-6.77, 7.06) | 0.97 |
| 586 | Other disorders of the kidney and ureters | 6384 | -0.01 (-0.65, 0.62) | 0.97 | -0.7 (-9.56, 8.17) | 0.887 | 0.14 (-6.61, 6.9) | 0.97 | 0.28 (-9.74, 10.29) | 0.961 | 0.17 (-7.85, 8.19) | 0.97 |
| 586.11 | Small kidney | 141 | -0.32 (-15.4, 14.76) | 0.97 | -0.42 (-20.21, 19.38) | 0.97 | 0.2 (-9.48, 9.89) | 0.97 | 0.46 (-21.21, 22.13) | 0.97 | 0.64 (-29.84, 31.13) | 0.97 |
| 586.2 | Cyst of kidney, acquired | 721 | -0.48 (-21.59, 20.63) | 0.968 | 0.1 (-4.75, 4.95) | 0.97 | -3.05 (-29.63, 23.52) | 0.833 | 1.06 (-12.37, 14.49) | 0.887 | 0.65 (-27.73, 29.03) | 0.967 |
| 586.4 | Stricture/obstruction of ureter | 2224 | -0.57 (-12.19, 11.06) | 0.93 | -0.47 (-22.5, 21.56) | 0.97 | 2 (-15.39, 19.38) | 0.833 | 0.32 (-13.69, 14.34) | 0.967 | 0.22 (-10.19, 10.63) | 0.97 |
| 587 | Kidney replaced by transpant | 660 | -0.12 (-5.58, 5.35) | 0.97 | -0.41 (-19.86, 19.04) | 0.97 | 1.02 (-41.01, 43.05) | 0.966 | 0.06 (-2.91, 3.04) | 0.97 | -0.4 (-19.42, 18.61) | 0.97 |
| 588 | Disorders resulting from impaired renal function | 619 | -0.02 (-0.73, 0.7) | 0.97 | 0.21 (-9.59, 10.01) | 0.97 | 0.92 (-42.58, 44.42) | 0.97 | -0.24 (-11.74, 11.25) | 0.97 | 0.01 (-0.54, 0.56) | 0.97 |
| 590 | Pyelonephritis | 2804 | 0.5 (-13.19, 14.2) | 0.948 | 0.33 (-15.4, 16.07) | 0.97 | 0.22 (-9.98, 10.41) | 0.97 | -0.68 (-9.35, 7.99) | 0.887 | 0.25 (-11.63, 12.14) | 0.97 |
| 591 | Urinary tract infection | 23736 | 0.03 (-1.22, 1.28) | 0.97 | 0.06 (-2.6, 2.71) | 0.97 | -0.04 (-2.08, 1.99) | 0.97 | -0.04 (-2.02, 1.94) | 0.97 | 0.1 (-4.06, 4.26) | 0.966 |
| 592 | Cystitis and urethritis | 48639 | 0.02 (-0.84, 0.87) | 0.97 | 0.19 (-2.24, 2.63) | 0.887 | 0.01 (-0.56, 0.58) | 0.97 | -0.1 (-2.18, 1.97) | 0.93 | -0.07 (-3.45, 3.31) | 0.97 |
| 592.1 | Cystitis | 17198 | -0.05 (-2.43, 2.33) | 0.97 | 0.23 (-4.47, 4.92) | 0.93 | 0.01 (-0.31, 0.32) | 0.97 | -0.05 (-2.22, 2.13) | 0.97 | 0.09 (-4.27, 4.45) | 0.97 |
| 592.11 | Acute cystitis | 34985 | 0.01 (-0.67, 0.7) | 0.97 | 0.16 (-3.45, 3.77) | 0.936 | 0.11 (-4.98, 5.19) | 0.97 | -0.1 (-2.41, 2.21) | 0.936 | -0.16 (-3.41, 3.09) | 0.929 |
| 592.12 | Chronic cystitis | 1502 | -0.76 (-16.32, 14.8) | 0.93 | -0.79 (-33.26, 31.68) | 0.966 | 0.94 (-43.31, 45.18) | 0.97 | 0.89 (-10.78, 12.56) | 0.89 | 0.8 (-21.01, 22.62) | 0.948 |
| 592.13 | Chronic interstitial cystitis | 1242 | -1.05 (-9.47, 7.38) | 0.82 | -0.29 (-14.21, 13.62) | 0.97 | 1.24 (-32.63, 35.12) | 0.948 | 0.96 (-7.39, 9.3) | 0.833 | 0.36 (-16.77, 17.5) | 0.97 |
| 592.2 | Urethritis and urethral syndrome | 527 | 0.02 (-0.97, 1.01) | 0.97 | 0.62 (-28.68, 29.92) | 0.97 | -2.66 (-58.44, 53.12) | 0.932 | 0.33 (-15.15, 15.81) | 0.97 | -1.56 (-43.98, 40.86) | 0.948 |
| 592.3 | Urethral stricture due to infecton | 165 | 1.01 (-40.74, 42.77) | 0.966 | -1.43 (-63.99, 61.12) | 0.967 | -0.75 (-36.05, 34.56) | 0.97 | -0.48 (-23.36, 22.39) | 0.97 | 1.19 (-47.77, 50.15) | 0.966 |
| 593 | Hematuria | 494 | 0.32 (-14.95, 15.6) | 0.97 | -1.53 (-30.07, 27.01) | 0.923 | -2.71 (-58.32, 52.9) | 0.93 | 0.62 (-24.77, 26) | 0.966 | 0.04 (-2.05, 2.14) | 0.97 |
| 594 | Urinary calculus | 20964 | 0.04 (-1.67, 1.74) | 0.97 | -0.07 (-3.43, 3.29) | 0.97 | 0.4 (-9.33, 10.12) | 0.942 | -0.08 (-3.75, 3.6) | 0.97 | -0.18 (-7.54, 7.18) | 0.966 |
| 594.1 | Calculus of kidney | 11345 | 0.18 (-4.66, 5.02) | 0.948 | -0.1 (-4.9, 4.7) | 0.97 | 0.19 (-8.72, 9.09) | 0.97 | -0.17 (-4.8, 4.46) | 0.948 | 0.06 (-2.6, 2.72) | 0.97 |
| 594.2 | Calculus of lower urinary tract | 2146 | -0.67 (-10.35, 9.01) | 0.901 | 0.52 (-23.74, 24.77) | 0.97 | 0.07 (-3.17, 3.3) | 0.97 | 0.48 (-12.48, 13.43) | 0.948 | 0.7 (-18.39, 19.8) | 0.948 |
| 594.3 | Calculus of ureter | 11775 | 0.02 (-0.79, 0.82) | 0.97 | -0.06 (-2.9, 2.78) | 0.97 | 0.75 (-8.79, 10.29) | 0.887 | -0.12 (-5.95, 5.7) | 0.97 | -0.56 (-5.47, 4.34) | 0.833 |
| 595 | Hydronephrosis | 5907 | -0.01 (-0.6, 0.58) | 0.97 | -0.13 (-6.08, 5.83) | 0.97 | -0.38 (-17.07, 16.3) | 0.967 | 0.13 (-6.02, 6.28) | 0.97 | -0.08 (-3.91, 3.75) | 0.97 |
| 596 | Other disorders of bladder | 4506 | 0.01 (-0.28, 0.3) | 0.97 | -0.18 (-8.64, 8.28) | 0.97 | 0.91 (-18.22, 20.04) | 0.932 | -0.1 (-4.65, 4.45) | 0.97 | 0.26 (-12.05, 12.57) | 0.97 |
| 596.1 | Bladder neck obstruction | 565 | -1.2 (-28.38, 25.98) | 0.937 | 0.72 (-33.3, 34.74) | 0.97 | 0.83 (-38.59, 40.26) | 0.97 | 0.8 (-32.06, 33.65) | 0.966 | 0.11 (-5.18, 5.41) | 0.97 |
| 596.5 | Functional disorders of bladder | 2563 | 0.11 (-5.16, 5.38) | 0.97 | -0.76 (-16.75, 15.23) | 0.932 | 1.18 (-22.95, 25.3) | 0.93 | -0.03 (-1.45, 1.39) | 0.97 | 0.15 (-6.91, 7.21) | 0.97 |
| 597 | Other disorders of urethra and urinary tract | 3707 | -0.22 (-10.67, 10.23) | 0.97 | -0.14 (-6.73, 6.46) | 0.97 | -0.56 (-26.86, 25.74) | 0.97 | 0.39 (-10.27, 11.06) | 0.948 | -0.63 (-14.04, 12.77) | 0.932 |
| 597.1 | Urethral stricture (not specified as infectious) | 2683 | -0.29 (-14.03, 13.45) | 0.97 | 0.15 (-6.77, 7.06) | 0.97 | -0.44 (-21.3, 20.42) | 0.97 | 0.32 (-13.84, 14.49) | 0.967 | -0.54 (-22.75, 21.67) | 0.966 |
| 598 | Abnormal findings on examination of urine | 1199 | -0.25 (-11.04, 10.54) | 0.968 | -1.1 (-10.67, 8.47) | 0.833 | 0.63 (-25.24, 26.5) | 0.966 | 0.57 (-6.07, 7.21) | 0.877 | -0.34 (-15.38, 14.69) | 0.967 |
| 598.9 | Other nonspecific findings on examination of urine | 108 | -3.43 (-39.88, 33.03) | 0.864 | -0.53 (-25.65, 24.58) | 0.97 | 2.35 (-108.89, 113.6) | 0.97 | 3.21 (-37.59, 44) | 0.887 | 4.72 (-55.34, 64.79) | 0.887 |
| 599 | Other symptoms/disorders or the urinary system | 31107 | 0.02 (-1.02, 1.07) | 0.97 | -0.22 (-1.99, 1.55) | 0.82 | 0.09 (-4.04, 4.21) | 0.97 | 0.05 (-2.16, 2.27) | 0.966 | 0 (-0.16, 0.15) | 0.97 |
| 599.1 | Urinary obstruction | 365 | 0.43 (-18.42, 19.28) | 0.967 | -0.3 (-14.64, 14.03) | 0.97 | -2.28 (-26.18, 21.62) | 0.862 | 0.25 (-11.42, 11.91) | 0.97 | -0.62 (-26.14, 24.9) | 0.966 |
| 599.2 | Retention of urine | 681 | -0.02 (-0.89, 0.85) | 0.97 | -0.6 (-11.8, 10.6) | 0.923 | 0.66 (-14.24, 15.55) | 0.937 | 0.09 (-4.39, 4.58) | 0.97 | 0.23 (-10.76, 11.23) | 0.97 |
| 599.3 | Dysuria | 1738 | 0.02 (-1.07, 1.12) | 0.97 | -0.86 (-10.01, 8.29) | 0.864 | 0.13 (-6.18, 6.45) | 0.97 | 0.34 (-10.32, 10.99) | 0.955 | -0.12 (-5.93, 5.69) | 0.97 |
| 599.4 | Urinary incontinence | 8936 | -0.01 (-0.28, 0.26) | 0.97 | 0.01 (-0.27, 0.28) | 0.97 | -0.39 (-9.3, 8.52) | 0.938 | 0.06 (-2.97, 3.1) | 0.97 | -0.23 (-4.87, 4.42) | 0.931 |
| 599.8 | Other symptoms involving urinary system | 1018 | -0.36 (-10.04, 9.33) | 0.948 | -0.67 (-14.44, 13.09) | 0.93 | -0.36 (-17.45, 16.73) | 0.97 | 0.69 (-5.29, 6.66) | 0.833 | 0.59 (-11.71, 12.89) | 0.932 |
| 599.9 | Other abnormality of urination | 17400 | 0.07 (-1.8, 1.94) | 0.948 | -0.23 (-2.11, 1.65) | 0.82 | 0.27 (-2.59, 3.12) | 0.864 | -0.02 (-1.03, 0.98) | 0.97 | 0.09 (-3.06, 3.24) | 0.96 |
| 601 | Inflammatory diseases of prostate | 3245 | 0.02 (-0.98, 1.02) | 0.97 | 0.3 (-14, 14.6) | 0.97 | -0.7 (-31.09, 29.69) | 0.967 | -0.03 (-1.22, 1.17) | 0.97 | -0.67 (-15.68, 14.34) | 0.936 |
| 601.1 | Prostatitis | 380 | -0.51 (-24.61, 23.59) | 0.97 | -0.33 (-15.92, 15.26) | 0.97 | -0.64 (-31.02, 29.73) | 0.97 | 0.74 (-29.95, 31.44) | 0.966 | -0.11 (-5.22, 5.01) | 0.97 |
| 601.11 | Acute prostatitis | 360 | 0.4 (-18.51, 19.31) | 0.97 | -4.67 (-19.75, 10.42) | 0.556 | -0.33 (-15.94, 15.28) | 0.97 | 1.41 (-24.87, 27.69) | 0.923 | -2.42 (-28.2, 23.36) | 0.864 |
| 601.12 | Chronic prostatitis | 210 | -1.11 (-50.06, 47.84) | 0.968 | 2.97 (-64.94, 70.87) | 0.938 | 1.01 (-46.98, 49.01) | 0.97 | -0.19 (-9.22, 8.83) | 0.97 | 4.36 (-42.06, 50.79) | 0.864 |
| 601.3 | Orchitis and epididymitis | 239 | 0.71 (-32.99, 34.41) | 0.97 | -2.75 (-35.34, 29.83) | 0.878 | 0.18 (-8.15, 8.5) | 0.97 | 0.36 (-16.77, 17.5) | 0.97 | 1.52 (-39.73, 42.76) | 0.948 |
| 601.4 | Balanoposthitis | 630 | 1.47 (-14.21, 17.15) | 0.864 | -0.66 (-31.98, 30.66) | 0.97 | -4.44 (-40.2, 31.32) | 0.82 | -0.35 (-16.72, 16.03) | 0.97 | -0.35 (-16.93, 16.22) | 0.97 |
| 601.8 | Other inflammatory disorders of male genital organs | 1541 | 0.53 (-13.92, 14.98) | 0.948 | 1.52 (-12.24, 15.27) | 0.84 | -1.35 (-34.6, 31.9) | 0.942 | -0.95 (-11.05, 9.15) | 0.864 | -1.95 (-8.25, 4.35) | 0.556 |
| 602 | Other disorders of prostate | 240 | -0.62 (-29.79, 28.56) | 0.97 | 1.74 (-70.01, 73.5) | 0.966 | -0.43 (-20.9, 20.03) | 0.97 | -0.08 (-3.62, 3.47) | 0.97 | -2.3 (-53.88, 49.28) | 0.936 |
| 603 | Other disorders of testis | 10411 | -0.05 (-2.22, 2.13) | 0.97 | 0.42 (-8.32, 9.15) | 0.932 | 0.03 (-1.17, 1.22) | 0.97 | -0.13 (-6.28, 6.02) | 0.97 | 0.33 (-8.59, 9.24) | 0.948 |
| 603.1 | Hydrocele | 2978 | -0.02 (-0.81, 0.78) | 0.97 | 0.1 (-4.66, 4.87) | 0.97 | 0.06 (-2.68, 2.8) | 0.97 | -0.04 (-1.76, 1.68) | 0.97 | 0.49 (-9.89, 10.87) | 0.932 |
| 603.2 | Spermatocele | 2308 | -0.32 (-14.08, 13.45) | 0.967 | 0.73 (-19.17, 20.63) | 0.948 | -0.45 (-21.89, 20.98) | 0.97 | 0.12 (-5.34, 5.57) | 0.97 | -0.37 (-18.04, 17.29) | 0.97 |
| 604 | Disorders of penis | 1445 | 0.22 (-10.3, 10.75) | 0.97 | 0.45 (-20.87, 21.77) | 0.97 | -0.73 (-33.1, 31.63) | 0.968 | -0.28 (-13.32, 12.77) | 0.97 | 0.49 (-19.8, 20.79) | 0.966 |
| 604.1 | Redundant prepuce and phimosis/BXO | 838 | -0.23 (-11.29, 10.83) | 0.97 | 1.06 (-22.62, 24.74) | 0.936 | -0.01 (-0.54, 0.51) | 0.97 | -0.21 (-10.05, 9.63) | 0.97 | 0.46 (-21.12, 22.03) | 0.97 |
| 605 | Erectile dysfunction [ED] | 2185 | -0.17 (-8.42, 8.07) | 0.97 | 0.31 (-14.15, 14.76) | 0.97 | -0.35 (-16.78, 16.09) | 0.97 | 0.1 (-4.7, 4.9) | 0.97 | 0.15 (-6.9, 7.19) | 0.97 |
| 608 | Other disorders of male genital organs | 4263 | -0.61 (-7.04, 5.83) | 0.864 | 1.04 (-10.02, 12.1) | 0.864 | 1.27 (-17.01, 19.55) | 0.9 | -0.04 (-1.75, 1.68) | 0.97 | -0.09 (-4.2, 4.03) | 0.97 |
| 610 | Benign mammary dysplasias | 11509 | 0.07 (-3.03, 3.16) | 0.97 | -0.35 (-8.27, 7.56) | 0.936 | -0.23 (-10.91, 10.46) | 0.97 | 0.11 (-5.14, 5.36) | 0.97 | 0.48 (-3.73, 4.7) | 0.833 |
| 610.1 | Cystic mastopathy | 2839 | -0.05 (-2.38, 2.28) | 0.97 | 0.4 (-15.88, 16.67) | 0.966 | 0.15 (-6.76, 7.05) | 0.97 | -0.13 (-6.11, 5.86) | 0.97 | -0.22 (-10.78, 10.34) | 0.97 |
| 610.2 | Fibroadenosis of breast | 8117 | 0.08 (-3.55, 3.7) | 0.97 | -0.56 (-6.54, 5.42) | 0.865 | -0.18 (-8.64, 8.28) | 0.97 | 0.17 (-7, 7.35) | 0.966 | 0.42 (-5.1, 5.95) | 0.89 |
| 610.3 | Fibrosclerosis of breast | 122 | -0.64 (-30.78, 29.5) | 0.97 | 3.28 (-121.87, 128.43) | 0.963 | -0.13 (-6.27, 6.01) | 0.97 | -0.39 (-18.68, 17.9) | 0.97 | 4.62 (-50.14, 59.38) | 0.878 |
| 610.4 | Benign neoplasm of breast | 379 | 0.07 (-3.05, 3.18) | 0.97 | -0.94 (-26.54, 24.66) | 0.948 | 0.85 (-34.26, 35.96) | 0.966 | 0.09 (-4.34, 4.53) | 0.97 | -0.01 (-0.31, 0.3) | 0.97 |
| 610.8 | Other specified benign mammary dysplasias | 1067 | 0.5 (-9.7, 10.71) | 0.929 | -0.22 (-10.69, 10.24) | 0.97 | -0.5 (-23.99, 22.99) | 0.97 | -0.33 (-13.75, 13.1) | 0.966 | 0.37 (-16.01, 16.75) | 0.968 |
| 612 | Breast conditions, congenital or relating to hormones | 1503 | 0.04 (-1.73, 1.81) | 0.97 | -0.06 (-2.87, 2.75) | 0.97 | 0.49 (-19.76, 20.74) | 0.966 | -0.11 (-5.33, 5.11) | 0.97 | 0.16 (-7.24, 7.55) | 0.97 |
| 612.1 | Galactorrhea | 304 | 0.2 (-9.04, 9.43) | 0.97 | 0.03 (-1.51, 1.58) | 0.97 | -1.04 (-43.9, 41.82) | 0.966 | -0.06 (-2.85, 2.73) | 0.97 | -0.53 (-22.37, 21.31) | 0.966 |
| 613 | Other nonmalignant breast conditions | 7317 | 0.05 (-2.28, 2.38) | 0.97 | -0.21 (-9.04, 8.61) | 0.966 | -0.14 (-6.78, 6.5) | 0.97 | 0.07 (-3.12, 3.26) | 0.97 | 0.45 (-4.3, 5.19) | 0.864 |
| 613.1 | Inflammatory disease of breast | 310 | -0.51 (-24.76, 23.74) | 0.97 | -1.5 (-72.35, 69.35) | 0.97 | 0.16 (-7.58, 7.91) | 0.97 | 1 (-42.71, 44.71) | 0.967 | 0.27 (-12.35, 12.88) | 0.97 |
| 613.5 | Mastodynia | 1581 | -0.24 (-10.28, 9.79) | 0.966 | -0.07 (-3.55, 3.4) | 0.97 | 0.29 (-13.3, 13.88) | 0.97 | 0.23 (-9.11, 9.56) | 0.966 | -0.57 (-7.88, 6.73) | 0.887 |
| 613.7 | Other signs and symptoms in breast | 1868 | -0.01 (-0.33, 0.31) | 0.97 | 0.63 (-7.35, 8.61) | 0.887 | -0.69 (-19.59, 18.2) | 0.948 | -0.14 (-6.6, 6.33) | 0.97 | 0.42 (-10.95, 11.78) | 0.948 |
| 613.8 | Other specified disorders of breast | 1394 | 0.09 (-3.98, 4.16) | 0.97 | -0.58 (-13.58, 12.41) | 0.936 | 0.1 (-4.79, 5) | 0.97 | 0.12 (-5.39, 5.62) | 0.97 | 0.67 (-6.46, 7.8) | 0.864 |
| 613.9 | Breast disorder NOS | 751 | -0.01 (-0.4, 0.38) | 0.97 | -0.02 (-0.99, 0.95) | 0.97 | 2.09 (-40.72, 44.89) | 0.93 | -0.44 (-21.11, 20.24) | 0.97 | 1.31 (-25.07, 27.7) | 0.929 |
| 614 | Inflammatory diseases of female pelvic organs | 19463 | -0.19 (-4.06, 3.68) | 0.93 | 0.66 (-0.87, 2.19) | 0.404 | 0.07 (-3.47, 3.62) | 0.97 | -0.11 (-4.73, 4.51) | 0.966 | 0.1 (-4.54, 4.74) | 0.97 |
| 614.1 | Pelvic peritoneal adhesions, female (postoperative) (postinfection) | 1281 | 0.44 (-17.51, 18.38) | 0.966 | 1.64 (-12.6, 15.87) | 0.833 | -1.63 (-29.06, 25.8) | 0.915 | -0.79 (-14.13, 12.54) | 0.915 | -0.24 (-11.54, 11.06) | 0.97 |
| 614.3 | Pelvic inflammatory disease (PID) | 688 | -0.93 (-15.34, 13.48) | 0.907 | -0.69 (-32.8, 31.42) | 0.97 | 0.51 (-23.52, 24.53) | 0.97 | 1.08 (-10.42, 12.58) | 0.864 | 0.48 (-22.34, 23.31) | 0.97 |
| 614.31 | Acute inflammatory pelvic disease | 5386 | -0.26 (-5.62, 5.09) | 0.93 | 0.63 (-4.88, 6.15) | 0.833 | -0.05 (-2.19, 2.1) | 0.97 | 0.02 (-0.79, 0.82) | 0.97 | 0.44 (-5.12, 5.99) | 0.887 |
| 614.32 | Chronic inflammatory pelvic disease | 2327 | 0.1 (-4.48, 4.67) | 0.97 | 0.53 (-21.22, 22.28) | 0.966 | -0.25 (-12, 11.5) | 0.97 | -0.28 (-13.53, 12.97) | 0.97 | 0.36 (-16.69, 17.41) | 0.97 |
| 614.33 | Pelvic inflammatory disease, NOS | 554 | 0.69 (-27.56, 28.93) | 0.966 | -1.44 (-34.24, 31.35) | 0.937 | 2.42 (-63.52, 68.36) | 0.948 | -0.32 (-15.37, 14.73) | 0.97 | -0.56 (-26.85, 25.74) | 0.97 |
| 614.4 | Inflammatory diseases of uterus, except cervix | 5282 | 0.15 (-6.8, 7.09) | 0.97 | 0.36 (-9.5, 10.22) | 0.948 | -0.85 (-11.62, 9.92) | 0.887 | -0.15 (-7.4, 7.1) | 0.97 | 0.31 (-9.02, 9.65) | 0.952 |
| 614.5 | Inflammatory disease of cervix, vagina, and vulva | 534 | -0.3 (-14.68, 14.07) | 0.97 | -0.83 (-39.71, 38.05) | 0.97 | 2.82 (-33.02, 38.66) | 0.887 | 0.13 (-6.23, 6.5) | 0.97 | -0.3 (-14.56, 13.96) | 0.97 |
| 614.51 | Cervicitis and endocervicitis | 660 | -0.1 (-5.06, 4.85) | 0.97 | -0.19 (-9.4, 9.01) | 0.97 | 1 (-14.55, 16.56) | 0.907 | -0.05 (-2.53, 2.43) | 0.97 | 0.58 (-12.42, 13.58) | 0.936 |
| 614.52 | Vaginitis and vulvovaginitis | 1568 | -0.44 (-18.62, 17.74) | 0.966 | -0.39 (-18.76, 17.98) | 0.97 | -0.02 (-1.18, 1.13) | 0.97 | 0.61 (-13.34, 14.57) | 0.937 | 0.03 (-1.22, 1.27) | 0.97 |
| 614.53 | Cyst or abscess of Bartholin's gland | 2395 | -0.41 (-11.6, 10.77) | 0.948 | 1.01 (-9.64, 11.65) | 0.863 | 0.65 (-26.22, 27.53) | 0.966 | -0.19 (-9.29, 8.9) | 0.97 | 0.17 (-7.97, 8.32) | 0.97 |
| 614.54 | Abscess or ulceration of vulva | 2899 | -0.43 (-9.98, 9.13) | 0.936 | 0.94 (-9.07, 10.95) | 0.864 | 1.9 (-13.42, 17.22) | 0.82 | -0.34 (-12.64, 11.96) | 0.961 | 0.14 (-6.65, 6.94) | 0.97 |
| 615 | Endometriosis | 6640 | -0.05 (-2.26, 2.17) | 0.97 | 0.13 (-5.84, 6.09) | 0.97 | -0.08 (-3.77, 3.61) | 0.97 | 0.01 (-0.32, 0.34) | 0.97 | 0.23 (-8.64, 9.11) | 0.963 |
| 617 | Disorders secondary to childbirth, surgery, trauma | 726 | 0.14 (-6.36, 6.64) | 0.97 | 1.1 (-28.81, 31) | 0.948 | 0.33 (-15.12, 15.77) | 0.97 | -0.61 (-24.01, 22.78) | 0.963 | 0.7 (-30.02, 31.43) | 0.967 |
| 618 | Genital prolapse | 12139 | -0.08 (-4.04, 3.87) | 0.97 | 0.44 (-5.2, 6.09) | 0.887 | 0.11 (-4.96, 5.17) | 0.97 | -0.12 (-5.92, 5.67) | 0.97 | -0.05 (-2.62, 2.52) | 0.97 |
| 618.1 | Prolapse of vaginal walls | 9644 | -0.27 (-5.67, 5.14) | 0.929 | 0.58 (-5.59, 6.75) | 0.864 | -0.15 (-7.35, 7.05) | 0.97 | 0.06 (-2.77, 2.89) | 0.97 | -0.14 (-6.66, 6.38) | 0.97 |
| 618.2 | Uterine/Uterovaginal prolapse | 3351 | 0.03 (-1.54, 1.61) | 0.97 | 0.21 (-9.9, 10.33) | 0.97 | -0.04 (-2.15, 2.06) | 0.97 | -0.12 (-5.89, 5.65) | 0.97 | -0.26 (-12.65, 12.12) | 0.97 |
| 618.5 | Prolapse of vaginal vault after hysterectomy | 519 | 0.43 (-14.6, 15.45) | 0.96 | -0.53 (-22.2, 21.15) | 0.966 | -2.26 (-26.3, 21.78) | 0.864 | 0.11 (-5.03, 5.24) | 0.97 | 0.54 (-21.73, 22.81) | 0.966 |
| 618.6 | Vaginal enterocele, congenital or acquired | 2381 | 0.21 (-9.72, 10.14) | 0.97 | -0.39 (-18.67, 17.9) | 0.97 | -0.33 (-15.8, 15.15) | 0.97 | 0 (-0.04, 0.04) | 0.97 | -0.49 (-20.78, 19.79) | 0.966 |
| 619 | Noninflammatory female genital disorders | 9707 | -0.06 (-2.95, 2.83) | 0.97 | 0.75 (-2.23, 3.73) | 0.634 | 0.2 (-9.15, 9.54) | 0.97 | -0.29 (-3.99, 3.41) | 0.887 | -0.27 (-7.54, 7) | 0.948 |
| 619.1 | Noninflammatory disorders of ovary, fallopian tube, and broad ligament | 1612 | -0.34 (-15.08, 14.4) | 0.967 | 0.19 (-8.59, 8.96) | 0.97 | 0.51 (-23.67, 24.69) | 0.97 | 0.17 (-8.03, 8.38) | 0.97 | -1.01 (-13.79, 11.78) | 0.887 |
| 619.2 | Disorders of uterus, NEC | 1852 | 0.32 (-12.91, 13.55) | 0.966 | 0.43 (-18.28, 19.14) | 0.967 | 1.56 (-15.02, 18.13) | 0.864 | -0.75 (-7.25, 5.75) | 0.833 | -0.48 (-17.64, 16.68) | 0.96 |
| 619.3 | Noninflammatory disorders of cervix | 3229 | 0.09 (-4.11, 4.29) | 0.97 | 0.47 (-5.47, 6.4) | 0.887 | -0.01 (-0.37, 0.35) | 0.97 | -0.3 (-4.17, 3.56) | 0.887 | 0.23 (-9.16, 9.61) | 0.966 |
| 619.4 | Noninflammatory disorders of vagina | 1205 | -0.68 (-16.18, 14.81) | 0.937 | 2.02 (-15.6, 19.65) | 0.833 | -1.61 (-34.83, 31.61) | 0.931 | 0.28 (-12.83, 13.38) | 0.97 | 1.21 (-14.23, 16.66) | 0.887 |
| 619.5 | Noninflammatory disorders of vulva and perineum | 1025 | 0.21 (-9.79, 10.22) | 0.97 | 0.19 (-8.97, 9.36) | 0.97 | -1.29 (-25.39, 22.81) | 0.923 | -0.07 (-3.15, 3.02) | 0.97 | -0.3 (-14.43, 13.84) | 0.97 |
| 620 | Dysplasia of female genital organs | 682 | 0.13 (-6.22, 6.48) | 0.97 | 0.1 (-4.47, 4.67) | 0.97 | 2.81 (-21.61, 27.22) | 0.833 | -0.52 (-14.77, 13.72) | 0.948 | -0.26 (-12.66, 12.14) | 0.97 |
| 621 | Endometrial hyperplasia | 1016 | 0.22 (-10.19, 10.63) | 0.97 | 0.68 (-31.53, 32.89) | 0.97 | -1.61 (-67.94, 64.72) | 0.966 | -0.32 (-15.28, 14.65) | 0.97 | -0.43 (-20.72, 19.86) | 0.97 |
| 622 | Polyp of female genital organs | 9045 | 0.09 (-4.31, 4.49) | 0.97 | 0.32 (-6.27, 6.91) | 0.93 | -0.12 (-5.85, 5.61) | 0.97 | -0.21 (-4.45, 4.04) | 0.931 | 0.09 (-4.14, 4.32) | 0.97 |
| 622.1 | Polyp of corpus uteri | 7801 | 0.15 (-6.17, 6.47) | 0.966 | 0.37 (-7.05, 7.79) | 0.929 | -0.01 (-0.56, 0.54) | 0.97 | -0.31 (-3.56, 2.95) | 0.864 | -0.02 (-1.07, 1.03) | 0.97 |
| 622.2 | Mucous polyp of cervix | 1401 | 0.03 (-1.6, 1.67) | 0.97 | -0.43 (-18.26, 17.4) | 0.966 | -0.36 (-17.45, 16.73) | 0.97 | 0.23 (-10.44, 10.9) | 0.97 | 0.32 (-13.73, 14.37) | 0.967 |
| 623 | Hypertrophy of female genital organs | 953 | -0.07 (-3.49, 3.35) | 0.97 | -0.4 (-19.2, 18.4) | 0.97 | 0.79 (-31.84, 33.43) | 0.966 | 0.1 (-4.44, 4.64) | 0.97 | -0.11 (-5.51, 5.28) | 0.97 |
| 624 | Symptoms involving female genital tract | 5565 | 0.01 (-0.27, 0.28) | 0.97 | -0.12 (-5.98, 5.73) | 0.97 | -0.45 (-12.67, 11.77) | 0.948 | 0.13 (-5.34, 5.59) | 0.967 | -0.23 (-6.62, 6.15) | 0.948 |
| 624.1 | Dystrophy of female genital tract | 109 | 1.43 (-66.4, 69.27) | 0.97 | -1.59 (-76.64, 73.46) | 0.97 | -2.98 (-143.69, 137.74) | 0.97 | 0.02 (-1.06, 1.1) | 0.97 | -2.59 (-109.16, 103.99) | 0.966 |
| 624.9 | stress incontinence, female | 5355 | 0 (-0.21, 0.2) | 0.97 | -0.09 (-4.56, 4.37) | 0.97 | -0.46 (-12.96, 12.04) | 0.948 | 0.12 (-5.29, 5.54) | 0.967 | -0.19 (-8.18, 7.79) | 0.966 |
| 625 | Pain and other symptoms associated with female genital organs | 4332 | 0.44 (-5.17, 6.05) | 0.887 | 0.69 (-8.09, 9.47) | 0.887 | -0.57 (-24.18, 23.03) | 0.966 | -0.62 (-5.65, 4.4) | 0.82 | 0.09 (-4.21, 4.39) | 0.97 |
| 625.1 | Dyspareunia | 2865 | 0.2 (-9.25, 9.65) | 0.97 | 0.27 (-12.64, 13.19) | 0.97 | 0.07 (-3.46, 3.61) | 0.97 | -0.33 (-14.03, 13.36) | 0.966 | 0.18 (-8.19, 8.54) | 0.97 |
| 626 | Disorders of menstruation and other abnormal bleeding from female genital tract | 46885 | -0.06 (-2.39, 2.28) | 0.966 | -0.04 (-1.81, 1.73) | 0.97 | 0.13 (-5.38, 5.63) | 0.967 | 0.05 (-2.18, 2.28) | 0.968 | 0.12 (-2.51, 2.74) | 0.936 |
| 626.1 | Irregular menstrual cycle/bleeding | 2558 | -0.17 (-8, 7.67) | 0.97 | 0.7 (-13.32, 14.72) | 0.929 | -0.91 (-25.73, 23.9) | 0.948 | 0.03 (-1.47, 1.54) | 0.97 | 0.03 (-1.61, 1.68) | 0.97 |
| 626.11 | Absent or infrequent menstruation | 1568 | -0.27 (-12.19, 11.65) | 0.967 | 0.28 (-13.17, 13.74) | 0.97 | -1.3 (-17.85, 15.24) | 0.887 | 0.38 (-9.85, 10.6) | 0.948 | 0.29 (-13.51, 14.09) | 0.97 |
| 626.12 | Excessive or frequent menstruation | 10504 | 0.06 (-2.74, 2.85) | 0.97 | 0.25 (-4.97, 5.47) | 0.932 | 0.33 (-8.71, 9.38) | 0.948 | -0.22 (-2.56, 2.12) | 0.864 | 0.32 (-3.07, 3.71) | 0.864 |
| 626.13 | Irregular menstrual cycle | 3044 | -0.11 (-5.27, 5.05) | 0.97 | -0.5 (-14.17, 13.16) | 0.948 | 0.28 (-12.82, 13.38) | 0.97 | 0.27 (-10.75, 11.29) | 0.966 | -0.3 (-13.57, 12.96) | 0.967 |
| 626.14 | Irregular menstrual bleeding | 17006 | -0.07 (-3.36, 3.22) | 0.97 | 0.22 (-5.68, 6.11) | 0.948 | -0.11 (-5.39, 5.17) | 0.97 | 0 (-0.01, 0.01) | 0.97 | -0.04 (-1.8, 1.73) | 0.97 |
| 626.15 | Infertility, female, associated with anovulation | 1056 | -0.15 (-7.37, 7.07) | 0.97 | -0.02 (-0.9, 0.86) | 0.97 | 0.61 (-12.98, 14.19) | 0.936 | 0.04 (-1.93, 2.01) | 0.97 | -0.09 (-4.58, 4.39) | 0.97 |
| 626.2 | Dysmenorrhea | 3507 | 0.21 (-8.51, 8.93) | 0.966 | -0.75 (-7.31, 5.8) | 0.833 | -0.61 (-17.17, 15.95) | 0.948 | 0.21 (-8.42, 8.84) | 0.966 | 0.35 (-9.2, 9.9) | 0.948 |
| 626.21 | Mittelschmerz | 940 | 0.02 (-0.76, 0.79) | 0.97 | 0.5 (-21.37, 22.37) | 0.968 | 0.48 (-22.15, 23.11) | 0.97 | -0.32 (-15.63, 14.98) | 0.97 | -0.87 (-18.8, 17.05) | 0.93 |
| 626.8 | Infertility, female | 16184 | -0.05 (-2.06, 1.97) | 0.968 | -0.09 (-3.72, 3.55) | 0.966 | 0.17 (-4.46, 4.8) | 0.948 | 0.06 (-2.25, 2.36) | 0.966 | 0.1 (-2.55, 2.74) | 0.948 |
| 627 | Menopausal and postmenopausal disorders | 13132 | -0.13 (-5.6, 5.34) | 0.967 | 0.24 (-9.75, 10.24) | 0.966 | 0.6 (-11.37, 12.56) | 0.929 | -0.06 (-3.02, 2.9) | 0.97 | 0.41 (-3.92, 4.74) | 0.864 |
| 627.1 | Postmenopausal bleeding | 6341 | 0.21 (-7.67, 8.08) | 0.963 | -0.69 (-6.7, 5.32) | 0.833 | -0.34 (-16.18, 15.51) | 0.97 | 0.12 (-5.45, 5.68) | 0.97 | 0.34 (-7.45, 8.13) | 0.938 |
| 627.2 | Symptomatic menopause | 276 | -0.13 (-6.05, 5.8) | 0.97 | -0.45 (-21.82, 20.91) | 0.97 | 6.41 (-45.26, 58.08) | 0.82 | -0.51 (-24.48, 23.47) | 0.97 | 1.46 (-50.02, 52.94) | 0.96 |
| 627.3 | Postmenopausal atrophic vaginitis | 530 | -1.83 (-8.06, 4.4) | 0.576 | 1.07 (-28.14, 30.28) | 0.948 | 2.38 (-47.51, 52.27) | 0.932 | 1.09 (-10.58, 12.75) | 0.865 | -0.14 (-6.96, 6.67) | 0.97 |
| 627.4 | Premenopausal menorrhagia | 6244 | -0.06 (-3.02, 2.89) | 0.97 | 0.02 (-1.09, 1.14) | 0.97 | 0.56 (-5.43, 6.55) | 0.864 | -0.04 (-1.7, 1.63) | 0.97 | 0.53 (-0.85, 1.91) | 0.457 |
| 627.5 | Premature menopause and other ovarian failure | 184 | 0.61 (-28.46, 29.69) | 0.97 | 0.11 (-4.94, 5.16) | 0.97 | 0.15 (-6.98, 7.28) | 0.97 | -0.68 (-30.76, 29.39) | 0.968 | -0.29 (-14.13, 13.54) | 0.97 |
| 628 | Ovarian cyst | 12517 | -0.1 (-4.69, 4.49) | 0.97 | 0.3 (-7.77, 8.36) | 0.948 | 0.1 (-4.76, 4.96) | 0.97 | -0.04 (-2.08, 1.99) | 0.97 | 0.27 (-7.13, 7.68) | 0.948 |
| 643 | Excessive vomiting in pregnancy | 4315 | 0.05 (-2.12, 2.21) | 0.97 | -0.13 (-6.43, 6.16) | 0.97 | -0.02 (-0.96, 0.92) | 0.97 | 0.03 (-1.28, 1.34) | 0.97 | 0.01 (-0.62, 0.64) | 0.97 |
| 643.1 | Hyperemesis gravidarum | 3559 | 0.01 (-0.41, 0.43) | 0.97 | 0.05 (-2.52, 2.63) | 0.97 | -0.06 (-3.05, 2.92) | 0.97 | -0.02 (-1.16, 1.11) | 0.97 | -0.47 (-2.63, 1.68) | 0.68 |
| 644 | Anemia during pregnancy | 8586 | -0.24 (-2.16, 1.68) | 0.82 | 0.41 (-1.22, 2.04) | 0.634 | -0.3 (-6.61, 6.01) | 0.932 | 0.11 (-2.96, 3.19) | 0.948 | 0.03 (-1.38, 1.44) | 0.97 |
| 650 | Normal delivery | 91121 | -0.03 (-1.13, 1.08) | 0.965 | 0 (-0.02, 0.02) | 0.97 | -0.14 (-1.37, 1.08) | 0.833 | 0.05 (-0.52, 0.62) | 0.864 | -0.13 (-0.39, 0.14) | 0.353 |
| 686 | Other local infections of skin and subcutaneous tissue | 37995 | 0.01 (-0.52, 0.54) | 0.97 | 0.23 (-2.75, 3.22) | 0.887 | -0.03 (-1.39, 1.33) | 0.97 | -0.11 (-3.07, 2.85) | 0.948 | 0.06 (-2.56, 2.67) | 0.97 |
| 686.1 | Carbuncle and furuncle | 19893 | 0.01 (-0.37, 0.39) | 0.97 | 0.24 (-5.1, 5.58) | 0.936 | -0.06 (-2.68, 2.57) | 0.97 | -0.1 (-4.35, 4.14) | 0.966 | 0.03 (-1.49, 1.55) | 0.97 |
| 686.2 | Impetigo | 1465 | -0.22 (-10.85, 10.4) | 0.97 | 0.68 (-27.26, 28.62) | 0.966 | 0.18 (-8.31, 8.66) | 0.97 | -0.09 (-4.56, 4.37) | 0.97 | -0.32 (-15.39, 14.75) | 0.97 |
| 686.3 | Pilonidal cyst | 4854 | 0.03 (-1.29, 1.35) | 0.97 | 0.03 (-1.24, 1.3) | 0.97 | 1.1 (-10.64, 12.85) | 0.864 | -0.26 (-7.28, 6.77) | 0.948 | -0.15 (-7.38, 7.07) | 0.97 |
| 686.4 | Pyogenic granuloma | 409 | 0.28 (-12.94, 13.5) | 0.97 | 1.16 (-49.3, 51.61) | 0.967 | -7.61 (-31.14, 15.92) | 0.537 | 0.46 (-21.46, 22.39) | 0.97 | -0.33 (-16.09, 15.42) | 0.97 |
| 686.5 | Pyoderma | 229 | 1.47 (-53.12, 56.05) | 0.962 | -1.02 (-49.31, 47.27) | 0.97 | 0.93 (-42.82, 44.67) | 0.97 | -1.23 (-52.1, 49.63) | 0.966 | -1.59 (-67.17, 63.99) | 0.966 |
| 687 | Symptoms affecting skin | 6418 | -0.24 (-3.42, 2.93) | 0.89 | 0.13 (-5.97, 6.23) | 0.97 | 0.25 (-11.47, 11.96) | 0.97 | 0.15 (-6.15, 6.45) | 0.966 | 0.16 (-6.79, 7.11) | 0.967 |
| 687.1 | Rash and other nonspecific skin eruption | 841 | 0.49 (-10.58, 11.55) | 0.937 | 0.51 (-21, 22.02) | 0.966 | -1.03 (-43.41, 41.35) | 0.966 | -0.54 (-11.59, 10.51) | 0.93 | -0.34 (-16.24, 15.57) | 0.97 |
| 687.2 | Localized superficial swelling, mass, or lump | 612 | -0.66 (-17.87, 16.54) | 0.945 | -0.8 (-31.28, 29.68) | 0.963 | 0.24 (-11.03, 11.5) | 0.97 | 1.06 (-10.19, 12.3) | 0.864 | 0.63 (-25.41, 26.68) | 0.966 |
| 687.4 | Disturbance of skin sensation | 1887 | -0.54 (-6.33, 5.24) | 0.864 | 0.21 (-9.9, 10.33) | 0.97 | 0.95 (-18.56, 20.45) | 0.931 | 0.32 (-8.26, 8.89) | 0.948 | -0.15 (-7.11, 6.81) | 0.97 |
| 689 | Disorder of skin and subcutaneous tissue NOS | 1549 | -0.1 (-4.86, 4.66) | 0.97 | -0.55 (-24.43, 23.33) | 0.967 | 0.35 (-16.32, 17.02) | 0.97 | 0.28 (-12.9, 13.45) | 0.97 | 0.58 (-23.45, 24.62) | 0.966 |
| 690 | Erythematosquamous dermatosis | 1588 | 0 (-0.22, 0.23) | 0.97 | 0.43 (-19.79, 20.64) | 0.97 | -0.71 (-34.09, 32.67) | 0.97 | -0.07 (-3.44, 3.29) | 0.97 | 0.1 (-4.59, 4.79) | 0.97 |
| 690.1 | Seborrheic dermatitis | 1408 | -0.1 (-4.7, 4.51) | 0.97 | 0.46 (-21.06, 21.97) | 0.97 | -0.61 (-29.4, 28.18) | 0.97 | 0 (-0.02, 0.02) | 0.97 | -0.04 (-2.14, 2.06) | 0.97 |
| 694 | Dyschromia and Vitiligo | 2105 | -0.52 (-7.07, 6.04) | 0.887 | -0.26 (-12.41, 11.89) | 0.97 | -0.05 (-2.49, 2.39) | 0.97 | 0.63 (-6.11, 7.38) | 0.864 | -0.25 (-11.92, 11.43) | 0.97 |
| 694.2 | Other dyschromia | 1282 | -0.53 (-15.05, 13.98) | 0.948 | -0.37 (-17.65, 16.91) | 0.97 | 0.27 (-12.59, 13.13) | 0.97 | 0.65 (-12.33, 13.63) | 0.929 | 0.05 (-2.1, 2.19) | 0.97 |
| 694.3 | Vascular disorders of skin | 833 | -0.37 (-15.57, 14.84) | 0.966 | 0.11 (-5.21, 5.43) | 0.97 | -1.01 (-28.6, 26.57) | 0.948 | 0.51 (-10.04, 11.06) | 0.931 | -0.64 (-14.95, 13.67) | 0.936 |
| 695 | Erythematous conditions | 7111 | -0.25 (-7.02, 6.53) | 0.948 | 0.48 (-8.58, 9.55) | 0.923 | -0.52 (-14.7, 13.66) | 0.948 | 0.14 (-6.61, 6.9) | 0.97 | 0.03 (-1.45, 1.51) | 0.97 |
| 695.1 | Toxic erythema | 717 | -0.31 (-15.17, 14.54) | 0.97 | 2.26 (-26.49, 31.01) | 0.887 | -5.33 (-51.72, 41.06) | 0.833 | 0.1 (-4.78, 4.98) | 0.97 | -0.44 (-21.07, 20.2) | 0.97 |
| 695.2 | Bullous dermatoses | 130 | -3.13 (-18.62, 12.35) | 0.705 | 1.58 (-63.58, 66.75) | 0.966 | 2.69 (-108.32, 113.71) | 0.966 | 2.06 (-24.11, 28.22) | 0.887 | 2.23 (-43.46, 47.91) | 0.93 |
| 695.21 | Dermatitis herpetiformis | 157 | -1.33 (-59.88, 57.22) | 0.968 | 2.23 (-89.6, 94.05) | 0.966 | -9.53 (-187.56, 168.51) | 0.923 | 1.13 (-52.21, 54.46) | 0.97 | 2.24 (-90.05, 94.53) | 0.966 |
| 695.22 | Pemphigus and pemphigoid | 445 | 0.95 (-18.13, 20.03) | 0.929 | -1.18 (-33.36, 31) | 0.948 | 1.19 (-54.28, 56.65) | 0.97 | -0.7 (-25.3, 23.9) | 0.96 | 2.16 (-16.6, 20.91) | 0.833 |
| 695.3 | Rosacea | 1581 | -0.08 (-4.04, 3.88) | 0.97 | 0.17 (-7.94, 8.28) | 0.97 | -0.36 (-17.15, 16.44) | 0.97 | 0.08 (-3.92, 4.09) | 0.97 | -0.43 (-15.83, 14.98) | 0.961 |
| 695.41 | Cutaneous lupus erythematosus | 493 | 0.18 (-8.31, 8.66) | 0.97 | 0.85 (-34.27, 35.97) | 0.966 | -0.28 (-13.63, 13.07) | 0.97 | -0.53 (-22.97, 21.9) | 0.966 | -0.85 (-35.71, 34.01) | 0.966 |
| 695.42 | Systemic lupus erythematosus | 792 | -0.76 (-21.38, 19.87) | 0.948 | 0.46 (-21.2, 22.12) | 0.97 | -0.03 (-1.35, 1.3) | 0.97 | 0.56 (-22.41, 23.52) | 0.966 | -0.8 (-33.81, 32.21) | 0.966 |
| 695.7 | Prurigo and Lichen | 2218 | -0.24 (-10.93, 10.44) | 0.967 | -0.28 (-13.42, 12.86) | 0.97 | 0.07 (-3.13, 3.26) | 0.97 | 0.36 (-9.38, 10.09) | 0.948 | 0.79 (-7.66, 9.25) | 0.864 |
| 695.8 | Other specified erythematous conditions | 622 | -0.79 (-17.5, 15.92) | 0.932 | 0.75 (-30.03, 31.53) | 0.966 | -3.01 (-27.28, 21.26) | 0.82 | 1.2 (-11.55, 13.94) | 0.864 | -1.35 (-18.56, 15.85) | 0.887 |
| 695.9 | Unspecified erythematous condition | 452 | 0.51 (-23.39, 24.4) | 0.97 | 0.79 (-36.44, 38.01) | 0.97 | -2.99 (-70.84, 64.86) | 0.937 | -0.28 (-13.3, 12.75) | 0.97 | -0.9 (-43.29, 41.5) | 0.97 |
| 696 | Psoriasis and related disorders | 5687 | -0.25 (-7.16, 6.66) | 0.948 | 0.54 (-8.6, 9.69) | 0.915 | 0.17 (-7.93, 8.27) | 0.97 | 0 (-0.05, 0.05) | 0.97 | 0.17 (-7.74, 8.07) | 0.97 |
| 696.2 | Parapsoriasis | 143 | -2.11 (-28.88, 24.67) | 0.887 | -3.07 (-55.1, 48.97) | 0.915 | 2.82 (-115.7, 121.34) | 0.966 | 2.93 (-20.71, 26.58) | 0.82 | -3.89 (-35.19, 27.42) | 0.82 |
| 696.4 | Psoriasis | 2302 | -0.48 (-13.46, 12.51) | 0.948 | 1.05 (-16.67, 18.78) | 0.915 | 0.42 (-19.54, 20.38) | 0.97 | -0.02 (-0.83, 0.8) | 0.97 | 0.31 (-14.55, 15.18) | 0.97 |
| 696.41 | Psoriasis vulgaris | 4381 | -0.28 (-6.11, 5.55) | 0.931 | 0.6 (-5.81, 7.01) | 0.864 | -0.06 (-3.08, 2.95) | 0.97 | 0.05 (-2.21, 2.31) | 0.97 | 0.26 (-10.34, 10.86) | 0.966 |
| 696.42 | Psoriatic arthropathy | 1807 | 0.32 (-12.93, 13.57) | 0.966 | 0.15 (-6.98, 7.28) | 0.97 | -0.7 (-29.48, 28.08) | 0.966 | -0.27 (-11.89, 11.36) | 0.967 | 0.24 (-11.22, 11.71) | 0.97 |
| 697 | Sarcoidosis | 1852 | -0.79 (-10.85, 9.27) | 0.887 | 0.75 (-27.2, 28.7) | 0.962 | 1.32 (-34.61, 37.25) | 0.948 | 0.26 (-12.1, 12.63) | 0.97 | -0.72 (-20.27, 18.83) | 0.948 |
| 698 | Pruritus and related conditions | 1602 | -0.63 (-7.38, 6.11) | 0.864 | 1.2 (-8.48, 10.88) | 0.82 | 0.38 (-17.44, 18.19) | 0.97 | 0 (-0.12, 0.13) | 0.97 | 0.34 (-15.64, 16.32) | 0.97 |
| 701 | Other hypertrophic and atrophic conditions of skin | 6705 | 0.12 (-5.37, 5.61) | 0.97 | 0.25 (-10.02, 10.52) | 0.966 | -0.45 (-12.56, 11.67) | 0.948 | -0.14 (-6.01, 5.73) | 0.966 | 0.23 (-9.09, 9.54) | 0.966 |
| 701.2 | Scar conditions and fibrosis of skin | 3627 | -0.32 (-4.35, 3.71) | 0.887 | 0.39 (-7.79, 8.57) | 0.932 | -0.05 (-2.26, 2.17) | 0.97 | 0.16 (-6.55, 6.87) | 0.966 | 0.44 (-5.13, 6.01) | 0.887 |
| 701.3 | Circumscribed scleroderma | 225 | 0.64 (-29.77, 31.05) | 0.97 | -2.58 (-54.52, 49.35) | 0.929 | 1.76 (-81.59, 85.11) | 0.97 | 0.07 (-3.19, 3.33) | 0.97 | -0.31 (-15.09, 14.47) | 0.97 |
| 701.4 | Keloid scar | 761 | 0.15 (-6.72, 7.01) | 0.97 | 1.54 (-20.57, 23.64) | 0.9 | -0.23 (-11.16, 10.69) | 0.97 | -0.71 (-20, 18.59) | 0.948 | -0.95 (-34.55, 32.65) | 0.96 |
| 701.5 | Abnormal granulation tissue | 539 | -0.07 (-3.23, 3.1) | 0.97 | -0.78 (-32.86, 31.3) | 0.966 | -0.23 (-11.28, 10.81) | 0.97 | 0.48 (-19.21, 20.16) | 0.966 | -0.05 (-2.39, 2.29) | 0.97 |
| 702 | Degenerative skin conditions and other dermatoses | 3459 | 0.07 (-3.28, 3.42) | 0.97 | 0.21 (-9.5, 9.91) | 0.97 | -0.94 (-10.94, 9.06) | 0.864 | 0.01 (-0.54, 0.57) | 0.97 | -0.42 (-6.43, 5.6) | 0.9 |
| 702.1 | Actinic keratosis | 3307 | -0.01 (-0.59, 0.56) | 0.97 | 0.27 (-10.85, 11.38) | 0.966 | -1.08 (-9.81, 7.64) | 0.82 | 0.09 (-4.34, 4.53) | 0.97 | -0.5 (-5.77, 4.78) | 0.864 |
| 703 | Diseases of nail, NOS | 2821 | -0.08 (-3.77, 3.62) | 0.97 | 0.03 (-1.62, 1.69) | 0.97 | 0.09 (-4.23, 4.41) | 0.97 | 0.05 (-2.27, 2.37) | 0.97 | 0.13 (-5.9, 6.15) | 0.97 |
| 703.1 | Ingrowing nail | 2257 | 0.16 (-7.48, 7.81) | 0.97 | -0.12 (-5.67, 5.43) | 0.97 | -0.01 (-0.54, 0.52) | 0.97 | -0.12 (-5.62, 5.38) | 0.97 | 0.12 (-5.42, 5.65) | 0.97 |
| 704 | Diseases of hair and hair follicles | 4104 | -0.07 (-3.44, 3.3) | 0.97 | 0.55 (-10.74, 11.84) | 0.93 | 0.16 (-7.59, 7.92) | 0.97 | -0.24 (-9.92, 9.45) | 0.966 | -0.06 (-2.72, 2.61) | 0.97 |
| 704.1 | Alopecia | 394 | -1.09 (-19.57, 17.39) | 0.915 | 0.27 (-12.39, 12.93) | 0.97 | 2.72 (-70.73, 76.17) | 0.947 | 0.69 (-27.7, 29.07) | 0.966 | 0.79 (-31.65, 33.22) | 0.966 |
| 704.11 | Alopecia Areata | 363 | 0.13 (-6.19, 6.45) | 0.97 | 0.98 (-39.58, 41.54) | 0.966 | -0.23 (-10.99, 10.53) | 0.97 | -0.65 (-27.3, 26.01) | 0.966 | 0.55 (-25.25, 26.34) | 0.97 |
| 704.2 | Hirsutism | 1779 | -0.02 (-0.93, 0.89) | 0.97 | 0.13 (-5.93, 6.19) | 0.97 | 0.54 (-21.76, 22.84) | 0.966 | -0.18 (-8.6, 8.25) | 0.97 | -0.25 (-12.23, 11.72) | 0.97 |
| 704.8 | Other specified diseases of hair and hair follicles | 772 | 0.17 (-8.08, 8.43) | 0.97 | 0.23 (-10.64, 11.1) | 0.97 | 1.47 (-31.48, 34.42) | 0.936 | -0.53 (-14.97, 13.91) | 0.948 | -0.01 (-0.49, 0.47) | 0.97 |
| 705 | Disorders of sweat glands | 3352 | -0.28 (-11.68, 11.12) | 0.966 | 0.15 (-7.05, 7.36) | 0.97 | -0.45 (-21.65, 20.75) | 0.97 | 0.3 (-12.02, 12.62) | 0.966 | 0.35 (-14.19, 14.88) | 0.966 |
| 705.3 | Hidradenitis | 1339 | 0.23 (-10.44, 10.89) | 0.97 | 0.01 (-0.65, 0.68) | 0.97 | -0.47 (-22.59, 21.65) | 0.97 | -0.14 (-6.81, 6.52) | 0.97 | 0.88 (-17.26, 19.02) | 0.931 |
| 705.8 | Hyperhidrosis | 1816 | -0.32 (-14.29, 13.65) | 0.967 | -0.04 (-1.85, 1.77) | 0.97 | -0.86 (-36.5, 34.77) | 0.966 | 0.48 (-12.66, 13.63) | 0.948 | -0.16 (-7.76, 7.44) | 0.97 |
| 706 | Diseases of sebaceous glands | 3837 | -0.09 (-4.58, 4.39) | 0.97 | 1 (-7.67, 9.66) | 0.833 | 0.05 (-2.42, 2.53) | 0.97 | -0.34 (-9.71, 9.02) | 0.948 | 0.45 (-13.38, 14.27) | 0.954 |
| 706.1 | Acne | 1224 | -0.13 (-6.37, 6.11) | 0.97 | 0.84 (-16, 17.68) | 0.929 | -0.35 (-16.68, 15.99) | 0.97 | -0.18 (-8.48, 8.13) | 0.97 | 0.68 (-14.46, 15.81) | 0.936 |
| 706.2 | Sebaceous cyst | 2477 | -0.09 (-4.12, 3.95) | 0.97 | 1.11 (-13.01, 15.23) | 0.887 | 0.69 (-31.87, 33.25) | 0.97 | -0.5 (-14.19, 13.19) | 0.948 | 0.32 (-15.01, 15.66) | 0.97 |
| 706.8 | Other specified diseases of sebaceous glands | 165 | 0.5 (-23.18, 24.18) | 0.97 | -1.4 (-30.22, 27.41) | 0.93 | -2.08 (-87.9, 83.74) | 0.966 | 0.48 (-22.31, 23.28) | 0.97 | 0.67 (-30.89, 32.23) | 0.97 |
| 707 | Chronic ulcer of skin | 2122 | -0.2 (-9.83, 9.43) | 0.97 | -0.59 (-12.72, 11.53) | 0.93 | -0.89 (-23.7, 21.92) | 0.944 | 0.64 (-4.91, 6.18) | 0.833 | 0.53 (-10.59, 11.65) | 0.932 |
| 709 | Diffuse diseases of connective tissue | 7300 | -0.39 (-3.78, 3) | 0.833 | 0.35 (-7.41, 8.11) | 0.936 | 1.02 (-7.2, 9.24) | 0.82 | 0.09 (-4.25, 4.43) | 0.97 | -0.1 (-4.69, 4.5) | 0.97 |
| 709.2 | Sicca syndrome | 1150 | -0.42 (-17.87, 17.02) | 0.966 | 0.22 (-10.34, 10.78) | 0.97 | 0.74 (-34.13, 35.61) | 0.97 | 0.23 (-10.46, 10.91) | 0.97 | 0.29 (-13.35, 13.93) | 0.97 |
| 709.3 | Systemic sclerosis | 393 | -1.26 (-23.69, 21.17) | 0.919 | 1.65 (-43.19, 46.48) | 0.948 | -2.39 (-67.34, 62.56) | 0.948 | 1.06 (-22.8, 24.93) | 0.936 | -1.53 (-32.36, 29.29) | 0.929 |
| 709.4 | Polymyositis | 172 | -0.18 (-8.86, 8.49) | 0.97 | 0.44 (-20.26, 21.13) | 0.97 | 1.08 (-49.98, 52.14) | 0.97 | -0.25 (-11.94, 11.44) | 0.97 | -0.79 (-37.98, 36.41) | 0.97 |
| 709.5 | Dermatomyositis | 154 | -1.56 (-57.77, 54.66) | 0.961 | 0.73 (-33.8, 35.26) | 0.97 | 8.78 (-61.95, 79.5) | 0.82 | -0.36 (-17.14, 16.43) | 0.97 | -0.71 (-34.48, 33.05) | 0.97 |
| 709.6 | Other specified diffuse diseases of connective tissue | 245 | -0.26 (-12.7, 12.18) | 0.97 | -1.11 (-46.93, 44.71) | 0.966 | 2.17 (-87.44, 91.79) | 0.966 | 0.51 (-23.47, 24.48) | 0.97 | -0.64 (-31.1, 29.82) | 0.97 |
| 709.7 | Unspecified diffuse connective tissue disease | 5544 | -0.31 (-4.22, 3.61) | 0.887 | 0.35 (-9.25, 9.96) | 0.948 | 0.98 (-9.04, 11) | 0.858 | 0.01 (-0.53, 0.55) | 0.97 | 0 (-0.15, 0.14) | 0.97 |
| 710 | Osteomyelitis, periostitis, and other infections involving bone | 2074 | -0.62 (-13.33, 12.1) | 0.93 | 1.26 (-12.15, 14.68) | 0.864 | 0.49 (-22.72, 23.7) | 0.97 | 0.03 (-1.2, 1.26) | 0.97 | 0.51 (-20.56, 21.58) | 0.966 |
| 710.11 | Acute osteomyelitis | 358 | 0.74 (-15.72, 17.19) | 0.936 | -0.62 (-30.05, 28.81) | 0.97 | -0.22 (-10.84, 10.39) | 0.97 | -0.48 (-20.12, 19.16) | 0.966 | 0.41 (-18.81, 19.62) | 0.97 |
| 710.12 | Chronic osteomyelitis | 591 | 0.07 (-3.11, 3.25) | 0.97 | 0.82 (-21.5, 23.14) | 0.948 | -2.09 (-20.3, 16.12) | 0.833 | 0.06 (-2.57, 2.69) | 0.97 | 0.48 (-20.51, 21.47) | 0.967 |
| 710.19 | Unspecified osteomyelitis | 1121 | -1.28 (-15.04, 12.47) | 0.865 | 2.36 (-22.39, 27.11) | 0.862 | 1.4 (-59.88, 62.69) | 0.967 | 0.12 (-5.33, 5.56) | 0.97 | 0.62 (-28.6, 29.83) | 0.97 |
| 711 | Arthropathy associated with infections | 2483 | 0.1 (-4.47, 4.66) | 0.97 | 1.28 (-12.38, 14.95) | 0.864 | 0.02 (-0.9, 0.94) | 0.97 | -0.61 (-12.93, 11.7) | 0.929 | 0.12 (-5.5, 5.74) | 0.97 |
| 711.1 | Pyogenic arthritis | 1439 | -0.12 (-5.65, 5.42) | 0.97 | 1.99 (-15.36, 19.35) | 0.833 | -1.08 (-45.6, 43.44) | 0.966 | -0.41 (-18.31, 17.5) | 0.968 | 0.18 (-8.26, 8.61) | 0.97 |
| 711.2 | Reiter's disease | 704 | 0.01 (-0.55, 0.58) | 0.97 | -0.63 (-30.51, 29.25) | 0.97 | 2.2 (-47.11, 51.5) | 0.936 | -0.23 (-11.14, 10.68) | 0.97 | 0.24 (-11.31, 11.8) | 0.97 |
| 713 | Arthropathy associated with other disorders classified elsewhere | 3670 | -0.06 (-2.91, 2.79) | 0.97 | 0.11 (-5.12, 5.34) | 0.97 | -0.78 (-16.49, 14.93) | 0.929 | 0.15 (-7.01, 7.31) | 0.97 | -0.05 (-2.2, 2.1) | 0.97 |
| 713.5 | Arthropathy associated with neurological disorders | 357 | 0.83 (-16.52, 18.17) | 0.932 | -0.85 (-36.46, 34.77) | 0.966 | -2.89 (-32.33, 26.55) | 0.858 | 0.05 (-2.39, 2.49) | 0.97 | -0.84 (-35.58, 33.9) | 0.966 |
| 714 | Rheumatoid arthritis and other inflammatory polyarthropathies | 8313 | 0.08 (-3.49, 3.64) | 0.97 | 0.16 (-7.42, 7.74) | 0.97 | -0.13 (-6.18, 5.92) | 0.97 | -0.12 (-5.69, 5.45) | 0.97 | -0.38 (-8.12, 7.35) | 0.929 |
| 714.1 | Rheumatoid arthritis | 7303 | 0.09 (-3.95, 4.12) | 0.97 | 0.08 (-3.66, 3.82) | 0.97 | 0.11 (-5.14, 5.36) | 0.97 | -0.14 (-6.67, 6.39) | 0.97 | -0.41 (-8.03, 7.22) | 0.923 |
| 714.2 | Juvenile rheumatoid arthritis | 795 | -0.27 (-13.01, 12.47) | 0.97 | 0.64 (-29.43, 30.7) | 0.97 | 0.33 (-15.4, 16.06) | 0.97 | -0.03 (-1.63, 1.57) | 0.97 | -0.51 (-24.41, 23.4) | 0.97 |
| 715 | Other inflammatory spondylopathies | 1494 | -0.32 (-9.01, 8.37) | 0.948 | 0.01 (-0.28, 0.29) | 0.97 | 0.45 (-20.79, 21.68) | 0.97 | 0.25 (-9.87, 10.36) | 0.966 | -0.38 (-16.04, 15.28) | 0.966 |
| 715.1 | Sacroiliitis NEC | 366 | -0.07 (-3.22, 3.09) | 0.97 | -0.15 (-7.33, 7.03) | 0.97 | 0.53 (-24.51, 25.56) | 0.97 | 0.02 (-1.13, 1.18) | 0.97 | 0.86 (-34.54, 36.26) | 0.966 |
| 716 | Other arthropathies | 39797 | 0.07 (-2.75, 2.89) | 0.966 | 0.15 (-3.66, 3.96) | 0.945 | 0 (-0.01, 0.01) | 0.97 | -0.13 (-1.75, 1.49) | 0.887 | 0.1 (-3.93, 4.13) | 0.966 |
| 716.2 | Unspecified monoarthritis | 15585 | 0.13 (-5.11, 5.37) | 0.965 | 0.27 (-5.7, 6.24) | 0.936 | -0.16 (-7.78, 7.46) | 0.97 | -0.21 (-2.94, 2.53) | 0.89 | 0.26 (-4.56, 5.08) | 0.923 |
| 716.8 | Palindromic rheumatism | 338 | 0.54 (-25.18, 26.27) | 0.97 | -1.51 (-42.59, 39.58) | 0.948 | 0.62 (-28.59, 29.82) | 0.97 | -0.04 (-1.75, 1.68) | 0.97 | -1.58 (-34.13, 30.97) | 0.931 |
| 716.9 | Arthropathy NOS | 38702 | 0.06 (-2.6, 2.73) | 0.966 | 0.15 (-3.3, 3.61) | 0.937 | 0.03 (-1.3, 1.35) | 0.97 | -0.13 (-1.76, 1.5) | 0.884 | 0.07 (-3.24, 3.38) | 0.97 |
| 717 | Polymyalgia Rheumatica | 3824 | 0.11 (-5.18, 5.4) | 0.97 | 0.08 (-3.84, 4.01) | 0.97 | -0.68 (-15.03, 13.66) | 0.932 | -0.04 (-1.84, 1.77) | 0.97 | -0.25 (-10.38, 9.89) | 0.966 |
| 720 | Spinal stenosis | 14969 | 0.05 (-2.26, 2.36) | 0.97 | -0.01 (-0.54, 0.52) | 0.97 | 0.14 (-6.01, 6.3) | 0.967 | -0.07 (-2.99, 2.85) | 0.966 | -0.14 (-3.02, 2.74) | 0.931 |
| 721 | Spondylosis and allied disorders | 14782 | 0.07 (-3.44, 3.59) | 0.97 | -0.07 (-3.43, 3.29) | 0.97 | -0.12 (-5.97, 5.72) | 0.97 | -0.02 (-1.19, 1.14) | 0.97 | -0.02 (-0.73, 0.7) | 0.97 |
| 721.1 | Spondylosis without myelopathy | 10019 | 0.21 (-4.15, 4.57) | 0.932 | -0.14 (-6.76, 6.48) | 0.97 | -0.14 (-6.86, 6.58) | 0.97 | -0.13 (-5.47, 5.22) | 0.966 | -0.14 (-6.61, 6.34) | 0.97 |
| 721.2 | Spondylosis with myelopathy | 1402 | -0.09 (-4.3, 4.12) | 0.97 | -0.19 (-8.97, 8.6) | 0.97 | 0.18 (-8.37, 8.73) | 0.97 | 0.13 (-6.11, 6.37) | 0.97 | 0.03 (-1.33, 1.39) | 0.97 |
| 721.8 | Other allied disorders of spine | 2430 | 0.17 (-7.97, 8.31) | 0.97 | 0.17 (-7.84, 8.18) | 0.97 | -0.56 (-26.81, 25.69) | 0.97 | -0.17 (-7.97, 7.64) | 0.97 | -0.23 (-11.01, 10.55) | 0.97 |
| 722 | Intervertebral disc disorders | 17488 | -0.05 (-2.28, 2.19) | 0.97 | 0.2 (-5.13, 5.52) | 0.948 | 0.04 (-1.92, 2) | 0.97 | -0.04 (-1.96, 1.88) | 0.97 | -0.16 (-5.84, 5.52) | 0.96 |
| 722.1 | Displacement of intervertebral disc | 555 | 0.33 (-15.48, 16.15) | 0.97 | 1.14 (-22.31, 24.6) | 0.93 | -0.2 (-9.82, 9.41) | 0.97 | -0.74 (-15.59, 14.11) | 0.929 | -1.02 (-21.89, 19.86) | 0.93 |
| 722.6 | Degeneration of intervertebral disc | 10783 | 0.02 (-1.01, 1.06) | 0.97 | 0.1 (-4.71, 4.92) | 0.97 | 0.33 (-8.77, 9.44) | 0.948 | -0.12 (-4.62, 4.38) | 0.961 | -0.03 (-1.42, 1.37) | 0.97 |
| 722.7 | Intervertebral disc disorder with myelopathy | 747 | -0.48 (-13.64, 12.68) | 0.948 | 1.41 (-9.96, 12.79) | 0.82 | 1.33 (-34.95, 37.62) | 0.948 | -0.37 (-15.63, 14.89) | 0.966 | -0.22 (-10.42, 9.99) | 0.97 |
| 722.8 | Postlaminectomy syndrome | 133 | -1.8 (-20.97, 17.36) | 0.864 | 0.65 (-30.01, 31.3) | 0.97 | 1.31 (-60.85, 63.48) | 0.97 | 1.3 (-28.17, 30.76) | 0.937 | 3.76 (-16.77, 24.29) | 0.733 |
| 722.9 | Other and unspecified disc disorder | 7756 | -0.12 (-5.6, 5.37) | 0.97 | 0.42 (-8.07, 8.92) | 0.929 | -0.44 (-18.61, 17.73) | 0.966 | 0.02 (-0.89, 0.93) | 0.97 | -0.21 (-8.91, 8.49) | 0.966 |
| 723 | Other disorders of cervical region | 1412 | -0.03 (-1.67, 1.6) | 0.97 | 1.01 (-12.19, 14.21) | 0.89 | -0.47 (-22.92, 21.97) | 0.97 | -0.36 (-15.29, 14.58) | 0.966 | -0.27 (-12.96, 12.43) | 0.97 |
| 723.1 | Torticollis | 510 | 0.24 (-11.19, 11.67) | 0.97 | 0.43 (-19.98, 20.85) | 0.97 | -0.57 (-27.29, 26.16) | 0.97 | -0.32 (-15.5, 14.86) | 0.97 | 0.64 (-29.46, 30.73) | 0.97 |
| 724 | Other and unspecified disorders of back | 1254 | -0.28 (-13.72, 13.15) | 0.97 | 1.55 (-10.97, 14.08) | 0.82 | -1.02 (-43.17, 41.12) | 0.966 | -0.23 (-11.27, 10.8) | 0.97 | -0.25 (-12.17, 11.67) | 0.97 |
| 724.9 | Other unspecified back disorders | 1177 | -0.38 (-16.19, 15.43) | 0.966 | 1.51 (-11.63, 14.65) | 0.833 | -0.46 (-22, 21.09) | 0.97 | -0.22 (-10.8, 10.36) | 0.97 | -0.42 (-20.13, 19.3) | 0.97 |
| 726 | Peripheral enthesopathies and allied syndromes | 48003 | -0.05 (-2.03, 1.94) | 0.966 | 0.09 (-3.49, 3.68) | 0.963 | 0.26 (-2.46, 2.97) | 0.864 | -0.04 (-1.7, 1.63) | 0.97 | 0.03 (-1.22, 1.27) | 0.97 |
| 726.1 | Enthesopathy | 24109 | -0.06 (-2.67, 2.56) | 0.97 | 0.19 (-3.64, 4.01) | 0.93 | 0.38 (-3.66, 4.42) | 0.864 | -0.09 (-3.42, 3.24) | 0.963 | 0.05 (-2.34, 2.44) | 0.97 |
| 726.2 | Synoviopathy | 731 | 0.45 (-17.94, 18.83) | 0.966 | -0.5 (-23.91, 22.92) | 0.97 | 0.87 (-40.21, 41.94) | 0.97 | -0.39 (-16.66, 15.87) | 0.966 | -0.41 (-19.81, 18.99) | 0.97 |
| 726.3 | Bursitis | 6323 | 0.08 (-3.83, 3.99) | 0.97 | 0.04 (-1.83, 1.9) | 0.97 | -0.23 (-11.31, 10.84) | 0.97 | -0.06 (-3.08, 2.95) | 0.97 | -0.02 (-0.81, 0.78) | 0.97 |
| 726.4 | Calcaneal spur; Exostosis NOS | 721 | -0.22 (-10.39, 9.96) | 0.97 | -0.74 (-31.42, 29.93) | 0.966 | 0.33 (-15.06, 15.71) | 0.97 | 0.48 (-19.25, 20.21) | 0.966 | -0.11 (-5.52, 5.29) | 0.97 |
| 727 | Other disorders of synovium, tendon, and bursa | 30487 | -0.01 (-0.31, 0.3) | 0.97 | 0.23 (-4.41, 4.87) | 0.929 | 0.01 (-0.62, 0.65) | 0.97 | -0.09 (-3.75, 3.57) | 0.966 | 0.13 (-5.1, 5.36) | 0.966 |
| 727.1 | Synovitis and tenosynovitis | 15197 | 0.06 (-2.83, 2.95) | 0.97 | 0.03 (-1.51, 1.57) | 0.97 | 0.03 (-1.57, 1.64) | 0.97 | -0.08 (-3.93, 3.77) | 0.97 | 0.01 (-0.51, 0.53) | 0.97 |
| 727.2 | Bursitis disorders | 382 | -0.57 (-24.01, 22.87) | 0.966 | 0.68 (-31.45, 32.81) | 0.97 | 2.57 (-30.95, 36.08) | 0.89 | -0.06 (-2.83, 2.72) | 0.97 | 1.03 (-26.88, 28.93) | 0.948 |
| 727.4 | Ganglion and cyst of synovium, tendon, and bursa | 7134 | 0.18 (-7.14, 7.49) | 0.966 | 0.01 (-0.32, 0.33) | 0.97 | -0.39 (-17.49, 16.71) | 0.967 | -0.11 (-5.32, 5.1) | 0.97 | 0.07 (-3.15, 3.29) | 0.97 |
| 727.5 | Rupture of synovium | 3226 | 0.05 (-2.35, 2.46) | 0.97 | 0.54 (-10.59, 11.67) | 0.93 | 0.91 (-14.33, 16.14) | 0.915 | -0.45 (-5.21, 4.32) | 0.864 | 0.3 (-11.99, 12.58) | 0.966 |
| 727.6 | Rupture of tendon, nontraumatic | 811 | 0.31 (-14.13, 14.74) | 0.97 | 0.09 (-3.98, 4.16) | 0.97 | -0.76 (-33.89, 32.38) | 0.967 | -0.18 (-8.72, 8.36) | 0.97 | 0 (-0.04, 0.05) | 0.97 |
| 727.7 | Contracture of tendon (sheath) | 391 | 0.61 (-24.34, 25.55) | 0.966 | -0.64 (-30.81, 29.54) | 0.97 | 0.61 (-28.09, 29.3) | 0.97 | -0.41 (-19.83, 19.01) | 0.97 | -1.79 (-17.39, 13.8) | 0.833 |
| 728 | Disorders of muscle, ligament, and fascia | 11338 | -0.1 (-5, 4.8) | 0.97 | 0.16 (-7.25, 7.56) | 0.97 | 0.26 (-11.81, 12.32) | 0.97 | 0 (-0.19, 0.2) | 0.97 | -0.03 (-1.43, 1.37) | 0.97 |
| 728.2 | Laxity of ligament or hypermobility syndrome | 1949 | -0.02 (-1.02, 0.97) | 0.97 | -0.04 (-2.06, 1.97) | 0.97 | 0.48 (-22.34, 23.3) | 0.97 | -0.04 (-1.9, 1.82) | 0.97 | 0.47 (-12.4, 13.35) | 0.948 |
| 728.7 | Fasciitis | 1913 | -0.24 (-10.31, 9.82) | 0.966 | 0.6 (-11.9, 13.09) | 0.932 | 0.51 (-23.51, 24.53) | 0.97 | -0.09 (-4.29, 4.12) | 0.97 | -0.12 (-5.89, 5.65) | 0.97 |
| 728.71 | Contracture of palmar fascia [Dupuytren's disease] | 5857 | -0.15 (-7.24, 6.94) | 0.97 | 0.18 (-8.36, 8.72) | 0.97 | 0.07 (-3.14, 3.28) | 0.97 | 0.07 (-3.37, 3.51) | 0.97 | -0.12 (-5.98, 5.73) | 0.97 |
| 729 | Other disorders of soft tissues | 32004 | -0.06 (-2.68, 2.55) | 0.966 | 0.14 (-3.55, 3.82) | 0.948 | -0.05 (-2.23, 2.13) | 0.97 | 0.01 (-0.59, 0.61) | 0.97 | 0.17 (-1.95, 2.28) | 0.887 |
| 729.1 | Rheumatism, unspecified and fibrositis | 2236 | -0.36 (-7.85, 7.12) | 0.93 | -0.18 (-8.53, 8.18) | 0.97 | 0.49 (-22.66, 23.64) | 0.97 | 0.38 (-7.48, 8.25) | 0.93 | -0.15 (-7.38, 7.07) | 0.97 |
| 729.3 | Panniculitis | 210 | 0.79 (-31.71, 33.29) | 0.966 | 2.3 (-26.94, 31.53) | 0.887 | -3.13 (-66.14, 59.87) | 0.929 | -1.24 (-29, 26.51) | 0.936 | -1.59 (-44.8, 41.63) | 0.948 |
| 731 | Osteitis deformans and osteopathies associated with other disorders classified elsewhere | 198 | -1.02 (-43.19, 41.14) | 0.966 | -0.72 (-34.56, 33.13) | 0.97 | 4.7 (-54.89, 64.28) | 0.886 | 0.52 (-23.98, 25.02) | 0.97 | 0.49 (-22.81, 23.79) | 0.97 |
| 732 | Osteochondropathies | 4134 | 0.29 (-11.79, 12.37) | 0.966 | -0.7 (-14.68, 13.29) | 0.929 | 0.35 (-16.14, 16.83) | 0.97 | -0.09 (-4.22, 4.04) | 0.97 | -0.81 (-9.41, 7.8) | 0.864 |
| 732.1 | Juvenile osteochondrosis | 2169 | 0.34 (-13.53, 14.2) | 0.966 | -0.48 (-21.65, 20.68) | 0.967 | -0.31 (-14.86, 14.24) | 0.97 | -0.09 (-4.11, 3.94) | 0.97 | -1.02 (-11.89, 9.85) | 0.864 |
| 732.7 | Osteochondritis dissecans | 1214 | 0.66 (-17.29, 18.61) | 0.948 | -1.34 (-28.17, 25.5) | 0.929 | 0.53 (-24.4, 25.46) | 0.97 | -0.24 (-11.72, 11.24) | 0.97 | -1.42 (-16.56, 13.71) | 0.864 |
| 733 | Other disorders of bone and cartilage | 6382 | -0.18 (-7.75, 7.38) | 0.966 | 0.52 (-6.97, 8.02) | 0.9 | 0.91 (-8.77, 10.59) | 0.864 | -0.18 (-7.71, 7.34) | 0.966 | 0.28 (-11.15, 11.71) | 0.966 |
| 733.2 | Cyst of bone | 372 | -0.6 (-29.01, 27.81) | 0.97 | 0.46 (-21.44, 22.36) | 0.97 | 0.27 (-12.67, 13.22) | 0.97 | 0.41 (-19.09, 19.91) | 0.97 | 2.12 (-24.87, 29.12) | 0.887 |
| 733.4 | Aseptic necrosis of bone | 735 | 0.15 (-6.89, 7.18) | 0.97 | 0.42 (-19.36, 20.19) | 0.97 | 1.93 (-18.63, 22.49) | 0.864 | -0.69 (-10.57, 9.2) | 0.9 | -0.41 (-19.85, 19.03) | 0.97 |
| 733.8 | Malunion and nonunion of fracture | 1460 | 0.17 (-7.65, 7.98) | 0.97 | -0.28 (-13.53, 12.97) | 0.97 | 0.12 (-5.34, 5.57) | 0.97 | -0.08 (-3.72, 3.57) | 0.97 | 0.18 (-8.18, 8.54) | 0.97 |
| 735 | Acquired foot deformities | 14287 | 0.14 (-5.83, 6.12) | 0.966 | 0.36 (-5.97, 6.68) | 0.919 | -0.12 (-5.81, 5.57) | 0.97 | -0.27 (-3.17, 2.63) | 0.864 | 0.31 (-5.2, 5.82) | 0.919 |
| 735.1 | Flat foot | 1061 | 0.35 (-16.08, 16.78) | 0.97 | 0.82 (-32.91, 34.55) | 0.966 | -0.75 (-36.45, 34.94) | 0.97 | -0.53 (-22.25, 21.2) | 0.966 | -0.22 (-10.53, 10.09) | 0.97 |
| 735.2 | Acquired toe deformities | 921 | -0.16 (-7.6, 7.28) | 0.97 | -1.71 (-16.23, 12.82) | 0.829 | 0.04 (-1.83, 1.91) | 0.97 | 0.89 (-8.54, 10.31) | 0.864 | 0.03 (-1.5, 1.56) | 0.97 |
| 735.21 | Hammer toe (acquired) | 2739 | -0.12 (-5.58, 5.35) | 0.97 | 0.81 (-12.55, 14.17) | 0.913 | 0.88 (-22.94, 24.69) | 0.948 | -0.34 (-11.18, 10.49) | 0.955 | 0.13 (-6.04, 6.31) | 0.97 |
| 735.23 | Hallux rigidus | 2497 | 0.53 (-5.15, 6.21) | 0.865 | -0.02 (-1, 0.96) | 0.97 | -0.51 (-24.73, 23.7) | 0.97 | -0.45 (-9.39, 8.5) | 0.929 | 0.03 (-1.38, 1.44) | 0.97 |
| 735.3 | Hallux valgus (Bunion) | 8115 | 0.11 (-4.94, 5.16) | 0.97 | 0.35 (-9.26, 9.97) | 0.948 | -0.36 (-17.15, 16.44) | 0.97 | -0.19 (-8.19, 7.8) | 0.966 | 0.39 (-7.56, 8.34) | 0.93 |
| 736 | Other acquired deformities of limbs | 1986 | 0.1 (-4.48, 4.67) | 0.97 | -0.25 (-12.12, 11.62) | 0.97 | -1.52 (-20.97, 17.92) | 0.887 | 0.25 (-11.43, 11.92) | 0.97 | -0.35 (-16.96, 16.26) | 0.97 |
| 736.2 | Acquired deformities of finger | 221 | -0.35 (-16.88, 16.18) | 0.97 | 0.44 (-20.43, 21.32) | 0.97 | -2.11 (-94.09, 89.88) | 0.967 | 0.43 (-19.82, 20.67) | 0.97 | -0.67 (-32.32, 30.98) | 0.97 |
| 737 | Curvature of spine | 4742 | -0.15 (-7.38, 7.08) | 0.97 | 0.47 (-12.23, 13.16) | 0.948 | -0.25 (-12.05, 11.55) | 0.97 | 0 (-0.01, 0.01) | 0.97 | 0.16 (-7.61, 7.94) | 0.97 |
| 737.1 | Kyphosis (acquired) | 940 | -0.18 (-8.5, 8.15) | 0.97 | 0.64 (-29.47, 30.74) | 0.97 | 0.93 (-43.02, 44.88) | 0.97 | -0.28 (-13.63, 13.07) | 0.97 | -0.98 (-41.48, 39.51) | 0.966 |
| 737.3 | Kyphoscoliosis and scoliosis | 3889 | -0.29 (-8.64, 8.05) | 0.95 | 0.55 (-11.07, 12.18) | 0.932 | -0.14 (-6.77, 6.49) | 0.97 | 0.09 (-3.99, 4.16) | 0.97 | 0.4 (-10.48, 11.28) | 0.948 |
| 738 | Other acquired musculoskeletal deformity | 7313 | -0.02 (-0.94, 0.9) | 0.97 | 0.15 (-7.03, 7.33) | 0.97 | 0.91 (-3.57, 5.38) | 0.705 | -0.22 (-4.68, 4.24) | 0.929 | 0 (-0.07, 0.07) | 0.97 |
| 738.4 | Acquired spondylolisthesis | 4123 | 0.1 (-4.41, 4.6) | 0.97 | 0.06 (-2.58, 2.69) | 0.97 | 0.52 (-13.62, 14.66) | 0.948 | -0.23 (-6.38, 5.93) | 0.948 | -0.02 (-0.77, 0.73) | 0.97 |
| 739 | Contracture of joint | 679 | 1.01 (-19.33, 21.35) | 0.929 | -0.7 (-34.02, 32.61) | 0.97 | 0.36 (-16.48, 17.19) | 0.97 | -0.8 (-22.57, 20.97) | 0.948 | 0.61 (-28.24, 29.46) | 0.97 |
| 740 | Osteoarthrosis | 53711 | -0.04 (-1.87, 1.79) | 0.967 | 0.14 (-1.66, 1.94) | 0.887 | 0.06 (-2.74, 2.86) | 0.97 | -0.02 (-1.1, 1.05) | 0.97 | -0.08 (-2.24, 2.08) | 0.948 |
| 740.1 | Osteoarthritis; localized | 10926 | 0.09 (-4.19, 4.37) | 0.97 | -0.11 (-5.27, 5.05) | 0.97 | 0.14 (-6.51, 6.79) | 0.97 | -0.07 (-3.58, 3.43) | 0.97 | -0.11 (-5.23, 5.01) | 0.97 |
| 740.11 | Osteoarthrosis, localized, primary | 40386 | -0.05 (-1.51, 1.4) | 0.948 | 0.16 (-1.54, 1.85) | 0.864 | -0.1 (-4.35, 4.14) | 0.966 | 0.01 (-0.51, 0.53) | 0.97 | -0.06 (-2.43, 2.31) | 0.966 |
| 740.12 | Osteoarthrosis, localized, secondary | 2064 | -0.06 (-2.77, 2.65) | 0.97 | 0.29 (-13.37, 13.95) | 0.97 | -0.19 (-8.98, 8.61) | 0.97 | -0.02 (-1.01, 0.97) | 0.97 | -0.63 (-8.7, 7.43) | 0.887 |
| 740.2 | Osteoarthrosis, generalized | 574 | 1 (-8.78, 10.78) | 0.852 | -0.2 (-9.53, 9.14) | 0.97 | -0.96 (-40.42, 38.51) | 0.966 | -0.72 (-14.22, 12.77) | 0.923 | -0.67 (-19.25, 17.91) | 0.949 |
| 740.9 | Osteoarthrosis NOS | 10961 | -0.07 (-3.14, 3.01) | 0.97 | -0.02 (-0.73, 0.7) | 0.97 | 0.51 (-5.94, 6.96) | 0.887 | -0.03 (-1.22, 1.17) | 0.97 | -0.06 (-2.72, 2.61) | 0.97 |
| 741 | Symptoms and disorders of the joints | 5108 | 0.23 (-6.15, 6.62) | 0.948 | -0.39 (-10.94, 10.17) | 0.948 | 0.14 (-6.36, 6.64) | 0.97 | -0.11 (-5.26, 5.04) | 0.97 | 0.08 (-3.58, 3.74) | 0.97 |
| 741.2 | Stiffness of joint | 100 | 1.8 (-34.76, 38.37) | 0.929 | 0.45 (-20.68, 21.58) | 0.97 | 1.61 (-74.55, 77.77) | 0.97 | -2.46 (-28.66, 23.74) | 0.864 | -3.09 (-36.01, 29.82) | 0.864 |
| 741.3 | Difficulty in walking | 603 | -0.23 (-10.47, 10) | 0.967 | 0.51 (-20.58, 21.6) | 0.966 | 0.49 (-22.79, 23.77) | 0.97 | 0.01 (-0.34, 0.36) | 0.97 | -0.21 (-10.19, 9.76) | 0.97 |
| 741.4 | Joint effusions | 823 | 0.01 (-0.55, 0.58) | 0.97 | -1.45 (-31.22, 28.32) | 0.93 | 2.37 (-27.76, 32.5) | 0.887 | 0.05 (-2.48, 2.59) | 0.97 | 0.24 (-10.89, 11.37) | 0.97 |
| 741.5 | Hemarthrosis | 290 | 0.02 (-0.98, 1.03) | 0.97 | 0.18 (-8.3, 8.65) | 0.97 | 2.09 (-89.95, 94.13) | 0.968 | -0.41 (-19.9, 19.07) | 0.97 | 0.26 (-12.22, 12.75) | 0.97 |
| 742 | Derangement of joint, non-traumatic | 16653 | -0.22 (-3.32, 2.89) | 0.9 | 0.31 (-5.98, 6.6) | 0.93 | -0.16 (-7.6, 7.29) | 0.97 | 0.12 (-5, 5.25) | 0.966 | 0.35 (-3.38, 4.09) | 0.864 |
| 742.1 | Loose body in joint | 1608 | 0.26 (-11.87, 12.38) | 0.97 | -0.8 (-33.94, 32.33) | 0.966 | 1.46 (-38.24, 41.15) | 0.948 | -0.18 (-8.65, 8.29) | 0.97 | -0.3 (-14.34, 13.75) | 0.97 |
| 742.2 | Pathological, developmental or recurrent dislocation | 1766 | -0.21 (-9.36, 8.95) | 0.968 | 1.27 (-3.77, 6.31) | 0.634 | -0.35 (-16.82, 16.13) | 0.97 | -0.23 (-9.83, 9.36) | 0.966 | -0.23 (-11.04, 10.58) | 0.97 |
| 742.8 | Articular cartilage disorder | 1354 | -0.09 (-4.33, 4.15) | 0.97 | 1.16 (-4.58, 6.91) | 0.705 | 0.07 (-3.33, 3.48) | 0.97 | -0.4 (-8.6, 7.8) | 0.93 | -0.15 (-7.32, 7.02) | 0.97 |
| 742.9 | Other derangement of joint | 13670 | -0.23 (-3.2, 2.74) | 0.887 | 0.35 (-4.15, 4.85) | 0.887 | -0.27 (-11.6, 11.05) | 0.966 | 0.14 (-4.95, 5.23) | 0.96 | 0.43 (-3.01, 3.87) | 0.82 |
| 743 | Osteoporosis, osteopenia and pathological fracture | 15875 | -0.03 (-1.38, 1.33) | 0.97 | -0.09 (-4.18, 4.01) | 0.97 | 0.3 (-5.76, 6.35) | 0.93 | 0.01 (-0.68, 0.71) | 0.97 | -0.31 (-1.81, 1.2) | 0.705 |
| 743.11 | Osteoporosis NOS | 13633 | -0.04 (-1.96, 1.88) | 0.97 | -0.01 (-0.31, 0.3) | 0.97 | 0.1 (-4.55, 4.75) | 0.97 | 0.03 (-1.28, 1.34) | 0.97 | -0.26 (-2.55, 2.02) | 0.833 |
| 743.2 | Pathologic fracture | 1002 | -0.23 (-10.26, 9.8) | 0.967 | -0.12 (-5.96, 5.71) | 0.97 | 0.68 (-27.51, 28.88) | 0.966 | 0.17 (-7.64, 7.97) | 0.97 | 0.01 (-0.69, 0.72) | 0.97 |
| 743.21 | Pathologic fracture of vertebrae | 618 | 0.22 (-10.02, 10.45) | 0.97 | -0.31 (-14.98, 14.36) | 0.97 | 0.38 (-17.64, 18.4) | 0.97 | -0.15 (-7.26, 6.96) | 0.97 | -0.46 (-13.09, 12.16) | 0.948 |
| 743.9 | Osteopenia or other disorder of bone and cartilage | 1510 | 0.18 (-8.35, 8.71) | 0.97 | -0.96 (-11.13, 9.21) | 0.864 | 2.07 (-15.51, 19.64) | 0.829 | -0.1 (-4.64, 4.45) | 0.97 | -0.35 (-16.49, 15.8) | 0.97 |
| 745 | Pain in joint | 8444 | -0.03 (-1.58, 1.51) | 0.97 | 0.23 (-9.49, 9.95) | 0.966 | 0.47 (-12.24, 13.18) | 0.948 | -0.15 (-6.46, 6.15) | 0.966 | -0.56 (-5.44, 4.32) | 0.833 |
| 860 | Bone marrow or stem cell transplant | 142 | -0.39 (-19.01, 18.22) | 0.97 | -1.06 (-44.55, 42.44) | 0.966 | 3.5 (-122.85, 129.86) | 0.961 | 0.58 (-21.53, 22.69) | 0.963 | -0.24 (-11.62, 11.14) | 0.97 |
| 931 | Contact dermatitis and other eczema due to plants [except food] | 159 | 0.15 (-6.84, 7.14) | 0.97 | -2.13 (-57.71, 53.44) | 0.945 | 4.66 (-90.97, 100.29) | 0.93 | 0.24 (-11.02, 11.5) | 0.97 | -2.39 (-39.79, 35) | 0.908 |
| 938 | Dermatitis due to solar radiation | 944 | -0.36 (-15.34, 14.61) | 0.966 | -2.03 (-10.81, 6.75) | 0.664 | 0.82 (-33.74, 35.39) | 0.966 | 0.94 (-7.24, 9.12) | 0.833 | 0.12 (-5.58, 5.82) | 0.97 |
| 938.1 | Acute dermatitis due to solar radiation | 275 | 0.02 (-0.71, 0.74) | 0.97 | -4.27 (-21.17, 12.64) | 0.634 | 0.62 (-28.78, 30.02) | 0.97 | 1.37 (-24.22, 26.96) | 0.923 | 0.68 (-31.7, 33.07) | 0.97 |
| 939 | Atopic/contact dermatitis due to other or unspecified | 15304 | -0.24 (-3.36, 2.87) | 0.887 | 0.28 (-6.32, 6.89) | 0.939 | 0.49 (-9.66, 10.65) | 0.93 | 0.04 (-1.7, 1.77) | 0.97 | 0.17 (-7.01, 7.36) | 0.966 |
| 947 | Urticaria | 11043 | -0.3 (-4.1, 3.5) | 0.887 | 0.14 (-6.5, 6.78) | 0.97 | 1.26 (-2.81, 5.32) | 0.556 | 0 (-0.22, 0.23) | 0.97 | 0.29 (-7.64, 8.22) | 0.948 |

a. Statistically significant effect estimates are marked with bold (FDR adjusted p-value<0.05).
b. The FDR adjusted p-values and 95% confidence intervals are presented.
c. FDR adjusted p-values above 0.97 were set to 0.97 to enable estimation of adjusted confidence intervals.
d. Estimates represent increases or decreases in years of age of first diagnosis.
