# Supplementary material for: Associations of ABO and Rhesus D blood groups with phenome-wide disease incidence: A 41-year retrospective cohort study of 482,914 patients
Source: eLife. 2023 Mar 9;12:e83116. doi: 10.7554/eLife.83116 (PMC10042530; doi:10.7554/eLife.83116)
Supplement: Supplementary file 6. — a. Statistically significant IRRs are marked with bold (FDR adjusted P-value <0.05). b. The IRRs are adjusted for age, sex, interaction between age and sex, and birth year. c. Blood group O and the RhD negative blood group was used as a reference, respectively. d. The FDR adjusted p-values and 95% confidence intervals are presented. e. FDR adjusted p-values above 0.97 were set to 0.97 to avoid exploding adjusted confidence intervals. f. Phecodes are divided by PheWAS disease categories. g. The number of events and the follow-up time in person-years for each Phecode is also presented. h. For study results of congenital Phecodes estimates marked with ** are prevalence ratios instead of IRRs and the corresponding person-year marked with * are the size of the cohort. [file elife-83116-supp6.docx]

## Supplementary file 6: Statistically significant associations for blood groups A, B and AB relative to blood group O. Further, also for RhD positive blood group relative to the RhD negative blood group.

|  | | | | **A** | | | **B** | | | **AB** | | | **Rhesus** | | |
| --- | --- | --- | --- | --- | --- | --- | --- | --- | --- | --- | --- | --- | --- | --- | --- |
| **Phecode** | **Phenotype** | **Total events** | **Person-years** | **Events** | **IRR (95%CI)** | **P-value** | **Events** | **IRR (95%CI)** | **P-value** | **Events** | **IRR (95%CI)** | **P-value** | **Events** | **IRR (95%CI)** | **P-value** |
| **Infectious Diseases** | | | | | | | | | | | | | | | |
| 010 | Tuberculosis | 2101 | 17603440 | 831 | 0.94 (0.64, 1.38) | 0.767 | 326 | **1.36 (1.13, 1.64)** | **0.001** | 102 | 1.09 (0.36, 3.33) | 0.885 | 1852 | **1.36 (1.12, 1.65)** | **0.002** |
| 070 | Viral hepatitis | 6596 | 17557078 | 2613 | **0.9 (0.83, 0.98)** | **0.02** | 920 | **1.16 (1.03, 1.31)** | **0.013** | 299 | 0.97 (0.4, 2.36) | 0.954 | 5679 | 1.12 (0.99, 1.27) | 0.064 |
| 070.2 | Viral hepatitis B | 1664 | 17613572 | 567 | **0.77 (0.66, 0.91)** | **0.002** | 309 | **1.49 (1.25, 1.77)** | **<0.001** | 85 | 1.07 (0.26, 4.36) | 0.929 | 1471 | **1.36 (1.07, 1.71)** | **0.011** |
| 071 | Human immunodeficiency virus [HIV] disease | 1182 | 17620808 | 446 | 0.84 (0.59, 1.19) | 0.327 | 164 | 1.1 (0.35, 3.41) | 0.883 | 64 | 1.12 (0.2, 6.16) | 0.906 | 1055 | **1.49 (1.04, 2.14)** | **0.029** |
| 071.1 | HIV infection, symptomatic | 1182 | 17620808 | 446 | 0.84 (0.59, 1.19) | 0.327 | 164 | 1.1 (0.35, 3.41) | 0.883 | 64 | 1.12 (0.2, 6.16) | 0.906 | 1055 | **1.49 (1.04, 2.14)** | **0.029** |
| **Neoplasms** | | | | | | | | | | | | | | | |
| 145.2 | Cancer of tongue | 606 | 17629055 | 257 | 0.99 (0.68, 1.45) | 0.97 | 74 | 1.14 (0.59, 2.22) | 0.708 | 32 | 1.23 (0.5, 3.04) | 0.67 | 483 | **0.74 (0.6, 0.92)** | **0.007** |
| 157 | Pancreatic cancer | 2828 | 17627948 | 1396 | **1.34 (1.2, 1.49)** | **<0.001** | 312 | 1.21 (0.92, 1.59) | 0.173 | 137 | 1.31 (0.89, 1.92) | 0.173 | 2381 | 1.01 (0.52, 1.98) | 0.97 |
| 180 | Cervical cancer and dysplasia | 12538 | 10308860 | 5462 | 1.03 (0.92, 1.15) | 0.623 | 1361 | **0.91 (0.83, 1)** | **0.046** | 554 | 0.96 (0.64, 1.45) | 0.861 | 10504 | 0.93 (0.86, 1.01) | 0.078 |
| 180.3 | Cervical intraepithelial neoplasia [CIN] [Cervical dysplasia] | 10895 | 10327107 | 4745 | 1.03 (0.9, 1.17) | 0.685 | 1182 | **0.9 (0.81, 1)** | **0.04** | 476 | 0.95 (0.67, 1.34) | 0.767 | 9128 | 0.93 (0.85, 1.01) | 0.093 |
| 195.1 | Malignant neoplasm, other | 7383 | 17603424 | 3124 | 0.93 (0.87, 1.01) | 0.082 | 783 | 0.92 (0.79, 1.06) | 0.234 | 317 | 0.93 (0.66, 1.3) | 0.668 | 6084 | **0.88 (0.82, 0.94)** | **<0.001** |
| 214.1 | Lipoma of skin and subcutaneous tissue | 5742 | 17582124 | 2472 | 1.04 (0.88, 1.23) | 0.697 | 717 | **1.14 (1.01, 1.28)** | **0.027** | 283 | 1.14 (0.9, 1.43) | 0.273 | 4866 | 1.03 (0.74, 1.42) | 0.883 |
| 216 | Benign neoplasm of skin | 12993 | 17495431 | 5590 | 0.99 (0.76, 1.3) | 0.954 | 1399 | **0.89 (0.81, 0.98)** | **0.016** | 589 | 0.97 (0.59, 1.61) | 0.923 | 10906 | 0.95 (0.85, 1.05) | 0.327 |
| 860 | Bone marrow or stem cell transplant | 142 | 17631302 | 61 | 0.84 (0.41, 1.73) | 0.656 | 11 | 0.55 (0.26, 1.18) | 0.128 | 1 | 0.13 (0.02, 1.13) | 0.064 | 136 | **4.15 (2.12, 8.11)** | **<0.001** |
| **Endocrine/Metabolic** | | | | | | | | | | | | | | | |
| 242 | Thyrotoxicosis with or without goiter | 9744 | 17527426 | 4025 | **0.91 (0.85, 0.98)** | **0.01** | 1100 | 0.94 (0.76, 1.16) | 0.576 | 397 | 0.86 (0.69, 1.06) | 0.156 | 8244 | 1.02 (0.72, 1.43) | 0.92 |
| 250 | Diabetes mellitus | 36810 | 17295033 | 16107 | **1.04 (1, 1.08)** | **0.033** | 4346 | **1.11 (1.07, 1.16)** | **<0.001** | 1716 | **1.09 (1.01, 1.18)** | **0.03** | 31265 | **1.07 (1.03, 1.11)** | **0.001** |
| 250.2 | Type 2 diabetes | 32505 | 17346533 | 14194 | 1.04 (0.99, 1.08) | 0.088 | 3869 | **1.13 (1.08, 1.18)** | **<0.001** | 1531 | **1.11 (1.02, 1.2)** | **0.018** | 27599 | **1.07 (1.02, 1.12)** | **0.004** |
| 261 | Vitamin deficiency | 6674 | 17594082 | 2787 | 0.96 (0.85, 1.09) | 0.531 | 848 | **1.12 (1.01, 1.25)** | **0.036** | 278 | 0.92 (0.65, 1.31) | 0.671 | 5676 | 1.06 (0.92, 1.23) | 0.428 |
| 261.4 | Vitamin D deficiency | 4105 | 17613013 | 1677 | 0.94 (0.85, 1.04) | 0.234 | 557 | **1.2 (1.09, 1.31)** | **<0.001** | 175 | 0.95 (0.55, 1.63) | 0.853 | 3527 | **1.14 (1.04, 1.25)** | **0.006** |
| 272 | Disorders of lipoid metabolism | 41222 | 17347032 | 18531 | **1.08 (1.03, 1.12)** | **<0.001** | 4502 | 1.05 (0.93, 1.18) | 0.443 | 1858 | 1.07 (0.9, 1.27) | 0.459 | 34767 | 1.02 (0.9, 1.17) | 0.733 |
| 272.11 | Hypercholesterolemia | 35565 | 17395012 | 15991 | **1.08 (1.05, 1.1)** | **<0.001** | 3885 | 1.05 (0.99, 1.11) | 0.107 | 1609 | 1.07 (0.99, 1.17) | 0.088 | 30005 | 1.03 (0.96, 1.1) | 0.455 |
| 272.13 | Mixed hyperlipidemia | 1324 | 17619911 | 593 | 1.12 (0.81, 1.55) | 0.512 | 151 | 1.13 (0.59, 2.16) | 0.725 | 80 | **1.49 (1, 2.21)** | **0.048** | 1128 | 1.09 (0.56, 2.1) | 0.818 |
| 276.14 | Hypopotassemia | 6139 | 17612482 | 2674 | 1.02 (0.82, 1.27) | 0.883 | 739 | **1.11 (1, 1.23)** | **0.04** | 235 | 0.88 (0.72, 1.08) | 0.212 | 5167 | 1 (0.86, 1.18) | 0.97 |
| **Hematopoietic** | | | | | | | | | | | | | | | |
| 282 | Hereditary hemolytic anemias | 947 | 482914* | 338 | 0.83 (0.51, 1.37)** | 0.48 | 154 | 1.28 (0.7, 2.35)** | 0.432 | 62 | 1.37 (0.53, 3.57)** | 0.531 | 856 | **1.65 (1.06, 2.56)**** | **0.026** |
| 282.8 | Other hemoglobinopathies | 557 | 482914* | 177 | **0.72 (0.54, 0.96)**** | **0.027** | 103 | 1.39 (0.94, 2.04)** | 0.096 | 38 | 1.37 (0.59, 3.2)** | 0.478 | 517 | **2.24 (1.52, 3.3)**** | **<0.001** |
| 286 | Coagulation defects | 4124 | 17606796 | 1882 | **1.18 (1.04, 1.33)** | **0.011** | 477 | 1.04 (0.5, 2.17) | 0.923 | 216 | 1.24 (0.85, 1.8) | 0.263 | 3461 | 0.93 (0.65, 1.34) | 0.723 |
| 286.11 | Von willebrand's disease | 214 | 482914* | 73 | **0.58 (0.35, 0.95)**** | **0.029** | 14 | 0.37 (0.14, 1.01)** | 0.051 | 4 | 0.28 (0.03, 3.09)** | 0.305 | 170 | 0.68 (0.28, 1.65)** | 0.405 |
| 286.3 | Coagulation defects complicating pregnancy or postpartum | 2015 | 10502418 | 902 | **1.2 (1.13, 1.28)** | **<0.001** | 267 | 1.14 (1, 1.29) | 0.054 | 108 | **1.27 (1.08, 1.49)** | **0.004** | 1687 | **0.88 (0.8, 0.97)** | **0.013** |
| 286.7 | Other and unspecified coagulation defects | 1085 | 17621421 | 520 | **1.36 (1.16, 1.59)** | **<0.001** | 125 | 1.19 (0.74, 1.93) | 0.476 | 73 | **1.79 (1.34, 2.4)** | **<0.001** | 922 | 1.03 (0.34, 3.11) | 0.958 |
| **Mental Disorders** | | | | | | | | | | | | | | | |
| 300.1 | Anxiety disorder | 7985 | 17603188 | 3487 | 1.02 (0.89, 1.17) | 0.771 | 889 | 0.98 (0.74, 1.29) | 0.873 | 348 | 0.97 (0.62, 1.51) | 0.901 | 6658 | **0.92 (0.86, 0.99)** | **0.027** |
| **Neurological** | | | | | | | | | | | | | | | |
| 333.8 | Other degenerative diseases of the basal ganglia | 381 | 17630209 | 144 | **0.74 (0.58, 0.95)** | **0.016** | 43 | 0.9 (0.26, 3.14) | 0.875 | 11 | 0.56 (0.23, 1.39) | 0.214 | 320 | 1 (0.95, 1.05) | 0.97 |
| 339 | Other headache syndromes | 3466 | 17603269 | 1514 | 1.09 (0.98, 1.21) | 0.096 | 453 | **1.16 (1.02, 1.33)** | **0.027** | 164 | 1.09 (0.7, 1.7) | 0.707 | 2961 | 1.06 (0.82, 1.36) | 0.695 |
| 345 | Epilepsy, recurrent seizures, convulsions | 23469 | 17228780 | 9960 | 0.98 (0.89, 1.08) | 0.714 | 2717 | 0.95 (0.86, 1.05) | 0.307 | 1051 | 0.95 (0.76, 1.2) | 0.7 | 19711 | **0.94 (0.89, 1)** | **0.038** |
| 345.3 | Convulsions | 14391 | 17351676 | 6076 | 0.98 (0.8, 1.21) | 0.883 | 1700 | 0.96 (0.79, 1.15) | 0.644 | 668 | 0.98 (0.5, 1.96) | 0.967 | 12065 | **0.92 (0.85, 0.99)** | **0.034** |
| **Sense Organs** | | | | | | | | | | | | | | | |
| 361.1 | Retinal detachment with retinal defect | 3037 | 17594394 | 1366 | 1.11 (0.95, 1.3) | 0.171 | 351 | 1.14 (0.86, 1.52) | 0.362 | 164 | **1.32 (1, 1.73)** | **0.046** | 2542 | 0.97 (0.54, 1.76) | 0.938 |
| 381 | Otitis media and Eustachian tube disorders | 22790 | 17144551 | 9978 | **1.07 (1.03, 1.12)** | **0.001** | 2698 | 0.98 (0.77, 1.25) | 0.877 | 1084 | 1.04 (0.8, 1.35) | 0.767 | 19277 | 0.96 (0.87, 1.06) | 0.455 |
| 381.1 | Otitis media | 12313 | 17364091 | 5443 | **1.08 (1.03, 1.15)** | **0.005** | 1447 | 0.99 (0.66, 1.48) | 0.952 | 567 | 1.02 (0.55, 1.87) | 0.959 | 10410 | 0.97 (0.82, 1.14) | 0.7 |
| 384.4 | Perforation of tympanic membrane | 2943 | 17598765 | 1276 | 1.1 (0.94, 1.29) | 0.248 | 404 | **1.23 (1.04, 1.45)** | **0.015** | 146 | 1.15 (0.72, 1.86) | 0.566 | 2518 | 1.06 (0.73, 1.55) | 0.775 |
| 385.5 | Tympanosclerosis and middle ear disease related to otitis media | 530 | 17625215 | 256 | **1.33 (1.02, 1.74)** | **0.038** | 61 | 1.17 (0.44, 3.1) | 0.77 | 29 | 1.42 (0.59, 3.42) | 0.445 | 453 | 1.08 (0.34, 3.37) | 0.906 |
| 386.9 | Dizziness and giddiness (Light-headedness and vertigo) | 1060 | 17624097 | 414 | **0.81 (0.71, 0.93)** | **0.003** | 124 | 0.93 (0.46, 1.9) | 0.861 | 39 | 0.74 (0.45, 1.21) | 0.23 | 904 | 1.08 (0.7, 1.68) | 0.739 |
| 389 | Hearing loss | 43238 | 17166114 | 18753 | 0.99 (0.88, 1.12) | 0.903 | 4773 | 1 (0.88, 1.14) | 0.97 | 1917 | 1 (0.91, 1.11) | 0.97 | 36625 | **1.06 (1.01, 1.11)** | **0.009** |
| 389.3 | Degenerative and vascular disorders of ear | 22354 | 17419947 | 9749 | 0.99 (0.79, 1.24) | 0.955 | 2404 | 1 (0.96, 1.04) | 0.97 | 990 | 1.01 (0.61, 1.69) | 0.959 | 18942 | **1.08 (1.02, 1.15)** | **0.012** |
| **Circulatory System** | | | | | | | | | | | | | | | |
| 402 | Elevated blood pressure reading without diagnosis of hypertension | 462 | 17630163 | 176 | **0.79 (0.63, 0.99)** | **0.038** | 54 | 0.92 (0.31, 2.69) | 0.883 | 20 | 0.86 (0.18, 4.03) | 0.861 | 394 | 1.07 (0.41, 2.77) | 0.898 |
| 411.2 | Myocardial infarction | 25905 | 17411193 | 11511 | 1.06 (0.99, 1.12) | 0.08 | 2954 | **1.1 (1.01, 1.19)** | **0.038** | 1166 | 1.06 (0.83, 1.36) | 0.632 | 21825 | 1.02 (0.82, 1.27) | 0.874 |
| 415 | Pulmonary heart disease | 10870 | 17565369 | 5214 | **1.29 (1.23, 1.35)** | **<0.001** | 1265 | **1.23 (1.13, 1.34)** | **<0.001** | 547 | **1.33 (1.18, 1.49)** | **<0.001** | 9068 | 0.95 (0.82, 1.1) | 0.492 |
| 415.11 | Pulmonary embolism and infarction, acute | 1533 | 17612465 | 792 | **1.56 (1.35, 1.79)** | **<0.001** | 183 | **1.43 (1.09, 1.89)** | **0.011** | 77 | 1.49 (0.94, 2.35) | 0.088 | 1262 | 0.88 (0.58, 1.34) | 0.575 |
| 428.1 | Congestive heart failure (CHF) NOS | 8357 | 17595328 | 3721 | 1.06 (0.99, 1.12) | 0.087 | 917 | 1.06 (0.91, 1.22) | 0.48 | 408 | **1.15 (1.02, 1.3)** | **0.019** | 7027 | 1.01 (0.74, 1.37) | 0.952 |
| 440 | Atherosclerosis | 10901 | 17554704 | 4890 | **1.1 (1.03, 1.16)** | **0.002** | 1274 | **1.16 (1.06, 1.26)** | **<0.001** | 530 | **1.18 (1.03, 1.36)** | **0.018** | 9205 | 1.04 (0.87, 1.24) | 0.683 |
| 440.2 | Atherosclerosis of the extremities | 8348 | 17570336 | 3772 | **1.1 (1.03, 1.18)** | **0.004** | 966 | **1.15 (1.03, 1.28)** | **0.012** | 394 | 1.15 (0.94, 1.42) | 0.184 | 7041 | 1.03 (0.79, 1.35) | 0.832 |
| 442.2 | Aneurysm of iliac artery | 326 | 17630660 | 121 | **0.76 (0.64, 0.9)** | **0.001** | 39 | 1 (0.95, 1.06) | 0.97 | 17 | 1.07 (0.22, 5.19) | 0.94 | 258 | **0.72 (0.6, 0.87)** | **<0.001** |
| 443 | Peripheral vascular disease | 13791 | 17546796 | 6173 | **1.06 (1.01, 1.12)** | **0.029** | 1515 | 1.05 (0.89, 1.23) | 0.6 | 626 | 1.07 (0.85, 1.35) | 0.588 | 11650 | 1.03 (0.9, 1.19) | 0.644 |
| 443.7 | Peripheral angiopathy in diseases classified elsewhere | 3677 | 17611414 | 1621 | 1.05 (0.9, 1.24) | 0.53 | 435 | 1.13 (0.96, 1.33) | 0.128 | 177 | 1.14 (0.83, 1.58) | 0.424 | 3166 | **1.18 (1.05, 1.31)** | **0.004** |
| 443.9 | Peripheral vascular disease, unspecified | 9899 | 17576545 | 4478 | **1.08 (1.01, 1.14)** | **0.02** | 1050 | 1.02 (0.73, 1.43) | 0.91 | 449 | 1.08 (0.82, 1.42) | 0.62 | 8321 | 1.01 (0.74, 1.36) | 0.97 |
| 444 | Arterial embolism and thrombosis | 2390 | 17614619 | 1120 | **1.25 (1.11, 1.4)** | **<0.001** | 288 | **1.27 (1.03, 1.57)** | **0.025** | 133 | **1.46 (1.12, 1.9)** | **0.005** | 2009 | 1 (0.92, 1.08) | 0.97 |
| 444.1 | Arterial embolism and thrombosis of lower extremity artery | 1286 | 17623872 | 599 | **1.25 (1.01, 1.54)** | **0.042** | 157 | 1.3 (0.9, 1.89) | 0.163 | 77 | **1.59 (1.05, 2.38)** | **0.027** | 1077 | 0.98 (0.35, 2.76) | 0.97 |
| 451 | Phlebitis and thrombophlebitis | 16748 | 17479709 | 8053 | **1.36 (1.31, 1.41)** | **<0.001** | 2092 | **1.38 (1.3, 1.45)** | **<0.001** | 941 | **1.55 (1.44, 1.67)** | **<0.001** | 14036 | 0.97 (0.84, 1.13) | 0.723 |
| 451.2 | Phlebitis and thrombophlebitis of lower extremities | 15650 | 17489528 | 7573 | **1.38 (1.33, 1.43)** | **<0.001** | 1944 | **1.38 (1.31, 1.46)** | **<0.001** | 890 | **1.59 (1.48, 1.71)** | **<0.001** | 13108 | 0.97 (0.84, 1.12) | 0.685 |
| 452 | Other venous embolism and thrombosis | 4275 | 17607194 | 2076 | **1.42 (1.32, 1.52)** | **<0.001** | 564 | **1.49 (1.34, 1.66)** | **<0.001** | 246 | **1.64 (1.41, 1.9)** | **<0.001** | 3601 | 1 (0.89, 1.11) | 0.97 |
| 452.8 | Postphlebitic syndrome | 341 | 17629425 | 172 | **1.74 (1.38, 2.2)** | **<0.001** | 48 | **1.81 (1.26, 2.61)** | **0.001** | 27 | **2.61 (1.76, 3.86)** | **<0.001** | 295 | 1.18 (0.53, 2.63) | 0.699 |
| 454 | Varicose veins | 16500 | 17381971 | 7512 | **1.11 (1.03, 1.2)** | **0.005** | 1806 | 1.04 (0.69, 1.56) | 0.864 | 760 | 1.09 (0.74, 1.61) | 0.664 | 13840 | 0.98 (0.69, 1.38) | 0.9 |
| 454.1 | Varicose veins of lower extremity | 12511 | 17415172 | 5728 | **1.14 (1.01, 1.28)** | **0.034** | 1394 | 1.07 (0.69, 1.66) | 0.767 | 581 | 1.12 (0.62, 2) | 0.724 | 10484 | 0.97 (0.59, 1.58) | 0.911 |
| 455 | Hemorrhoids | 9001 | 17523962 | 3782 | **0.92 (0.85, 0.99)** | **0.027** | 946 | **0.87 (0.78, 0.98)** | **0.019** | 370 | 0.86 (0.69, 1.08) | 0.201 | 7585 | 0.99 (0.67, 1.46) | 0.97 |
| 456 | Chronic venous insufficiency [CVI] | 925 | 17626709 | 452 | **1.37 (1.18, 1.58)** | **<0.001** | 105 | 1.25 (0.88, 1.79) | 0.212 | 55 | **1.64 (1.18, 2.26)** | **0.003** | 770 | 0.94 (0.48, 1.84) | 0.864 |
| 459 | Other disorders of circulatory system | 2555 | 17616713 | 1168 | **1.2 (1.08, 1.34)** | **<0.001** | 327 | **1.25 (1.05, 1.48)** | **0.012** | 132 | 1.29 (0.97, 1.71) | 0.083 | 2184 | 1.08 (0.79, 1.48) | 0.649 |
| 459.9 | Circulatory disease NEC | 2174 | 17618930 | 1009 | **1.26 (1.14, 1.41)** | **<0.001** | 283 | **1.3 (1.09, 1.55)** | **0.003** | 119 | **1.41 (1.08, 1.83)** | **0.01** | 1845 | 1.02 (0.48, 2.19) | 0.956 |
| **Respiratory** | | | | | | | | | | | | | | | |
| 474 | Acute and chronic tonsillitis | 41427 | 16817602 | 18276 | **1.1 (1.07, 1.13)** | **<0.001** | 4898 | 0.97 (0.88, 1.08) | 0.619 | 1974 | 1.05 (0.92, 1.2) | 0.46 | 35152 | 0.97 (0.9, 1.05) | 0.48 |
| 474.2 | Chronic tonsillitis and adenoiditis | 27077 | 17030480 | 12170 | **1.14 (1.1, 1.17)** | **<0.001** | 3088 | 0.95 (0.87, 1.03) | 0.224 | 1305 | 1.08 (0.95, 1.22) | 0.249 | 22923 | 0.96 (0.89, 1.03) | 0.268 |
| 477 | Epistaxis or throat hemorrhage | 12337 | 17506794 | 5142 | **0.9 (0.86, 0.94)** | **<0.001** | 1284 | **0.87 (0.81, 0.94)** | **<0.001** | 518 | 0.88 (0.75, 1.03) | 0.106 | 10409 | 1.01 (0.76, 1.35) | 0.939 |
| **Digestive** | | | | | | | | | | | | | | | |
| 530.11 | GERD | 7461 | 17582158 | 3184 | 1.02 (0.86, 1.22) | 0.79 | 958 | **1.14 (1.05, 1.25)** | **0.003** | 350 | 1.07 (0.77, 1.47) | 0.708 | 6410 | **1.12 (1.03, 1.21)** | **0.007** |
| 530.3 | Stricture and stenosis of esophagus | 1798 | 17620968 | 726 | **0.85 (0.74, 0.99)** | **0.034** | 194 | 0.91 (0.55, 1.5) | 0.725 | 79 | 0.92 (0.32, 2.67) | 0.885 | 1525 | 1.06 (0.6, 1.88) | 0.856 |
| 531 | Peptic ulcer (excl. esophageal) | 16678 | 17443704 | 6864 | **0.88 (0.85, 0.92)** | **<0.001** | 1837 | 0.94 (0.84, 1.05) | 0.254 | 686 | **0.87 (0.77, 0.98)** | **0.027** | 14065 | 1.02 (0.83, 1.26) | 0.865 |
| 531.1 | Hemorrhage from gastrointestinal ulcer | 6277 | 17583751 | 2589 | **0.86 (0.8, 0.92)** | **<0.001** | 614 | **0.82 (0.72, 0.93)** | **0.002** | 254 | 0.84 (0.65, 1.08) | 0.166 | 5331 | 1.07 (0.9, 1.29) | 0.443 |
| 531.2 | Gastric ulcer | 6745 | 17555312 | 2783 | **0.89 (0.83, 0.96)** | **0.002** | 750 | 0.95 (0.72, 1.25) | 0.734 | 276 | 0.87 (0.67, 1.13) | 0.3 | 5646 | 0.97 (0.72, 1.31) | 0.849 |
| 531.3 | Duodenal ulcer | 4534 | 17555684 | 1834 | **0.87 (0.8, 0.95)** | **0.001** | 519 | 0.98 (0.47, 2.06) | 0.965 | 194 | 0.91 (0.54, 1.51) | 0.722 | 3837 | 1.05 (0.75, 1.46) | 0.802 |
| 550 | Abdominal hernia | 47761 | 16976073 | 20831 | 1 (0.86, 1.15) | 0.97 | 5112 | **0.94 (0.89, 0.99)** | **0.014** | 2083 | 0.96 (0.82, 1.13) | 0.645 | 40279 | 1.01 (0.83, 1.22) | 0.959 |
| 550.1 | Inguinal hernia | 29335 | 17183189 | 12729 | 0.98 (0.9, 1.07) | 0.671 | 3140 | **0.93 (0.87, 1)** | **0.046** | 1256 | 0.94 (0.8, 1.1) | 0.432 | 24703 | 1 (0.93, 1.07) | 0.97 |
| 562 | Diverticulosis and diverticulitis | 16569 | 17515568 | 6992 | **0.91 (0.86, 0.96)** | **0.001** | 1671 | **0.87 (0.79, 0.96)** | **0.005** | 669 | 0.86 (0.72, 1.02) | 0.088 | 13879 | 0.98 (0.75, 1.28) | 0.885 |
| 562.1 | Diverticulosis | 16569 | 17515568 | 6992 | **0.91 (0.86, 0.96)** | **0.001** | 1671 | **0.87 (0.79, 0.96)** | **0.005** | 669 | 0.86 (0.72, 1.02) | 0.088 | 13879 | 0.98 (0.75, 1.28) | 0.885 |
| 571.81 | Portal hypertension | 1101 | 17627608 | 463 | 0.94 (0.67, 1.3) | 0.709 | 141 | 1.11 (0.69, 1.79) | 0.691 | 31 | **0.61 (0.4, 0.94)** | **0.026** | 926 | 0.99 (0.52, 1.88) | 0.97 |
| 574 | Cholelithiasis and cholecystitis | 31530 | 17299206 | 13940 | **1.05 (1, 1.11)** | **0.045** | 3533 | 1 (0.83, 1.21) | 0.97 | 1409 | 1.02 (0.6, 1.73) | 0.958 | 26641 | 1.01 (0.77, 1.32) | 0.949 |
| 575.2 | Obstruction of bile duct | 1593 | 17626684 | 779 | **1.29 (1.13, 1.48)** | **<0.001** | 171 | 1.12 (0.64, 1.95) | 0.697 | 73 | 1.19 (0.56, 2.53) | 0.664 | 1359 | 1.1 (0.7, 1.71) | 0.7 |
| 578 | Gastrointestinal hemorrhage | 20111 | 17502200 | 8543 | **0.94 (0.89, 0.99)** | **0.014** | 2141 | **0.91 (0.84, 0.99)** | **0.027** | 824 | 0.88 (0.77, 1) | 0.055 | 16975 | 1.01 (0.79, 1.3) | 0.923 |
| 578.8 | Hemorrhage of rectum and anus | 11095 | 17555331 | 4689 | **0.93 (0.87, 1)** | **0.038** | 1171 | **0.89 (0.8, 1)** | **0.047** | 457 | 0.87 (0.71, 1.07) | 0.201 | 9357 | 1 (0.8, 1.26) | 0.97 |
| **Genitourinary** | | | | | | | | | | | | | | | |
| 614.1 | Pelvic peritoneal adhesions, female (postoperative) (postinfection) | 1281 | 10484656 | 561 | 1.1 (0.85, 1.43) | 0.455 | 152 | 1.1 (0.63, 1.9) | 0.751 | 76 | **1.4 (1.02, 1.93)** | **0.038** | 1086 | 1.02 (0.42, 2.45) | 0.97 |
| 614.51 | Cervicitis and endocervicitis | 660 | 10488791 | 279 | 1.02 (0.5, 2.09) | 0.952 | 79 | 1.14 (0.72, 1.8) | 0.6 | 41 | **1.44 (1.05, 1.98)** | **0.025** | 568 | 1.16 (0.86, 1.58) | 0.332 |
| 618.2 | Uterine/Uterovaginal prolapse | 3351 | 10475337 | 1441 | 1.04 (0.82, 1.33) | 0.751 | 415 | **1.19 (1.02, 1.39)** | **0.027** | 173 | 1.23 (0.94, 1.59) | 0.129 | 2794 | 0.95 (0.69, 1.3) | 0.773 |
| 622.2 | Mucous polyp of cervix | 1401 | 10498802 | 651 | **1.22 (1.07, 1.39)** | **0.002** | 162 | 1.11 (0.7, 1.75) | 0.672 | 71 | 1.24 (0.79, 1.96) | 0.361 | 1179 | 0.97 (0.49, 1.93) | 0.938 |
| 626.12 | Excessive or frequent menstruation | 10504 | 10375823 | 4270 | **0.91 (0.85, 0.97)** | **0.003** | 1187 | 0.91 (0.79, 1.06) | 0.221 | 490 | 0.96 (0.55, 1.67) | 0.9 | 8850 | 0.97 (0.75, 1.25) | 0.823 |
| **Pregnancy Complications** | | | | | | | | | | | | | | | |
| 634.3 | Ectopic pregnancy | 5034 | 10426967 | 2079 | 1 (0.87, 1.16) | 0.97 | 673 | **1.12 (1.02, 1.22)** | **0.015** | 260 | 1.13 (0.96, 1.33) | 0.136 | 4269 | 0.99 (0.7, 1.39) | 0.949 |
| 643 | Excessive vomiting in pregnancy | 4314 | 10470323 | 1696 | 0.95 (0.83, 1.07) | 0.391 | 633 | **1.15 (1.03, 1.28)** | **0.011** | 227 | 1.11 (0.84, 1.48) | 0.474 | 3678 | 1 (0.89, 1.11) | 0.97 |
| 647.3 | Major puerperal infection | 274 | 10504689 | 119 | 1.12 (0.68, 1.84) | 0.671 | 42 | **1.44 (1.04, 1.99)** | **0.028** | 10 | 0.87 (0.12, 6.43) | 0.898 | 219 | **0.72 (0.56, 0.93)** | **0.01** |
| 649.1 | Diabetes or abnormal glucose tolerance complicating pregnancy | 5053 | 10480682 | 2001 | 0.98 (0.48, 2.03) | 0.966 | 773 | 1.22 (0.98, 1.52) | 0.073 | 275 | 1.19 (0.7, 2.04) | 0.527 | 4446 | **1.26 (1.03, 1.55)** | **0.026** |
| 654.1 | Abnormality of organs and soft tissues of pelvis complicating pregnancy, childbirth, or the puerperium | 5842 | 10479533 | 2339 | **0.94 (0.9, 0.99)** | **0.029** | 808 | 1.05 (0.94, 1.17) | 0.364 | 255 | 0.91 (0.78, 1.07) | 0.254 | 5036 | **1.08 (1.01, 1.15)** | **0.029** |
| 654.2 | Rhesus isoimmunization in pregnancy | 808 | 10503190 | 361 | 1.06 (0.72, 1.57) | 0.784 | 82 | 0.82 (0.59, 1.14) | 0.24 | 31 | 0.82 (0.39, 1.75) | 0.629 | 485 | **0.26 (0.24, 0.29)** | **<0.001** |
| 656.1 | Isoimmunization of fetus or newborn | 508 | 482914* | 330 | **4.49 (1.96, 10.24)**** | **<0.001** | 96 | **4.2 (1.38, 12.73)**** | **0.011** | 10 | 1.18 (0, 2997.98)** | 0.97 | 436 | 1.03 (0.21, 5.13)** | 0.97 |
| **Dermatologic** | | | | | | | | | | | | | | | |
| 704 | Diseases of hair and hair follicles | 4102 | 17578353 | 1728 | 1.04 (0.8, 1.36) | 0.791 | 572 | **1.21 (1.06, 1.38)** | **0.006** | 195 | 1.08 (0.55, 2.09) | 0.841 | 3499 | 1.04 (0.66, 1.63) | 0.883 |
| 704.2 | Hirsutism | 1778 | 17608888 | 717 | 1.04 (0.66, 1.63) | 0.882 | 281 | **1.36 (1.19, 1.57)** | **<0.001** | 104 | **1.34 (1.03, 1.74)** | **0.028** | 1539 | 1.13 (0.87, 1.46) | 0.377 |
| **Musculoskeletal** | | | | | | | | | | | | | | | |
| 727 | Other disorders of synovium, tendon, and bursa | 30487 | 17272753 | 13190 | 1 (0.88, 1.14) | 0.97 | 3348 | **0.94 (0.88, 0.99)** | **0.028** | 1370 | 0.98 (0.73, 1.32) | 0.912 | 25643 | 0.97 (0.9, 1.04) | 0.364 |
| 727.7 | Contracture of tendon (sheath) | 391 | 17628006 | 157 | 0.94 (0.32, 2.69) | 0.909 | 53 | 1.11 (0.25, 4.88) | 0.898 | 20 | 1.09 (0.05, 24.22) | 0.958 | 307 | **0.65 (0.47, 0.9)** | **0.009** |
| 740 | Osteoarthrosis | 53711 | 17187748 | 23474 | 1 (1, 1) | 0.97 | 5629 | **0.95 (0.9, 0.99)** | **0.022** | 2411 | 1.01 (0.74, 1.38) | 0.956 | 44931 | 0.97 (0.93, 1.01) | 0.131 |
| 740.11 | Osteoarthrosis, localized, primary | 40386 | 17333061 | 17695 | 1 (0.9, 1.11) | 0.97 | 4178 | **0.94 (0.9, 0.98)** | **0.006** | 1761 | 0.98 (0.78, 1.22) | 0.861 | 33774 | 0.97 (0.92, 1.02) | 0.192 |
| 741 | Symptoms and disorders of the joints | 5085 | 17578450 | 2149 | 0.95 (0.81, 1.11) | 0.501 | 531 | **0.82 (0.72, 0.94)** | **0.005** | 220 | 0.89 (0.6, 1.33) | 0.578 | 4314 | 1 (0.82, 1.24) | 0.97 |
| 742 | Derangement of joint, non-traumatic | 16652 | 17438176 | 7225 | 1.01 (0.83, 1.23) | 0.925 | 1811 | **0.9 (0.83, 0.97)** | **0.009** | 748 | 0.97 (0.64, 1.46) | 0.883 | 14005 | 0.95 (0.87, 1.04) | 0.3 |
| 742.9 | Other derangement of joint | 13669 | 17490182 | 5892 | 1 (0.94, 1.06) | 0.97 | 1495 | **0.9 (0.83, 0.99)** | **0.027** | 619 | 0.97 (0.62, 1.52) | 0.908 | 11485 | 0.95 (0.86, 1.04) | 0.268 |
| **Congenital Anomalies** | | | | | | | | | | | | | | | |
| 747 | Cardiac and circulatory congenital anomalies | 15297 | 482914* | 6831 | **1.07 (1.01, 1.14)**** | **0.022** | 1685 | 0.98 (0.71, 1.34)** | 0.893 | 710 | 1.05 (0.75, 1.48)** | 0.784 | 12876 | 0.98 (0.77, 1.25)** | 0.885 |
| 755 | Congenital anomalies of limbs | 6557 | 482914* | 2722 | 0.97 (0.79, 1.19)** | 0.773 | 798 | 0.94 (0.74, 1.19)** | 0.607 | 298 | 0.93 (0.57, 1.53)** | 0.802 | 5478 | **0.88 (0.8, 0.97)**** | **0.013** |
| 755.61 | Congenital hip dysplasia and deformity | 2823 | 482914* | 1184 | 0.97 (0.64, 1.47)** | 0.887 | 321 | 0.85 (0.66, 1.1)** | 0.231 | 120 | 0.86 (0.48, 1.53)** | 0.612 | 2328 | **0.81 (0.71, 0.94)**** |  |

a. Statistically significant IRRs are marked with bold (FDR adjusted p-value<0.05).
b. The IRRs are adjusted for age, sex, interaction between age and sex, and birth year.
c. Blood group O and the RhD negative blood group was used as a reference, respectively.
d. The FDR adjusted p-values and 95% confidence intervals are presented.
e. FDR adjusted p-values above 0.97 were set to 0.97 to avoid exploding adjusted confidence intervals.
f. Phecodes are divided by PheWAS disease categories.
g. The number of events and the follow-up time in person-years for each Phecode is also presented.
h. For study results of congenital Phecodes estimates marked with ** are prevalence ratios instead of IRRs and the corresponding person-year marked with * are the size of the cohort.
