# Supplementary material for: Associations of ABO and Rhesus D blood groups with phenome-wide disease incidence: A 41-year retrospective cohort study of 482,914 patients
Source: eLife. 2023 Mar 9;12:e83116. doi: 10.7554/eLife.83116 (PMC10042530; doi:10.7554/eLife.83116)
Supplement: Supplementary file 7. — a. Statistically significant IRRs are marked with bold (FDR adjusted P-value <0.05). b. The IRRs are adjusted for age, sex, interaction between age and sex, and birth year. c. Blood group O and the RhD negative blood group was used as a reference, respectively. d. The FDR adjusted p-values and 95% confidence intervals are presented. e. FDR adjusted p-values above 0.97 were set to 0.97 to avoid exploding adjusted confidence intervals. f. Phecodes are divided by PheWAS disease categories. g. The number of events and the follow-up time in person-years for each Phecode is also presented. h. For study results of congenital Phecodes estimates marked with ** are prevalence ratios instead of IRRs and the corresponding person-year marked with * are the size of the cohort. [file elife-83116-supp7.docx]

## Supplementary file 7: Associations between the ABO/RhD blood groups and Phecode incidence rate ratios of all analyzed Phecodes with blood group O and RhD negative as the reference, respectively.

|  | | | | **A** | | | **B** | | | **AB** | | | **Rhesus** | | |
| --- | --- | --- | --- | --- | --- | --- | --- | --- | --- | --- | --- | --- | --- | --- | --- |
| **Phecode** | **Phenotype** | **Total events** | **Person-years** | **Events** | **IRR (95%CI)** | **P-value** | **Events** | **IRR (95%CI)** | **P-value** | **Events** | **IRR (95%CI)** | **P-value** | **Events** | **IRR (95%CI)** | **P-value** |
| **Infectious Diseases** | | | | | | | | | | | | | | | |
| 008 | Intestinal infection | 12259 | 17508444 | 5294 | 1.02 (0.87, 1.21) | 0.788 | 1472 | 1.05 (0.87, 1.27) | 0.62 | 549 | 1 (0.96, 1.04) | 0.97 | 10345 | 0.99 (0.68, 1.44) | 0.958 |
| 008.5 | Bacterial enteritis | 3288 | 17586394 | 1386 | 0.99 (0.67, 1.46) | 0.97 | 411 | 1.07 (0.55, 2.09) | 0.859 | 153 | 1.02 (0.34, 3.09) | 0.97 | 2739 | 0.9 (0.66, 1.24) | 0.543 |
| 008.51 | Intestinal e.coli | 141 | 17630155 | 66 | 1.07 (0.1, 11.12) | 0.959 | 14 | 0.83 (0.04, 15.42) | 0.909 | 2 | 0.3 (0.01, 12.23) | 0.539 | 119 | 0.99 (0.59, 1.65) | 0.97 |
| 008.52 | Intestinal infection due to C. difficile | 3776 | 17621694 | 1636 | 1.01 (0.74, 1.37) | 0.97 | 427 | 1.04 (0.61, 1.8) | 0.885 | 175 | 1.06 (0.46, 2.42) | 0.901 | 3164 | 0.98 (0.55, 1.73) | 0.949 |
| 008.6 | Viral Enteritis | 4398 | 17579904 | 1964 | 1.1 (0.99, 1.22) | 0.066 | 530 | 1.05 (0.73, 1.53) | 0.794 | 190 | 0.98 (0.43, 2.23) | 0.97 | 3765 | 1.07 (0.85, 1.35) | 0.576 |
| 008.7 | Intestinal infection due to protozoa | 446 | 17622771 | 168 | 0.84 (0.5, 1.42) | 0.535 | 62 | 1.09 (0.27, 4.43) | 0.908 | 24 | 1.11 (0.11, 11.62) | 0.939 | 357 | 0.71 (0.5, 1.02) | 0.063 |
| 010 | Tuberculosis | 2101 | 17603440 | 831 | 0.94 (0.64, 1.38) | 0.767 | 326 | **1.36 (1.13, 1.64)** | **0.001** | 102 | 1.09 (0.36, 3.33) | 0.885 | 1852 | **1.36 (1.12, 1.65)** | **0.002** |
| 031 | Diseases due to other mycobacteria | 131 | 17630926 | 58 | 1.01 (0.78, 1.3) | 0.97 | 15 | 0.94 (0.04, 21.09) | 0.97 | 3 | 0.49 (0.04, 5.95) | 0.584 | 108 | 0.86 (0.17, 4.33) | 0.861 |
| 038 | Septicemia | 25404 | 17504470 | 10903 | 0.97 (0.91, 1.04) | 0.366 | 2803 | 0.98 (0.79, 1.22) | 0.86 | 1087 | 0.94 (0.78, 1.15) | 0.575 | 21411 | 1.01 (0.82, 1.25) | 0.935 |
| 038.1 | Gram negative septicemia | 3816 | 17606498 | 1630 | 0.98 (0.67, 1.42) | 0.915 | 437 | 1.02 (0.47, 2.22) | 0.97 | 172 | 1 (0.89, 1.13) | 0.97 | 3239 | 1.05 (0.74, 1.5) | 0.79 |
| 038.2 | Gram positive septicemia | 2088 | 17621191 | 931 | 1.07 (0.67, 1.7) | 0.796 | 237 | 1.05 (0.37, 2.96) | 0.929 | 96 | 1.07 (0.24, 4.85) | 0.935 | 1767 | 1.03 (0.36, 2.92) | 0.958 |
| 041 | Bacterial infection NOS | 19973 | 17513535 | 8653 | 1.02 (0.88, 1.19) | 0.771 | 2309 | 0.99 (0.71, 1.4) | 0.97 | 916 | 1.02 (0.49, 2.12) | 0.97 | 16926 | 1.01 (0.69, 1.48) | 0.964 |
| 041.1 | Staphylococcus infections | 1539 | 17622977 | 666 | 1.02 (0.59, 1.77) | 0.938 | 183 | 1.04 (0.46, 2.33) | 0.935 | 70 | 1.02 (0.45, 2.31) | 0.97 | 1313 | 1.06 (0.64, 1.77) | 0.826 |
| 041.2 | Streptococcus infection | 6160 | 17592761 | 2684 | 1.07 (0.68, 1.67) | 0.791 | 731 | 0.96 (0.33, 2.79) | 0.943 | 292 | 1.03 (0.3, 3.56) | 0.97 | 5254 | 1.01 (0.63, 1.62) | 0.97 |
| 041.4 | E. coli | 284 | 17630853 | 128 | 1.07 (0.24, 4.77) | 0.938 | 33 | 1.07 (0.06, 19.23) | 0.966 | 9 | 0.73 (0.03, 19.34) | 0.861 | 235 | 0.9 (0.18, 4.44) | 0.906 |
| 053 | Herpes zoster | 2573 | 17612188 | 1074 | 0.96 (0.66, 1.38) | 0.819 | 319 | 1.09 (0.71, 1.69) | 0.708 | 112 | 0.96 (0.22, 4.12) | 0.963 | 2163 | 0.98 (0.47, 2.07) | 0.97 |
| 053.1 | Herpes zoster with nervous system complications | 282 | 17629544 | 122 | 1.02 (0.37, 2.83) | 0.97 | 31 | 1.03 (0.29, 3.67) | 0.97 | 16 | 1.32 (0.07, 25.51) | 0.864 | 245 | 1.25 (0.24, 6.56) | 0.801 |
| 054 | Herpes simplex | 3683 | 17578407 | 1553 | 0.99 (0.54, 1.79) | 0.967 | 434 | 0.93 (0.6, 1.45) | 0.767 | 165 | 0.94 (0.38, 2.34) | 0.901 | 3122 | 0.98 (0.49, 1.96) | 0.956 |
| 070 | Viral hepatitis | 6596 | 17557078 | 2613 | **0.9 (0.83, 0.98)** | **0.02** | 920 | **1.16 (1.03, 1.31)** | **0.013** | 299 | 0.97 (0.4, 2.36) | 0.954 | 5679 | 1.12 (0.99, 1.27) | 0.064 |
| 070.1 | Viral hepatitis A | 404 | 17624712 | 170 | 1.05 (0.22, 4.89) | 0.956 | 56 | 1.24 (0.44, 3.52) | 0.693 | 23 | 1.32 (0.28, 6.18) | 0.734 | 344 | 1.03 (0.22, 4.75) | 0.97 |
| 070.2 | Viral hepatitis B | 1664 | 17613572 | 567 | **0.77 (0.66, 0.91)** | **0.002** | 309 | **1.49 (1.25, 1.77)** | **<0.001** | 85 | 1.07 (0.26, 4.36) | 0.929 | 1471 | **1.36 (1.07, 1.71)** | **0.011** |
| 070.3 | Viral hepatitis C | 1836 | 17616684 | 755 | 0.93 (0.59, 1.46) | 0.761 | 230 | 1.04 (0.33, 3.34) | 0.947 | 80 | 0.94 (0.14, 6.33) | 0.953 | 1568 | 1.07 (0.48, 2.37) | 0.883 |
| 070.4 | Chronic hepatitis | 936 | 17623005 | 368 | 0.86 (0.52, 1.43) | 0.576 | 125 | 1.08 (0.24, 4.76) | 0.929 | 34 | 0.75 (0.16, 3.44) | 0.723 | 797 | 1.05 (0.22, 4.88) | 0.959 |
| 070.9 | Hepatitis NOS | 1713 | 17615330 | 737 | 1 (0.9, 1.11) | 0.97 | 201 | 1.02 (0.36, 2.91) | 0.97 | 74 | 0.96 (0.13, 7.24) | 0.97 | 1465 | 1.09 (0.64, 1.84) | 0.767 |
| 071 | Human immunodeficiency virus [HIV] disease | 1182 | 17620808 | 446 | 0.84 (0.59, 1.19) | 0.327 | 164 | 1.1 (0.35, 3.41) | 0.883 | 64 | 1.12 (0.2, 6.16) | 0.906 | 1055 | **1.49 (1.04, 2.14)** | **0.029** |
| 071.1 | HIV infection, symptomatic | 1182 | 17620808 | 446 | 0.84 (0.59, 1.19) | 0.327 | 164 | 1.1 (0.35, 3.41) | 0.883 | 64 | 1.12 (0.2, 6.16) | 0.906 | 1055 | **1.49 (1.04, 2.14)** | **0.029** |
| 078 | Viral warts & HPV | 8674 | 17507344 | 3727 | 1.02 (0.49, 2.09) | 0.97 | 981 | 0.9 (0.01, 155.11) | 0.97 | 397 | 0.97 (0.22, 4.29) | 0.97 | 7335 | 0.96 (0.15, 6.27) | 0.97 |
| 079 | Viral infection | 25044 | 17304684 | 10735 | 1.03 (0.94, 1.12) | 0.544 | 3003 | 0.97 (0.8, 1.19) | 0.815 | 1188 | 1.02 (0.67, 1.56) | 0.935 | 21285 | 1 (0.89, 1.11) | 0.97 |
| 079.1 | Varicella infection | 1002 | 17611821 | 422 | 1.1 (0.01, 86.98) | 0.97 | 165 | 1.39 (0, 128690.37) | 0.959 | 40 | 0.91 (0.01, 87.85) | 0.97 | 864 | 1.07 (0.04, 31.38) | 0.97 |
| 079.2 | Infectious mononucleosis | 3492 | 17567432 | 1567 | 1.1 (0.71, 1.7) | 0.68 | 368 | 0.86 (0.42, 1.75) | 0.693 | 173 | 1.08 (0.2, 5.85) | 0.935 | 2926 | 0.9 (0.51, 1.6) | 0.733 |
| 080 | Postoperative infection | 11784 | 17528016 | 5010 | 0.97 (0.83, 1.14) | 0.708 | 1320 | 0.97 (0.66, 1.42) | 0.883 | 537 | 1 (0.9, 1.11) | 0.97 | 9928 | 0.99 (0.65, 1.51) | 0.97 |
| 081 | Infection/inflammation of internal prosthetic device; implant; and graft | 1473 | 17622812 | 639 | 0.96 (0.63, 1.45) | 0.849 | 138 | 0.79 (0.62, 1.02) | 0.07 | 63 | 0.91 (0.32, 2.61) | 0.874 | 1234 | 0.96 (0.54, 1.72) | 0.903 |
| 090 | Sexually transmitted infections (not HIV or hepatitis) | 2671 | 17600535 | 1146 | 1.02 (0.41, 2.53) | 0.97 | 304 | 0.92 (0.35, 2.47) | 0.883 | 136 | 1.09 (0.26, 4.64) | 0.916 | 2286 | 1.05 (0.41, 2.66) | 0.93 |
| 090.2 | Gonococcal infections | 1223 | 17614204 | 524 | 1.01 (0.63, 1.63) | 0.97 | 138 | 0.92 (0.09, 9.34) | 0.948 | 61 | 1.06 (0.06, 18.99) | 0.97 | 1053 | 1.1 (0.2, 6.01) | 0.923 |
| 090.3 | Venereal diseases due to Chlamydia trachomatis | 1005 | 17624635 | 443 | 1.08 (0.75, 1.56) | 0.706 | 112 | 0.89 (0.5, 1.58) | 0.711 | 50 | 1.06 (0.22, 4.99) | 0.949 | 844 | 0.9 (0.58, 1.41) | 0.67 |
| 110 | Dermatophytosis / Dermatomycosis | 3697 | 17611134 | 1505 | 0.97 (0.45, 2.08) | 0.949 | 515 | 1.15 (0.73, 1.81) | 0.562 | 183 | 1.08 (0.25, 4.58) | 0.929 | 3161 | 1.05 (0.45, 2.43) | 0.924 |
| 110.1 | Dermatophytosis | 897 | 17625587 | 378 | 0.99 (0.51, 1.91) | 0.97 | 115 | 1.08 (0.24, 4.84) | 0.924 | 37 | 0.9 (0.06, 14.33) | 0.946 | 775 | 1.15 (0.45, 2.92) | 0.781 |
| 110.11 | Dermatophytosis of nail | 210 | 17630071 | 82 | 0.84 (0.41, 1.71) | 0.635 | 21 | 0.81 (0.19, 3.53) | 0.79 | 14 | 1.37 (0.32, 5.89) | 0.686 | 176 | 0.96 (0.11, 8.04) | 0.97 |
| 110.12 | Althete's foot | 245 | 17629429 | 101 | 0.94 (0.23, 3.88) | 0.938 | 30 | 1.04 (0.16, 6.98) | 0.97 | 12 | 1.06 (0.06, 18.57) | 0.97 | 206 | 0.97 (0.21, 4.38) | 0.97 |
| 110.13 | Dermatophytosis of the body | 211 | 17630531 | 84 | 1 (0.81, 1.25) | 0.97 | 38 | 1.64 (0.95, 2.85) | 0.076 | 9 | 1.01 (0.78, 1.29) | 0.97 | 182 | 1.13 (0.2, 6.47) | 0.898 |
| 110.2 | Dermatomycoses | 542 | 17626754 | 213 | 0.9 (0.43, 1.89) | 0.8 | 75 | 1.13 (0.34, 3.72) | 0.849 | 28 | 1.1 (0.1, 12.04) | 0.943 | 467 | 1.12 (0.35, 3.54) | 0.86 |
| 112 | Candidiasis | 4742 | 17589391 | 2014 | 0.98 (0.7, 1.38) | 0.909 | 549 | 0.98 (0.46, 2.1) | 0.97 | 211 | 0.97 (0.34, 2.77) | 0.956 | 4044 | 1.06 (0.8, 1.41) | 0.691 |
| 112.3 | Candidiasis of skin and nails | 108 | 17630849 | 47 | 1.18 (0.15, 9.53) | 0.885 | 18 | 1.7 (0.42, 6.84) | 0.463 | 5 | 1.2 (0, 626.77) | 0.959 | 94 | 1.24 (0.08, 19.24) | 0.885 |
| 117 | Mycoses | 756 | 17628155 | 324 | 0.99 (0.74, 1.33) | 0.97 | 90 | 1.05 (0.35, 3.2) | 0.933 | 33 | 0.97 (0.29, 3.33) | 0.97 | 639 | 1.01 (0.54, 1.9) | 0.97 |
| 117.4 | Aspergillosis | 302 | 17630483 | 143 | 1.26 (0.81, 1.95) | 0.314 | 37 | 1.23 (0.45, 3.36) | 0.7 | 14 | 1.18 (0.12, 12.06) | 0.898 | 256 | 1.03 (0.28, 3.71) | 0.97 |
| 130 | Spirochetal infection | 1883 | 17612135 | 809 | 1.01 (0.69, 1.48) | 0.97 | 233 | 1.07 (0.53, 2.16) | 0.861 | 75 | 0.88 (0.33, 2.37) | 0.813 | 1564 | 0.9 (0.65, 1.23) | 0.503 |
| 130.1 | Lyme disease | 1424 | 17617867 | 604 | 0.96 (0.55, 1.7) | 0.909 | 174 | 1.03 (0.34, 3.12) | 0.96 | 49 | 0.74 (0.39, 1.4) | 0.362 | 1183 | 0.9 (0.61, 1.33) | 0.611 |
| 131 | Protozoan infection | 392 | 17622798 | 184 | 1.15 (0.48, 2.76) | 0.767 | 36 | 0.82 (0.14, 4.74) | 0.835 | 18 | 1.05 (0.11, 10.24) | 0.97 | 330 | 0.97 (0.25, 3.75) | 0.97 |
| 132 | Infestation (lice, mites) | 361 | 17623576 | 143 | 0.84 (0.43, 1.65) | 0.628 | 40 | 0.85 (0.16, 4.51) | 0.859 | 15 | 0.82 (0.05, 12.9) | 0.898 | 307 | 1.03 (0.24, 4.48) | 0.97 |
| 132.1 | Pediculosis and phthirus infestation | 120 | 17629686 | 46 | 0.76 (0.24, 2.46) | 0.662 | 9 | 0.53 (0.1, 3.01) | 0.487 | 7 | 1.08 (0.03, 34.69) | 0.97 | 100 | 0.91 (0.04, 21.56) | 0.956 |
| 133 | Arthropod-borne diseases | 667 | 17620641 | 250 | 0.82 (0.46, 1.45) | 0.503 | 98 | 1.16 (0.31, 4.29) | 0.835 | 27 | 0.83 (0.06, 11.93) | 0.898 | 579 | 1.19 (0.4, 3.5) | 0.767 |
| 134 | Helminthiases | 658 | 17622251 | 277 | 1.05 (0.37, 3.03) | 0.929 | 91 | 1.2 (0.51, 2.79) | 0.691 | 36 | 1.25 (0.31, 4.99) | 0.767 | 575 | 1.23 (0.61, 2.47) | 0.575 |
| 134.1 | Intestinal helminthiases | 301 | 17626655 | 116 | 0.93 (0.11, 7.86) | 0.953 | 51 | 1.44 (0.49, 4.22) | 0.519 | 14 | 1.03 (0.21, 5.1) | 0.97 | 269 | 1.5 (0.44, 5.05) | 0.525 |
| 136 | Other infectious and parasitic diseases | 475 | 17626438 | 202 | 1.06 (0.35, 3.15) | 0.928 | 70 | 1.35 (0.78, 2.32) | 0.284 | 21 | 1.04 (0.17, 6.23) | 0.97 | 393 | 0.87 (0.4, 1.89) | 0.742 |
| **Neoplasms** | | | | | | | | | | | | | | | |
| 145 | Cancer of mouth | 1890 | 17621574 | 819 | 0.98 (0.43, 2.24) | 0.966 | 204 | 0.98 (0.31, 3.04) | 0.97 | 82 | 0.97 (0.26, 3.62) | 0.97 | 1567 | 0.92 (0.58, 1.46) | 0.73 |
| 145.1 | Cancer of lip | 153 | 17630366 | 71 | 1.06 (0.11, 10.36) | 0.965 | 14 | 0.84 (0.06, 12.64) | 0.909 | 5 | 0.74 (0.01, 53.03) | 0.898 | 131 | 1.14 (0.13, 9.71) | 0.913 |
| 145.2 | Cancer of tongue | 606 | 17629055 | 257 | 0.99 (0.68, 1.45) | 0.97 | 74 | 1.14 (0.59, 2.22) | 0.708 | 32 | 1.23 (0.5, 3.04) | 0.67 | 483 | **0.74 (0.6, 0.92)** | **0.007** |
| 145.3 | Cancer of major salivary glands | 120 | 17631140 | 58 | 1.14 (0.17, 7.69) | 0.901 | 9 | 0.7 (0.04, 12.55) | 0.818 | 5 | 0.96 (0.17, 5.39) | 0.97 | 102 | 1.07 (0.05, 25.31) | 0.97 |
| 145.4 | Cancer of the gums | 176 | 17630883 | 82 | 1.03 (0.27, 3.94) | 0.97 | 14 | 0.71 (0.11, 4.43) | 0.724 | 5 | 0.62 (0.02, 19.34) | 0.8 | 153 | 1.27 (0.25, 6.29) | 0.786 |
| 145.5 | Cancer of the mouth floor | 366 | 17630189 | 162 | 0.99 (0.52, 1.87) | 0.97 | 38 | 0.93 (0.03, 32.38) | 0.97 | 12 | 0.73 (0.01, 39.48) | 0.885 | 306 | 0.97 (0.18, 5.18) | 0.97 |
| 149 | Cancer of larynx, pharynx, nasal cavities | 3451 | 17614285 | 1516 | 1.01 (0.79, 1.28) | 0.97 | 364 | 0.96 (0.49, 1.9) | 0.924 | 158 | 1.04 (0.34, 3.18) | 0.949 | 2864 | 0.92 (0.72, 1.18) | 0.534 |
| 149.1 | Cancer of oropharynx | 1518 | 17625258 | 645 | 0.94 (0.42, 2.1) | 0.883 | 164 | 0.95 (0.17, 5.35) | 0.956 | 64 | 0.92 (0.06, 13.43) | 0.958 | 1266 | 0.95 (0.3, 2.94) | 0.93 |
| 149.2 | Cancer of nasopharynx | 203 | 17630710 | 86 | 0.94 (0.14, 6.2) | 0.956 | 23 | 0.98 (0.32, 3.01) | 0.97 | 8 | 0.86 (0.01, 63.29) | 0.949 | 186 | 2.04 (0.99, 4.24) | 0.054 |
| 149.3 | Cancer of hypopharynx | 608 | 17629864 | 286 | 1.15 (0.8, 1.66) | 0.455 | 62 | 1.01 (0.79, 1.29) | 0.97 | 28 | 1.13 (0.21, 6.1) | 0.898 | 501 | 0.89 (0.49, 1.59) | 0.699 |
| 149.4 | Cancer of larynx | 1164 | 17624631 | 515 | 1.05 (0.63, 1.76) | 0.866 | 123 | 1.01 (0.64, 1.6) | 0.97 | 67 | 1.36 (0.92, 2.02) | 0.126 | 973 | 0.97 (0.43, 2.2) | 0.942 |
| 149.9 | Cancer of of nasal cavities | 381 | 17629769 | 190 | 1.24 (0.85, 1.82) | 0.268 | 33 | 0.86 (0.19, 4.02) | 0.861 | 14 | 0.9 (0.04, 22.97) | 0.956 | 306 | 0.77 (0.5, 1.21) | 0.26 |
| 150 | Cancer of esophagus | 2442 | 17627330 | 1019 | 0.92 (0.81, 1.04) | 0.185 | 270 | 0.98 (0.43, 2.23) | 0.97 | 113 | 1.02 (0.48, 2.17) | 0.97 | 2033 | 0.94 (0.72, 1.24) | 0.697 |
| 151 | Cancer of stomach | 2423 | 17625251 | 1043 | 0.98 (0.59, 1.62) | 0.934 | 261 | 0.98 (0.42, 2.32) | 0.97 | 117 | 1.09 (0.44, 2.68) | 0.864 | 2050 | 1.04 (0.61, 1.78) | 0.885 |
| 153 | Colorectal cancer | 9507 | 17583178 | 4125 | 0.98 (0.75, 1.29) | 0.904 | 1049 | 1.01 (0.58, 1.78) | 0.97 | 375 | 0.89 (0.64, 1.23) | 0.483 | 7942 | 0.97 (0.73, 1.28) | 0.828 |
| 153.2 | Colon cancer | 8870 | 17587021 | 3836 | 0.97 (0.75, 1.27) | 0.86 | 975 | 1.01 (0.77, 1.32) | 0.97 | 353 | 0.89 (0.62, 1.28) | 0.542 | 7406 | 0.97 (0.73, 1.28) | 0.817 |
| 153.3 | Malignant neoplasm of rectum, rectosigmoid junction, and anus | 658 | 17627874 | 296 | 1.06 (0.44, 2.56) | 0.906 | 72 | 1.02 (0.36, 2.94) | 0.97 | 25 | 0.88 (0.09, 8.08) | 0.914 | 552 | 0.98 (0.47, 2.04) | 0.97 |
| 155 | Cancer of liver and intrahepatic bile duct | 1368 | 17629519 | 589 | 0.97 (0.56, 1.69) | 0.929 | 150 | 0.99 (0.73, 1.36) | 0.97 | 60 | 0.98 (0.46, 2.12) | 0.97 | 1173 | 1.14 (0.84, 1.54) | 0.406 |
| 155.1 | Malignant neoplasm of liver, primary | 853 | 17630406 | 370 | 0.98 (0.43, 2.21) | 0.959 | 87 | 0.92 (0.33, 2.54) | 0.883 | 41 | 1.08 (0.22, 5.18) | 0.932 | 741 | 1.25 (0.93, 1.69) | 0.135 |
| 157 | Pancreatic cancer | 2828 | 17627948 | 1396 | **1.34 (1.2, 1.49)** | **<0.001** | 312 | 1.21 (0.92, 1.59) | 0.173 | 137 | 1.31 (0.89, 1.92) | 0.173 | 2381 | 1.01 (0.52, 1.98) | 0.97 |
| 158 | Neoplasm of unspecified nature of digestive system | 811 | 17621760 | 385 | 1.14 (0.71, 1.83) | 0.609 | 69 | 0.82 (0.33, 2.05) | 0.68 | 38 | 1.11 (0.14, 9.11) | 0.93 | 700 | 1.2 (0.65, 2.23) | 0.567 |
| 159 | Malignant neoplasm of other and ill-defined sites within the digestive organs and peritoneum | 4458 | 17617336 | 1963 | 0.99 (0.75, 1.31) | 0.97 | 440 | 0.89 (0.65, 1.22) | 0.487 | 192 | 0.96 (0.3, 3.06) | 0.952 | 3713 | 0.95 (0.69, 1.3) | 0.741 |
| 159.2 | Malignant neoplasm of small intestine, including duodenum | 535 | 17629997 | 227 | 0.91 (0.5, 1.66) | 0.767 | 50 | 0.8 (0.37, 1.73) | 0.582 | 22 | 0.87 (0.12, 6.57) | 0.9 | 445 | 0.94 (0.33, 2.67) | 0.91 |
| 159.3 | Malignant neoplasm of gallbladder and extrahepatic bile ducts | 805 | 17630280 | 355 | 1.01 (0.65, 1.58) | 0.97 | 82 | 0.94 (0.39, 2.25) | 0.897 | 36 | 1.02 (0.5, 2.08) | 0.97 | 670 | 0.94 (0.51, 1.74) | 0.863 |
| 159.4 | Malignant neoplasm of retroperitoneum and peritoneum | 439 | 17629837 | 190 | 1.01 (0.69, 1.48) | 0.97 | 46 | 0.95 (0.09, 9.77) | 0.97 | 24 | 1.24 (0.06, 25.15) | 0.898 | 374 | 1.08 (0.09, 13.02) | 0.957 |
| 164 | Cancer of intrathoracic organs | 194 | 17630841 | 90 | 1.12 (0.38, 3.29) | 0.846 | 22 | 1.07 (0.07, 15.75) | 0.964 | 6 | 0.73 (0.04, 12.83) | 0.84 | 164 | 1.03 (0.3, 3.49) | 0.97 |
| 165 | Cancer within the respiratory system | 11019 | 17606902 | 4748 | 0.97 (0.85, 1.1) | 0.612 | 1203 | 0.99 (0.61, 1.61) | 0.97 | 433 | 0.87 (0.71, 1.08) | 0.211 | 9241 | 0.99 (0.67, 1.47) | 0.963 |
| 165.1 | Cancer of bronchus; lung | 10403 | 17608543 | 4477 | 0.97 (0.84, 1.11) | 0.626 | 1135 | 0.99 (0.6, 1.63) | 0.97 | 416 | 0.89 (0.69, 1.14) | 0.365 | 8718 | 0.99 (0.71, 1.37) | 0.935 |
| 170 | Cancer of bone and connective tissue | 1750 | 17621538 | 743 | 0.94 (0.55, 1.61) | 0.84 | 174 | 0.84 (0.5, 1.43) | 0.539 | 85 | 1.04 (0.17, 6.42) | 0.97 | 1484 | 1.04 (0.38, 2.85) | 0.949 |
| 170.1 | Bone cancer | 564 | 17628202 | 251 | 1.07 (0.41, 2.82) | 0.902 | 57 | 0.92 (0.16, 5.3) | 0.935 | 33 | 1.35 (0.43, 4.24) | 0.62 | 474 | 0.98 (0.35, 2.75) | 0.97 |
| 170.2 | Cancer of connective tissue | 1368 | 17624362 | 573 | 0.91 (0.58, 1.43) | 0.703 | 134 | 0.82 (0.46, 1.44) | 0.492 | 65 | 1 (0.88, 1.13) | 0.97 | 1164 | 1.06 (0.42, 2.66) | 0.909 |
| 172 | Skin cancer | 17775 | 17508297 | 7713 | 0.99 (0.81, 1.22) | 0.933 | 1923 | 0.98 (0.74, 1.3) | 0.898 | 781 | 0.99 (0.56, 1.72) | 0.962 | 14908 | 0.99 (0.79, 1.23) | 0.903 |
| 172.11 | Melanomas of skin | 6833 | 17579888 | 2972 | 1.01 (0.79, 1.29) | 0.97 | 725 | 0.94 (0.7, 1.28) | 0.724 | 328 | 1.07 (0.66, 1.74) | 0.784 | 5713 | 0.95 (0.76, 1.2) | 0.691 |
| 172.2 | Other non-epithelial cancer of skin | 11667 | 17556859 | 5067 | 0.99 (0.79, 1.22) | 0.905 | 1275 | 1 (0.82, 1.23) | 0.97 | 486 | 0.94 (0.67, 1.31) | 0.722 | 9791 | 1 (0.89, 1.11) | 0.97 |
| 172.3 | Carcinoma in situ of skin | 442 | 17629052 | 185 | 0.92 (0.46, 1.83) | 0.814 | 50 | 0.98 (0.42, 2.27) | 0.97 | 16 | 0.78 (0.16, 3.72) | 0.767 | 373 | 1.02 (0.32, 3.29) | 0.97 |
| 173 | Neoplasm of uncertain behavior of skin | 328 | 17625890 | 148 | 1.07 (0.22, 5.2) | 0.934 | 31 | 0.85 (0.09, 8.48) | 0.901 | 18 | 1.25 (0.08, 20.82) | 0.885 | 279 | 1.06 (0.07, 15.72) | 0.97 |
| 174 | Breast cancer | 13763 | 17495015 | 6013 | 1.01 (0.74, 1.37) | 0.968 | 1429 | 0.94 (0.8, 1.11) | 0.467 | 601 | 0.97 (0.57, 1.66) | 0.929 | 11510 | 0.97 (0.82, 1.14) | 0.683 |
| 174.1 | Breast cancer [female] | 1217 | 10502439 | 499 | 0.9 (0.76, 1.05) | 0.18 | 116 | 0.81 (0.64, 1.02) | 0.076 | 67 | 1.16 (0.7, 1.9) | 0.576 | 1015 | 0.94 (0.62, 1.43) | 0.796 |
| 174.11 | Malignant neoplasm of female breast | 12875 | 10388823 | 5634 | 1.01 (0.78, 1.31) | 0.933 | 1341 | 0.95 (0.77, 1.17) | 0.62 | 563 | 0.98 (0.48, 1.98) | 0.956 | 10768 | 0.97 (0.8, 1.16) | 0.728 |
| 174.3 | Neoplasm of uncertain behavior of breast | 582 | 17619322 | 246 | 0.94 (0.35, 2.52) | 0.903 | 59 | 0.89 (0.18, 4.36) | 0.892 | 26 | 0.96 (0.15, 6.07) | 0.97 | 483 | 0.93 (0.27, 3.15) | 0.909 |
| 175 | Acquired absence of breast | 2260 | 10494264 | 981 | 0.99 (0.62, 1.56) | 0.959 | 222 | 0.84 (0.7, 1) | 0.054 | 97 | 0.92 (0.49, 1.72) | 0.8 | 1925 | 1.07 (0.81, 1.41) | 0.665 |
| 180 | Cervical cancer and dysplasia | 12538 | 10308860 | 5462 | 1.03 (0.92, 1.15) | 0.623 | 1361 | **0.91 (0.83, 1)** | **0.046** | 554 | 0.96 (0.64, 1.45) | 0.861 | 10504 | 0.93 (0.86, 1.01) | 0.078 |
| 180.1 | Cervical cancer | 2003 | 10489016 | 876 | 1.05 (0.59, 1.85) | 0.883 | 225 | 1 (0.86, 1.17) | 0.97 | 94 | 1.06 (0.24, 4.62) | 0.941 | 1655 | 0.88 (0.63, 1.21) | 0.438 |
| 180.3 | Cervical intraepithelial neoplasia [CIN] [Cervical dysplasia] | 10895 | 10327107 | 4745 | 1.03 (0.9, 1.17) | 0.685 | 1182 | **0.9 (0.81, 1)** | **0.04** | 476 | 0.95 (0.67, 1.34) | 0.767 | 9128 | 0.93 (0.85, 1.01) | 0.093 |
| 182 | Malignant neoplasm of uterus | 3447 | 10477631 | 1593 | 1.13 (0.96, 1.31) | 0.133 | 342 | 0.97 (0.41, 2.29) | 0.942 | 158 | 1.09 (0.43, 2.78) | 0.863 | 2864 | 0.94 (0.65, 1.34) | 0.732 |
| 184 | Cancer of other female genital organs | 4018 | 10426769 | 1721 | 0.99 (0.52, 1.86) | 0.97 | 456 | 1.02 (0.43, 2.4) | 0.97 | 168 | 0.93 (0.22, 3.89) | 0.923 | 3320 | 0.89 (0.62, 1.28) | 0.549 |
| 184.1 | Malignant neoplasm of ovary and other uterine adnexa | 261 | 10510294 | 128 | 1.35 (0.8, 2.28) | 0.268 | 28 | 1.17 (0.16, 8.47) | 0.886 | 14 | 1.44 (0.25, 8.35) | 0.697 | 219 | 0.99 (0.6, 1.62) | 0.97 |
| 184.2 | Cancer of other female genital organs (excluding uterus and ovary) | 3144 | 10435473 | 1311 | 0.94 (0.3, 2.93) | 0.921 | 360 | 1.01 (0.73, 1.39) | 0.97 | 135 | 0.93 (0.03, 27.7) | 0.97 | 2591 | 0.88 (0.31, 2.5) | 0.823 |
| 187 | Cancer of other male genital organs | 2195 | 7094649 | 979 | 1.02 (0.5, 2.06) | 0.97 | 216 | 0.84 (0.52, 1.37) | 0.49 | 92 | 0.91 (0.22, 3.73) | 0.901 | 1829 | 0.92 (0.54, 1.56) | 0.767 |
| 187.1 | Malignant neoplasm of unspecified male genital organ | 319 | 7118064 | 137 | 0.94 (0.26, 3.4) | 0.929 | 34 | 0.93 (0.09, 9.98) | 0.959 | 12 | 0.82 (0.05, 14.21) | 0.898 | 262 | 0.87 (0.28, 2.69) | 0.823 |
| 187.2 | Malignant neoplasm of testis | 1673 | 7098534 | 755 | 1.05 (0.56, 1.96) | 0.887 | 166 | 0.85 (0.47, 1.54) | 0.6 | 73 | 0.95 (0.1, 9.09) | 0.97 | 1399 | 0.93 (0.44, 1.97) | 0.859 |
| 187.8 | Neoplasm of uncertain behavior of male genital organs | 188 | 7118255 | 89 | 1.08 (0.08, 14.41) | 0.959 | 13 | 0.63 (0.05, 8.06) | 0.732 | 9 | 1.07 (0.04, 28.79) | 0.97 | 156 | 0.93 (0.02, 34.75) | 0.97 |
| 189 | Cancer of urinary organs (incl. kidney and bladder) | 4208 | 17608664 | 1808 | 0.96 (0.71, 1.31) | 0.816 | 458 | 0.99 (0.77, 1.28) | 0.97 | 183 | 0.97 (0.25, 3.72) | 0.969 | 3500 | 0.95 (0.69, 1.29) | 0.739 |
| 189.2 | Cancer of bladder | 953 | 17626812 | 428 | 1.07 (0.47, 2.43) | 0.883 | 103 | 1.05 (0.18, 6.01) | 0.959 | 47 | 1.18 (0.22, 6.19) | 0.859 | 784 | 0.89 (0.46, 1.7) | 0.73 |
| 189.21 | Malignant neoplasm of bladder | 3371 | 17613276 | 1456 | 0.96 (0.67, 1.37) | 0.842 | 344 | 0.93 (0.58, 1.49) | 0.767 | 154 | 1.02 (0.49, 2.12) | 0.97 | 2794 | 0.93 (0.69, 1.24) | 0.624 |
| 190 | Cancer of eye | 362 | 17628463 | 158 | 0.95 (0.18, 5.1) | 0.958 | 34 | 0.8 (0.17, 3.86) | 0.796 | 13 | 0.76 (0.04, 13.55) | 0.864 | 305 | 1.01 (0.71, 1.42) | 0.97 |
| 191 | Manlignant and unknown neoplasms of brain and nervous system | 3388 | 17614639 | 1443 | 0.96 (0.56, 1.65) | 0.885 | 350 | 0.89 (0.5, 1.61) | 0.724 | 167 | 1.07 (0.28, 4.07) | 0.924 | 2835 | 0.96 (0.48, 1.92) | 0.906 |
| 191.1 | Cancer of brain and nervous system | 169 | 17630919 | 74 | 0.9 (0.09, 9.42) | 0.937 | 10 | 0.47 (0.05, 4.08) | 0.5 | 7 | 0.82 (0, 777.06) | 0.959 | 133 | 0.69 (0.18, 2.71) | 0.606 |
| 191.11 | Cancer of brain | 2498 | 17623360 | 1045 | 0.93 (0.66, 1.3) | 0.684 | 269 | 0.93 (0.43, 2.02) | 0.863 | 120 | 1.04 (0.18, 6.05) | 0.97 | 2087 | 0.95 (0.5, 1.81) | 0.883 |
| 194 | Cancer of other endocrine glands | 265 | 17630227 | 116 | 1.03 (0.23, 4.62) | 0.97 | 25 | 0.82 (0.1, 6.86) | 0.864 | 17 | 1.43 (0.25, 8.11) | 0.7 | 230 | 1.2 (0.26, 5.6) | 0.824 |
| 195 | Cancer, suspected or other | 92728 | 17159614 | 40230 | 1 (0.88, 1.12) | 0.957 | 10165 | 0.98 (0.9, 1.06) | 0.648 | 4135 | 1 (0.87, 1.14) | 0.97 | 77894 | 0.98 (0.93, 1.04) | 0.544 |
| 195.1 | Malignant neoplasm, other | 7383 | 17603424 | 3124 | 0.93 (0.87, 1.01) | 0.082 | 783 | 0.92 (0.79, 1.06) | 0.234 | 317 | 0.93 (0.66, 1.3) | 0.668 | 6084 | **0.88 (0.82, 0.94)** | **<0.001** |
| 198 | Secondary malignant neoplasm | 15007 | 17593918 | 6497 | 0.99 (0.82, 1.19) | 0.888 | 1640 | 0.99 (0.68, 1.45) | 0.97 | 649 | 0.97 (0.62, 1.51) | 0.902 | 12536 | 0.96 (0.85, 1.09) | 0.527 |
| 198.1 | Secondary malignancy of lymph nodes | 3442 | 17614741 | 1432 | 0.9 (0.8, 1.01) | 0.079 | 353 | 0.87 (0.69, 1.1) | 0.247 | 153 | 0.94 (0.4, 2.21) | 0.897 | 2865 | 0.93 (0.72, 1.21) | 0.611 |
| 198.2 | Secondary malignancy of respiratory organs | 3618 | 17625468 | 1540 | 0.96 (0.72, 1.26) | 0.767 | 414 | 1.03 (0.48, 2.19) | 0.949 | 144 | 0.88 (0.48, 1.61) | 0.698 | 3033 | 0.98 (0.51, 1.89) | 0.959 |
| 198.3 | Secondary malignant neoplasm of digestive systems | 2432 | 17627694 | 1093 | 1.04 (0.68, 1.6) | 0.859 | 247 | 0.93 (0.49, 1.75) | 0.835 | 99 | 0.92 (0.3, 2.84) | 0.898 | 2020 | 0.92 (0.65, 1.31) | 0.671 |
| 198.4 | Secondary malignant neoplasm of liver | 4039 | 17625012 | 1759 | 0.99 (0.7, 1.41) | 0.97 | 442 | 1 (0.88, 1.15) | 0.97 | 167 | 0.93 (0.23, 3.73) | 0.929 | 3419 | 1.05 (0.54, 2.03) | 0.901 |
| 198.5 | Secondary malignancy of brain/spine | 2495 | 17629359 | 1084 | 0.99 (0.75, 1.31) | 0.97 | 271 | 0.99 (0.62, 1.58) | 0.97 | 108 | 0.98 (0.3, 3.19) | 0.97 | 2084 | 0.96 (0.57, 1.61) | 0.883 |
| 198.6 | Secondary malignancy of bone | 2389 | 17628765 | 1046 | 1.03 (0.64, 1.67) | 0.901 | 286 | 1.14 (0.79, 1.64) | 0.495 | 104 | 1.02 (0.43, 2.42) | 0.97 | 1982 | 0.93 (0.63, 1.35) | 0.7 |
| 198.7 | Secondary malignant neoplasm of skin | 703 | 17629397 | 303 | 0.97 (0.49, 1.93) | 0.935 | 72 | 0.92 (0.37, 2.29) | 0.861 | 32 | 1.01 (0.72, 1.4) | 0.97 | 576 | 0.86 (0.62, 1.19) | 0.362 |
| 199 | Neoplasm of uncertain behavior | 2738 | 17605525 | 1153 | 0.96 (0.68, 1.34) | 0.805 | 321 | 1.03 (0.43, 2.45) | 0.959 | 119 | 0.95 (0.29, 3.12) | 0.942 | 2295 | 0.97 (0.56, 1.66) | 0.909 |
| 199.4 | Neurofibromatosis | 291 | 482914 | 125 | 1.06 (0.15, 7.25) | 0.96 | 35 | 1.03 (0.29, 3.61) | 0.97 | 17 | 1.31 (0.1, 17.7) | 0.849 | 247 | 1 (0.92, 1.08) | 0.97 |
| 200 | Myeloproliferative disease | 2051 | 17622258 | 871 | 0.94 (0.71, 1.25) | 0.697 | 199 | 0.86 (0.63, 1.16) | 0.327 | 107 | 1.14 (0.63, 2.08) | 0.683 | 1758 | 1.14 (0.89, 1.45) | 0.307 |
| 201 | Hodgkin's disease | 889 | 17622302 | 386 | 1.03 (0.27, 3.91) | 0.97 | 103 | 1 (0.82, 1.22) | 0.97 | 42 | 1.05 (0.1, 10.75) | 0.97 | 742 | 0.92 (0.31, 2.72) | 0.885 |
| 202 | Cancer of other lymphoid, histiocytic tissue | 4011 | 17608050 | 1755 | 1.01 (0.58, 1.76) | 0.97 | 445 | 1.01 (0.6, 1.71) | 0.97 | 173 | 0.98 (0.33, 2.87) | 0.97 | 3368 | 0.99 (0.5, 1.93) | 0.97 |
| 202.2 | Non-Hodgkins lymphoma | 3358 | 17611297 | 1481 | 1.03 (0.69, 1.53) | 0.904 | 368 | 1.01 (0.73, 1.4) | 0.97 | 148 | 1.01 (0.72, 1.4) | 0.97 | 2815 | 0.98 (0.52, 1.82) | 0.945 |
| 202.21 | Nodular lymphoma | 396 | 17629509 | 164 | 0.91 (0.27, 3.07) | 0.883 | 46 | 1.01 (0.79, 1.28) | 0.97 | 15 | 0.81 (0.04, 16.03) | 0.901 | 339 | 1.12 (0.23, 5.56) | 0.898 |
| 202.24 | Large cell lymphoma | 1587 | 17624873 | 682 | 0.98 (0.5, 1.92) | 0.965 | 189 | 1.08 (0.65, 1.8) | 0.771 | 62 | 0.88 (0.38, 2.05) | 0.782 | 1341 | 1.03 (0.52, 2.03) | 0.938 |
| 204 | Leukemia | 4423 | 17610289 | 1969 | 1.06 (0.86, 1.31) | 0.604 | 494 | 1.05 (0.58, 1.89) | 0.882 | 206 | 1.09 (0.55, 2.15) | 0.818 | 3742 | 1.04 (0.65, 1.65) | 0.89 |
| 204.1 | Lymphoid leukemia | 293 | 17629269 | 128 | 1.04 (0.2, 5.31) | 0.97 | 38 | 1.2 (0.12, 12.15) | 0.889 | 10 | 0.79 (0.01, 75.77) | 0.924 | 247 | 1.01 (0.77, 1.32) | 0.97 |
| 204.11 | Lymphoid leukemia, acute | 398 | 17629015 | 174 | 1.12 (0.53, 2.37) | 0.777 | 53 | 1.18 (0.39, 3.51) | 0.784 | 22 | 1.29 (0.32, 5.17) | 0.728 | 337 | 0.98 (0.37, 2.56) | 0.97 |
| 204.12 | Lymphoid leukemia, chronic | 1476 | 17623935 | 646 | 1.03 (0.59, 1.79) | 0.929 | 168 | 1.08 (0.6, 1.95) | 0.802 | 71 | 1.13 (0.5, 2.54) | 0.788 | 1259 | 1.11 (0.78, 1.56) | 0.578 |
| 204.2 | Myeloid leukemia | 366 | 17630591 | 158 | 1 (0.79, 1.26) | 0.97 | 39 | 0.98 (0.31, 3.08) | 0.97 | 19 | 1.18 (0.16, 8.88) | 0.883 | 308 | 1 (0.83, 1.22) | 0.97 |
| 204.21 | Myeloid leukemia, acute | 752 | 17629821 | 328 | 0.96 (0.45, 2.04) | 0.929 | 76 | 0.87 (0.42, 1.82) | 0.73 | 26 | 0.74 (0.29, 1.89) | 0.547 | 639 | 1.06 (0.44, 2.58) | 0.901 |
| 204.22 | Myeloid leukemia, chronic | 249 | 17630511 | 122 | 1.25 (0.85, 1.86) | 0.26 | 22 | 0.88 (0.17, 4.46) | 0.885 | 13 | 1.3 (0.33, 5.19) | 0.724 | 207 | 0.92 (0.28, 3.04) | 0.903 |
| 204.3 | Monocytic leukemia | 179 | 17631300 | 80 | 0.95 (0.23, 3.91) | 0.951 | 18 | 0.87 (0.14, 5.19) | 0.883 | 2 | 0.24 (0.05, 1.18) | 0.079 | 146 | 0.84 (0.36, 1.99) | 0.707 |
| 204.4 | Multiple myeloma | 1212 | 17626677 | 560 | 1.16 (0.88, 1.53) | 0.284 | 144 | 1.21 (0.74, 1.97) | 0.462 | 54 | 1.11 (0.24, 5.07) | 0.898 | 1018 | 1 (0.97, 1.03) | 0.97 |
| 208 | Benign neoplasm of colon | 18529 | 17505791 | 8012 | 0.98 (0.84, 1.14) | 0.773 | 1995 | 0.97 (0.71, 1.32) | 0.866 | 794 | 0.95 (0.62, 1.46) | 0.837 | 15590 | 1.01 (0.78, 1.29) | 0.97 |
| 210 | Benign neoplasm of lip, oral cavity, and pharynx | 4901 | 17578074 | 2167 | 1.04 (0.83, 1.3) | 0.767 | 542 | 1 (0.87, 1.15) | 0.97 | 207 | 0.96 (0.46, 2) | 0.912 | 4153 | 1.04 (0.72, 1.5) | 0.86 |
| 211 | Benign neoplasm of other parts of digestive system | 2252 | 17615941 | 985 | 1.01 (0.61, 1.66) | 0.97 | 242 | 0.97 (0.31, 2.96) | 0.956 | 100 | 1 (0.85, 1.17) | 0.97 | 1878 | 0.94 (0.52, 1.69) | 0.849 |
| 212 | Benign neoplasm of respiratory and intrathoracic organs | 3875 | 17586920 | 1691 | 1.01 (0.65, 1.56) | 0.97 | 422 | 0.98 (0.45, 2.14) | 0.958 | 176 | 1.02 (0.39, 2.68) | 0.97 | 3243 | 0.96 (0.63, 1.47) | 0.86 |
| 213 | Benign neoplasm of bone and articular cartilage | 3173 | 17580666 | 1433 | 1.09 (0.92, 1.29) | 0.309 | 352 | 0.97 (0.48, 1.97) | 0.935 | 130 | 0.92 (0.38, 2.26) | 0.87 | 2659 | 0.94 (0.68, 1.29) | 0.7 |
| 214 | Lipoma | 8639 | 17543445 | 3776 | 1.05 (0.95, 1.17) | 0.306 | 1043 | 1.11 (0.98, 1.25) | 0.106 | 413 | 1.11 (0.86, 1.43) | 0.447 | 7289 | 1 (0.97, 1.03) | 0.97 |
| 214.1 | Lipoma of skin and subcutaneous tissue | 5742 | 17582124 | 2472 | 1.04 (0.88, 1.23) | 0.697 | 717 | **1.14 (1.01, 1.28)** | **0.027** | 283 | 1.14 (0.9, 1.43) | 0.273 | 4866 | 1.03 (0.74, 1.42) | 0.883 |
| 215 | Other benign neoplasm of connective and other soft tissue | 2463 | 17605166 | 1062 | 0.99 (0.67, 1.46) | 0.97 | 285 | 0.99 (0.63, 1.57) | 0.97 | 94 | 0.83 (0.41, 1.67) | 0.616 | 2074 | 0.98 (0.4, 2.39) | 0.966 |
| 216 | Benign neoplasm of skin | 12993 | 17495431 | 5590 | 0.99 (0.76, 1.3) | 0.954 | 1399 | **0.89 (0.81, 0.98)** | **0.016** | 589 | 0.97 (0.59, 1.61) | 0.923 | 10906 | 0.95 (0.85, 1.05) | 0.327 |
| 217 | Vascular hamartomas and non-neoplastic nevi | 1418 | 17614877 | 624 | 1.05 (0.69, 1.61) | 0.817 | 156 | 0.89 (0.53, 1.52) | 0.691 | 64 | 0.97 (0.25, 3.85) | 0.97 | 1188 | 0.91 (0.61, 1.35) | 0.649 |
| 217.1 | Nevus, non-neoplastic | 312 | 17628159 | 157 | 1.36 (0.88, 2.1) | 0.171 | 35 | 1.05 (0.1, 11.15) | 0.97 | 8 | 0.63 (0.06, 6.25) | 0.708 | 268 | 1.09 (0.19, 6.36) | 0.931 |
| 218 | Benign neoplasm of uterus | 18307 | 10183854 | 7858 | 1 (0.98, 1.02) | 0.97 | 2040 | 0.99 (0.7, 1.39) | 0.955 | 843 | 1.02 (0.67, 1.56) | 0.934 | 15410 | 0.99 (0.75, 1.3) | 0.956 |
| 218.1 | Uterine leiomyoma | 17472 | 10208868 | 7466 | 0.99 (0.81, 1.22) | 0.95 | 1961 | 0.99 (0.71, 1.4) | 0.97 | 808 | 1.02 (0.68, 1.53) | 0.925 | 14709 | 0.99 (0.75, 1.31) | 0.956 |
| 218.2 | Other benign neoplasm of uterus | 1131 | 10479491 | 523 | 1.11 (0.74, 1.67) | 0.629 | 108 | 0.91 (0.29, 2.84) | 0.886 | 51 | 1.06 (0.1, 10.96) | 0.965 | 944 | 0.96 (0.31, 3.04) | 0.953 |
| 221 | Benign neoplasm of other female genital organs | 1077 | 10493577 | 480 | 1.08 (0.71, 1.65) | 0.73 | 124 | 1.03 (0.24, 4.36) | 0.97 | 44 | 0.93 (0.15, 5.8) | 0.944 | 895 | 0.9 (0.55, 1.49) | 0.706 |
| 222 | Benign neoplasm of male genital organs | 528 | 7112675 | 264 | 1.32 (0.85, 2.07) | 0.221 | 53 | 1.02 (0.38, 2.72) | 0.97 | 24 | 1.16 (0.06, 20.89) | 0.924 | 445 | 1 (0.97, 1.03) | 0.97 |
| 223 | Benign neoplasm of kidney and other urinary organs | 3991 | 17598252 | 1800 | 1.08 (0.92, 1.27) | 0.376 | 422 | 1.02 (0.42, 2.45) | 0.969 | 200 | 1.19 (0.85, 1.66) | 0.313 | 3321 | 0.95 (0.7, 1.27) | 0.721 |
| 224 | Benign neoplasm of eye | 1080 | 17622849 | 487 | 1.08 (0.66, 1.78) | 0.767 | 111 | 0.93 (0.29, 3.01) | 0.917 | 53 | 1.13 (0.26, 4.95) | 0.883 | 914 | 1.02 (0.37, 2.8) | 0.97 |
| 224.1 | Benign neoplasm of eye, uveal | 377 | 17629659 | 165 | 0.98 (0.36, 2.67) | 0.97 | 37 | 0.87 (0.1, 7.72) | 0.907 | 15 | 0.87 (0.01, 64.32) | 0.954 | 313 | 0.92 (0.13, 6.61) | 0.943 |
| 225 | Benign neoplasm of brain and other parts of nervous system | 3775 | 17599116 | 1611 | 0.97 (0.62, 1.52) | 0.901 | 418 | 0.96 (0.45, 2.02) | 0.92 | 165 | 0.95 (0.26, 3.5) | 0.947 | 3164 | 0.96 (0.55, 1.68) | 0.898 |
| 225.1 | Benign neoplasm of brain, cranial nerves, meninges | 3487 | 17602052 | 1492 | 0.97 (0.58, 1.64) | 0.924 | 386 | 0.96 (0.42, 2.21) | 0.929 | 149 | 0.93 (0.3, 2.88) | 0.91 | 2929 | 0.97 (0.43, 2.19) | 0.955 |
| 225.2 | Benign neoplasm of spinal cord, meninges | 246 | 17629018 | 103 | 1 (0.98, 1.02) | 0.97 | 32 | 1.18 (0.15, 9.49) | 0.883 | 13 | 1.21 (0.06, 25.83) | 0.91 | 200 | 0.81 (0.27, 2.41) | 0.714 |
| 227 | Benign neoplasm of other endocrine glands and related structures | 2490 | 17613786 | 1073 | 0.99 (0.56, 1.75) | 0.97 | 279 | 0.97 (0.28, 3.44) | 0.97 | 104 | 0.92 (0.22, 3.8) | 0.913 | 2074 | 0.92 (0.54, 1.59) | 0.783 |
| 227.1 | Benign neoplasm of adrenal gland | 1115 | 17625441 | 501 | 1.05 (0.11, 10.23) | 0.97 | 102 | 0.83 (0.05, 14.27) | 0.906 | 59 | 1.2 (0.02, 67.68) | 0.935 | 912 | 0.84 (0.13, 5.35) | 0.864 |
| 227.2 | Benign neoplasm of parathyroid gland | 258 | 17630094 | 117 | 1.02 (0.35, 3) | 0.97 | 26 | 0.88 (0.14, 5.36) | 0.898 | 6 | 0.51 (0.1, 2.62) | 0.425 | 212 | 0.86 (0.31, 2.39) | 0.788 |
| 227.3 | Benign neoplasm of pituitary gland and craniopharyngeal duct (pouch) | 960 | 17623085 | 384 | 0.89 (0.53, 1.49) | 0.664 | 128 | 1.09 (0.32, 3.71) | 0.897 | 35 | 0.76 (0.2, 2.95) | 0.71 | 810 | 0.99 (0.5, 1.94) | 0.97 |
| 228 | Hemangioma and lymphangioma, any site | 2416 | 17605340 | 1050 | 1.04 (0.68, 1.61) | 0.864 | 295 | 1.05 (0.52, 2.11) | 0.902 | 103 | 0.95 (0.27, 3.32) | 0.938 | 2065 | 1.06 (0.65, 1.74) | 0.814 |
| 229 | Benign neoplasm of unspecified sites | 2115 | 17606297 | 914 | 1.03 (0.59, 1.78) | 0.935 | 257 | 1.07 (0.55, 2.08) | 0.849 | 93 | 0.99 (0.57, 1.71) | 0.97 | 1794 | 1.03 (0.43, 2.44) | 0.958 |
| 860 | Bone marrow or stem cell transplant | 142 | 17631302 | 61 | 0.84 (0.41, 1.73) | 0.656 | 11 | 0.55 (0.26, 1.18) | 0.128 | 1 | 0.13 (0.02, 1.13) | 0.064 | 136 | **4.15 (2.12, 8.11)** | **<0.001** |
| **Endocrine/Metabolic** | | | | | | | | | | | | | | | |
| 240 | Simple and unspecified goiter | 4078 | 17587886 | 1719 | 0.96 (0.77, 1.19) | 0.717 | 459 | 0.95 (0.59, 1.55) | 0.859 | 176 | 0.93 (0.48, 1.81) | 0.837 | 3441 | 1 (0.8, 1.24) | 0.97 |
| 241 | Nontoxic nodular goiter | 8992 | 17519286 | 3884 | 0.99 (0.77, 1.27) | 0.935 | 968 | 0.94 (0.79, 1.12) | 0.501 | 379 | 0.92 (0.68, 1.25) | 0.622 | 7574 | 0.99 (0.78, 1.27) | 0.97 |
| 241.1 | Nontoxic uninodular goiter | 4232 | 17593407 | 1854 | 1.03 (0.76, 1.39) | 0.866 | 480 | 0.99 (0.73, 1.35) | 0.97 | 166 | 0.87 (0.59, 1.3) | 0.507 | 3577 | 1.01 (0.72, 1.42) | 0.97 |
| 241.2 | Nontoxic multinodular goiter | 6011 | 17546654 | 2575 | 0.97 (0.79, 1.21) | 0.817 | 650 | 0.95 (0.72, 1.25) | 0.724 | 257 | 0.94 (0.58, 1.52) | 0.8 | 5083 | 1.03 (0.74, 1.42) | 0.885 |
| 242 | Thyrotoxicosis with or without goiter | 9744 | 17527426 | 4025 | **0.91 (0.85, 0.98)** | **0.01** | 1100 | 0.94 (0.76, 1.16) | 0.576 | 397 | 0.86 (0.69, 1.06) | 0.156 | 8244 | 1.02 (0.72, 1.43) | 0.92 |
| 242.1 | Graves' disease | 4419 | 17577734 | 1827 | 0.93 (0.82, 1.06) | 0.3 | 524 | 0.99 (0.71, 1.39) | 0.97 | 185 | 0.89 (0.58, 1.38) | 0.619 | 3745 | 1.02 (0.65, 1.61) | 0.929 |
| 242.2 | Toxic multinodular goiter | 2538 | 17604505 | 1041 | 0.87 (0.75, 1) | 0.054 | 250 | 0.83 (0.62, 1.1) | 0.186 | 103 | 0.84 (0.44, 1.58) | 0.597 | 2132 | 1 (0.84, 1.18) | 0.97 |
| 242.3 | Exophthalmos | 440 | 17627786 | 178 | 0.85 (0.46, 1.56) | 0.607 | 45 | 0.8 (0.26, 2.42) | 0.7 | 15 | 0.67 (0.12, 3.83) | 0.668 | 385 | 1.29 (0.58, 2.86) | 0.542 |
| 244 | Hypothyroidism | 14511 | 17538651 | 6085 | 0.97 (0.84, 1.12) | 0.671 | 1714 | 1 (0.92, 1.09) | 0.97 | 665 | 1 (0.83, 1.2) | 0.97 | 12223 | 0.98 (0.74, 1.29) | 0.885 |
| 244.1 | Secondary hypothyroidism | 1152 | 17623008 | 481 | 0.97 (0.3, 3.12) | 0.958 | 141 | 1.05 (0.19, 5.86) | 0.956 | 52 | 0.99 (0.59, 1.65) | 0.97 | 958 | 0.91 (0.34, 2.42) | 0.859 |
| 244.2 | Acquired hypothyroidism | 661 | 17628302 | 278 | 0.96 (0.16, 5.87) | 0.968 | 85 | 1.1 (0.14, 8.6) | 0.935 | 20 | 0.66 (0.08, 5.69) | 0.715 | 524 | 0.7 (0.43, 1.17) | 0.173 |
| 244.4 | Hypothyroidism NOS | 13472 | 17549153 | 5663 | 0.98 (0.81, 1.18) | 0.814 | 1604 | 1.01 (0.62, 1.65) | 0.962 | 618 | 1 (0.91, 1.1) | 0.97 | 11375 | 0.99 (0.7, 1.4) | 0.97 |
| 244.5 | Congenital hypothyroidism | 529 | 482914* | 218 | 0.96 (0.24, 3.9)** | 0.957 | 57 | 0.92 (0.14, 6)** | 0.934 | 35 | 1.44 (0.54, 3.85)** | 0.476 | 453 | 1.09 (0.28, 4.21)** | 0.904 |
| 245 | Thyroiditis | 2848 | 17610302 | 1199 | 1.01 (0.66, 1.55) | 0.97 | 351 | 1.03 (0.54, 1.99) | 0.931 | 145 | 1.12 (0.65, 1.91) | 0.7 | 2391 | 0.93 (0.69, 1.27) | 0.68 |
| 245.1 | Thyroiditis, acute and subacute | 696 | 17624321 | 289 | 1 (0.87, 1.14) | 0.97 | 90 | 1.13 (0.48, 2.65) | 0.792 | 38 | 1.22 (0.43, 3.45) | 0.715 | 574 | 0.85 (0.53, 1.38) | 0.534 |
| 245.21 | Chronic lymphocytic thyroiditis | 1959 | 17620045 | 830 | 1.02 (0.49, 2.09) | 0.97 | 239 | 1 (0.94, 1.06) | 0.97 | 93 | 1.03 (0.26, 4.02) | 0.97 | 1648 | 0.94 (0.59, 1.48) | 0.796 |
| 246 | Other disorders of thyroid | 595 | 17625153 | 257 | 1 (0.87, 1.15) | 0.97 | 64 | 0.93 (0.15, 5.88) | 0.946 | 28 | 1.04 (0.2, 5.4) | 0.97 | 507 | 1.06 (0.27, 4.2) | 0.935 |
| 249 | Secondary diabetes mellitus | 159 | 17630770 | 72 | 1.06 (0.14, 8.18) | 0.959 | 14 | 0.81 (0.06, 10.76) | 0.883 | 9 | 1.3 (0.05, 31) | 0.881 | 136 | 1.12 (0.12, 10.57) | 0.931 |
| 250 | Diabetes mellitus | 36810 | 17295033 | 16107 | **1.04 (1, 1.08)** | **0.033** | 4346 | **1.11 (1.07, 1.16)** | **<0.001** | 1716 | **1.09 (1.01, 1.18)** | **0.03** | 31265 | **1.07 (1.03, 1.11)** | **0.001** |
| 250.1 | Type 1 diabetes | 12504 | 17513735 | 5465 | 1.04 (0.94, 1.14) | 0.478 | 1469 | 1.09 (0.99, 1.2) | 0.096 | 577 | 1.07 (0.83, 1.37) | 0.629 | 10621 | 1.06 (0.96, 1.17) | 0.267 |
| 250.11 | Type 1 diabetes with ketoacidosis | 994 | 17623282 | 394 | 0.9 (0.62, 1.29) | 0.575 | 131 | 1.07 (0.41, 2.8) | 0.896 | 49 | 1.04 (0.18, 6.02) | 0.97 | 840 | 0.98 (0.41, 2.33) | 0.97 |
| 250.12 | Type 1 diabetes with renal manifestations | 988 | 17622129 | 419 | 0.93 (0.63, 1.36) | 0.713 | 99 | 0.85 (0.52, 1.41) | 0.546 | 44 | 0.95 (0.15, 6.17) | 0.963 | 825 | 0.95 (0.46, 1.96) | 0.892 |
| 250.13 | Type 1 diabetes with ophthalmic manifestations | 1615 | 17608477 | 663 | 0.93 (0.68, 1.27) | 0.64 | 200 | 1.05 (0.45, 2.46) | 0.924 | 71 | 0.95 (0.2, 4.41) | 0.949 | 1351 | 0.94 (0.52, 1.69) | 0.849 |
| 250.14 | Type 1 diabetes with neurological manifestations | 1069 | 17618695 | 449 | 0.93 (0.54, 1.59) | 0.8 | 116 | 0.95 (0.28, 3.18) | 0.935 | 49 | 1 (0.8, 1.23) | 0.97 | 894 | 0.96 (0.33, 2.82) | 0.948 |
| 250.2 | Type 2 diabetes | 32505 | 17346533 | 14194 | 1.04 (0.99, 1.08) | 0.088 | 3869 | **1.13 (1.08, 1.18)** | **<0.001** | 1531 | **1.11 (1.02, 1.2)** | **0.018** | 27599 | **1.07 (1.02, 1.12)** | **0.004** |
| 250.21 | Type 2 diabetes with ketoacidosis | 358 | 17629921 | 146 | 0.88 (0.51, 1.52) | 0.661 | 42 | 0.98 (0.37, 2.62) | 0.97 | 13 | 0.76 (0.17, 3.39) | 0.733 | 306 | 1.1 (0.34, 3.55) | 0.883 |
| 250.22 | Type 2 diabetes with renal manifestations | 3390 | 17610456 | 1414 | 0.95 (0.76, 1.18) | 0.647 | 411 | 1.11 (0.85, 1.46) | 0.455 | 163 | 1.09 (0.56, 2.11) | 0.819 | 2878 | 1.07 (0.79, 1.44) | 0.68 |
| 250.23 | Type 2 diabetes with ophthalmic manifestations | 3453 | 17586121 | 1454 | 0.97 (0.71, 1.32) | 0.843 | 415 | 1.07 (0.76, 1.51) | 0.716 | 163 | 1.05 (0.48, 2.3) | 0.902 | 2930 | 1.05 (0.73, 1.51) | 0.818 |
| 250.24 | Type 2 diabetes with neurological manifestations | 4018 | 17593460 | 1738 | 0.98 (0.57, 1.7) | 0.958 | 446 | 1.02 (0.49, 2.1) | 0.97 | 176 | 0.99 (0.59, 1.67) | 0.97 | 3409 | 1.06 (0.76, 1.49) | 0.732 |
| 250.41 | Impaired fasting glucose | 349 | 17628739 | 145 | 0.93 (0.26, 3.35) | 0.921 | 39 | 0.98 (0.38, 2.5) | 0.97 | 18 | 1.13 (0.06, 22.32) | 0.941 | 290 | 0.92 (0.18, 4.74) | 0.929 |
| 250.42 | Other abnormal glucose | 1605 | 17625130 | 685 | 1 (0.8, 1.24) | 0.97 | 206 | 1.16 (0.87, 1.55) | 0.327 | 63 | 0.89 (0.36, 2.21) | 0.813 | 1354 | 1.01 (0.78, 1.3) | 0.97 |
| 250.6 | Polyneuropathy in diabetes | 516 | 17628958 | 233 | 1.14 (0.77, 1.69) | 0.527 | 59 | 1.15 (0.53, 2.48) | 0.733 | 32 | 1.55 (0.96, 2.51) | 0.074 | 433 | 0.99 (0.51, 1.9) | 0.97 |
| 250.7 | Diabetic retinopathy | 4762 | 17578672 | 2064 | 1.02 (0.76, 1.38) | 0.898 | 565 | 1.08 (0.87, 1.36) | 0.483 | 223 | 1.07 (0.65, 1.78) | 0.794 | 4037 | 1.04 (0.78, 1.4) | 0.8 |
| 251 | Other disorders of pancreatic internal secretion | 3669 | 17575435 | 1523 | 0.94 (0.78, 1.12) | 0.487 | 430 | 1.01 (0.75, 1.35) | 0.97 | 172 | 1.02 (0.47, 2.22) | 0.97 | 3105 | 1.02 (0.58, 1.81) | 0.949 |
| 251.1 | Hypoglycemia | 3589 | 17576119 | 1490 | 0.94 (0.78, 1.14) | 0.535 | 425 | 1.02 (0.46, 2.25) | 0.965 | 168 | 1.02 (0.44, 2.36) | 0.97 | 3036 | 1.02 (0.55, 1.88) | 0.959 |
| 252 | Disorders of parathyroid gland | 2643 | 17614108 | 1116 | 0.97 (0.59, 1.59) | 0.901 | 317 | 1.07 (0.55, 2.08) | 0.849 | 110 | 0.92 (0.29, 2.91) | 0.902 | 2227 | 1.01 (0.78, 1.29) | 0.97 |
| 252.1 | Hyperparathyroidism | 2137 | 17617708 | 890 | 0.95 (0.55, 1.62) | 0.859 | 265 | 1.11 (0.63, 1.96) | 0.734 | 89 | 0.92 (0.21, 4.04) | 0.923 | 1792 | 0.98 (0.33, 2.91) | 0.97 |
| 252.2 | Hypoparathyroidism | 513 | 17628184 | 229 | 1.04 (0.33, 3.23) | 0.956 | 53 | 0.91 (0.24, 3.52) | 0.902 | 20 | 0.87 (0.11, 6.84) | 0.901 | 438 | 1.09 (0.36, 3.25) | 0.891 |
| 253 | Disorders of the pituitary gland and its hypothalamic control | 4013 | 17601844 | 1642 | 0.95 (0.73, 1.24) | 0.727 | 522 | 1.06 (0.68, 1.66) | 0.806 | 185 | 0.98 (0.43, 2.24) | 0.97 | 3405 | 1 (0.99, 1.01) | 0.97 |
| 253.1 | Pituitary hyperfunction | 2127 | 17618116 | 832 | 0.89 (0.66, 1.2) | 0.462 | 280 | 1.03 (0.28, 3.75) | 0.968 | 108 | 1.05 (0.16, 6.84) | 0.965 | 1804 | 0.99 (0.61, 1.62) | 0.97 |
| 253.11 | Acromegaly and gigantism | 371 | 17626930 | 147 | 0.95 (0.24, 3.83) | 0.949 | 62 | 1.39 (0.77, 2.51) | 0.278 | 13 | 0.76 (0.07, 7.78) | 0.831 | 306 | 0.83 (0.37, 1.86) | 0.665 |
| 253.2 | Pituitary hypofunction | 1149 | 17622819 | 480 | 0.97 (0.38, 2.5) | 0.958 | 151 | 1.1 (0.45, 2.64) | 0.849 | 46 | 0.87 (0.19, 3.91) | 0.861 | 980 | 1.04 (0.37, 2.92) | 0.941 |
| 253.3 | Diabetes insipidus | 272 | 17629359 | 113 | 0.98 (0.33, 2.85) | 0.97 | 37 | 1.14 (0.21, 6.1) | 0.886 | 11 | 0.88 (0.02, 35.7) | 0.951 | 235 | 1.14 (0.23, 5.8) | 0.881 |
| 253.7 | Other disorders of neurohypophysis | 102 | 17631466 | 53 | 1.32 (0.56, 3.14) | 0.54 | 7 | 0.7 (0.07, 7.02) | 0.771 | 4 | 0.98 (0.48, 2.01) | 0.97 | 88 | 1.19 (0.13, 10.68) | 0.883 |
| 255 | Disorders of adrenal glands | 2468 | 17614814 | 1086 | 1.05 (0.63, 1.75) | 0.866 | 282 | 1.02 (0.32, 3.24) | 0.97 | 112 | 1.03 (0.24, 4.43) | 0.97 | 2093 | 1.03 (0.41, 2.6) | 0.953 |
| 255.1 | Adrenal hyperfunction | 100 | 17630753 | 41 | 1.05 (0.09, 11.97) | 0.97 | 13 | 1.14 (0.01, 91.57) | 0.959 | 8 | 1.85 (0.18, 18.93) | 0.617 | 87 | 1.21 (0.04, 32.78) | 0.919 |
| 255.11 | Cushing's syndrome | 308 | 17629259 | 134 | 1 (0.96, 1.04) | 0.97 | 35 | 0.95 (0.09, 10.11) | 0.97 | 10 | 0.7 (0.04, 11.89) | 0.814 | 259 | 0.96 (0.16, 5.88) | 0.97 |
| 255.12 | Hyperaldosteronism | 252 | 17629991 | 130 | 1.47 (0.92, 2.36) | 0.106 | 30 | 1.29 (0.31, 5.35) | 0.739 | 8 | 0.87 (0.01, 102.13) | 0.959 | 214 | 1.04 (0.14, 7.8) | 0.97 |
| 255.21 | Glucocorticoid deficiency | 974 | 17625756 | 409 | 0.96 (0.38, 2.43) | 0.934 | 115 | 1 (0.96, 1.05) | 0.97 | 42 | 0.93 (0.08, 10.86) | 0.959 | 826 | 1.02 (0.32, 3.24) | 0.97 |
| 255.3 | Adrenogenital disorders | 410 | 17628348 | 181 | 1.02 (0.4, 2.6) | 0.97 | 45 | 0.98 (0.37, 2.59) | 0.97 | 16 | 0.87 (0.05, 16.89) | 0.935 | 352 | 1.14 (0.3, 4.27) | 0.861 |
| 256 | Ovarian dysfunction | 3687 | 10483211 | 1541 | 1.03 (0.63, 1.68) | 0.924 | 506 | 1.08 (0.71, 1.65) | 0.723 | 164 | 0.96 (0.27, 3.38) | 0.949 | 3166 | 1.05 (0.59, 1.85) | 0.885 |
| 256.4 | Polycystic ovaries | 2907 | 10489473 | 1218 | 1.04 (0.82, 1.31) | 0.789 | 400 | 1.09 (0.88, 1.35) | 0.455 | 132 | 0.98 (0.35, 2.76) | 0.97 | 2507 | 1.08 (0.88, 1.32) | 0.487 |
| 257 | Testicular dysfunction | 681 | 7115394 | 297 | 0.99 (0.75, 1.32) | 0.97 | 75 | 0.94 (0.07, 13.57) | 0.967 | 28 | 0.9 (0.02, 38.42) | 0.958 | 579 | 1.04 (0.16, 6.97) | 0.97 |
| 257.1 | Testicular hypofunction | 623 | 7116207 | 277 | 1.02 (0.38, 2.77) | 0.97 | 67 | 0.93 (0.27, 3.18) | 0.911 | 24 | 0.85 (0.14, 4.94) | 0.863 | 528 | 1.02 (0.41, 2.54) | 0.97 |
| 258 | Iatrogenic endocrine disorders | 734 | 17627625 | 326 | 1.07 (0.47, 2.44) | 0.873 | 94 | 1.12 (0.35, 3.62) | 0.859 | 25 | 0.77 (0.17, 3.55) | 0.752 | 631 | 1.11 (0.39, 3.17) | 0.859 |
| 259 | Other endocrine disorders | 3121 | 17598437 | 1288 | 0.96 (0.66, 1.38) | 0.819 | 373 | 0.95 (0.49, 1.87) | 0.902 | 157 | 1.05 (0.35, 3.15) | 0.931 | 2671 | 1.05 (0.61, 1.83) | 0.86 |
| 259.2 | Carcinoid syndrome | 416 | 17629580 | 159 | 0.8 (0.59, 1.07) | 0.133 | 43 | 0.85 (0.35, 2.01) | 0.717 | 25 | 1.22 (0.4, 3.72) | 0.734 | 343 | 0.88 (0.45, 1.73) | 0.727 |
| 259.3 | Delay in sexual development and puberty NEC | 281 | 17627619 | 111 | 0.84 (0, 3551.38) | 0.97 | 27 | 0.68 (0, 75628865.84) | 0.97 | 14 | 0.93 (0.03, 30.85) | 0.97 | 239 | 0.98 (0.36, 2.66) | 0.97 |
| 259.4 | Precocious sexual development and puberty NEC | 403 | 17624629 | 156 | 0.85 (0, 2319.64) | 0.97 | 47 | 0.82 (0, 12583.26) | 0.97 | 19 | 0.88 (0, 436.57) | 0.97 | 349 | 1.11 (0.01, 190.96) | 0.97 |
| 260 | Protein-calorie malnutrition | 4567 | 17620677 | 1982 | 1 (0.91, 1.11) | 0.97 | 503 | 1 (0.91, 1.1) | 0.97 | 209 | 1.03 (0.26, 4.18) | 0.965 | 3877 | 1.06 (0.67, 1.66) | 0.826 |
| 260.6 | Anorexia | 4359 | 17622526 | 1893 | 1 (0.93, 1.08) | 0.97 | 480 | 1 (0.86, 1.15) | 0.97 | 196 | 1.02 (0.49, 2.12) | 0.97 | 3698 | 1.05 (0.84, 1.31) | 0.668 |
| 261 | Vitamin deficiency | 6674 | 17594082 | 2787 | 0.96 (0.85, 1.09) | 0.531 | 848 | **1.12 (1.01, 1.25)** | **0.036** | 278 | 0.92 (0.65, 1.31) | 0.671 | 5676 | 1.06 (0.92, 1.23) | 0.428 |
| 261.1 | Vitamin A deficiency | 343 | 17630035 | 159 | 1.11 (0.49, 2.54) | 0.813 | 35 | 0.97 (0.27, 3.52) | 0.97 | 14 | 0.96 (0.17, 5.55) | 0.97 | 285 | 0.93 (0.22, 3.86) | 0.929 |
| 261.2 | Vitamin B-complex deficiencies | 1473 | 17624836 | 648 | 1 (0.86, 1.17) | 0.97 | 161 | 0.99 (0.56, 1.75) | 0.97 | 54 | 0.82 (0.34, 2.01) | 0.683 | 1220 | 0.91 (0.59, 1.42) | 0.7 |
| 261.4 | Vitamin D deficiency | 4105 | 17613013 | 1677 | 0.94 (0.85, 1.04) | 0.234 | 557 | **1.2 (1.09, 1.31)** | **<0.001** | 175 | 0.95 (0.55, 1.63) | 0.853 | 3527 | **1.14 (1.04, 1.25)** | **0.006** |
| 261.41 | Rickets or osteomalacia | 749 | 17622472 | 281 | 0.81 (0.62, 1.05) | 0.114 | 105 | 1.1 (0.41, 2.93) | 0.861 | 30 | 0.81 (0.23, 2.91) | 0.758 | 627 | 0.93 (0.37, 2.32) | 0.888 |
| 262 | Mineral deficiency NEC | 533 | 17629768 | 253 | 1.18 (0.79, 1.75) | 0.435 | 50 | 0.86 (0.29, 2.62) | 0.809 | 24 | 1.06 (0.07, 15.76) | 0.97 | 463 | 1.22 (0.68, 2.18) | 0.512 |
| 263 | Other nutritional deficiency | 1992 | 17623843 | 852 | 0.96 (0.69, 1.33) | 0.8 | 199 | 0.88 (0.63, 1.22) | 0.443 | 98 | 1.08 (0.43, 2.72) | 0.883 | 1706 | 1.13 (0.89, 1.43) | 0.326 |
| 264 | Lack of normal physiological development | 2944 | 17597896 | 1242 | 1.03 (0.29, 3.58) | 0.97 | 395 | 1.05 (0.21, 5.35) | 0.959 | 133 | 0.96 (0.14, 6.56) | 0.97 | 2522 | 1.04 (0.18, 5.82) | 0.97 |
| 264.3 | Delayed milestones | 1313 | 17615243 | 537 | 0.98 (0.34, 2.83) | 0.97 | 184 | 1.08 (0.11, 10.73) | 0.953 | 61 | 0.97 (0.23, 4.08) | 0.97 | 1105 | 0.91 (0.17, 4.76) | 0.92 |
| 264.9 | Lack of normal physiological development, unspecified | 1957 | 17611759 | 855 | 1.09 (0.28, 4.21) | 0.91 | 251 | 1.02 (0.4, 2.62) | 0.97 | 86 | 0.96 (0.11, 8.21) | 0.97 | 1693 | 1.12 (0.19, 6.64) | 0.906 |
| 269 | Proteinuria | 901 | 17624493 | 396 | 1.04 (0.56, 1.96) | 0.904 | 111 | 1.09 (0.46, 2.6) | 0.856 | 33 | 0.83 (0.29, 2.37) | 0.736 | 774 | 1.12 (0.66, 1.9) | 0.691 |
| 270 | Disorders of protein plasma/amino-acid transport and metabolism | 2102 | 17620047 | 922 | 1.02 (0.55, 1.92) | 0.946 | 225 | 0.98 (0.46, 2.12) | 0.97 | 103 | 1.12 (0.46, 2.73) | 0.813 | 1777 | 1.03 (0.46, 2.3) | 0.949 |
| 270.1 | Disturbances of amino-acid transport | 231 | 17630054 | 103 | 1.14 (0.14, 9.17) | 0.911 | 33 | 1.39 (0.17, 11.26) | 0.768 | 9 | 0.96 (0.12, 7.75) | 0.97 | 198 | 1.12 (0.03, 44.76) | 0.958 |
| 270.32 | Paraproteinemia | 1025 | 17627012 | 441 | 0.95 (0.51, 1.78) | 0.883 | 100 | 0.87 (0.44, 1.7) | 0.691 | 46 | 0.98 (0.44, 2.19) | 0.97 | 861 | 1 (0.89, 1.12) | 0.97 |
| 270.33 | Amyloidosis | 354 | 17630106 | 151 | 0.94 (0.2, 4.48) | 0.945 | 33 | 0.79 (0.16, 3.79) | 0.781 | 18 | 1.09 (0.02, 53.1) | 0.97 | 297 | 0.97 (0.23, 4.15) | 0.97 |
| 270.35 | Macroglobulinemia | 358 | 17630041 | 153 | 1.02 (0.4, 2.57) | 0.97 | 41 | 1.12 (0.37, 3.4) | 0.857 | 23 | 1.53 (0.92, 2.55) | 0.1 | 306 | 1.13 (0.54, 2.35) | 0.766 |
| 271 | Disorders of carbohydrate transport and metabolism | 1306 | 17619038 | 505 | 0.91 (0.64, 1.29) | 0.597 | 198 | 1.23 (0.9, 1.67) | 0.195 | 65 | 1.06 (0.21, 5.39) | 0.949 | 1133 | 1.16 (0.77, 1.74) | 0.487 |
| 271.3 | Intestinal disaccharidase deficiencies and disaccharide malabsorption | 1154 | 17621337 | 441 | 0.9 (0.64, 1.26) | 0.544 | 180 | 1.26 (0.94, 1.69) | 0.116 | 58 | 1.07 (0.24, 4.86) | 0.935 | 1000 | 1.15 (0.72, 1.82) | 0.575 |
| 271.9 | Other disorders of carbohydrate transport and metabolism | 159 | 17629327 | 65 | 0.91 (0.1, 7.84) | 0.935 | 18 | 0.88 (0.02, 32.1) | 0.949 | 7 | 0.89 (0, 194.26) | 0.97 | 138 | 1.18 (0.09, 15.43) | 0.909 |
| 272 | Disorders of lipoid metabolism | 41222 | 17347032 | 18531 | **1.08 (1.03, 1.12)** | **<0.001** | 4502 | 1.05 (0.93, 1.18) | 0.443 | 1858 | 1.07 (0.9, 1.27) | 0.459 | 34767 | 1.02 (0.9, 1.17) | 0.733 |
| 272.1 | Hyperlipidemia | 8676 | 17571684 | 3876 | 1.07 (0.83, 1.38) | 0.626 | 990 | 1.09 (0.71, 1.7) | 0.7 | 395 | 1.08 (0.41, 2.85) | 0.885 | 7308 | 1.01 (0.56, 1.83) | 0.97 |
| 272.11 | Hypercholesterolemia | 35565 | 17395012 | 15991 | **1.08 (1.05, 1.1)** | **<0.001** | 3885 | 1.05 (0.99, 1.11) | 0.107 | 1609 | 1.07 (0.99, 1.17) | 0.088 | 30005 | 1.03 (0.96, 1.1) | 0.455 |
| 272.12 | Hyperglyceridemia | 291 | 17627372 | 128 | 1.03 (0.27, 3.88) | 0.97 | 33 | 1.05 (0.1, 10.63) | 0.97 | 13 | 1.03 (0.25, 4.17) | 0.97 | 256 | 1.38 (0.72, 2.63) | 0.332 |
| 272.13 | Mixed hyperlipidemia | 1324 | 17619911 | 593 | 1.12 (0.81, 1.55) | 0.512 | 151 | 1.13 (0.59, 2.16) | 0.725 | 80 | **1.49 (1, 2.21)** | **0.048** | 1128 | 1.09 (0.56, 2.1) | 0.818 |
| 272.9 | Unspecified disorder of lipoid metabolism | 949 | 17625215 | 432 | 1.1 (0.82, 1.47) | 0.541 | 102 | 1.03 (0.32, 3.37) | 0.959 | 45 | 1.13 (0.41, 3.1) | 0.82 | 800 | 1.02 (0.48, 2.15) | 0.97 |
| 274 | Gout and other crystal arthropathies | 8028 | 17568915 | 3477 | 0.99 (0.75, 1.31) | 0.94 | 904 | 1.02 (0.63, 1.64) | 0.949 | 342 | 0.96 (0.56, 1.65) | 0.885 | 6704 | 0.95 (0.81, 1.13) | 0.597 |
| 274.1 | Gout | 4013 | 17604934 | 1704 | 0.95 (0.76, 1.19) | 0.675 | 457 | 1.03 (0.52, 2.03) | 0.946 | 176 | 0.98 (0.32, 2.95) | 0.97 | 3338 | 0.94 (0.73, 1.21) | 0.628 |
| 274.11 | Gouty arthropathy | 4013 | 17599861 | 1749 | 1.01 (0.76, 1.34) | 0.97 | 448 | 1.03 (0.64, 1.68) | 0.903 | 188 | 1.08 (0.61, 1.9) | 0.813 | 3357 | 0.97 (0.66, 1.42) | 0.884 |
| 274.2 | Crystal arthropathies | 262 | 17630308 | 124 | 1.04 (0.14, 7.57) | 0.97 | 23 | 0.78 (0.14, 4.37) | 0.79 | 3 | 0.25 (0.03, 2.37) | 0.229 | 212 | 0.81 (0.3, 2.16) | 0.683 |
| 274.21 | Chondrocalcinosis | 1062 | 17621980 | 451 | 0.97 (0.47, 1.99) | 0.932 | 124 | 0.96 (0.26, 3.53) | 0.959 | 40 | 0.8 (0.3, 2.13) | 0.67 | 891 | 0.95 (0.42, 2.11) | 0.901 |
| 275 | Disorders of mineral metabolism | 3083 | 17619767 | 1266 | 0.9 (0.81, 1) | 0.053 | 358 | 1 (0.91, 1.09) | 0.97 | 127 | 0.88 (0.56, 1.4) | 0.611 | 2597 | 1 (0.85, 1.18) | 0.97 |
| 275.3 | Disorders of magnesium metabolism | 798 | 17629835 | 324 | 0.89 (0.62, 1.26) | 0.512 | 96 | 1.04 (0.25, 4.33) | 0.958 | 32 | 0.86 (0.18, 4.04) | 0.861 | 678 | 1.07 (0.43, 2.62) | 0.897 |
| 275.5 | Disorders of calcium/phosphorus metabolism | 1656 | 17626491 | 684 | 0.91 (0.76, 1.1) | 0.347 | 195 | 1.02 (0.43, 2.47) | 0.961 | 66 | 0.86 (0.45, 1.65) | 0.668 | 1391 | 0.99 (0.56, 1.73) | 0.97 |
| 275.53 | Disorders of phosphorus metabolism | 114 | 17631130 | 44 | 0.78 (0.31, 1.94) | 0.599 | 12 | 0.8 (0.08, 8.44) | 0.861 | 4 | 0.68 (0.02, 26.07) | 0.845 | 98 | 1.13 (0.11, 11.98) | 0.924 |
| 276 | Disorders of fluid, electrolyte, and acid-base balance | 15761 | 17561667 | 6911 | 1.02 (0.88, 1.19) | 0.807 | 1789 | 1.06 (0.91, 1.22) | 0.48 | 655 | 0.95 (0.66, 1.37) | 0.813 | 13227 | 0.99 (0.73, 1.35) | 0.959 |
| 276.1 | Electrolyte imbalance | 697 | 17630041 | 308 | 1.04 (0.38, 2.85) | 0.949 | 76 | 1.02 (0.39, 2.66) | 0.97 | 32 | 1.06 (0.08, 14.99) | 0.967 | 586 | 1 (0.96, 1.04) | 0.97 |
| 276.11 | Hyperosmolality and/or hypernatremia | 507 | 17630488 | 219 | 1.03 (0.39, 2.77) | 0.953 | 67 | 1.26 (0.8, 1.99) | 0.327 | 21 | 0.98 (0.36, 2.63) | 0.97 | 437 | 1.18 (0.71, 1.98) | 0.534 |
| 276.12 | Hyposmolality and/or hyponatremia | 6043 | 17609006 | 2652 | 1.01 (0.79, 1.31) | 0.92 | 669 | 1.03 (0.73, 1.46) | 0.86 | 250 | 0.95 (0.56, 1.61) | 0.864 | 5073 | 1 (0.92, 1.08) | 0.97 |
| 276.13 | Hyperpotassemia | 2659 | 17624460 | 1157 | 1.03 (0.74, 1.42) | 0.882 | 306 | 1.09 (0.86, 1.4) | 0.483 | 135 | 1.19 (0.92, 1.54) | 0.184 | 2222 | 0.97 (0.65, 1.44) | 0.882 |
| 276.14 | Hypopotassemia | 6139 | 17612482 | 2674 | 1.02 (0.82, 1.27) | 0.883 | 739 | **1.11 (1, 1.23)** | **0.04** | 235 | 0.88 (0.72, 1.08) | 0.212 | 5167 | 1 (0.86, 1.18) | 0.97 |
| 276.41 | Acidosis | 1608 | 17611660 | 735 | 1.07 (0.68, 1.69) | 0.773 | 169 | 0.97 (0.23, 4.11) | 0.97 | 57 | 0.82 (0.28, 2.35) | 0.719 | 1336 | 0.92 (0.5, 1.72) | 0.813 |
| 276.42 | Alkalosis | 227 | 17629385 | 99 | 0.96 (0.12, 7.92) | 0.97 | 20 | 0.76 (0.12, 4.89) | 0.784 | 10 | 0.94 (0.05, 16.6) | 0.97 | 187 | 0.88 (0.15, 5.32) | 0.898 |
| 276.6 | Fluid overload | 310 | 17631050 | 144 | 1.03 (0.44, 2.4) | 0.949 | 26 | 0.74 (0.47, 1.17) | 0.198 | 8 | 0.57 (0.28, 1.14) | 0.109 | 265 | 1.12 (0.61, 2.05) | 0.738 |
| 276.8 | Polydipsia | 142 | 17630402 | 55 | 0.89 (0.17, 4.65) | 0.901 | 21 | 1.26 (0.2, 7.76) | 0.817 | 7 | 1.07 (0.04, 25.74) | 0.97 | 117 | 0.85 (0.12, 6.07) | 0.883 |
| 277 | Other disorders of metabolism | 2393 | 17609693 | 1062 | 1.05 (0.74, 1.48) | 0.805 | 268 | 0.98 (0.39, 2.45) | 0.97 | 95 | 0.89 (0.41, 1.91) | 0.773 | 2025 | 1.01 (0.64, 1.6) | 0.97 |
| 277.1 | Disorders of porphyrin metabolism | 277 | 17628306 | 116 | 0.91 (0.28, 2.98) | 0.885 | 27 | 0.81 (0.16, 4.15) | 0.815 | 13 | 0.98 (0.47, 2.07) | 0.97 | 238 | 1.14 (0.23, 5.63) | 0.883 |
| 277.4 | Disorders of bilirubin excretion | 367 | 17627547 | 149 | 0.93 (0.28, 3.09) | 0.909 | 51 | 1.13 (0.2, 6.24) | 0.898 | 13 | 0.75 (0.06, 9.66) | 0.835 | 301 | 0.81 (0.35, 1.9) | 0.647 |
| 277.5 | Other disorders of lipoid metabolism | 561 | 17627386 | 249 | 1.06 (0.43, 2.65) | 0.901 | 67 | 1.06 (0.16, 6.84) | 0.955 | 22 | 0.89 (0.07, 11.29) | 0.935 | 473 | 0.99 (0.49, 2) | 0.97 |
| 277.51 | Lipoprotein disorders | 223 | 17630366 | 103 | 1.17 (0.49, 2.78) | 0.74 | 25 | 1.02 (0.49, 2.1) | 0.97 | 10 | 1.05 (0.1, 11.56) | 0.97 | 191 | 1.08 (0.13, 9.27) | 0.949 |
| 278 | Overweight, obesity and other hyperalimentation | 43021 | 17287273 | 18336 | 1.01 (0.88, 1.16) | 0.901 | 5153 | 1 (0.85, 1.18) | 0.97 | 1972 | 1 (0.91, 1.11) | 0.97 | 36352 | 0.98 (0.86, 1.11) | 0.765 |
| 278.1 | Obesity | 42869 | 17290924 | 18271 | 1.01 (0.88, 1.16) | 0.901 | 5135 | 1 (0.85, 1.18) | 0.97 | 1965 | 1 (0.91, 1.11) | 0.97 | 36218 | 0.98 (0.87, 1.1) | 0.724 |
| 279 | Disorders involving the immune mechanism | 1360 | 17621820 | 581 | 1.02 (0.53, 1.98) | 0.947 | 167 | 1.03 (0.38, 2.83) | 0.952 | 67 | 1.09 (0.34, 3.42) | 0.897 | 1132 | 0.89 (0.65, 1.21) | 0.462 |
| 279.1 | Immunity deficiency | 704 | 17626882 | 287 | 0.95 (0.44, 2.06) | 0.901 | 96 | 1.11 (0.41, 2.98) | 0.849 | 30 | 0.91 (0.11, 7.22) | 0.934 | 579 | 0.83 (0.55, 1.24) | 0.364 |
| 279.11 | Deficiency of humoral immunity | 517 | 17627443 | 231 | 1.09 (0.57, 2.09) | 0.808 | 53 | 0.88 (0.3, 2.55) | 0.818 | 29 | 1.25 (0.42, 3.74) | 0.7 | 435 | 0.95 (0.29, 3.09) | 0.935 |
| 279.7 | Other immunological findings | 163 | 17630676 | 74 | 1.28 (0.81, 2.01) | 0.3 | 24 | 1.45 (0.82, 2.56) | 0.198 | 9 | 1.43 (0.42, 4.87) | 0.578 | 137 | 0.94 (0.18, 4.97) | 0.949 |
| 279.8 | Other specified disorders involving the immune mechanism | 103 | 17631220 | 48 | 1.05 (0.12, 9.1) | 0.97 | 9 | 0.67 (0.14, 3.2) | 0.626 | 2 | 0.39 (0.03, 5.97) | 0.512 | 89 | 1.13 (0.11, 11.42) | 0.924 |
| **Hematopoietic** | | | | | | | | | | | | | | | |
| 275.1 | Disorders of iron metabolism | 473 | 17628447 | 193 | 0.86 (0.62, 1.19) | 0.36 | 50 | 0.85 (0.4, 1.8) | 0.691 | 18 | 0.78 (0.25, 2.45) | 0.68 | 403 | 1.07 (0.41, 2.81) | 0.901 |
| 280 | Iron deficiency anemias | 8958 | 17567318 | 3875 | 1.02 (0.67, 1.55) | 0.935 | 1075 | 1.08 (0.75, 1.54) | 0.7 | 378 | 0.95 (0.36, 2.51) | 0.929 | 7567 | 1.01 (0.56, 1.82) | 0.97 |
| 280.1 | Iron deficiency anemias, unspecified or not due to blood loss | 7666 | 17573823 | 3296 | 1.01 (0.52, 1.96) | 0.97 | 942 | 1.1 (0.72, 1.68) | 0.685 | 321 | 0.94 (0.29, 3.07) | 0.93 | 6469 | 1 (0.91, 1.1) | 0.97 |
| 280.2 | Iron deficiency anemia secondary to blood loss (chronic) | 1890 | 17622849 | 832 | 1.02 (0.6, 1.71) | 0.959 | 196 | 0.95 (0.52, 1.73) | 0.87 | 86 | 1.03 (0.26, 4.01) | 0.97 | 1600 | 1.05 (0.65, 1.68) | 0.864 |
| 281 | Other deficiency anemia | 2145 | 17616869 | 899 | 0.93 (0.72, 1.21) | 0.607 | 234 | 0.94 (0.46, 1.92) | 0.879 | 97 | 0.98 (0.34, 2.84) | 0.97 | 1797 | 0.97 (0.51, 1.85) | 0.935 |
| 281.1 | Megaloblastic anemia | 163 | 17630967 | 69 | 0.96 (0.14, 6.73) | 0.968 | 17 | 0.94 (0.06, 14.31) | 0.97 | 9 | 1.23 (0.09, 16.85) | 0.885 | 135 | 0.92 (0.15, 5.49) | 0.931 |
| 281.11 | Pernicious anemia | 552 | 17625811 | 237 | 0.94 (0.41, 2.11) | 0.883 | 53 | 0.81 (0.37, 1.81) | 0.624 | 22 | 0.84 (0.12, 6.15) | 0.877 | 458 | 0.92 (0.34, 2.46) | 0.873 |
| 281.12 | Other vitamin B12 deficiency anemia | 1049 | 17625203 | 421 | 0.87 (0.66, 1.13) | 0.3 | 122 | 0.97 (0.24, 3.87) | 0.97 | 45 | 0.9 (0.2, 4.04) | 0.898 | 880 | 0.98 (0.33, 2.91) | 0.97 |
| 281.13 | Folate-deficiency anemia | 337 | 17630022 | 150 | 1.1 (0.34, 3.61) | 0.883 | 45 | 1.29 (0.48, 3.51) | 0.625 | 13 | 0.93 (0.03, 25.26) | 0.97 | 286 | 1.06 (0.14, 8.22) | 0.962 |
| 281.9 | Deficiency anemias | 184 | 17630557 | 80 | 1.06 (0.09, 11.79) | 0.968 | 24 | 1.2 (0.12, 12.13) | 0.883 | 8 | 1.02 (0.49, 2.08) | 0.97 | 158 | 1.13 (0.11, 11.32) | 0.923 |
| 282 | Hereditary hemolytic anemias | 947 | 482914* | 338 | 0.83 (0.51, 1.37)** | 0.48 | 154 | 1.28 (0.7, 2.35)** | 0.432 | 62 | 1.37 (0.53, 3.57)** | 0.531 | 856 | **1.65 (1.06, 2.56)**** | **0.026** |
| 282.8 | Other hemoglobinopathies | 557 | 482914* | 177 | **0.72 (0.54, 0.96)**** | **0.027** | 103 | 1.39 (0.94, 2.04)** | 0.096 | 38 | 1.37 (0.59, 3.2)** | 0.478 | 517 | **2.24 (1.52, 3.3)**** | **<0.001** |
| 282.9 | Other hereditary hemolytic anemias | 350 | 482914* | 151 | 1.09 (0.23, 5.25)** | 0.92 | 43 | 1.08 (0.05, 24.44)** | 0.965 | 23 | 1.52 (0.3, 7.62)** | 0.626 | 303 | 1.15 (0.16, 8.2)** | 0.897 |
| 283 | Acquired hemolytic anemias | 645 | 17627970 | 276 | 1.02 (0.36, 2.88) | 0.97 | 77 | 1.08 (0.14, 8.55) | 0.949 | 35 | 1.24 (0.16, 9.91) | 0.849 | 537 | 0.92 (0.23, 3.74) | 0.914 |
| 283.1 | Autoimmune hemolytic anemias | 333 | 17630182 | 147 | 1.05 (0.22, 4.9) | 0.956 | 36 | 0.99 (0.59, 1.65) | 0.97 | 17 | 1.17 (0.1, 13.83) | 0.906 | 277 | 0.92 (0.2, 4.27) | 0.924 |
| 283.2 | Non-autoimmune hemolytic anemias | 103 | 17631312 | 43 | 1.14 (0.18, 7.18) | 0.901 | 17 | 1.71 (0.62, 4.7) | 0.3 | 7 | 1.78 (0.3, 10.61) | 0.539 | 83 | 0.77 (0.2, 2.88) | 0.708 |
| 284 | Aplastic anemia | 1103 | 17627177 | 498 | 1.08 (0.7, 1.67) | 0.73 | 118 | 0.99 (0.73, 1.34) | 0.97 | 52 | 1.1 (0.25, 4.83) | 0.909 | 930 | 1.01 (0.77, 1.31) | 0.97 |
| 285 | Other anemias | 24892 | 17520419 | 10894 | 1.01 (0.87, 1.18) | 0.897 | 2717 | 1 (0.85, 1.18) | 0.97 | 1094 | 1 (0.9, 1.11) | 0.97 | 20943 | 1.01 (0.78, 1.31) | 0.958 |
| 285.1 | Acute posthemorrhagic anemia | 187 | 17626491 | 78 | 1.03 (0.24, 4.37) | 0.97 | 28 | 1.38 (0, 6351499.99) | 0.97 | 8 | 0.96 (0.17, 5.43) | 0.97 | 157 | 0.96 (0.14, 6.41) | 0.97 |
| 285.2 | Anemia of chronic disease | 1276 | 17627846 | 580 | 1.07 (0.76, 1.51) | 0.722 | 131 | 0.97 (0.27, 3.49) | 0.97 | 54 | 0.99 (0.5, 1.93) | 0.97 | 1061 | 0.94 (0.52, 1.72) | 0.858 |
| 285.22 | Anemia in neoplastic disease | 3640 | 17626574 | 1595 | 1 (0.88, 1.13) | 0.97 | 374 | 0.94 (0.6, 1.48) | 0.814 | 165 | 1.02 (0.34, 3.07) | 0.97 | 2992 | 0.88 (0.77, 1) | 0.055 |
| 285.3 | Sideroblastic anemia | 285 | 17629827 | 108 | 0.75 (0.43, 1.29) | 0.3 | 32 | 0.86 (0.11, 6.66) | 0.897 | 8 | 0.54 (0.08, 3.8) | 0.546 | 246 | 1.19 (0.28, 5.05) | 0.827 |
| 286 | Coagulation defects | 4124 | 17606796 | 1882 | **1.18 (1.04, 1.33)** | **0.011** | 477 | 1.04 (0.5, 2.17) | 0.923 | 216 | 1.24 (0.85, 1.8) | 0.263 | 3461 | 0.93 (0.65, 1.34) | 0.723 |
| 286.11 | Von willebrand's disease | 214 | 482914* | 73 | **0.58 (0.35, 0.95)**** | **0.029** | 14 | 0.37 (0.14, 1.01)** | 0.051 | 4 | 0.28 (0.03, 3.09)** | 0.305 | 170 | 0.68 (0.28, 1.65)** | 0.405 |
| 286.12 | Congenital deficiency of other clotting factors (including factor VII) | 145 | 482914* | 64 | 1.22 (0.28, 5.26)** | 0.806 | 19 | 1.23 (0.09, 16.54)** | 0.885 | 11 | 1.89 (0.39, 9.1)** | 0.438 | 126 | 1.17 (0.1, 13.77)** | 0.908 |
| 286.3 | Coagulation defects complicating pregnancy or postpartum | 2015 | 10502418 | 902 | **1.2 (1.13, 1.28)** | **<0.001** | 267 | 1.14 (1, 1.29) | 0.054 | 108 | **1.27 (1.08, 1.49)** | **0.004** | 1687 | **0.88 (0.8, 0.97)** | **0.013** |
| 286.4 | Acquired coagulation factor deficiency | 470 | 17629631 | 223 | 1.11 (0.53, 2.31) | 0.8 | 37 | 0.74 (0.31, 1.75) | 0.498 | 20 | 0.98 (0.4, 2.43) | 0.97 | 394 | 0.99 (0.57, 1.7) | 0.97 |
| 286.5 | Hemorrhagic disorder due to intrinsic circulating anticoagulants | 503 | 17629245 | 239 | 1.22 (0.85, 1.77) | 0.287 | 54 | 1.12 (0.28, 4.39) | 0.885 | 26 | 1.32 (0.42, 4.14) | 0.645 | 417 | 0.92 (0.31, 2.79) | 0.898 |
| 286.7 | Other and unspecified coagulation defects | 1085 | 17621421 | 520 | **1.36 (1.16, 1.59)** | **<0.001** | 125 | 1.19 (0.74, 1.93) | 0.476 | 73 | **1.79 (1.34, 2.4)** | **<0.001** | 922 | 1.03 (0.34, 3.11) | 0.958 |
| 287 | Purpura and other hemorrhagic conditions | 5975 | 17575367 | 2508 | 0.95 (0.81, 1.1) | 0.5 | 653 | 0.88 (0.75, 1.04) | 0.146 | 275 | 0.96 (0.45, 2.05) | 0.93 | 5026 | 0.96 (0.74, 1.24) | 0.742 |
| 287.1 | Spontaneous ecchymoses | 282 | 17627926 | 121 | 0.97 (0.19, 5) | 0.97 | 32 | 0.87 (0.16, 4.65) | 0.883 | 8 | 0.58 (0.13, 2.64) | 0.487 | 229 | 0.76 (0.44, 1.31) | 0.33 |
| 287.2 | Allergic purpura | 1100 | 17611665 | 450 | 0.9 (0.67, 1.2) | 0.476 | 117 | 0.79 (0.57, 1.09) | 0.149 | 47 | 0.84 (0.35, 2.02) | 0.705 | 923 | 0.91 (0.56, 1.49) | 0.73 |
| 287.3 | Thrombocytopenia | 2012 | 17616765 | 861 | 0.95 (0.6, 1.51) | 0.85 | 199 | 0.82 (0.59, 1.16) | 0.27 | 92 | 0.97 (0.22, 4.29) | 0.97 | 1703 | 1.02 (0.48, 2.14) | 0.97 |
| 287.31 | Primary thrombocytopenia | 1137 | 17622942 | 488 | 0.99 (0.64, 1.53) | 0.97 | 122 | 0.9 (0.4, 2.02) | 0.807 | 56 | 1.06 (0.16, 7.28) | 0.955 | 966 | 1.03 (0.29, 3.64) | 0.97 |
| 287.32 | Secondary thrombocytopenia | 248 | 17630721 | 113 | 1.11 (0.29, 4.27) | 0.883 | 29 | 1.1 (0.08, 14.71) | 0.95 | 10 | 0.95 (0.1, 9.36) | 0.97 | 204 | 0.86 (0.2, 3.66) | 0.849 |
| 287.4 | Qualitative platelet defects | 110 | 17630504 | 41 | 0.73 (0.21, 2.53) | 0.635 | 11 | 0.69 (0.05, 8.93) | 0.791 | 4 | 0.66 (0, 121.65) | 0.883 | 99 | 1.62 (0.25, 10.63) | 0.629 |
| 288 | Diseases of white blood cells | 1789 | 17620380 | 722 | 0.92 (0.56, 1.49) | 0.734 | 233 | 1.11 (0.48, 2.59) | 0.815 | 83 | 1.01 (0.79, 1.28) | 0.97 | 1477 | 0.87 (0.53, 1.43) | 0.596 |
| 288.3 | Eosinophilia | 303 | 17627543 | 128 | 0.98 (0.32, 3.01) | 0.97 | 37 | 1.05 (0.1, 11.46) | 0.97 | 13 | 0.94 (0.05, 16.45) | 0.97 | 249 | 0.85 (0.24, 2.97) | 0.806 |
| 289 | Other diseases of blood and blood-forming organs | 10271 | 17502098 | 4447 | 1.03 (0.86, 1.23) | 0.797 | 1214 | 1.01 (0.7, 1.44) | 0.97 | 447 | 0.96 (0.56, 1.63) | 0.883 | 8688 | 0.99 (0.67, 1.46) | 0.97 |
| 289.4 | Lymphadenitis | 8566 | 17516797 | 3735 | 1.04 (0.9, 1.2) | 0.624 | 998 | 0.99 (0.59, 1.65) | 0.97 | 375 | 0.96 (0.54, 1.73) | 0.908 | 7241 | 0.98 (0.68, 1.41) | 0.934 |
| 289.5 | Diseases of spleen | 378 | 17628131 | 157 | 0.99 (0.62, 1.59) | 0.97 | 53 | 1.25 (0.37, 4.16) | 0.734 | 17 | 1.02 (0.44, 2.37) | 0.97 | 317 | 0.95 (0.09, 10.37) | 0.97 |
| 289.8 | Polycythemia, secondary | 856 | 17625499 | 355 | 0.91 (0.22, 3.78) | 0.906 | 103 | 1.01 (0.72, 1.41) | 0.97 | 28 | 0.69 (0.03, 14.73) | 0.825 | 736 | 1.14 (0.18, 7.29) | 0.901 |
| **Mental Disorders** | | | | | | | | | | | | | | | |
| 290 | Delirium dementia and amnestic and other cognitive disorders | 12977 | 17566840 | 5615 | 0.98 (0.78, 1.24) | 0.901 | 1440 | 1.02 (0.68, 1.53) | 0.935 | 549 | 0.95 (0.58, 1.57) | 0.864 | 10879 | 0.99 (0.64, 1.53) | 0.968 |
| 290.1 | Dementias | 1590 | 17627063 | 677 | 0.99 (0.57, 1.72) | 0.97 | 200 | 1.21 (0.84, 1.74) | 0.322 | 68 | 1 (0.88, 1.13) | 0.97 | 1330 | 0.99 (0.51, 1.91) | 0.97 |
| 290.11 | Alzheimer's disease | 5298 | 17609412 | 2261 | 0.96 (0.16, 5.7) | 0.97 | 590 | 1.04 (0.16, 6.6) | 0.97 | 238 | 1.02 (0.43, 2.42) | 0.97 | 4423 | 0.98 (0.3, 3.14) | 0.97 |
| 290.12 | Dementia with cerebral degenerations | 308 | 17630924 | 132 | 0.98 (0.37, 2.57) | 0.966 | 33 | 1.01 (0.79, 1.28) | 0.97 | 16 | 1.19 (0.46, 3.07) | 0.733 | 265 | 1.18 (0.77, 1.81) | 0.447 |
| 290.16 | Vascular dementia | 1451 | 17626887 | 642 | 1.03 (0.43, 2.46) | 0.955 | 167 | 1.1 (0.47, 2.58) | 0.835 | 55 | 0.88 (0.19, 4.16) | 0.885 | 1209 | 0.96 (0.36, 2.62) | 0.949 |
| 290.2 | Delirium due to conditions classified elsewhere | 4980 | 17613636 | 2165 | 0.99 (0.6, 1.63) | 0.967 | 553 | 1.01 (0.57, 1.79) | 0.97 | 203 | 0.92 (0.48, 1.75) | 0.812 | 4194 | 1.01 (0.55, 1.86) | 0.97 |
| 290.3 | Other persistent mental disorders due to conditions classified elsewhere | 1281 | 17612968 | 563 | 1.02 (0.47, 2.21) | 0.97 | 138 | 0.91 (0.35, 2.36) | 0.864 | 52 | 0.89 (0.19, 4.08) | 0.885 | 1081 | 0.99 (0.51, 1.9) | 0.97 |
| 291 | Other specified nonpsychotic and/or transient mental disorders | 1748 | 17618709 | 727 | 0.95 (0.62, 1.45) | 0.825 | 219 | 1.06 (0.51, 2.21) | 0.885 | 73 | 0.9 (0.28, 2.88) | 0.874 | 1487 | 1.04 (0.54, 2.02) | 0.907 |
| 291.1 | Transient mental disorders due to conditions classified elsewhere | 501 | 17627327 | 208 | 0.98 (0.41, 2.33) | 0.97 | 68 | 1.18 (0.34, 4.12) | 0.808 | 23 | 1.02 (0.32, 3.25) | 0.97 | 437 | 1.25 (0.51, 3.05) | 0.642 |
| 291.4 | Specific nonpsychotic mental disorders due to brain damage | 600 | 17626556 | 249 | 0.91 (0.56, 1.47) | 0.703 | 69 | 0.93 (0.29, 2.98) | 0.909 | 20 | 0.69 (0.26, 1.8) | 0.455 | 498 | 0.89 (0.48, 1.65) | 0.733 |
| 291.8 | Alteration of consciousness | 674 | 17628188 | 282 | 0.98 (0.37, 2.6) | 0.965 | 85 | 1.1 (0.46, 2.63) | 0.842 | 32 | 1.06 (0.12, 8.92) | 0.964 | 572 | 1.03 (0.3, 3.58) | 0.966 |
| 292 | Neurological disorders | 8633 | 17596001 | 3653 | 0.96 (0.58, 1.59) | 0.89 | 1019 | 1.08 (0.55, 2.11) | 0.842 | 379 | 0.99 (0.49, 1.97) | 0.97 | 7256 | 1 (0.92, 1.08) | 0.97 |
| 292.1 | Aphasia/speech disturbance | 1022 | 17625079 | 426 | 0.95 (0.42, 2.14) | 0.901 | 121 | 1.01 (0.53, 1.95) | 0.97 | 47 | 1 (0.96, 1.04) | 0.97 | 859 | 0.97 (0.25, 3.77) | 0.97 |
| 292.12 | Symbolic dysfunction | 151 | 17630108 | 68 | 1.23 (0.43, 3.57) | 0.712 | 25 | 1.57 (0.6, 4.08) | 0.362 | 5 | 0.82 (0, 157.72) | 0.947 | 118 | 0.63 (0.34, 1.17) | 0.14 |
| 292.2 | Mild cognitive impairment | 844 | 17628286 | 358 | 0.95 (0.47, 1.91) | 0.892 | 97 | 1.04 (0.2, 5.27) | 0.97 | 33 | 0.87 (0.17, 4.52) | 0.875 | 707 | 0.98 (0.42, 2.28) | 0.97 |
| 292.3 | Memory loss | 3241 | 17620397 | 1340 | 0.93 (0.34, 2.5) | 0.892 | 399 | 1.12 (0.27, 4.68) | 0.885 | 140 | 0.97 (0.19, 5.03) | 0.97 | 2759 | 1.09 (0.3, 3.95) | 0.905 |
| 292.4 | Altered mental status | 3282 | 17624168 | 1401 | 0.98 (0.7, 1.36) | 0.907 | 386 | 1.1 (0.86, 1.4) | 0.463 | 147 | 1.02 (0.33, 3.14) | 0.97 | 2743 | 0.97 (0.65, 1.46) | 0.901 |
| 292.6 | Hallucinations | 182 | 17630846 | 83 | 1.08 (0.03, 37.87) | 0.97 | 19 | 0.96 (0.13, 7.14) | 0.97 | 7 | 0.88 (0, 285.61) | 0.97 | 157 | 1.18 (0, 2674.8) | 0.97 |
| 293 | Symptoms involving head and neck | 5651 | 17571376 | 2459 | 1.02 (0.78, 1.33) | 0.908 | 650 | 1.01 (0.66, 1.55) | 0.97 | 253 | 1 (0.89, 1.13) | 0.97 | 4720 | 0.93 (0.81, 1.07) | 0.319 |
| 293.1 | Swelling, mass, or lump in head and neck [Space-occupying lesion, intracranial NOS] | 939 | 17625575 | 396 | 0.98 (0.4, 2.4) | 0.965 | 119 | 1.09 (0.46, 2.56) | 0.861 | 38 | 0.89 (0.2, 3.9) | 0.883 | 791 | 0.98 (0.33, 2.93) | 0.97 |
| 295 | Schizophrenia and other psychotic disorders | 3037 | 17602099 | 1267 | 0.94 (0.74, 1.18) | 0.59 | 337 | 0.92 (0.6, 1.43) | 0.736 | 143 | 1 (0.89, 1.12) | 0.97 | 2564 | 0.99 (0.77, 1.28) | 0.97 |
| 295.1 | Schizophrenia | 2220 | 17614618 | 927 | 0.94 (0.68, 1.28) | 0.7 | 255 | 0.95 (0.45, 2.01) | 0.898 | 95 | 0.91 (0.32, 2.59) | 0.864 | 1886 | 1.03 (0.47, 2.26) | 0.946 |
| 295.2 | Paranoid disorders | 739 | 17623210 | 326 | 1.03 (0.31, 3.37) | 0.965 | 76 | 0.91 (0.25, 3.41) | 0.902 | 36 | 1.09 (0.14, 8.77) | 0.938 | 619 | 0.96 (0.23, 4) | 0.958 |
| 295.3 | Psychosis | 367 | 17625680 | 137 | 0.78 (0.46, 1.33) | 0.371 | 39 | 0.85 (0.15, 4.71) | 0.864 | 25 | 1.38 (0.37, 5.14) | 0.645 | 307 | 0.95 (0.12, 7.68) | 0.965 |
| 296 | Mood disorders | 4033 | 17602612 | 1701 | 0.96 (0.74, 1.24) | 0.766 | 473 | 1.02 (0.49, 2.11) | 0.97 | 170 | 0.92 (0.46, 1.85) | 0.83 | 3395 | 0.99 (0.57, 1.71) | 0.97 |
| 296.1 | Bipolar | 1551 | 17621045 | 688 | 1.08 (0.75, 1.55) | 0.693 | 190 | 1.14 (0.7, 1.85) | 0.616 | 66 | 1 (0.85, 1.17) | 0.97 | 1294 | 0.94 (0.52, 1.69) | 0.836 |
| 296.22 | Major depressive disorder | 2405 | 17617215 | 1000 | 0.94 (0.8, 1.09) | 0.403 | 285 | 1.02 (0.53, 1.96) | 0.959 | 102 | 0.92 (0.5, 1.68) | 0.796 | 2034 | 1.02 (0.63, 1.66) | 0.939 |
| 297 | Suicidal ideation or attempt | 376 | 17629501 | 161 | 1.05 (0.38, 2.85) | 0.935 | 47 | 1.04 (0.17, 6.45) | 0.97 | 19 | 1.1 (0.13, 9.68) | 0.935 | 312 | 0.86 (0.44, 1.66) | 0.661 |
| 297.2 | Suicide or self-inflicted injury | 376 | 17629501 | 161 | 1.05 (0.38, 2.85) | 0.935 | 47 | 1.04 (0.17, 6.45) | 0.97 | 19 | 1.1 (0.13, 9.68) | 0.935 | 312 | 0.86 (0.44, 1.66) | 0.661 |
| 300 | Anxiety disorders | 12598 | 17547177 | 5464 | 1.01 (0.72, 1.42) | 0.967 | 1393 | 0.95 (0.76, 1.19) | 0.67 | 553 | 0.96 (0.57, 1.64) | 0.898 | 10524 | 0.93 (0.84, 1.03) | 0.173 |
| 300.1 | Anxiety disorder | 7985 | 17603188 | 3487 | 1.02 (0.89, 1.17) | 0.771 | 889 | 0.98 (0.74, 1.29) | 0.873 | 348 | 0.97 (0.62, 1.51) | 0.901 | 6658 | **0.92 (0.86, 0.99)** | **0.027** |
| 300.11 | Generalized anxiety disorder | 378 | 17629660 | 159 | 0.95 (0.4, 2.23) | 0.911 | 39 | 0.85 (0.34, 2.09) | 0.733 | 19 | 1.06 (0.07, 17.31) | 0.97 | 324 | 1.09 (0.36, 3.33) | 0.883 |
| 300.12 | Agorophobia, social phobia, and panic disorder | 882 | 17625483 | 396 | 1.07 (0.73, 1.58) | 0.728 | 93 | 0.9 (0.45, 1.83) | 0.788 | 39 | 0.98 (0.37, 2.6) | 0.97 | 723 | 0.82 (0.65, 1.04) | 0.096 |
| 300.13 | Phobia | 278 | 17628838 | 127 | 1.13 (0.42, 3.05) | 0.826 | 28 | 0.88 (0.12, 6.27) | 0.906 | 14 | 1.15 (0.06, 23.19) | 0.935 | 237 | 1.04 (0.15, 7.14) | 0.97 |
| 300.3 | Obsessive-compulsive disorders | 382 | 17629483 | 159 | 0.96 (0.25, 3.72) | 0.959 | 43 | 0.86 (0.22, 3.31) | 0.84 | 19 | 1.02 (0.4, 2.58) | 0.97 | 327 | 1.04 (0.18, 5.89) | 0.97 |
| 300.4 | Dysthymic disorder | 985 | 17613790 | 406 | 0.88 (0.57, 1.36) | 0.59 | 100 | 0.86 (0.38, 1.95) | 0.725 | 43 | 0.91 (0.12, 6.7) | 0.935 | 806 | 0.85 (0.53, 1.36) | 0.51 |
| 300.8 | Acute reaction to stress | 1073 | 17622478 | 486 | 1.11 (0.84, 1.47) | 0.477 | 116 | 0.93 (0.41, 2.11) | 0.864 | 48 | 1 (0.98, 1.02) | 0.97 | 908 | 0.98 (0.46, 2.09) | 0.97 |
| 300.9 | Posttraumatic stress disorder | 605 | 17628598 | 234 | 0.88 (0.59, 1.3) | 0.52 | 85 | 1.12 (0.48, 2.58) | 0.806 | 29 | 1 (0.96, 1.04) | 0.97 | 515 | 1.02 (0.38, 2.72) | 0.97 |
| 301 | Personality disorders | 1696 | 17610666 | 750 | 1.04 (0.58, 1.86) | 0.906 | 177 | 0.87 (0.51, 1.46) | 0.599 | 73 | 0.93 (0.2, 4.25) | 0.929 | 1413 | 0.9 (0.59, 1.36) | 0.619 |
| 301.2 | Antisocial/borderline personality disorder | 708 | 17627745 | 311 | 1.05 (0.25, 4.5) | 0.949 | 79 | 0.89 (0.15, 5.44) | 0.906 | 31 | 0.93 (0.02, 35.44) | 0.97 | 590 | 0.87 (0.26, 2.93) | 0.836 |
| 302 | Sexual and gender identity disorders | 1052 | 17620491 | 445 | 1.01 (0.51, 2.04) | 0.97 | 138 | 1.12 (0.34, 3.63) | 0.861 | 49 | 1.04 (0.19, 5.76) | 0.97 | 913 | 1.18 (0.57, 2.43) | 0.664 |
| 302.1 | Decreased libido | 169 | 17629838 | 69 | 1.06 (0.16, 6.78) | 0.958 | 28 | 1.52 (0.77, 2.99) | 0.225 | 9 | 1.27 (0.1, 16.27) | 0.863 | 142 | 0.94 (0.12, 7.62) | 0.959 |
| 303 | Psychogenic and somatoform disorders | 2866 | 17577631 | 1194 | 0.96 (0.69, 1.33) | 0.808 | 335 | 0.96 (0.51, 1.82) | 0.906 | 136 | 1.01 (0.67, 1.53) | 0.97 | 2406 | 0.94 (0.65, 1.37) | 0.773 |
| 303.1 | Dissociative disorder | 522 | 17619353 | 235 | 1.02 (0.39, 2.68) | 0.97 | 53 | 0.87 (0.18, 4.2) | 0.876 | 14 | 0.58 (0.14, 2.4) | 0.463 | 432 | 0.89 (0.3, 2.68) | 0.851 |
| 303.3 | Psychogenic disorder | 1149 | 17602800 | 459 | 0.89 (0.62, 1.28) | 0.539 | 138 | 0.94 (0.31, 2.89) | 0.924 | 54 | 0.96 (0.16, 5.82) | 0.97 | 966 | 0.95 (0.4, 2.23) | 0.906 |
| 303.4 | Somatoform disorder | 1137 | 17620600 | 480 | 1 (0.97, 1.03) | 0.97 | 129 | 0.95 (0.33, 2.68) | 0.923 | 65 | 1.24 (0.64, 2.41) | 0.535 | 953 | 0.93 (0.49, 1.75) | 0.826 |
| 304 | Adjustment reaction | 4219 | 17583165 | 1787 | 0.98 (0.66, 1.46) | 0.929 | 468 | 0.92 (0.67, 1.27) | 0.624 | 211 | 1.07 (0.52, 2.22) | 0.861 | 3596 | 1.04 (0.68, 1.61) | 0.858 |
| 305.2 | Eating disorder | 871 | 17624339 | 394 | 1.12 (0.71, 1.76) | 0.644 | 97 | 0.9 (0.3, 2.72) | 0.861 | 36 | 0.89 (0.14, 5.88) | 0.915 | 734 | 0.93 (0.36, 2.41) | 0.89 |
| 305.21 | Anorexia nervosa | 588 | 17622677 | 252 | 1 (0.99, 1.01) | 0.97 | 64 | 0.83 (0.4, 1.73) | 0.629 | 25 | 0.87 (0.13, 5.66) | 0.892 | 488 | 0.84 (0.5, 1.41) | 0.531 |
| 306 | Other mental disorder | 6671 | 17575887 | 2857 | 1.02 (0.81, 1.29) | 0.885 | 809 | 1.01 (0.72, 1.42) | 0.97 | 296 | 0.96 (0.56, 1.67) | 0.903 | 5689 | 1.03 (0.81, 1.32) | 0.796 |
| 306.9 | Tension headache | 6038 | 17579963 | 2596 | 1.03 (0.86, 1.23) | 0.777 | 733 | 1.02 (0.62, 1.67) | 0.956 | 270 | 0.98 (0.45, 2.13) | 0.958 | 5162 | 1.05 (0.88, 1.26) | 0.577 |
| 312 | Conduct disorders | 361 | 17627951 | 147 | 0.96 (0.16, 5.72) | 0.97 | 49 | 1.11 (0.13, 9.65) | 0.932 | 18 | 1.07 (0.05, 24.72) | 0.97 | 298 | 0.84 (0.26, 2.66) | 0.773 |
| 313 | Pervasive developmental disorders | 2254 | 17593942 | 945 | 0.99 (0.73, 1.36) | 0.97 | 282 | 0.99 (0.58, 1.68) | 0.97 | 106 | 0.99 (0.63, 1.56) | 0.97 | 1894 | 0.91 (0.64, 1.3) | 0.624 |
| 313.1 | Attention deficit hyperactivity disorder | 216 | 17629922 | 100 | 1.12 (0.25, 5.11) | 0.888 | 23 | 0.86 (0.06, 12.93) | 0.923 | 7 | 0.7 (0.02, 32.46) | 0.865 | 191 | 1.33 (0.31, 5.75) | 0.712 |
| 313.2 | Tics and stuttering | 466 | 17625907 | 178 | 0.83 (0.56, 1.22) | 0.341 | 66 | 1.03 (0.26, 4.02) | 0.97 | 14 | 0.58 (0.21, 1.61) | 0.3 | 385 | 0.82 (0.48, 1.41) | 0.487 |
| 313.3 | Autism | 504 | 17628281 | 199 | 0.91 (0.48, 1.74) | 0.796 | 64 | 0.99 (0.49, 1.98) | 0.97 | 32 | 1.32 (0.59, 2.98) | 0.507 | 419 | 0.85 (0.49, 1.5) | 0.597 |
| 315 | Develomental delays and disorders | 1949 | 17601176 | 811 | 0.97 (0.56, 1.68) | 0.924 | 243 | 1.01 (0.68, 1.5) | 0.97 | 92 | 1 (0.84, 1.2) | 0.97 | 1652 | 0.98 (0.48, 2.04) | 0.97 |
| 315.2 | Speech and language disorder | 221 | 17629502 | 92 | 1 (0.86, 1.17) | 0.97 | 27 | 1.01 (0.61, 1.67) | 0.97 | 14 | 1.39 (0.27, 7.01) | 0.706 | 194 | 1.27 (0.4, 4.02) | 0.7 |
| 315.3 | Mental retardation | 1553 | 17605095 | 640 | 0.95 (0.54, 1.67) | 0.881 | 194 | 1 (0.91, 1.1) | 0.97 | 74 | 1 (0.87, 1.15) | 0.97 | 1316 | 0.98 (0.39, 2.47) | 0.97 |
| 316 | Substance addiction and disorders | 3520 | 17598011 | 1534 | 1.03 (0.67, 1.6) | 0.897 | 400 | 0.96 (0.47, 1.97) | 0.92 | 164 | 1.02 (0.36, 2.89) | 0.97 | 2963 | 0.96 (0.55, 1.65) | 0.881 |
| 317 | Alcohol-related disorders | 30907 | 17268964 | 13593 | 1.03 (0.96, 1.09) | 0.462 | 3423 | 0.96 (0.87, 1.07) | 0.512 | 1294 | 0.93 (0.82, 1.05) | 0.235 | 26035 | 0.98 (0.86, 1.12) | 0.79 |
| 317.1 | Alcoholism | 6553 | 17581395 | 2908 | 1.03 (0.89, 1.21) | 0.693 | 722 | 0.99 (0.7, 1.41) | 0.97 | 274 | 0.95 (0.56, 1.61) | 0.864 | 5504 | 0.98 (0.72, 1.32) | 0.883 |
| 317.11 | Alcoholic liver damage | 4796 | 17599244 | 2120 | 1.01 (0.7, 1.45) | 0.97 | 496 | 0.93 (0.72, 1.2) | 0.597 | 202 | 0.95 (0.48, 1.85) | 0.88 | 4010 | 0.96 (0.75, 1.22) | 0.739 |
| 318 | Tobacco use disorder | 19440 | 17524678 | 8346 | 0.99 (0.86, 1.15) | 0.929 | 2246 | 0.99 (0.75, 1.32) | 0.97 | 843 | 0.95 (0.81, 1.12) | 0.551 | 16414 | 0.99 (0.78, 1.27) | 0.97 |
| **Neurological** | | | | | | | | | | | | | | | |
| 320 | Meningitis | 3199 | 17577999 | 1404 | 1.05 (0.78, 1.42) | 0.767 | 363 | 0.95 (0.5, 1.81) | 0.893 | 143 | 0.98 (0.37, 2.59) | 0.97 | 2692 | 0.95 (0.62, 1.46) | 0.823 |
| 323 | Encephalitis | 1134 | 17616670 | 471 | 0.92 (0.56, 1.5) | 0.742 | 121 | 0.86 (0.41, 1.83) | 0.715 | 51 | 0.93 (0.09, 9.43) | 0.958 | 973 | 1.1 (0.5, 2.41) | 0.817 |
| 323.2 | Acute (transverse) myelitis | 141 | 17630609 | 67 | 1.14 (0.28, 4.71) | 0.863 | 12 | 0.74 (0.1, 5.74) | 0.79 | 6 | 0.96 (0.14, 6.43) | 0.97 | 114 | 0.77 (0.26, 2.23) | 0.64 |
| 323.8 | Encephalitis, non-infectious | 141 | 17629118 | 50 | 0.7 (0.1, 4.78) | 0.73 | 16 | 0.83 (0, 224.71) | 0.952 | 7 | 0.93 (0.02, 34.34) | 0.97 | 120 | 1.05 (0.13, 8.39) | 0.97 |
| 324 | Other CNS infection and poliomyelitis | 571 | 17627360 | 245 | 1.03 (0.24, 4.44) | 0.97 | 74 | 1.19 (0.39, 3.62) | 0.767 | 27 | 1.1 (0.04, 27.44) | 0.958 | 495 | 1.21 (0.49, 3.02) | 0.695 |
| 327 | Sleep disorders | 14393 | 17538153 | 6169 | 0.97 (0.9, 1.05) | 0.483 | 1587 | 0.96 (0.84, 1.09) | 0.544 | 649 | 0.99 (0.73, 1.36) | 0.97 | 12105 | 0.98 (0.81, 1.19) | 0.86 |
| 327.3 | Sleep apnea | 10411 | 17552401 | 4467 | 0.97 (0.88, 1.07) | 0.539 | 1126 | 0.94 (0.83, 1.06) | 0.32 | 480 | 1.01 (0.53, 1.94) | 0.97 | 8729 | 0.96 (0.85, 1.09) | 0.576 |
| 327.4 | Insomnia | 282 | 17630143 | 110 | 0.84 (0.38, 1.87) | 0.686 | 31 | 0.86 (0.13, 5.72) | 0.885 | 16 | 1.15 (0.06, 21.76) | 0.933 | 239 | 1.01 (0.68, 1.49) | 0.97 |
| 327.41 | Organic or persistent insomnia | 216 | 17630302 | 94 | 1.08 (0.3, 3.95) | 0.912 | 29 | 1.22 (0.31, 4.77) | 0.784 | 10 | 1.09 (0.02, 55.22) | 0.97 | 180 | 0.91 (0.18, 4.5) | 0.915 |
| 327.6 | Circadian rhythm sleep disorder | 254 | 17629894 | 102 | 0.85 (0.38, 1.91) | 0.708 | 29 | 0.87 (0.13, 6.06) | 0.898 | 9 | 0.7 (0.07, 7.58) | 0.784 | 207 | 0.79 (0.34, 1.85) | 0.606 |
| 331 | Other cerebral degenerations | 4449 | 17606997 | 1844 | 0.92 (0.79, 1.06) | 0.257 | 514 | 0.99 (0.54, 1.82) | 0.97 | 186 | 0.9 (0.5, 1.61) | 0.725 | 3745 | 0.99 (0.73, 1.35) | 0.97 |
| 331.1 | Hydrocephalus | 2724 | 17615051 | 1109 | 0.89 (0.74, 1.06) | 0.194 | 309 | 0.93 (0.48, 1.81) | 0.847 | 117 | 0.9 (0.35, 2.27) | 0.827 | 2283 | 0.96 (0.51, 1.78) | 0.897 |
| 331.9 | Cerebral degeneration, unspecified | 723 | 17628122 | 306 | 0.95 (0.49, 1.86) | 0.901 | 84 | 1.06 (0.34, 3.32) | 0.929 | 31 | 0.96 (0.14, 6.74) | 0.97 | 612 | 1.05 (0.42, 2.64) | 0.92 |
| 333 | Extrapyramidal disease and abnormal movement disorders | 2996 | 17609538 | 1240 | 0.92 (0.79, 1.06) | 0.256 | 339 | 0.96 (0.54, 1.73) | 0.911 | 132 | 0.94 (0.4, 2.24) | 0.901 | 2515 | 0.98 (0.57, 1.68) | 0.933 |
| 333.1 | Essential tremor | 698 | 17627033 | 301 | 1.02 (0.45, 2.3) | 0.97 | 82 | 1.1 (0.35, 3.47) | 0.883 | 35 | 1.16 (0.25, 5.41) | 0.86 | 584 | 0.97 (0.25, 3.73) | 0.966 |
| 333.4 | Torsion dystonia | 1480 | 17619180 | 620 | 0.94 (0.67, 1.32) | 0.724 | 163 | 0.92 (0.48, 1.76) | 0.817 | 66 | 0.95 (0.22, 4.14) | 0.949 | 1239 | 0.95 (0.52, 1.71) | 0.864 |
| 333.8 | Other degenerative diseases of the basal ganglia | 381 | 17630209 | 144 | **0.74 (0.58, 0.95)** | **0.016** | 43 | 0.9 (0.26, 3.14) | 0.875 | 11 | 0.56 (0.23, 1.39) | 0.214 | 320 | 1 (0.95, 1.05) | 0.97 |
| 334 | Degenerative disease of the spinal cord | 5503 | 17610120 | 2358 | 1 (0.86, 1.16) | 0.97 | 651 | 1.08 (0.77, 1.52) | 0.655 | 260 | 1.08 (0.5, 2.33) | 0.861 | 4591 | 0.95 (0.7, 1.29) | 0.739 |
| 334.1 | Spinocerebellar disease | 216 | 482914* | 88 | 0.9 (0.18, 4.59)** | 0.906 | 24 | 0.93 (0.03, 30.69)** | 0.97 | 11 | 1.08 (0.03, 34.24)** | 0.97 | 181 | 0.96 (0.13, 7.24)** | 0.97 |
| 334.2 | Anterior horn cell disease | 543 | 17628250 | 224 | 0.91 (0.26, 3.19) | 0.894 | 58 | 0.92 (0.07, 12.52) | 0.954 | 29 | 1.15 (0.06, 23.3) | 0.933 | 460 | 1.04 (0.19, 5.73) | 0.97 |
| 335 | Multiple sclerosis | 138 | 17628202 | 55 | 0.92 (0.18, 4.6) | 0.923 | 16 | 1.05 (0.13, 8.75) | 0.97 | 10 | 1.62 (0.43, 6.13) | 0.487 | 111 | 0.77 (0.3, 2.01) | 0.611 |
| 337 | Disorders of the autonomic nervous system | 1030 | 17624022 | 433 | 0.94 (0.57, 1.55) | 0.818 | 125 | 1.05 (0.34, 3.19) | 0.943 | 35 | 0.73 (0.34, 1.6) | 0.445 | 874 | 1.05 (0.43, 2.55) | 0.927 |
| 337.1 | Peripheral autonomic neuropathy | 548 | 17628145 | 233 | 0.95 (0.43, 2.08) | 0.898 | 68 | 1.08 (0.33, 3.47) | 0.908 | 14 | 0.55 (0.24, 1.29) | 0.17 | 466 | 1.07 (0.39, 2.92) | 0.906 |
| 338 | Pain | 34421 | 17467353 | 14905 | 1.03 (0.99, 1.07) | 0.208 | 4050 | 1.03 (0.95, 1.11) | 0.548 | 1545 | 1 (0.92, 1.09) | 0.97 | 29078 | 1 (0.83, 1.19) | 0.962 |
| 338.1 | Acute pain | 25349 | 17521135 | 10941 | 1.02 (0.96, 1.09) | 0.51 | 3016 | 1.04 (0.95, 1.13) | 0.424 | 1138 | 1 (0.94, 1.07) | 0.97 | 21458 | 1.01 (0.85, 1.2) | 0.935 |
| 338.2 | Chronic pain | 11033 | 17571080 | 4821 | 1.04 (0.97, 1.11) | 0.33 | 1266 | 1 (0.79, 1.27) | 0.97 | 489 | 0.99 (0.64, 1.52) | 0.97 | 9311 | 0.99 (0.76, 1.29) | 0.95 |
| 339 | Other headache syndromes | 3466 | 17603269 | 1514 | 1.09 (0.98, 1.21) | 0.096 | 453 | **1.16 (1.02, 1.33)** | **0.027** | 164 | 1.09 (0.7, 1.7) | 0.707 | 2961 | 1.06 (0.82, 1.36) | 0.695 |
| 340 | Migraine | 12365 | 17473751 | 5352 | 1.04 (0.94, 1.15) | 0.442 | 1492 | 1.02 (0.76, 1.37) | 0.885 | 549 | 0.98 (0.53, 1.82) | 0.954 | 10493 | 1.01 (0.73, 1.38) | 0.97 |
| 340.1 | Migrain with aura | 3450 | 17605363 | 1509 | 1.08 (0.97, 1.22) | 0.171 | 433 | 1.07 (0.82, 1.41) | 0.624 | 159 | 1.04 (0.47, 2.29) | 0.935 | 2907 | 0.95 (0.73, 1.23) | 0.708 |
| 341 | Other demyelinating diseases of central nervous system | 709 | 17624623 | 300 | 0.95 (0.41, 2.23) | 0.917 | 79 | 0.9 (0.28, 2.87) | 0.866 | 27 | 0.8 (0.19, 3.33) | 0.767 | 608 | 1.09 (0.38, 3.09) | 0.883 |
| 342 | Hemiplegia | 676 | 17626392 | 301 | 1.1 (0.61, 2.01) | 0.758 | 87 | 1.16 (0.51, 2.65) | 0.732 | 28 | 0.96 (0.17, 5.47) | 0.97 | 551 | 0.8 (0.53, 1.21) | 0.294 |
| 343 | Infantile cerebral palsy | 1542 | 17605402 | 683 | 1.05 (0.57, 1.92) | 0.887 | 180 | 0.94 (0.36, 2.46) | 0.908 | 52 | 0.72 (0.35, 1.48) | 0.376 | 1319 | 1.05 (0.42, 2.6) | 0.931 |
| 344 | Other paralytic syndromes | 2595 | 17612279 | 1140 | 1.03 (0.69, 1.54) | 0.9 | 288 | 0.97 (0.49, 1.89) | 0.924 | 112 | 0.96 (0.3, 3.11) | 0.949 | 2191 | 0.99 (0.77, 1.29) | 0.97 |
| 345 | Epilepsy, recurrent seizures, convulsions | 23469 | 17228780 | 9960 | 0.98 (0.89, 1.08) | 0.714 | 2717 | 0.95 (0.86, 1.05) | 0.307 | 1051 | 0.95 (0.76, 1.2) | 0.7 | 19711 | **0.94 (0.89, 1)** | **0.038** |
| 345.1 | Epilepsy | 2691 | 17607393 | 1145 | 0.97 (0.65, 1.44) | 0.885 | 307 | 0.96 (0.51, 1.8) | 0.898 | 112 | 0.89 (0.44, 1.81) | 0.767 | 2256 | 0.95 (0.63, 1.42) | 0.802 |
| 345.11 | Generalized convulsive epilepsy | 2425 | 17600946 | 1032 | 1.02 (0.59, 1.75) | 0.949 | 304 | 1.06 (0.6, 1.88) | 0.849 | 117 | 1.06 (0.41, 2.79) | 0.909 | 2017 | 0.88 (0.72, 1.09) | 0.248 |
| 345.12 | Partial epilepsy | 4915 | 17583062 | 2102 | 0.98 (0.68, 1.43) | 0.935 | 544 | 0.94 (0.67, 1.32) | 0.738 | 232 | 1.03 (0.35, 2.98) | 0.965 | 4120 | 0.95 (0.73, 1.23) | 0.7 |
| 345.3 | Convulsions | 14391 | 17351676 | 6076 | 0.98 (0.8, 1.21) | 0.883 | 1700 | 0.96 (0.79, 1.15) | 0.644 | 668 | 0.98 (0.5, 1.96) | 0.967 | 12065 | **0.92 (0.85, 0.99)** | **0.034** |
| 346 | Abnormal findings on study of brain and/or nervous system | 743 | 17628175 | 324 | 1.04 (0.51, 2.12) | 0.924 | 92 | 1.08 (0.41, 2.84) | 0.883 | 29 | 0.87 (0.19, 4) | 0.873 | 621 | 0.93 (0.46, 1.89) | 0.845 |
| 346.2 | Nonspecific abnormal results of function study of brain and central nervous system | 638 | 17628979 | 273 | 1 (0.81, 1.23) | 0.97 | 78 | 1.04 (0.24, 4.56) | 0.963 | 25 | 0.86 (0.17, 4.31) | 0.861 | 534 | 0.93 (0.39, 2.21) | 0.885 |
| 347 | Cataplexy and narcolepsy | 314 | 17628686 | 133 | 0.96 (0.16, 5.77) | 0.97 | 42 | 1.08 (0.05, 21.51) | 0.965 | 6 | 0.4 (0.04, 4.39) | 0.462 | 267 | 1.02 (0.41, 2.52) | 0.97 |
| 348 | Other conditions of brain | 3263 | 17608090 | 1446 | 1.07 (0.86, 1.34) | 0.537 | 385 | 1.07 (0.64, 1.79) | 0.819 | 152 | 1.07 (0.43, 2.69) | 0.887 | 2773 | 1.04 (0.6, 1.79) | 0.898 |
| 348.2 | Cerebral edema and compression of brain | 643 | 17628958 | 300 | 1.23 (0.98, 1.54) | 0.072 | 80 | 1.18 (0.66, 2.11) | 0.597 | 29 | 1.1 (0.23, 5.4) | 0.91 | 535 | 0.89 (0.52, 1.54) | 0.7 |
| 348.4 | Cerebral cysts | 463 | 17628776 | 195 | 1 (0.81, 1.24) | 0.97 | 56 | 1.03 (0.21, 5.13) | 0.97 | 26 | 1.25 (0.47, 3.3) | 0.672 | 393 | 1.01 (0.55, 1.85) | 0.97 |
| 348.7 | Coma | 776 | 17628835 | 357 | 1.16 (0.88, 1.51) | 0.3 | 88 | 1.09 (0.39, 2.99) | 0.882 | 38 | 1.19 (0.45, 3.1) | 0.742 | 668 | 1.15 (0.72, 1.84) | 0.576 |
| 348.8 | Encephalopathy, not elsewhere classified | 484 | 17627794 | 207 | 0.93 (0.33, 2.61) | 0.901 | 57 | 0.98 (0.3, 3.15) | 0.97 | 10 | 0.43 (0.12, 1.6) | 0.212 | 408 | 0.99 (0.72, 1.37) | 0.97 |
| 348.9 | Other conditions of brain, NOS | 591 | 17622642 | 260 | 1.08 (0.47, 2.48) | 0.864 | 74 | 1.15 (0.4, 3.27) | 0.808 | 28 | 1.11 (0.12, 10.18) | 0.935 | 511 | 1.17 (0.55, 2.52) | 0.693 |
| 349 | Other and unspecified disorders of the nervous system | 10929 | 17584780 | 4679 | 0.99 (0.82, 1.19) | 0.929 | 1268 | 1.03 (0.83, 1.26) | 0.824 | 496 | 1.01 (0.58, 1.75) | 0.966 | 9136 | 0.95 (0.88, 1.01) | 0.12 |
| 350 | Abnormal movement | 7396 | 17578858 | 3200 | 1.01 (0.73, 1.4) | 0.97 | 848 | 1 (0.94, 1.06) | 0.97 | 327 | 0.98 (0.39, 2.45) | 0.97 | 6245 | 1 (0.98, 1.02) | 0.97 |
| 350.1 | Abnormal involuntary movements | 5605 | 17593256 | 2434 | 1.01 (0.74, 1.36) | 0.97 | 626 | 0.96 (0.58, 1.6) | 0.883 | 243 | 0.95 (0.43, 2.09) | 0.912 | 4742 | 1.01 (0.73, 1.39) | 0.97 |
| 350.2 | Abnormality of gait | 1472 | 17621234 | 624 | 0.98 (0.43, 2.22) | 0.966 | 179 | 1.09 (0.55, 2.17) | 0.824 | 67 | 1.02 (0.36, 2.89) | 0.97 | 1234 | 0.98 (0.35, 2.71) | 0.965 |
| 350.3 | Lack of coordination | 252 | 17628756 | 127 | 1.37 (0.82, 2.3) | 0.235 | 29 | 1.17 (0.16, 8.51) | 0.885 | 8 | 0.82 (0.02, 41.95) | 0.929 | 211 | 0.95 (0.08, 10.78) | 0.968 |
| 350.6 | Disturbances of sensation of smell and taste | 164 | 17630323 | 63 | 0.9 (0.15, 5.29) | 0.911 | 22 | 1.18 (0.1, 13.77) | 0.904 | 12 | 1.63 (0.27, 9.76) | 0.606 | 139 | 1.02 (0.32, 3.29) | 0.97 |
| 351 | Other peripheral nerve disorders | 18968 | 17465196 | 8233 | 1.01 (0.83, 1.22) | 0.943 | 2122 | 0.99 (0.75, 1.29) | 0.929 | 831 | 0.98 (0.68, 1.41) | 0.906 | 16039 | 1.02 (0.83, 1.24) | 0.879 |
| 352 | Disorders of other cranial nerves | 5530 | 17573865 | 2324 | 0.96 (0.79, 1.16) | 0.671 | 646 | 1 (0.82, 1.22) | 0.97 | 247 | 0.97 (0.4, 2.33) | 0.949 | 4722 | 1.08 (0.88, 1.31) | 0.471 |
| 352.1 | Trigeminal nerve disorders [CN5] | 1858 | 17612532 | 779 | 0.93 (0.64, 1.35) | 0.708 | 198 | 0.9 (0.47, 1.74) | 0.773 | 82 | 0.94 (0.17, 5.1) | 0.949 | 1562 | 0.98 (0.43, 2.24) | 0.97 |
| 352.2 | Facial nerve disorders [CN7] | 3506 | 17595378 | 1482 | 0.98 (0.63, 1.52) | 0.935 | 424 | 1.03 (0.56, 1.9) | 0.921 | 157 | 0.98 (0.39, 2.45) | 0.97 | 3022 | 1.14 (0.96, 1.36) | 0.14 |
| 353 | Nerve root and plexus disorders | 2124 | 17617149 | 933 | 1.06 (0.8, 1.4) | 0.703 | 249 | 1.09 (0.71, 1.68) | 0.7 | 108 | 1.19 (0.75, 1.88) | 0.462 | 1799 | 1.03 (0.56, 1.89) | 0.924 |
| 353.1 | Nerve plexus lesions | 876 | 17623758 | 406 | 1.16 (0.87, 1.55) | 0.319 | 97 | 1.05 (0.29, 3.86) | 0.944 | 41 | 1.13 (0.25, 5.01) | 0.883 | 756 | 1.17 (0.73, 1.88) | 0.531 |
| 353.2 | Nerve root lesions | 729 | 17627680 | 318 | 1.02 (0.5, 2.11) | 0.956 | 85 | 1.06 (0.46, 2.46) | 0.897 | 32 | 1 (0.86, 1.16) | 0.97 | 610 | 0.96 (0.49, 1.88) | 0.907 |
| 355 | Complex regional/central pain syndrome | 182 | 17630726 | 77 | 0.98 (0.44, 2.17) | 0.97 | 22 | 1.1 (0.14, 8.55) | 0.935 | 9 | 1.13 (0.03, 36.62) | 0.952 | 143 | 0.69 (0.43, 1.1) | 0.114 |
| 356 | Hereditary and idiopathic peripheral neuropathy | 611 | 482914* | 272 | 0.99 (0.64, 1.53)** | 0.97 | 58 | 0.81 (0.34, 1.91)** | 0.637 | 21 | 0.74 (0.17, 3.21)** | 0.697 | 522 | 1.09 (0.36, 3.34)** | 0.888 |
| 357 | Inflammatory and toxic neuropathy | 5784 | 17590723 | 2548 | 1.01 (0.67, 1.53) | 0.958 | 596 | 0.93 (0.69, 1.26) | 0.671 | 265 | 1.04 (0.48, 2.22) | 0.935 | 4900 | 1.05 (0.81, 1.35) | 0.734 |
| 358 | Myoneural disorders | 608 | 17627104 | 234 | 0.8 (0.49, 1.32) | 0.398 | 71 | 0.93 (0.11, 8.23) | 0.956 | 27 | 0.89 (0.04, 19.38) | 0.949 | 526 | 1.19 (0.46, 3.13) | 0.733 |
| 358.1 | Myasthenia gravis | 306 | 17628555 | 118 | 0.84 (0.18, 3.98) | 0.836 | 35 | 0.94 (0.05, 17.74) | 0.97 | 19 | 1.29 (0.03, 47.92) | 0.898 | 265 | 1.2 (0.1, 13.92) | 0.894 |
| 359 | Muscular dystrophies and other myopathies | 2467 | 17606241 | 1028 | 0.94 (0.73, 1.22) | 0.675 | 290 | 0.99 (0.57, 1.71) | 0.97 | 110 | 0.96 (0.28, 3.24) | 0.949 | 2073 | 0.96 (0.57, 1.63) | 0.9 |
| 359.1 | Muscular dystrophies | 372 | 17627028 | 167 | 1.09 (0.32, 3.66) | 0.9 | 41 | 0.95 (0.07, 13.7) | 0.97 | 17 | 1.02 (0.34, 3.08) | 0.97 | 322 | 1.15 (0.26, 5.2) | 0.863 |
| 359.2 | Myopathy | 2207 | 17610042 | 908 | 0.92 (0.75, 1.13) | 0.445 | 261 | 0.99 (0.56, 1.73) | 0.97 | 98 | 0.94 (0.32, 2.77) | 0.924 | 1850 | 0.95 (0.58, 1.57) | 0.857 |
| **Sense Organs** | | | | | | | | | | | | | | | |
| 360 | Disorders of the globe | 943 | 17621030 | 399 | 0.98 (0.38, 2.53) | 0.97 | 113 | 1.03 (0.27, 3.93) | 0.97 | 42 | 0.98 (0.34, 2.8) | 0.97 | 810 | 1.12 (0.64, 1.96) | 0.706 |
| 360.2 | Progressive myopia | 563 | 17625541 | 248 | 1.07 (0.44, 2.61) | 0.883 | 71 | 1.11 (0.3, 4.06) | 0.883 | 22 | 0.89 (0.07, 10.99) | 0.934 | 488 | 1.18 (0.56, 2.51) | 0.675 |
| 361 | Retinal detachments and defects | 5399 | 17572886 | 2373 | 1.04 (0.85, 1.28) | 0.724 | 606 | 1.06 (0.76, 1.47) | 0.747 | 267 | 1.15 (0.86, 1.55) | 0.347 | 4550 | 1.02 (0.6, 1.73) | 0.956 |
| 361.1 | Retinal detachment with retinal defect | 3037 | 17594394 | 1366 | 1.11 (0.95, 1.3) | 0.171 | 351 | 1.14 (0.86, 1.52) | 0.362 | 164 | **1.32 (1, 1.73)** | **0.046** | 2542 | 0.97 (0.54, 1.76) | 0.938 |
| 361.2 | Retinoschisis and retinal cysts | 395 | 17627612 | 157 | 0.84 (0.49, 1.44) | 0.542 | 44 | 0.94 (0.12, 7.4) | 0.959 | 18 | 0.95 (0.09, 10.19) | 0.97 | 328 | 0.93 (0.24, 3.52) | 0.92 |
| 362 | Other retinal disorders | 18937 | 17484224 | 8314 | 1.02 (0.89, 1.17) | 0.807 | 2035 | 1 (0.88, 1.14) | 0.97 | 890 | 1.09 (0.91, 1.29) | 0.364 | 15904 | 1 (0.93, 1.08) | 0.97 |
| 362.1 | Retinopathy of prematurity | 275 | 17628264 | 117 | 1.11 (0.01, 187.69) | 0.97 | 42 | 1.29 (0, 191299.31) | 0.97 | 14 | 1.16 (0, 1437.33) | 0.97 | 237 | 1.1 (0.01, 101.12) | 0.97 |
| 362.29 | Macular degeneration (senile) of retina NOS | 11754 | 17545346 | 5235 | 1.04 (0.71, 1.53) | 0.86 | 1250 | 1.01 (0.54, 1.88) | 0.97 | 516 | 1.02 (0.33, 3.17) | 0.97 | 9857 | 1 (0.87, 1.16) | 0.97 |
| 362.3 | Other nondiabetic retinopathy | 1697 | 17623750 | 741 | 0.99 (0.67, 1.46) | 0.97 | 175 | 0.95 (0.43, 2.11) | 0.906 | 76 | 1.01 (0.58, 1.75) | 0.97 | 1423 | 0.99 (0.6, 1.64) | 0.97 |
| 362.31 | Separation of retinal layers | 571 | 17627940 | 229 | 0.91 (0.36, 2.32) | 0.859 | 78 | 1.18 (0.46, 3.07) | 0.741 | 27 | 1.04 (0.15, 7.29) | 0.97 | 459 | 0.76 (0.49, 1.16) | 0.203 |
| 362.4 | Retinal vascular changes and abnomalities | 5501 | 17583568 | 2397 | 1.01 (0.78, 1.3) | 0.97 | 574 | 0.96 (0.6, 1.53) | 0.87 | 281 | 1.16 (0.89, 1.53) | 0.273 | 4665 | 1.06 (0.84, 1.34) | 0.648 |
| 362.6 | Peripheral retinal degenerations | 165 | 17629858 | 77 | 1.22 (0.48, 3.1) | 0.695 | 18 | 1.12 (0.08, 14.94) | 0.938 | 10 | 1.54 (0.29, 8.19) | 0.624 | 139 | 1.01 (0.67, 1.52) | 0.97 |
| 362.7 | Hereditary retinal dystrophies | 245 | 482914* | 82 | 0.74 (0.39, 1.41)** | 0.368 | 37 | 1.22 (0.26, 5.6)** | 0.814 | 20 | 1.69 (0.61, 4.71)** | 0.319 | 209 | 1.05 (0.09, 12.2)** | 0.97 |
| 362.8 | Retinal hemorrhage/ischemia | 264 | 17629250 | 121 | 1.25 (0.62, 2.51) | 0.549 | 33 | 1.32 (0.4, 4.36) | 0.664 | 18 | 1.8 (0.69, 4.69) | 0.232 | 223 | 1.02 (0.41, 2.55) | 0.97 |
| 363 | Chorioretinal inflammations, scars, and other disorders of choroid | 355 | 17628340 | 167 | 1.24 (0.79, 1.94) | 0.359 | 40 | 1.13 (0.24, 5.43) | 0.883 | 20 | 1.43 (0.47, 4.3) | 0.539 | 291 | 0.85 (0.4, 1.81) | 0.68 |
| 364 | Corneal opacity and other disorders of cornea | 2390 | 17611427 | 1066 | 1.05 (0.09, 12.78) | 0.97 | 254 | 0.96 (0.15, 6.09) | 0.97 | 109 | 1.04 (0.17, 6.22) | 0.97 | 2025 | 1.03 (0.21, 5.1) | 0.97 |
| 364.1 | Corneal opacity | 380 | 17627753 | 172 | 1.16 (0, 1221.27) | 0.97 | 51 | 1.31 (0, 82342) | 0.965 | 16 | 1.04 (0.19, 5.66) | 0.97 | 329 | 1.2 (0, 6344.35) | 0.97 |
| 364.2 | Corneal edema | 168 | 17630098 | 72 | 1.05 (0.12, 9.03) | 0.97 | 23 | 1.32 (0.29, 6.04) | 0.734 | 8 | 1.14 (0.01, 129.47) | 0.961 | 138 | 0.87 (0.13, 6) | 0.896 |
| 364.4 | Corneal degenerations | 259 | 17629245 | 113 | 1.07 (0.08, 14.69) | 0.965 | 37 | 1.39 (0.27, 7.17) | 0.708 | 9 | 0.84 (0, 371.73) | 0.959 | 214 | 0.9 (0.06, 13.79) | 0.947 |
| 364.41 | Keratoconus | 321 | 17628644 | 134 | 1.02 (0.41, 2.55) | 0.97 | 46 | 1.25 (0.43, 3.59) | 0.697 | 15 | 1.05 (0.09, 12.51) | 0.97 | 269 | 0.93 (0.18, 4.88) | 0.935 |
| 364.5 | Corneal dystrophy | 772 | 482914* | 375 | 1.18 (0.86, 1.63)** | 0.303 | 61 | 0.77 (0.39, 1.52)** | 0.463 | 37 | 1.15 (0.2, 6.52)** | 0.883 | 644 | 0.96 (0.26, 3.48)** | 0.956 |
| 364.9 | Cornea replaced by transplant | 197 | 17630415 | 93 | 1.17 (0.23, 6.07) | 0.86 | 19 | 0.95 (0.07, 13.06) | 0.97 | 10 | 1.24 (0.02, 89.25) | 0.929 | 162 | 0.87 (0.1, 7.41) | 0.909 |
| 365 | Glaucoma | 5440 | 17577829 | 2302 | 0.97 (0.75, 1.24) | 0.805 | 638 | 1.08 (0.83, 1.41) | 0.578 | 256 | 1.07 (0.56, 2.03) | 0.853 | 4558 | 0.99 (0.56, 1.74) | 0.97 |
| 365.11 | Primary open angle glaucoma | 2608 | 17607225 | 1106 | 0.96 (0.65, 1.44) | 0.863 | 303 | 1.07 (0.64, 1.8) | 0.807 | 118 | 1.02 (0.35, 3.03) | 0.97 | 2190 | 1.01 (0.78, 1.29) | 0.97 |
| 365.2 | Primary angle-closure glaucoma | 1207 | 17617535 | 504 | 0.93 (0.6, 1.42) | 0.738 | 131 | 0.97 (0.27, 3.48) | 0.97 | 58 | 1.06 (0.15, 7.3) | 0.958 | 993 | 0.89 (0.57, 1.38) | 0.613 |
| 366 | Cataract | 56829 | 17217730 | 24851 | 1 (0.85, 1.19) | 0.959 | 6148 | 1.02 (0.85, 1.22) | 0.849 | 2499 | 1.01 (0.69, 1.48) | 0.959 | 47858 | 1.03 (0.96, 1.11) | 0.438 |
| 366.1 | Nonsenile Cataract | 2970 | 17595824 | 1272 | 0.98 (0.6, 1.59) | 0.934 | 335 | 1.02 (0.42, 2.48) | 0.962 | 136 | 1.03 (0.25, 4.25) | 0.97 | 2487 | 0.98 (0.5, 1.89) | 0.95 |
| 366.2 | Senile cataract | 52310 | 17270509 | 22934 | 1.01 (0.9, 1.14) | 0.883 | 5632 | 1.02 (0.85, 1.22) | 0.861 | 2290 | 1.01 (0.69, 1.48) | 0.97 | 44042 | 1.03 (0.96, 1.11) | 0.413 |
| 366.3 | Traumatic cataract | 196 | 17627523 | 88 | 1.01 (0.63, 1.62) | 0.97 | 20 | 0.88 (0.07, 10.76) | 0.928 | 6 | 0.67 (0.04, 12.11) | 0.797 | 163 | 0.92 (0.14, 6.06) | 0.935 |
| 367 | Disorders of refraction and accommodation; blindness and low vision | 11954 | 17524028 | 5065 | 1 (0.83, 1.22) | 0.97 | 1494 | 1.07 (0.93, 1.22) | 0.359 | 554 | 1.02 (0.59, 1.78) | 0.943 | 10155 | 1.02 (0.79, 1.33) | 0.883 |
| 367.1 | Myopia | 5639 | 17577787 | 2353 | 0.98 (0.7, 1.37) | 0.929 | 720 | 1.05 (0.77, 1.43) | 0.76 | 257 | 0.98 (0.47, 2.06) | 0.97 | 4809 | 1.03 (0.73, 1.46) | 0.861 |
| 367.2 | Astigmatism | 1991 | 17615394 | 811 | 0.99 (0.67, 1.48) | 0.97 | 282 | 1.2 (0.9, 1.6) | 0.212 | 109 | 1.22 (0.7, 2.13) | 0.503 | 1701 | 1.04 (0.5, 2.19) | 0.92 |
| 367.4 | Presbyopia | 941 | 17624271 | 399 | 1 (0.87, 1.15) | 0.97 | 116 | 1.12 (0.42, 2.97) | 0.836 | 47 | 1.14 (0.23, 5.72) | 0.885 | 794 | 1.01 (0.77, 1.32) | 0.97 |
| 367.8 | Hypermetropia | 2452 | 17604585 | 1086 | 1.11 (0.93, 1.32) | 0.234 | 295 | 1.05 (0.53, 2.06) | 0.896 | 129 | 1.2 (0.78, 1.85) | 0.405 | 2050 | 0.91 (0.69, 1.2) | 0.512 |
| 367.9 | Blindness and low vision | 2862 | 17614675 | 1221 | 0.98 (0.6, 1.59) | 0.942 | 348 | 1.08 (0.73, 1.6) | 0.713 | 112 | 0.87 (0.47, 1.6) | 0.672 | 2426 | 1.04 (0.62, 1.75) | 0.883 |
| 368 | Visual disturbances | 6403 | 17577780 | 2682 | 0.97 (0.77, 1.23) | 0.818 | 775 | 1.04 (0.72, 1.51) | 0.834 | 311 | 1.07 (0.64, 1.79) | 0.817 | 5445 | 1.04 (0.8, 1.37) | 0.767 |
| 368.1 | Amblyopia | 992 | 17620670 | 440 | 1.07 (0.64, 1.79) | 0.814 | 128 | 1.14 (0.64, 2.05) | 0.67 | 30 | 0.68 (0.33, 1.44) | 0.321 | 838 | 1 (0.82, 1.2) | 0.97 |
| 368.2 | Diplopia and disorders of binocular vision | 1164 | 17622615 | 480 | 0.93 (0.53, 1.61) | 0.802 | 134 | 0.98 (0.39, 2.47) | 0.97 | 58 | 1.07 (0.16, 7.3) | 0.946 | 1011 | 1.23 (0.81, 1.84) | 0.334 |
| 368.3 | Anisometropia | 482 | 17626757 | 193 | 0.95 (0.25, 3.6) | 0.949 | 68 | 1.19 (0.42, 3.36) | 0.761 | 26 | 1.18 (0.15, 9.41) | 0.885 | 400 | 0.87 (0.33, 2.29) | 0.795 |
| 368.4 | Visual field defects | 1658 | 17620114 | 678 | 0.93 (0.65, 1.33) | 0.7 | 191 | 0.99 (0.58, 1.69) | 0.97 | 96 | 1.26 (0.77, 2.06) | 0.364 | 1402 | 1.01 (0.59, 1.74) | 0.97 |
| 368.9 | Subjective visual disturbances | 449 | 17628618 | 202 | 1.14 (0.65, 2) | 0.671 | 52 | 1.08 (0.24, 4.93) | 0.923 | 25 | 1.33 (0.45, 3.94) | 0.616 | 387 | 1.15 (0.48, 2.73) | 0.77 |
| 369 | Infection of the eye | 18641 | 17393048 | 7989 | 1.01 (0.82, 1.24) | 0.937 | 2209 | 1 (0.98, 1.02) | 0.97 | 850 | 1 (0.83, 1.2) | 0.97 | 15744 | 0.98 (0.79, 1.22) | 0.87 |
| 369.2 | Eye infection, viral | 1412 | 17617341 | 595 | 0.98 (0.45, 2.12) | 0.959 | 165 | 1 (0.89, 1.13) | 0.97 | 71 | 1.1 (0.32, 3.77) | 0.885 | 1204 | 1.06 (0.51, 2.22) | 0.883 |
| 369.5 | Conjunctivitis, infectious | 16933 | 17411353 | 7286 | 1.02 (0.87, 1.19) | 0.836 | 2015 | 1.01 (0.77, 1.32) | 0.97 | 762 | 0.99 (0.54, 1.8) | 0.965 | 14300 | 0.98 (0.8, 1.2) | 0.842 |
| 370 | Keratitis | 3909 | 17594472 | 1726 | 1.05 (0.83, 1.32) | 0.697 | 433 | 0.96 (0.56, 1.66) | 0.902 | 174 | 0.99 (0.73, 1.35) | 0.97 | 3294 | 0.98 (0.58, 1.64) | 0.94 |
| 370.1 | Corneal ulcer | 378 | 17627533 | 172 | 1.11 (0.35, 3.49) | 0.873 | 38 | 0.92 (0.08, 10.23) | 0.949 | 20 | 1.23 (0.1, 15.91) | 0.883 | 329 | 1.24 (0.43, 3.56) | 0.7 |
| 370.3 | Keratoconjunctivitis | 713 | 17624257 | 325 | 1.13 (0.61, 2.1) | 0.7 | 82 | 1.05 (0.11, 9.91) | 0.97 | 32 | 1.05 (0.1, 10.83) | 0.97 | 600 | 0.97 (0.22, 4.31) | 0.97 |
| 371 | Inflammation of the eye | 8334 | 17546506 | 3586 | 1.01 (0.7, 1.45) | 0.97 | 935 | 0.95 (0.75, 1.21) | 0.707 | 403 | 1.06 (0.7, 1.6) | 0.8 | 7094 | 1.04 (0.85, 1.27) | 0.718 |
| 371.1 | Uveitis, noninfectious or NOS | 2926 | 17599736 | 1233 | 0.97 (0.65, 1.45) | 0.883 | 331 | 0.96 (0.52, 1.8) | 0.91 | 146 | 1.08 (0.5, 2.38) | 0.849 | 2471 | 1 (0.8, 1.24) | 0.97 |
| 371.2 | Conjunctivitis, noninfectious | 593 | 17625157 | 259 | 1.07 (0.47, 2.41) | 0.882 | 79 | 1.17 (0.55, 2.48) | 0.698 | 22 | 0.84 (0.13, 5.28) | 0.864 | 506 | 1.05 (0.29, 3.84) | 0.946 |
| 371.3 | Inflammation of eyelids | 4504 | 17588977 | 1953 | 1.02 (0.74, 1.41) | 0.906 | 492 | 0.92 (0.71, 1.2) | 0.544 | 222 | 1.08 (0.62, 1.87) | 0.806 | 3856 | 1.08 (0.87, 1.33) | 0.514 |
| 371.33 | Noninfectious dermatoses of eyelid | 124 | 17630905 | 51 | 1.07 (0.12, 9.38) | 0.959 | 18 | 1.34 (0.32, 5.68) | 0.703 | 9 | 1.74 (0.42, 7.29) | 0.455 | 99 | 0.71 (0.29, 1.72) | 0.46 |
| 372 | Disorders of conjunctiva | 3676 | 17595372 | 1542 | 0.98 (0.7, 1.36) | 0.901 | 450 | 1.09 (0.85, 1.4) | 0.491 | 186 | 1.14 (0.8, 1.62) | 0.488 | 3144 | 1.1 (0.92, 1.32) | 0.3 |
| 374 | Other disorders of eyelids | 7119 | 17578854 | 3083 | 1 (0.97, 1.03) | 0.97 | 766 | 0.97 (0.3, 3.08) | 0.958 | 342 | 1.08 (0.33, 3.57) | 0.911 | 5995 | 1 (0.89, 1.13) | 0.97 |
| 374.1 | Ectropion or entropion | 1925 | 17619489 | 841 | 1 (0.94, 1.06) | 0.97 | 197 | 0.95 (0.07, 13.18) | 0.97 | 94 | 1.11 (0.01, 142.55) | 0.97 | 1613 | 0.99 (0.61, 1.62) | 0.97 |
| 374.2 | Lagophthalmos | 302 | 17629955 | 123 | 0.87 (0.29, 2.55) | 0.805 | 24 | 0.65 (0.19, 2.16) | 0.487 | 20 | 1.36 (0.21, 8.93) | 0.763 | 260 | 1.15 (0.19, 6.86) | 0.885 |
| 374.3 | Ptosis of eyelid | 4143 | 17600558 | 1814 | 1.04 (0.81, 1.33) | 0.791 | 460 | 1.02 (0.48, 2.14) | 0.968 | 203 | 1.12 (0.76, 1.66) | 0.578 | 3514 | 1.05 (0.77, 1.43) | 0.784 |
| 375 | Disorders of lacrimal system | 1688 | 17612734 | 719 | 0.93 (0.65, 1.34) | 0.724 | 170 | 0.84 (0.55, 1.28) | 0.428 | 65 | 0.81 (0.37, 1.74) | 0.599 | 1444 | 1.1 (0.69, 1.76) | 0.7 |
| 375.2 | Epiphora | 339 | 17629098 | 147 | 0.95 (0.2, 4.58) | 0.958 | 30 | 0.77 (0.22, 2.7) | 0.698 | 16 | 1.02 (0.44, 2.34) | 0.97 | 304 | 1.65 (0.93, 2.91) | 0.085 |
| 376 | Disorders of the orbit | 303 | 17628341 | 150 | 1.35 (0.91, 2.02) | 0.14 | 37 | 1.23 (0.38, 3.99) | 0.749 | 10 | 0.85 (0.03, 22.89) | 0.929 | 261 | 1.14 (0.28, 4.57) | 0.868 |
| 377 | Disorders of optic nerve and visual pathways | 1564 | 17617888 | 652 | 0.94 (0.61, 1.45) | 0.806 | 193 | 1.02 (0.45, 2.32) | 0.97 | 59 | 0.8 (0.39, 1.63) | 0.549 | 1322 | 0.99 (0.71, 1.4) | 0.97 |
| 377.1 | Optic atrophy | 436 | 17626888 | 164 | 0.79 (0.49, 1.27) | 0.336 | 56 | 0.97 (0.23, 4.12) | 0.97 | 17 | 0.76 (0.09, 6.74) | 0.818 | 375 | 1.11 (0.25, 4.88) | 0.898 |
| 377.3 | Optic neuritis/neuropathy | 457 | 17628611 | 197 | 0.94 (0.35, 2.54) | 0.917 | 47 | 0.87 (0.23, 3.25) | 0.842 | 15 | 0.69 (0.16, 3.07) | 0.644 | 389 | 1.07 (0.26, 4.38) | 0.934 |
| 378 | Strabismus and other disorders of binocular eye movements | 7239 | 17517508 | 3118 | 1.01 (0.68, 1.49) | 0.965 | 817 | 0.94 (0.73, 1.22) | 0.671 | 336 | 1.01 (0.74, 1.37) | 0.97 | 6118 | 0.98 (0.64, 1.5) | 0.947 |
| 378.1 | Strabismus (not specified as paralytic) | 5499 | 17534996 | 2371 | 1.02 (0.76, 1.39) | 0.883 | 630 | 0.96 (0.64, 1.42) | 0.84 | 268 | 1.06 (0.57, 1.96) | 0.858 | 4639 | 0.97 (0.69, 1.35) | 0.849 |
| 378.5 | Paralytic strabismus | 1674 | 17615639 | 714 | 0.96 (0.51, 1.78) | 0.898 | 186 | 0.94 (0.36, 2.51) | 0.915 | 65 | 0.83 (0.31, 2.25) | 0.732 | 1408 | 0.98 (0.38, 2.54) | 0.97 |
| 379 | Other disorders of eye | 15268 | 17516608 | 6549 | 1 (0.91, 1.1) | 0.97 | 1784 | 1.03 (0.75, 1.41) | 0.863 | 712 | 1.04 (0.63, 1.72) | 0.881 | 12934 | 1.03 (0.79, 1.33) | 0.861 |
| 379.1 | Scleritis and episcleritis | 389 | 17627890 | 162 | 0.95 (0.09, 9.59) | 0.97 | 44 | 0.94 (0.06, 15.63) | 0.97 | 20 | 1.1 (0.01, 110.92) | 0.97 | 336 | 1.15 (0.12, 11.06) | 0.909 |
| 379.2 | Disorders of vitreous body | 5579 | 17585972 | 2386 | 0.98 (0.72, 1.34) | 0.898 | 627 | 1.02 (0.56, 1.87) | 0.947 | 259 | 1.04 (0.51, 2.13) | 0.912 | 4673 | 0.98 (0.65, 1.46) | 0.914 |
| 379.3 | Aphakia and other disorders of lens | 1036 | 17613381 | 466 | 1.11 (0.7, 1.75) | 0.68 | 121 | 1.14 (0.54, 2.43) | 0.746 | 52 | 1.22 (0.43, 3.44) | 0.721 | 860 | 0.93 (0.39, 2.21) | 0.883 |
| 379.4 | Anomalies of pupillary function | 247 | 17629163 | 105 | 0.98 (0.33, 2.87) | 0.97 | 22 | 0.75 (0.19, 3.03) | 0.7 | 17 | 1.49 (0.39, 5.68) | 0.575 | 203 | 0.84 (0.26, 2.68) | 0.784 |
| 379.5 | Disorders of iris and ciliary body | 504 | 17626415 | 234 | 1.1 (0.47, 2.54) | 0.843 | 52 | 0.91 (0.17, 5.02) | 0.923 | 15 | 0.67 (0.14, 3.17) | 0.624 | 415 | 0.86 (0.4, 1.83) | 0.708 |
| 379.9 | Pain, swelling or discharge of eye | 1330 | 17624083 | 579 | 1.06 (0.67, 1.68) | 0.817 | 166 | 1.09 (0.55, 2.14) | 0.819 | 60 | 1.02 (0.44, 2.33) | 0.97 | 1149 | 1.15 (0.79, 1.67) | 0.484 |
| 380 | Disorders of external ear | 6705 | 17561999 | 2921 | 1.04 (0.86, 1.26) | 0.7 | 769 | 0.99 (0.72, 1.36) | 0.97 | 330 | 1.1 (0.75, 1.62) | 0.642 | 5638 | 0.96 (0.73, 1.26) | 0.777 |
| 380.1 | Otitis externa | 4544 | 17585245 | 1986 | 1.07 (0.91, 1.26) | 0.438 | 542 | 1.04 (0.63, 1.73) | 0.885 | 234 | 1.17 (0.85, 1.61) | 0.347 | 3830 | 0.97 (0.63, 1.49) | 0.883 |
| 380.4 | Impacted cerumen | 1545 | 17615078 | 676 | 1 (0.99, 1.01) | 0.97 | 160 | 0.88 (0.46, 1.7) | 0.721 | 66 | 0.93 (0.18, 4.81) | 0.935 | 1306 | 1.01 (0.75, 1.34) | 0.97 |
| 381 | Otitis media and Eustachian tube disorders | 22790 | 17144551 | 9978 | **1.07 (1.03, 1.12)** | **0.001** | 2698 | 0.98 (0.77, 1.25) | 0.877 | 1084 | 1.04 (0.8, 1.35) | 0.767 | 19277 | 0.96 (0.87, 1.06) | 0.455 |
| 381.1 | Otitis media | 12313 | 17364091 | 5443 | **1.08 (1.03, 1.15)** | **0.005** | 1447 | 0.99 (0.66, 1.48) | 0.952 | 567 | 1.02 (0.55, 1.87) | 0.959 | 10410 | 0.97 (0.82, 1.14) | 0.7 |
| 381.11 | Suppurative and unspecified otitis media | 11469 | 17406851 | 4994 | 1.07 (0.99, 1.16) | 0.071 | 1384 | 0.99 (0.73, 1.35) | 0.97 | 568 | 1.09 (0.82, 1.44) | 0.575 | 9716 | 0.97 (0.78, 1.2) | 0.767 |
| 381.2 | Eustachian tube disorders | 2358 | 17571118 | 1076 | 1.13 (0.95, 1.35) | 0.163 | 264 | 0.95 (0.45, 2.02) | 0.901 | 97 | 0.92 (0.28, 2.97) | 0.897 | 1964 | 0.88 (0.68, 1.13) | 0.327 |
| 381.3 | Mastoiditis & related conditions | 447 | 17623470 | 197 | 1.07 (0.37, 3.06) | 0.909 | 47 | 0.9 (0.16, 5.01) | 0.914 | 26 | 1.3 (0.32, 5.32) | 0.73 | 382 | 1.06 (0.16, 6.86) | 0.958 |
| 381.9 | Otorrhea | 110 | 17631171 | 43 | 0.86 (0.09, 8.47) | 0.902 | 15 | 1.06 (0.06, 18.17) | 0.97 | 4 | 0.74 (0, 553.51) | 0.934 | 93 | 0.98 (0.43, 2.24) | 0.97 |
| 382 | Otalgia | 1061 | 17625439 | 438 | 0.99 (0.67, 1.47) | 0.97 | 149 | 1.16 (0.74, 1.82) | 0.539 | 47 | 0.96 (0.15, 6.19) | 0.97 | 915 | 1.11 (0.65, 1.87) | 0.717 |
| 383 | Otosclerosis | 2321 | 17592363 | 983 | 0.96 (0.63, 1.46) | 0.863 | 257 | 0.97 (0.38, 2.46) | 0.959 | 109 | 1.03 (0.23, 4.67) | 0.97 | 1945 | 0.97 (0.51, 1.86) | 0.935 |
| 384 | Other disorders of tympanic membrane | 3200 | 17594033 | 1385 | 1.08 (0.91, 1.29) | 0.364 | 428 | 1.18 (0.98, 1.41) | 0.079 | 156 | 1.12 (0.65, 1.93) | 0.7 | 2735 | 1.05 (0.7, 1.58) | 0.818 |
| 384.1 | Myringitis | 241 | 17627123 | 101 | 0.92 (0.22, 3.83) | 0.918 | 23 | 0.72 (0.19, 2.67) | 0.635 | 11 | 0.91 (0.01, 89.68) | 0.97 | 206 | 1.04 (0.14, 7.74) | 0.97 |
| 384.4 | Perforation of tympanic membrane | 2943 | 17598765 | 1276 | 1.1 (0.94, 1.29) | 0.248 | 404 | **1.23 (1.04, 1.45)** | **0.015** | 146 | 1.15 (0.72, 1.86) | 0.566 | 2518 | 1.06 (0.73, 1.55) | 0.775 |
| 385 | Other disorders of middle ear and mastoid | 779 | 17623108 | 362 | 1.21 (0.93, 1.58) | 0.161 | 83 | 1.03 (0.22, 4.88) | 0.97 | 49 | 1.55 (0.98, 2.46) | 0.059 | 663 | 1.05 (0.36, 3.1) | 0.934 |
| 385.3 | Cholesteatoma | 212 | 17630059 | 93 | 1.02 (0.49, 2.11) | 0.97 | 17 | 0.71 (0.16, 3.22) | 0.67 | 15 | 1.58 (0.43, 5.79) | 0.503 | 177 | 0.94 (0.08, 10.92) | 0.965 |
| 385.5 | Tympanosclerosis and middle ear disease related to otitis media | 530 | 17625215 | 256 | **1.33 (1.02, 1.74)** | **0.038** | 61 | 1.17 (0.44, 3.1) | 0.77 | 29 | 1.42 (0.59, 3.42) | 0.445 | 453 | 1.08 (0.34, 3.37) | 0.906 |
| 386 | Vertiginous syndromes and other disorders of vestibular system | 9954 | 17537071 | 4362 | 1.01 (0.76, 1.33) | 0.959 | 1054 | 0.94 (0.8, 1.1) | 0.438 | 427 | 0.95 (0.62, 1.47) | 0.842 | 8426 | 1.03 (0.85, 1.26) | 0.771 |
| 386.1 | Meniere's disease | 1400 | 17612764 | 637 | 1.1 (0.79, 1.53) | 0.59 | 136 | 0.93 (0.35, 2.43) | 0.883 | 78 | 1.32 (0.8, 2.18) | 0.287 | 1194 | 1.1 (0.66, 1.82) | 0.73 |
| 386.2 | Peripheral or central vertigo | 5291 | 17587382 | 2351 | 1.04 (0.83, 1.31) | 0.761 | 555 | 0.94 (0.66, 1.34) | 0.746 | 230 | 0.98 (0.38, 2.54) | 0.97 | 4488 | 1.04 (0.75, 1.45) | 0.812 |
| 386.21 | Central origin vertigo | 354 | 17629004 | 156 | 1.03 (0.24, 4.51) | 0.97 | 38 | 1 (0.85, 1.17) | 0.97 | 17 | 1.11 (0.06, 19.62) | 0.95 | 296 | 0.97 (0.21, 4.42) | 0.97 |
| 386.3 | Labyrinthitis | 597 | 17623617 | 271 | 1.07 (0.47, 2.42) | 0.877 | 62 | 0.93 (0.2, 4.26) | 0.935 | 24 | 0.91 (0.07, 11.49) | 0.949 | 499 | 0.95 (0.29, 3.1) | 0.934 |
| 386.9 | Dizziness and giddiness (Light-headedness and vertigo) | 1060 | 17624097 | 414 | **0.81 (0.71, 0.93)** | **0.003** | 124 | 0.93 (0.46, 1.9) | 0.861 | 39 | 0.74 (0.45, 1.21) | 0.23 | 904 | 1.08 (0.7, 1.68) | 0.739 |
| 388 | Other disorders of ear | 5263 | 17569798 | 2286 | 0.99 (0.65, 1.51) | 0.959 | 581 | 1 (0.96, 1.04) | 0.97 | 211 | 0.9 (0.57, 1.4) | 0.644 | 4460 | 1.06 (0.81, 1.37) | 0.697 |
| 389 | Hearing loss | 43238 | 17166114 | 18753 | 0.99 (0.88, 1.12) | 0.903 | 4773 | 1 (0.88, 1.14) | 0.97 | 1917 | 1 (0.91, 1.11) | 0.97 | 36625 | **1.06 (1.01, 1.11)** | **0.009** |
| 389.1 | Sensorineural hearing loss | 9172 | 17538769 | 3939 | 0.99 (0.78, 1.26) | 0.92 | 1038 | 1.02 (0.66, 1.56) | 0.949 | 418 | 1.02 (0.52, 2) | 0.959 | 7771 | 1.05 (0.89, 1.23) | 0.611 |
| 389.2 | Conductive hearing loss | 2123 | 17613427 | 918 | 1.04 (0.72, 1.52) | 0.833 | 261 | 1.11 (0.77, 1.6) | 0.575 | 106 | 1.15 (0.64, 2.05) | 0.656 | 1780 | 0.96 (0.55, 1.66) | 0.883 |
| 389.3 | Degenerative and vascular disorders of ear | 22354 | 17419947 | 9749 | 0.99 (0.79, 1.24) | 0.955 | 2404 | 1 (0.96, 1.04) | 0.97 | 990 | 1.01 (0.61, 1.69) | 0.959 | 18942 | **1.08 (1.02, 1.15)** | **0.012** |
| 389.4 | Tinnitus | 6854 | 17566053 | 2984 | 1.03 (0.85, 1.24) | 0.802 | 792 | 1.05 (0.83, 1.34) | 0.698 | 322 | 1.07 (0.76, 1.52) | 0.7 | 5853 | 1.09 (0.98, 1.22) | 0.106 |
| 389.5 | Disorders of acoustic nerve | 776 | 17618474 | 337 | 1 (0.89, 1.13) | 0.97 | 86 | 0.97 (0.29, 3.28) | 0.97 | 34 | 0.97 (0.25, 3.83) | 0.97 | 673 | 1.22 (0.73, 2.04) | 0.462 |
| **Circulatory System** | | | | | | | | | | | | | | | |
| 394 | Rheumatic disease of the heart valves | 8422 | 17577658 | 3833 | 1.08 (1, 1.17) | 0.052 | 900 | 1.01 (0.54, 1.89) | 0.97 | 349 | 0.97 (0.46, 2.06) | 0.945 | 7036 | 0.96 (0.78, 1.19) | 0.731 |
| 394.2 | Mitral valve disease | 3861 | 17605677 | 1761 | 1.07 (0.88, 1.31) | 0.487 | 386 | 0.94 (0.56, 1.58) | 0.814 | 167 | 1 (0.87, 1.16) | 0.97 | 3213 | 0.94 (0.68, 1.29) | 0.711 |
| 394.3 | Aortic valve disease | 5049 | 17602080 | 2298 | 1.09 (0.99, 1.21) | 0.08 | 575 | 1.1 (0.87, 1.38) | 0.431 | 197 | 0.93 (0.5, 1.72) | 0.823 | 4219 | 0.96 (0.69, 1.35) | 0.843 |
| 395 | Heart valve disorders | 7798 | 17584882 | 3500 | 1.05 (0.92, 1.2) | 0.474 | 829 | 0.99 (0.71, 1.38) | 0.97 | 328 | 0.97 (0.44, 2.14) | 0.947 | 6527 | 0.97 (0.7, 1.36) | 0.883 |
| 395.1 | Nonrheumatic mitral valve disorders | 3999 | 17606578 | 1819 | 1.07 (0.89, 1.3) | 0.477 | 398 | 0.93 (0.59, 1.49) | 0.784 | 181 | 1.05 (0.41, 2.72) | 0.924 | 3327 | 0.94 (0.69, 1.27) | 0.691 |
| 395.2 | Nonrheumatic aortic valve disorders | 1474 | 17624111 | 646 | 1.02 (0.49, 2.15) | 0.959 | 171 | 1.09 (0.54, 2.21) | 0.814 | 62 | 0.98 (0.3, 3.18) | 0.97 | 1235 | 0.98 (0.46, 2.1) | 0.97 |
| 395.3 | Nonrheumatic tricuspid valve disorders | 300 | 17629788 | 130 | 0.94 (0.24, 3.61) | 0.933 | 29 | 0.82 (0.17, 3.97) | 0.82 | 10 | 0.71 (0.07, 7.14) | 0.781 | 250 | 0.94 (0.12, 7.27) | 0.958 |
| 395.4 | Nonrheumatic pulmonary valve disorders | 177 | 17630077 | 72 | 0.97 (0.29, 3.3) | 0.97 | 23 | 1.08 (0.04, 32.53) | 0.968 | 11 | 1.36 (0.11, 17.11) | 0.824 | 156 | 1.32 (0.31, 5.7) | 0.721 |
| 395.6 | Heart valve replaced | 3668 | 17612845 | 1640 | 1.06 (0.87, 1.29) | 0.575 | 429 | 1.12 (0.87, 1.43) | 0.382 | 145 | 0.93 (0.39, 2.22) | 0.881 | 3059 | 0.95 (0.68, 1.34) | 0.796 |
| 396 | Abnormal heart sounds | 1612 | 17619851 | 702 | 1.02 (0.55, 1.92) | 0.949 | 185 | 0.96 (0.41, 2.27) | 0.935 | 67 | 0.91 (0.27, 2.99) | 0.881 | 1366 | 1.01 (0.64, 1.58) | 0.97 |
| 401 | Hypertension | 11762 | 17493925 | 5080 | 0.98 (0.79, 1.23) | 0.883 | 1262 | 0.98 (0.69, 1.39) | 0.901 | 545 | 1.04 (0.63, 1.72) | 0.883 | 9910 | 1.02 (0.77, 1.35) | 0.911 |
| 401.1 | Essential hypertension | 3161 | 17542205 | 1353 | 0.97 (0.64, 1.47) | 0.899 | 335 | 0.98 (0.38, 2.51) | 0.966 | 163 | 1.16 (0.73, 1.85) | 0.532 | 2658 | 1.01 (0.5, 2.04) | 0.97 |
| 401.2 | Hypertensive heart and/or renal disease | 279 | 17630544 | 118 | 0.98 (0.43, 2.26) | 0.97 | 34 | 1.14 (0.19, 6.63) | 0.896 | 14 | 1.15 (0.07, 20.01) | 0.928 | 232 | 0.94 (0.16, 5.66) | 0.95 |
| 401.21 | Hypertensive heart disease | 3905 | 17612456 | 1713 | 1 (0.89, 1.13) | 0.97 | 411 | 0.97 (0.56, 1.71) | 0.935 | 172 | 1 (0.92, 1.09) | 0.97 | 3302 | 1.05 (0.78, 1.4) | 0.771 |
| 401.22 | Hypertensive chronic kidney disease | 858 | 17625964 | 346 | 0.88 (0.59, 1.32) | 0.545 | 106 | 1.05 (0.25, 4.38) | 0.949 | 35 | 0.87 (0.15, 5.03) | 0.885 | 737 | 1.14 (0.6, 2.19) | 0.7 |
| 401.3 | Other hypertensive complications | 4445 | 17603444 | 1912 | 0.98 (0.68, 1.41) | 0.92 | 482 | 0.97 (0.53, 1.78) | 0.928 | 205 | 1.03 (0.35, 3.02) | 0.963 | 3739 | 1 (0.83, 1.19) | 0.97 |
| 402 | Elevated blood pressure reading without diagnosis of hypertension | 462 | 17630163 | 176 | **0.79 (0.63, 0.99)** | **0.038** | 54 | 0.92 (0.31, 2.69) | 0.883 | 20 | 0.86 (0.18, 4.03) | 0.861 | 394 | 1.07 (0.41, 2.77) | 0.898 |
| 411 | Ischemic Heart Disease | 54853 | 17147186 | 24172 | 1.03 (0.99, 1.07) | 0.185 | 6091 | 1.04 (0.98, 1.11) | 0.178 | 2423 | 1.02 (0.79, 1.31) | 0.881 | 46223 | 1.02 (0.95, 1.1) | 0.59 |
| 411.1 | Unstable angina (intermediate coronary syndrome) | 8841 | 17563612 | 3841 | 1.02 (0.65, 1.59) | 0.95 | 1048 | 1.11 (0.87, 1.42) | 0.39 | 398 | 1.05 (0.43, 2.57) | 0.929 | 7502 | 1.06 (0.8, 1.41) | 0.678 |
| 411.2 | Myocardial infarction | 25905 | 17411193 | 11511 | 1.06 (0.99, 1.12) | 0.08 | 2954 | **1.1 (1.01, 1.19)** | **0.038** | 1166 | 1.06 (0.83, 1.36) | 0.632 | 21825 | 1.02 (0.82, 1.27) | 0.874 |
| 411.3 | Angina pectoris | 27819 | 17360545 | 12166 | 1.01 (0.87, 1.18) | 0.882 | 3130 | 1.05 (0.94, 1.17) | 0.373 | 1228 | 1.01 (0.63, 1.64) | 0.959 | 23440 | 1.02 (0.87, 1.19) | 0.823 |
| 411.4 | Coronary atherosclerosis | 18006 | 17499608 | 7934 | 1.03 (0.93, 1.14) | 0.598 | 2037 | 1.07 (0.96, 1.2) | 0.204 | 802 | 1.04 (0.7, 1.54) | 0.864 | 15119 | 1 (0.98, 1.02) | 0.97 |
| 411.41 | Aneurysm and dissection of heart | 721 | 17626558 | 309 | 0.94 (0.5, 1.75) | 0.845 | 76 | 0.9 (0.34, 2.39) | 0.84 | 24 | 0.71 (0.28, 1.81) | 0.48 | 613 | 1.06 (0.43, 2.67) | 0.902 |
| 411.8 | Other chronic ischemic heart disease, unspecified | 26663 | 17470723 | 11813 | 1.04 (0.94, 1.14) | 0.476 | 2979 | 1.06 (0.93, 1.22) | 0.398 | 1165 | 1.02 (0.55, 1.89) | 0.958 | 22491 | 1.03 (0.87, 1.22) | 0.739 |
| 411.9 | Other acute and subacute forms of ischemic heart disease | 2173 | 17616845 | 978 | 1.07 (0.8, 1.43) | 0.685 | 237 | 1.03 (0.42, 2.54) | 0.949 | 94 | 1.01 (0.53, 1.94) | 0.97 | 1845 | 1.07 (0.69, 1.66) | 0.784 |
| 414 | Other forms of chronic heart disease | 3471 | 17611092 | 1522 | 1.02 (0.61, 1.71) | 0.935 | 395 | 1.03 (0.41, 2.56) | 0.959 | 144 | 0.94 (0.3, 2.92) | 0.919 | 2909 | 0.97 (0.53, 1.78) | 0.924 |
| 415 | Pulmonary heart disease | 10870 | 17565369 | 5214 | **1.29 (1.23, 1.35)** | **<0.001** | 1265 | **1.23 (1.13, 1.34)** | **<0.001** | 547 | **1.33 (1.18, 1.49)** | **<0.001** | 9068 | 0.95 (0.82, 1.1) | 0.492 |
| 415.11 | Pulmonary embolism and infarction, acute | 1533 | 17612465 | 792 | **1.56 (1.35, 1.79)** | **<0.001** | 183 | **1.43 (1.09, 1.89)** | **0.011** | 77 | 1.49 (0.94, 2.35) | 0.088 | 1262 | 0.88 (0.58, 1.34) | 0.575 |
| 415.2 | Chronic pulmonary heart disease | 1452 | 17626006 | 671 | 1.12 (0.72, 1.74) | 0.627 | 138 | 0.91 (0.27, 3.07) | 0.887 | 76 | 1.24 (0.44, 3.51) | 0.693 | 1225 | 1.02 (0.38, 2.71) | 0.97 |
| 415.21 | Primary pulmonary hypertension | 1037 | 17625448 | 450 | 0.96 (0.44, 2.12) | 0.935 | 105 | 0.89 (0.36, 2.19) | 0.806 | 41 | 0.86 (0.17, 4.25) | 0.864 | 877 | 1.03 (0.32, 3.36) | 0.959 |
| 416 | Cardiomegaly | 337 | 17627958 | 143 | 0.9 (0.31, 2.66) | 0.864 | 31 | 0.78 (0.21, 2.86) | 0.724 | 14 | 0.87 (0.03, 22.72) | 0.941 | 282 | 0.97 (0.28, 3.44) | 0.97 |
| 418 | Nonspecific chest pain | 21599 | 17540307 | 9276 | 1.01 (0.64, 1.58) | 0.97 | 2567 | 1.05 (0.69, 1.59) | 0.825 | 1011 | 1.05 (0.5, 2.21) | 0.901 | 18329 | 1.03 (0.68, 1.57) | 0.885 |
| 418.1 | Precordial pain | 1106 | 17610974 | 471 | 0.95 (0.51, 1.78) | 0.884 | 121 | 0.96 (0.25, 3.66) | 0.954 | 47 | 0.93 (0.17, 4.93) | 0.935 | 940 | 1.06 (0.47, 2.39) | 0.889 |
| 420 | Carditis | 5366 | 17585637 | 2388 | 1.06 (0.91, 1.23) | 0.455 | 608 | 1.04 (0.65, 1.65) | 0.883 | 241 | 1.04 (0.48, 2.26) | 0.935 | 4542 | 1.03 (0.69, 1.52) | 0.906 |
| 420.1 | Myocarditis | 665 | 17624551 | 283 | 0.99 (0.56, 1.75) | 0.97 | 82 | 1.07 (0.24, 4.76) | 0.934 | 28 | 0.93 (0.05, 16.38) | 0.963 | 552 | 0.9 (0.38, 2.11) | 0.814 |
| 420.2 | Pericarditis | 1305 | 17623533 | 589 | 1.08 (0.74, 1.56) | 0.708 | 143 | 1.02 (0.38, 2.75) | 0.97 | 57 | 1.02 (0.43, 2.41) | 0.97 | 1108 | 1.05 (0.5, 2.21) | 0.898 |
| 420.21 | Acute pericarditis | 2187 | 17608619 | 953 | 1.02 (0.54, 1.93) | 0.956 | 241 | 0.97 (0.36, 2.62) | 0.956 | 108 | 1.11 (0.46, 2.65) | 0.834 | 1869 | 1.08 (0.73, 1.61) | 0.708 |
| 420.22 | Chronic pericarditis | 398 | 17628586 | 186 | 1.23 (0.66, 2.28) | 0.531 | 48 | 1.24 (0.37, 4.1) | 0.739 | 21 | 1.36 (0.26, 7.15) | 0.733 | 336 | 1.02 (0.47, 2.2) | 0.97 |
| 420.3 | Endocarditis | 1519 | 17622303 | 710 | 1.16 (0.91, 1.47) | 0.234 | 165 | 1.06 (0.39, 2.89) | 0.915 | 66 | 1.06 (0.16, 6.99) | 0.959 | 1277 | 0.99 (0.76, 1.3) | 0.97 |
| 425 | Cardiomyopathy | 3911 | 17604227 | 1676 | 0.98 (0.72, 1.32) | 0.885 | 439 | 1.01 (0.76, 1.33) | 0.97 | 179 | 1.02 (0.35, 3.03) | 0.97 | 3284 | 0.98 (0.57, 1.71) | 0.958 |
| 425.1 | Primary/intrinsic cardiomyopathies | 3329 | 17608241 | 1440 | 1 (0.98, 1.02) | 0.97 | 385 | 1.05 (0.69, 1.61) | 0.826 | 148 | 1.01 (0.67, 1.52) | 0.97 | 2800 | 0.99 (0.77, 1.29) | 0.97 |
| 425.11 | Hypertrophic obstructive cardiomyopathy | 413 | 17628783 | 178 | 0.95 (0.34, 2.62) | 0.929 | 47 | 0.99 (0.54, 1.82) | 0.97 | 11 | 0.58 (0.18, 1.81) | 0.35 | 344 | 0.94 (0.28, 3.18) | 0.924 |
| 425.12 | Other hypertrophic cardiomyopathy | 466 | 17628820 | 178 | 0.76 (0.57, 1) | 0.053 | 44 | 0.73 (0.39, 1.35) | 0.319 | 23 | 0.96 (0.12, 7.38) | 0.97 | 387 | 0.91 (0.3, 2.76) | 0.883 |
| 425.8 | Other cardiomyopathy | 140 | 17630746 | 62 | 0.92 (0.24, 3.5) | 0.91 | 9 | 0.53 (0.19, 1.47) | 0.226 | 6 | 0.89 (0.01, 58.73) | 0.959 | 111 | 0.72 (0.37, 1.4) | 0.338 |
| 426 | Cardiac conduction disorders | 13805 | 17552078 | 5968 | 0.98 (0.42, 2.32) | 0.97 | 1512 | 0.99 (0.57, 1.72) | 0.97 | 607 | 0.99 (0.49, 1.97) | 0.97 | 11599 | 0.99 (0.78, 1.27) | 0.97 |
| 426.2 | Atrioventricular [AV] block | 895 | 17626675 | 391 | 1.02 (0.41, 2.51) | 0.97 | 106 | 1.12 (0.36, 3.48) | 0.861 | 38 | 0.98 (0.41, 2.32) | 0.97 | 759 | 1.07 (0.37, 3.04) | 0.911 |
| 426.21 | First degree AV block | 455 | 17628792 | 190 | 0.85 (0.57, 1.28) | 0.455 | 44 | 0.79 (0.38, 1.64) | 0.539 | 12 | 0.53 (0.21, 1.32) | 0.173 | 381 | 0.98 (0.45, 2.16) | 0.97 |
| 426.23 | Second degree AV block | 1304 | 17624825 | 564 | 0.97 (0.52, 1.81) | 0.923 | 134 | 0.92 (0.39, 2.17) | 0.863 | 58 | 0.98 (0.47, 2.08) | 0.97 | 1093 | 0.99 (0.52, 1.86) | 0.97 |
| 426.24 | Atrioventricular block, complete | 2945 | 17617301 | 1278 | 0.98 (0.62, 1.54) | 0.928 | 304 | 0.94 (0.52, 1.71) | 0.861 | 138 | 1.05 (0.39, 2.85) | 0.929 | 2456 | 0.96 (0.6, 1.55) | 0.885 |
| 426.25 | Other heart block | 1227 | 17624851 | 538 | 1.04 (0.2, 5.49) | 0.97 | 140 | 1.07 (0.04, 31.26) | 0.97 | 59 | 1.12 (0.01, 245.83) | 0.97 | 1048 | 1.11 (0.01, 162.9) | 0.97 |
| 426.3 | Bundle branch block | 353 | 17628996 | 156 | 0.99 (0.58, 1.67) | 0.97 | 33 | 0.85 (0.08, 9.3) | 0.901 | 16 | 1.01 (0.73, 1.38) | 0.97 | 303 | 1.16 (0.18, 7.53) | 0.883 |
| 426.31 | Right bundle branch block | 255 | 17630209 | 100 | 0.82 (0.45, 1.52) | 0.549 | 31 | 1.01 (0.53, 1.93) | 0.97 | 10 | 0.81 (0.05, 12.12) | 0.887 | 215 | 1.02 (0.47, 2.22) | 0.97 |
| 426.32 | Left bundle branch block | 522 | 17629054 | 214 | 0.91 (0.51, 1.62) | 0.767 | 61 | 1.05 (0.2, 5.44) | 0.959 | 26 | 1.1 (0.16, 7.48) | 0.929 | 436 | 0.97 (0.24, 3.95) | 0.965 |
| 426.4 | Anomalous atrioventricular excitation | 962 | 17623508 | 386 | 0.89 (0.62, 1.29) | 0.56 | 111 | 0.91 (0.35, 2.39) | 0.86 | 51 | 1.09 (0.24, 5.01) | 0.921 | 808 | 0.94 (0.41, 2.14) | 0.889 |
| 426.9 | Cardiac pacemaker/device in situ | 471 | 17629078 | 204 | 0.94 (0.39, 2.28) | 0.906 | 46 | 0.85 (0.3, 2.4) | 0.771 | 18 | 0.82 (0.11, 6.23) | 0.861 | 392 | 0.94 (0.29, 3.05) | 0.923 |
| 426.91 | Cardiac pacemaker in situ | 9546 | 17584725 | 4152 | 0.99 (0.79, 1.24) | 0.936 | 1040 | 1 (0.88, 1.13) | 0.97 | 415 | 0.98 (0.51, 1.9) | 0.964 | 8012 | 0.99 (0.68, 1.45) | 0.97 |
| 427 | Cardiac dysrhythmias | 32646 | 17377770 | 14073 | 0.99 (0.64, 1.53) | 0.97 | 3653 | 0.99 (0.6, 1.63) | 0.97 | 1460 | 0.99 (0.73, 1.35) | 0.97 | 27414 | 0.98 (0.68, 1.4) | 0.906 |
| 427.1 | Paroxysmal tachycardia, unspecified | 3171 | 17597865 | 1336 | 0.94 (0.75, 1.19) | 0.635 | 342 | 0.91 (0.63, 1.32) | 0.64 | 144 | 0.97 (0.25, 3.76) | 0.97 | 2663 | 0.97 (0.56, 1.68) | 0.923 |
| 427.11 | Paroxysmal supraventricular tachycardia | 9088 | 17542062 | 3889 | 0.97 (0.83, 1.14) | 0.73 | 981 | 0.94 (0.77, 1.14) | 0.533 | 417 | 1 (0.83, 1.22) | 0.97 | 7667 | 1.01 (0.79, 1.28) | 0.97 |
| 427.12 | Paroxysmal ventricular tachycardia | 2922 | 17611909 | 1272 | 1.03 (0.69, 1.54) | 0.885 | 357 | 1.13 (0.84, 1.51) | 0.432 | 130 | 1.03 (0.29, 3.68) | 0.97 | 2451 | 0.97 (0.55, 1.71) | 0.93 |
| 427.2 | Atrial fibrillation and flutter | 889 | 17611028 | 399 | 1.03 (0.27, 3.93) | 0.97 | 86 | 0.9 (0.01, 109.69) | 0.97 | 41 | 1.05 (0.09, 12.34) | 0.97 | 720 | 0.82 (0.05, 14.29) | 0.901 |
| 427.3 | Other specified cardiac dysrhythmias | 3823 | 17608197 | 1672 | 1.03 (0.73, 1.43) | 0.888 | 427 | 1.03 (0.58, 1.83) | 0.919 | 186 | 1.12 (0.72, 1.74) | 0.638 | 3157 | 0.89 (0.77, 1.03) | 0.133 |
| 427.41 | Ventricular fibrillation and flutter | 803 | 17625975 | 370 | 1.09 (0.68, 1.75) | 0.725 | 87 | 1.02 (0.4, 2.63) | 0.97 | 28 | 0.82 (0.21, 3.23) | 0.784 | 674 | 0.99 (0.53, 1.85) | 0.97 |
| 427.42 | Cardiac arrest | 4174 | 17622233 | 1749 | 0.94 (0.46, 1.94) | 0.885 | 471 | 1.01 (0.78, 1.3) | 0.97 | 212 | 1.13 (0.24, 5.24) | 0.886 | 3505 | 0.99 (0.55, 1.78) | 0.97 |
| 427.5 | Arrhythmia (cardiac) NOS | 3345 | 17607661 | 1381 | 0.92 (0.74, 1.14) | 0.432 | 391 | 1.01 (0.65, 1.56) | 0.97 | 147 | 0.95 (0.3, 2.98) | 0.935 | 2791 | 0.94 (0.61, 1.46) | 0.807 |
| 427.6 | Premature beats | 3801 | 17599471 | 1690 | 1.05 (0.75, 1.47) | 0.784 | 411 | 0.98 (0.38, 2.53) | 0.97 | 174 | 1.04 (0.26, 4.15) | 0.956 | 3178 | 0.95 (0.56, 1.6) | 0.857 |
| 427.61 | Supraventricular premature beats | 1811 | 17618105 | 766 | 0.92 (0.7, 1.22) | 0.582 | 178 | 0.83 (0.59, 1.16) | 0.284 | 80 | 0.94 (0.26, 3.31) | 0.924 | 1531 | 1.02 (0.43, 2.45) | 0.962 |
| 427.7 | Tachycardia NOS | 2280 | 17609646 | 987 | 1.03 (0.59, 1.79) | 0.929 | 274 | 1.06 (0.5, 2.25) | 0.883 | 102 | 1.01 (0.72, 1.4) | 0.97 | 1944 | 1.06 (0.62, 1.83) | 0.835 |
| 427.8 | Sinoatrial node dysfunction (Bradycardia) | 2604 | 17617900 | 1179 | 1.09 (0.87, 1.35) | 0.471 | 294 | 1.11 (0.73, 1.67) | 0.64 | 109 | 1 (0.85, 1.18) | 0.97 | 2168 | 0.95 (0.57, 1.59) | 0.866 |
| 427.9 | Palpitations | 6952 | 17594678 | 3003 | 1 (0.98, 1.02) | 0.97 | 772 | 0.94 (0.76, 1.16) | 0.578 | 299 | 0.93 (0.63, 1.38) | 0.741 | 5872 | 0.99 (0.65, 1.51) | 0.97 |
| 428 | Congestive heart failure; nonhypertensive | 25709 | 17497863 | 11362 | 1.02 (0.81, 1.29) | 0.864 | 2775 | 1.01 (0.64, 1.58) | 0.97 | 1132 | 1.01 (0.55, 1.87) | 0.97 | 21537 | 0.98 (0.69, 1.4) | 0.935 |
| 428.1 | Congestive heart failure (CHF) NOS | 8357 | 17595328 | 3721 | 1.06 (0.99, 1.12) | 0.087 | 917 | 1.06 (0.91, 1.22) | 0.48 | 408 | **1.15 (1.02, 1.3)** | **0.019** | 7027 | 1.01 (0.74, 1.37) | 0.952 |
| 428.2 | Heart failure NOS | 23367 | 17510976 | 10310 | 1.02 (0.77, 1.35) | 0.908 | 2529 | 1.01 (0.6, 1.72) | 0.97 | 1023 | 1.01 (0.76, 1.32) | 0.97 | 19551 | 0.98 (0.69, 1.38) | 0.901 |
| 429 | Ill-defined descriptions and complications of heart disease | 895 | 17627824 | 388 | 0.99 (0.73, 1.36) | 0.97 | 105 | 1.03 (0.22, 4.76) | 0.97 | 32 | 0.79 (0.29, 2.21) | 0.671 | 753 | 0.99 (0.5, 1.96) | 0.97 |
| 429.1 | Heart transplant/surgery | 237 | 17630355 | 112 | 1.15 (0.06, 21.42) | 0.93 | 27 | 1.03 (0.21, 5.09) | 0.97 | 6 | 0.59 (0, 1499.15) | 0.903 | 200 | 0.99 (0.62, 1.58) | 0.97 |
| 429.3 | Symptoms involving cardiovascular system | 580 | 17629797 | 243 | 0.94 (0.41, 2.2) | 0.901 | 72 | 1.09 (0.31, 3.77) | 0.903 | 21 | 0.8 (0.16, 4.04) | 0.794 | 487 | 0.98 (0.34, 2.79) | 0.97 |
| 430 | Intracranial hemorrhage | 7796 | 17577866 | 3381 | 0.98 (0.75, 1.28) | 0.898 | 830 | 0.94 (0.73, 1.22) | 0.664 | 329 | 0.93 (0.59, 1.46) | 0.77 | 6546 | 0.98 (0.67, 1.44) | 0.938 |
| 430.1 | Subarachnoid hemorrhage | 2756 | 17607449 | 1180 | 0.97 (0.65, 1.44) | 0.883 | 292 | 0.92 (0.61, 1.4) | 0.714 | 127 | 1.01 (0.73, 1.38) | 0.97 | 2337 | 1.04 (0.63, 1.73) | 0.889 |
| 430.2 | Intracerebral hemorrhage | 4770 | 17605092 | 2069 | 0.98 (0.68, 1.4) | 0.909 | 497 | 0.93 (0.64, 1.36) | 0.732 | 209 | 0.97 (0.28, 3.39) | 0.969 | 3993 | 0.97 (0.62, 1.52) | 0.909 |
| 430.3 | Subdural hemorrhage | 440 | 17629103 | 187 | 0.95 (0.27, 3.27) | 0.935 | 56 | 1.13 (0.24, 5.35) | 0.886 | 11 | 0.55 (0.11, 2.78) | 0.477 | 373 | 1.06 (0.16, 7.19) | 0.958 |
| 433 | Cerebrovascular disease | 37160 | 17363677 | 16364 | 1.02 (0.96, 1.09) | 0.529 | 4022 | 1.01 (0.82, 1.24) | 0.935 | 1673 | 1.04 (0.88, 1.23) | 0.671 | 31155 | 0.99 (0.86, 1.14) | 0.885 |
| 433.1 | Occlusion and stenosis of precerebral arteries | 2798 | 17609544 | 1258 | 1.06 (0.73, 1.54) | 0.767 | 313 | 1.07 (0.5, 2.3) | 0.873 | 112 | 0.94 (0.23, 3.77) | 0.935 | 2340 | 0.97 (0.41, 2.33) | 0.959 |
| 433.11 | Occlusion of cerebral arteries, with cerebral infarction | 950 | 17624900 | 437 | 1.12 (0.86, 1.46) | 0.402 | 104 | 1.08 (0.47, 2.5) | 0.865 | 43 | 1.1 (0.29, 4.08) | 0.898 | 796 | 0.99 (0.5, 1.95) | 0.97 |
| 433.12 | Cerebral atherosclerosis | 293 | 17629591 | 131 | 0.95 (0.18, 5.06) | 0.955 | 24 | 0.71 (0.2, 2.56) | 0.612 | 8 | 0.58 (0.07, 4.9) | 0.626 | 231 | 0.72 (0.4, 1.28) | 0.267 |
| 433.2 | Occlusion of cerebral arteries | 17990 | 17532568 | 7946 | 1.03 (0.97, 1.09) | 0.327 | 1952 | 1.02 (0.84, 1.25) | 0.849 | 832 | 1.08 (0.95, 1.22) | 0.268 | 15060 | 0.98 (0.87, 1.1) | 0.742 |
| 433.21 | Cerebral artery occlusion, with cerebral infarction | 3011 | 17602882 | 1369 | 1.08 (0.9, 1.3) | 0.406 | 316 | 1 (0.96, 1.04) | 0.97 | 134 | 1.05 (0.37, 2.94) | 0.938 | 2504 | 0.94 (0.69, 1.28) | 0.71 |
| 433.3 | Cerebral ischemia | 222 | 17629692 | 94 | 0.99 (0.5, 1.93) | 0.97 | 24 | 0.99 (0.65, 1.52) | 0.97 | 14 | 1.44 (0.23, 9.1) | 0.713 | 188 | 1.04 (0.13, 8.13) | 0.97 |
| 433.31 | Transient cerebral ischemia | 12202 | 17526057 | 5341 | 1 (0.98, 1.02) | 0.97 | 1299 | 0.98 (0.71, 1.34) | 0.906 | 536 | 1 (0.81, 1.23) | 0.97 | 10224 | 0.99 (0.75, 1.3) | 0.929 |
| 433.5 | Cerebral aneurysm | 848 | 17626698 | 347 | 0.91 (0.59, 1.4) | 0.682 | 92 | 0.93 (0.32, 2.75) | 0.909 | 46 | 1.17 (0.39, 3.52) | 0.796 | 716 | 1.01 (0.55, 1.88) | 0.97 |
| 433.8 | Late effects of cerebrovascular disease | 17384 | 17541983 | 7650 | 1.02 (0.9, 1.14) | 0.819 | 1864 | 1 (0.97, 1.04) | 0.97 | 783 | 1.04 (0.79, 1.36) | 0.812 | 14572 | 0.99 (0.8, 1.22) | 0.93 |
| 440 | Atherosclerosis | 10901 | 17554704 | 4890 | **1.1 (1.03, 1.16)** | **0.002** | 1274 | **1.16 (1.06, 1.26)** | **<0.001** | 530 | **1.18 (1.03, 1.36)** | **0.018** | 9205 | 1.04 (0.87, 1.24) | 0.683 |
| 440.1 | Atherosclerosis of renal artery | 190 | 17630110 | 82 | 1.11 (0.25, 4.84) | 0.901 | 27 | 1.43 (0.51, 4.01) | 0.505 | 11 | 1.46 (0.22, 9.61) | 0.71 | 165 | 1.24 (0.29, 5.27) | 0.784 |
| 440.2 | Atherosclerosis of the extremities | 8348 | 17570336 | 3772 | **1.1 (1.03, 1.18)** | **0.004** | 966 | **1.15 (1.03, 1.28)** | **0.012** | 394 | 1.15 (0.94, 1.42) | 0.184 | 7041 | 1.03 (0.79, 1.35) | 0.832 |
| 440.9 | Atherosclerosis of aorta | 292 | 17629056 | 140 | 1.2 (0.72, 2) | 0.493 | 30 | 1.04 (0.17, 6.22) | 0.97 | 12 | 1.02 (0.41, 2.55) | 0.97 | 251 | 1.17 (0.44, 3.1) | 0.767 |
| 441 | Vascular insufficiency of intestine | 1470 | 17626588 | 636 | 0.97 (0.56, 1.66) | 0.91 | 151 | 0.92 (0.43, 1.95) | 0.84 | 61 | 0.92 (0.26, 3.27) | 0.902 | 1245 | 1.05 (0.52, 2.13) | 0.897 |
| 441.1 | Acute vascular insufficiency of intestine | 665 | 17629993 | 279 | 0.89 (0.56, 1.42) | 0.64 | 63 | 0.8 (0.4, 1.61) | 0.549 | 27 | 0.85 (0.13, 5.4) | 0.874 | 566 | 1.09 (0.39, 3) | 0.883 |
| 441.2 | Chronic vascular insufficiency of intestine | 650 | 17629237 | 275 | 0.94 (0.59, 1.5) | 0.817 | 73 | 1.01 (0.65, 1.57) | 0.97 | 26 | 0.88 (0.24, 3.22) | 0.861 | 542 | 0.95 (0.47, 1.95) | 0.906 |
| 442 | Other aneurysm | 7542 | 17592617 | 3323 | 1.01 (0.67, 1.54) | 0.962 | 805 | 0.99 (0.55, 1.77) | 0.97 | 324 | 0.98 (0.37, 2.63) | 0.97 | 6325 | 0.99 (0.57, 1.71) | 0.97 |
| 442.1 | Aortic aneurysm | 2720 | 17620190 | 1199 | 1.01 (0.57, 1.8) | 0.964 | 301 | 1.03 (0.43, 2.43) | 0.959 | 109 | 0.92 (0.37, 2.28) | 0.86 | 2310 | 1.07 (0.77, 1.5) | 0.703 |
| 442.11 | Abdominal aortic aneurysm | 4104 | 17611430 | 1811 | 1.02 (0.77, 1.35) | 0.897 | 449 | 1.04 (0.7, 1.55) | 0.861 | 178 | 1 (0.86, 1.17) | 0.97 | 3416 | 0.95 (0.78, 1.16) | 0.638 |
| 442.2 | Aneurysm of iliac artery | 326 | 17630660 | 121 | **0.76 (0.64, 0.9)** | **0.001** | 39 | 1 (0.95, 1.06) | 0.97 | 17 | 1.07 (0.22, 5.19) | 0.94 | 258 | **0.72 (0.6, 0.87)** | **<0.001** |
| 442.3 | Aneurysm of artery of lower extremity | 502 | 17629006 | 221 | 1.01 (0.58, 1.77) | 0.97 | 53 | 0.98 (0.32, 2.95) | 0.97 | 23 | 1.05 (0.12, 8.9) | 0.97 | 423 | 1.02 (0.45, 2.28) | 0.97 |
| 442.4 | Arterial dissection | 174 | 17630904 | 66 | 0.79 (0.47, 1.31) | 0.364 | 21 | 0.92 (0.13, 6.56) | 0.937 | 7 | 0.79 (0.06, 10.59) | 0.868 | 144 | 0.87 (0.26, 2.92) | 0.836 |
| 442.8 | Aneurysm of other specified artery | 891 | 17626918 | 407 | 1.05 (0.65, 1.7) | 0.859 | 84 | 0.85 (0.56, 1.3) | 0.463 | 33 | 0.83 (0.37, 1.87) | 0.671 | 752 | 1.02 (0.42, 2.49) | 0.97 |
| 443 | Peripheral vascular disease | 13791 | 17546796 | 6173 | **1.06 (1.01, 1.12)** | **0.029** | 1515 | 1.05 (0.89, 1.23) | 0.6 | 626 | 1.07 (0.85, 1.35) | 0.588 | 11650 | 1.03 (0.9, 1.19) | 0.644 |
| 443.1 | Raynaud's syndrome | 1003 | 17620189 | 452 | 1.06 (0.56, 2.04) | 0.861 | 107 | 0.95 (0.28, 3.25) | 0.935 | 40 | 0.9 (0.16, 5.05) | 0.912 | 835 | 0.92 (0.46, 1.84) | 0.817 |
| 443.7 | Peripheral angiopathy in diseases classified elsewhere | 3677 | 17611414 | 1621 | 1.05 (0.9, 1.24) | 0.53 | 435 | 1.13 (0.96, 1.33) | 0.128 | 177 | 1.14 (0.83, 1.58) | 0.424 | 3166 | **1.18 (1.05, 1.31)** | **0.004** |
| 443.8 | Other specified peripheral vascular diseases | 219 | 17629755 | 98 | 1.16 (0.27, 4.96) | 0.85 | 29 | 1.26 (0.18, 8.64) | 0.826 | 11 | 1.22 (0.03, 53.77) | 0.924 | 186 | 1.03 (0.27, 3.91) | 0.97 |
| 443.9 | Peripheral vascular disease, unspecified | 9899 | 17576545 | 4478 | **1.08 (1.01, 1.14)** | **0.02** | 1050 | 1.02 (0.73, 1.43) | 0.91 | 449 | 1.08 (0.82, 1.42) | 0.62 | 8321 | 1.01 (0.74, 1.36) | 0.97 |
| 444 | Arterial embolism and thrombosis | 2390 | 17614619 | 1120 | **1.25 (1.11, 1.4)** | **<0.001** | 288 | **1.27 (1.03, 1.57)** | **0.025** | 133 | **1.46 (1.12, 1.9)** | **0.005** | 2009 | 1 (0.92, 1.08) | 0.97 |
| 444.1 | Arterial embolism and thrombosis of lower extremity artery | 1286 | 17623872 | 599 | **1.25 (1.01, 1.54)** | **0.042** | 157 | 1.3 (0.9, 1.89) | 0.163 | 77 | **1.59 (1.05, 2.38)** | **0.027** | 1077 | 0.98 (0.35, 2.76) | 0.97 |
| 446 | Polyarteritis nodosa and allied conditions | 2763 | 17607320 | 1251 | 1.07 (0.83, 1.38) | 0.609 | 287 | 0.97 (0.38, 2.5) | 0.955 | 117 | 0.98 (0.37, 2.6) | 0.97 | 2342 | 1.05 (0.64, 1.75) | 0.849 |
| 446.3 | Hypersensitivity angiitis | 156 | 17630772 | 72 | 1.01 (0.66, 1.55) | 0.97 | 9 | 0.48 (0.03, 6.7) | 0.597 | 7 | 0.94 (0.05, 18.63) | 0.97 | 122 | 0.67 (0.16, 2.79) | 0.59 |
| 446.4 | Wegener's granulomatosis | 333 | 17629183 | 148 | 1.06 (0.33, 3.38) | 0.923 | 38 | 1.05 (0.1, 10.58) | 0.97 | 15 | 1.04 (0.15, 7.14) | 0.97 | 281 | 1.01 (0.72, 1.42) | 0.97 |
| 446.5 | Giant cell arteritis | 1661 | 17617833 | 759 | 1.07 (0.79, 1.45) | 0.67 | 171 | 0.99 (0.51, 1.92) | 0.97 | 62 | 0.87 (0.35, 2.15) | 0.777 | 1414 | 1.1 (0.76, 1.59) | 0.624 |
| 446.6 | Polyarteritis nodosa | 257 | 17629464 | 122 | 1.32 (0.72, 2.4) | 0.371 | 27 | 1.13 (0.1, 12.37) | 0.929 | 20 | 2.1 (0.99, 4.45) | 0.052 | 229 | 1.54 (0.69, 3.46) | 0.3 |
| 446.8 | Thrombotic microangiopathy | 144 | 17630636 | 57 | 0.89 (0.12, 6.88) | 0.92 | 20 | 1.14 (0.06, 21.82) | 0.936 | 6 | 0.88 (0, 337.21) | 0.97 | 120 | 0.91 (0.04, 19.74) | 0.955 |
| 446.9 | Arteritis NOS | 234 | 17629802 | 101 | 0.92 (0.25, 3.39) | 0.907 | 23 | 0.83 (0.12, 5.94) | 0.864 | 6 | 0.54 (0.06, 4.58) | 0.582 | 203 | 1.24 (0.38, 4.08) | 0.732 |
| 447 | Other disorders of arteries and arterioles | 742 | 17626167 | 322 | 0.99 (0.55, 1.76) | 0.97 | 77 | 0.89 (0.19, 4.21) | 0.895 | 33 | 0.97 (0.22, 4.31) | 0.97 | 621 | 0.95 (0.19, 4.68) | 0.954 |
| 448 | Disease of capillaries | 1564 | 17618864 | 691 | 1.04 (0.6, 1.82) | 0.898 | 169 | 0.96 (0.3, 3.06) | 0.954 | 70 | 1.01 (0.67, 1.51) | 0.97 | 1314 | 0.97 (0.4, 2.4) | 0.959 |
| 450 | Noninfectious disorders of lymphatic channels | 3018 | 17611647 | 1261 | 0.94 (0.77, 1.15) | 0.539 | 336 | 0.95 (0.55, 1.64) | 0.861 | 137 | 0.97 (0.28, 3.35) | 0.97 | 2522 | 0.94 (0.69, 1.29) | 0.723 |
| 451 | Phlebitis and thrombophlebitis | 16748 | 17479709 | 8053 | **1.36 (1.31, 1.41)** | **<0.001** | 2092 | **1.38 (1.3, 1.45)** | **<0.001** | 941 | **1.55 (1.44, 1.67)** | **<0.001** | 14036 | 0.97 (0.84, 1.13) | 0.723 |
| 451.2 | Phlebitis and thrombophlebitis of lower extremities | 15650 | 17489528 | 7573 | **1.38 (1.33, 1.43)** | **<0.001** | 1944 | **1.38 (1.31, 1.46)** | **<0.001** | 890 | **1.59 (1.48, 1.71)** | **<0.001** | 13108 | 0.97 (0.84, 1.12) | 0.685 |
| 452 | Other venous embolism and thrombosis | 4275 | 17607194 | 2076 | **1.42 (1.32, 1.52)** | **<0.001** | 564 | **1.49 (1.34, 1.66)** | **<0.001** | 246 | **1.64 (1.41, 1.9)** | **<0.001** | 3601 | 1 (0.89, 1.11) | 0.97 |
| 452.8 | Postphlebitic syndrome | 341 | 17629425 | 172 | **1.74 (1.38, 2.2)** | **<0.001** | 48 | **1.81 (1.26, 2.61)** | **0.001** | 27 | **2.61 (1.76, 3.86)** | **<0.001** | 295 | 1.18 (0.53, 2.63) | 0.699 |
| 454 | Varicose veins | 16500 | 17381971 | 7512 | **1.11 (1.03, 1.2)** | **0.005** | 1806 | 1.04 (0.69, 1.56) | 0.864 | 760 | 1.09 (0.74, 1.61) | 0.664 | 13840 | 0.98 (0.69, 1.38) | 0.9 |
| 454.1 | Varicose veins of lower extremity | 12511 | 17415172 | 5728 | **1.14 (1.01, 1.28)** | **0.034** | 1394 | 1.07 (0.69, 1.66) | 0.767 | 581 | 1.12 (0.62, 2) | 0.724 | 10484 | 0.97 (0.59, 1.58) | 0.911 |
| 454.11 | Varicose veins of lower extremity, symptomtic | 3478 | 17601786 | 1558 | 1.05 (0.83, 1.32) | 0.71 | 371 | 1 (0.86, 1.17) | 0.97 | 147 | 0.98 (0.4, 2.43) | 0.97 | 2884 | 0.93 (0.74, 1.15) | 0.498 |
| 455 | Hemorrhoids | 9001 | 17523962 | 3782 | **0.92 (0.85, 0.99)** | **0.027** | 946 | **0.87 (0.78, 0.98)** | **0.019** | 370 | 0.86 (0.69, 1.08) | 0.201 | 7585 | 0.99 (0.67, 1.46) | 0.97 |
| 456 | Chronic venous insufficiency [CVI] | 925 | 17626709 | 452 | **1.37 (1.18, 1.58)** | **<0.001** | 105 | 1.25 (0.88, 1.79) | 0.212 | 55 | **1.64 (1.18, 2.26)** | **0.003** | 770 | 0.94 (0.48, 1.84) | 0.864 |
| 458 | Hypotension | 5806 | 17603019 | 2590 | 1.04 (0.82, 1.32) | 0.767 | 621 | 0.99 (0.71, 1.38) | 0.97 | 242 | 0.96 (0.39, 2.33) | 0.929 | 4863 | 0.98 (0.6, 1.59) | 0.934 |
| 458.1 | Orthostatic hypotension | 2573 | 17620585 | 1104 | 0.97 (0.72, 1.31) | 0.849 | 291 | 1.02 (0.54, 1.95) | 0.949 | 103 | 0.89 (0.54, 1.47) | 0.671 | 2144 | 0.95 (0.72, 1.25) | 0.724 |
| 458.2 | Iatrogenic hypotension | 250 | 17630698 | 123 | 1.14 (0.71, 1.82) | 0.609 | 19 | 0.71 (0.37, 1.35) | 0.297 | 6 | 0.55 (0.19, 1.57) | 0.267 | 215 | 1.17 (0.59, 2.34) | 0.664 |
| 458.9 | Hypotension NOS | 3106 | 17614800 | 1408 | 1.08 (0.83, 1.41) | 0.575 | 329 | 1 (0.86, 1.18) | 0.97 | 139 | 1.05 (0.26, 4.2) | 0.95 | 2604 | 0.98 (0.43, 2.26) | 0.97 |
| 459 | Other disorders of circulatory system | 2555 | 17616713 | 1168 | **1.2 (1.08, 1.34)** | **<0.001** | 327 | **1.25 (1.05, 1.48)** | **0.012** | 132 | 1.29 (0.97, 1.71) | 0.083 | 2184 | 1.08 (0.79, 1.48) | 0.649 |
| 459.1 | Hemorrhage NOS | 139 | 17630087 | 52 | 0.71 (0.34, 1.46) | 0.353 | 12 | 0.62 (0.14, 2.7) | 0.531 | 5 | 0.65 (0.02, 19.25) | 0.816 | 126 | 1.8 (0.6, 5.4) | 0.3 |
| 459.9 | Circulatory disease NEC | 2174 | 17618930 | 1009 | **1.26 (1.14, 1.41)** | **<0.001** | 283 | **1.3 (1.09, 1.55)** | **0.003** | 119 | **1.41 (1.08, 1.83)** | **0.01** | 1845 | 1.02 (0.48, 2.19) | 0.956 |
| **Respiratory** | | | | | | | | | | | | | | | |
| 464 | Acute sinusitis | 5910 | 17579957 | 2501 | 0.99 (0.67, 1.47) | 0.97 | 729 | 1 (0.93, 1.08) | 0.97 | 241 | 0.87 (0.54, 1.4) | 0.575 | 4990 | 0.96 (0.61, 1.52) | 0.883 |
| 465 | Acute upper respiratory infections of multiple or unspecified sites | 22021 | 17339346 | 9365 | 1.02 (0.85, 1.22) | 0.84 | 2725 | 1 (0.82, 1.22) | 0.97 | 1017 | 0.99 (0.5, 1.94) | 0.97 | 18749 | 1 (0.94, 1.06) | 0.97 |
| 465.2 | Acute pharyngitis | 3654 | 17573557 | 1529 | 0.99 (0.55, 1.76) | 0.97 | 459 | 1 (0.89, 1.13) | 0.97 | 165 | 0.95 (0.35, 2.62) | 0.935 | 3117 | 1.02 (0.51, 2.04) | 0.963 |
| 465.4 | Acute laryngitis and tracheitis | 1843 | 17605720 | 812 | 1.05 (0.67, 1.64) | 0.849 | 209 | 0.95 (0.43, 2.07) | 0.901 | 75 | 0.89 (0.34, 2.34) | 0.82 | 1566 | 1.01 (0.57, 1.81) | 0.97 |
| 470 | Septal Deviations/Turbinate Hypertrophy | 9299 | 17482561 | 3972 | 1.02 (0.82, 1.27) | 0.864 | 1158 | 1.09 (0.96, 1.24) | 0.18 | 465 | 1.13 (0.93, 1.36) | 0.212 | 7927 | 1.05 (0.9, 1.23) | 0.544 |
| 471 | Nasal polyps | 2471 | 17599024 | 1059 | 1 (0.96, 1.04) | 0.97 | 294 | 1.06 (0.56, 2.03) | 0.86 | 118 | 1.08 (0.39, 3) | 0.896 | 2048 | 0.9 (0.69, 1.17) | 0.435 |
| 472 | Chronic pharyngitis and nasopharyngitis | 1553 | 17614593 | 665 | 0.97 (0.52, 1.8) | 0.934 | 168 | 0.9 (0.51, 1.57) | 0.725 | 66 | 0.91 (0.27, 3.07) | 0.885 | 1292 | 0.9 (0.62, 1.32) | 0.607 |
| 473 | Diseases of the larynx and vocal cords | 10107 | 17513914 | 4309 | 0.97 (0.78, 1.21) | 0.806 | 1110 | 0.94 (0.71, 1.25) | 0.699 | 463 | 1 (0.88, 1.13) | 0.97 | 8458 | 0.95 (0.78, 1.15) | 0.606 |
| 473.1 | Chronic laryngitis | 1934 | 17601125 | 835 | 0.96 (0.59, 1.57) | 0.883 | 182 | 0.81 (0.61, 1.09) | 0.167 | 94 | 1.05 (0.27, 4.1) | 0.95 | 1635 | 1.02 (0.44, 2.39) | 0.959 |
| 473.3 | Paralysis/spasm of vocal cords or larynx | 1580 | 17620221 | 659 | 0.93 (0.56, 1.57) | 0.805 | 164 | 0.9 (0.39, 2.08) | 0.814 | 87 | 1.19 (0.49, 2.89) | 0.706 | 1327 | 0.98 (0.39, 2.49) | 0.97 |
| 473.4 | Voice disturbance | 4719 | 17581560 | 1979 | 0.95 (0.65, 1.39) | 0.805 | 537 | 0.96 (0.44, 2.1) | 0.92 | 214 | 0.97 (0.27, 3.51) | 0.97 | 3958 | 0.95 (0.53, 1.71) | 0.883 |
| 474 | Acute and chronic tonsillitis | 41427 | 16817602 | 18276 | **1.1 (1.07, 1.13)** | **<0.001** | 4898 | 0.97 (0.88, 1.08) | 0.619 | 1974 | 1.05 (0.92, 1.2) | 0.46 | 35152 | 0.97 (0.9, 1.05) | 0.48 |
| 474.1 | Acute tonsillitis | 18161 | 17350104 | 7800 | 1.04 (0.95, 1.13) | 0.437 | 2203 | 0.98 (0.76, 1.26) | 0.866 | 845 | 1 (0.91, 1.09) | 0.97 | 15469 | 1 (0.98, 1.02) | 0.97 |
| 474.2 | Chronic tonsillitis and adenoiditis | 27077 | 17030480 | 12170 | **1.14 (1.1, 1.17)** | **<0.001** | 3088 | 0.95 (0.87, 1.03) | 0.224 | 1305 | 1.08 (0.95, 1.22) | 0.249 | 22923 | 0.96 (0.89, 1.03) | 0.268 |
| 475 | Chronic sinusitis | 5282 | 17568839 | 2261 | 1 (0.88, 1.15) | 0.97 | 636 | 1.05 (0.72, 1.54) | 0.812 | 235 | 0.99 (0.6, 1.64) | 0.97 | 4452 | 0.98 (0.57, 1.69) | 0.959 |
| 476 | Allergic rhinitis | 7408 | 17530894 | 3216 | 1.06 (0.96, 1.17) | 0.267 | 889 | 1.02 (0.68, 1.53) | 0.918 | 372 | 1.12 (0.89, 1.41) | 0.332 | 6284 | 1 (0.87, 1.14) | 0.97 |
| 477 | Epistaxis or throat hemorrhage | 12337 | 17506794 | 5142 | **0.9 (0.86, 0.94)** | **<0.001** | 1284 | **0.87 (0.81, 0.94)** | **<0.001** | 518 | 0.88 (0.75, 1.03) | 0.106 | 10409 | 1.01 (0.76, 1.35) | 0.939 |
| 478 | Throat pain | 576 | 17628858 | 238 | 1 (0.9, 1.11) | 0.97 | 75 | 1.1 (0.24, 5.13) | 0.909 | 33 | 1.27 (0.25, 6.42) | 0.788 | 502 | 1.21 (0.48, 3.05) | 0.695 |
| 479 | Other upper respiratory disease | 3932 | 17579026 | 1686 | 1.02 (0.52, 1.99) | 0.965 | 474 | 1.06 (0.53, 2.12) | 0.879 | 192 | 1.1 (0.43, 2.83) | 0.859 | 3335 | 1.02 (0.46, 2.29) | 0.958 |
| 480 | Pneumonia | 76134 | 16979131 | 32949 | 1 (0.88, 1.15) | 0.962 | 8590 | 1 (0.97, 1.03) | 0.97 | 3392 | 0.99 (0.75, 1.31) | 0.97 | 64183 | 1 (0.96, 1.04) | 0.97 |
| 480.1 | Bacterial pneumonia | 20705 | 17539641 | 9060 | 1.02 (0.89, 1.17) | 0.818 | 2310 | 1.02 (0.79, 1.32) | 0.886 | 913 | 1 (0.89, 1.13) | 0.97 | 17454 | 1.01 (0.82, 1.26) | 0.912 |
| 480.11 | Pneumococcal pneumonia | 2318 | 17607708 | 996 | 1 (0.87, 1.14) | 0.97 | 262 | 1 (0.85, 1.18) | 0.97 | 113 | 1.09 (0.44, 2.73) | 0.863 | 1942 | 0.96 (0.55, 1.7) | 0.906 |
| 480.12 | Pseudomonal pneumonia | 552 | 17629228 | 245 | 1.04 (0.47, 2.34) | 0.925 | 62 | 1 (0.81, 1.25) | 0.97 | 22 | 0.9 (0.13, 6.29) | 0.923 | 468 | 1.03 (0.27, 3.97) | 0.964 |
| 480.2 | Viral pneumonia | 2162 | 17596616 | 944 | 1.06 (0.45, 2.48) | 0.903 | 252 | 0.95 (0.17, 5.19) | 0.959 | 104 | 1.04 (0.15, 7.49) | 0.97 | 1852 | 1.06 (0.3, 3.76) | 0.938 |
| 480.3 | Pneumonia due to fungus (mycoses) | 274 | 17630488 | 114 | 0.96 (0.21, 4.43) | 0.965 | 33 | 1.05 (0.11, 9.71) | 0.97 | 15 | 1.22 (0.16, 9.29) | 0.861 | 234 | 1.08 (0.22, 5.25) | 0.934 |
| 480.5 | Bronchopneumonia and lung abscess | 2931 | 17593468 | 1296 | 1.04 (0.67, 1.61) | 0.883 | 318 | 0.97 (0.4, 2.39) | 0.959 | 131 | 1.01 (0.63, 1.61) | 0.97 | 2441 | 0.93 (0.66, 1.3) | 0.684 |
| 481 | Influenza | 4871 | 17597860 | 2042 | 0.97 (0.68, 1.38) | 0.861 | 584 | 0.98 (0.42, 2.3) | 0.968 | 214 | 0.93 (0.4, 2.19) | 0.885 | 4188 | 1.1 (0.87, 1.4) | 0.432 |
| 483 | Acute bronchitis and bronchiolitis | 15140 | 17323240 | 6562 | 1.04 (0.91, 1.18) | 0.588 | 1751 | 0.92 (0.8, 1.07) | 0.297 | 676 | 0.95 (0.62, 1.43) | 0.807 | 12825 | 0.97 (0.78, 1.19) | 0.767 |
| 495 | Asthma | 31106 | 17238738 | 13520 | 1.03 (0.98, 1.08) | 0.212 | 3516 | 0.95 (0.88, 1.02) | 0.18 | 1435 | 1.01 (0.69, 1.47) | 0.97 | 26344 | 0.99 (0.78, 1.27) | 0.97 |
| 496 | Chronic airway obstruction | 30336 | 17434570 | 13446 | 1.02 (0.74, 1.41) | 0.901 | 3170 | 0.97 (0.57, 1.65) | 0.912 | 1307 | 0.98 (0.42, 2.32) | 0.97 | 25408 | 0.98 (0.59, 1.63) | 0.946 |
| 496.1 | Emphysema | 2500 | 17612931 | 1108 | 1.02 (0.37, 2.85) | 0.97 | 267 | 0.98 (0.34, 2.81) | 0.97 | 101 | 0.92 (0.01, 60.76) | 0.97 | 2097 | 0.98 (0.46, 2.12) | 0.97 |
| 496.2 | Chronic bronchitis | 681 | 17623715 | 289 | 0.96 (0.3, 3.1) | 0.956 | 74 | 0.99 (0.51, 1.9) | 0.97 | 35 | 1.15 (0.18, 7.35) | 0.892 | 566 | 0.94 (0.32, 2.73) | 0.911 |
| 496.21 | Obstructive chronic bronchitis | 12484 | 17570623 | 5526 | 1.01 (0.73, 1.4) | 0.935 | 1250 | 0.93 (0.72, 1.21) | 0.597 | 568 | 1.04 (0.51, 2.11) | 0.924 | 10484 | 1 (0.87, 1.16) | 0.97 |
| 499 | Cystic fibrosis | 222 | 17627125 | 91 | 0.81 (0.25, 2.62) | 0.734 | 18 | 0.54 (0.12, 2.48) | 0.438 | 4 | 0.32 (0.01, 7.51) | 0.487 | 189 | 1.01 (0.59, 1.75) | 0.97 |
| 500 | Lung disease due to external agents | 1287 | 17624337 | 564 | 0.99 (0.51, 1.92) | 0.97 | 133 | 0.89 (0.49, 1.62) | 0.724 | 48 | 0.81 (0.36, 1.83) | 0.626 | 1097 | 1.08 (0.6, 1.94) | 0.817 |
| 500.1 | Extrinsic allergic alveolitis | 202 | 17630310 | 86 | 0.92 (0.2, 4.27) | 0.923 | 17 | 0.68 (0.17, 2.71) | 0.597 | 10 | 1.02 (0.44, 2.38) | 0.97 | 168 | 0.91 (0.13, 6.33) | 0.93 |
| 501 | Pneumonitis due to inhalation of food or vomitus | 2415 | 17626248 | 1092 | 1.05 (0.82, 1.33) | 0.723 | 248 | 0.94 (0.59, 1.49) | 0.806 | 93 | 0.88 (0.52, 1.48) | 0.635 | 2042 | 1.04 (0.67, 1.61) | 0.885 |
| 502 | Postinflammatory pulmonary fibrosis | 1529 | 17621362 | 710 | 1.11 (0.82, 1.5) | 0.499 | 156 | 0.96 (0.28, 3.25) | 0.949 | 59 | 0.9 (0.21, 3.91) | 0.898 | 1282 | 0.98 (0.3, 3.13) | 0.97 |
| 503 | Pulmonary congestion and hypostasis | 143 | 17631037 | 61 | 0.97 (0.27, 3.53) | 0.97 | 15 | 0.96 (0.16, 5.92) | 0.97 | 8 | 1.26 (0.06, 26.14) | 0.889 | 120 | 1 (0.9, 1.11) | 0.97 |
| 504 | Other alveolar and parietoalveolar pneumonopathy | 2677 | 17618453 | 1221 | 1.09 (0.93, 1.27) | 0.307 | 276 | 0.96 (0.51, 1.82) | 0.915 | 118 | 1.03 (0.3, 3.56) | 0.97 | 2255 | 1 (0.81, 1.25) | 0.97 |
| 506 | Empyema and pneumothorax | 5850 | 17580511 | 2520 | 0.98 (0.69, 1.4) | 0.934 | 653 | 0.97 (0.57, 1.66) | 0.929 | 253 | 0.95 (0.46, 1.96) | 0.901 | 4996 | 1.09 (0.91, 1.29) | 0.364 |
| 507 | Pleurisy; pleural effusion | 4556 | 17591981 | 2031 | 1.06 (0.85, 1.33) | 0.612 | 523 | 1.05 (0.57, 1.92) | 0.883 | 190 | 0.96 (0.3, 3.1) | 0.949 | 3849 | 1.02 (0.5, 2.06) | 0.97 |
| 508 | Pulmonary collapse; interstitial and compensatory emphysema | 2022 | 17615412 | 868 | 0.99 (0.54, 1.81) | 0.97 | 235 | 1 (0.84, 1.2) | 0.97 | 83 | 0.9 (0.05, 15.19) | 0.947 | 1711 | 1.02 (0.49, 2.13) | 0.97 |
| 509 | Respiratory failure, insufficiency, arrest | 25968 | 17557093 | 11337 | 1.01 (0.89, 1.14) | 0.901 | 2870 | 1 (0.95, 1.06) | 0.97 | 1138 | 0.99 (0.67, 1.47) | 0.963 | 21933 | 1.03 (0.94, 1.12) | 0.576 |
| 509.1 | Respiratory failure | 23903 | 17563206 | 10422 | 1.01 (0.86, 1.18) | 0.949 | 2630 | 0.99 (0.75, 1.32) | 0.965 | 1055 | 0.99 (0.78, 1.27) | 0.97 | 20228 | 1.04 (0.97, 1.11) | 0.267 |
| 509.2 | Respiratory insufficiency | 12520 | 17595490 | 5453 | 1 (0.93, 1.07) | 0.97 | 1362 | 0.97 (0.79, 1.2) | 0.806 | 542 | 0.97 (0.65, 1.44) | 0.883 | 10605 | 1.04 (0.93, 1.16) | 0.48 |
| 509.3 | Pulmonary insufficiency or respiratory failure following trauma and surgery | 268 | 17630738 | 115 | 0.95 (0.34, 2.66) | 0.929 | 32 | 1.06 (0.17, 6.77) | 0.955 | 7 | 0.57 (0.17, 1.88) | 0.363 | 220 | 0.87 (0.42, 1.8) | 0.721 |
| 509.5 | Respiratory arrest | 1251 | 17630174 | 545 | 1 (0.98, 1.02) | 0.97 | 134 | 0.98 (0.31, 3.06) | 0.97 | 59 | 1.07 (0.36, 3.15) | 0.914 | 1026 | 0.86 (0.71, 1.06) | 0.157 |
| 509.8 | Dependence on respirator [Ventilator] or supplemental oxygen | 2292 | 17623484 | 996 | 1.01 (0.61, 1.69) | 0.97 | 273 | 1.09 (0.64, 1.85) | 0.773 | 96 | 0.96 (0.21, 4.4) | 0.959 | 1931 | 1 (0.9, 1.11) | 0.97 |
| 510 | Other diseases of lung | 1027 | 17624653 | 434 | 0.92 (0.62, 1.37) | 0.7 | 114 | 0.92 (0.38, 2.21) | 0.859 | 32 | 0.65 (0.36, 1.18) | 0.158 | 878 | 1.09 (0.6, 2) | 0.788 |
| 510.2 | Lung transplant | 246 | 17630743 | 102 | 0.83 (0.43, 1.62) | 0.607 | 18 | 0.55 (0.26, 1.16) | 0.114 | 10 | 0.78 (0.06, 10.73) | 0.861 | 208 | 1 (0.83, 1.22) | 0.97 |
| 512 | Other symptoms of respiratory system | 32398 | 17490612 | 14120 | 1.02 (0.95, 1.09) | 0.597 | 3666 | 1.03 (0.91, 1.16) | 0.706 | 1460 | 1.02 (0.78, 1.34) | 0.875 | 27257 | 0.99 (0.83, 1.18) | 0.93 |
| 512.2 | Painful respiration | 1707 | 17626932 | 736 | 1.07 (0.05, 21.78) | 0.97 | 216 | 1.12 (0, 286.41) | 0.97 | 92 | 1.24 (0, 28562.53) | 0.97 | 1452 | 1.03 (0.28, 3.74) | 0.97 |
| 512.7 | Shortness of breath | 30316 | 17500632 | 13230 | 1.02 (0.95, 1.1) | 0.637 | 3399 | 1.02 (0.87, 1.2) | 0.834 | 1357 | 1.02 (0.77, 1.35) | 0.91 | 25486 | 0.99 (0.85, 1.16) | 0.898 |
| 512.8 | Cough | 145 | 17629982 | 53 | 0.73 (0.34, 1.57) | 0.426 | 20 | 0.97 (0.29, 3.25) | 0.97 | 2 | 0.25 (0.02, 3.79) | 0.325 | 127 | 1.27 (0.22, 7.41) | 0.805 |
| 512.9 | Other dyspnea | 675 | 17627312 | 282 | 0.95 (0.48, 1.9) | 0.902 | 78 | 0.96 (0.23, 3.91) | 0.956 | 33 | 1.05 (0.12, 9.09) | 0.97 | 571 | 1 (0.99, 1.01) | 0.97 |
| 513 | Respiratory abnormalities | 5909 | 17550123 | 2602 | 1.08 (0.98, 1.2) | 0.133 | 708 | 1.04 (0.66, 1.63) | 0.882 | 281 | 1.07 (0.64, 1.79) | 0.8 | 4966 | 0.94 (0.77, 1.16) | 0.592 |
| 513.3 | Hypoventilation | 510 | 17622662 | 236 | 1.16 (0.32, 4.23) | 0.838 | 56 | 0.98 (0.37, 2.61) | 0.97 | 22 | 1 (0.88, 1.14) | 0.97 | 424 | 0.89 (0.14, 5.75) | 0.909 |
| 513.4 | Hyperventilation | 5060 | 17561750 | 2226 | 1.08 (0.97, 1.2) | 0.145 | 608 | 1.03 (0.65, 1.64) | 0.898 | 240 | 1.07 (0.6, 1.9) | 0.84 | 4249 | 0.94 (0.77, 1.13) | 0.496 |
| 513.8 | Disorders of diaphragm | 260 | 17629718 | 114 | 1.05 (0.19, 5.75) | 0.958 | 31 | 1.11 (0.14, 8.99) | 0.929 | 13 | 1.17 (0.06, 22.55) | 0.923 | 227 | 1.29 (0.47, 3.51) | 0.636 |
| 514 | Abnormal findings examination of lungs | 500 | 17627485 | 225 | 1.07 (0.49, 2.33) | 0.883 | 51 | 0.94 (0.18, 4.88) | 0.949 | 24 | 1.11 (0.15, 8.37) | 0.929 | 414 | 0.9 (0.41, 1.99) | 0.814 |
| 514.1 | Abnormal results of function study of pulmonary system | 499 | 17627493 | 224 | 1.06 (0.47, 2.41) | 0.897 | 51 | 0.94 (0.18, 4.84) | 0.949 | 24 | 1.11 (0.15, 8.39) | 0.929 | 414 | 0.91 (0.36, 2.3) | 0.86 |
| 516 | Abnormal sputum | 4110 | 17595281 | 1717 | 0.96 (0.73, 1.28) | 0.814 | 516 | 1.12 (0.9, 1.39) | 0.33 | 193 | 1.05 (0.48, 2.31) | 0.912 | 3474 | 1.02 (0.57, 1.8) | 0.959 |
| 516.1 | Hemoptysis | 4033 | 17595656 | 1693 | 0.97 (0.69, 1.38) | 0.883 | 504 | 1.11 (0.89, 1.4) | 0.364 | 188 | 1.04 (0.44, 2.5) | 0.929 | 3408 | 1.01 (0.53, 1.96) | 0.968 |
| 519 | Other diseases of respiratory system, not elsewhere classified | 3366 | 17606355 | 1481 | 1.03 (0.7, 1.51) | 0.889 | 402 | 1.06 (0.64, 1.76) | 0.836 | 117 | 0.78 (0.53, 1.14) | 0.195 | 2811 | 0.94 (0.69, 1.29) | 0.706 |
| 519.2 | Respiratory complications | 655 | 17630207 | 265 | 0.88 (0.46, 1.67) | 0.698 | 71 | 0.9 (0.19, 4.22) | 0.898 | 32 | 1.02 (0.34, 3.07) | 0.97 | 556 | 1.04 (0.16, 6.82) | 0.967 |
| 519.8 | Other diseases of respiratory system, NEC | 972 | 17626530 | 441 | 1.09 (0.8, 1.49) | 0.588 | 118 | 1.12 (0.65, 1.91) | 0.703 | 30 | 0.72 (0.38, 1.34) | 0.3 | 808 | 0.92 (0.59, 1.42) | 0.71 |
| 519.9 | Symptoms involving respiratory system and other chest symptoms | 497 | 17627263 | 220 | 1.04 (0.27, 3.97) | 0.963 | 59 | 1.02 (0.4, 2.63) | 0.97 | 15 | 0.66 (0.15, 2.97) | 0.605 | 409 | 0.85 (0.42, 1.73) | 0.664 |
| **Digestive** | | | | | | | | | | | | | | | |
| 520 | Disorders of tooth development | 4305 | 17574398 | 1903 | 1.06 (0.83, 1.35) | 0.67 | 476 | 0.89 (0.66, 1.21) | 0.475 | 181 | 0.9 (0.46, 1.74) | 0.763 | 3620 | 0.93 (0.7, 1.23) | 0.609 |
| 520.1 | Hereditary disturbances in tooth structure | 1980 | 482914* | 866 | 1.03 (0.51, 2.08)** | 0.935 | 218 | 0.84 (0.52, 1.36)** | 0.487 | 76 | 0.79 (0.34, 1.79)** | 0.578 | 1664 | 0.91 (0.55, 1.49)** | 0.708 |
| 520.2 | Disturbances in tooth eruption | 2996 | 17592399 | 1308 | 1.04 (0.69, 1.57) | 0.86 | 348 | 0.95 (0.48, 1.86) | 0.883 | 125 | 0.9 (0.39, 2.04) | 0.805 | 2530 | 0.96 (0.56, 1.65) | 0.885 |
| 521 | Diseases of hard tissues of teeth | 4146 | 17590240 | 1788 | 1 (0.88, 1.13) | 0.97 | 496 | 1.02 (0.55, 1.92) | 0.949 | 157 | 0.83 (0.6, 1.15) | 0.267 | 3503 | 0.99 (0.78, 1.27) | 0.97 |
| 521.1 | Dental caries | 3671 | 17595466 | 1606 | 1.01 (0.62, 1.65) | 0.97 | 422 | 0.99 (0.61, 1.61) | 0.97 | 134 | 0.8 (0.58, 1.11) | 0.185 | 3110 | 1.02 (0.55, 1.87) | 0.962 |
| 521.4 | Tooth complications likely association with other diseases | 304 | 17628133 | 120 | 0.97 (0.18, 5.19) | 0.97 | 49 | 1.37 (0.53, 3.5) | 0.527 | 15 | 1.09 (0.02, 74.11) | 0.97 | 255 | 0.92 (0.14, 6.19) | 0.935 |
| 522 | Diseases of pulp and periapical tissues | 1504 | 17614390 | 636 | 0.97 (0.57, 1.63) | 0.906 | 176 | 0.99 (0.62, 1.59) | 0.97 | 65 | 0.94 (0.24, 3.64) | 0.93 | 1247 | 0.89 (0.65, 1.21) | 0.462 |
| 522.1 | Pulpitis and necrosis of tooth pulp | 430 | 17627141 | 165 | 0.84 (0.52, 1.36) | 0.487 | 56 | 1.03 (0.31, 3.37) | 0.97 | 21 | 1 (0.84, 1.18) | 0.97 | 362 | 0.96 (0.17, 5.38) | 0.965 |
| 522.5 | Periapical abscess | 702 | 17624609 | 323 | 1.08 (0.63, 1.85) | 0.783 | 77 | 0.96 (0.2, 4.49) | 0.958 | 18 | 0.57 (0.27, 1.22) | 0.15 | 587 | 0.93 (0.39, 2.24) | 0.889 |
| 523 | Gingival and periodontal diseases | 8183 | 17573839 | 3547 | 0.99 (0.7, 1.41) | 0.968 | 899 | 0.97 (0.68, 1.4) | 0.89 | 358 | 0.97 (0.53, 1.8) | 0.935 | 6868 | 0.97 (0.74, 1.28) | 0.861 |
| 523.1 | Gingivitis | 552 | 17626261 | 216 | 0.83 (0.5, 1.39) | 0.487 | 65 | 0.9 (0.19, 4.2) | 0.904 | 22 | 0.79 (0.1, 6.37) | 0.835 | 463 | 0.94 (0.2, 4.39) | 0.943 |
| 523.3 | Periodontitis (acute or chronic) | 138 | 17630866 | 74 | 1.37 (0.11, 17.15) | 0.817 | 9 | 0.65 (0, 341.91) | 0.901 | 4 | 0.72 (0, 168862.41) | 0.963 | 114 | 0.89 (0, 210.13) | 0.97 |
| 523.31 | Acute periodontitis | 736 | 17623576 | 300 | 0.88 (0.58, 1.31) | 0.534 | 81 | 0.88 (0.35, 2.24) | 0.8 | 29 | 0.8 (0.21, 3.07) | 0.763 | 614 | 0.92 (0.37, 2.27) | 0.87 |
| 523.32 | Chronic periodontitis | 6591 | 17590160 | 2885 | 1.01 (0.76, 1.35) | 0.935 | 728 | 1 (0.87, 1.15) | 0.97 | 293 | 1.01 (0.68, 1.48) | 0.97 | 5540 | 0.99 (0.62, 1.58) | 0.966 |
| 524 | Dentofacial anomalies, including malocclusion | 3212 | 17593565 | 1372 | 1.01 (0.79, 1.28) | 0.97 | 373 | 0.92 (0.61, 1.38) | 0.695 | 141 | 0.92 (0.38, 2.23) | 0.861 | 2700 | 0.92 (0.69, 1.24) | 0.599 |
| 524.3 | Anomalies of tooth position/malocclusion | 2606 | 17601269 | 1106 | 1 (0.83, 1.22) | 0.97 | 312 | 0.94 (0.48, 1.85) | 0.864 | 116 | 0.93 (0.3, 2.82) | 0.903 | 2210 | 0.97 (0.47, 1.98) | 0.934 |
| 525 | Other diseases of the teeth and supporting structures | 6712 | 17561263 | 2857 | 0.97 (0.82, 1.14) | 0.693 | 763 | 0.94 (0.76, 1.15) | 0.543 | 264 | 0.83 (0.68, 1.02) | 0.074 | 5651 | 0.97 (0.75, 1.25) | 0.804 |
| 525.2 | Atrophy of edentulous alveolar ridge | 807 | 17623792 | 345 | 0.94 (0.46, 1.92) | 0.885 | 85 | 0.85 (0.42, 1.74) | 0.671 | 27 | 0.69 (0.28, 1.71) | 0.431 | 680 | 0.98 (0.33, 2.87) | 0.97 |
| 526 | Diseases of the jaws | 6670 | 17564241 | 2900 | 1.03 (0.84, 1.27) | 0.804 | 761 | 0.94 (0.74, 1.21) | 0.668 | 289 | 0.94 (0.58, 1.51) | 0.8 | 5635 | 0.97 (0.7, 1.35) | 0.877 |
| 526.1 | Cysts of the jaws | 789 | 17622492 | 358 | 1.11 (0.66, 1.88) | 0.697 | 98 | 1.13 (0.43, 2.98) | 0.819 | 27 | 0.79 (0.16, 3.85) | 0.788 | 663 | 0.96 (0.26, 3.59) | 0.959 |
| 526.3 | Anomalies of jaw size/symmetry | 2410 | 17604085 | 1044 | 1.06 (0.77, 1.45) | 0.746 | 288 | 0.96 (0.45, 2.05) | 0.923 | 115 | 1.02 (0.36, 2.9) | 0.97 | 2038 | 0.95 (0.56, 1.6) | 0.849 |
| 526.41 | Temporomandibular joint disorder, unspecified | 2363 | 17608269 | 1001 | 0.97 (0.65, 1.46) | 0.898 | 265 | 0.89 (0.66, 1.19) | 0.439 | 98 | 0.86 (0.5, 1.47) | 0.6 | 2009 | 1.01 (0.63, 1.61) | 0.97 |
| 526.5 | Inflammatory conditions of jaw | 1059 | 17625250 | 473 | 1.03 (0.52, 2.07) | 0.93 | 108 | 0.92 (0.35, 2.38) | 0.87 | 46 | 0.98 (0.4, 2.4) | 0.97 | 880 | 0.92 (0.51, 1.65) | 0.788 |
| 526.9 | Jaw disease NOS | 504 | 17627449 | 211 | 0.98 (0.37, 2.62) | 0.97 | 64 | 1.09 (0.33, 3.57) | 0.898 | 23 | 1 (0.85, 1.19) | 0.97 | 437 | 1.19 (0.65, 2.18) | 0.588 |
| 527 | Diseases of the salivary glands | 2631 | 17604591 | 1139 | 1.01 (0.6, 1.7) | 0.97 | 292 | 0.98 (0.4, 2.4) | 0.962 | 125 | 1.06 (0.38, 2.94) | 0.92 | 2240 | 1.06 (0.69, 1.63) | 0.807 |
| 527.1 | Hypertrophy of salivary gland | 189 | 17629910 | 88 | 1.23 (0.52, 2.9) | 0.644 | 23 | 1.22 (0.16, 9) | 0.859 | 10 | 1.34 (0.1, 17.33) | 0.834 | 163 | 1.16 (0.19, 7.22) | 0.883 |
| 527.2 | Sialoadenitis | 1794 | 17611592 | 785 | 1.03 (0.56, 1.88) | 0.939 | 199 | 0.98 (0.3, 3.12) | 0.97 | 80 | 1 (0.81, 1.22) | 0.97 | 1524 | 1.04 (0.52, 2.09) | 0.914 |
| 527.7 | Disturbance of salivary secretion | 501 | 17628220 | 211 | 0.96 (0.2, 4.63) | 0.959 | 47 | 0.82 (0.16, 4.18) | 0.818 | 32 | 1.39 (0.32, 6.1) | 0.675 | 418 | 0.94 (0.12, 7.08) | 0.956 |
| 527.8 | Other specified diseases of the salivary glands | 111 | 17630838 | 45 | 0.98 (0.31, 3.05) | 0.97 | 13 | 1.06 (0.08, 13.32) | 0.97 | 9 | 1.86 (0.06, 58.17) | 0.737 | 92 | 0.89 (0, 189.9) | 0.969 |
| 528 | Diseases of the oral soft tissues, excluding lesions specific for gingiva and tongue | 6869 | 17552157 | 2941 | 0.98 (0.75, 1.28) | 0.898 | 771 | 0.96 (0.7, 1.3) | 0.782 | 298 | 0.94 (0.57, 1.56) | 0.826 | 5856 | 1.06 (0.89, 1.27) | 0.509 |
| 528.11 | Stomatitis and mucositis (ulcerative) | 733 | 17622919 | 334 | 1.13 (0.61, 2.1) | 0.7 | 82 | 0.99 (0.6, 1.63) | 0.97 | 34 | 1.06 (0.05, 20.75) | 0.97 | 634 | 1.15 (0.46, 2.91) | 0.773 |
| 528.12 | Oral aphthae | 695 | 17622714 | 306 | 1.03 (0.32, 3.36) | 0.959 | 73 | 0.85 (0.32, 2.31) | 0.767 | 30 | 0.92 (0.06, 13.98) | 0.956 | 602 | 1.15 (0.53, 2.52) | 0.733 |
| 528.3 | Cellulitis and abscess of oral soft tissues | 1450 | 17617221 | 592 | 0.92 (0.67, 1.26) | 0.607 | 174 | 0.99 (0.54, 1.82) | 0.97 | 69 | 1.01 (0.71, 1.44) | 0.97 | 1241 | 1.08 (0.62, 1.88) | 0.796 |
| 528.41 | Cyst of the salivary gland | 376 | 17626407 | 144 | 0.84 (0.47, 1.51) | 0.575 | 52 | 1.07 (0.15, 7.49) | 0.953 | 15 | 0.8 (0.08, 8.3) | 0.864 | 319 | 1 (0.94, 1.06) | 0.97 |
| 528.5 | Diseases of lips | 234 | 17628791 | 89 | 0.78 (0.23, 2.72) | 0.714 | 28 | 0.89 (0.02, 42.72) | 0.959 | 8 | 0.66 (0.01, 64.26) | 0.868 | 209 | 1.52 (0.29, 8.07) | 0.635 |
| 528.6 | Leukoplakia of oral mucosa | 1241 | 17617791 | 552 | 1.03 (0.51, 2.1) | 0.935 | 135 | 1.01 (0.69, 1.46) | 0.97 | 50 | 0.92 (0.17, 5.11) | 0.932 | 1035 | 0.95 (0.44, 2.08) | 0.909 |
| 528.7 | Sialolithiasis | 1275 | 17616341 | 550 | 0.98 (0.47, 2.08) | 0.97 | 135 | 0.9 (0.47, 1.72) | 0.767 | 57 | 0.97 (0.22, 4.24) | 0.97 | 1097 | 1.14 (0.76, 1.69) | 0.539 |
| 529 | Diseases and other conditions of the tongue | 932 | 17624214 | 402 | 1.02 (0.4, 2.58) | 0.97 | 112 | 1.08 (0.32, 3.63) | 0.911 | 43 | 1.05 (0.12, 9.15) | 0.97 | 766 | 0.85 (0.51, 1.44) | 0.564 |
| 529.1 | Glossitis | 577 | 17627756 | 250 | 1.04 (0.26, 4.14) | 0.959 | 69 | 1.1 (0.21, 5.87) | 0.92 | 30 | 1.21 (0.15, 9.56) | 0.869 | 473 | 0.84 (0.39, 1.83) | 0.68 |
| 529.6 | Glossodynia | 117 | 17630547 | 49 | 0.93 (0.14, 6.19) | 0.949 | 15 | 1.1 (0.06, 20.91) | 0.955 | 3 | 0.55 (0.03, 9.59) | 0.695 | 99 | 1.03 (0.3, 3.5) | 0.97 |
| 530 | Diseases of esophagus | 17852 | 17489392 | 7543 | 0.97 (0.89, 1.06) | 0.505 | 2127 | 1.04 (0.92, 1.19) | 0.536 | 812 | 1.01 (0.76, 1.34) | 0.97 | 15136 | 1.03 (0.92, 1.16) | 0.599 |
| 530.11 | GERD | 7461 | 17582158 | 3184 | 1.02 (0.86, 1.22) | 0.79 | 958 | **1.14 (1.05, 1.25)** | **0.003** | 350 | 1.07 (0.77, 1.47) | 0.708 | 6410 | **1.12 (1.03, 1.21)** | **0.007** |
| 530.12 | Ulcer of esophagus | 607 | 17628155 | 246 | 0.85 (0.55, 1.32) | 0.475 | 63 | 0.86 (0.28, 2.62) | 0.803 | 25 | 0.85 (0.11, 6.54) | 0.885 | 527 | 1.24 (0.68, 2.28) | 0.49 |
| 530.14 | Reflux esophagitis | 6429 | 17569091 | 2706 | 0.96 (0.82, 1.13) | 0.638 | 753 | 1.03 (0.68, 1.54) | 0.909 | 304 | 1.04 (0.57, 1.91) | 0.898 | 5385 | 0.96 (0.77, 1.19) | 0.713 |
| 530.2 | Esophageal bleeding (varices/hemorrhage) | 1945 | 17621418 | 842 | 0.96 (0.57, 1.6) | 0.883 | 201 | 0.9 (0.53, 1.52) | 0.7 | 76 | 0.85 (0.38, 1.91) | 0.708 | 1627 | 0.96 (0.5, 1.83) | 0.906 |
| 530.3 | Stricture and stenosis of esophagus | 1798 | 17620968 | 726 | **0.85 (0.74, 0.99)** | **0.034** | 194 | 0.91 (0.55, 1.5) | 0.725 | 79 | 0.92 (0.32, 2.67) | 0.885 | 1525 | 1.06 (0.6, 1.88) | 0.856 |
| 530.5 | Disorders of esophageal motility | 688 | 17625305 | 278 | 0.92 (0.41, 2.08) | 0.86 | 85 | 1.09 (0.31, 3.86) | 0.901 | 40 | 1.29 (0.46, 3.6) | 0.644 | 573 | 0.93 (0.32, 2.66) | 0.899 |
| 530.6 | Diverticulum of esophagus, acquired | 279 | 17629195 | 108 | 0.81 (0.12, 5.31) | 0.84 | 29 | 0.88 (0.01, 82.82) | 0.959 | 17 | 1.27 (0.01, 145.84) | 0.929 | 243 | 1.29 (0.08, 20.29) | 0.868 |
| 530.7 | Gastroesophageal laceration-hemorrhage syndrome | 597 | 17623900 | 278 | 1.13 (0.71, 1.79) | 0.633 | 72 | 1.08 (0.29, 3.94) | 0.919 | 12 | 0.46 (0.19, 1.09) | 0.078 | 511 | 1.09 (0.39, 3.02) | 0.883 |
| 531 | Peptic ulcer (excl. esophageal) | 16678 | 17443704 | 6864 | **0.88 (0.85, 0.92)** | **<0.001** | 1837 | 0.94 (0.84, 1.05) | 0.254 | 686 | **0.87 (0.77, 0.98)** | **0.027** | 14065 | 1.02 (0.83, 1.26) | 0.865 |
| 531.1 | Hemorrhage from gastrointestinal ulcer | 6277 | 17583751 | 2589 | **0.86 (0.8, 0.92)** | **<0.001** | 614 | **0.82 (0.72, 0.93)** | **0.002** | 254 | 0.84 (0.65, 1.08) | 0.166 | 5331 | 1.07 (0.9, 1.29) | 0.443 |
| 531.2 | Gastric ulcer | 6745 | 17555312 | 2783 | **0.89 (0.83, 0.96)** | **0.002** | 750 | 0.95 (0.72, 1.25) | 0.734 | 276 | 0.87 (0.67, 1.13) | 0.3 | 5646 | 0.97 (0.72, 1.31) | 0.849 |
| 531.3 | Duodenal ulcer | 4534 | 17555684 | 1834 | **0.87 (0.8, 0.95)** | **0.001** | 519 | 0.98 (0.47, 2.06) | 0.965 | 194 | 0.91 (0.54, 1.51) | 0.722 | 3837 | 1.05 (0.75, 1.46) | 0.802 |
| 531.4 | Peptic ulcer, site unspecified | 1743 | 17618232 | 754 | 0.98 (0.58, 1.64) | 0.938 | 191 | 0.96 (0.43, 2.14) | 0.935 | 68 | 0.86 (0.43, 1.72) | 0.681 | 1478 | 1.05 (0.59, 1.87) | 0.883 |
| 531.5 | Gastrojejunal ulcer | 586 | 17628539 | 227 | 0.89 (0.57, 1.37) | 0.607 | 88 | 1.34 (0.95, 1.89) | 0.093 | 29 | 1.11 (0.22, 5.7) | 0.911 | 480 | 0.85 (0.55, 1.31) | 0.458 |
| 535 | Gastritis and duodenitis | 17792 | 17419254 | 7546 | 0.98 (0.87, 1.09) | 0.713 | 2130 | 1.05 (0.93, 1.19) | 0.435 | 789 | 0.98 (0.6, 1.61) | 0.949 | 15089 | 1.04 (0.92, 1.17) | 0.57 |
| 535.1 | Acute gastritis | 2413 | 17614962 | 1091 | 1.06 (0.83, 1.35) | 0.673 | 260 | 0.97 (0.47, 2) | 0.935 | 83 | 0.78 (0.52, 1.15) | 0.207 | 2032 | 0.99 (0.77, 1.29) | 0.97 |
| 535.2 | Atrophic gastritis | 443 | 17628042 | 172 | 0.84 (0.48, 1.45) | 0.54 | 47 | 0.91 (0.15, 5.39) | 0.923 | 30 | 1.44 (0.54, 3.81) | 0.474 | 384 | 1.24 (0.55, 2.79) | 0.624 |
| 535.6 | Duodenitis | 1124 | 17622292 | 460 | 0.87 (0.71, 1.07) | 0.194 | 118 | 0.87 (0.53, 1.44) | 0.606 | 47 | 0.87 (0.31, 2.42) | 0.8 | 952 | 1.04 (0.46, 2.37) | 0.935 |
| 535.8 | Other specified gastritis | 3785 | 17599597 | 1596 | 0.99 (0.63, 1.55) | 0.959 | 473 | 1.12 (0.9, 1.39) | 0.3 | 179 | 1.07 (0.52, 2.21) | 0.87 | 3190 | 1 (0.88, 1.13) | 0.97 |
| 537 | Other disorders of stomach and duodenum | 1622 | 17615867 | 695 | 0.93 (0.35, 2.44) | 0.887 | 158 | 0.82 (0.28, 2.43) | 0.73 | 60 | 0.78 (0.1, 5.87) | 0.818 | 1369 | 1.01 (0.55, 1.88) | 0.97 |
| 540 | Appendiceal conditions | 11597 | 17477060 | 4950 | 1.01 (0.73, 1.4) | 0.958 | 1401 | 0.98 (0.61, 1.59) | 0.955 | 511 | 0.94 (0.62, 1.43) | 0.788 | 9777 | 0.95 (0.81, 1.11) | 0.539 |
| 540.11 | Acute appendicitis | 11416 | 17480208 | 4875 | 1.01 (0.71, 1.43) | 0.964 | 1370 | 0.98 (0.68, 1.41) | 0.906 | 504 | 0.94 (0.61, 1.45) | 0.797 | 9634 | 0.96 (0.8, 1.15) | 0.644 |
| 550 | Abdominal hernia | 47761 | 16976073 | 20831 | 1 (0.86, 1.15) | 0.97 | 5112 | **0.94 (0.89, 0.99)** | **0.014** | 2083 | 0.96 (0.82, 1.13) | 0.645 | 40279 | 1.01 (0.83, 1.22) | 0.959 |
| 550.1 | Inguinal hernia | 29335 | 17183189 | 12729 | 0.98 (0.9, 1.07) | 0.671 | 3140 | **0.93 (0.87, 1)** | **0.046** | 1256 | 0.94 (0.8, 1.1) | 0.432 | 24703 | 1 (0.93, 1.07) | 0.97 |
| 550.2 | Diaphragmatic hernia | 7386 | 17548576 | 3281 | 1.05 (0.9, 1.22) | 0.531 | 823 | 1.03 (0.66, 1.62) | 0.906 | 324 | 1.01 (0.6, 1.69) | 0.97 | 6272 | 1.06 (0.86, 1.31) | 0.6 |
| 550.3 | Femoral hernia | 1235 | 17615573 | 508 | 0.89 (0.57, 1.39) | 0.613 | 123 | 0.83 (0.39, 1.76) | 0.64 | 60 | 1.01 (0.51, 2.03) | 0.97 | 1012 | 0.85 (0.52, 1.38) | 0.523 |
| 550.4 | Umbilical hernia | 7428 | 17557384 | 3199 | 0.98 (0.6, 1.62) | 0.955 | 793 | 0.91 (0.65, 1.27) | 0.597 | 351 | 1.03 (0.34, 3.1) | 0.959 | 6276 | 1 (0.92, 1.09) | 0.97 |
| 550.5 | Ventral hernia | 6602 | 17564921 | 2902 | 1.01 (0.69, 1.47) | 0.959 | 693 | 0.93 (0.72, 1.22) | 0.629 | 285 | 0.96 (0.47, 1.98) | 0.924 | 5608 | 1.06 (0.85, 1.31) | 0.624 |
| 555 | Inflammatory bowel disease and other gastroenteritis and colitis | 8903 | 17528614 | 3784 | 0.99 (0.75, 1.31) | 0.94 | 1050 | 0.99 (0.6, 1.63) | 0.97 | 399 | 0.97 (0.53, 1.79) | 0.929 | 7481 | 0.95 (0.81, 1.12) | 0.567 |
| 555.1 | Regional enteritis | 3969 | 17588290 | 1724 | 1.04 (0.75, 1.43) | 0.835 | 464 | 0.99 (0.69, 1.43) | 0.97 | 184 | 1.02 (0.37, 2.85) | 0.97 | 3354 | 0.98 (0.52, 1.85) | 0.955 |
| 555.2 | Ulcerative colitis | 4990 | 17575608 | 2081 | 0.96 (0.78, 1.18) | 0.71 | 608 | 1.02 (0.57, 1.83) | 0.949 | 227 | 0.98 (0.38, 2.52) | 0.97 | 4200 | 0.97 (0.67, 1.39) | 0.861 |
| 555.21 | Ulcerative colitis (chronic) | 3783 | 17595263 | 1598 | 0.98 (0.71, 1.35) | 0.901 | 459 | 1.01 (0.73, 1.38) | 0.97 | 159 | 0.9 (0.56, 1.45) | 0.683 | 3186 | 0.96 (0.68, 1.36) | 0.84 |
| 556 | Ulceration of the lower GI tract | 175 | 17630323 | 86 | 1.13 (0.21, 6.1) | 0.892 | 13 | 0.66 (0.08, 5.08) | 0.7 | 4 | 0.51 (0.01, 18.59) | 0.725 | 145 | 0.9 (0.07, 11.34) | 0.942 |
| 557 | Intestinal malabsorption (non-celiac) | 1749 | 17611163 | 794 | 1.11 (0.89, 1.4) | 0.364 | 187 | 0.93 (0.43, 2.02) | 0.868 | 81 | 1.05 (0.21, 5.23) | 0.959 | 1451 | 0.88 (0.66, 1.16) | 0.362 |
| 557.1 | Celiac disease | 1032 | 17620502 | 474 | 1.15 (0.8, 1.64) | 0.464 | 107 | 0.91 (0.29, 2.9) | 0.883 | 52 | 1.15 (0.26, 5.11) | 0.861 | 853 | 0.85 (0.54, 1.34) | 0.504 |
| 558 | Noninfectious gastroenteritis | 10381 | 17524954 | 4411 | 0.98 (0.77, 1.24) | 0.859 | 1213 | 0.99 (0.63, 1.55) | 0.97 | 438 | 0.91 (0.64, 1.3) | 0.629 | 8779 | 1 (0.86, 1.17) | 0.97 |
| 559 | Ileostomy status | 3073 | 17617063 | 1307 | 0.94 (0.82, 1.09) | 0.426 | 323 | 0.9 (0.74, 1.1) | 0.327 | 132 | 0.93 (0.55, 1.55) | 0.784 | 2574 | 0.96 (0.71, 1.3) | 0.818 |
| 560 | Intestinal obstruction without mention of hernia | 10103 | 17555684 | 4401 | 0.99 (0.69, 1.43) | 0.97 | 1091 | 0.95 (0.72, 1.25) | 0.73 | 401 | 0.87 (0.66, 1.15) | 0.347 | 8531 | 1.01 (0.65, 1.58) | 0.953 |
| 560.1 | Paralytic ileus | 6016 | 17597131 | 2687 | 1.03 (0.76, 1.4) | 0.861 | 621 | 0.93 (0.67, 1.29) | 0.671 | 233 | 0.87 (0.57, 1.33) | 0.528 | 5074 | 1.01 (0.59, 1.73) | 0.97 |
| 560.2 | Impaction of intestine | 133 | 17631165 | 55 | 0.87 (0.13, 5.76) | 0.897 | 12 | 0.72 (0.06, 8.57) | 0.808 | 6 | 0.91 (0.01, 78.69) | 0.97 | 115 | 1.19 (0.1, 14.24) | 0.902 |
| 560.3 | Peritoneal or intestinal adhesions | 1660 | 17605959 | 708 | 0.96 (0.54, 1.7) | 0.891 | 194 | 1.02 (0.35, 3.02) | 0.97 | 57 | 0.75 (0.37, 1.53) | 0.435 | 1379 | 0.92 (0.55, 1.54) | 0.767 |
| 560.4 | Other intestinal obstruction | 5861 | 17576220 | 2498 | 0.97 (0.73, 1.29) | 0.835 | 670 | 1 (0.82, 1.21) | 0.97 | 241 | 0.9 (0.54, 1.5) | 0.696 | 4953 | 1.02 (0.58, 1.77) | 0.959 |
| 561 | Symptoms involving digestive system | 6628 | 17582842 | 2864 | 0.99 (0.67, 1.46) | 0.954 | 716 | 0.93 (0.73, 1.18) | 0.552 | 280 | 0.92 (0.58, 1.45) | 0.733 | 5586 | 0.99 (0.6, 1.62) | 0.97 |
| 561.1 | Diarrhea | 2419 | 17605799 | 1055 | 1.03 (0.52, 2.03) | 0.942 | 278 | 0.99 (0.65, 1.5) | 0.97 | 101 | 0.92 (0.22, 3.93) | 0.92 | 2023 | 0.93 (0.53, 1.65) | 0.818 |
| 562 | Diverticulosis and diverticulitis | 16569 | 17515568 | 6992 | **0.91 (0.86, 0.96)** | **0.001** | 1671 | **0.87 (0.79, 0.96)** | **0.005** | 669 | 0.86 (0.72, 1.02) | 0.088 | 13879 | 0.98 (0.75, 1.28) | 0.885 |
| 562.1 | Diverticulosis | 16569 | 17515568 | 6992 | **0.91 (0.86, 0.96)** | **0.001** | 1671 | **0.87 (0.79, 0.96)** | **0.005** | 669 | 0.86 (0.72, 1.02) | 0.088 | 13879 | 0.98 (0.75, 1.28) | 0.885 |
| 563 | Constipation | 28721 | 17433410 | 12247 | 0.99 (0.87, 1.12) | 0.836 | 3300 | 1 (0.99, 1.01) | 0.97 | 1344 | 1.03 (0.79, 1.36) | 0.817 | 24314 | 1.02 (0.88, 1.18) | 0.791 |
| 564 | Functional digestive disorders | 15599 | 17484157 | 6635 | 0.98 (0.86, 1.11) | 0.771 | 1753 | 0.95 (0.83, 1.1) | 0.521 | 731 | 1.02 (0.62, 1.67) | 0.949 | 13141 | 0.98 (0.82, 1.17) | 0.826 |
| 564.1 | Irritable Bowel Syndrome | 9443 | 17522846 | 4011 | 0.98 (0.82, 1.16) | 0.813 | 1054 | 0.93 (0.81, 1.07) | 0.308 | 436 | 0.99 (0.65, 1.51) | 0.97 | 7965 | 0.98 (0.75, 1.28) | 0.885 |
| 564.8 | Abnormal findings on exam of gastrointestinal tract/ abdominal area | 748 | 17629544 | 326 | 1 (0.92, 1.09) | 0.97 | 73 | 0.87 (0.53, 1.43) | 0.607 | 39 | 1.17 (0.56, 2.42) | 0.693 | 638 | 1.09 (0.69, 1.73) | 0.73 |
| 564.9 | Personal history of diseases of digestive system | 689 | 17627612 | 290 | 0.92 (0.62, 1.38) | 0.71 | 75 | 0.93 (0.35, 2.42) | 0.883 | 26 | 0.8 (0.31, 2.1) | 0.668 | 580 | 0.99 (0.76, 1.29) | 0.97 |
| 565 | Anal and rectal conditions | 16717 | 17405826 | 7057 | 0.97 (0.89, 1.06) | 0.491 | 1984 | 1.02 (0.78, 1.33) | 0.9 | 752 | 0.98 (0.58, 1.68) | 0.956 | 14102 | 0.99 (0.73, 1.34) | 0.956 |
| 565.1 | Anal and rectal polyp | 1071 | 17621716 | 459 | 0.95 (0.51, 1.77) | 0.892 | 109 | 0.9 (0.41, 1.96) | 0.799 | 49 | 1 (0.99, 1.01) | 0.97 | 918 | 1.14 (0.71, 1.81) | 0.606 |
| 567 | Peritonitis and retroperitoneal infections | 3948 | 17604395 | 1701 | 0.98 (0.58, 1.67) | 0.956 | 464 | 1.03 (0.53, 2.01) | 0.93 | 144 | 0.81 (0.53, 1.22) | 0.31 | 3369 | 1.08 (0.81, 1.44) | 0.593 |
| 568 | Other disorders of peritoneum | 2156 | 17596770 | 970 | 1.12 (0.87, 1.43) | 0.384 | 261 | 1.11 (0.64, 1.91) | 0.731 | 87 | 0.94 (0.18, 4.82) | 0.946 | 1782 | 0.87 (0.65, 1.17) | 0.361 |
| 568.1 | Peritoneal adhesions (postoperative) (postinfection) | 1918 | 17598922 | 855 | 1.11 (0.84, 1.47) | 0.488 | 236 | 1.12 (0.65, 1.93) | 0.693 | 81 | 0.98 (0.44, 2.21) | 0.97 | 1594 | 0.9 (0.61, 1.33) | 0.607 |
| 569 | Other disorders of intestine | 5293 | 17597291 | 2260 | 0.97 (0.73, 1.28) | 0.825 | 578 | 0.95 (0.61, 1.47) | 0.825 | 236 | 0.97 (0.3, 3.17) | 0.969 | 4469 | 1.01 (0.63, 1.61) | 0.97 |
| 569.1 | Toxic gastroenteritis and colitis | 409 | 17629444 | 177 | 1 (0.98, 1.02) | 0.97 | 44 | 0.96 (0.11, 7.97) | 0.97 | 20 | 1.09 (0.02, 71.08) | 0.97 | 341 | 0.93 (0.11, 8) | 0.955 |
| 569.2 | Gastrointestinal complications | 1263 | 17625436 | 525 | 0.91 (0.71, 1.16) | 0.448 | 145 | 0.96 (0.37, 2.51) | 0.946 | 45 | 0.75 (0.44, 1.28) | 0.297 | 1064 | 0.99 (0.77, 1.28) | 0.97 |
| 571 | Chronic liver disease and cirrhosis | 6850 | 17593413 | 2868 | 0.95 (0.83, 1.09) | 0.458 | 830 | 1.06 (0.83, 1.37) | 0.644 | 293 | 0.94 (0.51, 1.74) | 0.859 | 5751 | 0.98 (0.68, 1.39) | 0.901 |
| 571.5 | Other chronic nonalcoholic liver disease | 1524 | 17617333 | 595 | 0.87 (0.45, 1.67) | 0.684 | 201 | 1.11 (0.25, 4.92) | 0.897 | 76 | 1.06 (0.06, 18.42) | 0.97 | 1286 | 1 (0.9, 1.1) | 0.97 |
| 571.51 | Cirrhosis of liver without mention of alcohol | 2298 | 17619975 | 977 | 0.96 (0.52, 1.75) | 0.898 | 266 | 1.02 (0.34, 3.05) | 0.97 | 94 | 0.9 (0.22, 3.75) | 0.898 | 1924 | 0.96 (0.41, 2.26) | 0.938 |
| 571.6 | Primary biliary cirrhosis | 437 | 17628950 | 182 | 0.87 (0.33, 2.31) | 0.791 | 43 | 0.79 (0.17, 3.63) | 0.771 | 12 | 0.55 (0.07, 4.3) | 0.58 | 370 | 1.03 (0.27, 3.97) | 0.97 |
| 571.8 | Liver abscess and sequelae of chronic liver disease | 3918 | 17619257 | 1665 | 0.98 (0.73, 1.31) | 0.883 | 477 | 1.09 (0.89, 1.34) | 0.432 | 168 | 0.96 (0.45, 2.05) | 0.929 | 3289 | 0.98 (0.68, 1.4) | 0.906 |
| 571.81 | Portal hypertension | 1101 | 17627608 | 463 | 0.94 (0.67, 1.3) | 0.709 | 141 | 1.11 (0.69, 1.79) | 0.691 | 31 | **0.61 (0.4, 0.94)** | **0.026** | 926 | 0.99 (0.52, 1.88) | 0.97 |
| 573 | Other disorders of liver | 4826 | 17598998 | 2071 | 0.99 (0.64, 1.53) | 0.958 | 572 | 1.04 (0.67, 1.62) | 0.864 | 191 | 0.88 (0.59, 1.3) | 0.527 | 4085 | 1.02 (0.66, 1.58) | 0.923 |
| 573.2 | Liver replaced by transplant | 285 | 17630274 | 129 | 1.11 (0.38, 3.19) | 0.86 | 38 | 1.18 (0.31, 4.45) | 0.817 | 7 | 0.57 (0.09, 3.39) | 0.544 | 241 | 0.99 (0.71, 1.4) | 0.97 |
| 573.3 | Hepatomegaly | 449 | 17627418 | 181 | 0.86 (0.48, 1.57) | 0.644 | 48 | 0.87 (0.2, 3.74) | 0.859 | 21 | 0.96 (0.15, 6.36) | 0.97 | 383 | 1.07 (0.25, 4.67) | 0.932 |
| 573.7 | Abnormal results of function study of liver | 108 | 17631001 | 41 | 0.8 (0.24, 2.63) | 0.724 | 17 | 1.25 (0.13, 11.58) | 0.856 | 1 | 0.19 (0, 11.15) | 0.428 | 89 | 0.86 (0.1, 7.43) | 0.901 |
| 573.9 | Abnormal serum enzyme levels | 842 | 17626182 | 360 | 1 (0.99, 1.01) | 0.97 | 111 | 1.22 (0.93, 1.61) | 0.142 | 33 | 0.9 (0.26, 3.16) | 0.883 | 714 | 1.05 (0.53, 2.09) | 0.897 |
| 574 | Cholelithiasis and cholecystitis | 31530 | 17299206 | 13940 | **1.05 (1, 1.11)** | **0.045** | 3533 | 1 (0.83, 1.21) | 0.97 | 1409 | 1.02 (0.6, 1.73) | 0.958 | 26641 | 1.01 (0.77, 1.32) | 0.949 |
| 574.1 | Cholelithiasis | 24028 | 17364917 | 10616 | 1.05 (0.96, 1.15) | 0.254 | 2697 | 1 (0.92, 1.1) | 0.97 | 1070 | 1.01 (0.64, 1.6) | 0.97 | 20299 | 1.01 (0.75, 1.35) | 0.97 |
| 574.11 | Cholelithiasis with acute cholecystitis | 3331 | 17604919 | 1490 | 1.09 (0.94, 1.27) | 0.266 | 376 | 1.05 (0.6, 1.84) | 0.885 | 165 | 1.16 (0.77, 1.75) | 0.487 | 2809 | 1 (0.96, 1.04) | 0.97 |
| 574.12 | Cholelithiasis with other cholecystitis | 2650 | 17605642 | 1216 | 1.1 (0.94, 1.27) | 0.231 | 268 | 0.92 (0.62, 1.37) | 0.7 | 108 | 0.94 (0.37, 2.37) | 0.897 | 2220 | 0.96 (0.6, 1.55) | 0.883 |
| 574.2 | Calculus of bile duct | 8401 | 17560937 | 3755 | 1.07 (0.99, 1.15) | 0.076 | 921 | 0.99 (0.6, 1.62) | 0.958 | 370 | 1 (0.81, 1.24) | 0.97 | 7157 | 1.07 (0.94, 1.21) | 0.323 |
| 574.3 | Cholecystitis without cholelithiasis | 5707 | 17578328 | 2532 | 1.05 (0.89, 1.25) | 0.573 | 649 | 1.04 (0.67, 1.62) | 0.863 | 240 | 0.97 (0.39, 2.41) | 0.947 | 4814 | 1.01 (0.63, 1.63) | 0.97 |
| 575 | Other biliary tract disease | 4228 | 17609145 | 1920 | 1.08 (0.95, 1.24) | 0.252 | 458 | 1 (0.96, 1.04) | 0.97 | 168 | 0.92 (0.47, 1.81) | 0.817 | 3592 | 1.06 (0.79, 1.42) | 0.73 |
| 575.1 | Cholangitis | 1592 | 17624906 | 708 | 1.03 (0.57, 1.85) | 0.931 | 168 | 0.94 (0.41, 2.16) | 0.901 | 64 | 0.9 (0.26, 3.19) | 0.883 | 1362 | 1.11 (0.74, 1.65) | 0.624 |
| 575.2 | Obstruction of bile duct | 1593 | 17626684 | 779 | **1.29 (1.13, 1.48)** | **<0.001** | 171 | 1.12 (0.64, 1.95) | 0.697 | 73 | 1.19 (0.56, 2.53) | 0.664 | 1359 | 1.1 (0.7, 1.71) | 0.7 |
| 575.7 | Other disorders of gallbladder | 543 | 17628914 | 251 | 1.05 (0.39, 2.8) | 0.929 | 47 | 0.74 (0.36, 1.54) | 0.431 | 17 | 0.68 (0.18, 2.55) | 0.578 | 451 | 0.91 (0.33, 2.5) | 0.861 |
| 575.8 | Other disorders of biliary tract | 1262 | 17621209 | 532 | 0.95 (0.53, 1.69) | 0.875 | 143 | 0.97 (0.26, 3.58) | 0.97 | 54 | 0.93 (0.19, 4.41) | 0.929 | 1073 | 1.05 (0.49, 2.29) | 0.901 |
| 577 | Diseases of pancreas | 3101 | 17603838 | 1409 | 1.13 (0.94, 1.36) | 0.186 | 375 | 1.18 (0.86, 1.61) | 0.315 | 142 | 1.12 (0.45, 2.79) | 0.827 | 2614 | 1.01 (0.79, 1.29) | 0.97 |
| 577.1 | Acute pancreatitis | 151 | 17627509 | 56 | 0.8 (0.34, 1.84) | 0.607 | 20 | 1.13 (0.12, 10.41) | 0.92 | 9 | 1.27 (0.07, 23.57) | 0.883 | 126 | 0.95 (0.09, 10.45) | 0.97 |
| 577.2 | Chronic pancreatitis | 2170 | 17612028 | 981 | 1.11 (0.9, 1.37) | 0.325 | 265 | 1.17 (0.86, 1.61) | 0.323 | 93 | 1.03 (0.23, 4.59) | 0.97 | 1822 | 0.98 (0.38, 2.54) | 0.97 |
| 577.3 | Cyst and pseudocyst of pancreas | 739 | 17626529 | 347 | 1.22 (0.7, 2.12) | 0.493 | 84 | 1.15 (0.21, 6.18) | 0.883 | 39 | 1.34 (0.28, 6.3) | 0.725 | 630 | 1.08 (0.22, 5.28) | 0.93 |
| 578 | Gastrointestinal hemorrhage | 20111 | 17502200 | 8543 | **0.94 (0.89, 0.99)** | **0.014** | 2141 | **0.91 (0.84, 0.99)** | **0.027** | 824 | 0.88 (0.77, 1) | 0.055 | 16975 | 1.01 (0.79, 1.3) | 0.923 |
| 578.1 | Hematemesis | 5357 | 17597898 | 2271 | 0.94 (0.79, 1.12) | 0.503 | 592 | 0.94 (0.62, 1.45) | 0.804 | 211 | 0.85 (0.57, 1.25) | 0.413 | 4525 | 1.02 (0.54, 1.91) | 0.966 |
| 578.2 | Blood in stool | 3161 | 17614407 | 1352 | 0.93 (0.7, 1.22) | 0.611 | 307 | 0.84 (0.6, 1.18) | 0.323 | 131 | 0.89 (0.35, 2.27) | 0.818 | 2668 | 1.03 (0.46, 2.31) | 0.952 |
| 578.8 | Hemorrhage of rectum and anus | 11095 | 17555331 | 4689 | **0.93 (0.87, 1)** | **0.038** | 1171 | **0.89 (0.8, 1)** | **0.047** | 457 | 0.87 (0.71, 1.07) | 0.201 | 9357 | 1 (0.8, 1.26) | 0.97 |
| 578.9 | Hemorrhage of gastrointestinal tract | 3431 | 17618100 | 1512 | 0.98 (0.7, 1.38) | 0.917 | 330 | 0.85 (0.71, 1) | 0.05 | 135 | 0.86 (0.58, 1.27) | 0.457 | 2897 | 1.02 (0.65, 1.62) | 0.923 |
| 579 | Other symptoms involving abdomen and pelvis | 1352 | 17623608 | 569 | 0.98 (0.32, 3.01) | 0.97 | 168 | 1.08 (0.34, 3.4) | 0.909 | 60 | 0.98 (0.36, 2.68) | 0.97 | 1131 | 0.94 (0.36, 2.44) | 0.906 |
| 579.2 | Splenomegaly | 549 | 17627671 | 234 | 0.98 (0.4, 2.41) | 0.97 | 63 | 0.97 (0.28, 3.35) | 0.97 | 25 | 0.99 (0.62, 1.58) | 0.97 | 454 | 0.87 (0.25, 3.09) | 0.842 |
| 579.8 | Nonspecific abnormal findings in stool contents | 557 | 17629915 | 228 | 0.95 (0.51, 1.75) | 0.873 | 72 | 1.13 (0.62, 2.06) | 0.714 | 28 | 1.11 (0.29, 4.25) | 0.885 | 461 | 0.89 (0.57, 1.37) | 0.596 |
| **Genitourinary** | | | | | | | | | | | | | | | |
| 580 | Nephritis; nephrosis; renal sclerosis | 2488 | 17608885 | 1048 | 0.96 (0.59, 1.55) | 0.874 | 285 | 0.97 (0.36, 2.6) | 0.959 | 116 | 1.01 (0.68, 1.49) | 0.97 | 2102 | 1 (0.95, 1.05) | 0.97 |
| 580.11 | Proliferative glomerulonephritis | 553 | 17626842 | 238 | 0.97 (0.25, 3.67) | 0.963 | 58 | 0.86 (0.24, 3.11) | 0.832 | 22 | 0.84 (0.09, 7.89) | 0.887 | 463 | 0.94 (0.25, 3.56) | 0.93 |
| 580.12 | Non-proliferative glomerulonephritis | 568 | 17626417 | 256 | 1.08 (0.33, 3.5) | 0.905 | 64 | 0.98 (0.32, 3.02) | 0.97 | 22 | 0.87 (0.03, 22.96) | 0.938 | 502 | 1.38 (0.62, 3.09) | 0.443 |
| 580.14 | Chronic glomerulonephritis, NOS | 579 | 17625196 | 233 | 0.89 (0.46, 1.71) | 0.734 | 71 | 1 (0.89, 1.11) | 0.97 | 25 | 0.9 (0.05, 15.43) | 0.947 | 480 | 0.88 (0.34, 2.27) | 0.809 |
| 580.2 | Nephrotic syndrome without mention of glomerulonephritis | 916 | 17624896 | 403 | 1.05 (0.46, 2.39) | 0.911 | 108 | 1.05 (0.2, 5.47) | 0.959 | 41 | 1.02 (0.45, 2.28) | 0.97 | 781 | 1.06 (0.32, 3.49) | 0.929 |
| 580.32 | Nephritis and nephropathy with pathological lesion | 879 | 17624808 | 361 | 0.87 (0.51, 1.49) | 0.629 | 85 | 0.77 (0.35, 1.7) | 0.536 | 40 | 0.92 (0.06, 14.44) | 0.959 | 734 | 0.93 (0.27, 3.18) | 0.921 |
| 585 | Renal failure | 14334 | 17580942 | 6152 | 0.98 (0.85, 1.12) | 0.767 | 1619 | 1.03 (0.77, 1.37) | 0.877 | 642 | 1.01 (0.76, 1.32) | 0.97 | 12081 | 1.01 (0.77, 1.34) | 0.929 |
| 585.1 | Acute renal failure | 6877 | 17611408 | 2933 | 0.97 (0.7, 1.34) | 0.863 | 778 | 1.02 (0.53, 1.98) | 0.949 | 319 | 1.04 (0.43, 2.55) | 0.934 | 5834 | 1.06 (0.79, 1.41) | 0.715 |
| 585.3 | Chronic renal failure [CKD] | 9217 | 17596842 | 3990 | 0.99 (0.78, 1.26) | 0.949 | 1037 | 1.03 (0.8, 1.33) | 0.836 | 402 | 0.98 (0.52, 1.87) | 0.966 | 7717 | 0.97 (0.82, 1.16) | 0.773 |
| 585.31 | Renal dialysis | 1893 | 17625158 | 774 | 0.88 (0.75, 1.03) | 0.114 | 214 | 0.95 (0.48, 1.86) | 0.885 | 76 | 0.84 (0.45, 1.58) | 0.609 | 1596 | 1 (0.87, 1.16) | 0.97 |
| 586 | Other disorders of the kidney and ureters | 6374 | 17572477 | 2679 | 0.95 (0.3, 3.05) | 0.942 | 731 | 1 (0.83, 1.22) | 0.97 | 301 | 1.04 (0.2, 5.48) | 0.97 | 5427 | 1.07 (0.26, 4.46) | 0.932 |
| 586.11 | Small kidney | 141 | 17630811 | 61 | 0.98 (0.33, 2.88) | 0.97 | 15 | 0.89 (0.01, 124.8) | 0.965 | 5 | 0.75 (0, 394.56) | 0.935 | 119 | 0.99 (0.68, 1.46) | 0.97 |
| 586.2 | Cyst of kidney, acquired | 721 | 17625589 | 314 | 0.99 (0.76, 1.3) | 0.97 | 73 | 0.91 (0.12, 6.85) | 0.935 | 36 | 1.12 (0.06, 20.43) | 0.945 | 604 | 0.97 (0.28, 3.34) | 0.97 |
| 586.4 | Stricture/obstruction of ureter | 2217 | 17610778 | 883 | 0.87 (0.71, 1.07) | 0.18 | 249 | 0.93 (0.43, 2.02) | 0.864 | 121 | 1.14 (0.51, 2.56) | 0.761 | 1879 | 1.03 (0.4, 2.65) | 0.959 |
| 587 | Kidney replaced by transpant | 660 | 17626751 | 282 | 1.03 (0.35, 3.02) | 0.958 | 79 | 1.06 (0.28, 4.1) | 0.933 | 39 | 1.35 (0.63, 2.87) | 0.448 | 549 | 0.9 (0.44, 1.83) | 0.784 |
| 588 | Disorders resulting from impaired renal function | 618 | 17628310 | 244 | 0.84 (0.49, 1.43) | 0.534 | 68 | 0.91 (0.17, 4.87) | 0.92 | 31 | 1.04 (0.19, 5.7) | 0.97 | 513 | 0.91 (0.26, 3.2) | 0.896 |
| 590 | Pyelonephritis | 2785 | 17580483 | 1194 | 0.99 (0.71, 1.39) | 0.97 | 305 | 0.9 (0.59, 1.38) | 0.648 | 127 | 0.98 (0.34, 2.78) | 0.97 | 2377 | 1.05 (0.6, 1.84) | 0.87 |
| 591 | Urinary tract infection | 23713 | 17512977 | 10250 | 1.01 (0.88, 1.17) | 0.885 | 2664 | 1.01 (0.79, 1.3) | 0.924 | 1140 | 1.09 (0.98, 1.22) | 0.126 | 20072 | 1.03 (0.94, 1.14) | 0.487 |
| 592 | Cystitis and urethritis | 48623 | 17267909 | 20763 | 0.99 (0.84, 1.18) | 0.956 | 5611 | 1 (0.89, 1.12) | 0.97 | 2275 | 1.03 (0.85, 1.26) | 0.763 | 41079 | 1.01 (0.82, 1.24) | 0.959 |
| 592.1 | Cystitis | 17192 | 17525973 | 7420 | 1.01 (0.82, 1.25) | 0.935 | 1974 | 1.01 (0.67, 1.53) | 0.965 | 779 | 1.01 (0.61, 1.68) | 0.97 | 14533 | 1.01 (0.77, 1.33) | 0.933 |
| 592.11 | Acute cystitis | 34975 | 17383796 | 14858 | 0.99 (0.85, 1.14) | 0.883 | 4072 | 1 (0.92, 1.09) | 0.97 | 1650 | 1.04 (0.81, 1.33) | 0.783 | 29552 | 1 (0.82, 1.24) | 0.97 |
| 592.12 | Chronic cystitis | 1502 | 17610152 | 641 | 0.95 (0.56, 1.61) | 0.866 | 153 | 0.87 (0.51, 1.49) | 0.626 | 66 | 0.94 (0.17, 5.25) | 0.952 | 1243 | 0.9 (0.61, 1.33) | 0.6 |
| 592.13 | Chronic interstitial cystitis | 1242 | 17618026 | 535 | 0.99 (0.65, 1.51) | 0.97 | 137 | 0.95 (0.39, 2.36) | 0.924 | 53 | 0.93 (0.23, 3.78) | 0.931 | 1074 | 1.19 (0.9, 1.57) | 0.236 |
| 592.2 | Urethritis and urethral syndrome | 527 | 17621209 | 198 | 0.81 (0.52, 1.27) | 0.365 | 65 | 0.99 (0.61, 1.62) | 0.97 | 32 | 1.25 (0.28, 5.46) | 0.784 | 443 | 0.96 (0.18, 5.23) | 0.97 |
| 592.3 | Urethral stricture due to infecton | 165 | 17629196 | 79 | 1.12 (0.13, 9.36) | 0.921 | 15 | 0.83 (0.02, 40.72) | 0.933 | 5 | 0.69 (0, 163.16) | 0.904 | 137 | 0.92 (0.03, 31.18) | 0.965 |
| 593 | Hematuria | 494 | 17626549 | 209 | 0.92 (0.4, 2.1) | 0.859 | 56 | 0.93 (0.2, 4.48) | 0.938 | 14 | 0.59 (0.2, 1.75) | 0.347 | 426 | 1.16 (0.54, 2.53) | 0.714 |
| 594 | Urinary calculus | 20964 | 17385879 | 8969 | 0.98 (0.88, 1.08) | 0.672 | 2348 | 0.96 (0.82, 1.13) | 0.648 | 950 | 0.99 (0.63, 1.57) | 0.97 | 17837 | 1.06 (0.97, 1.14) | 0.19 |
| 594.1 | Calculus of kidney | 11345 | 17527685 | 4826 | 0.97 (0.82, 1.16) | 0.784 | 1305 | 0.98 (0.64, 1.52) | 0.949 | 516 | 0.99 (0.74, 1.34) | 0.97 | 9699 | 1.08 (0.98, 1.2) | 0.117 |
| 594.2 | Calculus of lower urinary tract | 2146 | 17608353 | 948 | 0.99 (0.77, 1.28) | 0.97 | 211 | 0.87 (0.49, 1.56) | 0.661 | 92 | 0.95 (0.15, 5.91) | 0.957 | 1815 | 1.04 (0.43, 2.51) | 0.942 |
| 594.3 | Calculus of ureter | 11775 | 17482575 | 5025 | 0.97 (0.85, 1.1) | 0.654 | 1330 | 0.97 (0.72, 1.32) | 0.864 | 522 | 0.97 (0.59, 1.57) | 0.902 | 9991 | 1.04 (0.87, 1.24) | 0.7 |
| 595 | Hydronephrosis | 5889 | 17594714 | 2497 | 0.95 (0.77, 1.17) | 0.63 | 620 | 0.9 (0.67, 1.21) | 0.512 | 271 | 0.99 (0.7, 1.41) | 0.97 | 4952 | 0.98 (0.5, 1.95) | 0.968 |
| 596 | Other disorders of bladder | 4506 | 17581848 | 1920 | 0.94 (0.8, 1.12) | 0.512 | 463 | 0.88 (0.71, 1.08) | 0.23 | 199 | 0.95 (0.44, 2.04) | 0.902 | 3789 | 0.99 (0.58, 1.69) | 0.97 |
| 596.1 | Bladder neck obstruction | 565 | 17622128 | 247 | 0.98 (0.39, 2.46) | 0.97 | 56 | 0.89 (0.19, 4.27) | 0.897 | 27 | 1.06 (0.06, 18.98) | 0.97 | 474 | 0.99 (0.68, 1.44) | 0.97 |
| 596.5 | Functional disorders of bladder | 2563 | 17604520 | 1071 | 0.91 (0.75, 1.1) | 0.323 | 261 | 0.84 (0.64, 1.11) | 0.234 | 114 | 0.93 (0.34, 2.57) | 0.898 | 2160 | 1 (0.81, 1.23) | 0.97 |
| 597 | Other disorders of urethra and urinary tract | 3706 | 17584708 | 1619 | 1.02 (0.54, 1.94) | 0.947 | 415 | 1.02 (0.37, 2.84) | 0.97 | 182 | 1.12 (0.46, 2.72) | 0.81 | 3142 | 1.05 (0.54, 2.01) | 0.901 |
| 597.1 | Urethral stricture (not specified as infectious) | 2682 | 17597294 | 1179 | 1.03 (0.54, 1.96) | 0.93 | 299 | 1.03 (0.28, 3.77) | 0.97 | 133 | 1.14 (0.43, 3.04) | 0.802 | 2267 | 1.03 (0.38, 2.75) | 0.959 |
| 598 | Abnormal findings on examination of urine | 1199 | 17623632 | 503 | 0.95 (0.55, 1.63) | 0.861 | 134 | 0.98 (0.34, 2.85) | 0.97 | 63 | 1.15 (0.48, 2.8) | 0.763 | 1008 | 0.98 (0.44, 2.18) | 0.97 |
| 598.9 | Other nonspecific findings on examination of urine | 108 | 17630344 | 47 | 1.15 (0.15, 8.97) | 0.902 | 15 | 1.36 (0.14, 13.5) | 0.805 | 7 | 1.62 (0.12, 21.91) | 0.73 | 94 | 1.23 (0.09, 17.59) | 0.887 |
| 599 | Other symptoms/disorders or the urinary system | 31107 | 17429032 | 13384 | 0.98 (0.91, 1.06) | 0.668 | 3526 | 1.02 (0.86, 1.21) | 0.864 | 1354 | 0.97 (0.78, 1.21) | 0.8 | 26257 | 1.02 (0.92, 1.13) | 0.745 |
| 599.1 | Urinary obstruction | 365 | 17629743 | 162 | 1.08 (0.41, 2.89) | 0.882 | 39 | 1.01 (0.62, 1.66) | 0.97 | 23 | 1.49 (0.65, 3.4) | 0.348 | 307 | 0.99 (0.77, 1.29) | 0.97 |
| 599.2 | Retention of urine | 681 | 17627138 | 283 | 0.9 (0.67, 1.22) | 0.514 | 67 | 0.86 (0.5, 1.5) | 0.611 | 38 | 1.2 (0.59, 2.46) | 0.629 | 562 | 0.9 (0.58, 1.41) | 0.671 |
| 599.3 | Dysuria | 1738 | 17621389 | 720 | 0.95 (0.59, 1.51) | 0.825 | 216 | 1.02 (0.34, 3.05) | 0.97 | 72 | 0.88 (0.28, 2.73) | 0.836 | 1472 | 1 (0.86, 1.17) | 0.97 |
| 599.4 | Urinary incontinence | 8936 | 17559956 | 3824 | 0.98 (0.79, 1.23) | 0.898 | 1032 | 1.02 (0.73, 1.43) | 0.901 | 354 | 0.87 (0.72, 1.05) | 0.149 | 7503 | 0.98 (0.75, 1.28) | 0.885 |
| 599.8 | Other symptoms involving urinary system | 1018 | 17627435 | 414 | 0.88 (0.61, 1.25) | 0.474 | 121 | 1.02 (0.42, 2.47) | 0.97 | 38 | 0.79 (0.25, 2.47) | 0.7 | 862 | 1.05 (0.35, 3.1) | 0.942 |
| 599.9 | Other abnormality of urination | 17400 | 17530657 | 7513 | 0.99 (0.87, 1.12) | 0.834 | 1967 | 1.03 (0.89, 1.2) | 0.695 | 792 | 1.03 (0.75, 1.42) | 0.866 | 14758 | 1.06 (0.99, 1.13) | 0.076 |
| 601 | Inflammatory diseases of prostate | 3245 | 7079805 | 1400 | 0.99 (0.55, 1.77) | 0.965 | 369 | 0.99 (0.68, 1.44) | 0.97 | 144 | 0.98 (0.41, 2.36) | 0.97 | 2755 | 1.04 (0.63, 1.73) | 0.888 |
| 601.1 | Prostatitis | 380 | 7116650 | 164 | 0.96 (0.16, 5.67) | 0.97 | 42 | 0.97 (0.22, 4.18) | 0.97 | 15 | 0.87 (0.01, 74.24) | 0.955 | 329 | 1.21 (0.28, 5.22) | 0.809 |
| 601.11 | Acute prostatitis | 360 | 7116036 | 152 | 0.91 (0.21, 3.88) | 0.91 | 37 | 0.89 (0.07, 12) | 0.935 | 16 | 0.96 (0.11, 8.25) | 0.97 | 306 | 1.07 (0.09, 13.3) | 0.962 |
| 601.12 | Chronic prostatitis | 210 | 7117344 | 91 | 0.96 (0.18, 5.26) | 0.97 | 22 | 0.92 (0.05, 16.39) | 0.959 | 9 | 0.94 (0.06, 15.37) | 0.97 | 184 | 1.33 (0.45, 4) | 0.62 |
| 601.3 | Orchitis and epididymitis | 239 | 7113556 | 123 | 1.33 (0.88, 2.02) | 0.173 | 26 | 1.11 (0.16, 7.75) | 0.921 | 4 | 0.43 (0.07, 2.61) | 0.364 | 193 | 0.79 (0.41, 1.53) | 0.492 |
| 601.4 | Balanoposthitis | 630 | 7111794 | 254 | 0.86 (0.53, 1.41) | 0.575 | 69 | 0.85 (0.31, 2.32) | 0.771 | 30 | 0.96 (0.15, 6.07) | 0.97 | 540 | 1.08 (0.34, 3.47) | 0.903 |
| 601.8 | Other inflammatory disorders of male genital organs | 1541 | 7104141 | 672 | 1.03 (0.54, 1.98) | 0.933 | 183 | 1.07 (0.45, 2.54) | 0.896 | 74 | 1.09 (0.31, 3.86) | 0.898 | 1303 | 1.01 (0.63, 1.62) | 0.97 |
| 602 | Other disorders of prostate | 240 | 7117705 | 102 | 0.99 (0.57, 1.73) | 0.97 | 29 | 1.15 (0.27, 4.91) | 0.861 | 13 | 1.26 (0.23, 7) | 0.806 | 194 | 0.81 (0.44, 1.49) | 0.503 |
| 603 | Other disorders of testis | 10409 | 6989610 | 4533 | 1 (0.95, 1.06) | 0.97 | 1174 | 0.99 (0.76, 1.3) | 0.97 | 455 | 0.97 (0.52, 1.81) | 0.934 | 8760 | 0.98 (0.71, 1.36) | 0.931 |
| 603.1 | Hydrocele | 2976 | 7089986 | 1261 | 0.96 (0.71, 1.3) | 0.802 | 345 | 1.01 (0.57, 1.8) | 0.97 | 139 | 1.03 (0.28, 3.83) | 0.969 | 2532 | 1.06 (0.74, 1.51) | 0.761 |
| 603.2 | Spermatocele | 2308 | 7088681 | 1031 | 1.05 (0.74, 1.48) | 0.8 | 249 | 0.99 (0.51, 1.91) | 0.97 | 108 | 1.07 (0.39, 2.93) | 0.897 | 1925 | 0.94 (0.63, 1.39) | 0.767 |
| 604 | Disorders of penis | 1445 | 7105718 | 625 | 0.99 (0.69, 1.43) | 0.97 | 163 | 1 (0.88, 1.14) | 0.97 | 68 | 1.06 (0.15, 7.64) | 0.96 | 1211 | 0.96 (0.34, 2.72) | 0.947 |
| 604.1 | Redundant prepuce and phimosis/BXO | 838 | 7112041 | 380 | 1.1 (0.55, 2.2) | 0.806 | 97 | 1.1 (0.28, 4.33) | 0.898 | 38 | 1.09 (0.07, 16.04) | 0.957 | 700 | 0.95 (0.26, 3.52) | 0.944 |
| 605 | Erectile dysfunction [ED] | 2185 | 7100871 | 933 | 0.97 (0.66, 1.41) | 0.873 | 262 | 1.08 (0.74, 1.58) | 0.708 | 92 | 0.95 (0.35, 2.54) | 0.92 | 1870 | 1.12 (0.9, 1.39) | 0.327 |
| 608 | Other disorders of male genital organs | 4261 | 7065803 | 1876 | 1.03 (0.75, 1.41) | 0.868 | 485 | 1.01 (0.74, 1.38) | 0.97 | 187 | 0.99 (0.53, 1.85) | 0.97 | 3596 | 0.99 (0.78, 1.27) | 0.97 |
| 610 | Benign mammary dysplasias | 11509 | 10305207 | 5063 | 1.03 (0.92, 1.16) | 0.604 | 1235 | 0.95 (0.79, 1.14) | 0.574 | 482 | 0.93 (0.7, 1.24) | 0.629 | 9652 | 0.96 (0.82, 1.13) | 0.671 |
| 610.1 | Cystic mastopathy | 2839 | 10479085 | 1239 | 1.01 (0.53, 1.93) | 0.97 | 297 | 0.89 (0.62, 1.28) | 0.543 | 120 | 0.92 (0.36, 2.32) | 0.863 | 2379 | 0.95 (0.61, 1.47) | 0.826 |
| 610.2 | Fibroadenosis of breast | 8117 | 10345726 | 3602 | 1.05 (0.96, 1.15) | 0.307 | 880 | 0.98 (0.68, 1.4) | 0.906 | 334 | 0.93 (0.67, 1.29) | 0.661 | 6795 | 0.96 (0.81, 1.13) | 0.624 |
| 610.3 | Fibrosclerosis of breast | 122 | 10509912 | 51 | 0.9 (0.1, 8.27) | 0.929 | 11 | 0.72 (0.04, 11.56) | 0.827 | 5 | 0.83 (0, 572.27) | 0.958 | 105 | 1.14 (0.06, 22.11) | 0.936 |
| 610.4 | Benign neoplasm of breast | 379 | 10500069 | 159 | 0.91 (0.35, 2.41) | 0.864 | 35 | 0.8 (0.25, 2.58) | 0.727 | 19 | 1.07 (0.05, 22.63) | 0.97 | 305 | 0.79 (0.44, 1.41) | 0.428 |
| 610.8 | Other specified benign mammary dysplasias | 1067 | 10501476 | 455 | 0.98 (0.46, 2.09) | 0.955 | 111 | 0.9 (0.49, 1.65) | 0.741 | 51 | 1.03 (0.21, 5.01) | 0.97 | 915 | 1.12 (0.72, 1.74) | 0.635 |
| 612 | Breast conditions, congenital or relating to hormones | 1502 | 17618278 | 637 | 1.01 (0.53, 1.94) | 0.97 | 176 | 0.93 (0.56, 1.54) | 0.791 | 75 | 1.05 (0.36, 3.08) | 0.929 | 1285 | 1.04 (0.58, 1.86) | 0.915 |
| 612.1 | Galactorrhea | 304 | 17627758 | 136 | 1.05 (0.42, 2.59) | 0.93 | 30 | 0.83 (0.35, 1.98) | 0.695 | 12 | 0.86 (0.12, 5.95) | 0.883 | 256 | 0.97 (0.26, 3.64) | 0.97 |
| 612.3 | Congenital anomalies of breast | 1201 | 482914* | 502 | 1.01 (0.65, 1.56)** | 0.97 | 146 | 0.95 (0.39, 2.29)** | 0.909 | 64 | 1.12 (0.35, 3.58)** | 0.859 | 1032 | 1.05 (0.48, 2.31)** | 0.908 |
| 613 | Other nonmalignant breast conditions | 7317 | 17570383 | 3128 | 1.02 (0.79, 1.31) | 0.898 | 866 | 0.97 (0.67, 1.4) | 0.885 | 331 | 0.97 (0.48, 1.98) | 0.949 | 6127 | 0.91 (0.83, 1.01) | 0.087 |
| 613.1 | Inflammatory disease of breast | 310 | 17627823 | 140 | 1.05 (0.21, 5.11) | 0.959 | 28 | 0.78 (0.19, 3.21) | 0.743 | 13 | 0.92 (0.01, 60.13) | 0.97 | 255 | 0.86 (0.26, 2.78) | 0.806 |
| 613.5 | Mastodynia | 1581 | 17619013 | 652 | 0.97 (0.64, 1.49) | 0.914 | 206 | 1.07 (0.68, 1.67) | 0.791 | 72 | 0.98 (0.35, 2.77) | 0.97 | 1313 | 0.87 (0.74, 1.03) | 0.114 |
| 613.7 | Other signs and symptoms in breast | 1868 | 17618618 | 813 | 1.07 (0.86, 1.32) | 0.57 | 225 | 1.05 (0.58, 1.89) | 0.883 | 90 | 1.09 (0.52, 2.26) | 0.835 | 1579 | 0.99 (0.53, 1.84) | 0.97 |
| 613.8 | Other specified disorders of breast | 1394 | 17622578 | 612 | 1.03 (0.6, 1.78) | 0.914 | 143 | 0.81 (0.59, 1.12) | 0.203 | 60 | 0.91 (0.26, 3.1) | 0.883 | 1152 | 0.84 (0.67, 1.04) | 0.114 |
| 613.9 | Breast disorder NOS | 751 | 17616950 | 313 | 1.05 (0.43, 2.57) | 0.921 | 110 | 1.37 (0.93, 2.02) | 0.116 | 41 | 1.29 (0.48, 3.46) | 0.62 | 625 | 0.91 (0.38, 2.17) | 0.843 |
| 614 | Inflammatory diseases of female pelvic organs | 19463 | 10152172 | 8204 | 1 (0.94, 1.06) | 0.97 | 2332 | 1 (0.93, 1.08) | 0.97 | 930 | 1.04 (0.76, 1.41) | 0.826 | 16526 | 1.01 (0.77, 1.33) | 0.947 |
| 614.1 | Pelvic peritoneal adhesions, female (postoperative) (postinfection) | 1281 | 10484656 | 561 | 1.1 (0.85, 1.43) | 0.455 | 152 | 1.1 (0.63, 1.9) | 0.751 | 76 | **1.4 (1.02, 1.93)** | **0.038** | 1086 | 1.02 (0.42, 2.45) | 0.97 |
| 614.3 | Pelvic inflammatory disease (PID) | 688 | 10499106 | 275 | 0.87 (0.62, 1.23) | 0.439 | 75 | 0.82 (0.47, 1.43) | 0.487 | 30 | 0.86 (0.19, 3.82) | 0.852 | 582 | 0.97 (0.29, 3.25) | 0.97 |
| 614.31 | Acute inflammatory pelvic disease | 5386 | 10374182 | 2361 | 1.05 (0.18, 6.12) | 0.958 | 593 | 0.95 (0.09, 10.38) | 0.97 | 252 | 1.03 (0.24, 4.42) | 0.97 | 4556 | 0.99 (0.67, 1.48) | 0.97 |
| 614.32 | Chronic inflammatory pelvic disease | 2327 | 10462283 | 999 | 1.04 (0.63, 1.71) | 0.898 | 278 | 1.06 (0.51, 2.21) | 0.89 | 116 | 1.12 (0.48, 2.65) | 0.805 | 1941 | 0.92 (0.62, 1.35) | 0.678 |
| 614.33 | Pelvic inflammatory disease, NOS | 554 | 10502914 | 240 | 1.04 (0.26, 4.19) | 0.959 | 73 | 1.12 (0.24, 5.12) | 0.897 | 17 | 0.67 (0.12, 3.82) | 0.668 | 468 | 0.98 (0.33, 2.89) | 0.97 |
| 614.4 | Inflammatory diseases of uterus, except cervix | 5282 | 10421412 | 2243 | 1.01 (0.69, 1.48) | 0.959 | 635 | 0.99 (0.68, 1.44) | 0.97 | 242 | 0.99 (0.6, 1.64) | 0.97 | 4491 | 1.01 (0.61, 1.67) | 0.97 |
| 614.5 | Inflammatory disease of cervix, vagina, and vulva | 534 | 10505645 | 214 | 0.91 (0.42, 2.01) | 0.836 | 66 | 0.97 (0.2, 4.72) | 0.97 | 26 | 1 (0.99, 1.01) | 0.97 | 447 | 0.91 (0.29, 2.85) | 0.881 |
| 614.51 | Cervicitis and endocervicitis | 660 | 10488791 | 279 | 1.02 (0.5, 2.09) | 0.952 | 79 | 1.14 (0.72, 1.8) | 0.6 | 41 | **1.44 (1.05, 1.98)** | **0.025** | 568 | 1.16 (0.86, 1.58) | 0.332 |
| 614.52 | Vaginitis and vulvovaginitis | 1568 | 10491503 | 676 | 1.03 (0.54, 1.97) | 0.933 | 179 | 0.94 (0.39, 2.25) | 0.901 | 74 | 1.02 (0.38, 2.77) | 0.97 | 1339 | 1.04 (0.46, 2.38) | 0.928 |
| 614.53 | Cyst or abscess of Bartholin's gland | 2395 | 10478871 | 939 | 0.89 (0.76, 1.06) | 0.184 | 316 | 1.04 (0.55, 1.98) | 0.903 | 116 | 1 (0.85, 1.18) | 0.97 | 2054 | 1.08 (0.73, 1.58) | 0.725 |
| 614.54 | Abscess or ulceration of vulva | 2899 | 10474068 | 1187 | 0.95 (0.73, 1.23) | 0.7 | 361 | 1.01 (0.73, 1.39) | 0.97 | 133 | 0.97 (0.3, 3.12) | 0.959 | 2469 | 1.03 (0.58, 1.83) | 0.935 |
| 615 | Endometriosis | 6640 | 10418467 | 2894 | 1.05 (0.92, 1.2) | 0.462 | 778 | 0.99 (0.54, 1.8) | 0.97 | 284 | 0.94 (0.56, 1.59) | 0.836 | 5618 | 0.99 (0.63, 1.55) | 0.956 |
| 617 | Disorders secondary to childbirth, surgery, trauma | 726 | 10506293 | 314 | 1.01 (0.78, 1.3) | 0.97 | 74 | 0.85 (0.12, 5.97) | 0.883 | 35 | 1.04 (0.14, 7.65) | 0.97 | 632 | 1.23 (0.4, 3.72) | 0.732 |
| 618 | Genital prolapse | 12139 | 10366245 | 5268 | 1.03 (0.59, 1.81) | 0.921 | 1399 | 1.07 (0.5, 2.27) | 0.87 | 573 | 1.09 (0.34, 3.47) | 0.893 | 10187 | 0.99 (0.49, 1.98) | 0.97 |
| 618.1 | Prolapse of vaginal walls | 9644 | 10399197 | 4181 | 1.02 (0.34, 3.04) | 0.97 | 1095 | 1.06 (0.29, 3.81) | 0.935 | 454 | 1.08 (0.18, 6.56) | 0.935 | 8120 | 1.01 (0.58, 1.77) | 0.97 |
| 618.2 | Uterine/Uterovaginal prolapse | 3351 | 10475337 | 1441 | 1.04 (0.82, 1.33) | 0.751 | 415 | **1.19 (1.02, 1.39)** | **0.027** | 173 | 1.23 (0.94, 1.59) | 0.129 | 2794 | 0.95 (0.69, 1.3) | 0.773 |
| 618.5 | Prolapse of vaginal vault after hysterectomy | 519 | 10508186 | 237 | 1.15 (0.78, 1.71) | 0.48 | 69 | 1.36 (0.91, 2.02) | 0.131 | 17 | 0.82 (0.15, 4.45) | 0.826 | 435 | 0.99 (0.59, 1.67) | 0.97 |
| 618.6 | Vaginal enterocele, congenital or acquired | 2381 | 10484847 | 1053 | 1.05 (0.67, 1.64) | 0.849 | 274 | 1.08 (0.57, 2.03) | 0.824 | 93 | 0.9 (0.27, 3.08) | 0.881 | 1969 | 0.91 (0.65, 1.27) | 0.578 |
| 619 | Noninflammatory female genital disorders | 9706 | 10377939 | 4249 | 1.06 (0.95, 1.19) | 0.312 | 1114 | 0.96 (0.69, 1.33) | 0.8 | 440 | 1 (0.81, 1.22) | 0.97 | 8162 | 0.94 (0.79, 1.12) | 0.503 |
| 619.1 | Noninflammatory disorders of ovary, fallopian tube, and broad ligament | 1612 | 10487115 | 695 | 1.03 (0.58, 1.83) | 0.918 | 191 | 0.99 (0.59, 1.65) | 0.97 | 71 | 0.96 (0.16, 5.68) | 0.966 | 1360 | 0.97 (0.44, 2.1) | 0.934 |
| 619.2 | Disorders of uterus, NEC | 1852 | 10469636 | 815 | 1.03 (0.26, 4.01) | 0.97 | 193 | 0.89 (0.01, 98.61) | 0.965 | 78 | 0.92 (0.02, 43.36) | 0.97 | 1557 | 0.97 (0.22, 4.34) | 0.97 |
| 619.3 | Noninflammatory disorders of cervix | 3229 | 10484043 | 1445 | 1.13 (0, 324.1) | 0.97 | 400 | 1.03 (0.31, 3.42) | 0.97 | 125 | 0.87 (0, 652.14) | 0.97 | 2698 | 0.89 (0, 267.15) | 0.97 |
| 619.4 | Noninflammatory disorders of vagina | 1205 | 10498366 | 548 | 1.14 (0.88, 1.49) | 0.32 | 124 | 0.89 (0.44, 1.83) | 0.767 | 66 | 1.25 (0.63, 2.47) | 0.537 | 1007 | 0.91 (0.55, 1.49) | 0.71 |
| 619.5 | Noninflammatory disorders of vulva and perineum | 1025 | 10503499 | 413 | 0.91 (0.59, 1.42) | 0.695 | 123 | 0.91 (0.37, 2.21) | 0.843 | 46 | 0.9 (0.19, 4.36) | 0.909 | 860 | 0.91 (0.47, 1.79) | 0.805 |
| 620 | Dysplasia of female genital organs | 682 | 10505987 | 281 | 0.92 (0.51, 1.67) | 0.806 | 86 | 1.05 (0.24, 4.56) | 0.955 | 21 | 0.65 (0.27, 1.53) | 0.327 | 551 | 0.77 (0.58, 1.02) | 0.07 |
| 621 | Endometrial hyperplasia | 1016 | 10497703 | 458 | 1.07 (0.59, 1.93) | 0.834 | 117 | 1.07 (0.36, 3.18) | 0.906 | 31 | 0.7 (0.28, 1.77) | 0.458 | 849 | 0.96 (0.32, 2.87) | 0.949 |
| 622 | Polyp of female genital organs | 9045 | 10434001 | 3864 | 1.01 (0.68, 1.5) | 0.97 | 1037 | 1.01 (0.66, 1.54) | 0.97 | 442 | 1.09 (0.64, 1.86) | 0.767 | 7605 | 0.97 (0.63, 1.49) | 0.91 |
| 622.1 | Polyp of corpus uteri | 7801 | 10445586 | 3298 | 0.99 (0.64, 1.52) | 0.952 | 894 | 1 (0.94, 1.07) | 0.97 | 379 | 1.07 (0.56, 2.04) | 0.845 | 6557 | 0.97 (0.64, 1.48) | 0.91 |
| 622.2 | Mucous polyp of cervix | 1401 | 10498802 | 651 | **1.22 (1.07, 1.39)** | **0.002** | 162 | 1.11 (0.7, 1.75) | 0.672 | 71 | 1.24 (0.79, 1.96) | 0.361 | 1179 | 0.97 (0.49, 1.93) | 0.938 |
| 623 | Hypertrophy of female genital organs | 953 | 10501172 | 406 | 1.04 (0.51, 2.12) | 0.93 | 118 | 1.06 (0.41, 2.79) | 0.909 | 48 | 1.12 (0.28, 4.54) | 0.883 | 793 | 0.89 (0.56, 1.41) | 0.632 |
| 624 | Symptoms involving female genital tract | 5565 | 10464757 | 2370 | 1 (0.93, 1.07) | 0.97 | 666 | 1.07 (0.84, 1.36) | 0.588 | 242 | 0.97 (0.42, 2.22) | 0.949 | 4696 | 1.01 (0.71, 1.42) | 0.97 |
| 624.1 | Dystrophy of female genital tract | 109 | 10510039 | 48 | 1.21 (0.27, 5.52) | 0.816 | 17 | 1.66 (0.54, 5.16) | 0.386 | 6 | 1.46 (0.07, 31.53) | 0.82 | 91 | 0.95 (0.08, 11.23) | 0.97 |
| 624.9 | stress incontinence, female | 5355 | 10467262 | 2276 | 0.99 (0.77, 1.29) | 0.97 | 642 | 1.07 (0.86, 1.33) | 0.542 | 232 | 0.96 (0.49, 1.92) | 0.925 | 4518 | 1.01 (0.75, 1.36) | 0.97 |
| 625 | Pain and other symptoms associated with female genital organs | 4332 | 10454892 | 1854 | 1.03 (0.76, 1.39) | 0.864 | 498 | 0.95 (0.64, 1.4) | 0.808 | 222 | 1.11 (0.75, 1.65) | 0.599 | 3672 | 0.99 (0.6, 1.64) | 0.97 |
| 625.1 | Dyspareunia | 2865 | 10465249 | 1201 | 0.99 (0.57, 1.72) | 0.97 | 336 | 0.97 (0.52, 1.82) | 0.935 | 146 | 1.1 (0.62, 1.93) | 0.758 | 2420 | 0.98 (0.57, 1.67) | 0.935 |
| 626 | Disorders of menstruation and other abnormal bleeding from female genital tract | 46884 | 9761252 | 19729 | 0.98 (0.91, 1.06) | 0.66 | 5539 | 0.97 (0.88, 1.08) | 0.629 | 2116 | 0.96 (0.81, 1.15) | 0.706 | 39647 | 0.98 (0.88, 1.1) | 0.771 |
| 626.1 | Irregular menstrual cycle/bleeding | 2558 | 10471597 | 1070 | 0.94 (0.7, 1.27) | 0.718 | 285 | 0.9 (0.6, 1.36) | 0.637 | 107 | 0.87 (0.44, 1.74) | 0.715 | 2160 | 0.99 (0.52, 1.88) | 0.97 |
| 626.11 | Absent or infrequent menstruation | 1568 | 10484323 | 633 | 0.92 (0.68, 1.25) | 0.624 | 195 | 0.97 (0.32, 2.96) | 0.965 | 72 | 0.94 (0.25, 3.63) | 0.939 | 1331 | 0.99 (0.72, 1.37) | 0.97 |
| 626.12 | Excessive or frequent menstruation | 10504 | 10375823 | 4270 | **0.91 (0.85, 0.97)** | **0.003** | 1187 | 0.91 (0.79, 1.06) | 0.221 | 490 | 0.96 (0.55, 1.67) | 0.9 | 8850 | 0.97 (0.75, 1.25) | 0.823 |
| 626.13 | Irregular menstrual cycle | 3044 | 10447192 | 1268 | 0.95 (0.73, 1.24) | 0.732 | 349 | 0.97 (0.48, 1.94) | 0.934 | 140 | 0.98 (0.46, 2.11) | 0.97 | 2562 | 0.98 (0.56, 1.7) | 0.935 |
| 626.14 | Irregular menstrual bleeding | 17006 | 10144183 | 7237 | 0.98 (0.83, 1.16) | 0.85 | 1929 | 0.98 (0.72, 1.34) | 0.924 | 745 | 0.96 (0.68, 1.35) | 0.814 | 14291 | 0.97 (0.82, 1.15) | 0.767 |
| 626.15 | Infertility, female, associated with anovulation | 1056 | 10502929 | 459 | 1.13 (0.66, 1.92) | 0.668 | 141 | 1.12 (0.35, 3.54) | 0.861 | 55 | 1.2 (0.28, 5.17) | 0.818 | 902 | 1.01 (0.63, 1.62) | 0.97 |
| 626.2 | Dysmenorrhea | 3507 | 10465901 | 1504 | 1.02 (0.47, 2.22) | 0.97 | 394 | 0.92 (0.02, 55.27) | 0.97 | 166 | 1.01 (0.57, 1.8) | 0.97 | 2966 | 0.97 (0.28, 3.36) | 0.97 |
| 626.21 | Mittelschmerz | 940 | 10500745 | 400 | 1.03 (0.41, 2.53) | 0.961 | 120 | 1.02 (0.47, 2.19) | 0.97 | 38 | 0.86 (0.22, 3.31) | 0.835 | 807 | 1.06 (0.44, 2.55) | 0.909 |
| 626.8 | Infertility, female | 16184 | 10334123 | 6827 | 1.02 (0.33, 3.15) | 0.97 | 2060 | 1.03 (0.28, 3.72) | 0.97 | 740 | 1 (0.8, 1.23) | 0.97 | 13721 | 0.97 (0.24, 3.87) | 0.97 |
| 627 | Menopausal and postmenopausal disorders | 13132 | 10297965 | 5651 | 0.97 (0.87, 1.08) | 0.592 | 1383 | 0.93 (0.82, 1.06) | 0.294 | 532 | 0.88 (0.75, 1.03) | 0.116 | 11006 | 0.98 (0.78, 1.22) | 0.849 |
| 627.1 | Postmenopausal bleeding | 6341 | 10442513 | 2736 | 0.98 (0.37, 2.57) | 0.97 | 682 | 0.98 (0.34, 2.85) | 0.97 | 259 | 0.9 (0.03, 29.63) | 0.958 | 5312 | 0.98 (0.41, 2.36) | 0.97 |
| 627.2 | Symptomatic menopause | 276 | 10508683 | 115 | 0.92 (0.21, 4.09) | 0.923 | 32 | 0.98 (0.37, 2.59) | 0.97 | 9 | 0.68 (0.04, 11.97) | 0.806 | 237 | 1.13 (0.19, 6.92) | 0.901 |
| 627.3 | Postmenopausal atrophic vaginitis | 530 | 10507027 | 227 | 0.96 (0.3, 3.09) | 0.956 | 62 | 1.05 (0.18, 6.3) | 0.958 | 16 | 0.67 (0.19, 2.29) | 0.528 | 443 | 0.97 (0.23, 4.12) | 0.97 |
| 627.4 | Premenopausal menorrhagia | 6244 | 10369603 | 2692 | 0.96 (0.77, 1.21) | 0.767 | 633 | 0.89 (0.72, 1.09) | 0.267 | 252 | 0.87 (0.59, 1.28) | 0.487 | 5230 | 0.97 (0.65, 1.45) | 0.898 |
| 627.5 | Premature menopause and other ovarian failure | 184 | 10509968 | 71 | 0.89 (0.28, 2.89) | 0.861 | 24 | 1 (0.96, 1.04) | 0.97 | 11 | 1.24 (0.12, 12.49) | 0.868 | 165 | 1.52 (0.7, 3.3) | 0.297 |
| 628 | Ovarian cyst | 12515 | 10360641 | 5357 | 1 (0.81, 1.24) | 0.97 | 1427 | 0.95 (0.8, 1.12) | 0.52 | 546 | 0.94 (0.69, 1.28) | 0.7 | 10581 | 0.99 (0.73, 1.34) | 0.934 |
| **Pregnancy Complications** | | | | | | | | | | | | | | | |
| 634 | Miscarriage; stillbirth | 90431 | 9549371 | 37518 | 0.99 (0.72, 1.36) | 0.969 | 11562 | 1 (0.89, 1.12) | 0.97 | 4287 | 1 (0.81, 1.23) | 0.97 | 77079 | 1 (0.97, 1.03) | 0.97 |
| 634.1 | Missed abortion/Hydatidiform mole | 15015 | 10370424 | 6351 | 1.02 (0.94, 1.11) | 0.62 | 1855 | 0.98 (0.82, 1.17) | 0.827 | 697 | 1 (0.87, 1.14) | 0.97 | 12750 | 0.98 (0.86, 1.12) | 0.767 |
| 634.3 | Ectopic pregnancy | 5034 | 10426967 | 2079 | 1 (0.87, 1.16) | 0.97 | 673 | **1.12 (1.02, 1.22)** | **0.015** | 260 | 1.13 (0.96, 1.33) | 0.136 | 4269 | 0.99 (0.7, 1.39) | 0.949 |
| 635 | Hemorrhage during pregnancy; childbirth and postpartum | 93100 | 10031154 | 38454 | 0.99 (0.63, 1.55) | 0.97 | 12165 | 1.01 (0.74, 1.36) | 0.97 | 4395 | 0.99 (0.59, 1.66) | 0.97 | 79377 | 0.99 (0.58, 1.68) | 0.97 |
| 635.2 | Antepartum hemorrhage, abruptio placentae, and placenta previa | 7411 | 10412135 | 3215 | 1.07 (0.84, 1.35) | 0.607 | 901 | 1 (0.91, 1.1) | 0.97 | 341 | 1.01 (0.61, 1.67) | 0.97 | 6264 | 0.96 (0.56, 1.63) | 0.883 |
| 635.3 | Placenta previa and abruptio placenta | 89824 | 10113527 | 37017 | 0.99 (0.73, 1.33) | 0.935 | 11810 | 1.01 (0.65, 1.58) | 0.97 | 4238 | 0.98 (0.44, 2.22) | 0.97 | 76611 | 0.99 (0.59, 1.66) | 0.97 |
| 636 | Early or threatened labor; hemorrhage in early pregnancy | 24129 | 10239300 | 10011 | 1.01 (0.68, 1.49) | 0.97 | 3164 | 1.05 (0.46, 2.41) | 0.909 | 1213 | 1.09 (0.33, 3.63) | 0.901 | 20536 | 0.99 (0.74, 1.34) | 0.97 |
| 636.3 | Hemorrhage in early pregnancy | 18844 | 10282572 | 7760 | 0.99 (0.75, 1.32) | 0.97 | 2487 | 1.06 (0.57, 1.97) | 0.863 | 948 | 1.08 (0.4, 2.9) | 0.883 | 15992 | 0.98 (0.43, 2.21) | 0.958 |
| 636.8 | Cervical incompetence | 843 | 10500130 | 355 | 1.05 (0.74, 1.49) | 0.788 | 109 | 1.09 (0.71, 1.68) | 0.71 | 49 | 1.3 (0.96, 1.77) | 0.09 | 717 | 1 (0.92, 1.09) | 0.97 |
| 637 | Short gestation; low birth weight; and fetal growth retardation | 4515 | 482914* | 1889 | 1.02 (0.36, 2.89)** | 0.97 | 612 | 1.04 (0.15, 7.39)** | 0.97 | 210 | 0.99 (0.54, 1.82)** | 0.97 | 3843 | 0.99 (0.71, 1.4)** | 0.97 |
| 638 | Other high-risk pregnancy | 50724 | 10230471 | 21057 | 1.01 (0.59, 1.73) | 0.97 | 6721 | 1.04 (0.16, 6.74) | 0.97 | 2395 | 1.02 (0.36, 2.91) | 0.97 | 43273 | 1 (0.91, 1.1) | 0.97 |
| 639 | Complications following abortion or ectopic and molar pregnancies | 4217 | 10460678 | 1745 | 0.95 (0.82, 1.12) | 0.566 | 493 | 0.89 (0.76, 1.04) | 0.132 | 189 | 0.91 (0.61, 1.36) | 0.664 | 3580 | 0.98 (0.65, 1.48) | 0.927 |
| 642 | Hypertension complicating pregnancy, childbirth, and the puerperium | 8724 | 10400534 | 3714 | 1.03 (0.82, 1.3) | 0.817 | 1066 | 0.98 (0.55, 1.77) | 0.963 | 404 | 1 (0.91, 1.09) | 0.97 | 7410 | 0.99 (0.58, 1.68) | 0.965 |
| 642.1 | Preeclampsia and eclampsia | 7923 | 10406113 | 3363 | 1.02 (0.68, 1.55) | 0.917 | 975 | 0.99 (0.7, 1.4) | 0.97 | 366 | 0.99 (0.73, 1.35) | 0.97 | 6733 | 0.99 (0.67, 1.48) | 0.97 |
| 643 | Excessive vomiting in pregnancy | 4314 | 10470323 | 1696 | 0.95 (0.83, 1.07) | 0.391 | 633 | **1.15 (1.03, 1.28)** | **0.011** | 227 | 1.11 (0.84, 1.48) | 0.474 | 3678 | 1 (0.89, 1.11) | 0.97 |
| 643.1 | Hyperemesis gravidarum | 3558 | 10488446 | 1384 | 0.93 (0.82, 1.06) | 0.267 | 524 | 1.12 (0.96, 1.3) | 0.138 | 187 | 1.1 (0.73, 1.65) | 0.668 | 3057 | 1.05 (0.77, 1.43) | 0.791 |
| 644 | Anemia during pregnancy | 8583 | 10367686 | 3638 | 1.03 (0.94, 1.12) | 0.527 | 1071 | 1.04 (0.89, 1.2) | 0.644 | 418 | 1.06 (0.87, 1.29) | 0.555 | 7312 | 1.02 (0.82, 1.26) | 0.885 |
| 645 | Late pregnancy and failed induction | 2623 | 10450398 | 1082 | 0.94 (0.82, 1.09) | 0.447 | 310 | 0.97 (0.58, 1.63) | 0.928 | 120 | 0.96 (0.49, 1.87) | 0.904 | 2225 | 1.01 (0.76, 1.32) | 0.97 |
| 646 | Other complications of pregnancy NEC | 27415 | 10268306 | 11467 | 1.01 (0.69, 1.47) | 0.97 | 3473 | 0.98 (0.57, 1.69) | 0.959 | 1273 | 0.98 (0.44, 2.19) | 0.97 | 23445 | 1.02 (0.71, 1.47) | 0.906 |
| 647 | Infectious and parasitic complications affecting pregnancy | 16102 | 10395027 | 6720 | 1.01 (0.58, 1.75) | 0.97 | 2100 | 1.02 (0.46, 2.27) | 0.97 | 751 | 0.99 (0.59, 1.65) | 0.97 | 13791 | 1.03 (0.23, 4.55) | 0.97 |
| 647.1 | Infections of genitourinary tract during pregnancy | 9991 | 10430617 | 4193 | 1.02 (0.37, 2.8) | 0.97 | 1304 | 1.03 (0.31, 3.38) | 0.97 | 460 | 0.98 (0.42, 2.29) | 0.97 | 8576 | 1.05 (0.11, 10.13) | 0.97 |
| 647.3 | Major puerperal infection | 274 | 10504689 | 119 | 1.12 (0.68, 1.84) | 0.671 | 42 | **1.44 (1.04, 1.99)** | **0.028** | 10 | 0.87 (0.12, 6.43) | 0.898 | 219 | **0.72 (0.56, 0.93)** | **0.01** |
| 649 | Other conditions or status of the mother complicating pregnancy, childbirth, or the puerperium | 22751 | 10405488 | 9248 | 0.99 (0.5, 1.95) | 0.97 | 3149 | 1.07 (0.4, 2.9) | 0.897 | 1130 | 1.06 (0.15, 7.51) | 0.96 | 19635 | 1.09 (0.48, 2.46) | 0.848 |
| 649.1 | Diabetes or abnormal glucose tolerance complicating pregnancy | 5053 | 10480682 | 2001 | 0.98 (0.48, 2.03) | 0.966 | 773 | 1.22 (0.98, 1.52) | 0.073 | 275 | 1.19 (0.7, 2.04) | 0.527 | 4446 | **1.26 (1.03, 1.55)** | **0.026** |
| 650 | Normal delivery | 91121 | 9417994 | 38038 | 1 (1, 1) | 0.97 | 11420 | 0.99 (0.9, 1.08) | 0.771 | 4301 | 1.01 (0.82, 1.25) | 0.935 | 77386 | 0.98 (0.93, 1.03) | 0.465 |
| 651 | Multiple gestation | 5389 | 10449676 | 2328 | 1.04 (0.92, 1.17) | 0.566 | 624 | 0.91 (0.81, 1.03) | 0.134 | 229 | 0.91 (0.7, 1.17) | 0.474 | 4577 | 0.98 (0.73, 1.32) | 0.914 |
| 652 | Malposition and malpresentation of fetus or obstruction | 23509 | 10218315 | 9851 | 0.99 (0.74, 1.34) | 0.97 | 2878 | 0.96 (0.19, 4.91) | 0.968 | 1061 | 0.95 (0.1, 8.91) | 0.97 | 20046 | 1.01 (0.55, 1.88) | 0.97 |
| 653 | Problems associated with amniotic cavity and membranes | 9932 | 10363382 | 4129 | 1.01 (0.64, 1.58) | 0.97 | 1304 | 1.08 (0.8, 1.48) | 0.624 | 501 | 1.1 (0.65, 1.86) | 0.733 | 8419 | 0.98 (0.53, 1.8) | 0.952 |
| 654 | Other and unspecified complications of birth; puerperium affecting management of mother | 8454 | 10450698 | 3442 | 0.96 (0.27, 3.43) | 0.958 | 1126 | 1.03 (0.29, 3.58) | 0.97 | 372 | 0.92 (0.06, 14.34) | 0.959 | 7073 | 0.88 (0.37, 2.1) | 0.791 |
| 654.1 | Abnormality of organs and soft tissues of pelvis complicating pregnancy, childbirth, or the puerperium | 5842 | 10479533 | 2339 | **0.94 (0.9, 0.99)** | **0.029** | 808 | 1.05 (0.94, 1.17) | 0.364 | 255 | 0.91 (0.78, 1.07) | 0.254 | 5036 | **1.08 (1.01, 1.15)** | **0.029** |
| 654.2 | Rhesus isoimmunization in pregnancy | 808 | 10503190 | 361 | 1.06 (0.72, 1.57) | 0.784 | 82 | 0.82 (0.59, 1.14) | 0.24 | 31 | 0.82 (0.39, 1.75) | 0.629 | 485 | **0.26 (0.24, 0.29)** | **<0.001** |
| 655 | Known or suspected fetal abnormality affecting management of mother | 47459 | 10162124 | 19687 | 0.99 (0.72, 1.37) | 0.97 | 6082 | 0.99 (0.49, 1.97) | 0.97 | 2196 | 0.97 (0.23, 4.01) | 0.97 | 40442 | 0.99 (0.74, 1.34) | 0.97 |
| 656 | Other perinatal conditions of fetus or newborn | 12313 | 482914* | 5355 | 1.09 (0.99, 1.21)** | 0.079 | 1588 | 1.04 (0.67, 1.6)** | 0.883 | 579 | 1.03 (0.42, 2.54)** | 0.956 | 10510 | 1.01 (0.69, 1.48)** | 0.97 |
| 656.1 | Isoimmunization of fetus or newborn | 508 | 482914* | 330 | **4.49 (1.96, 10.24)**** | **<0.001** | 96 | **4.2 (1.38, 12.73)**** | **0.011** | 10 | 1.18 (0, 2997.98)** | 0.97 | 436 | 1.03 (0.21, 5.13)** | 0.97 |
| 656.2 | Respiratory conditions of fetus and newborn | 3238 | 482914* | 1421 | 1.08 (0.03, 45.76)** | 0.97 | 378 | 0.91 (0.01, 89.88)** | 0.97 | 156 | 1.03 (0.29, 3.6)** | 0.97 | 2761 | 1 (0.89, 1.12)** | 0.97 |
| 656.22 | Interstitial emphysema and related conditions of newborn | 487 | 482914* | 208 | 1.04 (0.19, 5.52)** | 0.97 | 60 | 0.95 (0.09, 10.29)** | 0.97 | 23 | 0.99 (0.74, 1.33)** | 0.97 | 405 | 0.85 (0, 1942.66)** | 0.97 |
| 656.26 | Transitory tachypnea or apnea of newborn | 921 | 482914* | 406 | 1.16 (0, 1354.06)** | 0.97 | 124 | 1.11 (0.01, 182.43)** | 0.97 | 51 | 1.27 (0, 91479.19)** | 0.97 | 805 | 1.2 (0, 6538.1)** | 0.97 |
| 656.3 | Endocrine and metabolic disturbances of fetus and newborn | 2561 | 482914* | 1040 | 0.99 (0.49, 1.98)** | 0.97 | 344 | 1.02 (0.37, 2.83)** | 0.968 | 145 | 1.19 (0.73, 1.95)** | 0.5 | 2191 | 1.03 (0.44, 2.4)** | 0.958 |
| 656.4 | Hemorrhage of fetus or newborn | 438 | 482914* | 197 | 1.12 (0.5, 2.52)** | 0.79 | 50 | 0.9 (0.17, 4.65)** | 0.906 | 20 | 0.99 (0.52, 1.88)** | 0.97 | 382 | 1.18 (0.42, 3.36)** | 0.767 |
| 656.5 | Hematological disorders of newborn | 535 | 482914* | 212 | 0.98 (0.3, 3.2)** | 0.97 | 78 | 1.11 (0.37, 3.32)** | 0.861 | 32 | 1.27 (0.45, 3.61)** | 0.67 | 462 | 1.09 (0.36, 3.32)** | 0.883 |
| 656.6 | Perinatal disorders of digestive system | 102 | 482914* | 38 | 0.8 (0.08, 7.64)** | 0.859 | 9 | 0.61 (0.04, 8.8)** | 0.73 | 9 | 1.67 (0.13, 22.21)** | 0.71 | 86 | 0.94 (0.04, 20.6)** | 0.97 |
| 656.7 | Conditions involving the integument and temperature regulation of fetus and newborn | 416 | 482914* | 171 | 0.98 (0.4, 2.41)** | 0.97 | 60 | 1.18 (0.27, 5.09)** | 0.84 | 18 | 0.93 (0.03, 24.93)** | 0.97 | 343 | 0.82 (0.33, 2.05)** | 0.683 |
| 656.8 | Perinatal jaundice | 3142 | 482914* | 1364 | 1.11 (0.01, 173.02)** | 0.97 | 434 | 1.12 (0.01, 200.13)** | 0.97 | 149 | 1.06 (0.08, 14.73)** | 0.97 | 2669 | 0.98 (0.33, 2.91)** | 0.97 |
| 657 | Infections specific to the perinatal period | 2003 | 482914* | 838 | 1.01 (0.72, 1.41)** | 0.97 | 256 | 0.96 (0.17, 5.54)** | 0.97 | 97 | 1.01 (0.61, 1.67)** | 0.97 | 1700 | 0.97 (0.27, 3.52)** | 0.97 |
| 658 | Maternal complication of pregnancy affecting fetus or newborn | 3297 | 291649* | 1411 | 1.04 (0.17, 6.33)** | 0.97 | 376 | 0.88 (0, 341.07)** | 0.97 | 160 | 1.02 (0.47, 2.21)** | 0.97 | 2761 | 0.9 (0.01, 116.98)** | 0.97 |
| 661 | Fetal distress and abnormal forces of labor | 70331 | 291649* | 29427 | 1 (0.83, 1.21)** | 0.97 | 8773 | 0.99 (0.54, 1.81)** | 0.97 | 3337 | 1.01 (0.68, 1.5)** | 0.97 | 59792 | 0.99 (0.51, 1.91)** | 0.97 |
| 663 | Umbilical cord complications during labor and delivery | 1271 | 10476448 | 544 | 1.02 (0.61, 1.72) | 0.946 | 146 | 1.01 (0.51, 2.01) | 0.97 | 65 | 1.13 (0.64, 1.99) | 0.682 | 1051 | 0.87 (0.73, 1.03) | 0.106 |
| 665 | Obstetrical/birth trauma | 70741 | 291649* | 29517 | 1 (0.9, 1.12)** | 0.97 | 8862 | 0.96 (0.14, 6.43)** | 0.97 | 3313 | 0.98 (0.47, 2.07)** | 0.97 | 60017 | 0.96 (0.14, 6.8)** | 0.97 |
| 668 | Complications of the administration of anesthetic or other sedation in labor and delivery | 483 | 10508786 | 195 | 0.96 (0.39, 2.39) | 0.935 | 68 | 1.07 (0.31, 3.65) | 0.92 | 20 | 0.86 (0.14, 5.4) | 0.885 | 400 | 0.83 (0.52, 1.32) | 0.435 |
| 669 | Complications of labor and delivery NEC | 67921 | 9847638 | 28255 | 1 (0.95, 1.05) | 0.97 | 8677 | 1 (0.97, 1.03) | 0.97 | 3180 | 0.99 (0.74, 1.33) | 0.97 | 57824 | 0.99 (0.72, 1.38) | 0.97 |
| 671 | Venous/cerebrovascular complications embolism in pregnancy and the puerperium | 3082 | 10489609 | 1335 | 1.08 (0.75, 1.56) | 0.691 | 386 | 1.01 (0.59, 1.75) | 0.97 | 145 | 1.04 (0.16, 6.74) | 0.97 | 2605 | 0.94 (0.47, 1.92) | 0.883 |
| 674 | Other complications of the puerperium NEC | 4303 | 10473463 | 1707 | 0.89 (0.79, 1.01) | 0.062 | 526 | 0.9 (0.69, 1.17) | 0.432 | 193 | 0.89 (0.52, 1.55) | 0.7 | 3643 | 0.96 (0.62, 1.49) | 0.861 |
| 676 | Other disorders of the breast associated with childbirth and disorders of lactation | 3845 | 10487510 | 1594 | 1.01 (0.58, 1.78) | 0.97 | 514 | 1.05 (0.09, 12.11) | 0.97 | 191 | 1.06 (0.06, 18.35) | 0.97 | 3303 | 1.05 (0.1, 11.49) | 0.97 |
| **Dermatologic** | | | | | | | | | | | | | | | |
| 681 | Superficial cellulitis and abscess | 10075 | 17491042 | 4318 | 1 (0.9, 1.1) | 0.97 | 1178 | 1 (0.87, 1.14) | 0.97 | 452 | 0.98 (0.43, 2.24) | 0.97 | 8494 | 0.98 (0.53, 1.8) | 0.949 |
| 681.1 | Cellulitis and abscess of fingers/toes | 5878 | 17540091 | 2560 | 1.03 (0.46, 2.3) | 0.949 | 692 | 1.02 (0.48, 2.13) | 0.97 | 252 | 0.95 (0.11, 8.46) | 0.968 | 4953 | 0.97 (0.3, 3.2) | 0.968 |
| 681.2 | Cellulitis and abscess of face/neck | 1601 | 17615614 | 649 | 0.9 (0.71, 1.13) | 0.364 | 186 | 0.94 (0.42, 2.11) | 0.898 | 77 | 1.01 (0.79, 1.28) | 0.97 | 1359 | 1.02 (0.39, 2.65) | 0.965 |
| 681.3 | Cellulitis and abscess of arm/hand | 1287 | 17614763 | 545 | 0.96 (0.55, 1.69) | 0.898 | 148 | 0.97 (0.31, 3.01) | 0.959 | 55 | 0.92 (0.24, 3.54) | 0.912 | 1088 | 1 (0.95, 1.05) | 0.97 |
| 681.5 | Cellulitis and abscess of leg, except foot | 1288 | 17614749 | 545 | 0.96 (0.54, 1.69) | 0.894 | 148 | 0.97 (0.29, 3.16) | 0.958 | 55 | 0.92 (0.24, 3.46) | 0.909 | 1088 | 1 (0.83, 1.2) | 0.97 |
| 681.6 | Cellulitis and abscess of foot, toe | 1288 | 17614749 | 545 | 0.96 (0.54, 1.69) | 0.894 | 148 | 0.97 (0.29, 3.16) | 0.958 | 55 | 0.92 (0.24, 3.46) | 0.909 | 1088 | 1 (0.83, 1.2) | 0.97 |
| 681.7 | Cellulitis and abscess of trunk | 158 | 17630518 | 70 | 1.19 (0.33, 4.28) | 0.805 | 23 | 1.46 (0.45, 4.74) | 0.543 | 9 | 1.45 (0.14, 14.96) | 0.767 | 125 | 0.69 (0.32, 1.51) | 0.364 |
| 686 | Other local infections of skin and subcutaneous tissue | 37933 | 17229740 | 16182 | 1 (0.99, 1.01) | 0.97 | 4559 | 1.02 (0.9, 1.15) | 0.751 | 1725 | 1 (0.82, 1.21) | 0.97 | 32052 | 0.99 (0.86, 1.13) | 0.859 |
| 686.1 | Carbuncle and furuncle | 19885 | 17413050 | 8458 | 1 (0.81, 1.23) | 0.97 | 2411 | 1.04 (0.91, 1.19) | 0.6 | 908 | 1.01 (0.79, 1.28) | 0.97 | 16786 | 0.98 (0.81, 1.19) | 0.885 |
| 686.2 | Impetigo | 1464 | 17614718 | 619 | 0.98 (0.46, 2.08) | 0.959 | 175 | 0.94 (0.4, 2.22) | 0.898 | 59 | 0.84 (0.35, 2.01) | 0.706 | 1238 | 0.97 (0.41, 2.27) | 0.942 |
| 686.3 | Pilonidal cyst | 4850 | 17557709 | 2053 | 1.02 (0.68, 1.52) | 0.932 | 624 | 1.09 (0.82, 1.44) | 0.578 | 241 | 1.09 (0.65, 1.83) | 0.746 | 4078 | 0.94 (0.73, 1.21) | 0.633 |
| 686.4 | Pyogenic granuloma | 408 | 17626938 | 186 | 1.15 (0.52, 2.56) | 0.742 | 51 | 1.14 (0.19, 6.73) | 0.897 | 16 | 0.92 (0.02, 40.78) | 0.97 | 358 | 1.3 (0.52, 3.26) | 0.592 |
| 686.5 | Pyoderma | 198 | 17627595 | 77 | 1 (0.79, 1.25) | 0.97 | 32 | 1.51 (0.56, 4.04) | 0.423 | 15 | 1.81 (0.56, 5.9) | 0.327 | 164 | 0.87 (0.14, 5.49) | 0.892 |
| 687 | Symptoms affecting skin | 6418 | 17587705 | 2812 | 1.04 (0.91, 1.19) | 0.576 | 756 | 1.02 (0.7, 1.49) | 0.91 | 262 | 0.91 (0.66, 1.25) | 0.576 | 5413 | 0.98 (0.7, 1.38) | 0.924 |
| 687.1 | Rash and other nonspecific skin eruption | 841 | 17624846 | 361 | 0.95 (0.49, 1.85) | 0.883 | 88 | 0.87 (0.43, 1.75) | 0.706 | 29 | 0.73 (0.3, 1.76) | 0.487 | 702 | 0.93 (0.4, 2.2) | 0.883 |
| 687.2 | Localized superficial swelling, mass, or lump | 612 | 17627775 | 275 | 1.17 (0.73, 1.86) | 0.531 | 82 | 1.29 (0.72, 2.33) | 0.398 | 30 | 1.21 (0.23, 6.29) | 0.835 | 513 | 0.95 (0.24, 3.84) | 0.949 |
| 687.4 | Disturbance of skin sensation | 1887 | 17620750 | 837 | 1.07 (0.69, 1.65) | 0.771 | 221 | 1.01 (0.73, 1.39) | 0.97 | 77 | 0.91 (0.21, 3.87) | 0.909 | 1611 | 1.05 (0.48, 2.31) | 0.906 |
| 689 | Disorder of skin and subcutaneous tissue NOS | 1544 | 17619340 | 686 | 1.08 (0.6, 1.92) | 0.817 | 181 | 1.05 (0.27, 4.13) | 0.947 | 68 | 1.01 (0.68, 1.5) | 0.97 | 1271 | 0.85 (0.56, 1.3) | 0.468 |
| 690 | Erythematosquamous dermatosis | 1586 | 17615064 | 663 | 0.97 (0.54, 1.74) | 0.923 | 198 | 1.07 (0.48, 2.35) | 0.881 | 73 | 1.01 (0.65, 1.57) | 0.97 | 1350 | 1.05 (0.52, 2.09) | 0.909 |
| 690.1 | Seborrheic dermatitis | 1406 | 17616850 | 583 | 0.97 (0.51, 1.82) | 0.923 | 181 | 1.1 (0.62, 1.97) | 0.748 | 67 | 1.05 (0.2, 5.6) | 0.959 | 1199 | 1.06 (0.51, 2.19) | 0.89 |
| 691 | Congenital anomalies of skin | 1033 | 482914 | 455 | 1.09 (0.64, 1.86) | 0.773 | 126 | 1.08 (0.35, 3.32) | 0.897 | 49 | 1.09 (0.17, 6.94) | 0.935 | 893 | 1.16 (0.65, 2.05) | 0.628 |
| 691.1 | Ichthyosis congenita | 128 | 482914 | 56 | 1.03 (0.22, 4.77) | 0.97 | 13 | 0.86 (0.03, 27.25) | 0.938 | 7 | 1.2 (0.01, 181) | 0.949 | 116 | 1.76 (0.42, 7.31) | 0.445 |
| 691.3 | Congenital pigmentary anomalies of skin | 120 | 482914 | 54 | 1.11 (0.12, 10.35) | 0.935 | 15 | 1.1 (0.02, 74.58) | 0.966 | 4 | 0.76 (0, 261.66) | 0.933 | 103 | 1.1 (0.03, 35.63) | 0.962 |
| 694 | Dyschromia and Vitiligo | 2104 | 17615133 | 881 | 0.96 (0.6, 1.53) | 0.882 | 246 | 0.98 (0.45, 2.17) | 0.97 | 99 | 1.01 (0.51, 2.02) | 0.97 | 1776 | 0.99 (0.54, 1.8) | 0.97 |
| 694.2 | Other dyschromia | 1281 | 17620451 | 536 | 0.97 (0.48, 1.98) | 0.938 | 153 | 0.99 (0.59, 1.65) | 0.97 | 59 | 0.99 (0.53, 1.85) | 0.97 | 1094 | 1.06 (0.48, 2.34) | 0.902 |
| 694.3 | Vascular disorders of skin | 833 | 17626395 | 348 | 0.95 (0.51, 1.76) | 0.885 | 97 | 1.01 (0.7, 1.45) | 0.97 | 40 | 1.05 (0.13, 8.39) | 0.967 | 689 | 0.89 (0.57, 1.38) | 0.605 |
| 695 | Erythematous conditions | 7080 | 17558088 | 3010 | 0.99 (0.77, 1.28) | 0.97 | 836 | 1.04 (0.62, 1.74) | 0.897 | 343 | 1.08 (0.6, 1.96) | 0.813 | 5956 | 0.98 (0.6, 1.59) | 0.935 |
| 695.1 | Toxic erythema | 692 | 17619581 | 287 | 0.89 (0.5, 1.6) | 0.714 | 75 | 0.84 (0.33, 2.11) | 0.716 | 21 | 0.6 (0.2, 1.84) | 0.383 | 579 | 0.93 (0.3, 2.85) | 0.901 |
| 695.2 | Bullous dermatoses | 130 | 17630919 | 56 | 0.99 (0.55, 1.78) | 0.97 | 15 | 0.99 (0.61, 1.61) | 0.97 | 5 | 0.84 (0.01, 127.49) | 0.95 | 109 | 0.96 (0.13, 6.87) | 0.97 |
| 695.21 | Dermatitis herpetiformis | 156 | 17629338 | 70 | 1.05 (0.13, 8.69) | 0.97 | 19 | 1.06 (0.08, 14.47) | 0.97 | 3 | 0.42 (0, 156.58) | 0.788 | 129 | 0.88 (0.03, 30.26) | 0.948 |
| 695.22 | Pemphigus and pemphigoid | 443 | 17628832 | 187 | 0.92 (0.27, 3.2) | 0.909 | 46 | 0.91 (0.1, 8.1) | 0.935 | 19 | 0.93 (0.02, 35.79) | 0.97 | 384 | 1.24 (0.42, 3.68) | 0.714 |
| 695.3 | Rosacea | 1581 | 17619010 | 677 | 1 (0.82, 1.22) | 0.97 | 174 | 0.95 (0.28, 3.23) | 0.935 | 79 | 1.1 (0.24, 4.93) | 0.91 | 1327 | 0.96 (0.37, 2.49) | 0.935 |
| 695.41 | Cutaneous lupus erythematosus | 493 | 17626909 | 184 | 0.83 (0.48, 1.44) | 0.527 | 69 | 1.16 (0.36, 3.71) | 0.818 | 28 | 1.2 (0.17, 8.46) | 0.868 | 424 | 1.13 (0.32, 3.98) | 0.864 |
| 695.42 | Systemic lupus erythematosus | 792 | 17622954 | 338 | 1.08 (0.61, 1.91) | 0.805 | 110 | 1.27 (0.86, 1.9) | 0.234 | 42 | 1.25 (0.51, 3.03) | 0.636 | 669 | 0.99 (0.56, 1.75) | 0.97 |
| 695.7 | Prurigo and Lichen | 2218 | 17610409 | 949 | 1.01 (0.7, 1.45) | 0.97 | 264 | 1.08 (0.22, 5.44) | 0.929 | 109 | 1.12 (0.1, 12.03) | 0.932 | 1859 | 0.97 (0.21, 4.47) | 0.97 |
| 695.8 | Other specified erythematous conditions | 622 | 17626339 | 276 | 1.19 (0.77, 1.83) | 0.434 | 87 | 1.42 (0.93, 2.18) | 0.108 | 38 | 1.57 (0.82, 2.98) | 0.171 | 518 | 0.92 (0.32, 2.62) | 0.887 |
| 695.9 | Unspecified erythematous condition | 449 | 17625611 | 192 | 0.96 (0.22, 4.21) | 0.959 | 46 | 0.86 (0.16, 4.5) | 0.864 | 20 | 0.95 (0.07, 13.01) | 0.97 | 381 | 1.03 (0.22, 4.89) | 0.97 |
| 696 | Psoriasis and related disorders | 5686 | 17574071 | 2513 | 1.06 (0.92, 1.22) | 0.441 | 646 | 1.02 (0.59, 1.79) | 0.944 | 266 | 1.07 (0.62, 1.84) | 0.818 | 4813 | 1.02 (0.63, 1.64) | 0.949 |
| 696.2 | Parapsoriasis | 143 | 17630154 | 56 | 0.81 (0.35, 1.88) | 0.63 | 16 | 0.89 (0.07, 10.63) | 0.93 | 5 | 0.69 (0.03, 14.29) | 0.823 | 114 | 0.73 (0.33, 1.65) | 0.46 |
| 696.4 | Psoriasis | 2302 | 17608661 | 1053 | 1.13 (0.92, 1.39) | 0.247 | 254 | 1.04 (0.38, 2.85) | 0.949 | 110 | 1.14 (0.48, 2.71) | 0.786 | 1944 | 1.01 (0.71, 1.44) | 0.97 |
| 696.41 | Psoriasis vulgaris | 4380 | 17592978 | 1929 | 1.06 (0.91, 1.24) | 0.487 | 502 | 1.04 (0.64, 1.68) | 0.898 | 213 | 1.12 (0.77, 1.63) | 0.58 | 3690 | 0.99 (0.56, 1.74) | 0.966 |
| 696.42 | Psoriatic arthropathy | 1807 | 17615515 | 796 | 1.05 (0.69, 1.58) | 0.836 | 204 | 1.01 (0.76, 1.32) | 0.97 | 82 | 1.03 (0.28, 3.82) | 0.97 | 1537 | 1.05 (0.57, 1.92) | 0.885 |
| 697 | Sarcoidosis | 1852 | 17609332 | 810 | 1.03 (0.41, 2.62) | 0.954 | 211 | 0.99 (0.78, 1.27) | 0.97 | 82 | 0.99 (0.6, 1.64) | 0.97 | 1546 | 0.93 (0.41, 2.07) | 0.86 |
| 698 | Pruritus and related conditions | 1602 | 17620694 | 670 | 0.99 (0.66, 1.49) | 0.97 | 213 | 1.16 (0.77, 1.76) | 0.487 | 73 | 1.02 (0.42, 2.45) | 0.97 | 1344 | 0.95 (0.47, 1.93) | 0.901 |
| 701 | Other hypertrophic and atrophic conditions of skin | 6705 | 17570198 | 2846 | 0.99 (0.72, 1.38) | 0.97 | 784 | 0.97 (0.67, 1.39) | 0.864 | 308 | 0.99 (0.55, 1.77) | 0.97 | 5721 | 1.04 (0.84, 1.3) | 0.706 |
| 701.2 | Scar conditions and fibrosis of skin | 3627 | 17600757 | 1559 | 1.02 (0.73, 1.43) | 0.901 | 423 | 0.96 (0.58, 1.6) | 0.898 | 169 | 1.01 (0.67, 1.52) | 0.97 | 3098 | 1.04 (0.73, 1.49) | 0.823 |
| 701.3 | Circumscribed scleroderma | 225 | 17629589 | 109 | 1.21 (0.48, 3) | 0.702 | 22 | 0.89 (0.06, 12.9) | 0.935 | 7 | 0.72 (0.01, 35.05) | 0.88 | 186 | 0.87 (0.16, 4.84) | 0.883 |
| 701.4 | Keloid scar | 761 | 17622112 | 288 | 0.81 (0.4, 1.64) | 0.575 | 85 | 0.82 (0.17, 3.88) | 0.817 | 45 | 1.15 (0.07, 18.78) | 0.929 | 660 | 1.16 (0.24, 5.55) | 0.866 |
| 701.5 | Abnormal granulation tissue | 539 | 17627585 | 241 | 1.12 (0.59, 2.15) | 0.736 | 70 | 1.15 (0.37, 3.63) | 0.817 | 22 | 0.95 (0.07, 13.43) | 0.97 | 460 | 1.04 (0.17, 6.25) | 0.966 |
| 702 | Degenerative skin conditions and other dermatoses | 3459 | 17611275 | 1473 | 0.95 (0.75, 1.21) | 0.705 | 369 | 0.94 (0.59, 1.51) | 0.818 | 153 | 0.97 (0.31, 3.07) | 0.962 | 2911 | 1.01 (0.79, 1.29) | 0.97 |
| 702.1 | Actinic keratosis | 3307 | 17612749 | 1415 | 0.96 (0.72, 1.29) | 0.806 | 353 | 0.95 (0.55, 1.63) | 0.864 | 146 | 0.97 (0.27, 3.53) | 0.97 | 2785 | 1.01 (0.6, 1.7) | 0.97 |
| 703 | Diseases of nail, NOS | 2821 | 17599288 | 1230 | 1.03 (0.66, 1.6) | 0.901 | 319 | 0.96 (0.49, 1.89) | 0.91 | 127 | 0.99 (0.54, 1.81) | 0.97 | 2396 | 1.02 (0.45, 2.3) | 0.97 |
| 703.1 | Ingrowing nail | 2257 | 17604104 | 1007 | 1.08 (0.84, 1.38) | 0.575 | 253 | 0.96 (0.44, 2.13) | 0.934 | 98 | 0.97 (0.21, 4.55) | 0.97 | 1914 | 1 (0.81, 1.24) | 0.97 |
| 704 | Diseases of hair and hair follicles | 4102 | 17578353 | 1728 | 1.04 (0.8, 1.36) | 0.791 | 572 | **1.21 (1.06, 1.38)** | **0.006** | 195 | 1.08 (0.55, 2.09) | 0.841 | 3499 | 1.04 (0.66, 1.63) | 0.883 |
| 704.1 | Alopecia | 394 | 17628567 | 174 | 1.16 (0.61, 2.22) | 0.668 | 65 | 1.53 (0.99, 2.37) | 0.054 | 10 | 0.61 (0.11, 3.26) | 0.577 | 328 | 0.89 (0.29, 2.74) | 0.849 |
| 704.11 | Alopecia Areata | 362 | 17628354 | 155 | 1.13 (0.44, 2.86) | 0.814 | 55 | 1.4 (0.72, 2.73) | 0.323 | 19 | 1.26 (0.16, 9.68) | 0.834 | 304 | 0.93 (0.18, 4.97) | 0.942 |
| 704.2 | Hirsutism | 1778 | 17608888 | 717 | 1.04 (0.66, 1.63) | 0.882 | 281 | **1.36 (1.19, 1.57)** | **<0.001** | 104 | **1.34 (1.03, 1.74)** | **0.028** | 1539 | 1.13 (0.87, 1.46) | 0.377 |
| 704.8 | Other specified diseases of hair and hair follicles | 772 | 17625140 | 322 | 0.94 (0.41, 2.17) | 0.9 | 88 | 0.92 (0.26, 3.2) | 0.901 | 35 | 0.95 (0.08, 10.94) | 0.97 | 656 | 1.02 (0.46, 2.24) | 0.97 |
| 705 | Disorders of sweat glands | 3350 | 17591815 | 1447 | 1.04 (0.76, 1.43) | 0.805 | 406 | 1.02 (0.49, 2.1) | 0.97 | 156 | 1.02 (0.32, 3.24) | 0.97 | 2844 | 1 (0.99, 1.01) | 0.97 |
| 705.3 | Hidradenitis | 1339 | 17614210 | 583 | 1.09 (0.82, 1.45) | 0.578 | 168 | 1.11 (0.68, 1.81) | 0.7 | 71 | 1.22 (0.7, 2.11) | 0.487 | 1136 | 1 (0.87, 1.15) | 0.97 |
| 705.8 | Hyperhidrosis | 1816 | 17611875 | 774 | 0.99 (0.71, 1.38) | 0.97 | 210 | 0.92 (0.42, 2) | 0.836 | 76 | 0.88 (0.24, 3.14) | 0.849 | 1542 | 0.99 (0.7, 1.42) | 0.97 |
| 706 | Diseases of sebaceous glands | 3836 | 17586941 | 1669 | 1.05 (0.83, 1.32) | 0.695 | 451 | 1.02 (0.49, 2.14) | 0.955 | 192 | 1.13 (0.72, 1.77) | 0.62 | 3251 | 1.01 (0.74, 1.36) | 0.97 |
| 706.1 | Acne | 1224 | 17619319 | 514 | 1.03 (0.54, 1.97) | 0.935 | 155 | 1.04 (0.34, 3.13) | 0.953 | 70 | 1.25 (0.71, 2.2) | 0.455 | 1033 | 0.94 (0.47, 1.88) | 0.874 |
| 706.2 | Sebaceous cyst | 2476 | 17599937 | 1092 | 1.05 (0.77, 1.44) | 0.762 | 277 | 1 (0.86, 1.16) | 0.97 | 118 | 1.08 (0.41, 2.84) | 0.883 | 2104 | 1.04 (0.59, 1.84) | 0.898 |
| 706.8 | Other specified diseases of sebaceous glands | 165 | 17630882 | 76 | 1.19 (0.07, 19.49) | 0.909 | 24 | 1.48 (0.06, 34.44) | 0.82 | 5 | 0.77 (0, 40350.96) | 0.965 | 136 | 0.88 (0.01, 65.21) | 0.959 |
| 707 | Chronic ulcer of skin | 2121 | 17617533 | 888 | 0.98 (0.59, 1.62) | 0.929 | 283 | 1.22 (0.99, 1.49) | 0.056 | 89 | 0.95 (0.25, 3.57) | 0.95 | 1789 | 1.01 (0.59, 1.74) | 0.97 |
| 709 | Diffuse diseases of connective tissue | 7298 | 17568552 | 3177 | 1 (0.81, 1.25) | 0.97 | 812 | 0.99 (0.58, 1.69) | 0.964 | 292 | 0.89 (0.68, 1.15) | 0.366 | 6130 | 0.98 (0.72, 1.33) | 0.9 |
| 709.2 | Sicca syndrome | 1150 | 17620001 | 500 | 0.98 (0.48, 2.01) | 0.959 | 113 | 0.85 (0.55, 1.33) | 0.487 | 48 | 0.9 (0.26, 3.19) | 0.883 | 965 | 0.98 (0.37, 2.61) | 0.965 |
| 709.3 | Systemic sclerosis | 393 | 17627732 | 181 | 1.09 (0.29, 4.14) | 0.909 | 38 | 0.87 (0.09, 8.4) | 0.915 | 15 | 0.86 (0.01, 53.26) | 0.949 | 322 | 0.85 (0.24, 2.98) | 0.806 |
| 709.4 | Polymyositis | 171 | 17629821 | 72 | 1.04 (0.19, 5.74) | 0.97 | 25 | 1.39 (0.31, 6.15) | 0.68 | 8 | 1.11 (0.01, 171.61) | 0.97 | 140 | 0.84 (0.13, 5.34) | 0.864 |
| 709.5 | Dermatomyositis | 154 | 17630031 | 61 | 0.9 (0.16, 5.14) | 0.909 | 21 | 1.16 (0.1, 14.18) | 0.912 | 7 | 0.98 (0.38, 2.55) | 0.97 | 122 | 0.7 (0.27, 1.85) | 0.487 |
| 709.6 | Other specified diffuse diseases of connective tissue | 244 | 17629695 | 113 | 1.15 (0.43, 3.11) | 0.79 | 30 | 1.13 (0.16, 8.16) | 0.909 | 7 | 0.67 (0.04, 11.83) | 0.8 | 215 | 1.36 (0.52, 3.61) | 0.544 |
| 709.7 | Unspecified diffuse connective tissue disease | 5544 | 17587148 | 2415 | 1.01 (0.69, 1.49) | 0.965 | 629 | 1.01 (0.59, 1.72) | 0.97 | 220 | 0.88 (0.64, 1.22) | 0.46 | 4663 | 0.99 (0.63, 1.54) | 0.959 |
| 931 | Contact dermatitis and other eczema due to plants [except food] | 159 | 17629457 | 76 | 1.23 (0.44, 3.41) | 0.708 | 19 | 1.17 (0.1, 13.07) | 0.907 | 5 | 0.77 (0.01, 74.53) | 0.92 | 133 | 0.95 (0.09, 9.96) | 0.97 |
| 938 | Dermatitis due to solar radiation | 944 | 17623375 | 435 | 1.12 (0.77, 1.62) | 0.575 | 89 | 0.85 (0.44, 1.63) | 0.63 | 46 | 1.11 (0.25, 4.99) | 0.898 | 793 | 0.97 (0.31, 3.03) | 0.956 |
| 938.1 | Acute dermatitis due to solar radiation | 275 | 17628525 | 133 | 1.25 (0.62, 2.55) | 0.543 | 25 | 0.83 (0.09, 7.84) | 0.883 | 15 | 1.3 (0.1, 16.57) | 0.849 | 233 | 1 (0.97, 1.03) | 0.97 |
| 939 | Atopic/contact dermatitis due to other or unspecified | 15301 | 17456651 | 6582 | 1.02 (0.86, 1.2) | 0.866 | 1784 | 0.99 (0.69, 1.42) | 0.959 | 710 | 1.02 (0.6, 1.73) | 0.952 | 12903 | 0.97 (0.83, 1.14) | 0.748 |
| 947 | Urticaria | 11035 | 17503254 | 4736 | 1.02 (0.86, 1.22) | 0.818 | 1310 | 1.01 (0.68, 1.51) | 0.97 | 530 | 1.06 (0.76, 1.48) | 0.757 | 9390 | 1.03 (0.83, 1.28) | 0.81 |
| **Musculoskeletal** | | | | | | | | | | | | | | | |
| 710 | Osteomyelitis, periostitis, and other infections involving bone | 2069 | 17610654 | 877 | 0.96 (0.53, 1.73) | 0.898 | 227 | 0.94 (0.37, 2.37) | 0.905 | 98 | 1.03 (0.26, 4.02) | 0.97 | 1734 | 0.96 (0.43, 2.14) | 0.923 |
| 710.11 | Acute osteomyelitis | 356 | 17629157 | 167 | 1.16 (0.62, 2.16) | 0.662 | 40 | 1.05 (0.13, 8.48) | 0.97 | 12 | 0.8 (0.07, 9.26) | 0.866 | 293 | 0.86 (0.38, 1.94) | 0.728 |
| 710.12 | Chronic osteomyelitis | 591 | 17627131 | 244 | 0.95 (0.42, 2.11) | 0.899 | 68 | 0.99 (0.52, 1.87) | 0.97 | 34 | 1.26 (0.47, 3.39) | 0.664 | 498 | 0.99 (0.51, 1.9) | 0.97 |
| 710.19 | Unspecified osteomyelitis | 1118 | 17617865 | 475 | 0.95 (0.36, 2.54) | 0.927 | 123 | 0.93 (0.2, 4.38) | 0.935 | 46 | 0.88 (0.09, 8.36) | 0.921 | 931 | 0.92 (0.32, 2.64) | 0.886 |
| 711 | Arthropathy associated with infections | 2482 | 17600788 | 1095 | 1.05 (0.76, 1.45) | 0.771 | 264 | 0.95 (0.47, 1.94) | 0.906 | 135 | 1.24 (0.86, 1.79) | 0.254 | 2060 | 0.9 (0.7, 1.17) | 0.435 |
| 711.1 | Pyogenic arthritis | 1438 | 17614694 | 637 | 1.05 (0.58, 1.9) | 0.892 | 146 | 0.92 (0.37, 2.26) | 0.861 | 79 | 1.25 (0.67, 2.33) | 0.487 | 1194 | 0.91 (0.57, 1.46) | 0.71 |
| 711.2 | Reiter's disease | 704 | 17620531 | 314 | 1.06 (0.5, 2.27) | 0.885 | 72 | 0.91 (0.26, 3.14) | 0.885 | 37 | 1.19 (0.31, 4.6) | 0.813 | 588 | 0.93 (0.36, 2.42) | 0.89 |
| 713 | Arthropathy associated with other disorders classified elsewhere | 3670 | 17593284 | 1576 | 0.99 (0.61, 1.61) | 0.97 | 410 | 0.94 (0.65, 1.34) | 0.73 | 162 | 0.95 (0.41, 2.24) | 0.92 | 3085 | 0.96 (0.67, 1.37) | 0.823 |
| 713.5 | Arthropathy associated with neurological disorders | 357 | 17629368 | 166 | 1.14 (0.38, 3.37) | 0.826 | 35 | 0.95 (0.08, 11.5) | 0.97 | 19 | 1.29 (0.09, 17.5) | 0.86 | 300 | 0.99 (0.64, 1.53) | 0.97 |
| 714 | Rheumatoid arthritis and other inflammatory polyarthropathies | 8313 | 17536672 | 3635 | 1.02 (0.8, 1.29) | 0.901 | 897 | 0.96 (0.72, 1.29) | 0.802 | 370 | 1 (0.79, 1.26) | 0.97 | 6959 | 0.96 (0.8, 1.15) | 0.664 |
| 714.1 | Rheumatoid arthritis | 7303 | 17550362 | 3197 | 1.02 (0.8, 1.3) | 0.883 | 792 | 0.98 (0.64, 1.51) | 0.935 | 328 | 1.01 (0.52, 1.97) | 0.97 | 6096 | 0.95 (0.82, 1.1) | 0.487 |
| 714.2 | Juvenile rheumatoid arthritis | 795 | 17618974 | 346 | 1.02 (0.38, 2.78) | 0.97 | 84 | 0.82 (0.38, 1.78) | 0.624 | 35 | 0.91 (0.1, 8.32) | 0.938 | 681 | 1.04 (0.22, 4.88) | 0.963 |
| 715 | Other inflammatory spondylopathies | 1494 | 17623093 | 658 | 1.03 (0.64, 1.66) | 0.903 | 161 | 0.93 (0.51, 1.67) | 0.808 | 66 | 0.97 (0.29, 3.24) | 0.97 | 1272 | 1.05 (0.56, 1.96) | 0.897 |
| 715.1 | Sacroiliitis NEC | 366 | 17628547 | 160 | 1.11 (0.56, 2.22) | 0.773 | 47 | 1.14 (0.34, 3.79) | 0.842 | 20 | 1.27 (0.35, 4.67) | 0.731 | 308 | 0.95 (0.22, 4.09) | 0.946 |
| 716 | Other arthropathies | 39797 | 17262195 | 17565 | 1.03 (0.98, 1.08) | 0.294 | 4217 | 0.97 (0.88, 1.05) | 0.443 | 1786 | 1.02 (0.79, 1.32) | 0.883 | 33420 | 0.99 (0.86, 1.13) | 0.864 |
| 716.2 | Unspecified monoarthritis | 15585 | 17468668 | 6926 | 1.05 (0.97, 1.13) | 0.234 | 1668 | 1 (0.97, 1.03) | 0.97 | 725 | 1.08 (0.87, 1.33) | 0.494 | 13027 | 0.96 (0.84, 1.1) | 0.604 |
| 716.8 | Palindromic rheumatism | 338 | 17627150 | 141 | 0.92 (0.27, 3.09) | 0.897 | 37 | 0.92 (0.09, 9.63) | 0.947 | 14 | 0.88 (0.02, 31.83) | 0.948 | 288 | 1.07 (0.14, 8.24) | 0.953 |
| 716.9 | Arthropathy NOS | 38702 | 17273238 | 17075 | 1.03 (0.97, 1.08) | 0.341 | 4101 | 0.97 (0.88, 1.06) | 0.487 | 1738 | 1.02 (0.78, 1.34) | 0.883 | 32504 | 0.99 (0.85, 1.15) | 0.897 |
| 717 | Polymyalgia Rheumatica | 3824 | 17601525 | 1657 | 0.96 (0.8, 1.16) | 0.695 | 392 | 0.93 (0.71, 1.23) | 0.626 | 150 | 0.87 (0.62, 1.22) | 0.428 | 3207 | 1 (0.99, 1.01) | 0.97 |
| 720 | Spinal stenosis | 14969 | 17531168 | 6571 | 1.02 (0.93, 1.13) | 0.635 | 1653 | 1.04 (0.89, 1.21) | 0.664 | 684 | 1.06 (0.86, 1.31) | 0.62 | 12676 | 1.05 (0.98, 1.14) | 0.175 |
| 721 | Spondylosis and allied disorders | 14782 | 17495231 | 6384 | 0.99 (0.78, 1.26) | 0.955 | 1657 | 1 (0.85, 1.19) | 0.97 | 649 | 0.98 (0.58, 1.67) | 0.952 | 12469 | 1.01 (0.79, 1.3) | 0.935 |
| 721.1 | Spondylosis without myelopathy | 10019 | 17542827 | 4325 | 1 (0.92, 1.08) | 0.97 | 1141 | 1.03 (0.78, 1.37) | 0.826 | 455 | 1.03 (0.58, 1.83) | 0.934 | 8457 | 1.02 (0.77, 1.35) | 0.906 |
| 721.2 | Spondylosis with myelopathy | 1402 | 17621872 | 577 | 0.92 (0.76, 1.11) | 0.372 | 162 | 1.01 (0.66, 1.54) | 0.97 | 68 | 1.06 (0.38, 2.93) | 0.92 | 1190 | 1.05 (0.65, 1.7) | 0.84 |
| 721.8 | Other allied disorders of spine | 2430 | 17609824 | 1086 | 1.04 (0.73, 1.47) | 0.844 | 261 | 0.93 (0.61, 1.4) | 0.73 | 84 | 0.76 (0.53, 1.09) | 0.136 | 2041 | 0.96 (0.59, 1.58) | 0.893 |
| 722 | Intervertebral disc disorders | 17488 | 17451804 | 7667 | 1.03 (0.96, 1.11) | 0.36 | 1979 | 1.02 (0.8, 1.29) | 0.901 | 785 | 1.02 (0.68, 1.52) | 0.935 | 14782 | 1.02 (0.84, 1.23) | 0.883 |
| 722.1 | Displacement of intervertebral disc | 555 | 17627086 | 233 | 0.93 (0.55, 1.57) | 0.791 | 59 | 0.88 (0.4, 1.95) | 0.77 | 24 | 0.92 (0.15, 5.47) | 0.929 | 481 | 1.2 (0.77, 1.88) | 0.434 |
| 722.6 | Degeneration of intervertebral disc | 10783 | 17533724 | 4746 | 1.04 (0.96, 1.12) | 0.323 | 1205 | 1.01 (0.75, 1.36) | 0.97 | 490 | 1.03 (0.69, 1.54) | 0.883 | 9090 | 1 (0.91, 1.09) | 0.97 |
| 722.7 | Intervertebral disc disorder with myelopathy | 747 | 17625461 | 313 | 0.96 (0.49, 1.9) | 0.92 | 104 | 1.22 (0.84, 1.77) | 0.303 | 22 | 0.65 (0.34, 1.27) | 0.208 | 627 | 0.97 (0.34, 2.72) | 0.953 |
| 722.8 | Postlaminectomy syndrome | 133 | 17630310 | 57 | 1.04 (0.17, 6.36) | 0.97 | 17 | 1.19 (0.13, 10.61) | 0.883 | 7 | 1.24 (0.05, 28.82) | 0.903 | 113 | 1.05 (0.1, 11.11) | 0.97 |
| 722.9 | Other and unspecified disc disorder | 7756 | 17542150 | 3389 | 1.02 (0.81, 1.29) | 0.861 | 879 | 1.01 (0.59, 1.73) | 0.97 | 341 | 0.99 (0.66, 1.5) | 0.97 | 6572 | 1.03 (0.79, 1.34) | 0.829 |
| 723 | Other disorders of cervical region | 1408 | 17616363 | 620 | 1.09 (0.78, 1.52) | 0.624 | 180 | 1.16 (0.75, 1.79) | 0.527 | 65 | 1.07 (0.26, 4.52) | 0.929 | 1189 | 0.99 (0.53, 1.84) | 0.97 |
| 723.1 | Torticollis | 506 | 17623905 | 224 | 1.15 (0.59, 2.23) | 0.691 | 63 | 1.11 (0.24, 5.06) | 0.901 | 31 | 1.44 (0.53, 3.94) | 0.487 | 428 | 0.97 (0.23, 4.12) | 0.97 |
| 724 | Other and unspecified disorders of back | 1254 | 17619405 | 542 | 1.01 (0.53, 1.95) | 0.97 | 153 | 1.09 (0.53, 2.25) | 0.823 | 52 | 0.94 (0.16, 5.55) | 0.949 | 1072 | 1.09 (0.63, 1.91) | 0.767 |
| 724.9 | Other unspecified back disorders | 1177 | 17620139 | 501 | 0.99 (0.5, 1.95) | 0.97 | 146 | 1.1 (0.52, 2.32) | 0.823 | 48 | 0.91 (0.21, 4.01) | 0.909 | 1001 | 1.05 (0.48, 2.33) | 0.903 |
| 726 | Peripheral enthesopathies and allied syndromes | 48003 | 17188966 | 20813 | 1.01 (0.91, 1.11) | 0.898 | 5337 | 0.97 (0.91, 1.03) | 0.307 | 2172 | 1 (0.94, 1.07) | 0.97 | 40354 | 0.97 (0.93, 1.02) | 0.212 |
| 726.1 | Enthesopathy | 24109 | 17410204 | 10556 | 1.03 (0.96, 1.1) | 0.459 | 2637 | 0.96 (0.87, 1.05) | 0.366 | 1100 | 1.02 (0.71, 1.46) | 0.935 | 20224 | 0.96 (0.9, 1.02) | 0.178 |
| 726.2 | Synoviopathy | 731 | 17625434 | 310 | 0.92 (0.41, 2.07) | 0.859 | 73 | 0.83 (0.34, 2.04) | 0.7 | 29 | 0.83 (0.12, 5.74) | 0.861 | 616 | 1 (0.79, 1.26) | 0.97 |
| 726.3 | Bursitis | 6323 | 17566199 | 2837 | 1.05 (0.93, 1.18) | 0.48 | 673 | 0.94 (0.76, 1.16) | 0.576 | 248 | 0.88 (0.68, 1.13) | 0.323 | 5276 | 0.93 (0.83, 1.05) | 0.236 |
| 726.4 | Calcaneal spur; Exostosis NOS | 721 | 17625119 | 314 | 1.03 (0.33, 3.25) | 0.962 | 83 | 1.01 (0.55, 1.87) | 0.97 | 33 | 1.03 (0.3, 3.54) | 0.97 | 607 | 0.98 (0.33, 2.88) | 0.97 |
| 727 | Other disorders of synovium, tendon, and bursa | 30487 | 17272753 | 13190 | 1 (0.88, 1.14) | 0.97 | 3348 | **0.94 (0.88, 0.99)** | **0.028** | 1370 | 0.98 (0.73, 1.32) | 0.912 | 25643 | 0.97 (0.9, 1.04) | 0.364 |
| 727.1 | Synovitis and tenosynovitis | 15197 | 17441078 | 6660 | 1.03 (0.92, 1.14) | 0.648 | 1667 | 0.95 (0.82, 1.1) | 0.508 | 667 | 0.97 (0.64, 1.47) | 0.902 | 12746 | 0.95 (0.87, 1.05) | 0.342 |
| 727.2 | Bursitis disorders | 382 | 17627568 | 163 | 0.94 (0.34, 2.6) | 0.909 | 40 | 0.87 (0.19, 3.92) | 0.863 | 14 | 0.77 (0.1, 6.1) | 0.818 | 323 | 1.01 (0.61, 1.67) | 0.97 |
| 727.4 | Ganglion and cyst of synovium, tendon, and bursa | 7134 | 17550147 | 3063 | 0.99 (0.73, 1.33) | 0.938 | 761 | 0.89 (0.77, 1.02) | 0.103 | 332 | 1 (0.95, 1.06) | 0.97 | 6045 | 1.01 (0.63, 1.62) | 0.97 |
| 727.5 | Rupture of synovium | 3226 | 17601154 | 1428 | 1.06 (0.87, 1.29) | 0.597 | 370 | 1.07 (0.75, 1.53) | 0.722 | 147 | 1.06 (0.48, 2.36) | 0.895 | 2706 | 0.98 (0.59, 1.61) | 0.933 |
| 727.6 | Rupture of tendon, nontraumatic | 811 | 17623979 | 325 | 0.87 (0.67, 1.12) | 0.284 | 89 | 0.91 (0.37, 2.24) | 0.856 | 42 | 1.08 (0.27, 4.41) | 0.917 | 674 | 0.92 (0.53, 1.59) | 0.77 |
| 727.7 | Contracture of tendon (sheath) | 391 | 17628006 | 157 | 0.94 (0.32, 2.69) | 0.909 | 53 | 1.11 (0.25, 4.88) | 0.898 | 20 | 1.09 (0.05, 24.22) | 0.958 | 307 | **0.65 (0.47, 0.9)** | **0.009** |
| 728 | Disorders of muscle, ligament, and fascia | 11338 | 17510763 | 5096 | 1.06 (0.98, 1.13) | 0.142 | 1204 | 0.95 (0.8, 1.12) | 0.541 | 453 | 0.9 (0.73, 1.11) | 0.319 | 9452 | 0.93 (0.86, 1.01) | 0.079 |
| 728.1 | Muscular calcification and ossification | 134 | 17630274 | 73 | 1.36 (0.71, 2.61) | 0.364 | 10 | 0.7 (0.11, 4.42) | 0.718 | 0 | 0 (0, Inf) | 0.97 | 114 | 1.06 (0.08, 14.08) | 0.97 |
| 728.2 | Laxity of ligament or hypermobility syndrome | 1949 | 17607585 | 861 | 1.04 (0.66, 1.62) | 0.886 | 201 | 0.82 (0.63, 1.07) | 0.14 | 79 | 0.85 (0.43, 1.67) | 0.651 | 1644 | 0.95 (0.55, 1.64) | 0.866 |
| 728.7 | Fasciitis | 1913 | 17614537 | 854 | 1.08 (0.83, 1.4) | 0.592 | 218 | 1.02 (0.45, 2.33) | 0.97 | 83 | 0.99 (0.62, 1.59) | 0.97 | 1592 | 0.91 (0.67, 1.23) | 0.543 |
| 728.71 | Contracture of palmar fascia [Dupuytren's disease] | 5857 | 17571350 | 2672 | 1.08 (0.97, 1.19) | 0.155 | 624 | 1.02 (0.53, 1.93) | 0.966 | 231 | 0.92 (0.59, 1.44) | 0.733 | 4870 | 0.94 (0.79, 1.11) | 0.48 |
| 729 | Other disorders of soft tissues | 32004 | 17269078 | 13461 | 0.98 (0.9, 1.07) | 0.725 | 3896 | 1.03 (0.93, 1.15) | 0.578 | 1519 | 1.04 (0.86, 1.25) | 0.71 | 27091 | 1 (0.99, 1.01) | 0.97 |
| 729.1 | Rheumatism, unspecified and fibrositis | 2236 | 17601140 | 998 | 1.08 (0.88, 1.33) | 0.487 | 270 | 1.11 (0.79, 1.56) | 0.551 | 81 | 0.83 (0.48, 1.45) | 0.531 | 1905 | 1.07 (0.75, 1.53) | 0.721 |
| 729.3 | Panniculitis | 210 | 17630071 | 89 | 1.01 (0.5, 2.04) | 0.97 | 27 | 1.12 (0.15, 8.28) | 0.92 | 10 | 1.07 (0.05, 24.95) | 0.97 | 180 | 1.09 (0.17, 7.16) | 0.935 |
| 731 | Osteitis deformans and osteopathies associated with other disorders classified elsewhere | 198 | 17630360 | 76 | 0.79 (0.13, 4.97) | 0.815 | 22 | 0.83 (0.02, 40.43) | 0.93 | 8 | 0.78 (0, 697.19) | 0.947 | 160 | 0.76 (0.09, 6.45) | 0.814 |
| 732 | Osteochondropathies | 4133 | 17557183 | 1865 | 1.1 (0.96, 1.26) | 0.189 | 448 | 0.95 (0.55, 1.63) | 0.861 | 193 | 1.06 (0.47, 2.4) | 0.901 | 3449 | 0.91 (0.75, 1.11) | 0.364 |
| 732.1 | Juvenile osteochondrosis | 2168 | 17593280 | 977 | 1.11 (0.89, 1.4) | 0.353 | 248 | 1 (0.8, 1.27) | 0.97 | 103 | 1.09 (0.34, 3.48) | 0.898 | 1825 | 0.95 (0.49, 1.84) | 0.897 |
| 732.7 | Osteochondritis dissecans | 1214 | 17609206 | 569 | 1.16 (0.93, 1.45) | 0.18 | 129 | 0.96 (0.3, 3.03) | 0.944 | 48 | 0.92 (0.19, 4.41) | 0.923 | 1024 | 0.98 (0.34, 2.78) | 0.97 |
| 733 | Other disorders of bone and cartilage | 6381 | 17577934 | 2756 | 0.99 (0.68, 1.44) | 0.97 | 683 | 0.92 (0.72, 1.18) | 0.531 | 290 | 0.99 (0.75, 1.32) | 0.97 | 5352 | 0.96 (0.73, 1.26) | 0.776 |
| 733.2 | Cyst of bone | 372 | 17627626 | 173 | 1.14 (0.44, 2.96) | 0.802 | 33 | 0.77 (0.19, 3.07) | 0.723 | 20 | 1.21 (0.09, 15.96) | 0.893 | 307 | 0.85 (0.28, 2.57) | 0.784 |
| 733.4 | Aseptic necrosis of bone | 735 | 17627188 | 297 | 0.87 (0.62, 1.23) | 0.455 | 77 | 0.87 (0.42, 1.78) | 0.71 | 38 | 1.08 (0.2, 5.76) | 0.936 | 632 | 1.14 (0.64, 2.04) | 0.67 |
| 733.8 | Malunion and nonunion of fracture | 1459 | 17617457 | 658 | 1.07 (0.66, 1.72) | 0.796 | 157 | 0.96 (0.26, 3.63) | 0.959 | 59 | 0.92 (0.18, 4.61) | 0.923 | 1210 | 0.9 (0.57, 1.41) | 0.648 |
| 735 | Acquired foot deformities | 14277 | 17464828 | 6191 | 1.01 (0.85, 1.21) | 0.889 | 1605 | 0.99 (0.76, 1.3) | 0.97 | 637 | 0.99 (0.73, 1.35) | 0.97 | 12045 | 1 (0.98, 1.02) | 0.97 |
| 735.1 | Flat foot | 1059 | 17620655 | 456 | 1.01 (0.56, 1.85) | 0.97 | 117 | 0.95 (0.27, 3.4) | 0.944 | 54 | 1.12 (0.26, 4.77) | 0.883 | 899 | 1.03 (0.32, 3.34) | 0.97 |
| 735.2 | Acquired toe deformities | 913 | 17623820 | 384 | 0.98 (0.46, 2.12) | 0.97 | 107 | 1.03 (0.31, 3.44) | 0.97 | 48 | 1.17 (0.32, 4.2) | 0.825 | 757 | 0.89 (0.49, 1.62) | 0.723 |
| 735.21 | Hammer toe (acquired) | 2739 | 17604770 | 1215 | 1.04 (0.71, 1.51) | 0.868 | 286 | 0.95 (0.49, 1.85) | 0.897 | 120 | 0.99 (0.72, 1.38) | 0.97 | 2281 | 0.94 (0.68, 1.3) | 0.713 |
| 735.23 | Hallux rigidus | 2497 | 17607012 | 1108 | 1.08 (0.86, 1.35) | 0.527 | 295 | 1.11 (0.78, 1.59) | 0.576 | 115 | 1.08 (0.41, 2.83) | 0.883 | 2084 | 0.94 (0.63, 1.42) | 0.793 |
| 735.3 | Hallux valgus (Bunion) | 8115 | 17524435 | 3506 | 1.01 (0.76, 1.34) | 0.944 | 925 | 1.01 (0.69, 1.47) | 0.97 | 357 | 0.98 (0.47, 2.02) | 0.958 | 6871 | 1.02 (0.77, 1.36) | 0.874 |
| 736 | Other acquired deformities of limbs | 1985 | 17610350 | 893 | 1.06 (0.76, 1.49) | 0.742 | 204 | 0.87 (0.57, 1.34) | 0.544 | 82 | 0.91 (0.27, 3.05) | 0.883 | 1655 | 0.91 (0.64, 1.29) | 0.6 |
| 736.2 | Acquired deformities of finger | 220 | 17629527 | 100 | 1.03 (0.28, 3.84) | 0.97 | 21 | 0.81 (0.08, 8.61) | 0.868 | 6 | 0.58 (0.03, 11.07) | 0.733 | 172 | 0.66 (0.34, 1.27) | 0.214 |
| 737 | Curvature of spine | 4741 | 17568466 | 2056 | 1.04 (0.78, 1.39) | 0.805 | 559 | 1 (0.88, 1.13) | 0.97 | 219 | 1.01 (0.53, 1.93) | 0.97 | 3997 | 0.96 (0.62, 1.51) | 0.883 |
| 737.1 | Kyphosis (acquired) | 940 | 17618655 | 412 | 1.06 (0.52, 2.18) | 0.883 | 109 | 1.06 (0.3, 3.69) | 0.935 | 49 | 1.21 (0.44, 3.32) | 0.731 | 808 | 1.13 (0.62, 2.07) | 0.7 |
| 737.3 | Kyphoscoliosis and scoliosis | 3889 | 17581539 | 1683 | 1.03 (0.68, 1.57) | 0.887 | 455 | 0.97 (0.4, 2.34) | 0.955 | 176 | 0.98 (0.38, 2.52) | 0.97 | 3266 | 0.94 (0.66, 1.34) | 0.726 |
| 738 | Other acquired musculoskeletal deformity | 7313 | 17564068 | 3133 | 1.01 (0.79, 1.31) | 0.924 | 869 | 1.04 (0.83, 1.32) | 0.733 | 359 | 1.1 (0.86, 1.41) | 0.455 | 6202 | 1.02 (0.77, 1.37) | 0.883 |
| 738.4 | Acquired spondylolisthesis | 4123 | 17597056 | 1787 | 1.03 (0.78, 1.37) | 0.852 | 475 | 1.06 (0.76, 1.48) | 0.752 | 211 | 1.18 (0.91, 1.52) | 0.205 | 3496 | 1.04 (0.76, 1.43) | 0.808 |
| 739 | Contracture of joint | 677 | 17625173 | 279 | 0.91 (0.48, 1.7) | 0.771 | 72 | 0.84 (0.35, 2.01) | 0.7 | 31 | 0.93 (0.06, 15.61) | 0.965 | 561 | 0.87 (0.44, 1.71) | 0.7 |
| 740 | Osteoarthrosis | 53711 | 17187748 | 23474 | 1 (1, 1) | 0.97 | 5629 | **0.95 (0.9, 0.99)** | **0.022** | 2411 | 1.01 (0.74, 1.38) | 0.956 | 44931 | 0.97 (0.93, 1.01) | 0.131 |
| 740.1 | Osteoarthritis; localized | 10926 | 17523893 | 4753 | 0.99 (0.7, 1.4) | 0.959 | 1149 | 0.96 (0.69, 1.33) | 0.815 | 492 | 1.01 (0.58, 1.77) | 0.97 | 9106 | 0.95 (0.79, 1.14) | 0.604 |
| 740.11 | Osteoarthrosis, localized, primary | 40386 | 17333061 | 17695 | 1 (0.9, 1.11) | 0.97 | 4178 | **0.94 (0.9, 0.98)** | **0.006** | 1761 | 0.98 (0.78, 1.22) | 0.861 | 33774 | 0.97 (0.92, 1.02) | 0.192 |
| 740.12 | Osteoarthrosis, localized, secondary | 2064 | 17615823 | 919 | 1.03 (0.69, 1.54) | 0.883 | 214 | 0.94 (0.55, 1.6) | 0.823 | 88 | 0.96 (0.27, 3.41) | 0.959 | 1749 | 1.04 (0.63, 1.73) | 0.883 |
| 740.2 | Osteoarthrosis, generalized | 574 | 17627350 | 244 | 0.98 (0.37, 2.62) | 0.97 | 63 | 1.02 (0.41, 2.55) | 0.97 | 31 | 1.23 (0.5, 3.02) | 0.663 | 473 | 0.89 (0.52, 1.53) | 0.695 |
| 740.9 | Osteoarthrosis NOS | 10961 | 17542347 | 4780 | 1.01 (0.83, 1.24) | 0.898 | 1167 | 0.95 (0.79, 1.15) | 0.638 | 534 | 1.1 (0.91, 1.33) | 0.342 | 9163 | 0.95 (0.85, 1.06) | 0.377 |
| 741 | Symptoms and disorders of the joints | 5085 | 17578450 | 2149 | 0.95 (0.81, 1.11) | 0.501 | 531 | **0.82 (0.72, 0.94)** | **0.005** | 220 | 0.89 (0.6, 1.33) | 0.578 | 4314 | 1 (0.82, 1.24) | 0.97 |
| 741.2 | Stiffness of joint | 100 | 17630907 | 47 | 1.15 (0.26, 5.09) | 0.861 | 8 | 0.72 (0.09, 5.59) | 0.763 | 6 | 1.38 (0.1, 19.85) | 0.826 | 80 | 0.73 (0.28, 1.91) | 0.531 |
| 741.3 | Difficulty in walking | 603 | 17628321 | 288 | 1.13 (0.83, 1.54) | 0.431 | 56 | 0.86 (0.43, 1.75) | 0.697 | 18 | 0.69 (0.31, 1.52) | 0.364 | 511 | 1.05 (0.41, 2.66) | 0.929 |
| 741.4 | Joint effusions | 823 | 17619526 | 337 | 0.89 (0.6, 1.32) | 0.578 | 85 | 0.85 (0.42, 1.72) | 0.672 | 43 | 1.09 (0.22, 5.56) | 0.92 | 693 | 0.99 (0.56, 1.74) | 0.97 |
| 741.5 | Hemarthrosis | 290 | 17627454 | 114 | 0.81 (0.44, 1.48) | 0.503 | 32 | 0.88 (0.14, 5.61) | 0.898 | 11 | 0.76 (0.06, 9.66) | 0.842 | 245 | 1.02 (0.47, 2.18) | 0.97 |
| 742 | Derangement of joint, non-traumatic | 16652 | 17438176 | 7225 | 1.01 (0.83, 1.23) | 0.925 | 1811 | **0.9 (0.83, 0.97)** | **0.009** | 748 | 0.97 (0.64, 1.46) | 0.883 | 14005 | 0.95 (0.87, 1.04) | 0.3 |
| 742.1 | Loose body in joint | 1608 | 17597561 | 713 | 1.03 (0.63, 1.7) | 0.902 | 176 | 0.97 (0.33, 2.87) | 0.958 | 67 | 0.94 (0.25, 3.49) | 0.929 | 1354 | 0.99 (0.5, 1.95) | 0.97 |
| 742.2 | Pathological, developmental or recurrent dislocation | 1766 | 17610215 | 789 | 1.09 (0.81, 1.47) | 0.596 | 190 | 0.86 (0.57, 1.32) | 0.504 | 79 | 0.96 (0.17, 5.46) | 0.966 | 1499 | 0.98 (0.37, 2.59) | 0.966 |
| 742.8 | Articular cartilage disorder | 1354 | 17621140 | 582 | 1 (0.85, 1.17) | 0.97 | 151 | 0.93 (0.52, 1.67) | 0.818 | 63 | 1.01 (0.71, 1.44) | 0.97 | 1125 | 0.89 (0.69, 1.13) | 0.341 |
| 742.9 | Other derangement of joint | 13669 | 17490182 | 5892 | 1 (0.94, 1.06) | 0.97 | 1495 | **0.9 (0.83, 0.99)** | **0.027** | 619 | 0.97 (0.62, 1.52) | 0.908 | 11485 | 0.95 (0.86, 1.04) | 0.268 |
| 743 | Osteoporosis, osteopenia and pathological fracture | 15875 | 17536424 | 7091 | 1.04 (0.96, 1.12) | 0.352 | 1632 | 0.96 (0.82, 1.12) | 0.576 | 647 | 0.93 (0.75, 1.16) | 0.544 | 13332 | 1 (0.98, 1.02) | 0.97 |
| 743.11 | Osteoporosis NOS | 13633 | 17553976 | 6114 | 1.04 (0.99, 1.1) | 0.087 | 1381 | 0.95 (0.85, 1.05) | 0.314 | 564 | 0.95 (0.76, 1.19) | 0.674 | 11440 | 1 (0.82, 1.21) | 0.97 |
| 743.2 | Pathologic fracture | 1002 | 17626723 | 435 | 0.94 (0.4, 2.23) | 0.901 | 92 | 0.81 (0.34, 1.9) | 0.636 | 40 | 0.86 (0.11, 6.94) | 0.898 | 827 | 0.91 (0.36, 2.29) | 0.845 |
| 743.21 | Pathologic fracture of vertebrae | 618 | 17629988 | 273 | 0.98 (0.35, 2.75) | 0.97 | 62 | 0.9 (0.18, 4.59) | 0.91 | 19 | 0.68 (0.13, 3.62) | 0.663 | 503 | 0.84 (0.42, 1.66) | 0.619 |
| 743.9 | Osteopenia or other disorder of bone and cartilage | 1510 | 17617459 | 675 | 1.04 (0.58, 1.89) | 0.898 | 164 | 0.98 (0.31, 3.04) | 0.97 | 54 | 0.8 (0.33, 1.96) | 0.644 | 1282 | 1.05 (0.49, 2.27) | 0.906 |
| 745 | Pain in joint | 8444 | 17555591 | 3574 | 0.99 (0.76, 1.28) | 0.92 | 991 | 1 (0.97, 1.03) | 0.97 | 400 | 1.03 (0.6, 1.79) | 0.911 | 7121 | 0.98 (0.71, 1.35) | 0.915 |
| **Congenital Anomalies** | | | | | | | | | | | | | | | |
| 747 | Cardiac and circulatory congenital anomalies | 15297 | 482914* | 6831 | **1.07 (1.01, 1.14)**** | **0.022** | 1685 | 0.98 (0.71, 1.34)** | 0.893 | 710 | 1.05 (0.75, 1.48)** | 0.784 | 12876 | 0.98 (0.77, 1.25)** | 0.885 |
| 747.1 | Cardiac congenital anomalies | 1621 | 482914* | 748 | 1.15 (0.97, 1.38)** | 0.115 | 164 | 0.83 (0.58, 1.19)** | 0.314 | 79 | 1.07 (0.34, 3.43)** | 0.911 | 1384 | 1.02 (0.48, 2.16)** | 0.97 |
| 747.11 | Cardiac shunt/ heart septal defect | 2898 | 482914* | 1254 | 1.06 (0.78, 1.44)** | 0.721 | 351 | 0.98 (0.37, 2.58)** | 0.963 | 144 | 1.08 (0.41, 2.87)** | 0.888 | 2497 | 1.09 (0.76, 1.58)** | 0.652 |
| 747.12 | Valvular heart disease/ heart chambers | 1413 | 482914* | 625 | 1.11 (0.76, 1.61)** | 0.609 | 178 | 1.06 (0.38, 2.99)** | 0.921 | 65 | 1.03 (0.21, 4.99)** | 0.97 | 1207 | 1.03 (0.34, 3.11)** | 0.959 |
| 747.13 | Congenital anomalies of great vessels | 9896 | 482914* | 4465 | 1.07 (0.99, 1.16)** | 0.096 | 1061 | 0.99 (0.7, 1.4)** | 0.97 | 435 | 1.01 (0.54, 1.89)** | 0.97 | 8257 | 0.95 (0.81, 1.12)** | 0.576 |
| 747.2 | Congenital anomalies of peripheral vascular system | 1074 | 482914* | 465 | 1.09 (0.36, 3.34)** | 0.883 | 140 | 1.16 (0.25, 5.31)** | 0.863 | 60 | 1.3 (0.23, 7.26)** | 0.781 | 904 | 0.95 (0.16, 5.79)** | 0.959 |
| 748 | Anomalies of respiratory system, congenital | 670 | 482914* | 289 | 1.11 (0.58, 2.1)** | 0.767 | 89 | 1.13 (0.4, 3.2)** | 0.825 | 39 | 1.33 (0.5, 3.52)** | 0.575 | 574 | 1.05 (0.24, 4.65)** | 0.953 |
| 749 | Congenital anomalies of face and neck | 7549 | 482914* | 3292 | 1.05 (0.93, 1.18)** | 0.432 | 866 | 0.93 (0.77, 1.12)** | 0.438 | 346 | 0.99 (0.53, 1.84)** | 0.97 | 6353 | 0.93 (0.82, 1.06)** | 0.284 |
| 749.1 | Cleft palate | 546 | 482914* | 244 | 1.18 (0.72, 1.92)** | 0.528 | 71 | 1.14 (0.35, 3.69)** | 0.84 | 30 | 1.29 (0.37, 4.48)** | 0.706 | 483 | 1.34 (0.77, 2.32)** | 0.303 |
| 749.2 | Congenital anomalies of skull and face bones | 485 | 482914* | 190 | 0.89 (0.5, 1.58)** | 0.707 | 61 | 0.92 (0.26, 3.28)** | 0.902 | 27 | 1.11 (0.14, 8.54)** | 0.929 | 409 | 0.93 (0.31, 2.83)** | 0.906 |
| 750 | Digestive congenital anomalies | 3082 | 482914* | 1366 | 1.06 (0.83, 1.37)** | 0.644 | 351 | 0.95 (0.51, 1.78)** | 0.885 | 130 | 0.93 (0.35, 2.44)** | 0.885 | 2629 | 1.04 (0.59, 1.82)** | 0.897 |
| 750.1 | Upper gastrointestinal congenital anomalies | 134 | 482914* | 60 | 1.32 (0.34, 5.1)** | 0.7 | 22 | 1.61 (0.38, 6.79)** | 0.528 | 8 | 1.57 (0.08, 32.72)** | 0.784 | 116 | 1.13 (0.04, 33.76)** | 0.949 |
| 750.11 | Esophageal atresia/tracheoesophageal fistula | 143 | 482914* | 51 | 0.76 (0.33, 1.77)** | 0.541 | 20 | 0.96 (0.13, 6.92)** | 0.97 | 7 | 0.92 (0.02, 54.3)** | 0.97 | 122 | 1.01 (0.64, 1.59)** | 0.97 |
| 750.13 | Congenital anomalies of mouth/tongue | 338 | 482914* | 164 | 1.22 (0.71, 2.12)** | 0.48 | 31 | 0.76 (0.25, 2.31)** | 0.642 | 13 | 0.86 (0.04, 18.09)** | 0.927 | 288 | 1 (0.89, 1.12)** | 0.97 |
| 750.15 | Congenital anomalies of stomach | 961 | 482914* | 423 | 1.03 (0.39, 2.73)** | 0.956 | 109 | 1.02 (0.34, 3.12)** | 0.97 | 40 | 0.94 (0.08, 11.41)** | 0.966 | 821 | 1.1 (0.49, 2.46)** | 0.835 |
| 750.21 | Congenital anomalies of intestine | 863 | 482914* | 399 | 1.17 (0.83, 1.63)** | 0.376 | 93 | 0.9 (0.3, 2.73)** | 0.866 | 40 | 1.04 (0.14, 7.55)** | 0.97 | 746 | 1.13 (0.55, 2.31)** | 0.758 |
| 750.22 | Congenital anomaly of gallbladder, bile ducts, liver, pancreas | 291 | 482914* | 119 | 0.89 (0.27, 2.98)** | 0.861 | 31 | 0.81 (0.16, 4.05)** | 0.805 | 12 | 0.82 (0.04, 18.65)** | 0.91 | 245 | 0.96 (0.16, 5.69)** | 0.97 |
| 750.5 | Congenital hypertrophic pyloric stenosis | 363 | 482914* | 154 | 0.97 (0.19, 4.99)** | 0.97 | 44 | 0.9 (0.15, 5.61)** | 0.921 | 10 | 0.55 (0.11, 2.74)** | 0.474 | 306 | 0.92 (0.18, 4.61)** | 0.929 |
| 751 | Genitourinary congenital anomalies | 8973 | 482914* | 3830 | 0.99 (0.7, 1.38)** | 0.94 | 1026 | 0.93 (0.73, 1.2)** | 0.59 | 398 | 0.94 (0.5, 1.77)** | 0.87 | 7556 | 0.95 (0.75, 1.21)** | 0.71 |
| 751.1 | Congenital anomalies of genital organs | 457 | 482914* | 207 | 1.1 (0.33, 3.68)** | 0.883 | 56 | 1.05 (0.08, 13.11)** | 0.97 | 14 | 0.69 (0.07, 7.06)** | 0.767 | 392 | 1.08 (0.18, 6.41)** | 0.935 |
| 751.11 | Congenital anomalies of female genital organs | 1557 | 291649* | 682 | 1.05 (0.5, 2.21)** | 0.901 | 166 | 0.87 (0.39, 1.93)** | 0.742 | 75 | 1.04 (0.19, 5.73)** | 0.97 | 1300 | 0.89 (0.48, 1.65)** | 0.733 |
| 751.12 | Congenital anomalies of male genital organs | 3595 | 191265* | 1539 | 0.99 (0.56, 1.74)** | 0.968 | 422 | 0.96 (0.55, 1.67)** | 0.884 | 154 | 0.91 (0.46, 1.82)** | 0.806 | 3051 | 1 (0.85, 1.17)** | 0.97 |
| 751.2 | Congenital anomalies of urinary system | 671 | 482914* | 302 | 1.11 (0.63, 1.98)** | 0.727 | 74 | 0.92 (0.23, 3.66)** | 0.912 | 33 | 1.09 (0.1, 12.03)** | 0.947 | 567 | 0.96 (0.21, 4.49)** | 0.963 |
| 751.21 | Cystic kidney disease | 1875 | 482914* | 787 | 0.93 (0.67, 1.31)** | 0.7 | 207 | 0.93 (0.44, 1.98)** | 0.864 | 81 | 0.92 (0.27, 3.16)** | 0.905 | 1563 | 0.93 (0.58, 1.48)** | 0.767 |
| 751.22 | Other specified congenital anomalies of kidney | 668 | 482914* | 284 | 1.01 (0.55, 1.88)** | 0.97 | 78 | 0.95 (0.17, 5.24)** | 0.961 | 35 | 1.13 (0.19, 6.72)** | 0.901 | 565 | 0.98 (0.29, 3.23)** | 0.97 |
| 751.3 | Obstructive genitourinary defect | 835 | 482914* | 363 | 1.07 (0.43, 2.61)** | 0.898 | 97 | 0.93 (0.2, 4.39)** | 0.935 | 44 | 1.14 (0.17, 7.53)** | 0.901 | 708 | 0.97 (0.22, 4.24)** | 0.97 |
| 752 | Nervous system congenital anomalies | 1170 | 482914* | 512 | 1.11 (0.76, 1.62)** | 0.592 | 156 | 1.12 (0.59, 2.13)** | 0.731 | 55 | 1.06 (0.14, 7.91)** | 0.957 | 1000 | 1.03 (0.3, 3.52)** | 0.966 |
| 752.1 | Neural tube defects | 113 | 482914* | 44 | 1.02 (0.42, 2.47)** | 0.97 | 20 | 1.53 (0.28, 8.32)** | 0.638 | 7 | 1.43 (0.02, 99.72)** | 0.877 | 100 | 1.34 (0.08, 21.91)** | 0.849 |
| 752.11 | Spina bifida | 305 | 482914* | 135 | 1.19 (0.48, 2.94)** | 0.714 | 51 | 1.5 (0.7, 3.21)** | 0.307 | 9 | 0.71 (0.02, 21.16)** | 0.853 | 260 | 1.01 (0.54, 1.91)** | 0.97 |
| 752.2 | Other specified congenital anomalies of nervous system | 825 | 482914* | 359 | 1.07 (0.53, 2.17)** | 0.864 | 98 | 0.96 (0.16, 5.67)** | 0.97 | 42 | 1.11 (0.21, 5.81)** | 0.91 | 704 | 1.02 (0.45, 2.29)** | 0.97 |
| 753 | Congenital anomalies of the eye | 706 | 482914* | 288 | 0.91 (0.49, 1.69)** | 0.771 | 83 | 0.91 (0.25, 3.29)** | 0.892 | 29 | 0.83 (0.12, 5.59)** | 0.861 | 603 | 1.05 (0.22, 4.91)** | 0.958 |
| 753.1 | Congenital cataract and lens anomalies | 233 | 482914* | 93 | 0.93 (0.17, 5.06)** | 0.935 | 30 | 1.03 (0.29, 3.58)** | 0.97 | 13 | 1.17 (0.04, 36.52)** | 0.933 | 193 | 0.85 (0.16, 4.43)** | 0.861 |
| 753.2 | Congenital anomalies of posterior segment of eye | 205 | 482914* | 82 | 0.96 (0.15, 6.17)** | 0.97 | 31 | 1.27 (0.25, 6.55)** | 0.788 | 10 | 1.07 (0.04, 30.46)** | 0.97 | 181 | 1.35 (0.32, 5.68)** | 0.695 |
| 754 | Congenital musculoskeletal deformities of spine | 664 | 482914* | 278 | 0.97 (0.28, 3.3)** | 0.959 | 73 | 0.92 (0.19, 4.55)** | 0.924 | 37 | 1.2 (0.22, 6.64)** | 0.845 | 549 | 0.87 (0.41, 1.85)** | 0.73 |
| 754.2 | Spondylolisthesis, congenital | 275 | 482914* | 116 | 0.95 (0.11, 8.52)** | 0.97 | 27 | 0.86 (0.06, 12.58)** | 0.92 | 17 | 1.36 (0.12, 15.78)** | 0.818 | 233 | 1.04 (0.16, 6.63)** | 0.97 |
| 755 | Congenital anomalies of limbs | 6557 | 482914* | 2722 | 0.97 (0.79, 1.19)** | 0.773 | 798 | 0.94 (0.74, 1.19)** | 0.607 | 298 | 0.93 (0.57, 1.53)** | 0.802 | 5478 | **0.88 (0.8, 0.97)**** | **0.013** |
| 755.1 | Congenital deformities of feet | 2423 | 482914* | 1002 | 0.97 (0.63, 1.48)** | 0.89 | 304 | 0.98 (0.38, 2.53)** | 0.968 | 111 | 0.95 (0.31, 2.91)** | 0.935 | 2042 | 0.94 (0.65, 1.34)** | 0.73 |
| 755.3 | Congenital anomaly of fingers/toes | 459 | 482914* | 176 | 0.88 (0.46, 1.7)** | 0.715 | 58 | 0.94 (0.13, 6.62)** | 0.956 | 30 | 1.31 (0.41, 4.23)** | 0.662 | 399 | 1.15 (0.4, 3.33)** | 0.809 |
| 755.4 | Congenital anomalies of upper limb, including shoulder girdle | 490 | 482914* | 207 | 1.04 (0.26, 4.17)** | 0.956 | 69 | 1.15 (0.33, 3.94)** | 0.838 | 21 | 0.93 (0.04, 22.58)** | 0.97 | 417 | 0.99 (0.72, 1.37)** | 0.97 |
| 755.6 | Other congenital anomalies of lower limb, including pelvic girdle | 496 | 482914* | 202 | 0.94 (0.37, 2.4)** | 0.909 | 70 | 1.1 (0.3, 4.04)** | 0.893 | 16 | 0.67 (0.18, 2.49)** | 0.558 | 412 | 0.86 (0.41, 1.79)** | 0.7 |
| 755.61 | Congenital hip dysplasia and deformity | 2823 | 482914* | 1184 | 0.97 (0.64, 1.47)** | 0.887 | 321 | 0.85 (0.66, 1.1)** | 0.231 | 120 | 0.86 (0.48, 1.53)** | 0.612 | 2328 | **0.81 (0.71, 0.94)**** | **0.004** |
| 756 | Other congenital musculoskeletal anomalies | 2194 | 482914* | 954 | 1.02 (0.5, 2.06)** | 0.97 | 252 | 0.9 (0.54, 1.51)** | 0.708 | 79 | 0.75 (0.44, 1.29)** | 0.307 | 1849 | 0.94 (0.54, 1.62)** | 0.83 |
| 756.1 | Congenital anomalies of abdominal wall; diaphram | 279 | 482914* | 127 | 1.09 (0.41, 2.92)** | 0.871 | 30 | 0.83 (0.24, 2.86)** | 0.784 | 8 | 0.6 (0.13, 2.82)** | 0.531 | 234 | 0.91 (0.25, 3.39)** | 0.898 |
| 756.21 | Pectus excavatum | 584 | 482914* | 258 | 1.06 (0.21, 5.29)** | 0.946 | 72 | 1 (0.86, 1.16)** | 0.97 | 19 | 0.69 (0.05, 9.84)** | 0.8 | 492 | 0.93 (0.13, 6.49)** | 0.943 |
| 756.22 | Pectus carinatum | 156 | 482914* | 64 | 0.87 (0.16, 4.87)** | 0.887 | 10 | 0.46 (0.11, 1.91)** | 0.289 | 11 | 1.33 (0.06, 28.76)** | 0.866 | 124 | 0.67 (0.26, 1.71)** | 0.406 |
| 756.3 | Congenital anomalies of muscle, tendon, fascia, and connective tissue | 316 | 482914* | 141 | 1.05 (0.21, 5.31)** | 0.958 | 36 | 0.9 (0.13, 6.37)** | 0.92 | 8 | 0.53 (0.09, 3.06)** | 0.487 | 266 | 0.93 (0.14, 6.06)** | 0.947 |
| 756.5 | Congenital osteodystrophies | 603 | 482914* | 270 | 1.04 (0.4, 2.74)** | 0.935 | 63 | 0.85 (0.34, 2.14)** | 0.742 | 21 | 0.74 (0.18, 3.07)** | 0.693 | 516 | 1.06 (0.3, 3.79)** | 0.935 |
| 757 | Congenital anomalies of the integument | 185 | 482914* | 82 | 1.22 (0.38, 3.84)** | 0.752 | 31 | 1.63 (0.66, 4.02)** | 0.297 | 7 | 0.96 (0.11, 7.97)** | 0.97 | 152 | 0.82 (0.18, 3.87)** | 0.818 |
| 758 | Chromosomal anomalies and genetic disorders | 1310 | 482914* | 555 | 1.02 (0.43, 2.44)** | 0.968 | 169 | 1.03 (0.33, 3.21)** | 0.959 | 58 | 0.95 (0.16, 5.49)** | 0.959 | 1102 | 0.93 (0.52, 1.65)** | 0.809 |
| 758.1 | Chromosomal anomalies | 1043 | 482914* | 434 | 0.99 (0.54, 1.81)** | 0.97 | 141 | 1.06 (0.43, 2.63)** | 0.901 | 41 | 0.83 (0.3, 2.33)** | 0.737 | 872 | 0.89 (0.56, 1.42)** | 0.644 |
| 759 | Other and unspecified congenital anomalies | 1021 | 482914* | 470 | 1.21 (0.97, 1.53)** | 0.096 | 135 | 1.16 (0.65, 2.08)** | 0.624 | 41 | 0.95 (0.09, 9.86)** | 0.966 | 876 | 1.06 (0.43, 2.6)** | 0.905 |
| 759.1 | Anomalies of endocrine glands, congenital | 136 | 482914* | 66 | 1.35 (0.55, 3.32)** | 0.524 | 16 | 1.16 (0.07, 20.23)** | 0.924 | 7 | 1.32 (0.04, 45.69)** | 0.886 | 113 | 0.88 (0.1, 8.18)** | 0.92 |

a. Statistically significant IRRs are marked with bold (FDR adjusted p-value<0.05).
b. The IRRs are adjusted for age, sex, interaction between age and sex, and birth year.
c. Blood group O and the RhD negative blood group was used as a reference, respectively.
d. The FDR adjusted p-values and 95% confidence intervals are presented.
e. FDR adjusted p-values above 0.97 were set to 0.97 to avoid exploding adjusted confidence intervals.
f. Phecodes are divided by PheWAS disease categories.
g. The number of events and the follow-up time in person-years for each Phecode is also presented.
h. For study results of congenital Phecodes estimates marked with ** are prevalence ratios instead of IRRs and the corresponding person-year marked with * are the size of the cohort.
